# Bespoke Activity-Based Probes Reveal that the *Pseudomonas aeruginosa* Endoglycosidase, PslG, Is an Endo-β-glucanase

**DOI:** 10.1021/jacs.4c16806

**Published:** 2025-02-25

**Authors:** Gijs Ruijgrok, Wendy A. Offen, Isabelle B. Pickles, Deepa Raju, Thanasis Patsos, Casper de Boer, Tim Ofman, Joep Rompa, Daan van Oord, Eleanor J. Dodson, Alexander Beekers, Thijs Voskuilen, Michela Ferrari, Liang Wu, Antonius P. A. Janssen, Jeroen D. C. Codée, P. Lynne Howell, Gideon J. Davies, Herman S. Overkleeft

**Affiliations:** † Leiden Institute of Chemistry, Leiden University, 2300 RA Leiden, The Netherlands; ‡ Department of Chemistry, The University York, Heslington, York YO10 5DD, United Kingdom; § Molecular Medicine, Research Institute, The Hospital for Sick Children, Toronto, Ontario M5G 0A4, Canada; ∥ Department of Biochemistry, University of Toronto, Toronto, Ontario M5S 1A8, Canada

## Abstract

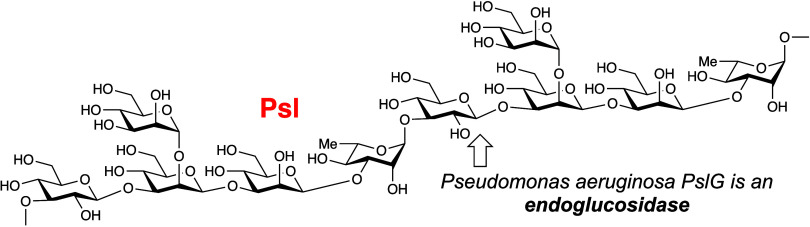

During infection, the human opportunistic pathogen Pseudomonas aeruginosa forms protective biofilms, whose matrix consists of proteins, nucleic acids, and polysaccharides such as alginate, Psl, and Pel. Psl, a polymeric pentasaccharide composed of mannose, rhamnose, and glucose, is produced during the early stages of biofilm formation, serving as a protective barrier against antibiotics and the immune system. The Psl biosynthesis gene cluster, besides encoding various glycosyltransferases, also includes an endoglycosidase, PslG. Here, we show, by activity-based protein profiling, structural studies on enzyme–inhibitor complexes, and defined substrate processing, that PslG is not, as previously suggested, an endo-β-mannosidase but instead a retaining endo-β-glucosidase. This insight allows the design of both competitive and covalent PslG inhibitors, as we show for repeating pentasaccharide mimetics featuring either a reducing end deoxynojirimycin or cyclophellitol moiety. This work provides valuable tools to deepen the understanding of Psl biosynthesis, its function in biofilm formation, and its contribution to antibiotic resistance. We demonstrate the enzyme’s actual endo−β–glucosidase activity, a means to monitor PslG activity in *P. aeruginosa* biofilms, and a blueprint for inhibitor design.

## Introduction

*Pseudomonas aeruginosa* is an opportunistic pathogen that causes life-threatening infections, particularly in cystic fibrosis patients, immunocompromised individuals, and burn victims.^1^*P. aeruginosa* is one of the ESKAPE pathogenic species and is particularly hard to combat because of its ability to encapsulate itself in almost impenetrable biofilms. *P. aeruginosa* biofilms contain, along with bacterial cells, proteins, and nucleic acids, up to three polysaccharides - alginate, Pel, and Psl - produced by distinct biosynthesis pathways.^2,3^

**Figure 1:**
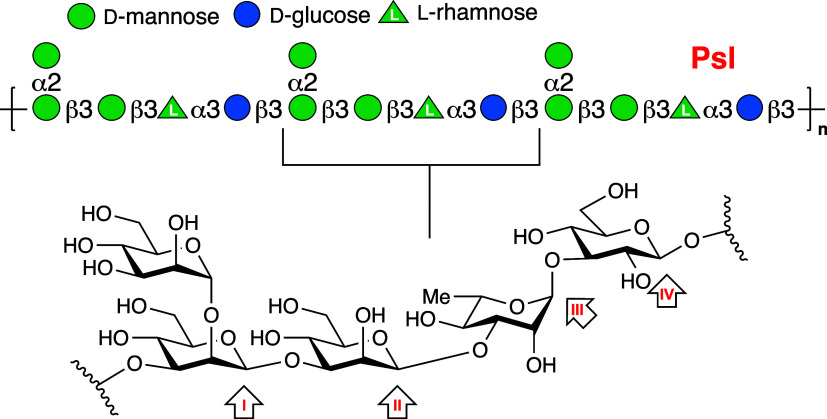
Psl structure and possible PslG cleavage sites (**I**–**IV**). Prior to these studies, PslG was tentatively assigned to be a β-endomannanase (hydrolysis at position **II**).

Alginate is a negatively charged, linear polysaccharide composed of β-1,4-linked d-mannuronic acids and l-guluronic acids, while Pel is a positively charged, linear polymer of α-1,4-linked *N*-acetyl-d-galactosamine and d-galactosamine. Psl, a neutral polysaccharide, is the most complex of the three exopolysaccharides. It is composed of d-mannose, l-rhamnose and d-glucose, assembled in the repeating, branched pentasaccharide: 2-*O*-(1,2-α-d-Man*p*)-1,3-β-d-Man*p*-1,3-β-d-Man*p*-1,3-α-l-Rham*p*-1,3-β-d-Glc*p* (Figure 1).^4^ Besides biosynthesis and transport genes, the gene clusters producing each of the three exopolysaccharides encode either a glycoside hydrolase (Pel, Psl) or a lyase (alginate).

This raises the question of whether polysaccharide processing is essential for *P. aeruginosa* survival and proliferation, and whether Pel and Psl hydrolases, similar to alginate lyase,^5^ could serve as targets for antibiotic development. Further insights into the role of PslG in *P. aeruginosa* biofilm formation, its potential as an antibiotic target as well as its effect on biofilm formation when administered exogenously would benefit from small-molecule enzyme activity modulators and reporters.^6−9^ The development of these requires insights into the mode of action and substrate specificity, which at the onset of these studies were known only in part. PslG was classified as a CAZY glycoside hydrolase (GH) 39 enzyme based on its amino acid sequence (CAZy) and its 3-D structure had been solved.^6^ This GH39 family is made up of retaining glycosidases acting on a variety of substrates including β-glucosides, β-galactosides and β-xylosides. Soaking of PslG crystals with mannose yielded structures with mannose bound in the active site.^6^ Ensuing docking studies^9^ showed the d-Glc*p*-1,3–2-*O*-(1,2-α-d-Man*p*)-β-d-Man*p*-1,3-β-d-Man*p* tetrasaccharide to align well within the enzyme active site with the reducing mannosides close to the putative active site residues (the nucleophile, Glu276 and the general acid–base, Glu165). Experimental and computational evidence suggested that PslG exhibits endo-β-mannanase activity, cleaving specifically after nonbranched mannose residues (Figure 1, position **II**).^9^ With this information in hand, we decided to develop an activity-based PslG probe, using a retaining glycosidase activity-based probe (ABP) design strategy based on the natural product retaining β-exoglucosidase inhibitor, cyclophellitol.^10,11^

Cyclophellitol resembles the transition-state structure of a β-d-glucopyranoside substrate when bound to the active site of a retaining β-exoglucosidase. This property makes it a valuable tool for probing enzyme mechanisms. Protonation of the epoxide by the general acid–base catalytic residue is then followed by epoxide displacement by the active site nucleophile, leading to a stable (as compared to the covalent, but transient acylal linkage that emerges during substrate processing) enzyme–inhibitor adduct. Elongating cyclophellitol with additional carbohydrate residues and modifying these, at the natural elongation side, with a reporter moiety (biotin, fluorophore, bioorthogonal tag) yielded effective and selective activity-based probes that report on various retaining β-endoglycosidases in the context of cell extracts, living cells and living animals.^11,12^

**Figure 2:**
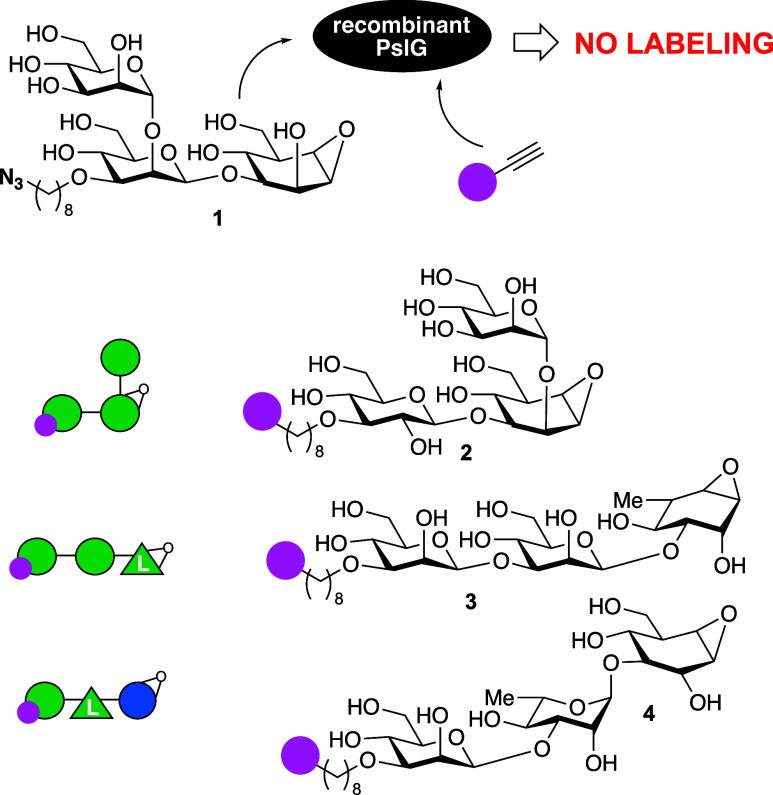
Reducing *manno*-cyclophellitol trisaccharide **1** failed to covalently and irreversibly modify recombinant PslG in a two-step, copper(I)-catalyzed azide–alkyne cycloaddition (CuAAC) ABPP experiment.^13^ This finding led to the design of ABPs **2**–**4** to assess whether PslG then processes Psl by cleaving at positions **I**, **III** or **IV** (Figure 1). The pink bulb represents a Cy5 dye (see for full structures the Supporting Information).

Here we present our development of cyclophellitol-derived activity-based probes and inhibitors for PslG. Using the cyclophellitol design principle, we developed trimannosidic ABP **1** (Figure 2) to emulate the structure of the tetrasaccharide from docking studies.^13^ This approach, however, did not produce an effective PslG probe. This was a surprising result, given the circumstantial evidence suggesting this enzyme to be a retaining mannosidase. In related studies on other retaining glycosidases, we almost invariably observed active site labeling by cyclophellitol-type ABPs configured and substituted to emulate the structure of the product produced from the substrate glycan by the retaining glycosidase (family) at hand.^11,12^ An alternative explanation is that PslG might not act as a retaining glycosidase. However, we considered it more likely that the substrate specificity had been mischaracterized. GH39 is a known retaining glycosidase family and with very few exceptions (for instance, CAZy family GH97, which contains both inverting and retaining enzymes^14^) mechanisms are conserved within a sequence (and hence structure) based family. Furthermore, neither the cocrystallization with mannose nor the docking with the tetrasaccharide appeared conclusive (and neither did the authors of these studies claim so).^6,9^ We synthesized three alternative trisaccharide ABPs, each with a different monosaccharide “cyclophellitol” at the reducing end (branched mannoside, rhamnoside, or glucoside). The results of these synthesis studies as well as the ability of the resulting probes (**2**–**4**) to label recombinant and *in situ* PslG are presented here. The selective reaction of PslG with ABP **4** identifies it as a retaining β-endoglucosidase. We confirmed this activity by demonstrating PslG-mediated digestion of a synthesized decasaccharide. Building upon probe **4**, we subsequently developed competitive and covalent PslG inhibitors, finding pentasaccharidic cyclophellitol to be significantly more potent than pentasaccharidic deoxynojirimycin, both of which share the Psl repeating pentasaccharide structure with the key glucose mimetic at the reducing end.

## Results

**Scheme 1:**
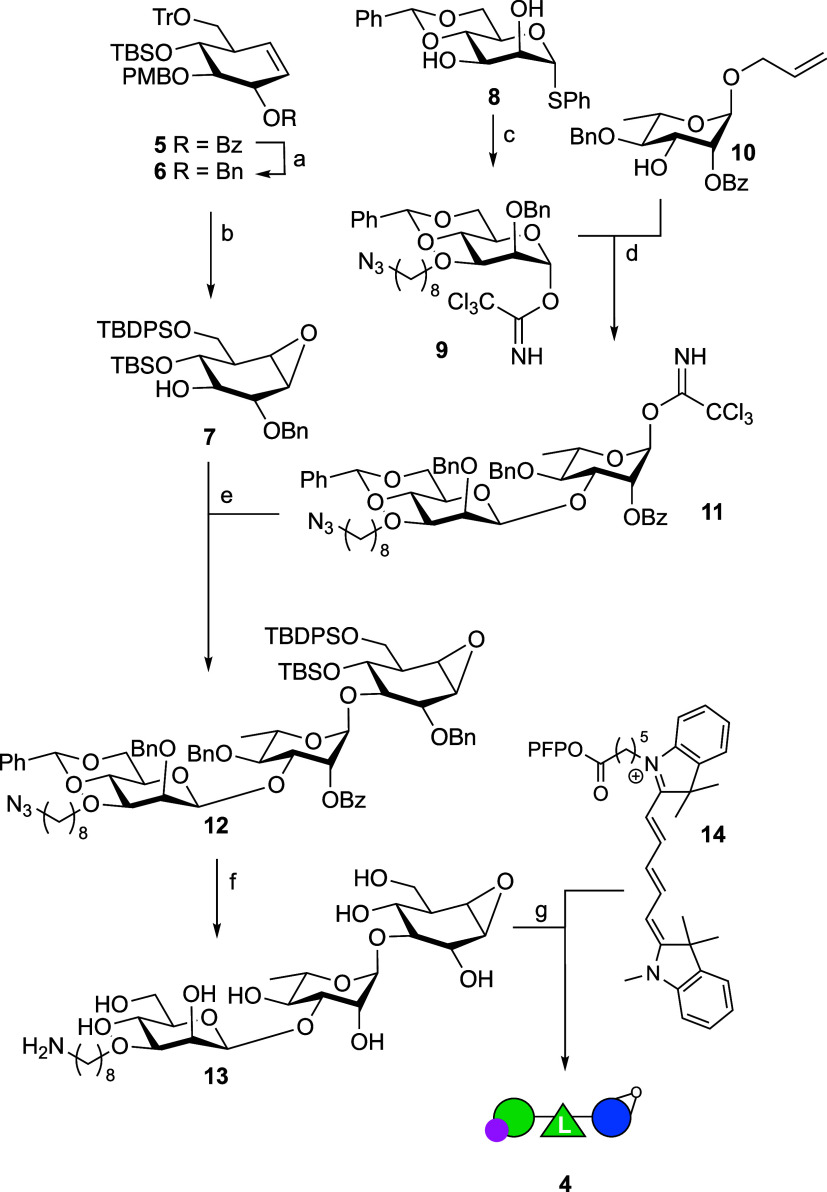
Reagents and conditions: (a) (i) NaOMe, MeOH; (ii) BnBr, NaH, DMF, 98%; (b) (i) TFA, TES-H, DCM, 63%; (ii) mCPBA, NaHCO_3_, DCM, 89%; (iii) TBDPS-Cl, imidazole, DMF, quant; (c) (i) 8-azido-1-octanyl triflate (generated *in situ*), 2-aminoethyl diphenyl borinate, K_2_CO_3_, DCM: MeCN 1:14; (ii) BnBr, TBAI, NaH, DMF, 81% over 3 steps; (iii) TFA, NIS, DCM, quant; (iv) trichloroacetonitrile, DBU, DCM, quant; (d) (i) TMSOTf, DCM, 3 Å MS, −80 °C -> −60 °C, 65%, α:β 1:3.4; (ii) {Ir(COD)[PCH_3_(C_6_H_5_)_2_]_2_}PF_6_, H_2_, THF; (iii) NIS, NaHCO_3_, H_2_O, 78%; (iv) trichloroacetonitrile, DBU, DCM, 73%; (e) TMSOTf, −40 °C, 63%; (f) (i) TBAF, THF (ii) Pt_2_O, H_2_; (iii) NH_3_, Na, *t*-BuOH, −60 °C, 51% over 3 steps; (g) **14**, DMF, DIPEA, H_2_O, 33%.

For ease of use we synthesized ABPs **2**–**4** (Figure 2) as direct probes equipped with a Cy5 dye at the nonreducing end at the carbon where the native polysaccharide extends. The pink bulb in the structures denote this conjugated Cy5 and the full chemical structure of all inhibitors and probes as well as full details of their synthesis and analysis are provided in the Supporting Information. Scheme 1, detailing the synthesis of probe **4**, provides a representative example of the synthesis strategy followed for the three ABPs as well as the nontagged cyclophellitol oligosaccharides that feature further on in this work. We have previously reported orthogonally protected cyclohexene **5** as a general precursor in the synthesis of cyclophellitol as well as configurational and substituted (glycosylated) analogues.^15^

Replacing the benzoyl at O2 (glucopyranose numbering) in **5** with a benzyl yielded fully protected cyclohexene **6**. Removal of the acid-labile trityl and para methoxybenzyl protective groups at O6 and O3, followed by treatment with mCPBA and silylating the O6, then gave with good selectivity partially protected cyclophellitol **7** that served as acceptor in an ensuing glycosylation with donor disaccharide **11** with a masked amine for final modification with a fluorophore. Donor **11** was prepared from donor trichloroacetimidate **9** (which had been prepared by regioselective alkylation of O3 in known phenylthiomannoside **8**, subsequent benzylation, thiophenol hydrolysis and trichloroacetimidate formation) and acceptor l-rhamnoside **10** in a stereoselective (as guided by the 4,6-benzylidene in **9**) β-mannosylation.^16^ Transforming the anomeric allyl in the resultant disaccharide into the trichloroacetimidate using standard functional group manipulations then yielded donor **11**, and ensuing stereoselective condensation with **7** gave fully protected trisaccharidic cyclophellitol **12**. Three-step global deprotection gave compound **13** that was then condensed with pentafluorophenol-activated Cy5 derivative **14** to give ABP **4**.

**Figure 3:**
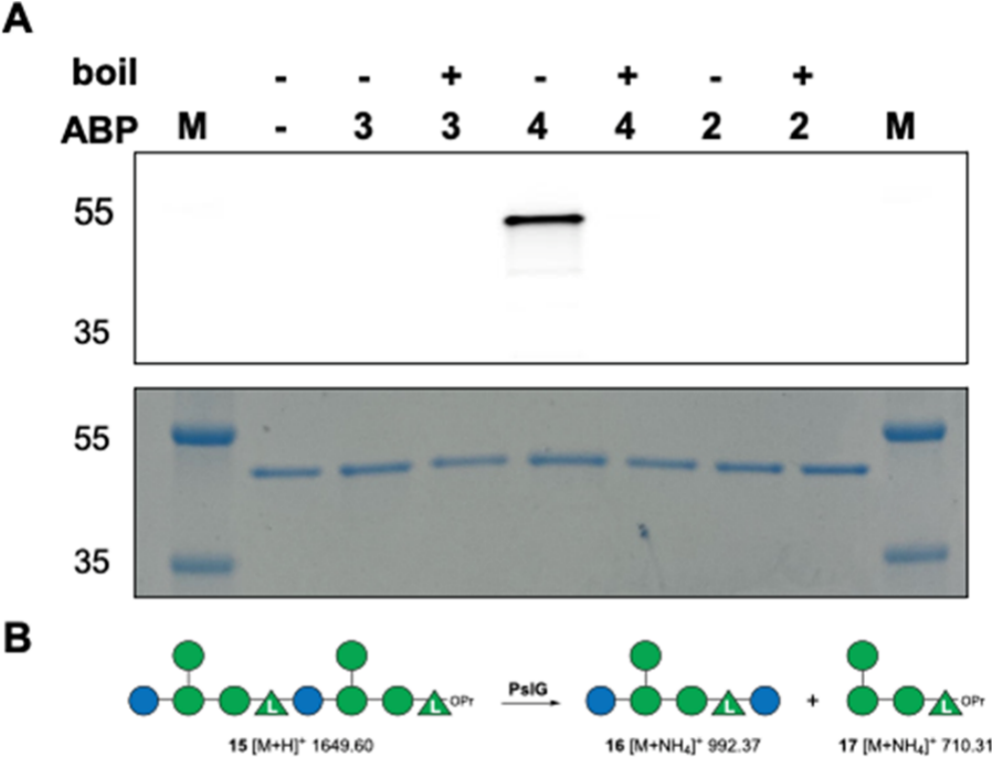
(**A**) Activity-based protein profiling of recombinant *P. aeruginosa* PslG with ABPs **3** (lanes 3, 4), **4** (lanes 5, 6) and **2** (lanes 7, 8). Denaturing of the sample by boiling is indicated above the gel. M: protein marker. Bottom: Coomassie stain of the same gel. (**B**) PslG-mediated processing of synthetic decasaccharide **15** (synthesis and full chemical structure given in the Supporting Information). OPr: Opropyl.

With the three ABPs (**2**-**4**) in hand, we then set out to assess their ability to react with recombinant PslG in a series of comparative and competitive ABPP experiments (Figure 3). Treatment of recombinant PslG with either of the three ABPs **2**-**4** followed by denaturing and resolving the protein mixture by SDS-PAGE and in-gel fluorescence scanning of the wet gel slabs revealed a clear fluorescent band (Figure 3A lane 5), at a height corresponding to the molecular weight of PslG, with ABP **4**, but not with ABPs **2** or **3**. Denaturing the protein sample by boiling prior to treatment with **4** (lane 6) gave no discernible fluorescent band, indicating that the PslG-ABP **4** reaction is dependent on enzyme activity.

To establish the substrate specificity of PslG, unambiguously, the synthetic^17,18^ Psl decasaccharide **15** featuring a nonreducing d-glucose and a reducing end l-rhamnose was digested enzymatically using PslG (Figure 3B; see for the synthesis and full chemical structure of compound **15** the Supporting Information). Treatment of **15** in 100 mM NH_4_OAc buffer with recombinant PslG for 24 h at 37 °C was followed by enzyme removal by filtration over a C18 stage tip. HRMS analysis of the resulting lyophilized and redissolved product gave hexamer **16** and tetramer **17** as the major products (shown are the [M+NH_4_]^+^ masses which are the predominant ion peaks observed besides [M + H]^+^ peaks, for the full MS spectrum see Figure S7), thus the products that emerge from glycoside hydrolysis at position **IV** (Figure 1). The MS trace also reveals some nonasaccharide product that emerges after removal of the nonreducing glucose in **15**, but no fragments that would emerge from hydrolysis of one of the glycosidic linkages at positions **I**–**III**. The results in Figure 3**AB** indicate that PslG exhibits endoglucosidase activity, contradicting the previous classification as an endomannosidase.

**Scheme 2:**
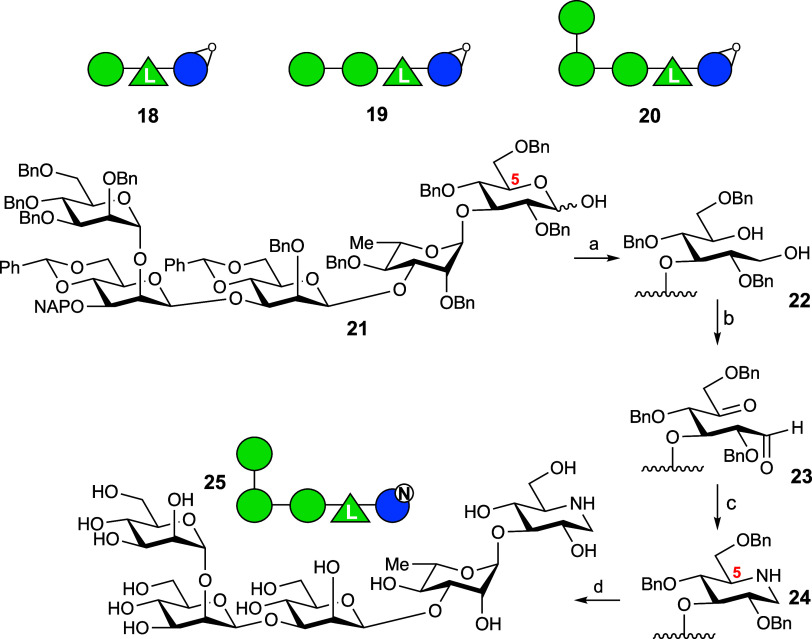
Structures of tri, Tetra- and Pentasaccharidic Cyclophellitols **18**–**20** and Key Steps in the Synthesis of Pentasaccharidic Deoxynojirimycin **25** (See for Full Details on the Synthesis of Protected Repeating Pentasaccharide **24** and that of Cyclophellitols **18**–**20** the Supporting Information) Reagents and Conditions: (a) LiAlH_4_, THF, 0 °C -> r.t., quant.; (b) oxalyl chloride, DMSO, Et_3_N, DCM, −78 to −10 °C; (c) HCO_2_NH_4_, NaCNBH_3_, Na_2_SO_4_, MeOH, 0 °C to r.t., 54% (two steps); (d) Na, NH_3_, *t*-BuOH, THF, −60 °C, 66%.

Having established that PslG is a retaining endoglucosidase, we then set out to synthesize and evaluate nontagged candidate-mechanism-based inhibitors **18**–**20** and putative competitive inhibitor **25** (Scheme 2).

Cyclophellitols **18**–**20** were designed to assess the influence of compound size on inhibitor potency, with compound **18** being the nontagged version of ABP **4**, compound **19** the 1-mannose homologue and compound **20** the 2-mannose homologue representing the full length of the Psl pentasaccharide repeat. These compounds were synthesized using routes like that of ABP **4** (see the Supporting Information). Pentasaccharidic iminosugar **25** was designed to investigate whether, like most retaining β-glucosidases, PslG would be inhibited by the appropriately configured deoxynojirimycin derivative. Compound **25** was synthesized using our previously reported procedure to transform monoglycosylated glucose derivatives into the corresponding deoxynojirimycin derivative.^19^ The key steps are shown in Scheme 2 (see for full experimental detail the Supporting Information) and comprise reduction of the reducing glucose hemiacetal in **21** into diol **22** and subsequent double Swern oxidation to give 5-ketoaldehyde **23**. Treatment of **23** with excess ammonium formate and sodium cyanoborohydride gives, after a double reductive amination, and with full retention of configuration at C5 of the reducing (imino)sugar, the fully protected pentasaccharidic iminosugar **24**. Birch reduction then yields iminosugar **25** in 35% overall yield over the four steps.

**Figure 4:**
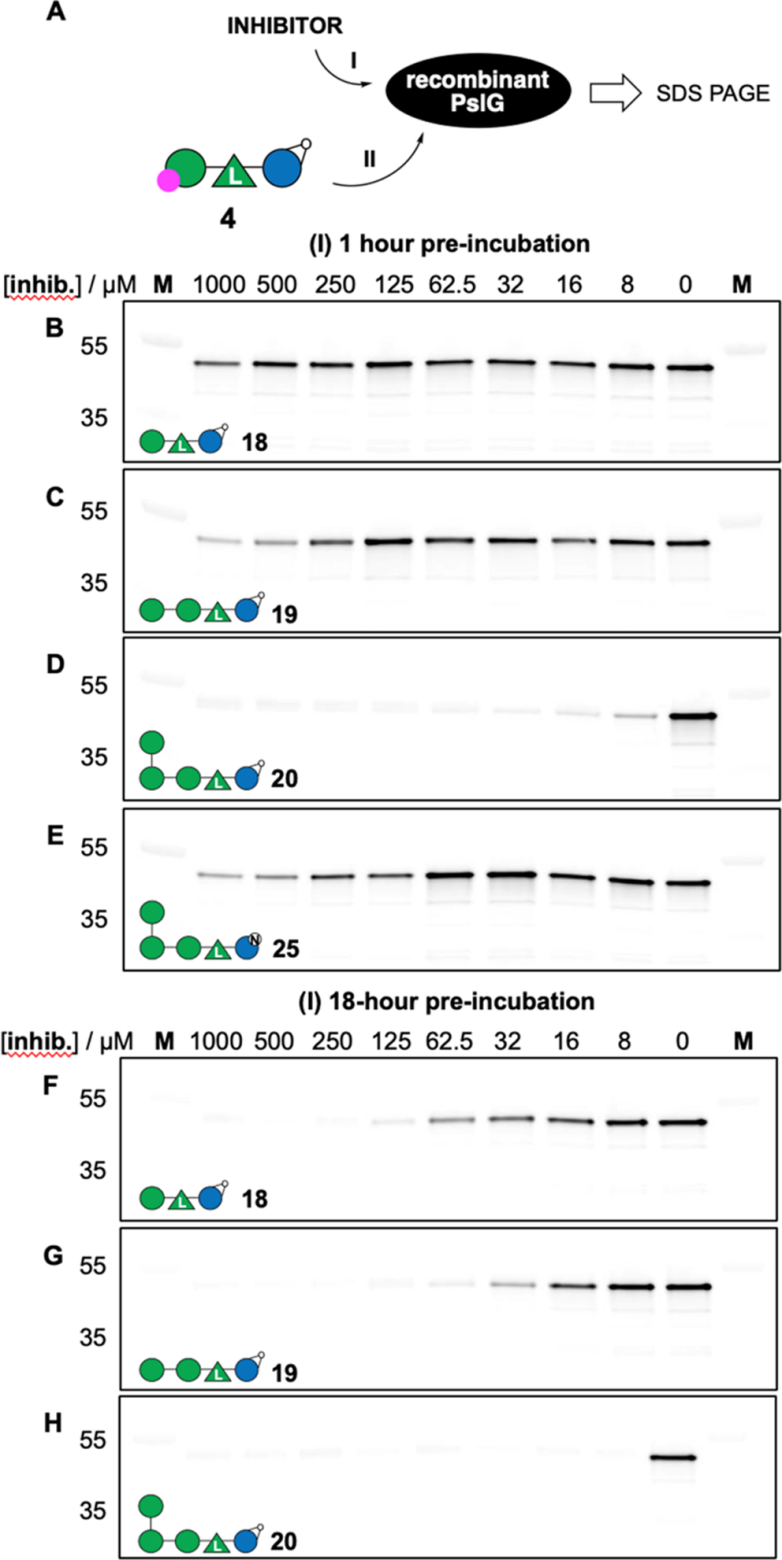
(A) Competitive ABPP workflow. (**B**–**H**) Concentration- and time-dependent inhibition of ABP **4** labeling of recombinant PslG. Lanes **B**-**E**: 1 h incubation with inhibitors **18** (B); **19** (C); **20** (D); and **25** (E). Lanes **F**–**H**: 18 h incubation with inhibitors **18** (F); **19** (G); and **20** (H). Full fluorescence and Coomassie-stained gels are shown in the Supporting Information.

**Figure 5:**
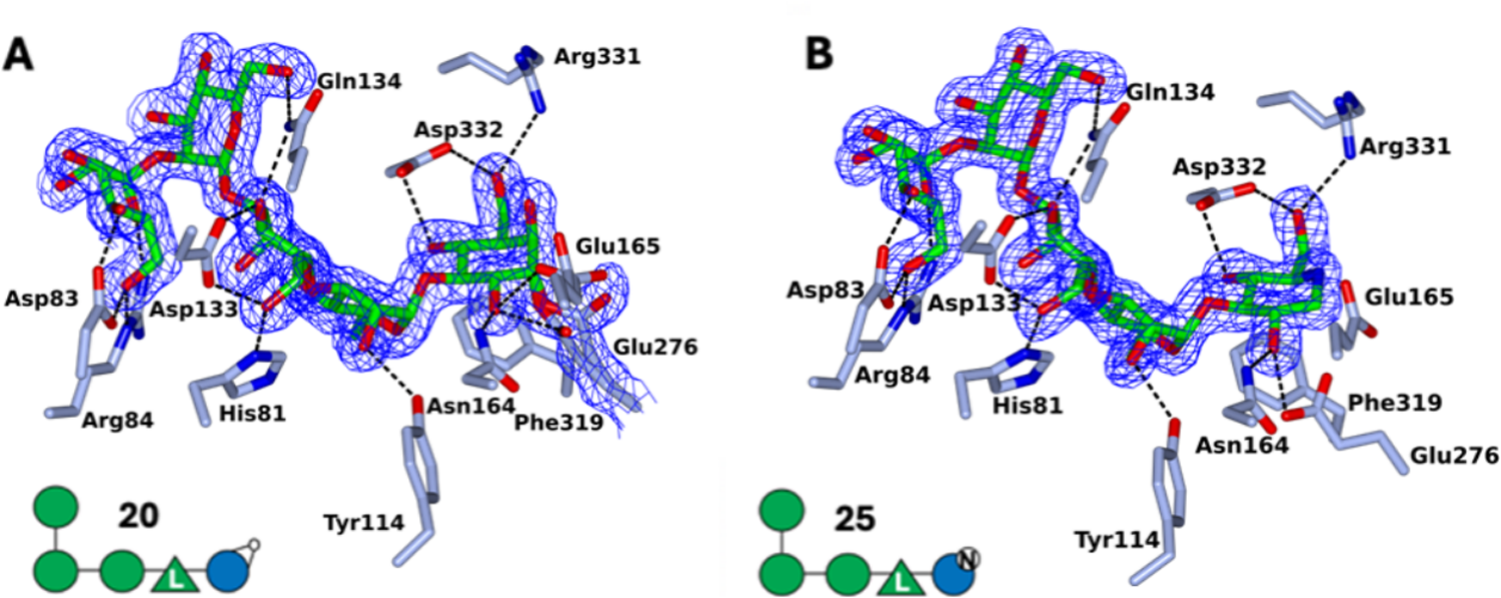
Structure of PslG complexed to (A) **20** and (B) **25** showing ligand omit electron density *F*_*obs*_–*F*_*calc*_ map contoured at 3 rmsd.

Compounds **18**–**20** and **25** were then subjected to a competitive ABPP experiment following the scheme depicted in Figure 4A. Thus, incubation (step I) of recombinant PslG with putative inhibitor at varying concentration and time is followed, after 1 or 18 h, by addition (step II) of ABP **4**, a further 30 min incubation, and then denaturation of the protein samples, SDS PAGE separation and in-gel fluorescence scanning. Inhibition potency is then reversibly proportional to compound concentration-dependent fluorescence intensity. As can be seen, trisaccharidic cyclophellitol **18** (Figure 4B) and tetrasaccharidic cyclophellitol **19** (Figure 4C) block ABP **4** labeling after 1 h preincubation at 500 μM to 1 mM final concentration, whereas pentasaccharidic cyclophellitol **20** blocks labeling at 8 μM final concentration (Figure 4D). Extending the preincubation time to 18 h improves PslG inactivation for all three compounds, with pentasaccharidic cyclophellitol **20** again being the most potent inhibitor (Figure 4F–H). Pentasaccharidic deoxynojirimycin **25** showed significant inhibition at 500 μM in a 1 h preincubation experiment (Figure 4D) validating this iminosugar as a bona fide competitive PslG inhibitor. We did not perform 18 h preincubation experiments with **25** on the grounds that this is a competitive inhibitor and does not show time-dependent inhibition. We aimed to elucidate PslG substrate specificity and better interpret chain-length effects in competition assays. To this end, we obtained X-ray structures of PslG complexed with pentasaccharidic cyclophellitol **20**, the most potent inhibitor. In the first instance, soaking PslG crystals with **20** using conditions adapted from Yu et al.^9^ proved abortive. We attempted to cocrystallize **20** with PslG and unexpectedly succeeded using batch crystallization. Crystals formed in an Eppendorf tube containing a mixture of 100 μL PslG (10.7 mg/mL in 20 mM MES, pH 6.0, 50 mM NaCl) and 10 μL **20** (20 mM in water) after incubation at room temperature for 1 day, followed by storage at 4 °C (see Supporting Information for experimental details).These crystals yielded a structure at a resolution of 1.55 Å with **20** reacted with Glu276 (the postulated nucleophile) and with Glu165 (the postulated acid–base) positioned nearby (Figure 5A). The side chain of Glu276 lies in a different position to that in the previously published structures^6,9^ and occupies two different conformations, both covalently bound to C1 of the ring-opened epoxide. The −1 to −3 subsite sugars are bound in a cavity in the active site, while the −4 and −5 subsite mannose sugars lie at the exterior face of the protein molecule. Atypically, compared to many oligosaccharide binding sites in glycoside hydrolases, there is just a single aromatic stacking interaction with **20** which is between the reacted cyclophellitol and Phe319. The −2 and −4 subsite sugars form single hydrogen bonds to amino acid side chains (Tyr114 and Gln134 respectively), while the other sugar moieties are tethered by several hydrogen bonding interactions: the epoxide-opened cyclophellitol to Arg331, Asp332 and Asn164, mannose (−3) to Asp133, His81 and Gln134, and the α-1,2-linked mannose (−5) to Asp83 and Arg84.

Having established suitable cocrystallization conditions for PslG and PslG-**20**, we then tried these with PslG and pentasaccharidic DNJ **25** and obtained crystals diffracting to 1.50 Å revealing a structure with **25** occupying the same binding site. The sugar residues at sites −2 to −4 feature essentially the same conformation and side chain interactions as those in the PslG-**20** structure. The −1 deoxynojirimycin moiety, like the ring-opened cyclophellitol, lies close to the active site residues, and forms a hydrogen bond between O2 and the Glu276 side chain.

Next, we examined whether ABP **4** was able to label and visualize PslG in its natural environment. We used the following *P. aeruginosa* strains: (i) PAO1 Δ*pelF*P_BAD_*psl* in which Psl is exclusively produced upon induction with arabinose; (ii) PAO1 Δ*pelF*P_BAD_*psl*Δ*pslG*, a PslG knockout strain in the same background and (iii) PAO1 Δ*pelF*P_BAD_*psl*Δ*pslG*::pPSV(*pslG)*, the *pslG* deletion strain complemented *in trans* with *PslG.*

**Figure 6:**
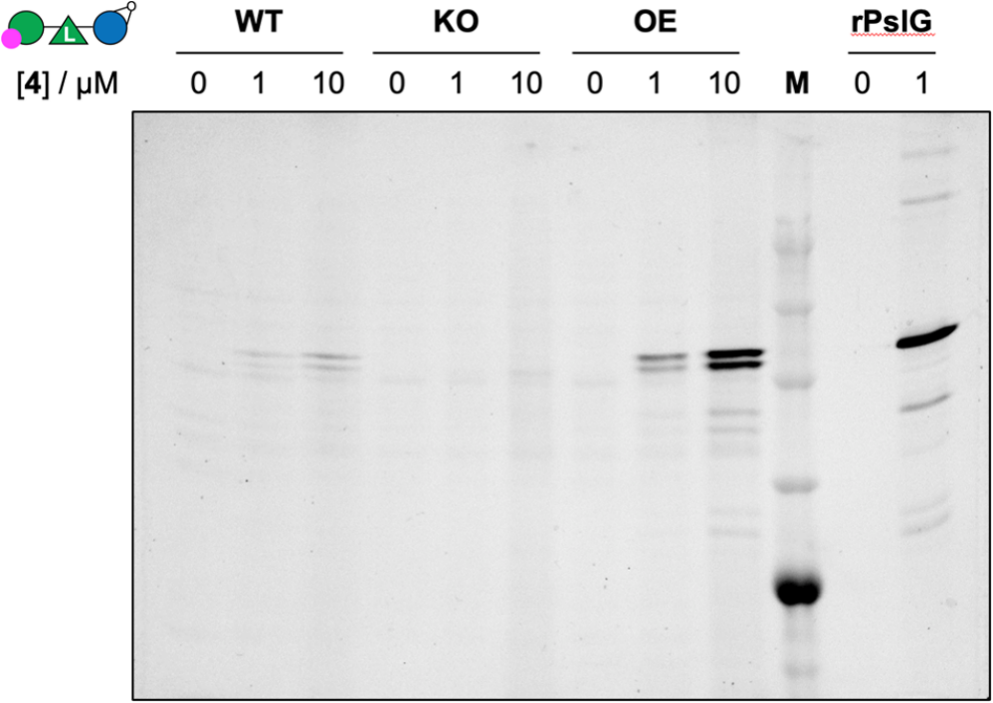
PslG labeling of whole bacteria. Samples are treated with ABP **4** at various concentrations for 45 min. Samples are then washed twice to remove excess probe, then lysed, protein samples denatured, resolved on SDS PAGE and the resulting wet gel slab scanned for fluorescence. Lanes 1–3: extracts of wild-type (WT) *P. aeruginosa* PAO1 strain treated with 0 (lane 1), 1 (lane 2) or 10 (lane 3) μM ABP **4**. Lanes 4–6: extracts of ΔPslG *P. aeruginosa* PAO1 strains (KO) treated with 0 (lane 4), 1 (lane 5) or 10 (lane 6) μM ABP **4**. Lanes 7–9: extracts of *P. aeruginosa* PAO1 strain overexpressing PslG (OE) treated with 0 (lane 7), 1 (lane 8) or 10 (lane 9) μM ABP **4**. Lane 11, 12: recombinant PslG treated (lane 12) or not (lane 11) with ABP **4**. M: protein marker. See for the Coomassie-stained gel the Supporting Information.

Overnight cultures of the strains were normalized to OD_600_ and treated with ABP **4** at 0, 1, or 10 μM final concentration at 37 °C for 45 min. Cells were then washed with PBS twice to remove excess probe prior to cell lysis. Fluorescence scans of SDS PAGE gels of the bacterial lysates (Figure 6) reveal ABP **4**-dependent fluorescent protein bands in the experiments using both the parental wild-type (lanes 2, 3) and complemented strains (lanes 8, 9), but not the knockout strain (lanes 5, 6). No fluorescent bands were visible in the absence of probe (lanes 1, 4 and 7) and the efficiency of binding was dependent on the concentration of ABP **4**. The highest intensity bands were observed in the complemented ΔpslG deletion mutant. This was anticipated as *in trans* complementation using the pPSV plasmid leads to increased expression of PslG relative to the parental strain (Figure S7). The absence of a signal in the Δ*pslG* strain indicates the specificity of the probe for PslG. As a control we also incubated recombinant, purified PslG with and without ABP **4** (lane 11). The fluorescent signal for the positive control (rPslG+probe) runs at the same molecular weight as the bands observed for PslG in the *P. aeruginosa* strains, albeit in this case two bands are observed. Multiple bands are also seen in the Western blot of the *P. aeruginosa* strains using a PslG specific antibody (Figure S7b). We speculate these closely resolved bands reflect PslG in two different but still probe sensitive isoforms. The two isoforms are likely mature periplasmic PslG and a cytoplasmic form of the protein where the signal peptide has not been processed correctly. Together these data demonstrate the capacity of probe **4** to report on the presence of PslG by ABPP in P. aeruginosa strains.

## Discussion

At the onset of the work described here, the *P. aeruginosa* endoglycosidase, PslG, was considered an agent to enhance *P. aeruginosa* susceptibility toward existing antibiotics. Conversely it was also a putative target for antibiotics itself^7,20^ We reasoned research in both directions would benefit from a PslG-active and selective ABP and set out to develop such a commodity. We based our first probe design on existing information on the substrate specificity of PslG, which had tentatively been assigned^6,9^ to be an endo-β-mannanase, even though we realized it is exceedingly rare to find β-mannosidases in the same CAZY family as enzymes active on *gluco*-configured substrates given the steric recognition of the axial versus equatorial O2 substituent. ABP **1** proved not to react with PslG and therefore we expanded the repertoire of probes to include other elements of the Psl oligosaccharide and in doing so unveiled the actual PslG cleavage cite.

We found that PslG is an endo-β-glucanase, a result we corroborated by digesting Psl decasaccharide **16**. Although starting with the Psl decasaccharide could have revealed PslG specificity, it would not have enabled us to monitor enzyme activity in its natural environment. We demonstrate that ABP **4** allows this, as we reveal by reporting on PslG activity in *P. aeruginosa* with endogenous and overexpressed PslG levels.

Probe **4**, unlike probes **1**–**3**, covalently and irreversibly modified PslG, revealing its glycosidic bond cleavage specificity. However, the exact number of substrate monosaccharides accommodated in the active site remained unclear. We addressed this in part by the synthesis and evaluation of cyclophellitol trisaccharide **18**, tetrasaccharide **19** and pentasaccharide **20**. Of these, the pentasaccharidic inhibitor proved by far the most potent in a competitive ABPP comparison. This pentasaccharide motif likely resembles the natural substrate recognized by PslG, in the negative subsites. Furthermore, literature speculations point toward possible Psl monosaccharide modifications which are lost during isolation of this polysaccharide and that would find interactions within the PslG binding pocket.^18^ Perusal of our inhibitor **20**-bound PslG structure reveals possible space for such modifications, and it may well be that inhibitors with enhanced potency can be developed by taking into consideration additional monosaccharides and modifications thereof. Machine learning algorithms could help identify additional interactions not visible in cocrystal structures by modeling PslG and its substrates. In such an approach the accuracy in modeling known (as seen in the crystal structures) enzyme–substrate interactions would indicate accuracies of new ones, which may include sugar modifications as well as saccharides extending from the +1 site. To probe the potential of this we performed some initial experiments, in which we took the online molecular structure prediction tool, Chai-1^21^ to which we subjected the PslG primary sequence together with substrates varying in sequence as well as our inhibitors (see Supporting Information for more details). In general, the structure of the glucose (mimetic) and rhamnose residues at the −1 and −2 sites were recapitulated well in these calculations, which however did not return the X-ray-observed structures of the (branched) mannose residues at −3 and −4. The program did not provide realistic structures of residues binding at +1 and further + sites but given the speed with which computational structural biology develops we deem it likely that future installments of this or related algorithms will provide such data–which is hard to obtain by experimental structural biology but for which structures such as ours may be needed to train and validate the algorithms.

Besides covalent inhibition, PslG is amenable to competitive inhibition, as is demonstrated by pentasaccharidic deoxynojirimycin **25** which, though less effective than **20**, competes for probe **4** binding. Compound **25** binds to PslG with the same occupancy as **20** in the crystal structures and adopts, apart from forming a covalent adduct, virtually the same conformational pose. The relatively poor, compared to **20**, inhibition potency as exerted by **25** is somewhat surprising. Possibly we have not identified the optimal inhibitor blueprint, or perhaps the assay format (competitive ABPP, featuring a noncovalent inhibitor competing for the PslG active site with a covalent and irreversible inactivator) disfavors compound **25**.

Recent in-depth research showed that lack of PslG has a large impact on bacterial and biofilm development.^8^ However, further research is needed, to unearth the specific role of PslG in *P. aeruginosa* infections, in Psl production, in biofilm formation and in antibiotic resistance. Our reagents and tools and the insights we have obtained with these may support such research. A fluorogenic substrate may for instance shed light on enzyme kinetics and such a substrate can now be designed based on the established substrate specificity: PslG is without doubt an endo-β-glucanase. Screening for PslG inhibitors, their activity, their selectivity and their target engagement potential can also be facilitated by wielding various ABPP formats, now that an effective and selective PslG ABP has become available. Further research is required to evaluate the in vivo activity of our probe, both in bacterial cultures and in host tissues during infection, to clarify the biological role of PslG in P. aeruginosa pathophysiology. Future studies will expand to other biofilm systems. These include the design of probes for glycoside hydrolases hydrolyzing Pel, which themselves are also retaining endoglycosidase. ABPs for alginate lyase, a mechanistic class for which no probe designs exist yet, are currently also being explored.

## Supporting Information Available

Methods for gene expression, protein purification, biochemical assays, structural biology, and complete synthetic protocols; atomic coordinates and structure factors deposited in the Protein Data Bank (PDB ID codes: 9G17, 9G18); NMR experiments, LC–MS experiments, enzyme kinetics experiments, full-length SDS-page gels, and additional structures; and details on crystal structure data collection and refinement (PDF)
