# Supplementary material for: Bespoke Activity-Based Probes Reveal that the Pseudomonas aeruginosa Endoglycosidase, PslG, Is an Endo-β-glucanase
Source: J Am Chem Soc. 2025 Feb 25;147(10):8578–86. doi: 10.1021/jacs.4c16806 (PMC11912335; doi:10.1021/jacs.4c16806)

Bespoke activity-based probes reveal that the *Pseudomonas aeruginosa* endoglycosidase, PslG, is an endo-β-glucanase

Gijs Ruijgrok[#], Wendy A. Offen[&], Isabelle B. Pickles[&], Deepa Raju[$], Thanasis Patsos[#], Casper de Boer[#], Tim Ofman[#], Joep Rompa[#], Daan van Oord[#], Eleanor J. Dodson, Alexander Beekers[#], Thijs Voskuilen[#], Michela Ferrari[#], Liang Wu[&], Antonius P. A. Janssen[#], Jeroen D. C. Codée[#], P. Lynne Howell[$,^], Gideon J. Davies[&*], Herman S. Overkleeft[#*]

[#]Leiden Institute of Chemistry, Leiden University, 2300 RA, Leiden, The Netherlands
[&]Department of Chemistry, The University York, Heslington, York YO10 5DD, United Kingdom
[$]Molecular Medicine, Research Institute, The Hospital for Sick Children, Toronto, Ontario, Canada
[^]Department of Biochemistry, University of Toronto, Toronto, Ontario, Canada

Table of Contents

Supplementary Figures **2**
Figure S1 pH profile of probe **4** labelling of recombinant PslG in McIlvaine and HEPES buffer 2
Figure S2 Full fluorescence and Coomassie gels of Figure 4B-D 3
Figure S3 Full fluorescence and Coomassie gels of Figure 4E 3
Figure S4 Full fluorescence (left) and Coomassie (right) gels of Figure 4F-H 4
Figure S5 Overlay of structures of PslG with **20** and **25** 5
Figure S6 Ribbon diagram of PslG structure bound to **20** 5
Figure S7 Corresponding Coomassie gel (A) and western blot (B) of Figure 6 6
Figure S8 MS analysis of decasaccharide **10** digestion by PslG 7
Figure S9 Chai-1-generated structures of PslG complexed to some substrates and inhibitors 8

Biochemistry Methods **9**
Recombinant protein production and purification 9
ABPP assays on recombinant PslG 9
Decasaccharide digestion 9
Crystallization, data collection and data refinement 9
ABPP on bacterial cells 10

Chai-1 structure prediction **10**

Compound Synthesis **11**

References **59**

NMR spectra **61**

Supplementary Figures

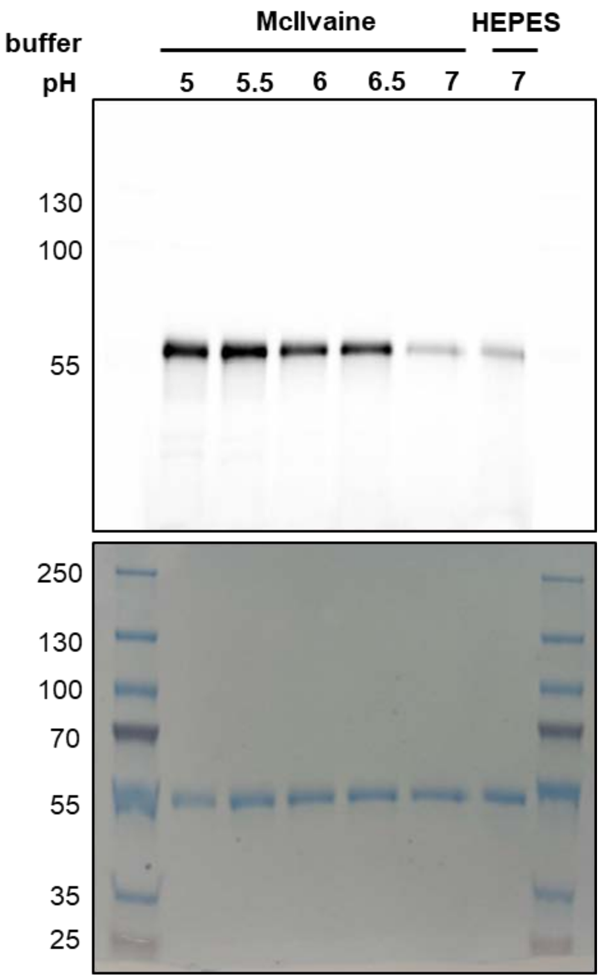

Figure S1. pH profile of probe **4** labelling of recombinant PslG in McIlvaine and HEPES buffer.

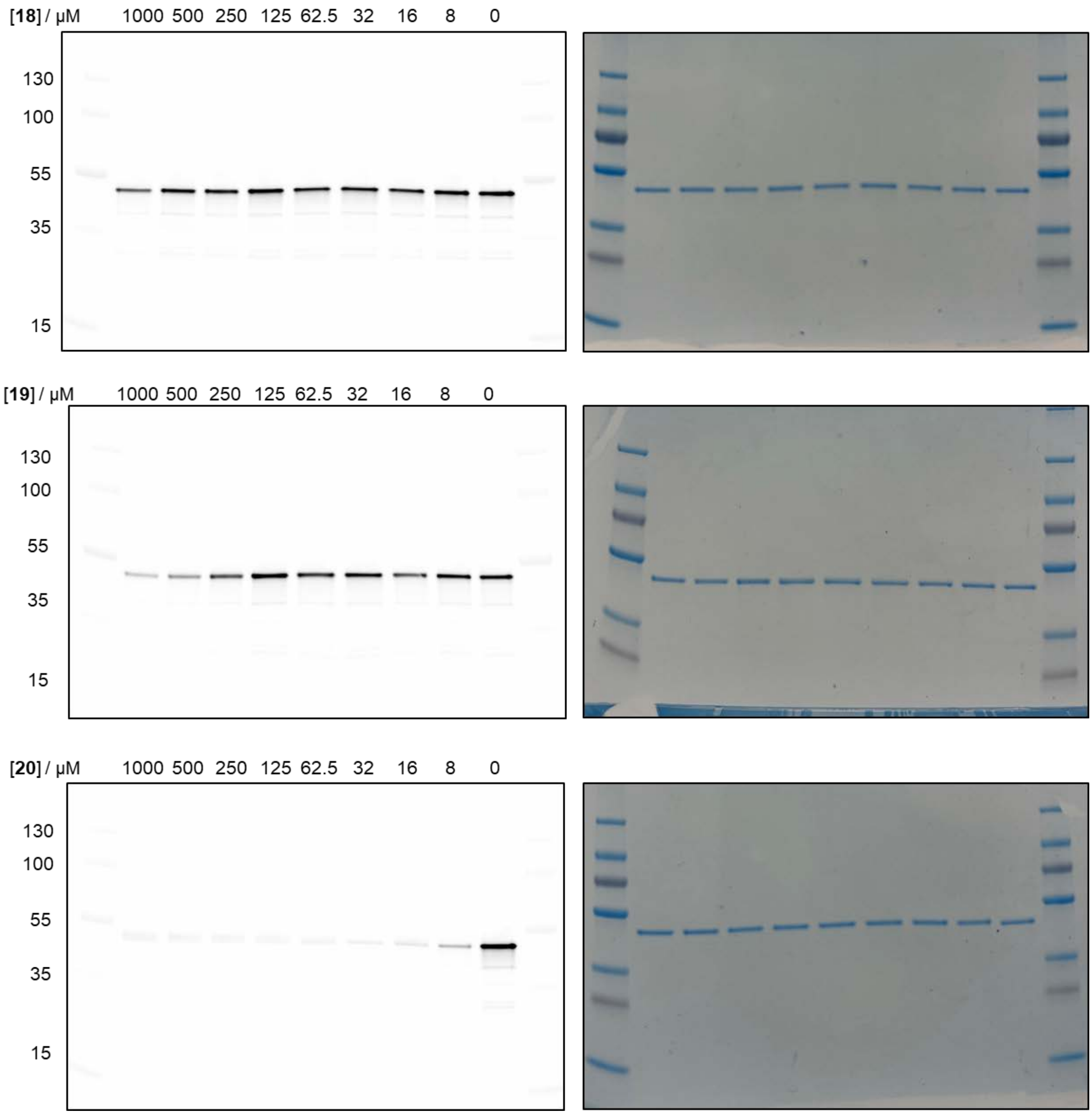

Figure S2. Full fluorescence (left) and Coomassie (right) gels of Figure 4B-D. Enzyme was pre-incubated at pH 5.5 with inhibitor compound for 1 hour before treatment with probe **4**.

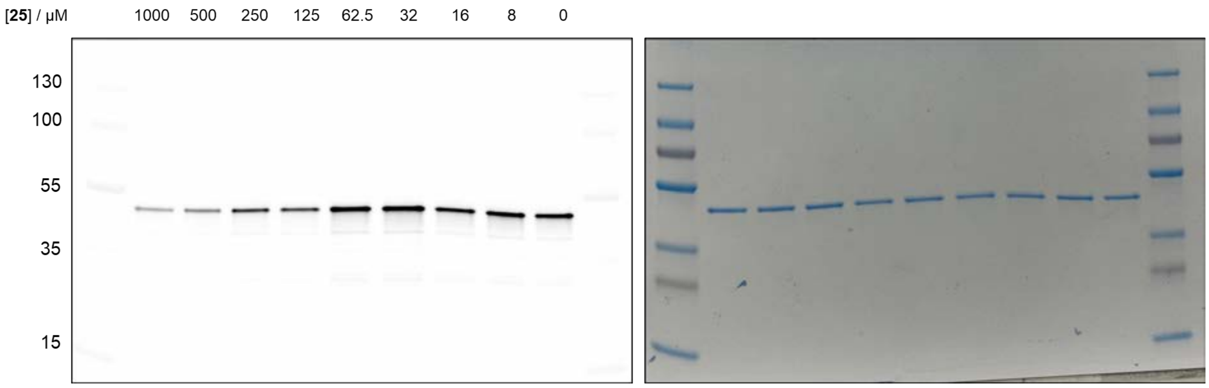

Figure S3. Full fluorescence (left) and Coomassie (right) gels of Figure 4E. Enzyme was pre-incubated at pH 5.5 with inhibitor **25** for 1 hour before treatment with probe **4**

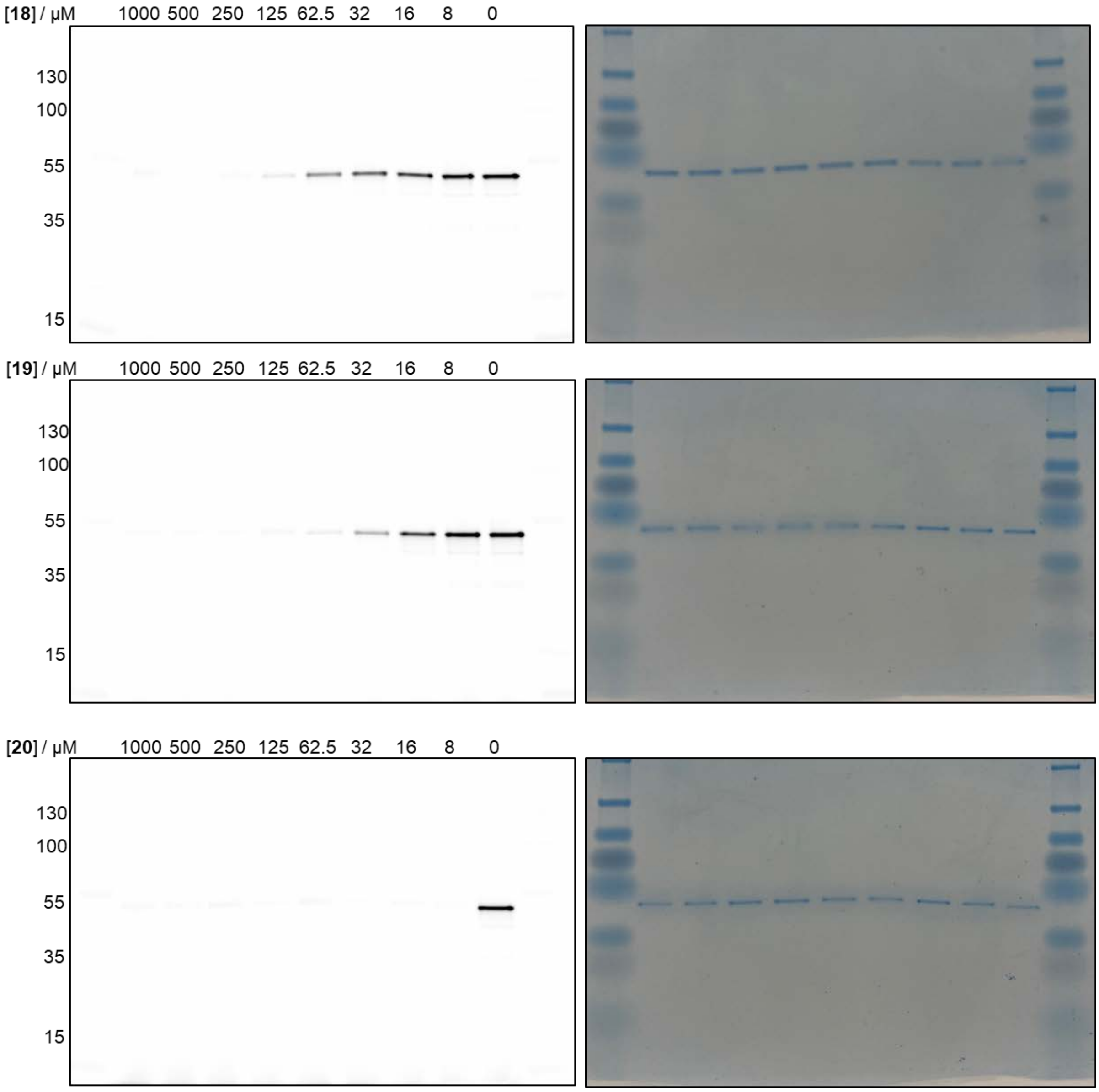

Figure S4. Full fluorescence (left) and Coomassie (right) gels of Figure 4F-H. Enzyme was pre-incubated at pH 5.5 with inhibitor compound for 18 hours before treatment with probe **4**.

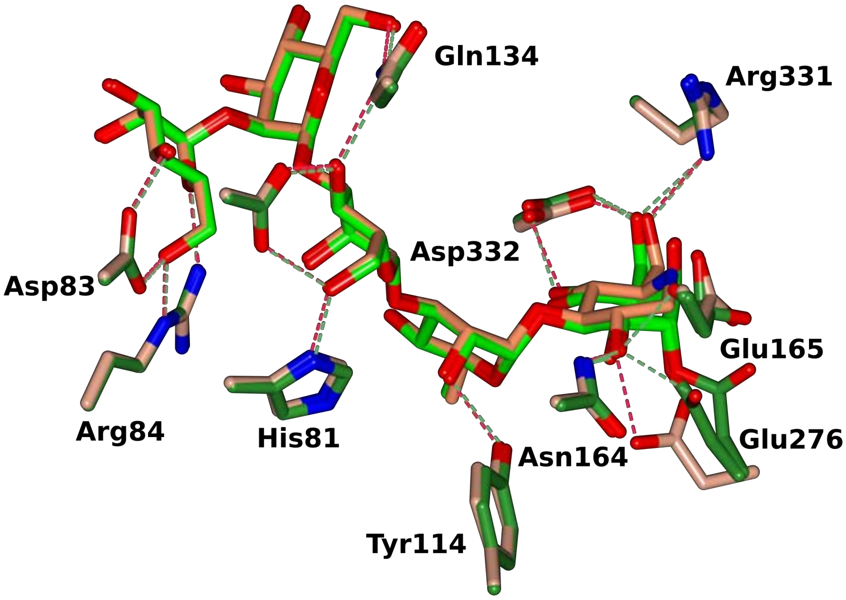

Figure S5. Overlay of structures of PslG with **20** and **25** showing ligands and neighboring side chains. C atoms are shown in green and coral. and hydrogen bonds as dashed lines in green and red, for **20** and **25** respectively. Figure made with *CCP4mg*.[1]

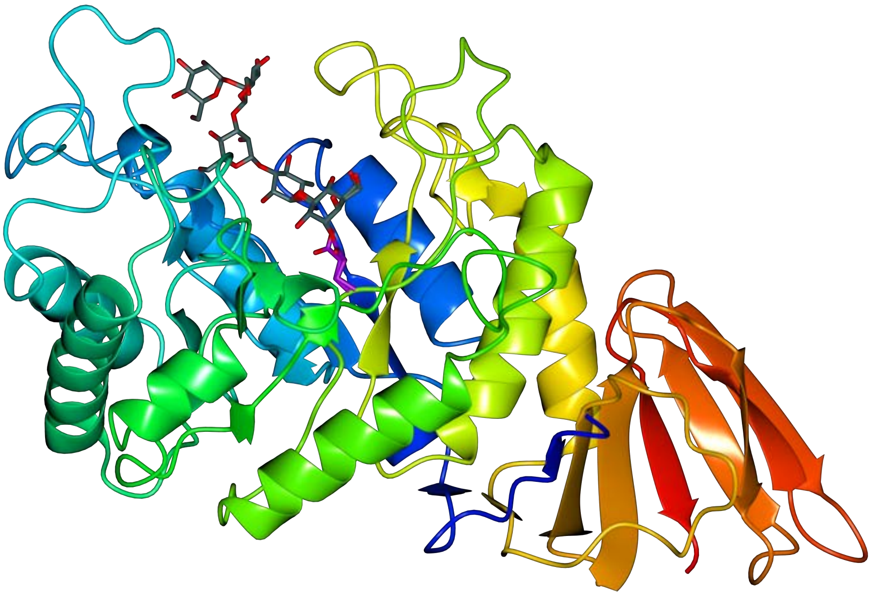

Figure S6. Ribbon diagram for PslG color ramped from blue at the N-terminus to red at the C-terminus. Ligand **20** is shown with C atoms in slate grey, covalently linked to the side chain of Glu276 (with C atoms in purple).

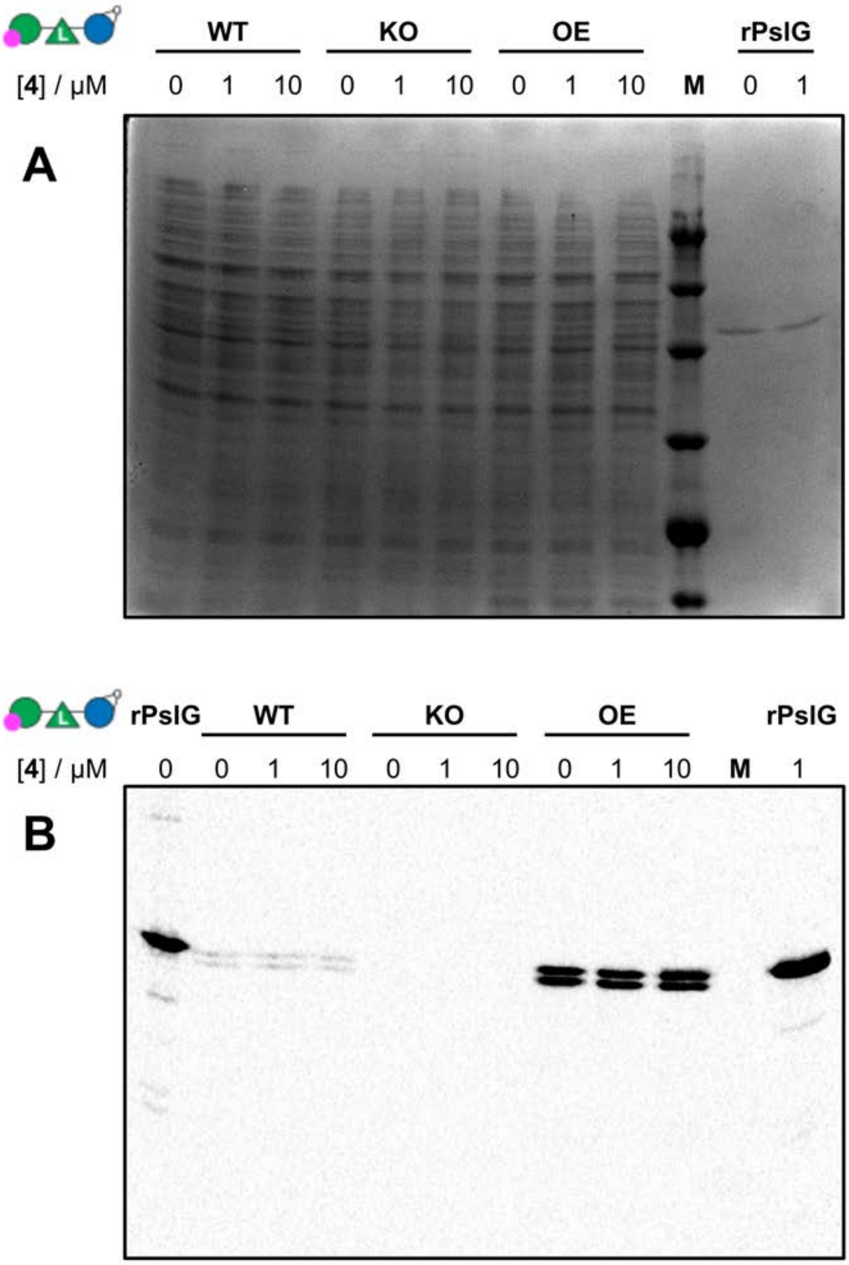

Figure S7. (**A**) Corresponding Coomassie gel of Figure 6. Cells were treated with ABP **4** for 45 minutes, then lysed, protein samples denatured, resolved on SDS PAGE and the gel was stained with Coomassie blue after being scanned for fluorescence. (**B**) Western blot of samples from Figure 6. Lysed samples from bacteria treated with ABP were resolved by SDS PAGE and subjected to Western blotting. The membranes were probed with a primary anti PslG antibody followed by an anti-rabbit secondary antibody tagged to horse radish peroxidase. The PslG specific bands were detected by chemiluminescence.

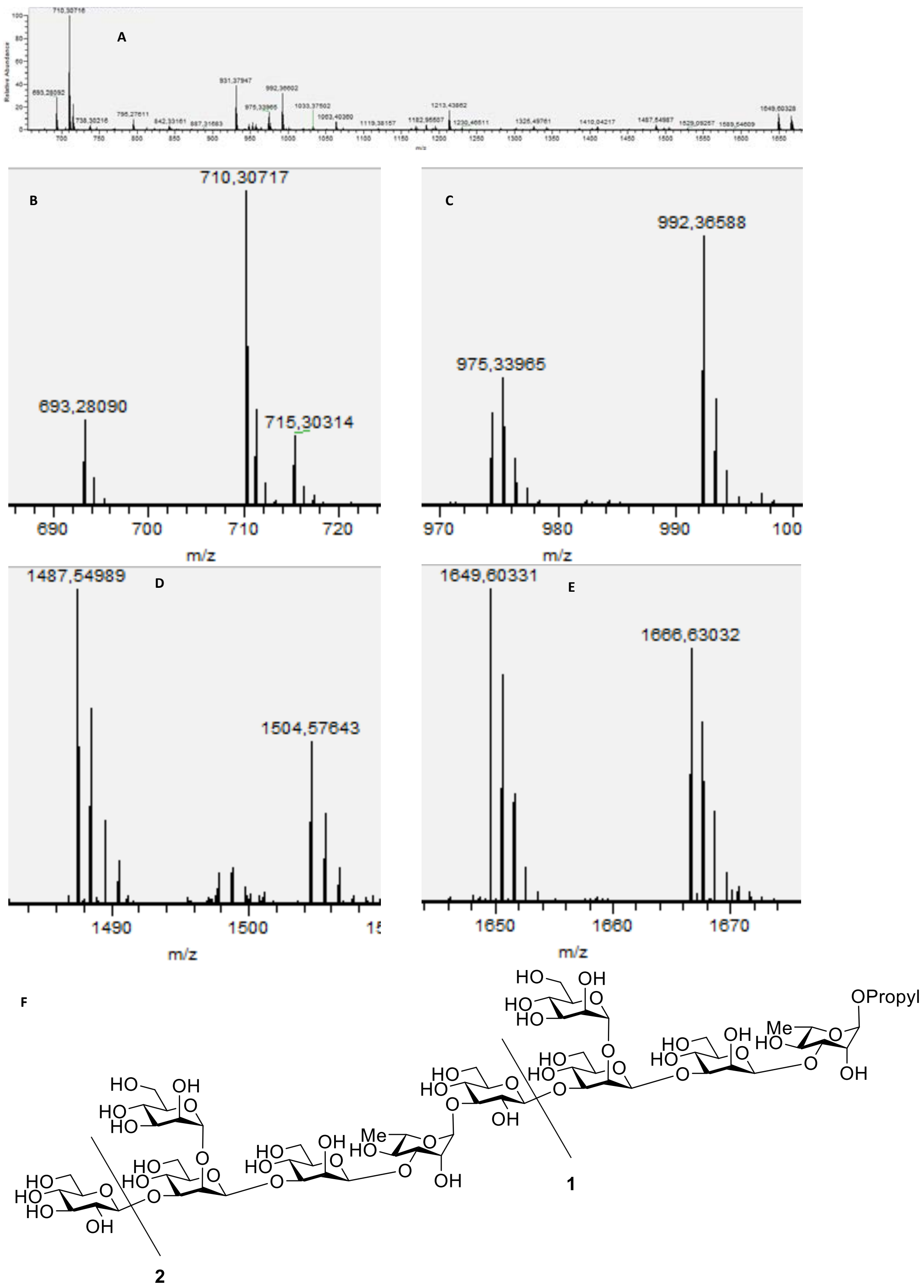

Figure S8. MS analysis of decasaccharide **10** digestion by PslG. **A**) Results of a digestion experiment with PslG using a decasaccharide fragment containing two Psl repeating units. **B**) H^+ ion and NH_4^+ ion of a tetrasaccharide fragment; **C**) H^+ ion and NH_4^+ ion of a hexasaccharide fragment; **D**) H^+ ion and NH_4^+ ion of a nonasaccharide fragment; **E**) H^+ ion and NH_4^+ ion of the decasaccharide; **F**) Determined cleavage sites 1 and 2 observed during this experiment.

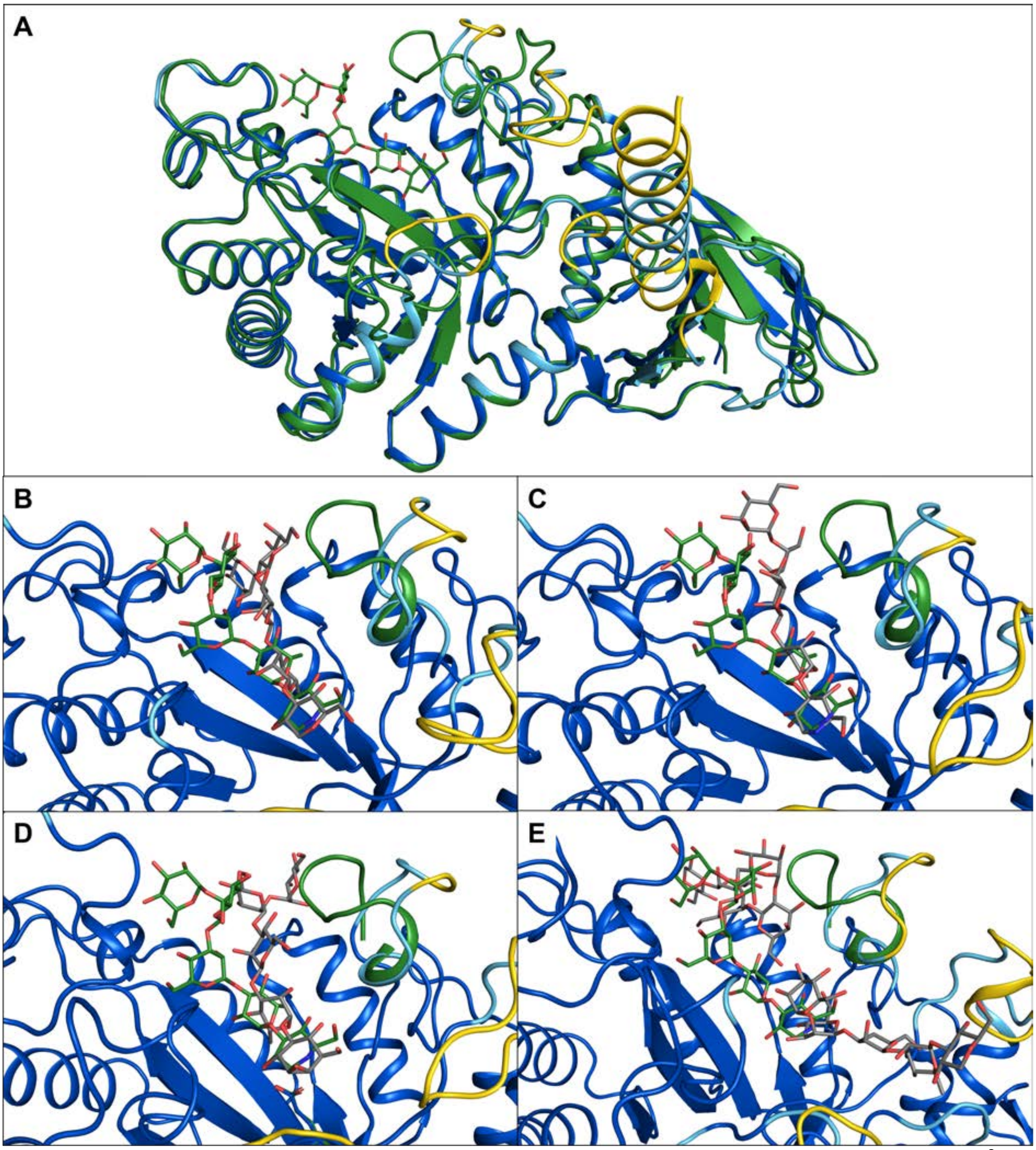

Figure S9. Chai-1-generated structures of PslG colored by model confidence (akin to AlphaFold2).[2] (**A**) Overlay of the nojirimycin X-ray structure (green) and a representative Chai-1 generated structure, highlighting the overall similarity and local differences between the structures, (**B**) overlay with 1-deoxyglucose repeating pentasaccharide (grey), (**C**) overlay with the deoxynojirimycin pentasaccharide (grey), (**D**) overlay with the cyclophellitol pentasaccharide (grey) also showing the nucleophilic E276 and (**E**) overlay with a structure containing the 1-deoxyglucose repeating pentasaccharide twice (grey), in an attempt to capture the +1 and beyond sites of the pocket. Figure generated using PyMOL 3.0 Open Source.

Biochemistry Methods

Recombinant protein production and purification

The coding sequence for PslG residues 31 – 442 was cloned into the pET28a vector (Novagen), behind an N-terminal hexahistidine tag and thrombin cleavage site. The construct was transformed into *E. coli* BL21(DE3) cells, and an overnight culture was grown up in 5 mL Luria Broth (LB) with 50 µg/mL kanamycin whilst shaking at 180 rpm at 37 °C. This was used to inoculate 0.5 L Terrific Broth with 50 µg/ml kanamycin, which was grown shaking at 200 rpm at 37 °C to an optical density at 600 nm of 0.9. Expression was induced with 0.1 mM IPTG followed by overnight growth at 200 rpm and 16 °C. The cells were harvested by centrifugation at 5,000 rpm for 20 min at 4 °C. The pellet was resuspended in approximately 40 mL HisTrap buffer A (20 mM Tris pH 8.0, 500 mM NaCl, 20 mM imidazole), and bovine pancreatic DNase I (Sigma) and cOmplete^{TM} EDTA-free protease inhibitors (Roche) were added, and the sample was then passed through a cell disrupter (Constant systems) at 40 kPSI operating pressure. The lysate was loaded onto a 1 mL HisTrap FF crude column (Cytiva) pre-equilibrated with HisTrap buffer A, and the column was washed with 10 column volumes (CV) of buffer A, before eluting the protein with HisTrap buffer B (20 mM Tris pH 8.0, 500 mM NaCl, 1 M imidazole) over a 20 CV gradient. Fractions containing PslG were pooled and diluted to ~ 50 mL in 20 mM HEPES pH 7.4. Thrombin (Sigma; bovine plasma) was added in a 1:100 mass ratio of thrombin:PslG and the digest reaction was left overnight at room temperature and then loaded onto a 1 ml HiTrap Q HP ion exchange column (GE Healthcare) pre-equilibrated with 20 mM HEPES pH 7.4. The unbound fractions were collected, concentrated to 2 mL and loaded onto a Superdex S75 HR 16/600 size exclusion chromatography column (Cytiva) equilibrated with 20 mM HEPES pH 7.4, 200 mM NaCl. The main peak fractions were pooled, concentrated and buffer exchanged into 20 mM HEPES pH 7.4, 50 mM NaCl at a final concentration of 12.9 mg/mL.

ABPP assays on recombinant PslG

1 µL 10 µM enzyme (1 µM final concentration) was added to 9 µL 150 mM McIlvaine buffer at pH 5.5 (or pH 5, 6, 6.5 or 7 or 100 mM HEPES pH 7 for pH profile) before addition of 1 µL 10 µM probe (1 µM final concentration) and incubation for 30 minutes at 37 °C, with shaking at 400 rpm. The reaction was quenched with 4x Laemmli buffer (3 µL) and boiled for 5 minutes at 95 °C. For the control experiments the enzyme was heated for 5 minutes at 95 °C and allowed to cool before addition of the probe. 10 µL of the denatured enzyme was then loaded onto a 12 well 4-20% pre-cast Mini-PROTEAN TGX gel (BioRad) and run at 200 V for 40 minutes. The gel was then extracted into de-ionised water and then transferred onto a Typhoon 5 laser-scanner (Cytiva) and the Cy5 fluorescence imaged (λ_{EX} 635 nm; λ_{EM} > 665 nm). The gel was placed in Coomassie Brilliant Blue solution and microwaved for 1 min to fix the proteins. After staining, the gel was de-stained in de-ionized water and the Coomassie stained proteins were imaged. For the competition gels, 1 µL 10 µM enzyme (1 µM final concentration) was added to 8 µL 150 mM McIlvaine buffer at pH 5.5 before addition of 1 µL 10x inhibitor or water and incubation for 1 hour or overnight for 18 hours at 37 °C, with shaking at 400 rpm. 1 µL 10 µM probe (1 µM final concentration) was then added and the reaction incubated for a further 30 minutes at 37 °C. 3 µL 4x Laemmli buffer was then added, and the samples boiled for 5 minutes at 95 °C. The samples were then loaded onto an SDS-PAGE and imaged as above.

Decasaccharide digestion

To decasaccharide **15** (20 nmol) was added NH_4OAc (final concentration 100 mM) and PslG (final concentration 0.75 µM). This solution (30 µL final volume) was allowed to react for 24 hours at 37 °C at 400 rpm shaking. The solution was then diluted to 1 mL with MilliQ, after an additional 24 hours at room temperature, PslG was removed using a C18 stage tip, which was washed with H_2O. The solution was then lyophilized to remove volatiles. The sample was then redissolved and analyzed using HRMS. The HRMS analysis was performed on a Q-Exactive HF Orbitrap (Thermo Scientific) equipped with an electrospray ion source (ESI), injection of 2 µl of a 1 µM solution via Ultimate 3000 nano UPLC (Dionex) system, with an external calibration (Thermo Scientific). Source voltage of 3,5 kV, capillary temperature 275 ºC, no sheath gas, resolution = 240.000 at m/z=400. Mass range was set at m/z 160-2000 or until a maximum of 6000. Eluents used: MeCN:H_2O (1:1 v/v) supplemented with 0,1% formic acid.

Crystallization, data collection and data refinement

PslG was buffer-exchanged into 20 mM MES pH 6, 50 mM NaCl at 10.7 mg/mL. 10 µL 20 mM ligand **17** dissolved in water was added to 100 µL PslG and the sample was incubated in an Eppendorf tube at room temperature for 24 hours, after which the samples were put at 4 °C. The PslG-ligand mixture looked hazy after a day at 4 °C. When the sample was centrifuged for 5 min at 13,000 rpm a pellet

emerged which consisted of crystals which had grown in the tube. The crystals were pipetted onto an MRC Maxi 48 well crystallization plate (Swissci) and fished into liquid nitrogen via a cryoprotectant comprised of 20 mM MES pH 6, 50 mM NaCl, 20% glycerol. Crystals with **23** were grown using a similar method and fished after 6 days.

X-ray data were collected at Diamond Light Source (UK) beamline io3, and processed using *DIALS*[3] as part of the *xia*2 pipeline[4], and scaled with AIMLESS[5] to 1.55 Å and 1.45 Å for **20** and **25** respectively. The structures were solved by molecular replacement using *Phaser*[6] with PDB entry 4ZN2.pdb as the model. The space group of the solutions was P65, with unit cell dimensions 97.7, 97.7 and 119.8 Å for **20** and 97.6, 97.6 and 119.8 Å for **25**, both with angles of 90, 90 and 120 degrees. The protein chains were built, and water molecules added using *Coot*[7], and cycles of *REFMAC*[8] were run to refine the structure between model building sessions. The ligands were built using sugars in the *Coot* monomer library. Programs were run in the *CCP4* cloud interface.[9]

Chai-1 structure prediction

For Chai-1 structure predictions the beta environment provided by Chai Discovery was used (https://lab.chaidiscovery.com/ accessed: October-November 2024). The full sequence of PslG was provided as protein modeling target, and the ligand was provided as SMILES (Chemdraw 22.0). For Chai-1, the language model was used to capture evolutionary statistics (no MSAs). No restraints were provided.

ABPP on bacterial cells

To test binding of the probe to native PslG in *P. aeruginosa*, 0, 1 and 10 μM of probe were incubated with wild type Psl producing PAO1 strains and strains lacking or overexpressing PslG. Overnight cultures of the bacterial strains were normalized to OD 1 and incubated with the probe for 45 min at 37 °C. Post incubation, bacteria were centrifuged at 5000xg for 5 min and the bacterial pellet washed twice with phosphate buffered saline (PBS). The bacterial pellet was then lysed by adding 100 μL of SDS loading dye (50 mM Tris pH8, 1% SDS, 5% glycerol, bromophenol blue) and boiling at 100 °C for 5-10 min. Samples were then processed using SDS-PAGE and assessed for binding of probe by fluorescence. The same gel was then stained with Coomassie blue to evaluate equal loading of proteins across the samples. For Western blot analysis, proteins form SDS PAGE were transferred onto a PVDF membrane. The blots were probed for PslG using a rabbit anti-PslG (Cedarlane) primary antibody followed by incubation with an HRP-tagged anti-rabbit secondary antibody (Biorad). Bands were detected using a chemiluminescene substrate (Supersignal™ West PICO PLUS, Thermo Fisher Scientific). SDS PAGE gels and western blots were imaged using the Chemidoc™ MP imaging system from BioRad.

Chai-1 structure prediction

For Chai-1 structure predictions the beta environment provided by Chai Discovery was used (https://lab.chaidiscovery.com/ accessed: October-November 2024). The full sequence of PslG was provided as protein modeling target, and the ligand was provided as SMILES (Chemdraw 22.0). For Chai-1, the language model was used to capture evolutionary statistics (no MSAs). No restraints were provided.

Table S1. Crystallographic data.

Data collection	PslG-**20**	PslG-**25**
Space group	$P6_5$	$P6_5$
Cell dimensions		
a, *b*, *c* (Å)	97.7, 97.7, 119.8	97.6, 97.6, 119.8
α, β, γ (°)	90.0, 90.0, 120.0	90.0, 90.0, 120.0
Resolution (Å)	84.60 – 1.55 (1.58 – 1.55)	69.17 - 1.50 (1.53 - 1.50)
R_{merge}	0.155 (2.495)[1]	0.127(2.584)
R_{pim}	0.057 (0.926)	0.041 (0.843)
$CC_{1/2}$	0.999 (0.663)	0.999 (0.669)
I / σ	11.0 (1.2)	14.1 (1.3)
Completeness (%)	100.0 (99.9)	100.0 (100.0)
Redundancy	16.2 (16.2)	20.4 (20.7)
Refinement		
No. reflections	89016	98087
R_{work} / R_{free}	0.14/0.17	0.13/0.16
No. atoms		
Protein	3528	3408
Ligand/ion	113	93
Water	638	636
B-factors		
Protein	19.8	21.1
Ligand/ion	26.9	31.0
Water	38.7	38.5
R.m.s deviations		
Bond lengths (Å)	0.013	0.014
Bond angles (°)	1.716	1.827
Ramachandran plot residues		
In most favorable regions (%)	97.1	97.3
In allowed regions (%)	2.9	2.7
PDB code	9G17	9G18

Compound Synthesis

General

Chemicals were purchased from Sigma Aldrich, Acros, Fluorochem, VWR, Carbosynth, Fischer Scientific and Merck. Chemicals were used as received unless stated otherwise. Toluene, DCM, THF, DMF and ACN were stored over flame-dried molecular sieves (either 3 or 4 Å) before use. All reactions were performed under a nitrogen atmosphere unless stated otherwise. Thin layer chromatography was performed on Merck aluminum sheets (Silica gel 60 F254). For initial UV detection a lamp set to 254 nm was used after which spots were further visualized by spraying with a solution of $(NH_4)_6Mo_7O_{24}\cdot 4H_2O$ (25 g/L) and $(NH_4)_4Ce(SO_4)_4\cdot 2H_2O$ (10 g/L) in 10% sulfuric acid or a solution of $KMnO_4$ (20 g/L) and K_2CO_3 (10 g/L) in water, followed by charring at 150 °C. Flash column chromatography was performed using Screening Device b.v. silica gel (particle size of 40 – 63 μm, pore diameter of 60 Å) with the indicated eluents. ^1H NMR and ^{13}C NMR spectra were recorded on a Bruker DPX-300 (300 and 75 MHz respectively), a Bruker AV-400 (400 and 101 MHz respectively), a Bruker AV-400WB (400 and 101 MHz respectively), a Bruker AV-500 (500 and 126MHz respectively), a Bruker AV-600 (600 and 151 MHz respectively) or a Bruker-850 (800 and 200 MHz respectively) spectrometer in the given solvent. Chemical shifts are reported in ppm (δ) relative to the residual solvent peak or tetramethyl silane (0 ppm) as internal standard and coupling constants are given in Hz. An Äkta Explorer (GE Healthcare) using 1.6x60 cm Toyopearl HW-40S resin was used for gel filtration. Elution of the compounds was done with a solution of 1% AcOH in ACN/H_2O, 1/9, v/v for acid labile compounds or 150 mM solution of NH_4HCO_3 for base labile compounds. Refractive index was used to analyze fractions. For reversed phase HPLC purifications an Agilent Technologies 1200 series instrument equipped with a semi-preparative column (Gemini C18, 250 x 10 mm, 5 μm particle size, Phenomenex) was used. LC/MS analysis was performed on a Surveyor HPLC system (Thermo Finnigan) equipped with a C18 column (Gemini, 4.6 mm x 50 mm, 5 μm particle size, Phenomenex), coupled to a LCQ Advantage Max (Thermo Finnigan) ion-trap spectrometer (ESI+). The applied buffers were H_2O, MeCN and 1% aqueous TFA. High resolution mass spectrometry (HRMS) analysis was performed with a Synapt G2-Si (waters) equipped with an electrospray ion source in positive mode (ESI-TOF), injection of 2 μl of a 2 μM solution via NanoEquity system (Waters), with LeuEnk (m/z 556.2771) as "lock mass". Source voltage of 3,5 kV, 275 °C as temperature. Mass range m/z = 160-2000 or until a maximum of 6000. Eluens used: MeCN:H_2O(1:1 v/v) supplemented with 0.1% formic acid or with a Q-Exactive HF Orbitrap (Thermo Scientific) equipped with an electronspray ion source(ESI), injection of 2 μl of a 1 μM solution via Ultimate 3000 nano UPLC (Dionex) system , with an external calibration (Thermo Scientific). Source voltage of 3,5 kV, capillary temperature 275 °C, no sheath gas, Resolution = 240.000 at m/z=400. Mass range m/z=160-2000 or until a maximum of 6000. Eluens used: MeCN:H_2O (1:1 v/v) supplemented with 0,1% formic acid. The high-resolution mass spectrometer was calibrated prior to measurements with a calibration mixture (Thermo Finnigan). NMR assignments are done as follows: the anomeric C/H is denoted as 1 and numbered through the ring, ending with C-6/H-6 at the primary alcohol. Cyclophellitol compounds are numbered as their parent carbohydrates, with the additional ring C/H denoted as 7. Each consecutive carbohydrate is assigned an additional '(so the 2nd carbohydrate residue has one 'and the 3rd carbohydrate has two " etc.).

Synthesis of probe 1

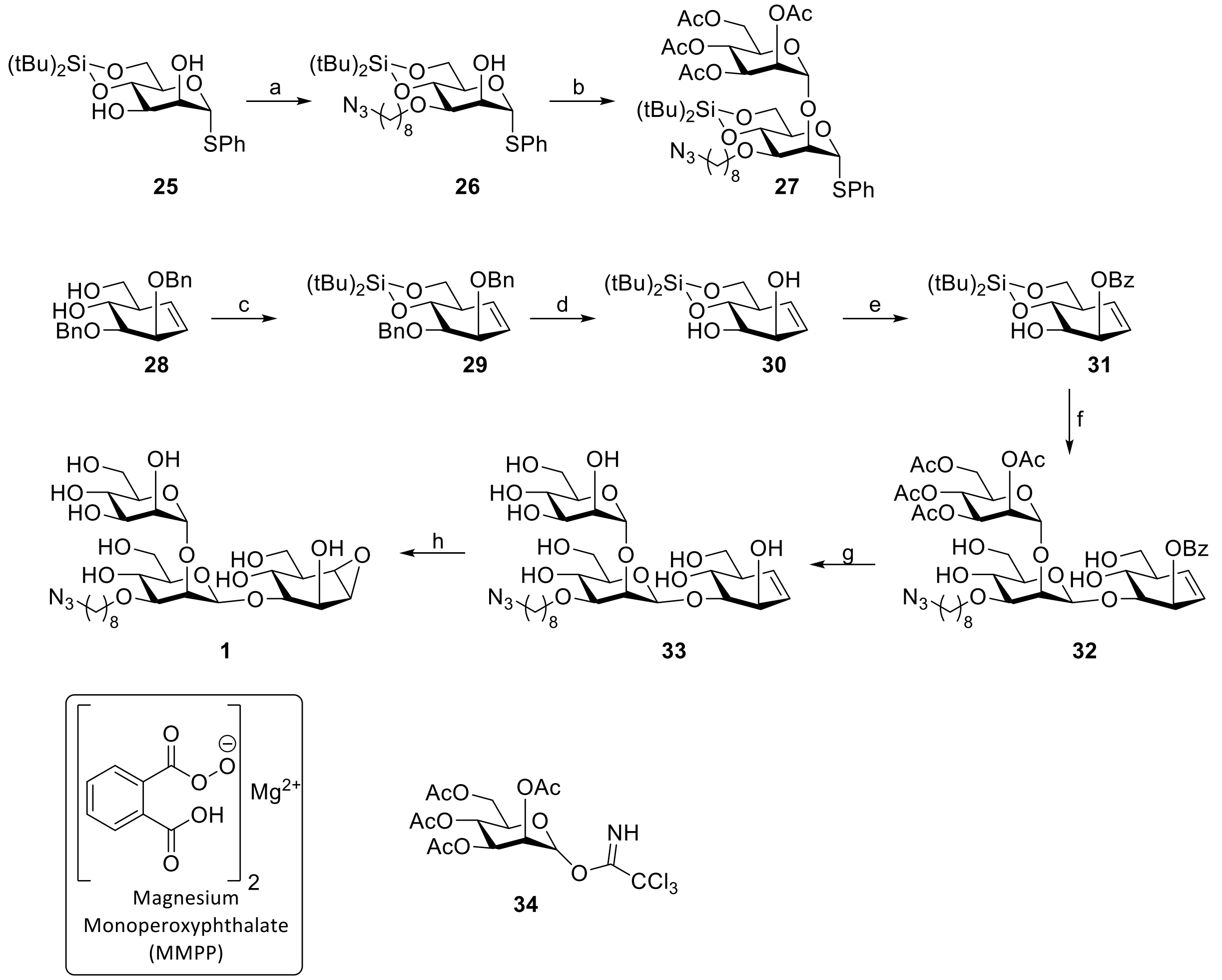

Scheme 1. a) 8-Azidooctyl trifluoromethanesulfonate, 2-aminoethyl diphenylborinate, K_2CO_3, MeCN, 0 °C, 80%; b) **34**[10], TMSOTf, DCM, -20 °C to 5 °C, 78%; c) Di-tert-butylsilyl ditriflate, imidazole, DMF, 73%; d) $TiCl_4$, DCM, toluene, 0 °C, 82%; e) BzCl, 2-aminoethyl diphenylborinate, DIPEA, MeCN, rt, 65%; f) i. Ph_2SO, Tf_2O, TTBP, DCM, cyclohexene, -80 °C -> -40°C; ii. 3HF·Et_3N, THF, 35% over 2 steps, 65% based on recovered **10**; g) NaOMe, MeOH; h) MMPP, NaOH, H_2O, 15% over 2 steps.

8-azidooctyl trifluoromethanesulfonate

Tf_2O (0.54 mL, 3.22 mmol) was dissolved in DCM (7 mL) and cooled to -20 °C. A solution of 8-azido-1-octanol (0.46 g, 2.68 mmol) and pyridine (0.25 mL, 3.22 mmol) in DCM (7 mL) was added and the reaction was stirred for 1 hour at the same temperature. The reaction mixture was diluted with DCM and subsequently washed with cold water and cold brine, dried with $MgSO_4$ and concentrated *in vacuo*. The reagent was used immediately without further purification.

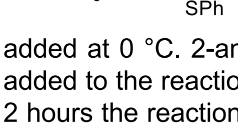

Phenyl 3-(8-azidooctyl)-4,6-*O*-ditertbutylsilyl-1-thio-α-D-mannopyranose (**26**). Diol **25**[11] (1.12 g, 2.69 mmol) and K_2CO_3 (0.42 g, 3.0 mmol) were coevaporated with toluene. Freshly prepared 8-azidooctyl trifluoromethanesulfonate (1.22 g, 4.03 mmol) was dissolved in ACN (7 mL) and added at 0 °C. 2-aminoethyl diphenylborinate (0.06 g, 0.27 mmol) was dissolved in ACN (7 mL) and added to the reaction mixture. DCM (1 ml) was added, and the reaction was slowly warmed to rt. After 2 hours the reaction was quenched with $NaHCO_3$ (aq. sat.) and diluted with H_2O. The water layer was extracted with EtOAc (2x) and the combined organic phase was washed with brine, dried over $MgSO_4$, filtered and concentrated *in vacuo*. The product was purified by column chromatography (pentane/EtOAc, 10/1, v/v) (1.21 g, 80%). ^{1}H NMR (400 MHz, $CDCl_3$) δ 7.47 – 7.42 (m, 2H), 7.34 – 7.23 (m, 3H), 5.55 (d, *J* = 1.3 Hz, 1H, H1), 4.28 – 4.13 (m, 3H, H4/H2/H5), 4.05 – 3.92 (m, 2H, H6ab), 3.89 – 3.81 (m, 1H, CH_2O), 3.76 – 3.69 (m, 1H, CH_2O), 3.50 (dd, *J* = 8.4, 3.4 Hz, 1H, H3), 3.26 (t, *J* = 6.9 Hz, 2H, CH_2N_3), 2.83 (s, 1H, OH), 1.65 – 1.55 (m, 4H, CH_2 (2x)), 1.44 – 1.29 (m, 8H, CH_2 (4x)), 1.06 (s, 9H, (*t*-Bu), 1.04 (s, 9H, *t*-Bu). ^{13}C NMR (101 MHz, $CDCl_3$) δ = 133.9, 131.5, 129.3, 127.6, 87.6 (C1), 79.0

(C3), 75.1, 72.0 (CH_2O), 71.7 , 68.3, 66.5 (C6), 51.6 (CH_2N_3), 30.2 (CH_2), 29.4 (CH_2), 29.3 (CH_2), 29.0 (CH_2), 27.5 ($SiC(CH_3)_3$), 27.2 ($SiC(CH_3)_3$), 26.8 (CH_2), 26.1 (CH_2), 22.7 ($SiC(CH_3)_3$), 20.1 ($SiC(CH_3)_3$). HRMS (ESI) m/z: $[M+Na]^+$ calculated for $C_{28}H_{47}N_3O_5SSiNa$ 588.2903, found 588.2902.

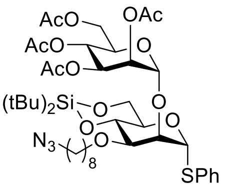

Phenyl 2-*O*-(2,3,4,6-tetra-*O*-acetyl-β-D-mannopyranosyl)-3-*O*-(8-azidooctyl)-4,6-*O*-ditertbutylsilyl-1-thio-α-D-mannopyranose (**27**). Acceptor **26** (1.21 gram, 2.14 mmol) and donor **34**[10] (1.22 gram, 2.57 mmol) were coevaporated with toluene (3x) and dissolved in DCM (11 mL, 0.2M). 4Å molecular sieves were added and the mixture was stirred for 30 minutes. The reaction was cooled to -20 °C and TMSOTf (0.08 ml, 0.43 mmol) was added. The reaction was allowed to warm to 5 °C in 3 hours. The reaction was quenched with Et_3N and diluted with DCM. The organic layer was washed with $NaHCO_3$ (sat. aq.). The water layer was extracted with DCM and the combined organic layers were washed with brine and dried over $MgSO_4$. The solvent was removed *in vacuo* and the product was isolated by column chromatography (pentane/EtOAc, 4/1, v/v) to afford a white sticky solid (1.38 g, 78%). Rf: 0.56 (8/2 pentane/EtOAc).^{1}H NMR (400 MHz, $CDCl_3$) δ 7.47 – 7.42 (m, 2H), 7.37 – 7.25 (m, 3H), 5.46 (d, *J* = 1.4 Hz, 1H, H1), 5.38 (dd, *J* = 3.4, 1.8 Hz, 1H, H2’), 5.33 (dd, *J* = 9.9, 3.4 Hz, 1H, H3’), 5.21 (t, *J* = 9.8 Hz, 1H, H4’), 5.16 (d, *J* = 1.8 Hz, 1H, H1), 4.26 – 4.15 (m, 4H, H5/H4/H2/H6a), 4.07 – 3.97 (m, 4H, H6b/H6ab’/H5’), 3.85 (dt, *J* = 9.3, 6.4 Hz, 1H, CH_2O), 3.62 (dt, *J* = 9.3, 6.3 Hz, 1H, CH_2O), 3.53 (dd, *J* = 8.8, 3.1 Hz, 1H, H3), 3.26 (t, *J* = 7.0 Hz, 2H, CH_2N_3), 2.15 (s, 3H, OAc), 2.03 (s, 3H, OAc), 1.98 (s, 3H, OAc), 1.90 (s, 3H, OAc), 1.64 – 1.49 (m, 4H, spacer CH_2 (2x)), 1.40 – 1.28 (m, 8H, spacer CH_2 (4x)), 1.09 (s, 9H, *t*-Bu), 1.03 (s, 9H, *t*-Bu). ^{13}C NMR (101 MHz, $CDCl_3$) δ = 170.8 (OAc), 170.0 (OAc), 169.8 (OAc), 169.7 (OAc), 133.9 (SPh), 131.4 (SPh), 129.4 (SPh), 127.8 (SPh), 99.6 (C1’), 87.8 (C1), 79.2 (C3), 78.4, 75.4, 72.3 (CH_2O), 69.4 (C2’), 69.2, 69.0, 68.9 (C3’), 66.5 (C4’), 66.4 (C6), 62.7 (C6), 51.6 (CH_2N_3), 30.2 (spacer), 29.4 (spacer), 29.2 (spacer), 28.9 (spacer), 27.6 (*t*-Bu), 27.2 (*t*-Bu), 26.8 (spacer), 26.1 (spacer), 22.8 (*t*-Bu), 21.0 (OAc), 20.8 (OAc), 20.8 (OAc), 20.6 (OAc), 20.1 (*t*-Bu). HRMS (ESI) m/z: $[M+Na]^+$ calculated for $C_{42}H_{65}N_3O_{14}SSiNa$ 918.3862, found 918.3854.

2,3-*O*-benzyl-4,6-*O*-ditertbutylsilyl-mannosecyclophellitolalkene (**29**). Alkene **28**[12] (1.1 g, 3.23 mmol) was coevaporated with toluene (2x), imidazole (0.9 g, 13.2 mmol) was added, and the mixture was dissolved in DMF (32 mL, 0.1M). The solution was cooled to 0 °C, di-tert-butyl-silyltriflate (2.8 mL, 8.7 mmol) was added dropwise and the reaction mixture was allowed to warm to room temperature and stirred overnight. The reaction was quenched with MeOH and the product was extracted with Et_2O (2x), the organic phase was washed with HCl (1 M), $NaHCO_3$ (aq. sat.) and brine, dried over $MgSO_4$ and

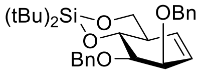

volatiles were removed under reduced pressure. The product was obtained after column chromatography (pentane/EtOAc, 20/1, v/v) as a colorless oil. (1.14 g, 73%).^{1}H NMR (400 MHz, $CDCl_3$) δ 7.44 – 7.26 (m, 10H), 5.69 (ddd, *J* = 9.8, 5.0, 3.0 Hz, 1H, alkene), 5.30 (dd, *J* = 9.8, 1.8 Hz, 1H, alkene), 5.06 (d, *J* = 12.4 Hz, 1H, CH_2Bn), 4.92 (d, *J* = 12.4 Hz, 1H, CH_2Bn), 4.82 (d, *J* = 12.4 Hz, 1H, CH_2Bn), 4.74 (d, *J* = 12.4 Hz, 1H, CH_2Bn), 4.43 (dd, *J* = 10.3, 9.1 Hz, 1H, H4), 4.08 (dd, *J* = 10.4, 4.6 Hz, 1H, H6a), 4.03 (t, *J* = 4.4 Hz, 1H, H2), 3.87 (dd, *J* = 12.0, 10.3 Hz, 1H, H6b), 3.52 (dd, *J* = 10.2, 4.3 Hz, 1H, H3), 2.53 – 2.44 (m, 1H, H5), 1.09 (s, 9H, *t*-Bu), 1.05 (s, 9H, *t*-Bu). ^{13}C NMR (101 MHz, $CDCl_3$) δ = 139.7, 139.2, 128.4, 128.4, 128.2, 127.7, 127.7, 127.6, 127.5, 127.3, 81.3 (C3), 75.6 (C4), 74.0 (CH_2Bn), 73.4 (CH_2Bn), 73.2 (C2), 68.5 (C6), 45.7 (C5), 27.6 (*t*-Bu), 27.4 (*t*-Bu), 22.9 (*t*-Bu), 20.0 (*t*-Bu). HRMS (ESI) m/z: $[M+NH_4]^+$ calculated for $C_{29}H_{44}O_4SiN$ 498.3034, found 498.3033.

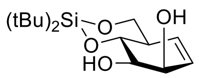

4,6-*O*-ditertbutylsilyl-mannocyclophellitolalkene (**30**). Alkene **29** (0.99 g, 2.06 mmol) was dissolved in DCM (20 ml, 0.1 M) and cooled to 0 °C. A solution of $TiCl_4$ (1M in toluene, 8.24 ml, 8.24 mmol) was added slowly. After 20 minutes, the reaction was quenched by careful addition of $NaHCO_3$ (sat. aq.). The obtained suspension was filtered over celite. The layers were separated, and the aqueous layer was extracted with EtOAc. The combined organic layers were washed with brine and dried over $MgSO_4$. The solvent was evaporated *in vacuo* and the product was isolated by column chromatography (pentane/EtOAc, 9/1 to 7.5/2, v/v) as a colorless oil (0.51 g, 82%).^{1}H NMR (400 MHz, $CDCl_3$) δ 5.89 (ddd, *J* = 9.8, 4.8, 2.9 Hz, 1H, alkene), 5.42 (dd, *J* = 9.9, 1.8 Hz, 1H, alkene), 4.39 (t, *J* = 4.5 Hz, 1H, H2), 4.13 (dd, *J* = 10.4, 4.8 Hz, 1H, H6a), 4.02 (t, *J* = 9.7 Hz, 1H, H4), 3.83 (dd, *J* = 12.0, 10.4 Hz, 1H, H6b), 3.65 (dd, *J* = 10.1, 4.5 Hz, 1H, H3), 3.11 (s, 1H, OH), 2.87 (s, 1H, OH), 2.56 – 2.45 (m, 1H, H5), 1.05 (s, 9H, *t*-Bu), 1.01 (s, 9H, *t*-Bu). ^{13}C NMR (101 MHz, $CDCl_3$) δ = 128.0 (alkene), 127.7 (alkene), 74.1 (C4), 73.7 (C3), 68.1 (C6), 66.1 (C2), 43.8 (C5), 27.6 (*t*-Bu), 27.2 (*t*-Bu), 22.8 (*t*-Bu), 20.0 (*t*-Bu). HRMS (ESI) m/z: $[M+Na]^+$ calculated for $C_{15}H_{28}O_4SiNa$ 323.1649, found 323.1647.

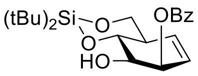

2-*O*-benzoyl-4,6-*O*-ditertbutylsilyl-mannocyclophellitolalkene (**31**). Diol **30** (0.28 g, 0.94 mmol) was dissolved in MeCN (4.8 ml, 0.2 M). DIPEA (0.82 ml, 4.72 mmol), BzCl (0.33 ml, 2.83 mmol) and 2-aminoethyl diphenylborinate (21 mg, 0.094 mmol) were added and the mixture was stirred for 17 hours at rt. The reaction was diluted with Et_2O and washed with HCl (1 M), $NaHCO_3$ (sat. aq.) and brine. The organic layer was dried over $MgSO_4$ and the solvent was removed under reduced pressure. The product was obtained by column chromatography (pentane/Et_2O, 95/5 to 85/15, v/v) as an orange oil. (250 mg, 65%) ^1H NMR (400 MHz, $CDCl_3$) δ 8.07 – 8.02 (m, 2H), 7.60 – 7.52 (m, 1H), 7.44 (m, 2H), 5.93 (ddd, *J* = 9.7, 5.0, 2.9 Hz, 1H, alkene), 5.83 (td, *J* = 4.8, 1.1 Hz, 1H, H2), 5.55 (dd, *J* = 9.7, 1.9 Hz, 1H, alkene), 4.24 – 4.15 (m, 2H, H6a/H4), 3.94 – 3.84 (m, 2H, H6b/H3), 2.63 – 2.53 (m, 1H, H5), 1.09 (s, 9H, *t*-Bu), 1.03 (s, 9H, *t*-Bu). ^{13}C NMR (101 MHz, $CDCl_3$) δ = 166.2 (PhCOO), 133.1 (Bz), 130.4 (Bz), 129.9 (Bz), 129.7 (alkene), 128.5 (Bz), 125.5 (alkene), 74.7 (C4), 72.5 (C3), 68.4 (C2), 68.1 (C6), 44.2 (C5), 27.6 (*t*-Bu), 27.2 (*t*-Bu), 23.0 (*t*-Bu), 20.0 (*t*-Bu). HRMS (ESI) m/z: $[M+H]^+$ calculated for $C_{22}H_{33}O_5Si$ 405.2092, found 405.2089.

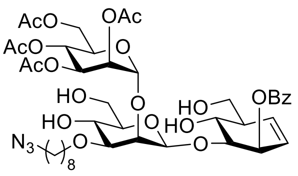

2-*O*-benzoyl-3-*O*-(2-*O*-(2,3,4,6-tetra-*O*-acetyl-α-D-mannopyranosyl)-3-*O*-(8-azidooctyl)-β-D-mannopyranosyl)-4,6-*O*-ditertbutylsilyl-mannocyclophellitolalkene (**32**). Disaccharide donor **27** (0.20 g, 0.22 mmol), diphenyl sulfoxide (0.045 g, 0.22 mmol) and TTBP (0.15 g, 0.59 mmol) were co-evaporated with toluene (2x). The dry starting materials were dissolved in DCM (2 ml), 4Å molecular sieves were added and the mixture was stirred at rt for 30 minutes. The reaction was cooled to -72 °C and Tf_2O (0.3 M in DCM, 0.7 ml, 0.21 mmol) was added. The reaction was warmed to -60 °C over 30 minutes and was subsequently cooled to -80 °C. Acceptor **31** (0.060 g, 0.15 mmol) and cyclohexene (0.08 ml, 0.74 mmol) were dissolved in DCM (1 ml) and added slowly to the reaction mixture. The reaction was allowed to warm to -40 °C and was quenched with Et_3N at that temperature. The mixture was diluted with EtOAc. The molecular sieves were removed, and the solution was washed with $NaHCO_3$ (sat. aq.) and brine. The organic layer was dried over $MgSO_4$, and the solvent was removed under reduced pressure. Column chromatography (pentane/EtOAc, 90/10 to 84/16, v/v) yielded a mixture of product and donor (85 mg) and unreacted acceptor (26 mg, 43%). The product mixture was taken up in THF (5 mL) and 3HF·Et_3N (0.15 mL, 6,59 mmol) was added. The reaction was stirred overnight. More 3HF·Et_3N (0.15 mL, 6.59 mmol) was added, and the reaction was stirred for 5 hours. The mixture was diluted with THF and $CaCO_3$ (1.0 g, 10 mmol) was added. The suspension was stirred for 30 minutes before it was filtered over celite. The solvent was removed *in vacuo* and the pure product was obtained by column chromatography (DCM/acetone, 1/0 to 2/8, v/v) as a colorless oil (47 mg, 35%, 65% based on recovered acceptor) ^1H NMR (400 MHz, $CDCl_3$) δ 8.04 – 7.98 (m, 2H, Bz), 7.60 – 7.54 (m, 1H, Bz), 7.45 (m, 2H, Bz), 5.92 – 5.82 (m, 3H, alkene (2x)/H2), 5.34 (dd, *J* = 10.1, 3.4 Hz, 1H, H3"), 5.22 – 5.14 (m, 2H, H4"/H2"), 4.90 (d, *J* = 2.0 Hz, 1H, H1"), 4.80 (s, 1H, H1'), 4.25 (dt, *J* = 10.0, 3.3 Hz, 1H, H5"), 4.12 (dd, *J* = 10.0, 3.5 Hz, 1H, H3), 4.09 – 3.99 (m, 2H, H6a"/H4), 3.99 – 3.79 (m, 6H, H2'/H6ab/H6ab'/H4'), 3.60 (dt, *J* = 9.1, 6.5 Hz, 1H, spacer), 3.55 – 3.41 (m, 2H, H6b"/spacer), 3.38 (ddd, *J* = 9.7, 5.0, 3.0 Hz, 1H, H5'), 3.30 (dd, *J* = 9.4, 2.5 Hz, 1H, H3'), 3.26 (t, *J* = 7.0 Hz, 2H, CH_2N3), 2.53 (m, 1H, H5), 2.08 (s, 3H, OAc), 2.04 (s, 3H, OAc), 2.02 (s, 3H, OAc), 1.96 (s, 3H, OAc), 1.59 (m, 4H, spacer), 1.39 – 1.27 (m, 8H, spacer). ^{13}C NMR (101 MHz, $CDCl_3$) δ = 170.7, 170.2, 169.8, 166.5, 133.6, 133.4, 129.8, 129.7, 128.8, 123.6 (alkene), 97.7 (C1"), 97.3 (C1'), 82.2 (C3'), 77.3 (C3), 76.6 (H5'), 72.8 (C2'), 70.2 (spacer), 69.5 (C2"), 69.2 (3"), 68.6 (C5"/C4), 66.7 (C4'), 66.1 (C4"), 65.7 (C2), 64.9 (C6'), 62.3 (C6"), 62.1 (C6), 51.6 (CH_2N_3), 46.7 (C5), 29.8, 29.4, 29.1, 28.9, 26.8, 26.0 (spacer 6x), 21.0, 20.9, 20.8 (OAc 4x). $^1J_{H,C}$ (H1") 4.90 ppm, 97.7 ppm = 171 Hz, $^1J_{H,C}$ (H1') 4.80 ppm, 97.3 ppm = 154 Hz. HRMS (ESI) m/z: $[M+Na]^+$ calculated for $C_{42}H_{59}N_3O_{19}Na$ 932.3640, found 932.3654.

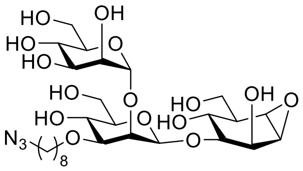

3-*O*-(2-*O*-(α-D-mannopyranosyl)-3-*O*-(8-azidooctyl)-β-D-mannopyranosyl)-β-mannocyclophellitol (**1**). Alkene **32** (17 mg, 19 µmol) was dissolved in MeOH (0.5 ml). A catalytic amount of NaOMe was added and the reaction was monitored by LC/MS. Upon completion the reaction was quenched with AcOH and the solvent was evaporated under reduced pressure. The crude product was dissolved in H_2O (1 ml), NaOH (20 mg, 500 µmol) and magnesium monoperoxyphthalate (80%, 21 mg) were added. The mixture was stirred for 5 hours followed by purification on HW40 (150 mM NH_4HCO_3, H_2O). This yielded the product after elution of the salts (1.83 mg, 15%). ^1H NMR (850 MHz, D_2O) δ 5.04 (d, *J* = 1.7 Hz, 1H, H1"), 4.70 (H1', obscured by HDO), 4.43 (t, *J* = 5.0 Hz, 1H, H2), 4.38 (d, *J* = 2.7 Hz, 1H, H2'), 4.08 (ddd, *J* = 10.2, 5.6, 2.3 Hz, 1H, H5"), 3.95 (dd, *J* = 3.5, 1.7 Hz, 1H, H2"), 3.89 (dd, *J* = 11.1, 4.4 Hz, 1H, H6a), 3.86 – 3.82

(m, 2H, H6a'/H3"), 3.79 (dd, J = 12.1, 2.3 Hz, 1H, H6a"), 3.75 (dd, J = 11.2, 8.0 Hz, 1H, H6b), 3.72 – 3.64 (m, 4H, H3/H6b'/H6b"/spacer), 3.58 (t, J = 8.6 Hz, 1H, H4"), 3.57 – 3.51 (m, 3H, H4'/H4/spacer), 3.47 (dd, J = 4.1, 2.1 Hz, 1H, epoxide), 3.45 – 3.42 (m, 2H, epoxide/H3'), 3.32 (ddd, J = 9.4, 6.7, 2.3 Hz, 1H, H5'), 3.24 (t, J = 6.9 Hz, 2H, CH_2N_3), 2.06 (tdd, J = 8.2, 4.4, 2.2 Hz, 1H, H5), 1.58 – 1.49 (m, 4H, spacer), 1.34 – 1.23 (m, 8H, spacer). ^{13}C NMR (214 MHz, D_2O) δ = 100.4 (C1"), 98.3 (C1'), 81.9 (C3'), 79.1 (C3), 76.8 (C5'), 72.4 (C5"), 71.5 (C2'), 70.6 (spacer), 70.3 (C3"), 70.0 (C2"), 66.7 (C4"), 66.0 (C4'), 64.2 (C4), 63.6 (C2), 61.0 (C6'/C6"), 60.7 (C6), 55.6 (epoxide), 53.4 (epoxide), 51.2 (CH_2N_3), 44.0 (C5), 28.8, 28.3, 28.1, 27.9, 25.8, 25.1 (spacer 6x). $^1J_{H,C}$ (H1") 5.04 ppm, 100.4 ppm = 172 Hz, $^1J_{H,C}$ (H1') 4.70 ppm, 98.3 ppm = 160 Hz. HRMS (ESI) m/z: $[M+Na]^+$ calculated for $C_{27}H_{47}N_3O_{15}Na$ 676.2905, found 676.2914.

Synthesis of probe 2

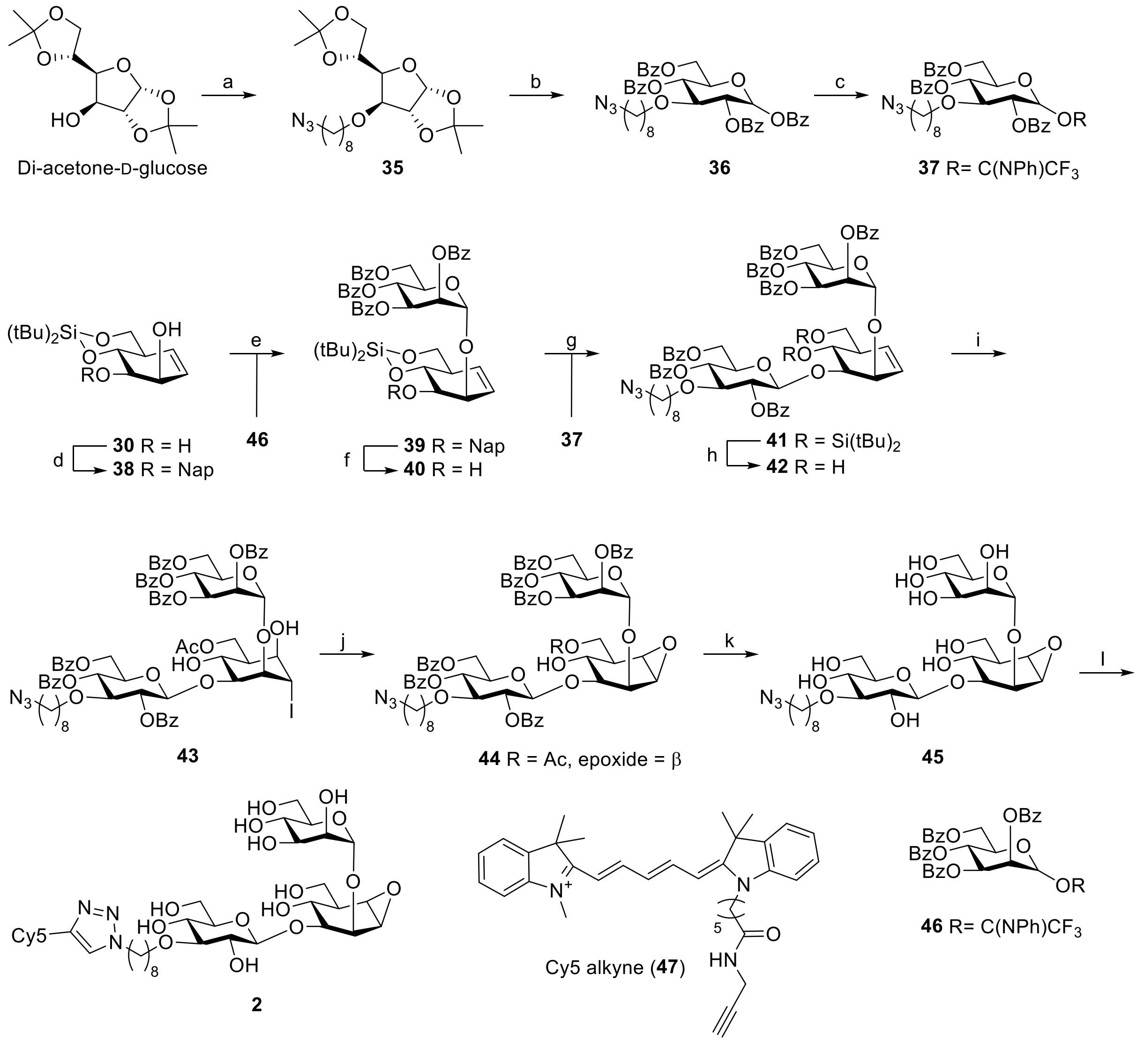

Scheme 2. a) 8-Azido-1-iodooctane, NaH, DMF, 0 °C, 60%; b) i) Amberlite H^+, THF:H_2O, 70 °C; ii) BzCl, DMAP, pyridine, 75%; c) i) $MeNH_2$, THF:DCM, 93%; ii) Cs_2CO_3, 2,2,2-trifluoro-N-phenylacetimidoyl chloride, DCM, 83%; d) i) Bu_2SnO, 140 °C, toluene; ii) NapBr, TBABr, 60 °C, 89%; e) 38, TMSOTf, DCM, 3 Å MS, -60 °C -> -40 °C, 88%; f) DDQ, DCM:phosphate buffer, 98%; g) 35, TMSOTf, DCM, 3 Å MS, -40 °C -> -20 °C, 78%; h) HF·pyridine, THF, 90%; i) NIS, DCM:AcOH, 40 °C, 68% based on recovered starting material; j) DBU, DCM, quant; k) NaOMe, DCM:MeOH, 57%; l) **47**, sodium ascorbate, $CuSO_4$, DMF, quant.

1,2:5,6-di-O-isopropylidene-3-O-(8-azido-octyl)-α-D-glucofuranose (35). 1,2:5,6-di-*O*-isopropylidene-α-D-glucofuranose (1.56 g, 6 mmol, 1 eq.) was coevaporated with toluene (3x) and dissolved in dry DMF (11.1 mL, 0.54 M) under N_2. 8-Azido-1-iodo-octane (2.53 g, 9 mmol, 1.5 eq.) was dissolved in dry DMF (11.1 mL, 0.81 M). The linker solution was added to the furanose solution, and the

mixture was cooled to 0 °C. NaH (0.22 g, 9 mmol, 1.5 eq.) was added portion wise and the mixture was stirred overnight under N_2 atmosphere. Upon completion, the mixture was quenched with H_2O at 0 °C and diluted with Et_2O. The organic layer was separated and washed with 1M HCl (1x), sat. aq. $NaHCO_3$, sat. aq. NaCl, dried over Na_2SO_4 and concentrated *in vacuo*. Purification by column chromatography (10% -> 30% Et_2O in pentane) yielded title compound **35** as a yellowish oil (2.31 g, 5.6 mmol, 60%).^{1}H NMR ($CDCl_3$, 400 MHz) δ = 5.88 (d, J = 3.7 Hz, 1H, H-1), 4.53 (d, J = 3.7 Hz, 1H H-2), 4.30 (dt, 1H, J = 7.4, 6.1, H-5), 4.13 (dd, J = 7.5, 3.1 Hz, 1H, H-4), 4.08 (dd, J = 8.5, 6.2 Hz, 1H, H-6), 3.99 (dd, J = 8.6, 5.9 Hz, 1H, H-6), 3.85 (d, J = 3.1, 1H, H-3), 3.60 (dt, J = 9.3, 6.4 Hz, 1H, H-7), 3.51 (dt, J = 9.3, 6.5, 1H, H-7), 3.26 (t, J = 6.9 Hz, 2H, CH_2N_3), 1.57 (m, 2H CH_2), 1.50 (s, 3H, CH_3 *i*-Pr), 1.43 (s, 3H, CH_3 *i*-Pr), 1.35 (s, 3H CH_3 *i*-Pr), 1.32 (s, 3H CH_3 *i*-Pr). 1.38 – 1.28 (m, 12H, CH_2). ^{13}C-APT NMR (101 MHz, $CDCl_3$) δ 111.9 (quaternary *i*-Pr), 109.0 (quaternary *i*-Pr), 105.4 (C-1), 82.7 (C-2), 82.2 (C-3), 81.2 (C-4), 72.7 (C-5), 70.8 (OCH_2) 67.4 (C-6), 51.6 (CH_3N_3), 29.8 (CH_2), 29.4 (CH_2), 29.2 (CH_2), 29.0 (CH_2), 27.0 (CH_3 *i*-Pr), 26.9 (CH_3 *i*-Pr), 26.8 (CH_2), 26.4 (CH_3 *i*-Pr), 26.1 (CH_2), 25.6 (CH_3 *i*-Pr). HRMS: $[M+Na]^+$ calculated for $C_{20}H_{35}N_3O_6$ 436.2418, found 436.2418.

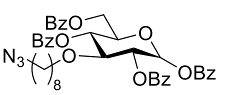

1,2,4,6-*O*-benzoyl-3-*O*-(8-azido-octyl)-D-glucopyranose (**36**). Protected glucofuranoside **35** (2.31, 5.6 mmol, 1 eq.) was dissolved in H_2O:THF (8 mL, 0.2 M, 2:1). Amberlite H^+ exchange resin (4.10 g) was added, and the reaction was stirred overnight at 70 °C. Upon completion, the solid resin was filtered off and the solvents were evaporated. The remaining crude intermediate was coevaporated with toluene (3x) and taken up in pyridine (32 mL, 0.25 M). The solution was cooled to 0 °C and BzCl (9.3 mL, 64 mmol, 8 eq.) and cat. DMAP (0.02 g, 0.16 mmol, 0.02 eq.) were added. The reaction mixture was allowed to reach rt. and was stirred overnight under nitrogen atmosphere. Upon completion, the reaction was quenched with ice water and title compound **36** was recrystallized from EtOAc with pentane. The remaining fraction in solution was diluted with DCM, washed with 1 M HCl (3x), sat. aq. $NaHCO_3$ (1x), sat. aq. NaCl (1x), dried over Na_2SO_4, filtered and concentrated *in vacuo*. Further purification by column chromatography (10% -> 40% Et_2O in pentane) yielded the title compound as a white solid (4.46 g, 6.0 mmol, 75% over 2 steps). ^{1}H NMR (400 MHz, $CDCl_3$) δ = 8.09 – 7.98 (m, 8H, Aromatic), 7.63 – 7.49 (m, 4H, Aromatic), 7.49 – 7.34 (m, 8H, Aromatic), 6.16 (d, J = 7.9 Hz, 1H, H-1), 5.65 (m, 2H, H-2, H-4), 4.61 (dd, J = 12.3, 3.1, 1H, H-6), 4.44 (dd, J = 12.3, 5.1, 1H, H-6), 4.24 (ddd, J = 9.6, 5.0, 3.1 Hz, 1H, H-5), 4.03 (t, J = 7.9 Hz, 1H, H-3), 3.59 (m, 2H, H-7), 3.15 (t, J = 7.0 Hz, 2H, CH_2-Linker), 1.42 (dq, J = 9.0, 7.1 Hz, 2H, CH_2-Linker), 1.36 – 1.26 (m, 2H, CH_2-Linker), 1.08 (q, J = 7.2 Hz, 2H, CH_2), 1.02 (m, 2H, CH_2-Linker), 0.98 – 0.86 (m, 4H, CH_2-Linker). ^{13}C NMR (101 MHz, CDCl3) δ 166.2, 165.0, 164.9 (C=O), 133.8, 133.5, 133.4, 133.0, 130.2, 129.9, 129.8, 129.8, 129.6, 129.3, 129.3, 128.6, 128.5, 128.2 (Aromatic), 92.6 (C-1), 80.6 (C-3), 73.1 (CH_2-Linker), 73.1 (C-5), 72.0 (C-2), 70.3 (C-4), 62.9 (C-6), 51.4 (CH_2-Linker), 30.0, 29.0, 28.9, 28.7, 26.5, 25.8 (CH_2-Linker). HRMS: $[M+NH_4]^+$ calculated for $C_{42}H_{43}N_3O_{10}$ 767.3287, found 767.3287.

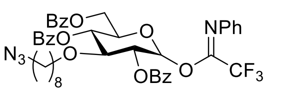

2,4,6-*O*-benzoyl-3-*O*-(8-azido-octyl)-D-glucopyranose (**37**). Perbenzoylated glucopyranoside **36** (0.37 g, 0.5 mmol, 1 eq.) was dissolved in THF: DCM (2.32 mL, 0.22 M, 9:1), $MeNH_2$ (1.38 mL, 2M in toluene, 5.5 eq.) was added and the reaction was stirred for 4 h at rt. under nitrogen atmosphere. Upon completion, the reaction mixture was quenched with 1 M HCl, diluted with DCM, washed with 1 M HCl (3x) and sat. aq. NaCl, dried over Na_2SO_4, filtered and concentrated *in vacuo*. Purification by column chromatography (10% -> 40% EtOAc in pentane) yielded the hemiacetal as a white solid (0.30 g, 0.46 mmol, 93%). ^{1}H NMR (400 MHz, $CDCl_3$, α) δ = 8.13 – 7.98 (m, 6H, H-Aromatic), 7.65 – 7.51 (m, 3H, H-Aromatic), 7.51 – 7.35 (m, 6H, H-Aromatic), 5.63 (t, J = 3.7 Hz, 1H, H-1), 5.52 (dd, J = 10.2, 9.3 Hz, 1H, H-4), 5.16 (ddd, J = 10.0, 3.6, 1.3 Hz, 1H, H-2), 4.60 (dd, J = 12.1, 2.8 Hz, 1H, H-6), 4.51 (ddd, J = 10.2, 4.3, 2.8 Hz, 1H, H-5), 4.36 (dd, J = 12.1, 4.5 Hz, 1H, H-6), 4.22 (t, J = 9.6 Hz, 1H, H-3), 3.69 (dt, J = 9.1, 6.1, 1H, CH_2-Linker), 3.57 (dt, J = 9.2, 6.4 Hz, 1H, CH_2-Linker), 3.15 (t, J = 7.1 Hz, 2H, CH_2-Linker), 3.07 (dd, J = 3.9, 1.3 Hz, 1H, OH), 1.47 – 1.38 (m, 2H, CH_2-Linker), 1.35 – 1.27 (m, 2H, CH_2-Linker), 1.14 – 0.80 (m, 8H, CH_2-Linker).^{13}C NMR (400 MHz, $CDCl_3$) δ 166.5, 165.8, 165.2 (C=O), 133.50, 133.50, 133.2, 130.0, 130.0, 129.8, 129.7, 129.7, 128.7, 128.7, 128.6, 128.5 (Aromatic), 90.7 (C-1), 77.3 (C-3), 73.9 (C-2), 73.6 (CH_2-Linker), 71.1 (C-4), 68.1 (C-5), 63.1 (C-6), 51.5 (CH_2-Linker), 30.3, 29.2, 28.9, 28.8, 26.6, 26.0 (CH_2-Linker). HRMS: $[M+NH4]^+$ calculated for $C_{35}H_{39}N_3O_9$ 663.30246, found 663.30246. The hemiacetal (0.5 mmol, 0.32 g, 1.0 eq.) was coevaporated with toluene (3x) and dissolved in dry DCM (2.5 mL, 0.2 M). 2,2,2-triuoro-N-phenylacetimidoyl chloride (0.75 mmol, 1.32 mL, 1.5 eq.) and Cs_2CO_3 (0.75 mmol, 0.29 g, 1.5 eq.) were added and the reaction mixture was stirred for 4 h under nitrogen atmosphere. Upon completion, the reaction mixture was diluted with DCM,

the Cs_2CO_3 precipitate was filtered using Celite® and the reaction mixture was concentrated in vacuo. Purification by column chromatography (5% -> 20% EtOAc in pentane) yielded title compound **37** as a colorless oil (0.41 mmol, 0.34 g, 83%). ^{1}H NMR (400 MHz, $CDCl_3$): 8.14 – 7.98 (m, 6H), 7.67 – 7.40 (m, 8H), 7.39 – 7.33 (m, 2H), 7.21 (t, J = 7.8 Hz, 2H), 7.14 – 6.98 (m, 2H), 6.69 (d, J = 7.7 Hz, 1H), 5.59 (t, J = 8.4 Hz, 1H), 5.53 (t, J = 8.9 Hz, 1H), 4.61 (dd, J = 12.1, 3.0 Hz, 1H), 4.46 (dd, J = 13.1, 7.1 Hz, 1H), 4.24 – 4.15 (m, 1H), 3.98 (t, J = 8.4 Hz, 1H), 3.64 – 3.51 (m, 2H), 3.16 (t, J = 7.0 Hz, 2H), 1.48 – 1.38 (m, 2H), 1.32 (p, J = 6.7 Hz, 2H), 1.27 –0.71 (m, 8H). ^{13}C NMR (101 MHz, $CDCl_3$): 166.30, 165.12, 133.69, 133.6, 133.25, 129.98, 129.95, 129.91, 129.66, 129.46, 129.30, 128.86, 128.69, 128.50, 119.38, 80.07, 73.44, 72.93, 71.78, 70.15, 63.24, 51.53, 30.11, 29.13, 28.92, 28.83, 26.59, 25.88. HRMS $[M+Na]^+$ calculated 839.2873, found 839.2874.

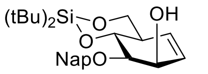

4,6-*O*-di*tert*butylsilyl-3-*O*-naphthyl-D-mannose-cyclohexene (**38**). Compound **30** (0.17 g, 0.55 mmol) was dissolved in toluene (2.8 mL, 0.2 M) and Bu_2SnO (0.14 g, 57 mmol, 1.02 eq.) was added and the reaction mixture was heated to 140 °C. After stirring for 2 hours the reaction mixture was cooled to 60 °C and NapBr (0.13 g, 0.58 mmol, 1.05 eq.), TBABr (0.188 g, 0.58 mmol, 1.050 eq.) were added. After an additional 17 hours TLC showed full conversion, and the reaction mixture was concentrated *in vacuo*. Silica gel column chromatography (10% -> 30% Et_2O in pentane) yielded compound **38** (0.22 g, 0.49 mmol) in 89%. ^{1}H NMR (400 MHz, $CDCl_3$) δ 7.97 – 7.83 (m, 4H, Nap), 7.61 (dd, *J* = 8.5, 1.7 Hz, 1H, Nap), 7.57 – 7.47 (m, 2H, Nap), 5.86 (ddd, *J* = 9.8, 4.8, 3.0 Hz, 1H, H-1), 5.39 (dd, *J* = 9.8, 1.8 Hz, 1H, H-7), 5.27 (d, *J* = 11.9 Hz, 1H, CH_2-Nap), 5.04 (d, *J* = 12.0 Hz, 1H, CH_2-Nap), 4.40 – 4.31 (m, 2H, H-2, H-4), 4.15 (dd, *J* = 10.4, 4.6 Hz, 1H, H-6), 3.90 (dd, *J* = 12.0, 10.4 Hz, 1H, H-6), 3.57 (dd, *J* = 10.1, 4.6 Hz, 1H, H-3), 2.57 (ddtt, *J* = 12.1, 9.0, 3.0, 1.5 Hz, 1H, H-5), 1.12 (d, *J* = 16.8 Hz, 18H, tBu_2Si).^{13}C NMR (101 MHz, $CDCl_3$) δ 136.3, 133.3, 133.2, 128.4, 128.0, 128.0, 127.9, 127.7, 126.7, 126.3, 126.1, 126.1, 125.7 (C-1, C-7, C-Aromatic), 79.8 (C-3), 75.2 (C-4/C-2), 74.2 (CH_2-Nap), 68.5 (C-6), 66.8 (C-4/C-2), 44.8 (C-5), 27.6 (t-Bu), 27.4 (t-Bu), 22.9 (C-q), 20.0 (C-q). HRMS $[M+Na]^+$ calculated 463.2277, found 463.2275.

2,3,4,6-tetra-O-benzoyl-α-D-mannose-(1->2)-4,6-O-ditertbutylsilyl-3-O-naphthyl-D-mannose-cyclohexene (**39**). Acceptor **38** (0.46 g, 1.05 mmol, 1 eq.) and donor **46** (1.45 g, 1.9 mmol, 1.5 eq.) were co-evaporated 3 times with toluene and dissolved in dry DCM (10.5 mL, 0.1 M) under N_2. 3 Å MS were added and the reaction mixture was stirred for 1 hour. Subsequently the mixture was cooled to -60 degrees and TMSOTf (0.019 mL, 0.1 mmol) was added. The reaction mixture was allowed to reach -40 °C. After stirring for 2.5 hours the reaction was quenched with Et_3N at -40 °C. The reaction mixture was diluted with DCM, washed with sat. aq. $NaHCO_3$, brine, dried over $MgSO_4$, filtrated and concentrated *in vacuo*. Column chromatography (10% -> 30% Et_2O in pentane) yielded compound **39** (0.94 g, 0.92 mmol) in 88%.^{1}H NMR (500 MHz, $CDCl_3$) δ 8.21 – 7.24 (m, 27H, H-Aromatic), 6.08 (t, *J* = 9.8 Hz, 1H, H-4'), 6.01 (dd, *J* = 10.0, 3.2 Hz, 1H, H-3'), 5.96 (dd, *J* = 3.2, 1.8 Hz, 1H, H-2'), 5.80 (ddd, *J* = 9.6, 5.1, 2.9 Hz, 1H, H-1), 5.62 (d, *J* = 1.8 Hz, 1H, H-1'), 5.46 (dd, *J* = 9.7, 1.9 Hz, 1H, H-7), 5.30 (d, *J* = 12.0 Hz, 1H, CH_2-Nap), 4.97 (d, *J* = 12.1 Hz, 1H, CH_2-Nap), 4.76 – 4.70 (m, 1H, H-6'), 4.56 – 4.49 (m, 3H, H-4, H-5', H-6'), 4.49 – 4.45 (m, 1H, H-2), 4.20 (dd, *J* = 10.5, 4.6 Hz, 1H, H-6), 4.05 (dd, *J* = 12.0, 10.5 Hz, 1H, H-6), 3.65 (dd, *J* = 10.2, 4.3 Hz, 1H, H-3), 2.66 – 2.52 (m, 1H, H-5), 1.21 (s, 9H, tBu), 1.13 (s, 9H, tBu). ^{13}C NMR (126 MHz, $CDCl_3$) δ 166.3, 165.6, 165.5, 165.0 (C=O), 136.7, 133.5, 133.3, 133.1, 133.1, 132.9, 130.0, 130.0, 129.9, 129.9, 129.8, 129.8, 129.7, 129.4, 129.1, 128.8, 128.6, 128.5, 128.5, 128.5, 128.3, 128.2, 128.0, 127.7, 126.7, 126.5, 126.0, 125.9, 125.6 (C-1, C-7, C-Aromatic), 99.4 (C-1'), 80.3 (C-3), 75.7 (C-4/C-5'), 74.5 (CH_2-Nap), 73.6 (C-2), 70.4 (C-2'), 70.2 (C-3'), 69.2 (C-4/C-5'), 68.3 (C-6), 67.5 (C-4'), 63.3 (C-6'), 45.3 (C-5), 27.7 (tBu), 27.4 (tBu), 22.9 (C-Quaternary), 20.0 (C-Quaternary). HRMS $[M+Na]^+$ calculated 1041.385, found 1041.385.

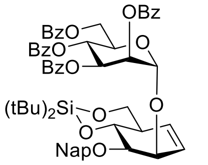

2,3,4,6-tetra-*O*-benzoyl-α-D-mannose-(1->2)-4,6-*O*-di*tert*butylsilyl-D-mannose-cyclohexene (**40**). Compound **39** (0.1 g, 0.1 mmol) was dissolved in DCM:phosphate buffer (1 mL, 0.1 M, 4:1, [buffer: 410 mL 1 M NaOH, 250 mL 0.2 M KH_2PO_4, 340 mL H_2O]) and DDQ (0.045 g, 0.2 mmol, 2 eq.) was added. The reaction was left to stir in the dark for 2 hours before TLC showed full conversion. The reaction mixture was then diluted with EtOAc, washed with sat. aq. $NaHCO_3$, sat. aq. $Na_2S_2O_3$, brine, dried over $MgSO_4$, filtrated and concentrated *in vacuo*. Column chromatography (10% -> 40% Et_2O in pentane) yielded compound **40** (0.086 g, 0.098 mmol) in 98%. ^{1}H NMR (400 MHz, $CDCl_3$) δ 8.18 – 7.24 (m, 20H, H-Aromatic), 6.11 (t, *J* = 9.6 Hz, 1H, H-4'), 5.99 (dd, *J* = 10.1, 3.3 Hz, 1H, H-3'), 5.91 (dd, *J* = 3.3, 1.8 Hz, 1H, H-2'), 5.87 (ddd, *J* = 9.8, 5.1, 2.9 Hz, 1H, H-1), 5.58 (d, *J* = 1.8 Hz, 1H, H-1'), 5.51 (dd, *J* = 9.8, 1.8 Hz, 1H, H-7), 4.79 – 4.71 (m, 1H, H-6'), 4.60 – 4.56 (m, 1H, H-2), 4.56 – 4.50 (m, 2H, H-5',

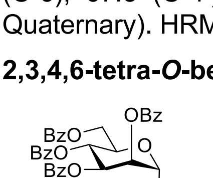

H-6’), 4.26 – 4.14 (m, 2H, H-4, H-6), 4.01 (dd, *J* = 12.0, 10.4 Hz, 1H, H-6), 3.78 (dd, *J* = 10.2, 4.3 Hz, 1H, H-3), 3.04 (s, 1H, 3-OH), 2.60 – 2.46 (m, 1H, H-5), 1.16 (s, 9H, tBu), 1.06 (s, 9H, tBu).^{13}C NMR (101 MHz, $CDCl_3$) δ 166.3, 165.6, 165.5, 165.4 (C=O), 133.5, 133.5, 133.2, 130.0, 129.9, 129.8, 129.6, 129.3, 129.1, 128.7, 128.6, 128.6, 128.5, 128.4 (C-7, C-Aromatic), 126.6 (C-1), 99.2 (C-1’), 74.3 (C-3), 74.2 (C-4), 72.6 (C-2), 70.4 (C-2’), 70.1 (C-3’), 69.1 (C-5’), 67.9 (C-6), 67.4 (C-4’), 63.2 (C-6’), 44.2 (C-5), 27.6 (tBu), 27.2 (tBu), 22.9 (C-Quaternary), 20.0 (C-Quaternary). HRMS [M+Na]$^+$ calculated 901.3227, found 901.3226.

[2,3,4,6-tetra-*O*-benzoyl-α-D-mannose-(1->2)]-[2,4,6-*O*-benzoyl-3-*O*-(8-azido-octane)-β-D-glucose-(1->3)]-4,6-*O*-di*tert*butylsilyl-D-mannose-cyclohexene (**41**). Acceptor **40** (0.35 g, 0.4 mmol) and donor **37** (0.59 g, 0.72 mmol, 1.8 eq.) were co-evaporated 3 times with toluene. The mixture was then dissolved in dry DCM (4 mL, 0.1 M), put under N_2 and 3 Å MS were added. After stirring for 75 min the reaction mixture was cooled to -40 °C and TMSOTf (0.1 mL, 0.8 M, 0.2 eq.). After stirring for 2.5h TLC showed almost complete conversion and the reaction mixture was allowed to warm to -20 °C, after stirring for an additional hour, TLC indicated no further conversion, and the reaction was quenched with Et_3N. Column chromatography (10% -> 40% Et_2O in pentane) yielded compound **41** (0.47 g, 0.31 mmol) in 78%. ^{1}H NMR (500 MHz, $CDCl_3$) δ 8.29 – 6.99 (m, 35H, H-Arom), 6.02 (t, *J* = 9.9 Hz, 1H, H-4’), 5.90 (dd, *J* = 10.0, 3.3 Hz, 1H, H-3’), 5.83 (dd, *J* = 3.3, 1.8 Hz, 1H, H-2’), 5.73 (ddd, *J* = 9.7, 5.2, 2.9 Hz, 1H, H-1), 5.63 (t, *J* = 9.0 Hz, 1H, H-4”), 5.47 (d, *J* = 1.8 Hz, 1H, H-1’), 5.43 (dd, *J* = 8.5, 6.9 Hz, 1H, H-2”), 5.37 (dd, *J* = 9.7, 1.9 Hz, 1H, H-7), 5.26 (d, *J* = 6.9 Hz, 1H, H-1”), 4.60 – 4.51 (m, 3H, H-6’, H-6”(2x)), 4.45 – 4.36 (m, 3H, H-4, H-5’, H-6’), 4.30 (t, *J* = 4.8 Hz, 1H, H-2), 4.14 (dd, *J* = 10.5, 4.6 Hz, 1H, H-6), 4.06 (dt, *J* = 8.6, 4.1 Hz, 1H, H-5”), 4.01 (t, *J* = 8.6 Hz, 1H, H-3”), 3.96 (dd, *J* = 12.1, 10.5 Hz, 1H, H-6), 3.91 (dd, *J* = 10.2, 4.2 Hz, 1H, H-3), 3.59 – 3.48 (m, 2H, OCH_2-Linker), 3.13 (t, *J* = 7.1 Hz, 2H, N_3CH_2-Linker), 2.52 (s, 1H, H-5), 1.45 – 1.35 (m, 2H, CH_2-Linker), 1.35 – 1.19 (m, 2H, CH_2-Linker), 1.12 – 0.96 (m, 22H, tBu, CH2-Linker), 0.95 – 0.89 (m, 4H, CH_2-Linker).^{13}C NMR (126 MHz, $CDCl_3$) δ 166.3, 165.7, 165.3, 165.3, 165.2, 165.0 (C=O), 133.4, 133.3, 133.3, 133.2, 133.1, 133.1, 132.8, 130.2, 130.1, 130.0, 130.0, 129.9, 129.9, 129.8, 129.8, 129.5, 129.2, 128.7, 128.6, 128.6, 128.5, 128.3, 128.2 (C-7, C-Aromatic), 126.7 (C-1), 101.1 (C-1”), 99.3 (C-1’), 80.2 (C-3”), 80.1 (C-3), 74.5 (C-2”), 74.0 (C-2), 73.5 (C-4/C-5’), 72.8 (C-5”), 71.8 (OCH_2-Linker), 70.7 (C-2’), 70.5 (C-4”), 69.9 (C-3’), 69.1 (C-4/C-5’), 68.4 (C-6), 67.7 (C-4’), 63.7 (C-6”), 63.5 (C-6’), 51.5 (N_3CH_2-Linker), 45.4 (C-5), 30.1, 29.1, 28.9, 28.8, 27.6, 27.4, 26.6, 25.8 (CH_2-linker 6x, tBu 2x), 22.9 (C- Quaternary), 20.0 (C- Quaternary). HRMS [M+Na]$^+$ calculated 1528.582, found 1528.581.

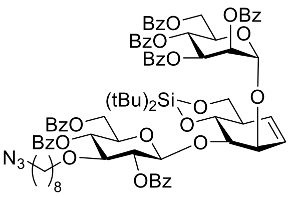

[2,3,4,6-tetra-*O*-benzoyl-α-D-mannose-(1->2)]-[2,4,6-*O*-benzoyl-3-*O*-(8-azido-octyl)-β-D-glucose-(1->3)]-D-mannose-cyclohexene (**42**). Compound **41** (0.31 mmol, 0.47 g) was co-evaporated 3 times with toluene and dissolved in dry THF (1.6 mL, 0.2M) under N_2. The solution was cooled to 0 °C and HF·pyridine (0.64 mL, 24.6 mmol HF, 61 eq., 70% HF, 30% pyridine) was added. The ice bath was removed and after stirring for 3 hours at room temperature TLC indicated full conversion and the mixture was diluted with DCM. The organic layer was washed with 1 M HCl, sat. aq. $NaHCO_3$, brine, dried over $MgSO_4$, filtrated and concentrated *in vacuo*. Column chromatography (30% -> 70% EtOAc in pentane) yielded compound **42** (0.38 g, 0.28 mmol) in 90%.^{1}H NMR (500 MHz, $CDCl_3$) δ 8.34 – 7.10 (m, 35H, H-Aromatic), 6.00 (t, *J* = 9.9 Hz, 1H, H-4’), 5.85 (dd, *J* = 9.9, 3.3 Hz, 1H, H-3’), 5.82 (dd, *J* = 3.4, 1.8 Hz, 1H, H-2’), 5.73 (ddd, *J* = 9.8, 5.4, 2.8 Hz, 1H, H-1), 5.64 (dd, *J* = 9.8, 2.2 Hz, 1H, H-7), 5.45 (dd, *J* = 9.9, 9.0 Hz, 1H, H-4”), 5.38 (dd, *J* = 9.4, 7.9 Hz, 1H, H-2”), 5.21 (d, *J* = 1.7 Hz, 1H, H-1’), 4.97 (d, *J* = 7.9 Hz, 1H, H-1”), 4.73 (dd, *J* = 12.1, 2.5 Hz, 1H, H-6”), 4.53 (dd, *J* = 11.7, 2.5 Hz, 1H, H-6’), 4.42 – 4.34 (m, 2H, H-5’, H-6”), 4.34 – 4.30 (m, 1H, H-6’), 4.21 – 4.16 (m, 1H, H-4), 4.16 – 4.11 (m, 2H, H-2, H-5”), 4.01 (t, *J* = 9.2 Hz, 1H, H-3”), 3.87 (dd, *J* = 10.8, 7.6 Hz, 1H, H-6), 3.81 (dd, *J* = 11.0, 4.3 Hz, 1H, H-6), 3.72 (dd, *J* = 10.1, 3.8 Hz, 1H, H-3), 3.50 (qt, *J* = 9.0, 6.2 Hz, 2H, OCH_2-Linker), 3.12 (t, *J* = 7.0 Hz, 2H, N_3CH_2-Linker), 2.44 – 2.34 (m, 1H, H-5), 1.43 – 1.32 (m, 2H, CH_2-Linker), 1.25 – 1.18 (m, 2H, CH_2-Linker), 1.08 – 1.00 (m, 2H, CH_2-Linker), 0.97 – 0.81 (m, 6H, CH_2-Linker).^{13}C NMR (126 MHz, $CDCl_3$) δ 166.3, 166.3, 165.7, 165.6, 165.2, 165.2, 164.9 (C=O), 133.7, 133.5, 133.4, 133.4, 133.3, 133.2 (C-Aromatic), 131.7 (C-7), 130.1, 130.1, 130.0, 130.0, 129.9, 129.9, 129.8, 129.5, 129.5, 129.3, 129.3, 129.1, 128.7, 128.6, 128.5, 128.5, 128.4 (C-Aromatic), 125.4 (C-1), 102.6 (C-1”), 99.1 (C-1’), 85.9 (C-3), 80.2 (C-3”), 73.6 (C-2”), 73.3 (C-2/C-5”), 73.1 (C-2/C-5”), 72.1 (OCH_2-Linker), 70.5 (C-4”), 70.2 (C-4/C-2’/C-3’), 70.1 (C-4/C-2’/C-3’), 70.0 (C-4/C-2’/C-3’), 69.0 (C-5’), 67.5 (C-4’), 66.0 (C-6), 63.6 (C-6”),

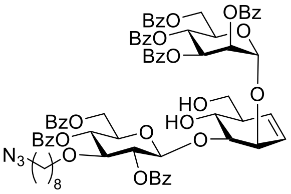

63.4 (C-6'), 51.5 (N_3CH_2-Linker), 46.7 (C-5), 30.0, 29.0, 28.8, 28.7, 26.5, 25.8 (CH_2-Linker). HRMS $[M+Na]^+$ calculated 1388.480, found 1388.479.

6-*O*-acetyl-[2,3,4,6-tetra-*O*-benzoyl-α-D-mannose-(1->2)]-[2,4,6-*O*-benzoyl-3-O-*(*8-azido-octyl)-β-D-glucose-(1->3)]-(1,7-trans-iodohydrin)-D-mannose-cyclohexane (**43**). Compound **42** (0.05 g, 0.037 mmol) was dissolved in dry DCM: AcOH (0.37 mL, 0.1 M, 2:1), NIS (0.033 g, 0.15 mmol, 4 eq.) was added and the mixture was heated to 40 °C. After stirring for 4.5 hours TLC indicated conversion and the reaction mixture was diluted with EtOAc, washed with sat. aq. $Na_2S_2O_3$, sat. aq. $NaHCO_3$, brine, dried over $MgSO_4$ and concentrated *in vacuo*. Column chromatography (10% -> 50% EtOAc in pentane) yielded compound **43** (9.7 mg, 6.25 µmol, 17%) and starting material **23** (0.037 g, 0.027 mmol, 75%). HRMS $[M+Na]^+$ calculated 1574.398, found 1574.396.

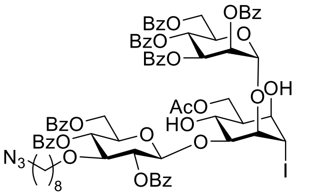

6-O-acetyl-[2,3,4,6-tetra-*O*-benzoyl-α-D-mannose-(1->2)]-[2,4,6-*O*-benzoyl-3-*O*-(8-azido-octyl)-β-D-glucose-(1->3)]-β-D-mannose-cyclophelllitol (**44**). Compound **43** (9.7 mg, 6.25 µmol) was dissolved in dry DCM (0.21 mL, 0.03M) and DBU (0.1 mL, 0.12 M, 2 eq.) was added. After stirring for 2 h TLC indicated full conversion, and the reaction mixture was diluted with DCM. The organic layer was washed with 1 M HCl, brine, dried over $MgSO_4$, filtrated and concentrated *in vacuo*. Product **44** was yielded quantitatively without the need for further purification. ^{1}H NMR (600 MHz, $CDCl_3$) δ = 8.19 –7.17 (m, 35H, H-Aromatic), 6.07 (t, *J*=10.2, 1H, H-4'), 5.92 (dd, *J*=10.1, 3.4, 1H, H-3'), 5.83 (dd, *J*=3.4, 1.7, 1H, H-2'), 5.46 (dd, *J*=9.9, 9.0, 1H, H-4"), 5.38 (dd, *J*=9.3, 7.7, 1H, H-2"), 5.10 (d, *J*=1.7, 1H, H-1'), 5.00 (d, *J*=7.7, 1H, H-1"), 4.74 (ddd, *J*=10.2, 4.5, 2.4, 1H, H-5'), 4.72 – 4.67 (m, 2H, H-6, H-6"), 4.60 (dd, *J*=12.2, 2.4, 1H, H-6'), 4.43 – 4.37 (m, 2H, H-6', H-6"), 4.29 (dd, *J*=11.0, 8.8, 1H, H-6), 4.14 (ddd, *J*=9.9, 7.1, 2.7, 1H, H-5"), 4.11 (t, *J*=5.1, 1H, H-2), 4.07 (t, *J*=9.1, 1H, H-3"), 3.83 (t, *J*=9.4, 1H, H-4) 3.59 (dd, *J*=9.8, 5.1, 1H, H-3), 3.57 – 3.49 (m, 3H, OCH_2-Linker), 3.23 (dd, *J*=5.0, 3.5, 1H, H-1), 3.16 (dd, *J*=3.5, 2.0, 1H, H-7), 3.13 (t, *J*=7.1, 2H, N_3CH_2-Linker), 2.15 (s, 3H, CH_3 Acetyl), 2.15 – 2.11 (m, 1H, H-5), 1.42 – 1.35 (m, 2H, CH_2-Linker), 1.26 – 1.22 (m, 2H, CH_2-Linker), 1.08 – 1.01 (m, 2H, CH_2-Linker), 0.99 – 0.93 (m, 2H, CH_2-Linker), 0.93 – 0.83 (m, 4H, CH_2-Linker).^{13}C NMR (151 MHz, $CDCl_3$) δ 171.1, 166.3, 166.2, 165.7, 165.3, 165.3, 165.2, 165.1 (C=O), 133.6, 133.5, 133.5, 133.4, 133.4, 133.2, 133.1, 130.2, 130.1, 130.1, 130.0, 130.0, 130.0, 130.0, 130.0, 129.9, 129.9, 129.8, 129.8, 129.6, 129.5, 129.4, 129.4, 129.1, 128.7, 128.7, 128.6, 128.6, 128.6, 128.5, 128.5, 128.5, 128.4 (C-Aromatic), 101.1 (C-1'), 100.8 (C-1"), 81.4 (C-3), 80.2 (C-3"), 74.9 (C-2), 73.7 (C-2"), 73.1 (C-5"), 72.4 (OCH_2-Linker), 70.7 (C-4"), 70.6 (C-2'), 70.1 (C-3'), 69.6 (C-5'), 67.1 (C-4'), 64.0 (C-4), 63.5 (C-6"), 63.2 9C-6), 63.1 (C-6'), 53.9 (C-7), 52.1 (C-1), 51.5 (N_3CH_2-Linker), 42.4 (C-5), 29.8, 29.1, 28.8, 28.8, 26.5, 25.8 (CH_2-Linker), 21.1 (CH_3-Acetyl). HRMS $[M+Na]^+$ calculated 1446.485, found 1446.484.

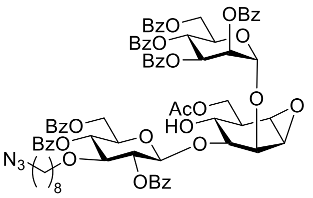

[α-D-mannose-(1->2)]-[3-O-(8-azido-octyl)-β-D-glucose-(1->3)]-β-D-mannose-cyclophelllitol (**45**). Compound **44** (5.1 mg, 3.6 µmol) was dissolved in DCM: MeOH (1 mL, 1.8 mM) and 1 mL of a pH 10 solution of NaOMe in MeOH was added. After stirring for 44 hours TLC-MS indicated one Bz remained. Additional NaOMe (10 µL, 4.37 M) was added and after stirring for an additional 5 hours, LC-MS indicated full conversion. The reaction mixture was quenched with AcOH and concentrated *in vacuo*. Reversed phase column chromatography over a prepacked C18 silica column (MeCN in H_2O, 0% -> 50%) yielded compound **45** (1.35 mg, 2.07 µmol) in 57%.^{1}H NMR (850 MHz, D_2O) δ = 5.08 (d, *J*=1.7, 1H, H-1'), 4.52 – 4.48 (m, 2H, H-2, H-1"), 4.02 (dd, *J*=3.4, 1.7, 1H, H-2'), 3.93 (dd, *J*=11.3, 4.5, 1H, H-6), 3.88 – 3.87 (m, 1H, H-3), 3.87 – 3.85 (m, 1H, H-6'), 3.85 – 3.84 (m, 1H, H-3'), 3.82 (ddd, *J*=10.0, 6.1, 2.1, 1H, H-5'), 3.80 – 3.75 (m, 4H, H-6, H-6" OCH_2-Linker), 3.73 (dd, *J*=12.2, 6.1, 1H, H-6'), 3.68 (dd, *J*=12.4, 5.7, 2H, H-6"), 3.64 – 3.59 (m, 2H, H-4, H-4'), 3.57 (t, *J*=4.4, 1H, H-1), 3.52 (dd, *J*=4.0, 2.2, 1H, H-7), 3.43 – 3.38 (m, 3H, H-4", H-5"), 3.33 – 3.30 (m, 2H, H-2", H-3"), 3.27 (t, *J*=6.9, 2H, N_3-CH_2-Linker), 2.11 (tdd, *J*=8.2, 4.5, 2.3, 1H, H-5), 1.60 – 1.53 (m, 4H, CH_2-Linker), 1.35 – 1.27 (m, 8H, CH_2-Linker).^{13}C NMR (214 MHz, D_2O) δ 102.9 (C-1'), 102.2 (C-1"), 85.3 (C-2"/C-3"), 79.3 (C-3), 76.7 (C-4"/C-5"), 74.4 (CH_2-Linker), 74.3 (C-5'), 73.6 (C-2"/C-3"), 72.1 (C-2), 71.3 (C-3'), 70.6 (C-2'), 70.0 (C-4"/C-5"), 67.6 (C-4/C-4'), 65.0 (C-4/C-4'), 61.9 (C-6'), 61.5 (C-6/C-6"), 61.4 (C-6/C-6"), 56.5 (C-7), 54.3 (C-1), 52.1 (N_3CH_2-Linker, 44.8 (C-5), 30.2, 29.3, 29.1, 28.9, 26.8, 25.9 (CH_2-Linker). HRMS $[M+Na]^+$ calculated 676.2901, found 676.2899.

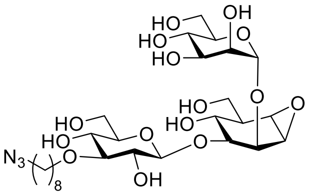

[α-D-mannose-(1->2)]-[3-*O*-(8-triazole-Cy5-octane)-β-D-glucose-(1->3)]-β-D-mannose-cyclophelllitol (**2**). Compound **45** (1.06 mg, 1.6 μmol) was dissolved in DMF (0.1 mL, 0.1 mL/mg) and Cy5 alkyne tag (**47**) (33 μL, 0.054 M in DMF) was added. Then 66 μL of a solution of sodium ascorbate (0.1 M) and $CuSO_4$ (0.073 M) in DMF was added. After stirring for 2.8 hours LC-MS indicated almost complete conversion and 10 μL of the sodium ascorbate/$CuSO_4$ solution was added. After stirring for an additional hour LC-MS indicated full conversion and the reaction mixture was diluted with water and freeze dried overnight. Reversed phase column chromatography (C18 silica) (10% -> 50% MeCN in H_2O then 20% -> 40% MeCN in H_2O with 1% AcOH) yielded compound **2** quantitatively with small impurities.

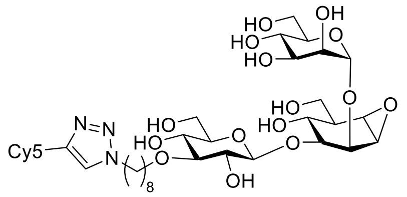

^{1}H NMR (850 MHz, D_2O) δ = 8.00 (t, *J*=13.2, 2H), 7.52 (t, *J*=7.3, 3H), 7.48 – 7.36 (m, 3H), 7.35 – 7.20 (m, 5H), 6.51 (t, *J*=12.3, 1H), 6.21 (dd, *J*=28.7, 13.8, 2H), 5.13 (s, 1H), 4.53 (t, *J*=5.1, 1H), 4.50 (d, *J*=7.6, 1H), 4.38 (s, 2H), 4.33 (t, *J*=6.9, 2H), 4.12 (q, *J*=6.9, 2H), 4.07 (dt, *J*=5.3, 2.6, 1H), 4.04 (t, *J*=7.2, 2H), 3.97 (dd, *J*=11.3, 4.5, 2H), 3.92 – 3.85 (m, 6H), 3.83 (dd, *J*=11.2, 8.0, 1H), 3.78 (dd, *J*=12.3, 6.1, 2H), 3.76 – 3.69 (m, 5H), 3.69 – 3.64 (m, 1H), 3.63 – 3.58 (m, 5H), 3.57 – 3.55 (m, 1H), 3.42 (d, *J*=7.3, 2H), 3.38 – 3.26 (m, 2H), 2.30 – 2.20 (m, 28H), 2.19 – 2.13 (m, 1H), 1.92 (d, *J*=0.8, 29H), 1.79 (dt, *J*=24.7, 7.4, 4H), 1.70 – 1.58 (m, 18H), 1.49 (p, *J*=6.9, 3H), 1.37 – 1.29 (m, 10H), 1.24 – 1.08 (m, 10H). ^{13}C NMR (214 MHz, D_2O) δ 144.7, 144.6, 142.8, 142.1, 141.3, 141.2, 129.5, 128.5, 126.7, 125.0, 124.9, 122.3, 122.2, 120.4, 110.9, 110.8, 103.0, 102.8, 101.9, 101.4, 96.6, 84.3, 78.4, 75.9, 73.3, 73.3, 72.6, 71.1, 70.4, 69.8, 69.1, 68.5, 66.7, 64.2, 61.9, 61.0, 60.6, 60.6, 55.5, 53.3, 50.3, 49.0, 43.9, 43.5, 35.2, 34.3, 33.7, 30.7, 30.2, 29.3, 29.2, 28.2, 27.8, 26.9, 26.7, 26.4, 25.4, 25.2, 24.9, 23.2, 20.0, 16.6. HRMS $[M]^+$ calculated 1173.634, found 1173.633.

Synthesis of probe 3

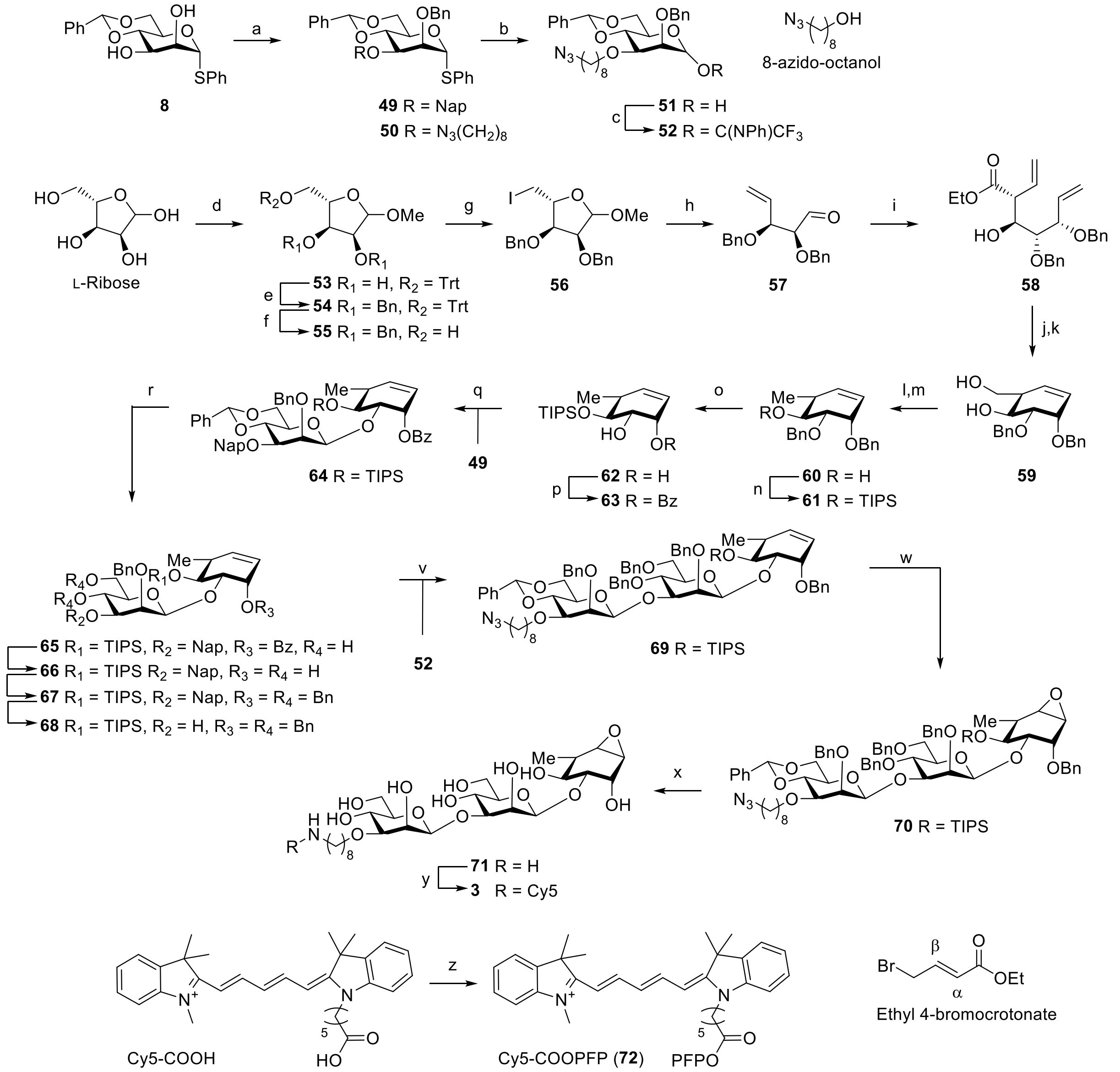

Scheme 3. a) For **49**; i) Bu_2SnO, toluene, 120 °C; ii) NapBr, CsF, DMF, 93%; iii) BnBr, NaH, DMF, 96%; for 50; i) 8-azidooctyl trifluoromethanesulfonate, 2-aminoethyl diphenylborinate, K_2CO_3, MeCN; ii) BnBr, NaH, TBAI, DMF, 81% over 2 steps; b) NIS, TFA, DCM, 90%; c) 2,2,2-trifluoro-N-phenylacetimidoyl chloride, Cs_2CO_3, DCM, 63%; d) i) AcCl, MeOH; ii) TrtCl, Et_3N, DMF, 75%; e) BnBr, NaH, DMF, 86%; f) pTsOH, DCM:MeOH, 95%; g) PPh_3, I_2, THF, 65 °C, 94%; h) Zn, THF:H_2O, sonication, 60 °C, 71%; i) Bromocrotonate, $La(OTf)_3$, In, H_2O, 75%; j) Grubbs 2nd generation, DCM, N_2, 40 °C, 75%; k) i) DIBAL-H, THF; ii) EtOAc, H_2O, $NaHB_4$, 85%; l) TsCl, Et_3N, 78%; m) $LiAlH_4$, THF, 80%; n) TIPSOTf, 2,6-lutidine, DCM, 0 °C, 98%; o) $TiCl_4$, DCM, 79%; p) DIPEA, BzCl, 2-ADB, MeCN, 81% q) Tf_2O, BSP, TTBP, DCM, 3 Å MS, -60 °C, quant; r) AcOH:H_2O, 4:1, 80 °C, 90%; s) NaOMe, MeOH, 74%; t) BnBr, NaH, DMF, 97%; u) DDQ, DCM:phosphate buffer 9:1, 55%; v) TMSOTf, DCM, 3 Å MS, -60 °C -> -20, 27%, 77% brsm; w) Oxone®, $F_3C(C{=}O)CH_3$, $NaHCO_3$, MeCN, 0.4 mM EDTA, 33%, 78% brsm; x) i) TBAF, THF; ii) PtO_2, H_2, THF; iii) Na, NH_3, t-BuOH, THF, -60 °C, 36% over 3 steps; y) Cy5-CO_2PFP, DMF, DIPEA, H_2O, 81%; z) PFP-TFA, DIPEA, DMF.

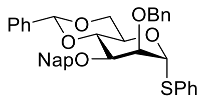

2-*O*-benzyl-4,6-*O*-benzylidene-3-*O*-naphthyl-1-deoxy-1-thiophenol-α-D-mannose (**49**). Compound **8**[13] (38.7 g, 107 mmol) was suspended in toluene and dibutyltin oxide (26.7 g, 107 mmol, 1 eq.) was added. The suspension was refluxed using a dean-stark apparatus. After refluxing for 2.5 hours the reaction mixture was concentrated *in vacuo* and redissolved in dry DMF under N_2. NapBr (35.5 g, 161 mmol, 1.5 eq.) and CsF (24.4 g, 161 mmol, 1.5 eq.) were added and the reaction was left to stir at room temperature. After stirring for 67 hours TLC showed complete conversion and the reaction mixture was diluted with DCM and washed with sat. aq. $NaHCO_3$. Subsequently, the organic layer and water layer were filtered, and

the H_2O layer was re-extracted with DCM and the combined organic layers were washed with brine, dried over $MgSO_4$, filtrated and concentrated *in vacuo*. Column chromatography (5% -> 100% EtOAc in pentane) yielded **8a** (50 g, 107 mmol) in 93%.^{1}H NMR (500 MHz, $CDCl_3$) δ = 7.82 – 7.69 (m, 4H, Aromatic), 7.54 – 7.17 (m, 13H, Aromatic), 5.60 (s, 1H, H-Benzylidene), 5.57 (d, *J*=1.2, 1H, H-1), 4.98 (d, *J*=12.1, 1H, CH_2-Nap), 4.87 (d, *J*=12.1, 1H, CH_2-Nap), 4.32 (td, *J*=9.8, 4.9, 1H, H-5), 4.27 (d, *J*=3.4, 1H, H-2), 4.24 – 4.16 (m, 2H, H-4, H-6), 3.99 (dd, *J*=9.6, 3.3, 1H, H-3), 3.84 (t, *J*=10.3, 1H, H-6), 3.06 (d, *J*=1.6, 1H, 2-OH).^{13}C NMR (126 MHz, $CDCl_3$) δ 137.6, 135.2, 133.4, 133.3, 133.2, 132.9, 131.7, 129.2, 129.1, 128.4, 128.4, 128.1, 127.8, 127.7, 127.2, 126.8, 126.3, 126.3, 126.2, 125.8 (C-Aromatic), 101.8 (benzylidene), 87.9 (C1), 79.0 (C4), 75.9 (C-3), 73.2 (CH_2-Nap), 71.4 (C2), 68.6 (C6), 64.8 (C5). HRMS $[M+Na]^+$ calculated 523.155, found 523.155. Compound **8a** (5 g, 10 mmol) was dissolved in DMF (50 mL, 0.2 M) under N_2 and cooled to 0 °C and BnBr (1.8 mL, 15 mmol, 1.5 eq.), NaH (0.6 g, 15 mmol, 1.5 eq., 60 wt.%) were added. The reaction was left to reach room temperature. After stirring for 18 hours TLC showed full conversion and the reaction mixture was cooled to 0 °C and quenched with MeOH. The reaction mixture was then diluted with Et_2O and the organic layer was washed with H_2O. The water layer was washed with Et_2O (4x) and the combined organic layers were dried over $MgSO_4$, filtrated and concentrated *in vacuo*. Column chromatography (5% -> 40 % Et_2O in pentane) yielded compound **49** (5.6 g, 9.6 mmol) in 96% yield. ^{1}H NMR (400 MHz, $CDCl_3$) δ = 7.86 – 7.69 (m, 4H, H-aromatic), 7.56 – 7.26 (m, 18H, H-Aromatic), 5.68 (s, 1H, H-Benzylidene), 5.52 (d, *J*=1.4, 1H, H-1), 4.95 (d, *J*=12.5, 1H, CH_2-Nap), 4.81 (d, *J*=12.5, 1H, CH_2-Nap), 4.75 (m, 2H, CH_2-Bn), 4.39 – 4.31 (m, 1H, H-5), 4.31 – 4.21 (m, 2H, H-6), 4.07 (dd, *J*=3.2, 1.4, 1H, H-2), 4.03 (dd, *J*=9.5, 3.2, 1H, H-3), 3.90 (t, *J*=9.9, 1H, H-4).^{13}C NMR (101 MHz, $CDCl_3$) δ 131.8, 129.3, 128.6, 128.4, 128.3, 128.1, 128.0, 127.8, 127.8, 126.4, 126.3, 126.0, 125.8 (C-Aromatic), 101.7 (Benzylidene), 87.2 (C-1), 79.2 (C-4), 78.1 (C-2), 76.4 (C-3), 73.2 (CH_2-Nap/Bn), 73.1 (CH_2-Nap/Bn), 68.7 (C-6), 65.6 (C-5).

2-*O*-benzyl-4,6-*O*-benzylidene-3-*O*-(8-azido-octane)-1-deoxy-1-thiophenol-α-D-mannose (**50**). Tf_2O (3 mL, 18 mmol, 1.2 eq.) was dissolved in dry DCM (37.5 mL, 0.48 M) and cooled to -20 °C. To this

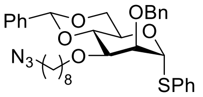

solution a solution of 8-azido-octanol (2.6 g, 15 mmol, 1 eq.) and Pyridine (1.5 mL, 18 mmol, 1.2 eq.) in dry DCM (37.5 mL, 0.4 M) was slowly added. After stirring for 1.3 hours TLC showed complete disappearance of the starting material and het reaction mixture was further diluted with DCM. The organic layer was washed with cold H_2O, brine, dried over $MgSO_4$, filtrated and concentrated *in vacuo*. Compound **48** (3.6 g, 10 mmol) and K_2CO_3 (1.5 g, 11 mmol, 1.1 eq.) were co-evaporated with toluene (3x), dissolved in dry MeCN (23 mL, 0.4 M) under N_2 and cooled to 0 °C. The crude triflated linker was dissolved in dry MeCN (23 mL) under N_2 and added to the reaction mixture, to which a solution of 2-aminoethyl diphenyl borinate (2-APB, Taylor's catalyst) (0.2 g, 1 mmol, 1 eq.) in dry DCM (3 mL) under N_2 was added. The reaction mixture was allowed to reach room temperature, after stirring for 18 hours TLC showed complete conversion and the reaction was quenched with sat. aq. $NaHCO_3$. The reaction mixture was diluted with EtOAc and the organic layer was washed with H_2O, brine, dried over $MgSO_4$, filtrated and concentrated *in vacuo*. The crude product was then dissolved in dry DMF under N_2 atmosphere and TBAI (0.19 g, 0.5 mmol, 0.05 eq.), BnBr (1.8 mL, 15 mmol, 1.5 eq.) were added. The solution was cooled to 0 °C degrees and NaH (0.6 g, 15 mmol, 1.5 eq., 60 wt.%) was added. The reaction mixture was allowed to reach room temperature. After stirring for 18 hours TLC showed complete conversion and the reaction was quenched with MeOH at 0 °C. The reaction mixture was diluted with Et_2O and the organic layer washed with H_2O. The H_2O layer was then back extracted 2 times. The combined organic layers were washed with brine, dried over $MgSO_4$, filtrated and concentrated *in vacuo*. Column chromatography (5% ->10% Et_2O in pentane) yielded compound **50** (5.2 g, 8.1 mmol) in 81%.^{1}H NMR (500 MHz, $CDCl_3$) δ = 7.54 – 7.26 (m, 15H, H-Aromatic)), 5.63 (s, 1H, H-Benzylidene), 5.53 (d, *J*=1.5, 1H, H-1), 4.79 – 4.71 (m, 2H, CH_2-Bn), 4.33 – 4.26 (m, 1H, H-5), 4.25 – 4.18 (m, 2H, H-4, H-6), 4.08 (dd, *J*=3.2, 1.5, 1H, H-2), 3.88 (t, *J*=10.1, 1H, H-6), 3.80 (dd, *J*=9.8, 3.2, 1H, H-3), 3.69 (dt, *J*=9.3, 6.6, 1H, CH_2-Linker), 3.54 (dt, *J*=9.3, 6.6, 1H, CH_2-Linker), 3.23 (t, *J*=7.0, 2H, CH_2-Linker), 1.57 (s, 4H, CH_2-Linker), 1.40 – 1.26 (m, 8H, CH_2-Linker).^{13}C NMR (126 MHz, $CDCl_3$) δ 137.8, 134.1, 131.7, 129.3, 129.0, 128.6, 128.3, 128.2, 128.0, 127.8, 126.2 (C-Aromatic), 101.6 (C-Benzylidene), 87.4 (C-1), 79.1 (C-4), 78.0 (C-2), 77.0 (C-3), 73.2 (CH_2-Bn), 71.4 (CH_2-Linker), 68.7 (C-6), 65.6 (C-5), 51.6 (CH_2-Linker), 30.1, 29.4, 29.3, 29.0, 26.8, 26.1 (CH_2-Linker). HRMS $[M+Na]^+$ calculated 626.2659, found 626.2659.

2-*O*-benzyl-4,6-*O*-benzylidene-3-*O*-(8-azido-octane)-D-mannose (**51**). Compound **50** (2.2 g, 3.7

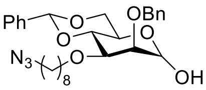

mmol) was co-evaporated with toluene (3x), dissolved in dry DCM (37 mL, 0.1 M) under N_2 and cooled to 0 °C. NIS (0.83 g, 3.7 mmol, 1 eq.) and TFA (0.29 mL, 3.7 mmol, 1 eq.) were added and the reaction was left to stir at 0 °C. After stirring for

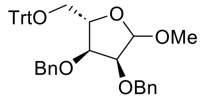

1¼ hours TLC showed complete conversion and the reaction was quenched with Et_3N (0.57 mL, 4.1 mmol, 1.1 eq.). The reaction mixture was then diluted with DCM and washed with sat. aq. $Na_2S_2O_3$, sat. aq. $NaHCO_3$, brine, dried over $MgSO_4$, filtrated and concentrated *in vacuo*. Column chromatography (5% -> 40% Et_2O in pentane) yielded compound **51** (1.7 g, 3.3 mmol) in 90%, α:β, 1:0.9.^{1}H NMR (500 MHz, $CDCl_3$) δ = 7.56 – 7.19 (m, 21H, H-Aromatic α/β), 5.62 (s, 1H, H-Benzylidene α/β), 5.60 (s, 1H, H-Benzylidene α/β), 5.18 (s, 1H, H-1 α), 5.13 (d, *J*=11.4, 1H, CH_2-Bn α/β), 4.83 (d, *J*=12.2, 1H, CH_2-Bn α/β), 4.74 (m, *J*=7.1, 1H, H-1 β), 4.71 (d, *J*=12.3, 1H, CH_2-Bn α/β), 4.66 (d, *J*=11.4, 1H, CH_2-Bn α/β), 4.31 (dd, *J*=10.4, 4.9, 1H, H-6 β), 4.22 (dd, *J*=10.1, 4.7, 1H, H-6 α), 4.15 (t, *J*=9.3, 1H, H-3 β), 4.06 – 3.98 (m, 2H, H-4 β, H-5 α), 3.91 (dd, *J*=3.1, 1.6, 1H, H-3 α), 3.89 – 3.79 (m, 6H, H-2 α, H-2 β, H-6 α, H-6 β, CH_2-Linker), 3.71 (dt, *J*=9.4, 6.5, 1H, CH_2-Linker), 3.65 (dt, *J*=9.3, 6.5, 1H, CH_2-Linker), 3.61 (dd, *J*=9.8, 3.0, 1H, H-4 α), 3.55 (dt, *J*=9.4, 6.6, 1H, CH_2-Linker), 3.35 (ddd, *J*=10.1, 9.2, 5.0, 1H, H-5 β), 3.22 (t, *J*=7.0, 4H, CH_2-Linker), 2.96 (s, 1H, 1-OH α), 1.70 – 1.49 (m, 8H, CH_2-Linker), 1.42 – 1.24 (m, 16H, CH_2-Linker).^{13}C NMR (126 MHz, $CDCl_3$) δ 138.3, 137.9, 137.8, 137.6, 129.0, 128.9, 128.8, 128.6, 128.5, 128.4, 128.3, 128.2, 128.2, 127.9, 126.2, 126.1, 125.6 (C-Aromatic), 101.5 (C-Benzylidene), 101.4 (C-Benzylidene), 94.4 (C-1 α, 94.2 (C-1 β), 80.2 (C4 α), 79.1 (C-3 β), 78.7 (C-4 β/C-5 α), 77.7 (C-3 α), 76.5 (C-2 α/C-2 β), 76.3 (C-2 α/C-2 β), 75.7 (CH_2-Bn), 73.7 (CH_2-Bn), 72.2 (CH_2-Linker), 71.5 (CH_2-Linker), 69.0 (H-6 α), 68.6 (C-6 β), 67.1 (C-5 β), 64.5 (C-4 β/C-5 α), 51.6 (CH_2-Linker), 51.5 (CH_2-Linker), 30.3, 30.1, 29.8, 29.4, 29.4, 29.2, 29.2, 28.9, 26.8, 26.1, 26.1 (CH_2-Linker).HRMS $[M+NH_4]^+$ calculated. 534.2578, found 534.2575.

2-O-benzyl-4,6-O-benzylidene-3-O-(8-azido-octane)-1-O-(trifluoro-N-phenyl-imidate)-D-mannose

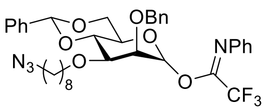

(**52**). Compound **51** (0.26 g, 0.5 mmol) was co-evaporated with toluene (3x) and dissolved in dry DCM (2.5 mL, 0.2 M) under N_2. 2,2,2-Trifluoro-N-phenylacetimidoyl chloride (0.12 mL, 0.75 mL, 1.5 eq.) and $CsCO_3$ (0.24 g, 0.750 mmol, 1.5 eq.) were added. After stirring for 5 hours TLC showed complete conversion, the reaction mixture was filtrated over Celite and concentrated *in vacuo*. Column chromatography (5% -> 20% Et_2O in pentane) yielded compound **52** (0.21 g, 0.313 mmol) in 63%.^{1}H NMR (400 MHz, $CDCl_3$) δ = 7.67 – 6.76 (m, 21H), 5.69 (d, *J*=14.6, 1H), 5.02 (q, *J*=12.0, 2H), 4.85 (t, *J*=10.4, 1H), 4.39 (dt, *J*=9.8, 4.9, 1H), 4.35 – 4.18 (m, 2H), 4.09 – 3.88 (m, 2H), 3.88 – 3.68 (m, 2H), 3.68 – 3.50 (m, 2H), 3.28 (td, *J*=7.0, 2.4, 3H), 1.77 – 1.56 (m, 6H), 1.53 – 1.32 (m, 12H).^{13}C NMR (101 MHz, $CDCl_3$) δ 143.4, 143.3, 137.9, 137.6, 137.5, 137.5, 129.4, 129.0, 128.9, 128.9, 128.7, 128.5, 128.4, 128.2, 128.2, 128.0, 128.0, 127.8, 126.1, 124.6, 119.5, 119.3, 101.5, 96.2, 79.2, 78.3, 78.1, 76.3, 74.7, 74.5, 74.1, 73.6, 71.7, 71.5, 68.5, 68.4, 68.3, 66.8, 51.5, 30.1, 30.1, 29.4, 29.3, 29.2, 28.9, 26.7, 26.0. HRMS $[M+Na]^+$ calculated 705.2871, found 705.2870.

1-*O*-methyl-5-*O*-trityl-L-ribofuranose (**53**). L-Ribose (16.5 g, 100 mmol) was suspended in MeOH (333

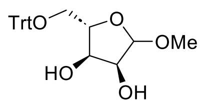

mL, 0.33 M). Subsequently AcCl (0.78 mL, 11 mmol, 0.1 eq.) was added and the reaction was stirred for 3.5 hours. Upon completion the reaction was quenched with solid $NaHCO_3$, filtrated and concentrated *in vacuo*. The crude product was then redissolved in EtOAc:MeOH (550 mL, 4:1, 0.2 M), filtrated again and concentrated *in vacuo*. The product was used without further purification. The crude product was co-evaporated 3 times with toluene and dissolved in dry DMF (550 mL, 0.2 M) under N_2. Subsequently, Et_3N (46 mL, 330 mmol, 3 eq.), DMAP (0.67 g, 5.5 mmol, 0.05 eq.) and Trt-Cl (61.3 g, 220 mmol, 2 eq.) were added. After stirring for 16 hours, the reaction was quenched with MeOH and additional Et_3N (15 mL) was added. The reaction mixture was concentrated *in vacuo*, redissolved in Et_2O, washed with H_2O, brine, dried over $MgSO_4$, filtrated and concentrated *in vacuo*. Column chromatography (10% -> 50% EtOAc in pentane) yielded compound **53** (33.3 g) in 74.5% over 2 steps, α: β, 3:1.^{1}H NMR (300 MHz, $CDCl_3$) δ = 7.54 – 7.37 (m, 9H, H-Aromatic), 7.37 – 7.08 (m, 22H, H-Aromatic), 5.02 (d, *J*=4.5, 0H, H-1 β), 4.86 (d, *J*=1.0, 1H, H-1 α), 4.26 (td, *J*=6.5, 4.8, 1H, H-3 α, H-2 β), 4.14 (td, *J*=3.7, 2.4, 0H, H-4 β), 4.10 – 4.01 (m, 2H, H-2 α, H-4 α), 3.99 – 3.93 (m, 0H, H-3 β), 3.50 (s, 1H, OMe β), 3.39 – 3.35 (m, 0H, H-5 β), 3.33 (s, 3H, OMe α), 3.29 (d, *J*=5.1, 2H, H-5 α), 3.14 (dd, *J*=10.2, 3.8, 0H, H-5 β), 2.88 (d, *J*=9.5, 0H, 2-OH β), 2.56 (d, *J*=3.5, 1H, 2-OH α), 2.53 (d, *J*=8.4, 0H, 3-OH β), 2.28 (d, *J*=6.1, 1H, 3-OH α).^{13}C NMR (75 MHz, $CDCl_3$) δ 144.0, 143.9, 129.2, 128.8, 128.8, 128.4, 128.0, 128.0, 127.2, 127.2, 125.4 (C-Aromatic), 108.2 (C-1 α), 103.1 (C-1 β), 84.6 (C-4 β), 82.1 (C-2 α/C-4 α), 75.4 (C-2 α/C-4 α), 72.8 (C-3 α), 72.1 (C-2 β), 71.8 (C-3 β), 65.1 (C-5 α), 64.0 (C-5 β), 55.8 (OMe β), 55.4 (OMe α). HRMS $[M+Na]^+$ calculated 429.1670, found 429.1673.

1-*O*-methyl-2,3-di-*O*-benzyl-5-*O*-trityl-L-ribofuranose (**54**). Compound **33** was co-evaporated 3 times with toluene, dissolved in dry DMF (447 mL, 0.2 M) under N_2 and cooled to 0 degrees. Benzyl bromide (26.6 mL, 223 mmol, 2.5 eq.), TBAI (3.3 g, 8.9 mmol, 0.1 eq.) and NaH (8.9 g, 223 mmol, 2.5 eq., 60%

wt.) were added. After stirring for 25 hours the reaction was quenched with MeOH and diluted with Et_2O. The organic layer was washed with water and brine. The water layer was re-extracted with Et_2O (2x), the combined organic layers were dried over $MgSO_4$, filtrated and concentrated *in vacuo*. Column chromatography (10% -> 50% Et_2O in pentane) yielded **54** (45.1 g, 77 mmol) in 86%, α: β, 3:1.^{1}H NMR (400 MHz, $CDCl_3$) δ = 7.54 – 7.13 (m, 33H, H-Aromatic), 4.97 – 4.92 (m, 1H, H-1 α/β), 4.71 – 4.60 (m, 2H, CH_2-Bn), 4.58 (d, *J*=5.7, 1H, CH_2-Bn), 4.49 – 4.35 (m, 2H, CH_2-Bn), 4.33 (dt, *J*=7.7, 4.0, 1H, H-4 α), 4.24 (q, *J*=3.7, 0H, H-4 β), 4.16 (dd, *J*=7.3, 4.5, 1H, H-3 α), 3.86 (m, *J*=4.6, 1.1, 1H, H-2 α, H-2 β), 3.78 (dd, *J*=6.6, 2.6, 0H, H-3 β), 3.48 (s, *J*=7.1, 1H, OMe β), 3.35 (s, 3H, OMe α), 3.31 (d, *J*=3.6, 1H, H-5 α), 3.18 (m, 0H, H-5 β), 3.13 (dd, *J*=10.2, 4.6, 1H, H-5 α), 2.97 (dd, *J*=10.1, 3.7, 0H, H-5 β).^{13}C NMR (101 MHz, $CDCl_3$) δ 144.2, 143.9, 138.0, 137.9, 128.9, 128.8, 128.5, 128.5, 128.4, 128.4, 128.2, 128.1, 128.1, 128.0, 127.9, 127.9, 127.8, 127.2, 127.1 (C Aromatic), 106.4 (C-1 α), 102.6 (C-1 β), 82.6 (C-4 α), 80.7 (C-3 α), 79.8 (C-2 α), 78.2, 72.5 (CH_2-Bn), 72.4 (CH_2-Bn), 64.2 (C-5 β), 64.0 (C-5 α), 55.8 (OMe β), 55.4 (OMe α).

1-*O*-methyl-2,3-di-*O*-benzyl-L-ribofuranose (**55**). Compound **54** was dissolved in DCM:MeOH (386 mL, 0.2 M, 1:1) and pTsOH was added until pH 2. After stirring for 18 hours, the reaction was quenched with Et_3N and the mixture was concentrated *in vacuo*. Column chromatography (5% -> 30% EtOAc in pentane) yielded **55** (25.2 g, 73 mmol) in 95%.^{1}H NMR (400 MHz, $CDCl_3$) δ = 7.40 – 7.27 (m, 11H, H-Aromatic), 4.89 (s, 1H, H-1 α/β), 4.76 (d, *J*=12.7, 0H, CH_2-Bn β), 4.69 – 4.60 (m, 2H, CH_2-Bn α/β), 4.59 – 4.47 (m, 2H, CH_2-Bn α/β), 4.28 (dt, *J*=6.8, 3.3, 1H, H-4 α), 4.18 (q, *J*=3.5, 0H, H-4 β), 4.13 (dd, *J*=7.1, 4.7, 1H, H-3 α), 3.86 (d, *J*=4.7, 1H, H-2 α), 3.85 – 3.83 (m, 0H, H-3 β), 3.80 (dd, *J*=12.0, 2.8, 1H, H-5 α), 3.73 (dd, *J*=6.9, 4.2, 0H, H-2 β), 3.66 (dd, *J*=12.0, 3.2, 0H, H-5 β), 3.57 (dd, *J*=12.0, 3.7, 1H, H-5 α), 3.47 (s, 1H, OMe β), 3.41 (dd, *J*=11.9, 3.9, 0H, H-5 β), 3.36 (s, 3H, OMe α).^{13}C NMR (101 MHz, $CDCl_3$) δ 137.8, 128.6, 128.5, 128.3, 128.2, 128.1, 128.0, 127.9 (C aromatic), 107.0 (C1 α), 102.8 (C-1 β), 83.2 (C-4 β), 82.5 (C-4 α), 80.3 (C-2 α), 78.3 (C-2 β), 77.4 (C-3 α), 74.8 (C-3 β), 72.8 (CH_2-Bn), 72.7 (CH_2-Bn), 72.6 (CH_2-Bn), 62.9 (C-5 β), 62.8 (C-5 α), 55.8 (OMe α), 55.7 (OMe β).

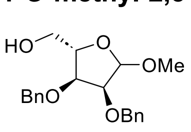

1-*O*-methyl-2,3-di-*O*-benzyl-5-deoxy-5-iodo-L-ribofuranose (**56**). Compound **55** was co-evaporated 3 times with toluene and dissolved in dry THF (292 mL, 0.25 M) under N_2. Subsequently imidazole (9.9 g, 146 mmol, 2 eq.) and PPh_3 (28.7 g, 110 mmol, 1.5 eq.) were added and the mixture was heated to 70 °C. A solution of Iodine (27.8 g, 110 mmol, 1.5 eq.) in THF (73 mL, 1.3 M) was then added. After stirring for 3 hours, the reaction mixture was concentrated *in vacuo* and redissolved in EtOAc. The organic layer was washed with sat. aq. $Na_2S_2O_3$, brine, dried over $MgSO_4$ and concentrated *in vacuo*. Column chromatography (2% -> 50% Et_2O in pentane) yielded **56** (31.2 g, 69 mmol) in 94%.^{1}H NMR (500 MHz, $CDCl_3$) δ 7.38 – 7.22 (m, 10H, H-Aromatic), 4.90 (s, 1H, H-1 α), 4.61 (d, *J* = 12.0 Hz, 1H, CH_2-Bn), 4.54 (d, *J* = 7.9 Hz, 1H, CH_2-Bn), 4.52 (d, *J* = 7.7 Hz, 1H, CH_2-Bn), 4.45 (d, *J* = 11.7 Hz, 1H. CH_2-Bn), 4.13 (dt, *J* = 6.8, 5.4 Hz, 1H, H-4), 3.92 (dd, *J* = 7.0, 4.6 Hz, 1H, H-3), 3.89 – 3.85 (d, 1H, *J* = 4.6 Hz H-2), 3.28 (m, 4H, H-5, OMe), 3.24 (dd, *J* = 10.6, 5.8 Hz, 1H, H-5).^{13}C NMR (126 MHz, $CDCl_3$) δ 137.5, 137.4, 128.3, 128.3, 127.9, 127.8, 127.8 (C aromatic), 106.0 (C-1), 81.5 (C-3), 80.1 (C-4), 79.9 (C-2), 72.4 (CH_2-Bn), 72.2 (CH_2-Bn), 55.2 (OMe), 8.7 (C-5). HRMS $[M+Na]^+$ calculated 477.0533, found 477.0533.

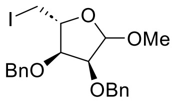

(2S,3S)-2,3-bis(benzyloxy)pent-4-enal (**57**). Zinc dust was activated by adding it portion wise (in 5 min) to a vigorously stirred 3 M HCl solution. The solution was then left to stir for an additional 10 min. The solution was then filtrated, and the Zinc dust was washed with water (2x), dioxane (2x) and Et_2O. The zinc dust was dried *in vacuo*. **56** was then dissolved in THF: H2O (375 mL, 2:1, 0.09 M) and flushed with argon for 15 min while sonicating. The activated Zn dust (33.5 g, 515 mmol, 15 eq.) was then added and the mixture was heated to 60 degrees while sonicating. TLC showed complete conversion after 4 hours. The reaction mixture was filtrated over Celite, concentrated *in vacuo*, redissolved in Et_2O, washed with H_2O, brine, dried over $MgSO_4$, filtrated and concentrated *in vacuo*. Column chromatography (5% -> 10% Et_2O in pentane) yielded **57** (14.4 g, 48.4 mmol) in 70.5%.

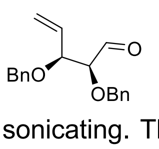

Alkene (**58**). Compound **57** (14.3 g, 48.4 mmol) was split in three portions and suspended in H_2O (3x 80mL, 0.2 M). Subsequently bromocrotonate (3x 7.9 mL, 48 mmol, 3 eq., 84 wt.%), $La(OTf)_3$ (3x 18.9 g, 32.3 mmol, 2 eq.) and Indium (3x 4.3 g, 37 mmol, 2.3 eq.) were added in this order. After 16 hours, additional Indium (3x 1.8 g, 1 eq.) was added. After stirring for an additional 24 hour, TLC showed complete conversion and the reaction mixture was diluted with Et_2O and filtrated over Celite. The Celite was

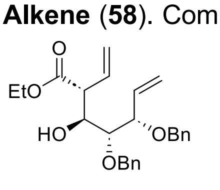

washed with sufficient Et_2O, the combined organic layers were washed with H_2O, brine, dried over $MgSO_4$, filtrated and concentrated *in vacuo*. Column chromatography (5% -> 30% Et_2O in pentane) yielded **58** (14.9 g, 36.3 mmol) in 75%.^{1}H NMR (400 MHz, $CDCl_3$) δ 7.39 – 7.26 (m, 10H. H-Aromatic), 5.89 (ddd, *J* = 17.5, 10.4, 7.3 Hz, 1H, H-1), 5.72 (ddd, *J* = 17.2, 10.2, 9.4 Hz, 1H, H-7), 5.45 – 5.33 (m, 2H, H-9), 5.22 – 5.03 (m, 2H, H-8), 4.72 – 4.61 (m, 2H, CH_2-Bn), 4.45 (d, *J* = 11.3 Hz, 1H, CH_2-Bn), 4.40 (d, *J* = 11.7 Hz, 1H, CH_2-Bn), 4.23 (d, *J* = 9.6 Hz, 1H, H-4), 4.19 – 4.10 (m, 3H, H-2, CH_2 ethyl), 3.44 (dd, *J* = 5.7, 1.3 Hz, 1H, H-3), 3.34 (t, *J* = 9.5 Hz, 1H, H-5), 1.24 (t, *J* = 7.2 Hz, 3H, CH_3).^{13}C NMR (101 MHz, $CDCl_3$) δ 135.6 (C-1/7), 133.1 (C-1/7), 128.6, 128.5, 128.1, 128.0, 128.0, 127.9 (C-Aromatic), 120.0 (C-8), 119.6 (C-9), 80.3 (C-2), 79.1 (C-3), 73.2 (CH_2-Bn), 72.1 (C-4), 71.1 (CH_2-Bn), 60.9 (CH_2-Ethyl), 55.1 (C-5), 14.3 (CH_3). HRMS $[M+H]^+$ calculated 411.2163, found 411.2166.

2,3-di-*O*-benzyl-6-ethanoate-L-mannose-cyclohexene (**58a**). Compound **58** (17.4 g, 42.3 mmol) was co-evaporated with toluene (3x) and dissolved in dry DCM (212 mL, 0.2 M) under N_2. Subsequently, Grubbs 2nd generation catalyst (0.89 g, 1.1 mmol, 0.025 eq.) was added and the mixture was heated to 40 degrees. After stirring for 17h TLC showed complete conversion, and the reaction mixture was concentrated *in vacuo*. Column chromatography (10% -> 50% Et_2O in pentane) yielded compound **58a** (15.8 g, 41 mmol) in 98%.^{1}H NMR (500 MHz, $CDCl_3$) δ 7.38 – 7.24 (m, 10H, H-Aromatic), 5.87 (ddd, *J* = 9.9, 5.1, 2.9 Hz, 1H, H-1), 5.80 (dd, *J* = 9.9, 2.5 Hz, 1H, H-7), 4.76 – 4.59 (m, 4H, CH_2-Bn), 4.56 (t, *J* = 9.4 Hz, 1H, H-4), 4.18 (qd, *J* = 7.1, 1.0 Hz, 2H, CH_2-Ethyl), 4.07 (t, *J* = 4.0 Hz, 1H, H-2), 3.46 (dd, *J* = 10.1, 3.8 Hz, 1H, H-3), 3.13 (dtd, *J* = 8.7, 2.7, 1.0 Hz, 1H, H-5), 3.07 (d, *J* = 2.2 Hz, 1H, 4-OH), 1.26 (d, *J* = 7.1 Hz, 5H, CH_3).^{13}C NMR (126 MHz, $CDCl_3$) δ 171.5 (C=O), 138.6, 138.1, 128.4, 128.3, 127.8, 127.8, 127.7, 127.6, 127.3, 126.6 (C-Aromatic, C-1, C-7), 80.1 (C-3), 72.0 (CH_2-Bn), 71.7 (CH_2-Bn), 69.7 (C-3), 67.1 (C-4), 61.2 (CH_2-Ethyl), 51.1 (C-5), 14.1 (CH_3). HRMS $[M+Na]^+$ calculated 405.1666, found 405.1673.

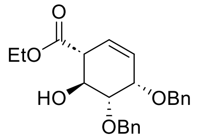

2,3-di-*O*-benzyl-L-mannose-cyclohexene (**59**). Compound **58a** (14.1 g, 36.8 mmol) was coevaporated with toluene (3x) and dissolved in dry THF (368 mL, 0.1 M) under N_2. The mixture was cooled to 0 degrees and DIBAL-H (221mL, 1 M, 221 mmol, 6 eq.) was added slowly. After stirring for 30 min at 0 degrees the mixture was warmed to rt and stirred for an additional 2 hours. After 2 hours the DIBAL-H was quenched with Ethyl acetate (76 mL, 773 mmol, 21 eq.) at 0 degrees and H_2O (86 mL, 130 eq.), $NaBH_4$ (9.1 g, 239 mmol, 6.5 eq.) were added at 0 degrees. After stirring for an additional 16 hour TLC showed full conversion and the reaction mixture was concentrated *in vacuo*. The crude product was then redissolved in ethyl acetate and wash with small amounts of 1 M HCl until gas forming stopped. The organic layer was then dried over $MgSO_4$, filtrated and concentrated *in vacuo*. Column chromatography (30% -> 70% EtOAc in pentane) yielded **59** (10.7 g, 31.3 mmol) in 85%. ^{1}H NMR (400 MHz, $CDCl_3$) δ 7.37 – 7.29 (m, 10H, H-Aromatic), 5.86 (ddd, *J* = 9.9, 5.2, 2.7 Hz, 1H, H-1), 5.64 (dd, *J* = 9.9, 2.3 Hz, 1H, H-7), 4.70 (d, *J* = 11.6 Hz, 1H, CH_2-Bn), 4.65 (s, 2H, CH_2-Bn), 4.52 (d, *J* = 11.7 Hz, 1H, CH_2-Bn), 4.17 – 4.03 (m, 2H, H-2, H-4), 3.80 – 3.68 (m, 2H, H-6), 3.45 (dd, *J* = 10.2, 3.9 Hz, 1H, H-3), 3.01 (s, 2H, 4-OH, 6-OH), 2.37 (dddt, *J* = 9.8, 7.7, 5.3, 2.8 Hz, 1H, H-5).^{13}C NMR (101 MHz, $CDCl_3$) δ 138.6, 137.9, 130.8, 128.6, 128.5, 128.0, 128.0, 128.0, 127.8, 125.9 (C-Aromatic, C-1, C-7), 81.1 (C-3), 71.8 (CH_2-Bn), 71.7 (CH_2-Bn), 69.7 (C-2/C-4), 69.6 (C-2/C-4), 65.4 (C-6), 46.6 (C-5). HRMS $[M+Na]^+$ calculated 363.1564, found 363.1567.

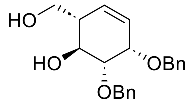

2,3-di-*O*-benzyl-6-*O*-tosyl-L-mannose-cyclohexene (**59a**). Compound **59** (12.7 g, 37 mmol) was dissolved in dry DCM under N_2 and cooled to 0 °C. Et_3N (15.6 mL, 112 mmol, 3 eq.) and TsCl (17.8 g, 93 mmol, 2.5 eq) were added and the reaction mixture was left to warm to room temperature. After stirring for 22 hours, TLC showed complete conversion, and the reaction mixture was quenched with H_2O at 0 °C. The reaction mixture was diluted with DCM and washed with H_2O, sat. aq. $NaHCO_3$, brine, dried over $MgSO_4$, filtrated and concentrated *in vacuo*. Column chromatography (20% -> 50% Et_2O in pentane) yielded **59a** (14.3 g, 28.9 mmol) in 78%.^{1}H NMR (400 MHz, $CDCl_3$) δ = 7.82 – 7.74 (m, 2H, H-Aromatic), 7.38 – 7.26 (m, 12H, H-Aromatic), 5.87 (ddd, *J*=10.0, 5.2, 2.6, 1H, H-1), 5.74 (dd, *J*=10.0, 2.2, 1H, H-7), 4.74 – 4.42 (m, 4H, CH_2-Bn), 4.30 (dd, *J*=9.4, 3.9, 1H, H-6), 4.15 – 4.00 (m, 2H, H-2, H-6), 3.89 (t, *J*=9.7, 1H, H-4), 3.39 (dd, *J*=10.1, 3.9, 1H, H-3), 2.45 (dq, *J*=6.6, 2.1, 1H, H-5), 2.41 (s, 3H, CH_3 Tosyl).^{13}C NMR (101 MHz, $CDCl_3$) δ 144.8, 138.5, 137.8, 132.9, 129.9, 129.6, 128.6, 128.5, 128.0, 128.0, 127.8, 126.3 (C-Aromatic, C-1, C-7), 80.9 (C-3), 71.7 (CH_2-Bn), 71.5 (CH_2-Bn), 70.1 (C-6), 69.3 (C-2), 66.2 (C-4), 44.1 (C-5), 21.7 (CH_3-Tosyl). HRMS $[M+Na]^+$ calculated 517.1654, found 517.1655.

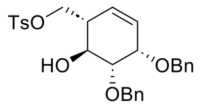

2,3-di-*O*-benzyl-L-rhamnose-cyclohexene (**60**). Compound **59a** (14.3 g, 28.9 mmol) was co-evaporated with toluene (3x). **59a** was then dissolved in dry THF (1.16 L, 0.025 M) under N_2 and cooled

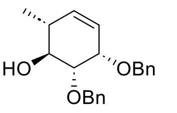

to 0 °C. $LiAlH_4$ (10.8 mL, 4 M, 43.4 mmol) was then added and the reaction was left to reach room temperature. After 24 hours TLC showed complete conversion, and the reaction was cooled to 0 degrees and quenched with brine. The solution was then stirred with sat. aq. sodium tartrate until all aluminum salts dissolved. The solution was then diluted with ethyl acetate and washed with H_2O, sat aq. $NaHCO_3$, brine, dried over $MgSO_4$, filtrated and concentrated *in vacuo*. Column chromatography (10% -> 30% Et_2O in pentane) yielded **60** (7.5 g, 23.2 mmol) in 80%.^{1}H NMR (400 MHz, $CDCl_3$) δ = 7.40 – 7.23 (m, 10H, H-Aromatic), 5.75 (ddd, *J*=9.8, 5.2, 2.7, 1H, H-1), 5.61 (dd, *J*=9.9, 2.2, 1H, H-7), 4.76 – 4.61 (m, 3H, CH_2-Bn), 4.49 (d, *J*=11.7, 1H, CH_2-Bn), 4.07 (ddd, *J*=5.0, 3.8, 0.8, 1H, H-2), 3.78 (dd, *J*=10.2, 8.6, 1H, H-4), 3.39 (dd, *J*=10.2, 3.9, 1H, H-3), 2.25 – 2.11 (m, 1H, H-5), 1.18 (d, *J*=7.1, 3H, H-6).^{13}C NMR (101 MHz, $CDCl_3$) δ 138.7, 138.0, 136.3, 128.6, 128.4, 128.0, 128.0, 127.9, 127.7, 123.2 (C-Aromatic, C-1, C-7), 81.7 (C-3), 71.9 (C-4), 71.6 (CH_2-Bn), 71.5 (CH_2-Bn), 69.7 (C-2), 39.2 (C-5), 18.2 (C-6). HRMS $[M+Na]^+$ calculated 347.1614, found 347.1618.

2,3-di-*O*-benzyl-4-*O*-TIPS-L-rhamnose-cyclohexene (**61**). Compound **60** (3.2 g, 10 mmol) was co-

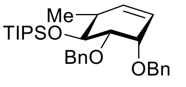

evaporated with toluene (3x) and dissolved in dry DCM (50 mL, 0.2 M) under N_2. The solution was cooled to 0 °C and 2,6 lutidine (2.9 mL, 25 mmol, 2.5 eq.), TIPSOTf (5.4 mL, 20 mmol, 2 eq.) were added. After stirring for 3 hours at 0 °C, TLC showed complete conversion, and the reaction mixture was quenched with sat. aq. $NaHCO_3$. The mixture was diluted with DCM, washed with 1 M HCl, sat. aq. $NaHCO_3$, dried over $MgSO_4$, filtrated and concentrated *in vacuo*. Column chromatography (0% -> 4% Et_2O in pentane) yielded compound **61** (4.7 g, 9.8 mmol) in 98%.^{1}H NMR (500 MHz, $CDCl_3$) δ = 7.41 – 7.19 (m, 12H, H-Aromatic), 5.72 (dqd, *J*=10.1, 2.5, 0.9, 1H, H-1), 5.62 (dddd, *J*=10.2, 3.1, 1.9, 1.0, 1H, H-7), 4.79 – 4.53 (m, 4H, CH_2-Bn), 4.28-4.22 (m, 1H, H-2), 3.98-3.93 (m, 1H, H-4), 3.69 (ddd, *J*=5.8, 3.8, 1.0, 1H, H-3), 2.24-215 (m, 1H, H-5), 1.19 (d, *J*=7.6, 3H, H-6), 0.97 (s, 21H, TIPS).^{13}C NMR (101 MHz, $CDCl_3$) δ 139.0, 132.7, 128.4, 128.3, 128.1, 127.6, 127.6, 127.5, 124.1 (C- Aromatic, C-1, C-7), 77.5 (C-3), 72.7 (C-2/C-4), 72.6 (C-2/C-4), 72.5 (CH_2-Bn), 71.1 (CH_2-Bn), 39.5 (C-5), 19.0 (C-6), 18.2 (TIPS), 18.2 (TIPS), 12.5 (TIPS). HRMS $[M+NH]^+$ calculated 498.3398, found 498.3398.

4-*O*-TIPS-rhamnose-L-cyclohexene (**62**). Compound **61** (0.76 g, 1.6 mmol) was coevaporated with

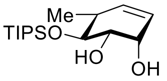

toluene (3x) and dissolved in dry DCM (15.7 mL, 0.1 M) under N_2 and cooled to -20 °C. $TiCl_4$ (3.3 mL, 1M, 2.1 eq.) was added dropwise, after 30 min TLC showed full conversion and the reaction was quenched dropwise with sat. aq. $NaHCO_3$ at -20 °C. The reaction mixture was filtrated, diluted with EtOAc, washed with H_2O, brine, dried over $MgSO_4$, filtrated and concentrated *in vacuo*. Column chromatography (5% -> 20% EtOAc in pentane) yielded compound **62** (0.37 g, 1.2 mmol) in 79%.^{1}H NMR (400 MHz, $CDCl_3$) δ = 5.71 (ddd, *J*=9.9, 4.4, 2.5, 1H, H-1), 5.61 (dd, *J*=10.2, 2.4, 1H, H-7), 4.33 (t, *J*=4.5, 1H, H-2), 3.79 (dd, *J*=8.7, 6.8, 1H, H-4), 3.64 (dt, *J*=8.4, 3.9, 1H, H-3), 2.55 (d, *J*=3.9, 1H, 3-OH), 2.46 (s, 1H, 2-OH), 2.26 – 2.13 (m, 1H, H-5), 1.16 (d, *J*=7.3, 3H, H-6), 1.14 – 1.05 (m, 21H, TIPS).^{13}C NMR (101 MHz, $CDCl_3$) δ 135.5 (C-7), 124.7 (C-1), 75.2 (C-4), 73.9 (C-3), 67.0 (C-2), 39.7 (C-5), 18.9 (C-6), 18.4 (TIPS), 18.4 (TIPS), 13.2 (TIPS). HRMS $[M+Na]^+$ calculated 323.2015 found, 323.2013.

2-*O*-benzoyl-4-*O*-TIPS-L-rhamnose-cyclohexene (**63**). Compound **62** (1.03 g, 3.4 mmol) was co-

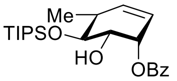

evaporated with toluene (3x) and dissolved in dry MeCN (17.2 mL, 0.2 M) under N_2. DIPEA (3 mL, 17.2 mmol, 5 eq.), BzCl (1.2 mL, 10.3 mmol, 3 eq.) and 2-aminoethyl diphenyl borinate (2-ADB/Taylor's catalyst) (0.077 g, 0.34 mmol, 0.1 eq.) were added. After stirring for 18 hours TLC showed full conversion and the reaction mixture was diluted with Et_2O, the organic layer was washed with 1 M HCl, sat. aq. $NaHCO_3$, brine, dried over $MgSO_4$, filtrated, concentrated *in vacuo*. Column chromatography (2% -> 8% Et_2O in pentane) yielded compound **63** (1.1 g, 2.8 mmol) in 81%.^{1}H NMR (400 MHz, $CDCl_3$) δ = 8.10 – 7.37 (m, 5H, Bz), 5.79 – 5.72 (m, 3H, H-1, H-2, H-7), 3.98 (dd, *J*=8.5, 5.9, 1H, H-4), 3.96 – 3.91 (m, 1H, H-3), 2.35 – 2.25 (m, 1H, H-5), 2.22 (d, *J*=3.9, 1H, 3-OH), 1.23 (d, *J*=7.4, 3H, H-6), 1.16 – 1.05 (m, 21H, TIPS).^{13}C NMR (101 MHz, $CDCl_3$) δ 166.3 (C=O), 137.1 (C-1/C-7), 133.1, 130.4, 129.8, 129.8, 128.4, 128.3 (C-Aromatic), 121.7 (C-1/C-7), 75.4 (C-4), 72.4 (C-3), 70.6 (C-2), 39.9 (C-5), 29.8, 18.9 (C-6), 18.4 (TIPS), 18.4 (TIPS), 13.1 (TIPS). HRMS $[M+Na]^+$ calculated 428.2305, found 238.2308.

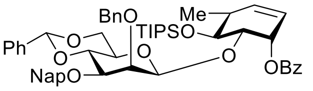

2-O-benzyl-4,6-O-benzylidene-3-O-naphthyl-β-D-mannose-(1->3)-2-O-benzoyl-4-O-TIPS-l-rhamnose-cyclohexene (**64**). Donor **49** (1.1 g, 1.87 mmol, 1.87 eq.), BSP (0.38 g, 1.82 mmol, 1.82 eq.) and TTBP (0.87 g, 3.5 mmol, 3.5 eq.) were co-evaporated with toluene (3x) and dissolved in dry DCM (16 mL, 0.0625 M) under N_2. 3 Å MS were added and the mixture was stirred for an hour, and then cooled to -60 °C. Tf_2O (0.3 mL, 1.82 mmol, 1.82 eq.) was slowly added and the reaction mixture was stirred for 30 min. After 30 min acceptor **63** (0.4 g, 1 mmol, 1 eq.) was added dropwise in a solution of DCM (4 mL, 0.25 M). After stirring for 4.5 hours at -60 °C, TLC showed full conversion, and the reaction was quenched with pyridine at -60 °C. The reaction mixture was then further diluted with DCM, washed with sat. aq. $NaHCO_3$, brine, dried over $MgSO_4$, filtrated and concentrated *in vacuo*. Column chromatography (5% -> 15% Et_2O in pentane) and (5% -> 10%) yielded compound **64** (0.95 g, 1.07 mmol) quantitatively. ^1H NMR (850 MHz, $CDCl_3$) δ = 8.15 – 7.60 (m, 6H, H-Aromatic), 7.55 – 7.12 (m, 16H, H-Aromatic), 5.81 (dt, *J*=10.1, 3.5, 2.3, 1H, H-1), 5.76 – 5.68 (m, 2H, H-2, H-7), 5.44 (s, 1H, H-Benzylidene), 4.91 (d, *J*=11.9, 1H, CH_2-Nap/Bn), 4.86 – 4.74 (m, 2H, CH_2-Nap/Bn), 4.70 (d, *J*=11.8, 1H, CH_2-Nap/Bn), 4.61 (s, 1H, H-1'), 4.16 (dd, *J*=7.0, 3.9, 1H, H-3), 4.09 – 4.03 (m, 3H, H-4, H-4'. H-6'), 3.93 (d, *J*=3.1, 1H, H-2'), 3.58 (dd, *J*=9.9, 3.1, 1H, H-3'), 3.28 (t, *J*=10.4, 1H, H-6'), 3.20 (ddd, *J*=10.0, 9.2, 4.7, 1H, H-5'), 2.37 – 2.27 (m, 1H, H-5), 1.18 (d, *J*=7.5, 3H, H-6), 1.14 – 1.05 (m, 21H, TIPS).^{13}C NMR (214 MHz, $CDCl_3$) δ 166.2, 139.0, 137.7, 135.9 (C-Aromatic), 134.7 (C-7), 133.4, 133.1, 132.7, 131.1, 130.1, 129.0, 128.4, 128.3, 128.3, 128.3, 128.3, 128.2, 128.2, 128.1, 128.1, 128.0, 128.0, 127.8, 127.8, 127.3, 126.4, 126.3, 126.3, 126.2, 126.2, 125.9, 125.7 (C-Aromatic), 122.7 (C-1), 102.1 (C-1'), 101.5 (C-Benzylidene), 78.6 (C-4/C-4'), 77.6 (C-3'), 76.7 (C-2'), 74.9 (C-Bn/Nap), 73.2 (C-4/C-4'), 72.1 (C-Bn/Nap), 70.1 (C-2), 68.4 (C-6'), 67.8 (C-5'), 39.8 (C-5), 19.3 (C-6), 18.3 (TIPS), 18.2 (TIPS), 13.1 (TIPS). HRMS $[M+Na]^+$ calculated 907.4209, found 907.4212.

2-*O*-benzyl-3-*O*-naphthyl-β-D-mannose-(1->3)-2-*O*-benzoyl-4-O-TIPS-L-rhamnose-cyclohexene (**65**). Compound **64** (0.7 g, 0.79 mmol) was suspended in AcOH:H_2O (4:1, 7.9 mL, 0.1 M) and heated to 80 °C. After stirring for 6 hours, TLC showed complete conversion. The reaction mixture was diluted with EtOAc and washed with H_2O, sat. aq. $NaHCO_3$, brine, dried over $MgSO_4$, filtrated and concentrated *in vacuo*. Column chromatography (10% -> 40% EtOAc in pentane) yielded compound **65** (0.57 g, 0.71 mmol) in 90%. ^1H NMR (500 MHz, $CDCl_3$) δ 8.06 – 8.00 (m, 2H, H-Aromatic), 7.91 – 7.69 (m, 4H, H-Aromatic), 7.55 – 7.17 (m, 13H, H-Aromatic), 5.87 (m, 1H, H-2), 5.84 – 5.74 (m, 2H, H-1, H-7), 4.92 (d, *J* = 11.9 Hz, 1H, CH_2-Bn/Nap), 4.74 (d, *J* = 12.0 Hz, 1H, CH_2-Bn/Nap), 4.62 – 4.53 (m, 3H, CH_2-Bn/Nap, H-1'), 4.37 (dd, *J* = 6.6, 4.2 Hz, 1H, H-3), 4.07 (dd, *J* = 6.6, 3.6 Hz, 1H, H-4), 3.92 (d, *J* = 2.9 Hz, 1H, H-2'), 3.73 – 3.67 (m, 2H, H-4', H-6'), 3.35 (dd, *J* = 9.3, 2.9 Hz, 1H, H-3'), 3.23 (ddd, *J* = 9.4, 6.6, 2.7 Hz, 1H, H-5'), 3.13 (dd, *J* = 12.0, 6.7 Hz, 1H, H-6'), 2.49 (s, 1H, OH), 2.42 – 2.33 (m, 1H, H-5), 1.24 (d, *J* = 7.5 Hz, 3H, H-6), 1.20 – 1.08 (m, 24H, TIPS).^{13}C NMR (126 MHz, $CDCl_3$) δ 167.4, 139.0, 135.3 (C-Aromatic), 134.4 (C-1/C-7), 133.3, 133.1, 132.8, 130.9, 129.9, 128.5, 128.2, 128.2, 127.9, 127.8, 127.7, 127.3, 126.5, 126.3, 126.1, 125.6 (C-Aromatic), 122.3 (C-1/C-7), 101.3 (C-1'), 81.7 (C-3'), 76.6 (C-5'), 75.7 (C-3), 74.1 (CH_2-Bn/Nap), 74.1 (C-2'), 72.8 (C-4), 71.2 (CH_2-Bn/Nap), 70.0 (C-2), 67.0 (C-4'), 62.6 (H-6'), 39.3 (C-5), 19.2 (C-6), 18.3 (TIPS), 18.2 (TIPS), 12.9 (TIPS). HRMS $[M+Na]^+$ calculated 819.3896, found 819.3899.

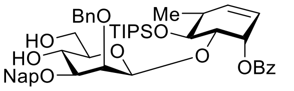

2-*O*-benzyl-3-*O*-naphthyl-β-D-mannose-(1->3)-4-*O*-TIPS-L-rhamnose-cyclohexene (**66**). Compound **65** (0.56 g, 0.7 mmol) was dissolved in MeOH (3.5 mL, 0.2 M) and NaOMe (0.16 mL, 0.7 mmol, 4.37 M) was added. After 26 hours TLC showed full conversion and the reaction mixture was diluted with EtOAc, the organic layer was washed with H_2O, brine, dried over $MgSO_4$, filtrated and concentrated *in vacuo*. Column chromatography (30% -> 70% EtOAc in pentane) yielded compound **66** (0.36 g, 0.52 mmol) in 74%. ^1H NMR (500 MHz, $CDCl_3$) δ 7.89 – 7.76 (m, 4H, H-Aromatic), 7.56 – 7.40 (m, 5H, H-Aromatic), 7.40 – 7.20 (m, 3H, H-Aromatic), 5.71 (dt, J = 10.1, 2.8 Hz, 1H, H-1), 5.62 – 5.52 (m, 1H, H-7), 4.92 – 4.82 (m, 2H, CH2-Bn/Nap), 4.80 (s, 2H, CH2-Bn/Nap), 4.61 (s, 1H, H-1'), 4.54 – 4.47 (m, 1H, H-2), 4.15 – 4.04 (m, 1H, H-4'), 3.95 (m, 2H, H-4, H-2'), 3.89 – 3.55 (m, 6H, H-3, H-6', 3xOH), 3.45 (dd, J = 9.5, 2.8 Hz, 1H, H-3'), 3.28 (dt, J = 9.9, 3.4 Hz, 1H, H-5'), 2.31 – 2.20 (m, 1H, H-5), 1.12 (q, J = 6.8, 6.4 Hz, 26H, H-6, TIPS).^{13}C NMR (126 MHz, $CDCl_3$) δ 138.6, 135.7, 133.4, 133.2 (C-Aromatic), 132.3 (C-7), 128.5, 128.4, 128.1, 128.0, 127.9, 127.7, 126.6, 126.4, 126.3, 126.1, 125.8 (C-Aromatic), 101.3 (C-1'), 81.8 (C-3'), 80.9 (C-3), 76.7 (C-5'), 75.8 (C-4/C-2'), 74.9 (CH2-Bn/Nap), 72.8 (C-4/C-2'), 72.2 (CH2-Bn/Nap), 67.1 (C-4'), 66.3 (C-2), 62.1 (C-6'), 39.4 (C-5), 19.3 (C-6/TIPS), 18.4 (C-6/TIPS), 18.3 (C-6/TIPS), 13.1 (C-6/TIPS). HRMS [M+Na]+ calculated 715.3633, found 715.3637.

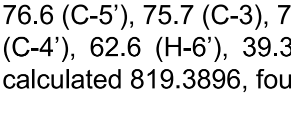

2,4,6-tri-*O*-benzyl-3-*O*-naphthyl-β-D-mannose-(1->3)-2-*O*-benzyl-4-*O*-TIPS-L-rhamnose-cyclohexene (**67**). Compound **66** (0.35 g, 0.51 mmol) was co-evaporated with toluene (3x) and dissolved in dry DMF under N_2. The solution was cooled to 0 °C and BnBr (0.24 mL, 2.04 mmol, 4 eq.), NaH (0.082 g, 2.04 mmol, 4 eq., 60 wt.%) were added. The reaction mixture was allowed to reach room temperature and after stirring for 19 hours, TLC showed full conversion. The reaction mixture was cooled to 0 °C and quenched with MeOH. The mixture was diluted with Et_2O and the organic layer washed with H_2O, brine, dried over $MgSO_4$, filtrated and concentrated *in vacuo*. Column chromatography (5% -> 20% Et_2O in pentane) yielded compound **67** (0.48 g, 0.49 mmol) in 97%.^{1}H NMR (500 MHz, $CDCl_3$) δ 8.04 – 7.84 (m, 4H, H-Aromatic), 7.70 – 7.57 (m, 6H, H-Aromatic), 7.54 – 7.30 (m, 20H, H-Aromatic), 5.87 (ddd, *J* = 9.9, 4.5, 2.5 Hz, 1H, H-1), 5.73 (dd, *J* = 10.0, 2.6 Hz, 1H, H-7), 5.28 – 5.17 (m, 2H, CH_2-Bn/Nap), 5.13 – 5.06 (m, 2H, CH_2-Bn/Nap), 5.02 – 4.88 (m, 3H, CH_2-Bn/Nap), 4.88 – 4.82 (m, 2H, CH_2-Bn/Nap, H-1'), 4.78 (d, *J* = 11.8 Hz, 1H, CH_2-Bn/Nap), 4.62 (d, *J* = 11.8 Hz, 1H, CH_2-Bn/Nap), 4.55 (t, *J* = 4.1 Hz, 1H, H-2), 4.31 – 4.27 (m, 2H, H-4, H-2'), 4.24 (t, *J* = 9.5 Hz, 1H, H-4'), 4.13 (dd, *J* = 8.7, 3.6 Hz, 1H, H-3), 4.01 (dd, *J* = 10.9, 1.9 Hz, 1H, H-6'), 3.96 (dd, *J* = 11.0, 5.8 Hz, 1H, H-6'), 3.83 (dd, *J* = 9.4, 2.9 Hz, 1H, H-3'), 3.70 (ddd, *J* = 9.8, 5.8, 1.9 Hz, 1H, H-5'), 2.49 – 2.38 (m, 1H, H-5), 1.36 – 1.22 (m, 25H, H-6, TIPS).^{13}C NMR (126 MHz, $CDCl_3$) δ 140.1, 139.3, 138.8, 136.0 (C-Aromatic), 133.7 (C-7), 133.6, 133.3, 128.6, 128.5, 128.5, 128.4, 128.3, 128.2, 128.1, 128.0, 127.9, 127.8, 127.7, 127.5, 127.2, 126.6, 126.4, 126.2, 126.0, 125.3 (C-Aromatic), 102.4 (C-1'), 82.8 (C-3'), 76.3 (C-3), 75.5 (C-5'), 75.3 (CH_2-Bn/Nap), 75.0 (C-4'), 74.5 (C-4/C-2'), 74.3 (CH_2-Bn/Nap), 73.7 (C-2), 73.3 (CH_2-Bn/Nap), 72.2 (C-4/C-2'), 71.7 (CH_2-Bn/Nap), 70.2 (CH_2-Bn/Nap), 66.1 (C-6'), 41.0 (C-5), 19.3 9 (C-6/TIPS), 18.6 (C-6/TIPS), 18.5 (C-6/TIPS), 15.6 (C-6/TIPS), 13.7 (C-6/TIPS). HRMS $[M+NH_4]^+$ calculated 980.5498, found 9805491.

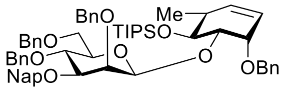

2,4,6-tri-*O*-benzyl-β-D-mannose-(1->3)-2-*O*-benzyl-4-*O*-TIPS-L-rhamnose-cyclohexene (**68**). Compound **67** (0.48 g, 0.49 mmol) was dissolved in DCM, phosphate buffer (4:1, 4.9 mL, 0.1 M, phosphate buffer: 410 mL 1 M NaOH, 250 mL 0.2 M KH_2PO_4, 340 mL H_2O) and DDQ (0.22 g, 0.99 mmol, 2 eq.) were added and the reaction was stirred in the dark. After stirring for 4 hours more DDQ (0.11 g, 1 eq.) was added. After stirring for 1 more hour TLC indicated no further conversion and the reaction mixture was diluted with EtOAc, the organic layer was washed with sat. aq. $NaHCO_3$, sat. aq. $Na_2S_2O_3$, brine, dried over $MgSO_4$, filtrated and concentrated *in vacuo*. Column chromatography (5% -> 20% Et_2O in pentane) yielded compound **68** (0.22 g, 0.27 mmol) in 55%.^{1}H NMR (400 MHz, $CDCl_3$) δ 7.48 – 7.25 (m, 20H, H-Aromatic), 5.76 (ddd, *J* = 10.0, 4.0, 2.3 Hz, 1H, H-1), 5.66 (dd, *J* = 10.2, 2.7 Hz, 1H, H-7), 5.29 (d, *J* = 11.6 Hz, 1H, CH_2-Bn), 5.08 – 4.94 (m, 2H, CH_2-Bn), 4.88 – 4.77 (m, 2H, CH_2-Bn, H-1'), 4.74 – 4.65 (m, 3H, CH_2-Bn), 4.54 (d, *J* = 11.9 Hz, 1H, CH_2-Bn), 4.46 (t, *J* = 3.5 Hz, 1H, H-2), 4.21 (dd, *J* = 7.9, 5.1 Hz, 1H, H-4), 4.14 (dd, *J* = 8.0, 3.6 Hz, 1H, H-3), 4.03 (d, *J* = 3.6 Hz, 1H, H-2'), 3.91 (dd, *J* = 10.9, 1.9 Hz, 1H, H-6'), 3.87 – 3.79 (m, 2H, H-3', H-6'), 3.75 (t, *J* = 9.2 Hz, 1H, H-3'), 3.57 – 3.52 (m, 1H, H-5'), 2.69 – 2.54 (m, 1H, 3-OH'), 2.43 – 2.28 (m, 1H, H-5), 1.33 – 1.17 (m, 26H, H-6. TIPS).^{13}C NMR (101 MHz, $CDCl_3$) δ 139.6, 138.6, 138.5 (C-Aromatic), 133.1 (C-7), 128.5, 128.5, 128.4, 128.2, 128.0, 127.8, 127.8, 127.7, 127.7, 127.5, 127.1 (C-Aromatic), 125.0 (C-1), 101.8 (C-1'), 78.1 (C-2'), 77.1 (C-4'), 75.5 (C-5'), 75.0 (CH_2-Bn), 74.9 (CH_2-Bn), 4.6 (C-3'), 73.7 (C-2), 73.5 (CH_2-Bn), 72.7 (C-4), 71.6 (CH_2-Bn), 69.9 (C-6), 40.4 (C-5), 19.3 (C-6/TIPS), 18.4 (C-6/TIPS), 18.3 (C-6/TIPS), 13.4 (C-6/TIPS). HRMS $[M+NH_4]^+$ calculated 840.4853, found 840.4865.

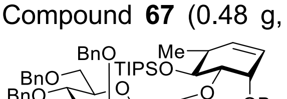

2-*O*-benzyl-4,6-*O*-benzylidene-3-*O*-(8-azido-octane)-β-D-mannose-(1->3)-2,4,6-tri-*O*-benzyl-β-D-mannose-(1->3)-2-*O*-benzyl-4-*O*-TIPS-L-rhamnose-cyclohexene (**69**). Donor **52** (0.11 g, 0.165 mmol, 1.62 eq.) and acceptor **68** (0.084 g, 0.102 mmol, 1 eq.) were co-evaporated with toluene (3x) and dissolved in dry DCM (1 mL, 0.1 M) under N_2 atmosphere. 3 Å MS were added and the mixture was stirred for 1 hour. After stirring for 1 hour, the mixture was cooled to -60 °C and TfOH (0.1 mL, 0.02 M, 0.2 eq.) was added. The reaction mixture was slowly warmed to -40 °C and stirred for 4 hours. Subsequently, the reaction mixture allowed to reach -20 °C and left to stir for an additional 91 hours. The reaction was quenched with Et_3N, diluted with DCM and the organic layer was washed with H_2O, brine, dried over $MgSO_4$, filtrated and evaporated *in vacuo*. Size exclusion chromatography (sephadex, 1:1 DCM:MeOH) and subsequent column chromatography (5% -> 20% Et_2O in pentane) yielded compound **69** (0.036 g, 0.027 mmol) in 27% together with acceptor **68** (0.054 g, 0.066 mmol), yielding **69** in 75% based on recovered starting material. ^{1}H NMR (500 MHz, $CDCl_3$) δ 7.59 – 7.13 (m, 30H, H-Aromatic), 5.67 (ddd, *J* = 10.0, 4.4, 2.4 Hz, 1H, H-1), 5.62 (s, 1H, H-Benzylidene),

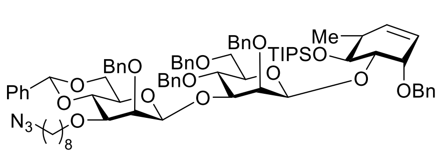

5.57 (dd, *J* = 9.9, 2.6 Hz, 1H, H-7), 5.09 – 5.00 (m, 2H, CH_2-Bn), 4.96 – 4.84 (m, 3H, CH_2-Bn), 4.84 – 4.75 (m, 2H, CH_2-Bn), 4.69 (s, 1H, H-1'), 4.66 – 4.59 (m, 1H, CH_2-Bn), 4.52 – 4.42 (m, 2H, CH_2-Bn), 4.35 (t, *J* = 4.0 Hz, 1H, H-2), 4.31 (s, 1H, H-1"), 4.28 (dd, *J* = 10.5, 4.8 Hz, 1H, H-6"), 4.14 – 4.11 (m, 1H, H-4), 4.11 – 4.07 (m, 1H, H-4"), 4.05 – 4.00 (m, 2H, H-2',H-3'), 3.99 – 3.95 (m, 1H, H-3), 3.92 (t, *J* = 9.1 Hz, 1H, H-4'), 3.86 – 3.76 (m, 3H, H-6', H-6"), 3.66 – 3.63 (m, 1H, H-2"), 3.63 – 3.59 (m, 1H, CH_2-Linker (1H)), 3.53 (ddd, *J* = 9.7, 5.7, 2.0 Hz, 1H, H-5'), 3.45 (dt, *J* = 9.1, 6.4 Hz, 1H, CH_2-Linker (1H)), 3.30 (dd, *J* = 9.9, 3.1 Hz, 1H, H-3"), 3.25 (t, *J* = 7.0 Hz, 2H, CH_2-Linker), 3.23 – 3.17 (m, 1H, H-5"), 2.31 – 2.23 (m, 1H, H-5), 1.63 – 1.55 (m, 4H, CH_2-Linker), 1.41 – 1.28 (m, 16H, CH_2-Linker), 1.22 – 1.12 (m, 27H, TIPS, H-6).^{13}C NMR (126 MHz, $CDCl_3$) δ 139.7, 139.0, 138.7, 138.6, 138.6, 137.7 (C-Aromatic), 133.5 (C-7), 128.9, 128.5, 128.4, 128.3, 128.3, 128.2, 128.2, 128.1, 128.1, 127.7, 127.7, 127.6, 127.5, 127.4, 127.1, 126.4, 126.1 (C-Aromatic), 125.0 (C-1), 102.1 (C-1'), 101.3 (C-Benzylidene), 98.1 (C-1"), 79.2 (C-2'/C-3'/C-3"), 79.1 (C-2'/C-3'/C-3"), 78.6 (C-4"), 76.5 (C-2"), 75.8 (C-5'), 75.1 (CH_2-Bn), 74.8 (CH_2-Bn), 74.2 (CH_2-Bn), 74.0 (C-2, C-4'), 73.5 (CH_2-Bn), 73.4 (C-2'/C-3'), 73.2 (C-4), 72.0 (CH_2-Bn), 71.2 (CH_2-Linker), 70.0 (C-6'), 68.8 (C-6"), 67.9 (C-5"), 51.5 (CH_2-Linker), 40.7 (C-5), 30.1 (CH_2-Linker), 29.8 (CH_2-Linker), 29.4 (CH_2-Linker), 29.2 (CH_2-Linker), 28.9(CH_2-Linker), 26.8 (CH_2-Linker), 26.1 (CH_2-Linker), 19.2 (C-6/TIPS), 18.4 (C-6/TIPS), 18.3 (C-6/TIPS), 13.6 (C-6/TIPS).HRMS $[M+NH_4]^+$ calculated 1333.7449, found 1333.744.

2-*O*-benzyl-4,6-*O*-benzylidene-3-*O*-(8-azido-octane)-β-D-mannose-(1->3)-2,4,6-tri-*O*-benzyl-β-D-mannose-(1->3)-2-*O*-benzyl-4-*O*-TIPS-α-L-rhamnose-cyclophellitol (**70**). Compound **69** (0.086 g, 0.065 mmol) was dissolved in MeCN (0.65 mL, 0.1 M) under argon atmosphere. EDTA (0.0325 mL, 0.013 µmol, 0.4 mM) and trifluoro-acetone (0.087 mL, 0.98 mmol, 15 eq.) were added at 0 °C. Subsequently, a mixture of $NaHCO_3$ (0.038 g, 0.455 mmol) and oxone (0.2 g, 0.325 mmol, 5 eq.) were grinded with pestle and mortar and added in 6 portions over a period of 1 hour. After stirring for 18 hours, additional trifluoro-acetone (0.12 mL, 1.3 mmol, 20 eq.) was added. After stirring for an additional 5 hours, the reaction mixture was diluted with EtOAc. The organic layer was then washed with sat. aq. $NaHCO_3$, brine, dried over $MgSO_4$, filtrated and concentrated *in vacuo*. Column chromatography (10% -> 20% Et_2O in pentane) yielded compound **70** (0.028 g, 0.021 mmol) in 32%, β-epoxide (0.0076 g, 0.0057 mmol) in 8.8% and starting material **65** (0.037 g, 0.028 mmol) in 43%. ^{1}H NMR (500 MHz, $CDCl_3$) δ 7.57 – 7.14 (m, 38H, H-Aromatic), 5.61 (s, 1H, H-Benzylidene), 5.07 – 5.01 (m, 1H, CH_2-Bn), 5.00 – 4.94 (m, 2H, CH_2-Bn), 4.87 – 4.83 (m, 2H, CH_2-Bn), 4.77 – 4.68 (m, 3H, CH_2-Bn), 4.61 (s, 1H, H-1'), 4.57 (d, *J* = 11.9 Hz, 1H, CH_2-Bn), 4.54 (t, *J* = 3.0 Hz, 1H, H-2), 4.46 (d, *J* = 10.3 Hz, 1H, CH_2-Bn), 4.35 (d, *J* = 0.9 Hz, 1H, H-1"), 4.29 (dd, *J* = 10.5, 4.8 Hz, 1H, H-6"), 4.09 (t, *J* = 9.6 Hz, 1H, H-4"), 4.06 (d, *J* = 2.5 Hz, 1H, H-2'), 4.02 – 3.93 (m, 3H, H-4, H-3', H-4'), 3.89 – 3.83 (t, *J* = 3.4 Hz, 2H, H-6'), 3.83 – 3.80 (m, 1H, H-3), 3.79 – 3.74 (m, 1H, H-6''), 3.65 – 3.57 (m, 2H, H-2", CH_2-Linker (1H)), 3.52 – 3.46 (m, 1H, H-5'), 3.43 (dt, *J* = 9.1, 6.4 Hz, 1H, CH_2-Linker (1H)), 3.30 (dd, *J* = 9.9, 3.1 Hz, 1H, H-3"), 3.24 (t, *J* = 7.0 Hz, 2H, CH_2-Linker), 3.22 – 3.18 (m, 2H, H-1, H-5''), 2.90 (d, *J* = 3.8 Hz, 1H, H-7), 2.19 (p, *J* = 7.3 Hz, 1H, H-5), 1.74 – 1.50 (m, 4H, CH_2-Linker (2xCH_2)), 1.41 – 1.21 (m, 11H, H-6, CH_2-Linker (4x CH_2)), 1.23 – 1.09 (m, 21H, TIPS).^{13}C NMR (126 MHz, $CDCl_3$) δ 139.2, 139.0, 138.7, 138.6, 138.6, 137.7, 128.9, 128.6, 128.5, 128.4, 128.3, 128.2, 128.2, 128.1, 128.1, 127.7, 127.7, 127.6, 127.5, 127.5, 127.4, 127.3, 127.3, 127.2, 126.4, 126.1, 125.6 (C-Aromatic), 103.3 (C-1'), 101.3 (C-Benzylidene), 98.2 (C-1"), 81.6 (C-3), 79.3 (C-4/C-3'/C-4', C-3''), 79.2 (C-4/C-3'/C-4', C-3''), 78.6 (C-4"), 76.5 (C-2/C-2"), 76.3 (C-2/C-2"), 75.8 (C-5'), 75.1 (CH_2-Bn), 74.8 (CH_2-Bn), 74.6 (CH_2-Bn), 74.4 (C-2'), 73.9 (CH_2-Bn), 73.9 (C-4/C-3'/C-4'), 73.7 (C-4/C-3'/C-4'), 73.6 (CH_2-Bn), 71.2 (CH_2-Linker), 69.7 (C-6'), 68.8 (C-6''), 68.0 (C-5"), 56.8 (C-7), 54.7 (C-1), 51.5 (CH_2-Linker), 38.8 (C-5), 30.1 (CH_2-Linker), 29.8 (CH_2-Linker), 29.4 (CH_2-Linker), 29.2 (CH_2-Linker), 28.9 (CH_2-Linker), 26.8 (CH_2-Linker), 26.1 (CH_2-Linker), 18.5 (TIPS), 18.5 (TIPS), 17.9 (C-6), 13.8 (TIPS). HRMS $[M+Na]^+$ calculated 1349.740, found 1349.739.

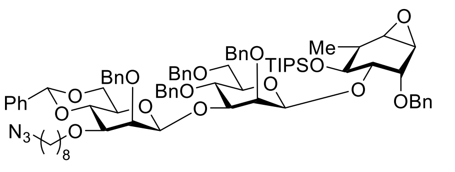

3-*O*-(8-amine-octane)-β-D-mannose-(1->3)-β-D-mannose-(1->3)-α-L-rhamnose-cyclophellitol (**71**). Compound **70** (0.047 g, 0.035 mmol) was dissolved in THF (1.18 mL, 0.03 M) and TBAF (0.071 mL, 0.071 mmol, 1 M, 2 eq.) was added. After stirring for 3.7 hours the reaction mixture was diluted with EtOAc and the organic layer washed with H_2O, sat. aq. $NaHCO_3$, brine, dried over $MgSO_4$, filtrated and concentrated *in vacuo*. The crude product was used without further purification. ^{1}H NMR (500 MHz, $CDCl_3$) δ 7.63 – 7.13 (m, 31H, H-Aromatic), 5.60 (s, 1H, H-Benzylidene), 5.08 – 5.00

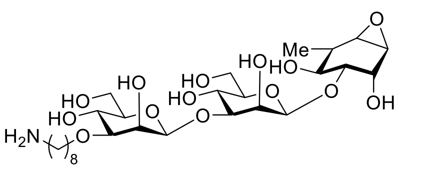

(m, 2H, CH_2-Bn), 4.96 – 4.82 (m, 4H, CH_2-Bn), 4.79 – 4.66 (m, 2H, CH_2-Bn), 4.64 (s, 1H, H-1'), 4.59 (t, *J* = 2.9 Hz, 1H, H-2), 4.56 – 4.42 (m, 2H, CH_2-Bn), 4.40 – 4.36 (m, 1H, H-1"), 4.26 (dd, *J* = 10.4, 4.8 Hz, 1H, H-6"), 4.09 (t, *J* = 9.6 Hz, 1H, H-4"), 4.02 – 3.98 (m, 2H, H-2', H-3'), 3.98 – 3.94 (m, 1H, H-4'), 3.88 – 3.82 (m, 2H, H-6'), 3.77 (d, *J* = 3.1 Hz, 1H, H-2"), 3.75 – 3.69 (m, 2H, H-4, H-6"), 3.67 (d, *J* = 3.0 Hz, 1H, H-3), 3.62 (dt, *J* = 9.1, 5.7 Hz, 1H, CH_2-Linker), 3.52 (ddd, *J* = 9.2, 4.9, 2.7 Hz, 1H, H-5"), 3.47 (dt, *J* = 9.1, 6.5 Hz, 1H, CH_2-Linker), 3.39 – 3.33 (m, 1H, H-3"), 3.27 – 3.21 (m, 3H, H-5", CH_2-Linker), 3.19 (t, *J* = 3.2 Hz, 1H, H-1), 2.88 (d, *J* = 3.5 Hz, 1H, H-7), 2.11 – 2.01 (m, 1H, H-5), 1.65 – 1.54 (m, 4H, CH_2-Linker), 1.41 – 1.25 (m, 11H, H-6, CH_2-Linker).^{13}C NMR (126 MHz, $CDCl_3$) δ 139.1, 138.7, 138.6, 138.5, 137.7, 128.9, 128.6, 128.5, 128.5, 128.4, 128.3, 128.3, 128.3, 128.2, 128.2, 128.1, 127.9, 127.9, 127.8, 127.8, 127.8, 127.7, 127.7, 127.7, 127.6, 127.6, 127.6, 127.5, 127.5, 127.5, 127.2, 126.3, 126.1 (C-Aromatic), 103.5 (C-1'), 101.3 (C-Benzylidene), 98.6 (C-1"), 82.9 (C-3), 79.2 (C-2'/C-3', C-3"), 79.1 (C-2'/C-3'), 78.5 (C-4"), 76.5 (C-2"), 75.4 (C-2/C-5), 75.3 (C-2/C-5), 75.1 (CH_2-Bn), 74.8 (CH_2-Bn) , 74.3 (CH_2-Bn), 74.3 (CH_2-Bn), 73.7 (C-2'/C-3'/C-4'), 73.7 (C-2'/C-3'/C-4'), 73.5 (CH_2-Bn), 71.1 (CH_2-Linker), 70.9 (C-4), 69.8 (C-6'), 68.7 (C-6"), 67.7 (C-5"), 56.5 (C-7), 55.3 (C-1), 51.5 (CH_2-Linker), 36.9 (C-5), 30.1, 29.8, 29.4, 29.2, 28.9, 26.7, 26.1 (CH_2-Linker), 17.1 (C-6). Crude desilylated **70** (0.041 g, 0.035 mmol) was dissolved in THF (0.88 mL, 0.04 M) and purged with N_2. PtO_2 (0.0032 g, 0.014 mmol, 0.4 eq.) was added and the mixture was purged again with N_2. Subsequently, the reaction was purged with H_2 and left to stir under H_2 atmosphere. After 2.5 hours, TLC showed complete conversion and the reaction mixture was purged with N_2, filtrated over a Whatman glassfiber filter and concentrated *in vacuo*. The reduced desilylated compound **70** (0.02 g, 0.0175 mmol) was then coevaporated with toluene (3x) and dissolved in dry THF (0.7 mL, 0.025 M) under N_2, and t-BuOH (0.1 mL, 1.05 mmol, 60 eq.) was added. Ammonia (10 mL) was condensed at -60 °C while maintaining a dry atmosphere and Na (0.024 g, 1.05 mmol, 60 eq.) was added, while using a glass stirring bar. After stirring for 10-20 min the Na was completely dissolved and the reduced compound **70** was added dropwise. After stirring for 1 hour, the reaction mixture was quenched with solid NH_4Cl (0.062 g, 1.16 mmol, 66 eq.) and the ammonia was evaporated at room temperature. Column chromatography over a pre-packed C18 column (0% -> 75% MeCN in water) yielded pure compound **71** (3.8 mg, 6.4 µmol) in 36% over 3 steps.^{1}H NMR (500 MHz, D_2O) δ 4.70 (s, D_2O, H-1', H-2") 4.57 (t, *J* = 3.0 Hz, 1H, H-2), 4.21 – 4.18 (m, 1H, H-2'), 4.18 – 4.15 (m, 1H, H-2"), 3.85 – 3.80 (m, 3H, H-3', H-6'), 3.70 – 3.66 (m, 1H, H-6"), 3.66 – 3.61 (m, 3H, CH_2-Linker, H-6", H-4'/H-5'), 3.61 – 3.58 (m, 1H, H-3), 3.50 (d, *J* = 9.7 Hz, 1H, H-4"), 3.44 (dt, *J* = 9.7, 6.8 Hz, 1H, CH_2-Linker), 3.40 – 3.36 (m, 1H, H-4), 3.36 – 3.34 (m, 1H, H-3"), 3.33 – 3.27 (m, 3H, H-1, H-5", H-4'/H-5'), 3.00 (dd, *J* = 3.7, 0.8 Hz, 1H, H-7), 2.89 (q, *J* = 7.6, 6.7 Hz, 2H, CH_2-Linker), 1.95 – 1.89 (m, 1H, H-5), 1.53 (ddd, *J* = 19.6, 13.9, 7.0 Hz, 4H, CH_2-Linker), 1.31 – 1.21 (m, 8H, CH_2-Linker), 1.14 (d, *J* = 7.3 Hz, 3H, H-6).^{13}C NMR (126 MHz, D_2O) δ 101.2 (C-1'/C-2'), 96.9 (C-1'/C-2'), 80.7 (C-4/C-3"), 79.8 (C-3), 79.0 (C-3'), 76.4 (C-5", C-4'/C-5'), 75.9 (C-5", C-4'/C-5'), 70.5 (C-4/C-3"), 69.6 (CH_2-Linker), 67.7 (C-2'), 67.6 (C-2/C-2''), 65.9 (C-4''), 65.1 (C-4'/C-5'), 61.1 (C-6'/C-6''), 60.9 (C-6'/C-6''), 57.3 (C-7), 56.0 (C-1), 39.6 (CH_2-Linker), 36.8 (C-5), 28.8, 28.3, 28.1, 26.7, 25.5, 25.0 (CH_2-Linker), 15.9 (C-6). HRMS [M+Na]$^+$ calculated 612.3221, found 612.3226.

3-*O*-(8-amide Cy5-octane)-β-D-mannose-(1->3)-β-D-mannose-(1->3)-α-L-rhamnose-cyclophellitol

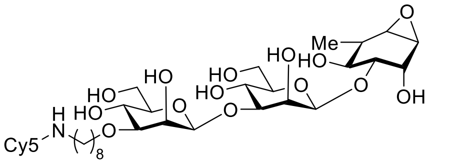

(**3**). A pre-activated Cy5 acid solution was prepared by: freeze drying Cy5 (6.4 mg, 0.013 mmol, 1 eq.), subsequently dissolving it in dry DMF (0.1 mL, 0.132 M) and adding DIPEA (0.1 mL, 0.066 mmol, 0.66 M in dry DMF, 5 eq.), pentafluoro-phenyl-2,2,2-trifluoroacetate (0.1 mL, 0.033 mmol, 0.33 M in dry DMF, 2.5 eq.). After stirring for 2 hours, LC-MS indicated full conversion to the activated carboxylic acid and the reaction was quenched with H_2O (0.1 mL, 0.066 mmol, 0.66 M in dry DMF, 5 eq.) and additional DIPEA (0.1 mL, 0.066 mmol, 0.66 M in dry DMF, 5 eq.). Subsequently 0.05 mL of dry DMF was added to a final concentration of 0.022 M activated Cy5. Of this pre-activated mixture 0.2 mL (0.0044 mmol activated Cy5, 1.5 eq.) was added to compound **71** (1.8 mg, 2.94 µmol, 1 eq.). After 3 hours LC-MS showed full conversion, and the reaction mixture was diluted with H_2O and freeze dried. HPLC purification (MeCN/H_2O/AcOH) yielded compound **3** (2.58 mg, 2.4 µmol) in 81%.^{1}H NMR (600 MHz, D_2O) δ 7.89 (t, *J* = 13.1 Hz, 3H), 7.45 (d, *J* = 7.3 Hz, 3H), 7.37 – 7.28 (m, 3H), 7.28 – 7.05 (m, 5H), 6.44 (t, *J* = 12.4 Hz, 1H), 6.14 (dd, *J* = 13.7, 6.4 Hz, 2H, H-Aromatic Cy5), 4.77 (s, 1H, H-1'), 4.72 (s, 1H, H-1"), 4.64 (t, *J* = 3.1 Hz, 1H, H-2), 4.26 (d, *J* = 3.3 Hz, 1H, H-2'), 4.12 (d, *J* = 3.1 Hz, 1H, H-2"), 4.06 (t, *J* = 6.9 Hz, 2H, Cy5 linker), 3.91 – 3.85 (m, 3H, H-3', H-6', H-6"), 3.74 (dd, *J* = 12.4, 5.8 Hz, 1H, H-6'), 3.72 – 3.65 (m, 3H, H-3, H-4', H-6"), 3.56 – 3.49 (m, 6H, H-4", CH_2-Linker, CH_3 Cy5), 3.44 (dd, *J* = 10.5, 8.9 Hz, 1H, H-4), 3.38 (t, *J* = 3.3 Hz, 1H, H-1), 3.36 (ddd, *J* = 9.8, 5.7, 2.3 Hz, 1H, H-5'),

3.34 – 3.29 (m, 2H, H-5”, CH_2-Linker), 3.26 (dd, *J* = 9.6, 3.1 Hz, 1H, H-3”), 3.06 (d, *J* = 3.6 Hz, 1H, H-7), 2.97 (t, *J* = 7.2 Hz, 2H, CH_2-Linker), 2.15 (t, *J* = 6.8 Hz, 2H, Cy5-Linker), 2.03 – 1.96 (m, 1H, H-5), 1.87 (s, 12H, Acetic acid), 1.80 (t, *J* = 7.9 Hz, 2H, Cy5 Linker), 1.62 – 1.50 (m, 18H, 4x CH_3, Cy5 linker), 1.43 (t, *J* = 7.1 Hz, 2H, CH_2-Linker), 1.34 – 1.23 (m, 6H, CH_2-Linker, Cy5 Linker), 1.20 (d, *J* = 7.4 Hz, 3H, H-6), 1.17 – 1.10 (m, 10H, CH_2-Linker, Cy5 linker). ^{13}C NMR (151 MHz, D_2O) δ 182.5, 177.1, 174.6, 174.4, 154.1, 143.8, 143.0, 142.2, 142.1, 129.4, 129.4, 125.9, 125.9, 125.2, 123.3, 123.1, 112.0, 111.6, 104.0, 102.1 (C-1’), 97.7 (C-1”), 81.6 (C-3”), 80.6 (C-3), 79.8 (C-3’), 77.3 (C-5”), 76.8 (C-5’), 71.4 (C-4), 70.4 (CH_2-Linker), 68.6 (C-2’), 68.4 (C-2/C-2”), 68.4 (C-2/C-2”), 66.7 (C-4’), 66.0 (C-4”), 62.0 (C-6’/C-6”), 61.8 (C-6’/C-6”), 58.1 (C-7), 56.9 (C-1), 50.0 (Quaternary, Cy5), 49.8 (Quaternary, Cy5), 44.5 (Cy5-Linker), 40.3 (CH_2-Linker), 37.7 (C-5), 31.6 (Cy5-Linker), 29.7 (CH_3 on N Cy5), 29.5, 29.3, 29.2, 27.9, 27.7, 27.7, 27.1, 26.3, 26.0, 25.9, 24.2 (CH_2-Linker/Cy5 Linker), 16.8 (C-6). HRMS $[M]^+$ calculated 1076.605, found 1076.605.

Synthesis of probe 4

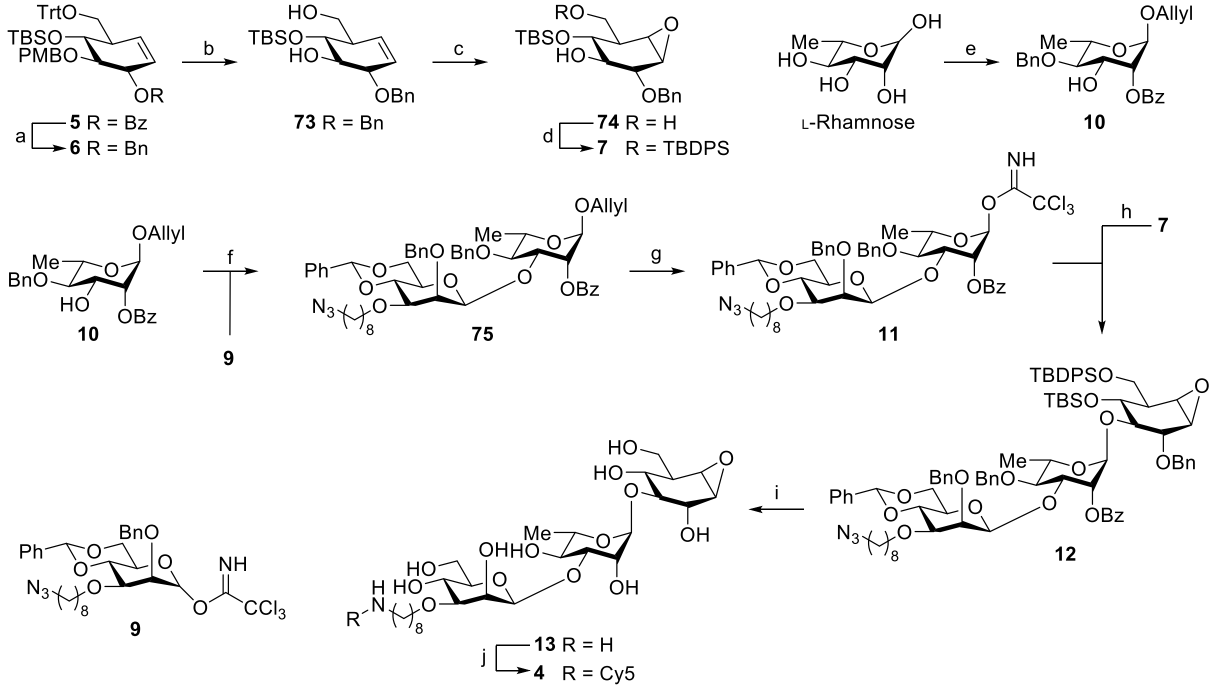

Scheme 4. a) i) NaOMe, MeOH; ii) BnBr, NaH, DMF, 98%; b) TFA, TES-H, DCM, 63%; c) mCPBA, $NaHCO_3$, DCM, 89%; d) TBDPS-Cl, imidazole, DMF, quant; e) i) TfOH, allyl alcohol, reflux; ii) $PhC(OCH_3)_3$, pTsOH, MeCN; iii) BnBr, NaH; 80% AcOH, 53%; f) TMSOTf, DCM, 3 Å MS, -80 °C -> -60 °C, 65%, α:β 1:3.4; g) i) $\{Ir(COD)[PCH_3(C_6H_5)_2]_2\}PF_6$, H_2, THF; ii) NIS, $NaHCO_3$, H_2O, 78%; iii) trichloroacetonitrile, DBU, DCM, 73%; h) TMSOTf, -40 °C, 63%; i) i) TBAF, THF ii) Pt_2O, H_2; iii) NH_3, Na, t-BuOH, -60 °C, 51% over 3 steps; j)Cy5-CO_2PFP, DMF, DIPEA, H_2O, 33%.

1-*O*-allyl-2-*O*-benzoyl-4-*O*-benzyl-α-L-rhamnose (10). L-Rhamnose (3.3 g, 20 mmol) was suspended in allyl alcohol (30 mL, 0.625 M) and TfOH (0.3 mL, 3.4 mmol, 0.17 eq.) was added. The suspension was heated to reflux, after 3 hours TLC indicated complete conversion and the reaction mixture was cooled to room temperature and quenched with Et_3N (0.56 mL, 4 mmol, 0.2 eq.). The crude reaction mixture was concentrated *in vacuo* and co-evaporated 3 times with toluene. The crude product was then dissolved in dry MeCN (200 mL, 0.1 M) under N_2 and trimethyl orthobenzoate (5.2 mL, 30 mmol, 1.5 eq.), pTsOH (0.5 g, 2.6 mmol, 0.13 eq. until pH3 is reached) were added. After 1 hour TLC indicated complete conversion and the reaction was quenched with Et_3N (0.5 mL, 3.6 mmol, 0.18 eq.). NaH (1.2 g, 30 mmol, 1.5 eq., 60 wt.%) and BnBr (2.9 mL, 24 mmol, 1.2 eq.) were then added at 0 degrees and the reaction mixture was left to reach room temperature. After stirring for 1¼ hour additional NaH (1.6 g, 40 mmol, 2 eq., 60 wt.%) and BnBr (4.8 mL, 40 mmol, 2 eq.) were added at 0 °C. After stirring for an additional 16 hours TLC indicated complete conversion and the reaction mixture was quenched with H_2O at 0 degrees. Then 80% AcOH (200 mL) was added slowly at 0 °C and the reaction was left to reach room temperature. After 5 hours TLC indicated complete conversion, and the reaction mixture was diluted with DCM. The organic layer was washed with H_2O, sat. aq. $NaHCO_3$ (2x), sat. aq. K_2CO_3, brine, dried over $MgSO_4$, filtrated and concentrated *in vacuo*. Column chromatography (2% -> 15% EtOAc in pentane) yielded

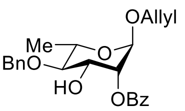

compound **10** (4.2 g, 10.5 mmol) in 53% over 4 steps. ^{1}H NMR (400 MHz, $CDCl_3$) δ 8.15 – 8.02 (m, 2H, H-Aromatic), 7.68 – 7.29 (m, 8H, H-Aromatic), 5.93 (dddd, *J* = 17.3, 10.3, 6.0, 5.1 Hz, 1H, CH-Allyl), 5.41 (dd, *J* = 3.5, 1.7 Hz, 1H, H-2), 5.34 (dq, *J* = 17.2, 1.6 Hz, 1H, CH_2-Allyl), 5.24 (dt, *J* = 10.4, 1.4 Hz, 1H, CH_2-Allyl), 4.94 (d, *J* = 1.7 Hz, 1H, H-1), 4.90 (d, *J* = 11.1 Hz, 1H, CH_2-Bn), 4.79 (d, *J* = 11.1 Hz, 1H, CH_2-Bn), 4.30 (dd, *J* = 9.4, 3.5 Hz, 1H, H-3), 4.22 (ddt, *J* = 12.9, 5.2, 1.6 Hz, 1H, CH_2-Allyl), 4.03 (ddt, *J* = 12.9, 6.0, 1.4 Hz, 1H, CH_2-Allyl), 3.88 (dq, *J* = 9.4, 6.2 Hz, 1H, H-5), 3.52 (t, *J* = 9.4 Hz, 1H, H-4), 1.43 (d, *J* = 6.2 Hz, 3H, H-6). HRMS $[M+Na]^+$ calculated 421.1618, found 421.1622.

2-*O*-benzyl-4,6-*O*-benzylidene-3-*O*-(8-azido-octane)-1-*O*-trichloroimidate-α-D-mannose (**9**). Compound **51** (1.02 g, 2 mmol) was co-evaporated with toluene (3x), dissolved in dry DCM (10 mL, 0.2 M) under N_2 and cooled to 0 °C. 2,2,2-Trichloroacetonitrile (0.8 mL, 8 mmol, 4 eq.) and DBU (0.06 mL, 0.4 mmol, 0.2 eq.) were added and the reaction was left to stir at 0 °C. After stirring for 4 hours TLC indicated complete conversion and the reaction mixture was concentrated *in vacuo*. Column chromatography (5% -> 20% Et_2O in pentane) yielded compound **9** (1.4 g, 2.2 mmol) quantitatively. ^{1}H NMR (500 MHz, $CDCl_3$) δ 8.63 (s, 1H, NH), 7.53 – 7.28 (m, 10H, H-Aromatic), 6.30 (d, *J* = 1.7 Hz, 1H, H-1), 5.64 (s, 1H, H-Benzylidene), 4.86 (d, *J* = 12.1 Hz, 1H, CH_2-Bn), 4.80 (d, *J* = 12.1 Hz, 1H, CH_2-Bn), 4.31 (dd, *J* = 10.2, 4.8 Hz, 1H, H-6), 4.25 (t, *J* = 9.8 Hz, 1H, H-4), 4.03 – 3.95 (m, 2H, H-2, H-5), 3.90 – 3.83 (m, 2H, H-3, H-6), 3.71 (dt, *J* = 9.3, 6.6 Hz, 1H, CH_2-Linker), 3.54 (dt, *J* = 9.2, 6.5 Hz, 1H, CH_2-Linker), 3.23 (t, *J* = 7.0 Hz, 2H, CH_2-Linker), 1.63 – 1.54 (m, 4H, CH_2-Linker), 1.40 – 1.25 (m, 8H, CH_2-Linker).^{13}C NMR (126 MHz, $CDCl_3$) δ 160.6, 129.0, 128.5, 128.3, 128.2, 128.0, 126.1 (C-Aromatic), 101.5 (C-Benzylidene), 96.8 (C-1), 78.4 (C-4), 76.5 (C-3), 75.0 (C-2/C-5), 73.8 (CH_2-Bn), 71.6 (CH_2-Linker), 68.6 (C-6), 67.0 (C-2/C-5), 51.6 (CH_2-Linker), 30.1, 29.4, 29.3, 29.0, 26.8, 26.1 (CH_2-Linker).

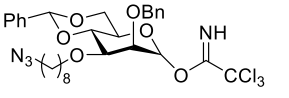

2-*O*-benzyl-4,6-*O*-benzylidene-3-*O*-(8-azido-octane)-β-D-mannose-(1->3)-1-*O*-allyl-2-*O*-benzoyl-4-*O*-benzyl-α-L-rhamnose (**75**). Donor **9** (0.64 g, 0.98 mmol, 1 eq.) and acceptor **10** (0.62 g, 1.6 mmol, 1.6 eq.) were coevaporated with toluene (3x), dissolved in dry DCM (19.5 mL, 0.05 M), 3 Å MS were added and the mixture was stirred for 1 hour under N_2. The reaction mixture was then cooled to -80 °C and TMSOTf (0.035 mL, 0.2 eq.) was added. The reaction mixture was then allowed to reach -60 degrees. After stirring for 4 hours, TLC indicated complete conversion, and the reaction was quenched with Et_3N. The mixture was diluted with DCM, washed with sat. aq. $NaHCO_3$, H_2O, brine, dried over $MgSO_4$, filtrated and concentrated *in vacuo*. Column chromatography (10% -> 30% Et_2O in pentane) yielded compound **75** (0.4 g, 0.46 mmol) in 65%, α:β 1:3.4. ^{1}H NMR (500 MHz, $CDCl_3$) δ 8.18 – 6.95 (m, 38H, H-Aromatic), 5.96 (ddd, *J* = 22.5, 10.9, 5.6 Hz, 1H, CH-Allyl), 5.53 (s, 1H, H-Benzylidene), 5.41 (dd, *J* = 3.5, 1.7 Hz, 1H, H-2), 5.34 (d, *J* = 19.4 Hz, 1H, CH_2-Allyl), 5.25 (dd, *J* = 10.3, 1.6 Hz, 1H, CH_2-Allyl), 4.99 (d, *J* = 1.7 Hz, 1H, H-1), 4.79 – 4.73 (m, 2H, CH_2-Bn), 4.71 (s, 1H, H-1'), 4.67 (d, *J* = 12.4 Hz, 1H, CH_2-Bn), 4.57 (d, *J* = 11.5 Hz, 1H, CH_2-Bn), 4.31 (dd, *J* = 10.5, 4.9 Hz, 1H, H-6'), 4.24 (dd, *J* = 9.6, 3.3 Hz, 1H, H-3), 4.23 – 4.17 (m, 1H, CH_2-Allyl), 4.10 – 4.04 (m, 1H, CH_2-Allyl), 4.00 (t, *J* = 9.6 Hz, 1H, H-4), 3.88 (dd, *J* = 9.6, 6.2 Hz, 1H, H-5), 3.83 (t, *J* = 10.3 Hz, 1H, H-6'), 3.69 – 3.63 (m, 2H, H-2', H-4), 3.42 (dt, *J* = 9.3, 6.6 Hz, 1H, CH_2-Linker), 3.34 – 3.26 (m, 2H, H-5', CH_2-Linker), 3.24 (t, *J* = 7.0 Hz, 2H, Ch_2-Linker), 3.18 (dd, *J* = 9.9, 3.0 Hz, 1H, H-3'), 1.57 – 1.49 (m, 4H, CH_2-Linker), 1.41 (d, *J* = 6.2 Hz, 3H, H-6), 1.29 (t, *J* = 13.0 Hz, 8H, CH_2-Linker). HRMS $[M+Na]^+$ calculated 914.4193, found 914.4198.

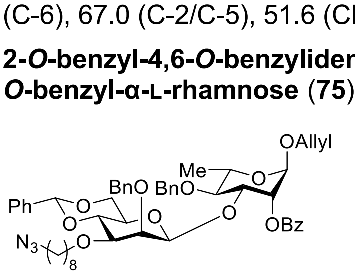

2-*O*-benzyl-4,6-*O*-benzylidene-3-*O*-(8-azido-octane)-β-D-mannose-(1->3)-2-*O*-benzoyl-4-*O*-benzyl-L-rhamnose (**75a**). Compound **75** (0.4 g, 0.46 mmol) was dissolved in freshly distilled THF (6 mL, 0.075 M) and degassed using argon. $\{Ir(COD)[PCH_3(C_6H_5)_2]_2\}PF_6$ (0.019 g, 0.023 mmol, 0.05 eq.) was added and the mixture was shortly purged with Argon again. The reaction mixture was then purged with H2 for 5-25 seconds or until the color changes from reddish to orange/colorless. The reaction mixture is then degassed with Argon again. After 2.5 hours TLC showed full conversion and additional THF (3 mL, 0.05 M), H_2O (1 mL), NIS (0.16 g, 0.69 mmol, 1.5 eq.) and $NaHCO_3$ (0.058 g, 0.69 mmol, 1.5 eq.) were added. After stirring for an additional 2 hours TLC showed full conversion and the reaction mixture was diluted with EtOAc, washed with sat. aq. $Na_2S_2O_3$, brine, dried over $MgSO_4$ and concentrated *in vacuo*. Column chromatography (20% -> 40% EtOAc in pentane) yielded compound **75a** (0.3 g, 0.36 mmol) in 78%. See compound **82** for an updated protocol.^{1}H NMR (500 MHz, $CDCl_3$) δ 8.25 – 8.06 (m, 2H, H-Aromatic), 7.66 – 7.16 (m, 21H, H-Aromatic), 5.53 (s, 1H, H-Benzylidene), 5.43 (dd, *J* = 3.4, 1.8 Hz, 1H, H-2), 5.38 (dd, *J* = 3.8, 1.8 Hz, 1H, H-1), 4.77 (dd, *J* = 16.2, 12.0 Hz, 2H, CH_2-Bn), 4.72 (d, *J* = 0.8 Hz, 1H, H-1'), 4.67 (d, *J* = 12.4 Hz, 1H, CH_2-Bn), 4.58 (d, *J* = 11.6 Hz, 1H, CH_2-Bn), 4.36 – 4.27 (m, 2H, H-3, H-6'), 4.14 – 4.05 (m, 1H, H-5), 4.01 (t, *J* = 9.6 Hz, 1H, H4'), 3.84 (t, *J* = 10.3 Hz, 1H, H-6'), 3.71 – 3.61 (m, 2H, H-4, H-2'), 3.42

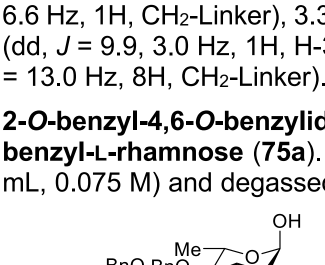

(dt, J = 9.2, 6.6 Hz, 1H, CH_2-linker), 3.33 – 3.26 (m, 2H, CH_2-Linker, H-5'), 3.24 (t, J = 7.0 Hz, 2H, CH_2-Linker), 3.17 (dd, J = 9.9, 3.0 Hz, 1H, H-3'), 2.93 (d, J = 3.9 Hz, 1H, 1-OH), 1.61 – 1.45 (m, 4H, CH_2-Linker), 1.40 (d, J = 6.2 Hz, 3H, H-6), 1.36 – 1.19 (m, 8H, CH_2-Linker).^{13}C NMR (126 MHz, $CDCl_3$) δ 165.9, 138.7, 138.4, 137.8, 133.3, 130.3, 130.3, 129.9, 128.9, 128.7, 128.6, 128.5, 128.2, 128.2, 128.1, 128.1, 128.1, 128.0, 127.9, 127.4, 127.3, 127.1, 126.2, 126.1 (C-Aromatic), 103.9 (C-1'), 101.3 (C-Benzylidene), 92.0 (C-1), 81.8 (C-4), 79.2 (C-3'), 78.2 (C-4'), 77.4 (C-3), 75.5 (C-2'), 75.4 (CH_2-Bn), 74.2 (CH_2-Bn), 74.1 (C-2), 70.9 (CH_2-Linker), 68.6 (C-6'), 68.0 (C-5/C-5'), 67.9 (C-5/C-5'), 51.6 (CH_2-Linker), 30.0, 29.5, 29.3, 29.0, 26.8, 26.1 (CH_2-Linker), 18.4 (C-6). HRMS [M+Na]$^+$ calculated 874.3884, found 874.3685.

2-*O*-benzyl-4,6-*O*-benzylidene-3-*O*-(8-azido-octane)-β-D-mannose-(1->3)-2-*O*-benzoyl-4-*O*-benzyl-1-*O*-trichloroimidate-α-L-rhamnose (**11**). Compound **75a** (0.29 g, 0.34 mmol) was co-evaporated with toluene (3x), dissolved in dry DCM (1.7 mL, 0.2 M) under N_2 and cooled to 0 °C. 2,2,2-Trichloroacetonitrile (0.14 mL, 1.4 mmol, 4 eq.) and DBU (0.069 mmol, 0.2 eq.) were added and the reaction was left to stir at 0 °C. After stirring for 18 hours TLC indicated complete conversion and the reaction mixture was concentrated *in vacuo*. Column chromatography (10% -> 40% Et_2O in pentane) yielded compound **11** (0.25 g, 0.25 mmol) in 73%.^{1}H NMR (500 MHz, $CDCl_3$) δ 8.73 (s, 1H, NH), 8.18 – 8.05 (m, 2H, H-Aromatic), 7.62 – 7.17 (m, 20H, H-Aromatic), 6.42 (d, J = 2.0 Hz, 1H, H-1), 5.58 (dd, J = 3.3, 2.0 Hz, 1H, H-2), 5.51 (s, 1H, H-Benzylidene), 4.82 – 4.72 (m, 2H, CH_2-Bn), 4.72 (d, J = 2.3 Hz, 2H, H-1' CH_2-Bn), 4.63 (m, 2H, CH_2-Bn), 4.31 (dd, J = 9.7, 3.3 Hz, 1H, H-3), 4.17 (dd, J = 10.4, 4.8 Hz, 1H, H-6'), 4.05 (dt, J = 6.1, 3.1 Hz, 1H, H-5), 4.03 – 3.96 (m, 1H, H-4'), 3.83 (t, J = 10.3 Hz, 1H, H-6'), 3.77 (t, J = 9.6 Hz, 1H, H-4), 3.68 (d, J = 3.0 Hz, 1H, H-2'), 3.51 – 3.46 (m, 1H, CH_2-Linker), 3.36 (dt, J = 9.3, 6.5 Hz, 1H, CH_2-Linker), 3.27 (td, J = 9.7, 4.9 Hz, 1H, H-5'), 3.23 (dd, J = 9.9, 3.1 Hz, 1H, H-3'), 3.19 (t, J = 7.0 Hz, 2H, CH_2-Linker), 1.59 – 1.47 (m, 4H, CH_2-Linker), 1.43 (d, J = 6.3 Hz, 3H, H-6), 1.37 – 1.23 (m, 8H, CH_2-Linker).^{13}C NMR (126 MHz, $CDCl_3$) δ 165.3, 159.9, 138.5, 137.9, 137.6, 133.4, 129.8, 129.7, 128.8, 128.5, 128.5, 128.1, 128.0, 127.9, 127.9, 127.5, 127.3, 126.0 (C-Aromatic), 103.8 (C-1'), 101.2 (C-Benzylidene), 94.6 (C-1), 91.0 (Quaternary C), 80.7 (C-4), 79.0 (C-3'), 78.1 (C-4'), 77.2 (C-3), 75.6 (CH_2-Bn), 75.3 (C-2'), 74.1 (CH_2-Bn), 72.2 (C-2), 70.9 (C-5), 70.8 (CH_2-Linker), 68.4 (C-6'), 67.8 (C-5'), 65.8, 51.4 (CH_2-Linker), 29.9, 29.3, 29.1, 28.8, 26.6, 26.0 (CH_2-Linker), 18.2 (C-6). HRMS [M+Na]$^+$ calculated 1017.298, found 1017.298.

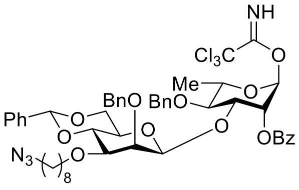

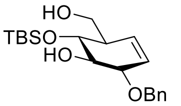

2-*O*-benzyl-3-*O*-*tert*-butyldimethylsilyl-β-glucose-cyclohexene (**73**). Compound **6**[14] (0.5 g, 0.5 mmol) was co-evaporated with toluene (3x), dissolved in dry DCM (10 mL, 0.05 M) under N_2 and cooled to 0 °C. TFA (0.62 mL, 8 mmol, 16 eq.) and TES-H (0.24 mL, 1.5 mmol, 3 eq.) were added and after stirring for 60 min at 0 °C the reaction was finished and quenched with sat. aq. $NaHCO_3$ (10 mL) and stirred vigorously. The organic layer was washed with sat. aq. $NaHCO_3$, brine, dried over $MgSO_4$, filtrated and concentrated *in vacuo*. Column chromatography (10% -> 60% Et_2O in pentane) yielded compound **73** (0.11 g, 0.31 mmol) in 63%. ^{1}H NMR (400 MHz, $CDCl_3$) δ 7.48 – 7.19 (m, 6H, H-Aromatic), 5.76 (ddd, J = 10.2, 2.8, 2.0 Hz, 1H, H-1/H-7), 5.60 (dt, J = 10.2, 2.1 Hz, 1H, H-1/H-7), 4.78 – 4.65 (m, 2H, CH_2-Bn), 4.07 – 3.99 (m, 1H, H-2), 3.78 – 3.66 (m, 4H, H-3, H-4, H-6), 2.38 – 2.25 (m, 1H, H-5), 0.92 (s, 9H, TBS), 0.15 (d, J = 9.9 Hz, 6H, TBS).^{13}C NMR (101 MHz, $CDCl_3$) δ 138.6, 128.7, 128.6, 128.5, 128.2, 127.9, 127.8 (C-Aromatic, C-1, C-7), 80.0 (C-2), 76.8 (C-3/C-4), 71.9 (CH_2-Bn), 71.8 (C-3/C-4), 62.3 (C-6), 47.0 (C-5), 26.1 (TBS), -3.6, -4.6 (TBS). HRMS [M+Na]$^+$ calculated 387.1965, found 387.1962.

2-*O*-benzyl-6-*O*-TBDPS -3-*O*-TBS-β-glucose-cyclophellitol (**7**). Compound **73** (0.11 g, 0.31 mmol) was dissolved in DCM (6.28 mL, 0.05 M) and cooled to 0 °C. mCPBA (0.22 g, 0.1.26 mmol, 4 eq.) and $NaHCO_3$ (0.13 g, 1.6 mmol, 5 eq.) were added and the reaction was left to stir at 4 °C. After stirring for 50 hours TLC showed almost full conversion and the reaction mixture was quenched with sat. aq. $NaHCO_3$ (5 mL) and sat. aq. $Na_2S_2O_3$ (5 mL). The mixture was diluted with DCM, washed with sat. aq. $NaHCO_3$, brine, dried over $MgSO_4$, filtrated and concentrated *in vacuo*. Column chromatography (5% -> 50% Et_2O in pentane) yielded partially purified compound (0.1 g, 0.26 mmol) in 84%. This compound was then co-evaporated with toluene (3x), dissolved in dry DMF (2.6 mL, 0.1 M) under N_2 and cooled to 0 °C. TBDPS-Cl (0.081 mL, 0.32 mmol, 1.2 eq.) and imidazole (0.054 g, 0.79 mmol, 3 eq.) were then added and the reaction mixture was allowed to reach room temperature. After stirring for 18 hours TLC indicated full conversion and the reaction mixture was diluted with Et_2O. The organic layer was washed with H_2O, brine, dried over $MgSO_4$, filtrated and concentrated *in vacuo*. Column chromatography (2% -> 10% Et_2O in pentane) yielded compound **7** (0.2 g, 0.26 mmol) quantitatively. ^{1}H NMR (500 MHz, $CDCl_3$) δ 7.91 – 7.65 (m, 7H, H-Aromatic), 7.50 – 7.36 (m, 16H, H-Aromatic), 4.88 – 4.78 (m, 2H, CH_2-Bn), 4.06 (dd, J = 9.4, 4.3 Hz,

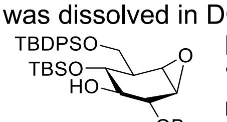

1H, H-6), 3.81 – 3.65 (m, 3H, H-2, H-6, H-7), 3.53 (dd, J = 9.6, 8.2 Hz, 1H, H-3), 3.32 (d, J = 3.7 Hz, 1H, H-1), 3.24 (t, J = 9.5 Hz, 1H, H-4), 2.23 (dddd, J = 10.4, 9.3, 4.3, 1.7 Hz, 1H, H-5), 1.13 (d, J = 6.5 Hz, 17H, TBDPS), 0.79 (s, 9H, TBS), 0.06 (d, J = 7.1 Hz, 3H, TBS), -0.21 (s, 3H, TBS). ^{13}C NMR (126 MHz, $CDCl_3$) δ 138.0, 135.7, 135.7, 135.4, 135.0, 133.6, 133.5, 129.8, 129.8, 129.8, 128.7, 128.0, 127.9, 127.8 (C-Aromatic), 79.1 (C-2), 76.7 (C-3), 73.1 (CH_2-Bn), 69.1 (C-4), 63.3 (C-6), 55.3 (C-7), 54.3 (C-1), 45.2 (C-5), 27.0 (TBDPS), 26.7 (TBDPS), 26.0 (TBS), 19.4 (Quaternary), 19.1 (Quaternary), 18.2 (Quaternary), -3.8 (TBS), -4.7 (TBS). HRMS [M+Na]$^+$ calculated 614.3093, found 614.3089.

2-*O*-benzyl-4,6-*O*-benzylidene-3-*O*-(8-azido-octane)-β-D-mannose-(1->3)-2-*O*-benzoyl-4-*O*-benzyl-α-L-rhamnose-(1->3)-2-*O*-benzyl-6-*O*-TBDPS-3-*O*-TBS-β-glucose-cyclophellitol (**12**). Donor **11** (0.17 g, 0.17 mmol, 1.3 eq.) and acceptor **7** (0.08 g, 0.13 mmol) were co-evaporated with toluene (3x) and dissolved in dry DCM (1.2 mL, 0.108 M) under N_2. 3 Å MS were added and the solution was stirred for 1 hour. The solution was then cooled to -40 °C and TMSOTf (0.1 mL, 0.013 mmol, 0.13 M in DCM) was added, the reaction was left to stir at -40 °C. After 3 hours the reaction was allowed to reach -20 °C and after 1 hour TLC indicated complete conversion and the reaction was quenched with Et_3N and diluted further with DCM. The organic layer was washed with sat. aq. $NaHCO_3$, brine, dried over $MgSO_4$, filtrated and concentrated *in vacuo*. Size exclusion chromatography (DCM: MeOH 1:1) yielded compound **12** (0.118 g, 0.081 mmol) in 63%. ^{1}H NMR (500 MHz, $CDCl_3$) δ 8.12 – 8.03 (m, 2H, H-Aromatic), 7.73 – 7.02 (m, 39H, H-Aromatic), 5.71 (d, J = 1.6 Hz, 1H, H-1’), 5.50 (s, 1H, H-Benzylidene), 5.43 (dd, J = 3.6, 1.6 Hz, 1H, H-2), 4.84 – 4.66 (m, 5H, CH_2-Bn), 4.61 (d, J = 12.5 Hz, 1H, CH_2-Bn), 4.58 (s, 1H, H-1”), 4.48 (d, J = 11.9 Hz, 1H, CH_2-Bn), 4.35 (dd, J = 9.7, 3.6 Hz, 1H, H-3’), 4.23 (dd, J = 10.4, 4.8 Hz, 1H, H-6”), 4.06 (dq, J = 9.6, 6.2 Hz, 1H, H-5’), 4.00 – 3.95 (m, 1H, H-6), 3.95 – 3.89 (m, 2H, H-2, H-4”), 3.83 (t, J = 10.3 Hz, 1H, H-6”), 3.77 – 3.71 (m, 2H, H-1/H-7, H-3), 3.64 (dd, J = 10.8, 9.2 Hz, 1H, H-6), 3.58 (t, J = 9.7 Hz, 1H, H-4’), 3.50 (d, J = 3.0 Hz, 1H, H-2”), 3.36 (t, J = 9.6 Hz, 1H, H-4), 3.30 (dt, J = 9.3, 6.6 Hz, 1H, CH_2-Linker), 3.24 – 3.17 (m, 4H, H-1/H-7, CH_2-Linker (3H)), 3.09 (td, J = 9.7, 4.9 Hz, 1H, H-5”), 2.96 (dd, J = 9.9, 3.0 Hz, 1H, H-3”), 2.28 (dddd, J = 10.9, 9.5, 4.5, 1.5 Hz, 1H, H-5), 1.64 – 1.52 (m, 2H, CH_2-Linker), 1.52 – 1.42 (m, 2H, CH_2-Linker), 1.36 – 1.20 (m, 11H, H-6’, CH_2-Linker), 1.07 (s, 9H, TBDPS), 0.70 (s, 9H, TBS), 0.09 (s, 3H, TBS), -0.30 (s, 3H, TBS). ^{13}C NMR (126 MHz, $CDCl_3$) δ 165.7, 138.7, 138.7, 137.7, 137.5, 135.7, 135.7, 133.6, 133.4, 133.1, 130.4, 129.9, 129.8, 128.8, 128.5, 128.4, 128.4, 128.4, 128.3, 128.2, 128.1, 128.1, 128.0, 127.9, 127.8, 127.5, 127.4, 126.5, 126.1 (C-Aromatic), 103.6 (C-1”), 101.2 (C-Benzylidene), 96.3 (C-1’), 82.3 (C-4’), 81.0 (C-4”), 79.1 (C-3”), 78.1 (C-2), 77.3 (C-3), 76.4(C-3’), 75.0 (CH_2-Bn), 75.0 (C-2”), 74.0 (CH_2-Bn), 73.7 (C-2’), 72.6 (CH_2-Bn), 70.8 (CH_2-Linker), 68.7 (C-6”), 67.8 (C-5”), 67.5 (C-5’), 67.0 (C-4), 63.7 (C-6), 54.8 (C-1/C-7), 54.2 (C-1/C-7), 51.5 (CH_2-Linker), 46.1 (C-5), 29.9, 29.8, 29.4, 29.2, 28.9 (CH_2-Linker), 27.0 (TBDPS), 26.8 (CH_2-Linker), 26.2 (TBS), 26.0 (CH_2-Linker), 19.4 (Quaternary), 18.1 (C-6), 18.0 (Quaternary), -2.7 (TBS), -4.1 (TBS). HRMS [M+H]$^+$ calculated 1469.743, found 1469.742.

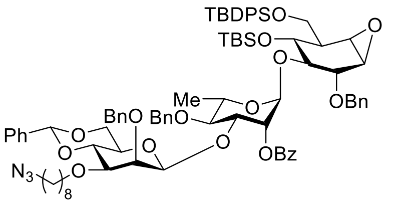

3-*O*-(8-amino-octane)-β-D-mannose-(1->3)-α-L-rhamnose-(1->3)-β-glucose-cyclophellitol (**13**). Compound **12** (0.05 g, 0.034 mmol) was dissolved in THF (1.15 mL, 0.03 M) and TBAF (0.14 mL, 0.14 mmol, 0.1 M, 4 eq.) was added. After stirring for 4 hours TLC indicated full conversion and the reaction mixture was diluted with EtOAc, washed with H_2O, sat. aq. $NaHCO_3$, brine, dried over $MgSO_4$, filtrated and concentrated *in vacuo*. Crude **12** (0.044 g, 0.04 mmol) was obtained quantitatively. ^{1}H NMR (400 MHz, $CDCl_3$) δ 8.13 – 8.00 (m, 2H, H-Aromatic), 7.81 – 7.67 (m, 4H, H-Aromatic), 7.62 – 7.13 (m, 31H, H-Aromatic), 5.50 (dd, J = 3.3, 1.9 Hz, 1H, H-2’), 5.45 (s, 1H, H-1’), 5.35 (d, J = 1.9 Hz, 1H, H-Benzylidene), 4.91 – 4.46 (m, 7H, H-1”, CH_2-Bn), 4.23 (dd, J = 9.5, 3.4 Hz, 1H, H-3’), 4.09 (dd, J = 10.6, 5.0 Hz, 1H, H-6”), 4.06 – 3.97 (m, 2H, H-6, H-5’), 3.96 – 3.90 (m, 2H, H-6, H-4”), 3.83 (d, J = 7.4 Hz, 1H, H-2), 3.71 – 3.61 (m, 4H, H-3, H-4', H-2'', H-6''), 3.57 (t, J = 9.6 Hz, 1H, H-4), 3.40 (dt, J = 9.3, 6.5 Hz, 1H, CH_2-Linker), 3.32 – 3.26 (m, 1H, CH_2-Linker), 3.21 (dd, J = 9.3, 4.6 Hz, 5H, CH_2-Linker, H-1/H-7, H-5”), 3.13 (dd, J = 7.6, 3.4 Hz, 2H, H-1/H-7, H-3”), 2.14 (dtd, J = 9.8, 4.9, 2.4 Hz, 1H, H-5), 1.62 – 1.45 (m, 4H, CH_2-Linker), 1.41 (dd, J = 9.7, 6.7 Hz, 3H, H-6’), 1.28 (qd, J = 15.0, 14.5, 6.0 Hz, 8H, CH_2-Linker). ^{13}C NMR (101 MHz, $CDCl_3$) δ 165.5, 138.6, 138.1, 137.7, 137.3, 135.5, 135.4, 134.9, 134.6, 134.5, 133.4, 130.4, 130.1, 129.9, 129.7, 128.9, 128.7, 128.6, 128.5, 128.5, 128.2, 128.2, 128.1, 128.1, 128.0, 128.0, 127.8, 127.5, 127.5, 127.2, 126.1 (C-Aromatic), 103.6 (C-1”), 101.3 (C-1’), 98.6 (C-Benzylidene), 85.2 (C-3/C-4’/C-2”), 81.6 (C-3/C-4’/C-2”), 79.1 (C-5”), 79.0 (C-2), 78.1 (C-4”), 77.0 (C-3’), 75.5 (C-3/C-4’/C-2”), 75.4 (CH_2-Bn), 74.2 (CH_2-Bn), 73.8 (CH_2-Bn), 73.7 (C-2’), 70.9 (CH_2-Linker), 69.0 (C-4/C-5’), 68.9 (C-4/C-5’), 68.5 (C-6”), 67.8 (C-3”), 64.3 (C-6), 55.2 (C-1/C-7), 53.0 (C-1/C-7), 51.5 (CH_2-Linker), 43.2 (C-5), 30.0, 29.4, 29.2, 28.9, 26.8, 26.1 (CH_2-Linker),

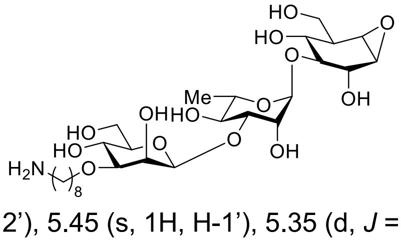

18.4 (C-6). Crude **12** (0.037 g, 0.034 mmol) was dissolved in THF (0.85 mL, 0.04 M) and purged with N_2. PtO_2 (0.0031 g, 0.014 mmol, 0.04 eq.) was added and the mixture was purged again with N_2. Subsequently, the reaction was purged with H_2 and left to stir under H_2 atmosphere. After 21 hours, TLC showed complete conversion and the reaction mixture was purged with N_2, filtrated over a Whatman glassfiber filter and concentrated *in vacuo*. The reduced compound (0.018 g, 0.017 mmol) was then co-evaporated with toluene (3x) and dissolved in dry THF (0.68 mL, 0.025 M) under N_2 and t-BuOH (0.065 mL, 0.68 mmol, 64 eq.). Ammonia (±10 mL) was condensed at -60 °C while maintaining a dry atmosphere and Na (0.016 g, 0.68 mmol, 40 eq.) was added, while using a glass stirring bar. After stirring for 10-20 min the Na was completely dissolved and the reduced desylilated compound **12** was added dropwise. After stirring for 1 hour, the reaction mixture was quenched with solid NH_4Cl (0.044 g, 0.82 mmol, 48 eq.) and the ammonia was evaporated at room temperature. The crude product was then dissolved in MeOH (0.5 mL, 0.038 M) and NaOMe was added until pH 10/11 was reached. After stirring for 5 hours TLC indicated complete conversion and the reaction was quenched with AcOH and concentrated *in vacuo*. Column chromatography over a pre-packed C18 column (0% -> 75% MeCN in water) yielded pure compound **13** (5.3 mg, 8.66 µmol) in 51% over 3 steps.^{1}H NMR (500 MHz, D_2O) δ = 5.13 (d, *J*=1.8, 1H, H-1’), 4.76 – 4.73 (m, 1H, H-1”), 4.20 (d, *J*=3.1, 1H, H-2”), 4.15 (dd, *J*=3.3, 1.8, 1H, H-2’), 3.95 – 3.84 (m, 4H, H-2, H-6, H-3’, H-5’), 3.82 (dd, *J*=12.0, 2.5, 1H, H-6”), 3.72 (dd, *J*=11.3, 7.2, 1H, H-6), 3.66 – 3.60 (m, 2H, H-6”, CH_2-Linker), 3.50 (t, *J*=9.0, 1H, H-4”), 3.49 – 3.44 (m, 3H, H-1/H-7, H-4’, CH_2-Linker), 3.44 – 3.38 (m, 1H, H-3), 3.33 (dd, *J*=9.6, 3.1, 1H, H-3”), 3.30 – 3.23 (m, 2H, H-4, H-5”), 3.12 (d, *J*=3.9, 1H, H-1/H-7), 2.88 (t, *J*=7.6, 2H, CH_2-Linker), 2.05 (d, *J*=9.0, 1H, H-5), 1.53 (dt, *J*=20.1, 7.0, 4H, CH_2-Linker), 1.24 (s, 8H, CH_2-Linker), 1.16 (d, *J*=6.2, 3H, H-6’).^{13}C NMR (126 MHz, D_2O) δ 101.5 (C-1”), 99.7 (C-1’), 82.1 (C-3), 80.7 (C-3”), 79.2 (C-2/C-3’), 76.3 (C-4/C-5”), 71.5 (C-2/C-3’), 71.1 (C-4’), 70.5 (C-2’), 69.6 (CH_2-Linker), 68.9 (C-5’), 67.4 (C-2”), 65.8 (C-4”), 65.0 (C-4/C-5”), 61.1 (C-6”), 60.4 (C-6), 56.3 (C-1/C-7), 56.0 (C-1/C-7), 43.3 (C-5), 39.5 (CH_2-Linker), 28.3, 28.1, 26.4, 25.0 (CH_2-Linker), 16.6 (C-6). HRMS $[M+H]^+$ calculated 612.3229, found 612.3226.

3-*O*-(8-amide Cy5-octane)-β-D-mannose-(1->3)-α-L-rhamnose-(1->3)-β-glucose-cyclophellitol (**4**).

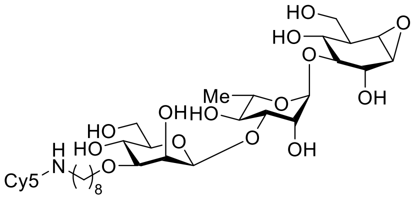

A pre-activated Cy5 acid solution was prepared by freeze drying Cy5 (6.4 mg, 0.013 mmol, 1 eq.), subsequently dissolving it in dry DMF (0.1 mL, 0.132 M) and adding DIPEA (0.1 mL, 0.066 mmol, 0.66 M in dry DMF, 5 eq.), pentafluoro-phenol (0.1 mL, 0.033 mmol, 0.33 M in dry DMF, 2.5 eq.). After stirring for 2 hours, LC-MS indicated full conversion to the mixed anhydride and the reaction was quenched with H_2O (0.1 mL, 0.066 mmol, 0.66 M in dry DMF, 5 eq.) and additional DIPEA (0.1 mL, 0.066 mmol, 0.66 M in dry DMF, 5 eq.). Subsequently 0.05 mL of dry DMF was added to a final concentration of 0.022 M activated Cy5. Of this pre-activated mixture 0.2 mL (0.0044 mmol Cy5, 1.5 eq.) was added to compound **13** (1.8 mg, 2.94 µmol, 1 eq.). After 3 hours LC-MS showed full conversion, and the reaction mixture was diluted with H_2O and freeze dried. HPLC purification (MeCN/H_2O/AcOH) yielded compound **4** (1.06 mg, 0.984 µmol) in 33%.^{1}H NMR (600 MHz, D_2O) δ 7.89 (t, *J* = 13.1 Hz, 2H, Cy5), 7.45 (d, *J* = 7.4 Hz, 2H, Cy5), 7.35 (td, *J* = 7.5, 3.8 Hz, 4H,Cy5), 7.26 – 7.18 (m, 4H, Cy5), 6.44 (t, *J* = 12.4 Hz, 1H, Cy5), 6.14 (dd, *J* = 13.8, 6.9 Hz, 2H, Cy5), 5.19 (d, *J* = 1.8 Hz, 1H, H-1’), 4.74 (s, 1H, H-1”), 4.21 (dd, *J* = 3.3, 1.8 Hz, 1H, H-2’), 4.14 (d, *J* = 3.1 Hz, 1H, H-2”), 4.06 (t, *J* = 6.7 Hz, 2H, CH_2-Linker//CH_2-Cy5 linker), 4.02 – 3.98 (m, 1H, H-5’), 3.96 (dd, *J* = 11.6, 4.1 Hz, 1H, H-6), 3.94 – 3.89 (m, 2H, H-2’, H-3’), 3.86 (dd, *J* = 12.3, 2.3 Hz, 1H, H-6”), 3.79 (dd, *J* = 11.3, 7.3 Hz, 1H, H-6), 3.67 (dd, *J* = 12.3, 6.4 Hz, 1H, H-6”), 3.57 – 3.52 (m, 6H CH_3-Cy5, H-7. H-4’, H-4”), 3.51 – 3.45 (m, 2H, H-3, CH_2-Linker (1H)), 3.32 (t, *J* = 10.1 Hz, 1H, H-4), 3.30 – 3.25 (m, 2H, CH_2-Linker (1H), H-5”), 3.21 – 3.16 (m, 2H, H-1, H-3”), 2.97 (t, *J* = 7.1 Hz, 2H, CH_2-Linker//CH_2-Cy5 linker), 2.18 – 2.11 (m, 3H, H-5, CH_2-Cy5), 1.87 (s, 10H, Acetic acid), 1.83 – 1.76 (m, 2H, CH_2-Linker//CH_2-Cy5 linker), 1.61 – 1.57 (m, 2H, CH_2-Cy5), 1.57 – 1.53 (m, 12H, 4x CH_3-Cy5), 1.46 – 1.38 (m, 2H, CH_2-Linker), 1.33 – 1.25 (m, 10H, CH_2-Linker/CH_2-Cy5), 1.24 (d, *J* = 6.2 Hz, 3H, H-6’), 1.14 (s, 8H, CH_2-Linker/CH_2-Cy5).^{13}C NMR (151 MHz, D_2O) δ 182.4, 177.1, 174.6, 174.4, 154.1, 143.8, 143.0, 142.2, 142.1, 129.5, 129.4, 126.0, 125.9, 125.2, 123.3, 123.2, 112.0, 111.6, 104.0 (Cy5), 102.3 (C-1”), 100.7 (C-1’), 83.1 (C-3), 81.6 (C-3”), 80.0 (C-2/C-3’), 77.1 (C-5”), 72.4 (C-2/C-3’), 72.1 (C-4’/C-4”), 71.4 (C-2’), 70.4 (Linker), 69.8 (C-5’), 68.2 (C-2”), 66.6 (C-4’/C-4”), 65.9 (C-4), 61.9 (C-6”), 61.2 (C-6), 57.1 (C-7), 56.9 (C-1), 50.0 (Cy5-Quartenary), 49.8 (Cy5-Quartenary), 44.5 (CH_2-Linker//CH_2-Cy5 linker), 44.2 (C-5), 40.3 (CH_2-Linker//CH_2-Cy5 linker), 36.3 (CH_2-Cy5), 31.6 (CH_3-Cy5), 29.7, 29.5, 29.3, 29.2 (CH_2-Linker/Cy5), 27.9 (CH_3-Cy5), 27.7 (CH_3-Cy5), 27.7, 27.1, 26.3, 26.0, 25.9 (CH_2-Linker/Cy5), 17.5 (C-6). HRMS $[M]^+$ calculated 1076.606, found 1076.605.

Synthesis of decasaccharide 18

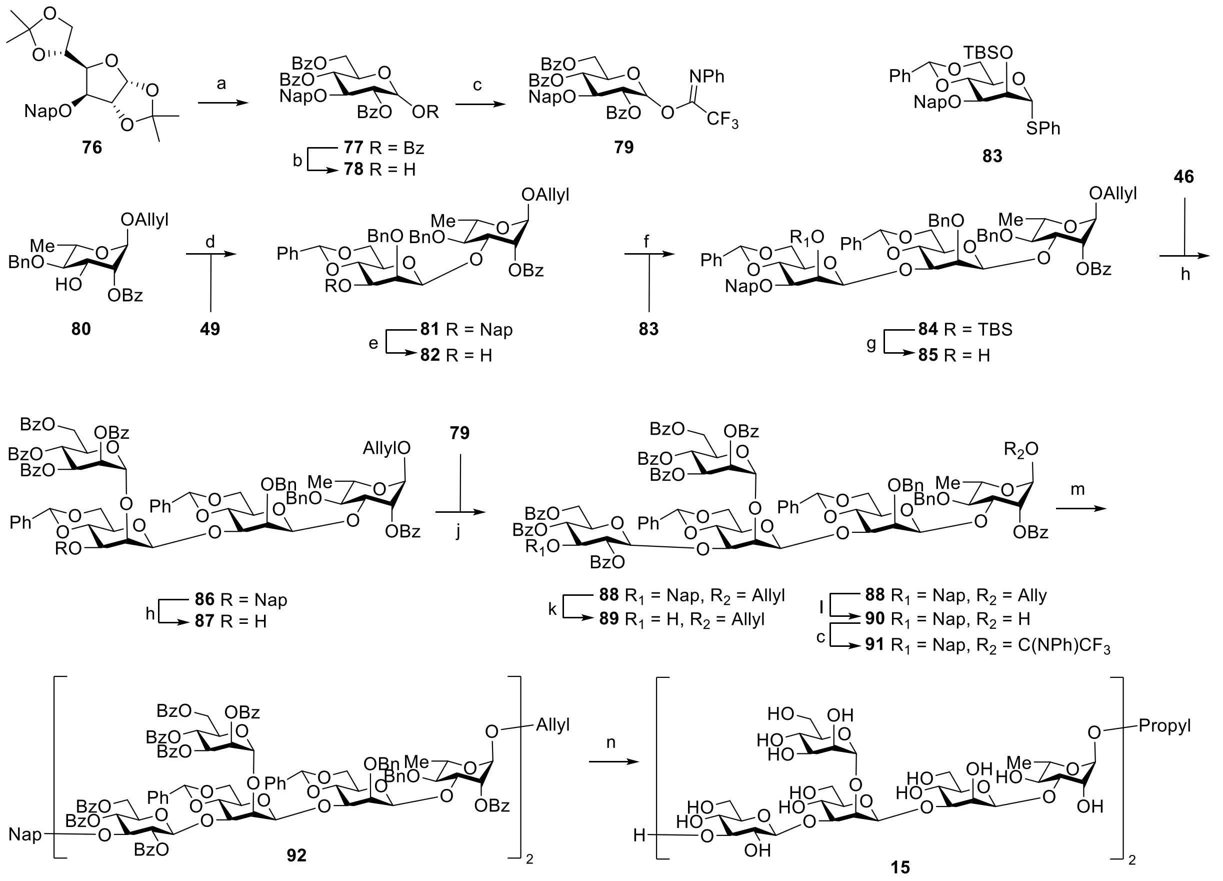

Scheme 5 a) i) NapBr, NaH, DMF, quant; ii) 2 M HCl, EtOH, 80 °C; iii) BzCl, DMAP, pyridine, 62%; b) $MeNH_2$, DCM:THF, 77%; c) 2,2,2-trifluoro-N-phenylacetimidoyl chloride, Cs_2CO_3, DCM, 100% for **97**, 76%, for **103**; d) BSP, Tf_2O, TTBP, DCM, 3 Å MS, -60 °C, 72%; e) DDQ, DCM:H2O, 9:1, quant; f) BSP, Tf_2O, TTBP, DCM, 3 Å MS, -60 °C, 70%; g) HF-pyridine, pyridine, 68% together with 22% of **77**; h) TMSOTf, DCM, 3 Å MS, -20 °C, 73%; i) DDQ, DCM:H_2O, 9:1, 94%; j) TfOH, DCM, 3 Å MS, 0 °C -> rt, 79%; k) DDQ, DCM:H_2O, 9:1, quantitative; l) i) {Ir(COD)[$PCH_3(C_6H_5)_2$]$_2$}PF_6, H_2; ii) NIS, $NaHCO_3$, H_2O, 66%; m) TfOH, DCM, 3 Å MS, 0 °C, 59%, 47% of **100** recovered; n) i) Pd black, H_2, H_2O:dioxane, 1:2; ii) Na, NH3, t-BuOH, -60 °C, 41%

1,2:5,6-di-*O*-isopropylidene-3-*O*-naphthyl-α-D-glucofuranose (**76**). 1,2:5,6-Di-O-isopropylidene-α-D-glucofuranose (5.2 g, 20 mmol) was dissolved in dry DMF (100 mL, 0.2 M) under N_2 and cooled to 0 °C. NapBr (6.6 g, 30 mmol, 1.5 eq.) and NaH (1.2 g, 30 mmol, 1.5 eq., 60 wt% in mineral oil) were added. The reaction mixture was allowed to slowly reach room temperature. After stirring for 118h TLC showed complete conversion and the reaction was cooled to 0 °C and slowly quenched with MeOH. The reaction mixture was diluted with Et_2O and washed with water. The water layer was extracted with Et_2O until all the compound was removed. The combined organic layers were washed with brine, dried over $MgSO_4$, filtrated and concentrated in vacuo. Column chromatography (5% -> 30% Et_2O in pentane) yielded pure compound **76** (8.1 g, 20.3 mmol) in quantitative yield.

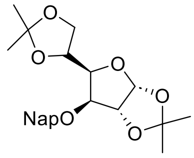

1,2,4,6-tetra-*O*-benzoyl-3-*O*-naphthyl-β-D-glucopyranose (**77**). Compound **76** (8 g, 20 mmol) was dissolved in EtOH (40 mL, 0.5 M) and 2 M HCl (20 mL, 2 eq.) was added, and the reaction mixture was heated to 80 °C. After stirring for 1h15min TLC showed complete conversion, and the reaction mixture was cooled to room temperature and quenched with Amberlite IRN78 OH-form. The resin was filtered off and washed with MeOH. The mixture was concentrated *in vacuo*, co-evaporated with toluene, redissolved/suspended in cold EtOAc and filtrated again. The suspension was then concentrated *in vacuo* again. The crude product was then co-evaporated 3 times with toluene, dissolved in pyridine (80 mL, 0.25 M) and cooled to 0 °C. DMAP (0.24 g, 2 mmol, 0.1 eq.) and BzCl (18.6 mL, 160 mmol, 8 eq.) were then added and the reaction mixture was allowed to slowly reach room temperature. After stirring for 69 hours the reaction was quenched with H_2O at 0 °C and dilute with Et_2O. The organic layer was washed with 1 M HCl (3x), sat. aq. $NaHCO_3$, brine, dried over $MgSO_4$, filtrated and concentrated *in vacuo*. The crude product was then recrystallized

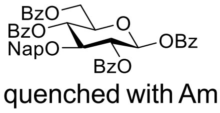

from ethyl acetate and pentane. To yield compound **77** (12.5 mmol, 9.2 g) in 62%. ^{1}H NMR (500 MHz, $CDCl_3$) δ 8.04 – 7.06 (m, 27H, H-Aromatic), 6.17 (d, *J* = 7.8 Hz, 1H, H-1), 5.82 – 5.68 (m, 2H. H-2, H-4), 4.85 – 4.73 (m, 2H, CH_2-Nap), 4.62 (dd, *J* = 12.3, 3.1 Hz, 1H, H-6), 4.43 (dd, *J* = 12.3, 5.0 Hz, 1H, H-6), 4.36 – 4.18 (m, 2H, H-2, H-4).^{13}C NMR (126 MHz, $CDCl_3$) δ 166.3, 165.1, 164.9, 134.6, 133.9, 133.6, 133.6, 133.1, 133.1, 133.0, 130.3, 129.9, 129.9, 129.7, 129.2, 129.2, 128.7, 128.6, 128.6, 128.6, 128.4, 128.3, 127.9, 127.7, 127.1, 126.1, 126.0, 126.0 (C-Aromatic), 92.7 (C-1), 79.4 (C-3/C-5), 74.5 (CH_2-Nap), 73.2 (C-3/C-5), 72.3 (C-2/C-4), 70.5 (C-2/C-4), 63.0 (C-6). HRMS $[M+NH_4]^+$ calculated 754.2647, found 754.2650.

2,4,6-tri-O-benzoyl-3-O-naphthyl-D-glucopyranose (**78**). Compound **77** (0.73 g, 0.99 mmol) was dissolved in DCM:THF (3.95 mL, 0.25 M, 9:1) under N_2 and $MeNH_2$ (5.4 mmol, 2.7 mL, 2 M, 5.5 eq.) was added. After 7.5 hours TLC indicated full conversion, and the reaction was quenched with 1 M HCl and diluted with DCM. The organic layer was washed with 1 M HCl, brine, dried over $MgSO_4$, filtrated and concentrated *in vacuo*. The crude product was re-crystallized from ethyl acetate and pentane to yield compound **78** (0.48 g, 0.76 mmol) in 77%. ^{1}H NMR (400 MHz, $CDCl_3$) δ 8.15 – 7.06 (m, 22H, H-Aromatic), 5.66 (t, *J* = 3.5 Hz, 1H, H-1), 5.62 (dd, *J* = 10.2, 9.2 Hz, 1H, H-4), 5.26 (dd, *J* = 9.9, 3.6 Hz, 1H, H-2), 4.92 – 4.75 (m, 2H, CH_2-Nap), 4.61 (dd, *J* = 12.2, 2.9 Hz, 1H, H-6), 4.55 – 4.44 (m, 2H, H-3, H-5), 4.36 (dd, *J* = 12.1, 4.4 Hz, 1H, H-6), 3.11 (d, *J* = 3.6 Hz, 1H, 1-OH). ^{13}C NMR (101 MHz, $CDCl_3$) δ 137.2, 133.6, 133.5, 133.2, 133.0, 129.9, 129.9, 129.5, 128.6, 128.5, 128.5, 128.2, 127.9, 127.7, 126.9, 126.1, 126.0, 125.9 (C-Aromatic), 90.7 (C-1), 76.9 (C-3), 75.4 (CH_2-Nap), 74.1 (C-2), 71.0 (C-4), 68.0 (C-5), 63.1 (C-6). HRMS $[M+Na]^+$ calculated 655.1939, found 655.1940.

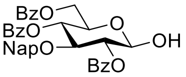

2,4,6-tri-O-benzoyl-3-O-naphthyl-1-O-trifluoro-N-phenyl-imidate-D-glucopyranose (**79**). Compound **78** (0.48 g, 0.76 mmol) was coevaporated 3 times with toluene and dissolved in dry DCM (3.8 mL, 0.2M) under N_2. 2,2,2-trifluoro-N-phenylacetimidoyl chloride (0.19 mL, 1.14 mmol, 1.5 eq) and Cs_2CO_3 (0.37 g, 1.14 mmol, 1.5 eq.) were added. After stirring for 16 hours TLC showed full conversion and the reaction mixture was diluted with DCM and filtrated over Celite. Column chromatography (10% -> 30% Et_2O in pentane) yielded compound **79** (0.61 g, 0.76 mmol) in 100% as an α/β mixture. ^{1}H NMR (400 MHz, $CDCl_3$) δ 8.26 – 6.96 (m, 45H), 5.78 (dt, *J* = 14.7, 9.5 Hz, 2H), 5.70 – 5.58 (m, 1H), 5.03 – 4.89 (m, 2H), 4.88 (s, 1H), 4.77 – 4.65 (m, 1H), 4.64 – 4.43 (m, 3H), 4.35 – 4.22 (m, 1H).^{13}C NMR (101 MHz, $CDCl_3$) δ 166.2, 166.2, 165.2, 165.1, 165.1, 164.8, 142.9, 134.8, 134.5, 133.8, 133.7, 133.6, 133.6, 133.3, 133.2, 133.1, 133.0, 133.0, 133.0, 129.9, 129.9, 129.9, 129.7, 129.6, 129.2, 129.1, 129.1, 129.1, 129.0, 128.8, 128.7, 128.7, 128.6, 128.6, 128.5, 128.4, 128.3, 128.3, 127.9, 127.9, 127.7, 127.7, 127.0, 126.9, 126.0, 126.0, 125.9, 125.9, 125.4, 124.5, 119.3, 119.2, 78.9, 77.1, 75.4, 74.3, 73.3, 72.3, 72.1, 70.9, 70.3, 70.1, 63.1, 62.7, 21.5. HRMS $[M+Na]^+$ calculated 826.2234, found 826.2241.

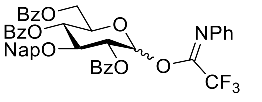

2-*O*-benzyl-4,6-*O*-benzylidene-3-*O*-naphthyl-β-D-mannose-(1->3)-1-*O*-allyl-2-*O*-benzoyl-4-*O*-benzyl-α-L-rhamnose (**81**)[15,16] Compound **49** (9.9 mmol, 5.86 g, 1.5 eq.), TTBP (14.8 mmol, 4.92 g, 3 eq.), and BSP (11.9 mmol, 2.5 g, 1,8 eq.) were coevaporated (3x) with toluene and dissolved in dry DCM (132 mL, 0.05 M). Then, 3Å MS were added and the solution was stirred for 1h under N_2. The mixture was then cooled down to -60 °C and Tf_2O (11.9 mmol, 2 mL, 1.8 eq.) was added. After stirring for 1h, compound **10** (6.6 mmol, 2.64 g, 1 eq.) was added and the solution was stirred for 90 minutes. After completion, the reaction was quenched with Et_3N (23 mmol, 2.9 mL, 3.5 eq.) and diluted with DCM. The mixture was then extracted with sat. aq. $NaHCO_3$ (2x) and brine. Subsequently, it was dried over $MgSO_4$, filtrated, and concentrated *in vacuo*. The crude residue was subjected to silica gel chromatography (5% -> 30% EtOAc in pentane) and compound **81** (4.75 mmol, 4.17 g) was yielded in 72% as a white solid. Rf = 0.18 (Pe:Et_2O, 9:1). ^{1}H NMR (500 MHz, $CDCl_3$) δ 8.14 – 7.08 (m, 27H, H-Aromatic), 5.91 (dddd, *J* = 17.2, 10.4, 6.0, 5.2 Hz, 1H, CH-Allyl), 5.54 (s, 1H, Benzylidene), 5.43 (dd, *J* = 3.4, 1.8 Hz, 1H, H-2), 5.30 (dq, *J* = 17.3, 1.7 Hz, 1H, CH_2-Allyl), 5.20 (dq, *J* = 10.4, 1.4 Hz, 1H, CH_2-Allyl), 4.97 (d, *J* = 1.7 Hz, 1H, H-1), 4.79 (d, *J* = 12.4 Hz, 1H, CH_2-Bn/Nap), 4.75 – 4.60 (m, 5H, CH_2-Bn/Nap, H-1'), 4.56 (d, *J* = 11.4 Hz, 1H, CH_2-Bn/Nap), 4.29 (dd, *J* = 10.4, 4.8 Hz, 1H, H-6'), 4.24 (dd, *J* = 9.6, 3.4 Hz, 1H, H-3), 4.21 – 4.09 (m, 2H, H-4' CH_2-Allyl (1H)), 4.02 (ddt, *J* = 13.0, 6.1, 1.4 Hz, 1H, CH_2-Allyl), 3.90 – 3.83 (m, 1H, H-5), 3.84 – 3.79 (m, 1H, H-6'), 3.76 – 3.71 (m, 1H, H-2'), 3.66 (t, *J* = 9.6 Hz, 1H, H-4), 3.44 – 3.37 (m, 1H, H-3'), 3.33 – 3.22 (m, 1H, H-5'), 1.38 (d, *J* = 6.3 Hz, 3H, H-6). ^{13}C NMR (126 MHz, $CDCl_3$) δ 165.8, 138.7, 138.3, 137.8, 136.0, 133.7, 133.4, 133.2, 133.0, 130.3, 129.9, 128.9, 128.6, 128.6, 128.6, 128.5, 128.5, 128.4, 128.3, 128.3, 128.1, 128.1, 128.0, 128.0, 127.9, 127.8,

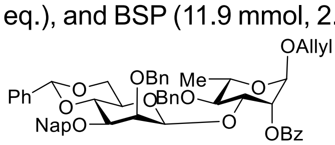

127.5, 127.2, 126.3, 126.1, 126.1, 125.9, 125.6, 117.8 (C-Aromatic), 103.7 (C-1’), 101.5 (Benzylidene), 96.5 (C-1), 81.7 (C-4), 78.5 (C-4’), 78.3 (C-3), 78.0 (C-3), 76.0 (C-2’), 75.4 (CH_2-Bn/nap), 74.4 (CH_2-Bn/nap), 73.7 (C-2), 72.4 (CH_2-Bn/nap), 68.6 (C-6’), 68.4 (CH_2-Allyl), 67.9 (C-5), 67.7 (C-5’), 18.3 (C-6). HRMS $[M+H]^+$ calculated 896.400, found 896.399.

2-*O*-benzyl-4,6-*O*-benzylidene-β-D-mannose-(1->3)-1-*O*-allyl-2-*O*-benzoyl-4-*O*-benzyl-α-L-rhamnose (**82**). Compound **81** (1.3 g, 1.5 mmol) was dissolved in DCM:H_2O (30 mL, 0.05 M, 9:1), DDQ (0.68 g, 3 mmol, 2 eq.) was added and the reaction was allowed to stir in the dark. After 1h20min TLC indicated full conversion, and the reaction mixture was diluted with DCM and quenched with sat. aq. $NaHCO_3$. The organic layer was then washed with sat. aq. $NaHCO_3$, sat. aq. $Na_2S_2O_3$, brine, dried over $MgSO_4$, filtrated and concentrated *in vacuo*. Column chromatography (10% -> 50% Et_2O in pentane) yielded compound **82** (1.14 g, 1.55 mmol) quantitatively. ^1H NMR (400 MHz, $CDCl_3$) δ = 8.16 – 6.98 (m, 20H, H-Aromatic), 6.00 – 5.86 (m, 1H, CH-Allyl), 5.44 (dd, *J*=3.4, 1.8, 1H, H-2), 5.41 (s, 1H, H-Benzylidene), 5.35 – 5.19 (m, 2H, CH_2-Benzylidene), 4.94 (s, 1H, H-1), 4.89 – 4.70 (m, 4H, H-1’, CH_2-Bn), 4.45 (d, *J*=11.5, 1H, CH_2-Bn), 4.32 – 4.23 (m, 2H, H-3, H-6’), 4.19 (ddt, *J*=12.9, 5.3, 1.5, 1H, CH_2-Allyl), 4.02 (ddt, *J*=12.9, 6.1, 1.4, 1H, CH_2-Allyl), 3.92 – 3.82 (m, 1H, H-5), 3.76 – 3.64 (m, 4H, H-4, H-2’, H-4’, H-6’), 3.60 – 3.53 (m, 1H, H-3’), 3.23 (td, *J*=9.7, 4.9, 1H, H-5’), 2.50 (s, 1H, OH-3’), 1.40 (d, *J*=6.2, 3H, H-6). ^{13}C NMR (101 MHz, $CDCl_3$) δ 165.6, 138.0, 138.0, 137.3, 133.6, 133.2, 130.0, 129.8, 129.5, 129.1, 128.8, 128.7, 128.6, 128.6, 128.6, 128.4, 128.4, 128.3, 128.3, 128.2, 128.2, 128.1, 128.0, 127.8, 127.7, 127.5, 126.4, 126.3, 126.3(C-Aromatic) , 117.7 (CH_2-Allyl), 103.5 (C-1’), 101.9 (Benzylidene), 96.4 (C-1), 81.5, 79.1, 78.4 (C-2’, C-4, C-4’), 77.9 (C-3), 75.6 (CH_2-Bn), 75.1 (CH_2-Bn), 73.5 (C-2), 70.7 (C-3’), 68.5 (C-6’), 68.3 (CH_2-Allyl), 67.8 (C-5), 67.1 (C-5’), 18.2 (C-6). HRMS $[M+Na]^+$ calculated 761.2932, found 761.2923.

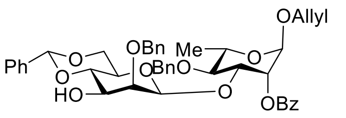

2-*O*-t-butyldimethylsilyl-4,6-*O*-benzylidene-3-*O*-naphthyl-β-D-mannose-(1->3)-2-*O*-benzyl-4,6-*O*-benzylidene-β-D-mannose-(1->3)-1-*O*-allyl-2-*O*-benzoyl-4-*O*-benzyl-α-L-rhamnose (**84**). Donor **83**[15] (1.9 g, 3.1 mmol, 1.7 eq.), BSP (0.68 g, 3.3 mmol, 1.8 eq.) and TTBP (1.4 g, 5.4 mmol, 3 eq.) were coevaporated three times with toluene, dissolved in dry DCM (36 mL, 0. 05 M) and 3 Å MS were added. After stirring for 60 minutes the reaction mixture was cooled to -60 °C and Tf_2O (0.55mL, 3.3 mmol, 1.8 eq.) was added dropwise. After stirring for 1 hour, co-evaporated acceptor **82** (1.4 g, 1.8 mmol) was added as a solution in dry DCM (2 mL) and the flask was washed with DCM (1 mL). Column chromatography (5% -> 30% Et_2O in pentane) yielded almost pure compound **84** (1.65 g, 1.26 mmol) in 70%. ^1H NMR (500 MHz, $CDCl_3$) δ = 8.42 – 7.41 (m, 32H, H-Aromatic), 6.28 – 6.15 (m, 1H, CH-Allyl), 5.87 (s, 1H, CH-Benzylidene), 5.75 – 5.72 (m, 2H, H-2, H-Benzylidene), 5.61 (dq, *J*=17.2, 1.6, 1H, CH_2-Allyl), 5.51 (dq, *J*=10.4, 1.4, 1H, CH_2-Allyl), 5.25 (d, *J*=1.7, 1H, H-1), 5.24 – 5.07 (m, 3H, CH_2-Bn/Nap), 5.06 (s, 1H, H-1”), 5.05 – 4.96 (m, 2H. CH_2-Bn/Nap), 4.79 (d, *J*=12.3, 1H, CH_2-Bn/Nap), 4.60 (dd, *J*=10.5, 4.7, 1H, H-6’), 4.57 (dd, *J*=9.5, 3.5, 1H, H-3), 4.52 – 4.44 (m, 4H), 4.40 (dd, *J*=3.0, 1.7, 1H), 4.37 (d, *J*=9.3, 1H), 4.34 – 4.29 (m, 2H), 4.27 (d, *J*=3.1, 2H, H-1’), 4.26 – 4.18 (m, 3H), 4.17 – 4.14 (m, 2H, H-5), 4.14 – 4.05 (m, 4H), 4.02 (d, *J*=3.2, 1H), 3.97 (t, *J*=9.6, 1H, H-4), 3.63 (td, *J*=9.7, 4.8, 1H, H-5’), 3.59 (dd, *J*=9.7, 2.9, 1H, H-3”), 3.34 (td, *J*=9.6, 4.7, 1H, H-5”), 1.70 (d, *J*=6.2, 3H, H-6), 1.11 (s, 9H, TBS), 0.37 (d, *J*=2.6, 3H, TBS), 0.28 (s, 3H, TBS). ^{13}C NMR (126 MHz, $CDCl_3$) δ 165.8, 138.4, 138.3, 137.9, 137.6, 136.2, 133.8, 133.5, 133.3, 133.1, 130.2, 129.9, 129.1, 129.0, 128.9, 128.7, 128.6, 128.4, 128.4, 128.3, 128.2, 128.1, 128.1, 128.1, 127.9, 127.9, 127.9, 127.8, 127.7, 127.1, 126.6, 126.4, 126.4, 126.4, 126.3, 126.2, 126.0, 125.8, 125.8, 117.9 (C-Aromatic), 104.0 (C-1’), 101.8 (Benzylidene), 101.7 (Benzylidene), 96.8 (C-1/C-1”), 96.6 (C-1/C-1”), 81.8, 79.5, 79.0, 78.1, 77.8, 77.5, 77.3, 77.0, 76.6, 75.6, 75.5, 74.3, 74.2, 74.0, 73.7, 72.8, 72.1, 71.5, 71.2, 69.0, 68.7, 68.5, 68.0, 67.9, 67.8, 64.6, 26.0, 18.3, -4.2, -4.5. HRMS $[M+Na]^+$ calculated 1265.526, found 1265.526.

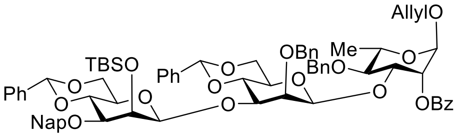

4,6-*O*-benzylidene-3-*O*-naphthyl-β-D-mannose-(1->3)-2-*O*-benzyl-4,6-*O*-benzylidene-β-D-mannose-(1->3)-1-*O*-allyl-2-*O*-benzoyl-4-*O*-benzyl-α-L-rhamnose (**85**). Compound **84** (0.98 g, 0.79 mmol) was c-evaporated 3x with toluene, cooled to 0 °C, dissolved in pyridine (19.7 mL, 0.04 M) and HF-pyridine (10.2 mL, 0.39 m, 500 eq.) was added. The reaction mixture was then allowed to reach room temperature. After stirring for 23 hours, the reaction mixture was diluted with more than 300 mL of ethyl acetate and washed with H_2O, sat. aq. $NaHCO_3$, 1 M HCl, brine, dried over $MgSO_4$, filtrated and concentrated *in vacuo*. The reaction was not yet finished and

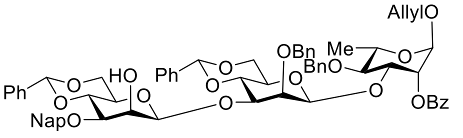

restarted using the amounts described above. After stirring for an additional 18 hours, TLC showed complete conversion and the reaction mixture was diluted with more than 300 mL of ethyl acetate, washed with H_2O, sat. aq. $NaHCO_3$, 1 M HCl, brine, dried over $MgSO_4$, filtrated and concentrated *in vacuo*. Column chromatography (10% -> 30% EtOAc in pentane) yielded compound **85** (0.6 g, 0.53 mmol) in 68%, together with compound **81** (0.13 g, 0.18 mmol) in 22%. ^{1}H NMR (400 MHz, $CDCl_3$) δ 8.14 – 6.89 (m, 32H, H-Aromatic), 6.04 – 5.86 (m, 1H, CH-Allyl), 5.47 (s, 1H, H-Benzylidene), 5.43 (dd, *J* = 3.5, 1.7 Hz, 1H, H-2), 5.38 (s, 1H, H-Benzylidene), 5.31 (dq, *J* = 17.2, 1.6 Hz, 1H, CH_2-Allyl), 5.25 – 5.20 (m, 1H, CH_2-Allyl), 4.96 (d, *J* = 1.7 Hz, 1H, H-1), 4.91 (d, *J* = 2.2 Hz, 2H, CH_2-Bn/Nap), 4.81 – 4.50 (m, 5H, H-1’, CH_2-Bn/Nap), 4.33 – 4.28 (m, 1H, H-6’), 4.27 (dd, *J* = 9.7, 3.6 Hz, 1H, H-3), 4.19 (ddt, *J* = 13.0, 5.3, 1.5 Hz, 1H, CH_2-Allyl), 4.16 – 4.07 (m, 1H, H-4”), 4.06 – 4.02 (m, 3H, H-1”, H-6”, CH_2-Allyl), 4.02 – 3.98 (m, 1H, H-4’), 3.91 – 3.77 (m, 3H, H-5, H-3’, H-2”, H-6”), 3.71 – 3.62 (m, 3H, H-4, H-2’, H-6”), 3.47 (dd, *J* = 9.3, 3.8 Hz, 1H, H-3”), 3.30 (td, *J* = 9.6, 4.8 Hz, 1H, H-5’), 3.07 (td, *J* = 9.8, 4.9 Hz, 1H, H-5”), 1.40 (d, *J* = 6.2 Hz, 1H, H-6). ^{13}C NMR (101 MHz, $CDCl_3$) δ 165.7, 138.2, 137.5, 137.3, 135.7, 133.6, 133.3, 133.2, 133.1, 130.1, 129.8, 129.1, 129.0, 128.6, 128.4, 128.3, 128.2, 128.2, 128.2, 128.1, 128.0, 127.9, 127.7, 127.6, 127.1, 126.7, 126.2, 126.2, 126.1, 126.0, 125.9 (C-Aromatic), 117.8 (CH_2-Allyl), 103.5 (C-1’), 101.6 (Benzylidene), 101.3 (Benzylidene), 96.9 (C-1”), 96.4 (C-1), 81.7 (C-4/C-2’), 78.3 (C-4”), 77.8 (C-3), 76.6 (C-4’), 75.8 (C-3”), 75.4 (CH_2-Bn/Nap), 74.8 (C-4/C-2’/C-3’/C-2”), 74.7 (C-4/C-2’/C-3’/C-2”), 74.2 (CH_2-Bn/Nap), 73.5 (C-2), 72.2 (CH_2-Bn/Nap), 69.6 (C-3’/C-2”), 68.6, 68.5, 68.4 (CH_2-Allyl/C-6’/C-6”), 67.8, 67.7 (C-5/C-5’), 66.9 (C-5”), 18.2 (C-6). HRMS $[M+Na]^+$ calculated 1151.440, found 1151.439.

2,3,4,6-*O*-benzoyl-α-D-mannose-(1->2)-4,6-*O*-benzylidene-3-*O*-naphthyl-β-D-mannose-(1->3)-2-*O*-benzyl-4,6-*O*-benzylidene-β-D-mannose-(1->3)-1-*O*-allyl-2-*O*-benzoyl-4-*O*-benzyl-α-L-rhamnose (**86**). Acceptor **85** (0.36 g, 0.32 mmol) and donor **46** (0.61 g, 0.79 mmol, 2.5 eq.) were coevaporated three times with toluene, dissolved in dry DCM (3.1 mL, 0.1 M) under N_2 and 3 Å MS were added. After stirring for 1 hour with MS, the mixture was cooled to -20 °C and TMSOTf (0.1 mL, 0.32 M, 0.1 eq.) was added. The reaction mixture was allowed to reach 0 °C. After stirring for 2 hours, TLC showed complete conversion, and the reaction was quenched with Et_3N. The mixture was further diluted with DC M and washed with sat. aq. $NaHCO_3$, brine, dried over $MgSO_4$, filtrated and concentrated *in vacuo*. Size exclusion chromatography and subsequent column chromatography (5% -> 25% EtOAc in pentane) yielded compound **86** (0.4 g, 0.23 mmol) in 73%. ^{1}H NMR (500 MHz, $CDCl_3$) δ 8.16 – 6.84 (m, 52H, H-Aromatic), 6.17 (t, *J* = 10.3 Hz, 1H, H-4’”), 6.03 (dd, *J* = 3.3, 1.8 Hz, 1H, H-2’”), 5.99 – 5.92 (m, 1H, CH-Allyl), 5.92 – 5.89 (m, 1H, H-3’”), 5.76 (s, 1H, H-Benzylidene), 5.49 (d, *J* = 1.8 Hz, 1H, H-1’”), 5.46 (s, 1H, H-Benzylidene), 5.43 (dd, *J* = 3.4, 1.8 Hz, 1H, H-2), 5.32 (dq, *J* = 17.2, 1.6 Hz, 1H, CH_2-Allyl), 5.23 (dq, *J* = 10.4, 1.3 Hz, 1H, CH_2-Allyl), 5.03 (d, *J* = 12.4 Hz, 1H, CH_2-Bn/Nap), 4.97 (d, *J* = 1.7 Hz, 1H, H-1), 4.87 (d, *J* = 12.3 Hz, 1H, H-5’”), 4.84 – 4.74 (m, 3H, CH_2-Bn/Nap), 4.74 (s, 1H, H-1’), 4.70 – 4.61 (m, 3H, H-6’”, CH_2-Bn/Nap), 4.30 (t, *J* = 9.5 Hz, 1H, H-4”), 4.28 – 4.23 (m, 3H, H-3, H-6’, H-6”), 4.20 (ddt, *J* = 13.0, 5.3, 1.5 Hz, 1H, CH_2-Allyl), 4.15 – 4.11 (m, 1H, H-6’”), 4.05 (ddt, *J* = 13.0, 6.1, 1.4 Hz, 1H, CH_2-Allyl), 4.01 – 3.94 (m, 3H, H-1”, H-2”, H-6”), 3.94 – 3.84 (m, 2H, H-5, H-6’), 3.76 – 3.64 (m, 4H, H-4, H-2’, H-3’, H-4’), 3.47 (dd, *J* = 9.7, 2.9 Hz, 1H, H-3”), 3.28 (td, *J* = 9.6, 4.8 Hz, 1H, H-5’), 3.10 (td, *J* = 9.7, 4.9 Hz, 1H, H-5”), 1.39 (d, *J* = 6.3 Hz, 3H, H-6). ^{13}C NMR (126 MHz, $CDCl_3$) δ 166.0, 165.7, 165.5, 165.1, 138.3, 138.0, 137.8, 137.5, 135.7, 133.7, 133.2, 133.0, 132.8, 130.5, 130.1, 129.9, 129.9, 129.8, 129.7, 129.7, 129.6, 129.4, 129.2, 129.0, 128.8, 128.7, 128.5, 128.5, 128.5, 128.4, 128.4, 128.3, 128.3, 128.2, 128.0, 127.9, 127.7, 127.7, 126.7, 126.6, 126.3, 126.1, 126.0, 125.9, 125.8, 117.9 (CH_2-Allyl), 104.1 (C-1’), 101.6 (Benzylidene), 100.8 (Benzylidene), 99.4 (C-1’”), 96.6 (C-1/C-1”), 96.5 (C-1/C-1”), 81.9 (C-4/C-2’/C-3’/C-4’), 79.3 (C-4”), 78.0 (C-3), 76.9 (C-3”), 76.4 (C-2”), 76.2 (C-5), 75.4 (C-4/C-2’/C-3’/C-4’), 75.2 (CH_2-Bn/Nap), 73.7 (CH_2-Bn/Nap), 73.6 (C-2’), 73.2 (CH_2-Bn/Nap), 73.1 (C-4/C-2’/C-3’/C-4’), 70.6 (C-3’”), 70.5 (C-2’”), 69.1 (C-5’”), 68.7 (C-6’), 68.5 (CH_2-Allyl), 68.3 (C-6”), 68.1 (C-5’), 67.8 (C-5), 67.6 (C-5”), 66.5 (C-4’”), 62.3 (C-6’”), 18.2 (C-6). HRMS $[M+Na]^+$ calculated 1730.60,1 found 1730.601.

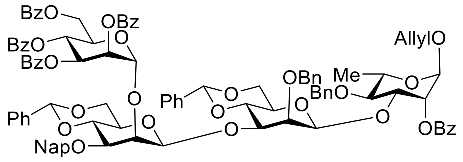

2,3,4,6-*O*-benzoyl-α-D-mannose-(1->2)-4,6-*O*-benzylidene-β-D-mannose-(1->3)-2-*O*-benzyl-4,6-*O*-benzylidene-β-D-mannose-(1->3)-1-*O*-allyl-2-*O*-benzoyl-4-*O*-benzyl-α-L-rhamnose (**87**). Compound **86** (0.52 g, 0.31 mmol) was dissolved in DCM:H_2O (9:1, 6.1 mL), DDQ (0.14 g, 0.61 mmol, 2 eq.) was added and the reaction was stirred in the dark. After

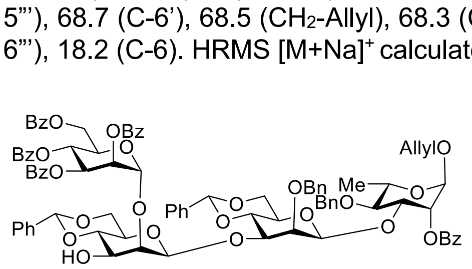

stirring for 2.3 hours TLC indicated full conversion and the reaction mixture was diluted with ethyl acetate, the organic layer was washed with sat. aq. $NaHCO_3$, sat. aq. $Na_2S_2O_3$ (2x), sat. aq. $NaHCO_3$, brine, dried over $MgSO_4$, filtrated and concentrated *in vacuo*. Column chromatography (10% -> 30% EtOAc in pentane) yielded compound **87** (0.45 g, 0.29 mmol) in 94%.^{1}H NMR (500 MHz, $CDCl_3$) δ 8.26 – 6.91 (m, 45H, H-Aromatic), 6.16 (q, *J* = 10. 5 Hz, 1H, H-4’”), 6.00 – 5.86 (m, 3H, CH-Allyl, H-2’”, H-3’”), 5.65 – 5.55 (m, 1H, H-Benzylidene), 5.48 (s, 1H, H-Benzylidene), 5.45 (dd, *J* = 3.4, 1.8 Hz, 1H, H-2), 5.42 (d, *J* = 1.7 Hz, 1H, H-1’”), 5.33 (dq, *J* = 17.2, 1.6 Hz, 1H, CH_2-Allyl), 5.26 – 5.21 (m, 1H. CH_2-Allyl), 4.99 (d, *J* = 1.7 Hz, 1H, H-1), 4.89 – 4.69 (m, 6H, H-1’, H-5’”, CH_2-Bn (2x)), 4.65 (dd, *J* = 12.3, 2.7 Hz, 1H, H-6’”), 4.29 (m, 2H. H-3, H-6’), 4.21 (m, 2H, H-6’, H-6”), 4.16 – 4.14 (m, 1H, H-1”), 4.09 – 4.02 (m, 2H, H-6”), 3.99 – 3.88 (m, 4H, H-5, H-4’, H-2”, H-4”), 3.80 – 3.66 (m, 6H, H-4, H-6’, H-2’, H-3’, H-3”), 3.31 (td, *J* = 9.6, 4.8 Hz, 1H, H-5’), 3.14 (td, *J* = 9.7, 5.0 Hz, 1H, H-5”), 1.41 (d, *J* = 6.2 Hz, 3H, H-6). ^{13}C NMR (126 MHz, $CDCl_3$) δ 166.0, 165.7, 165.6, 165.5, 165.3, 138.5, 137.9, 137.5, 137.3, 133.7, 133.4, 133.2, 133.1, 133.1, 132.9, 130.3, 130.1, 129.9, 129.9, 129.8, 129.8, 129.7, 129.6, 129.5, 129.2, 129.1, 128.9, 128.8, 128.7, 128.6, 128.6, 128.6, 128.6, 128.5, 128.5, 128.5, 128.5, 128.4, 128.3, 128.3, 128.3, 127.9, 127.9, 127.8, 127.4, 126.7, 126.4, 126.3, 126.0, 117.9 (C-Aromatic), 104.1 (C-1’), 101.9 (Benzylidene), 100.9 (Benzylidene), 99.1 (C-1’”), 96.5 (C-1), 81.9 (C-1”), 78.2, 77.4, 75.4, 75.2, 74.1, 73.6, 71.6, 70.6, 70.4, 69.2, 68.6 (C-6’’), 68.3 (C-6’), 68.0, 67.8, 67.2, 66.5, 62.3 (C-6’”), 18.2 (C-6). HRMS [M+Na]$^+$ calculated 1590.538, found 1590.539.

2,4,6-*O*-benzoyl-3-*O*-naphthyl-β-D-glucose-(1->3)-4,6-*O*-benzylidene-2-*O*-(2,3,4,6-*O*-benzoyl-α-D-mannose-(1->2))-β-D-mannose-(1->3)-2-*O*-benzyl-4,6-*O*-benzylidene-β-D-mannose-(1->3)-1-*O*-allyl-2-*O*-benzoyl-4-*O*-benzyl-α-L-rhamnose (**88**). Donor **79** (0.3 g, 0.371 mmol, 3 eq.) and acceptor **87** (0.19 g, 0.124 mmol, 1 eq.) were mixed and co-evaporated 3 times with toluene. The mixture was

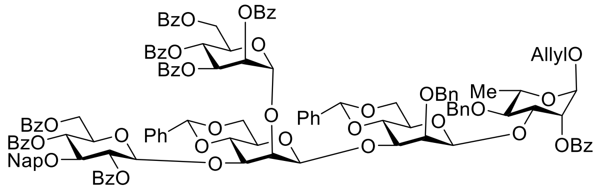

dissolved in dry DCM (1.2 mL, 0.1 M) and 3 Å MS were added. After stirring for 60 min the solution was cooled to 0 °C and TfOH (0.1 mL, 0.25 M, 0.2 eq.) was added. The ice bath was removed directly after addition of TfOH. After stirring for 2.5 h TLC showed full conversion and the reaction was quenched with Et_3N. The mixture was diluted with DCM, washed with H_2O, sat. aq. $NaHCO_3$, brine, dried over $MgSO_4$, filtrated and concentrated *in vacuo*. Column chromatography (10% -> 40% EtOAc in pentane) and subsequent size exclusion chromatography yielded **88** (0.21 g, 0.1 mmol) in 79%. ^{1}H NMR (500 MHz, $CDCl_3$) δ 8.18 – 6.72 (m, 77H, H-Aromatic), 6.22 (t, *J* = 10.3 Hz, 1H, H-4’”), 6.19 – 6.16 (m, 1H, H-2’”), 5.99 – 5.90 (m, 2H, H-3’”, CH-Allyl), 5.73 (t, *J* = 9.3 Hz, 1H, H-4””), 5.59 (s, 1H, H-Benzylidene), 5.57 (dd, *J* = 9.1, 7.5 Hz, 1H, H-2””), 5.51 (d, *J* = 1.8 Hz, 1H, H-1’”), 5.43 (s, 1H, H-Benzylidene), 5.41 (dd, *J* = 3.4, 1.7 Hz, 1H, H-2), 5.32 (dq, *J* = 17.2, 1.6 Hz, 1H, CH_2-Allyl), 5.23 (dq, *J* = 10.4, 1.3 Hz, 1H, CH_2-Allyl), 5.12 (d, *J* = 7.4 Hz, 1H, H-1””), 4.98 (d, *J* = 1.8 Hz, 1H, H-1), 4.84 – 4.78 (m, 2H, H-5’”, CH_2-Bn/Nap), 4.76 – 4.68 (m, 5H, H-1’, CH_2-Bn/Nap), 4.67 – 4.61 (m, 1H, CH_2-Bn/Nap), 4.54 (dd, *J* = 12.3, 2.7 Hz, 1H, H-6’”), 4.49 (dd, *J* = 12.0, 5.3 Hz, 1H, H-6””), 4.44 – 4.39 (m, 2H, H-4”, H-6””), 4.27 – 4.23 (m, 2H, H-3, H-6’), 4.22 – 4.15 (m, 2H, H-6’”, CH_2-Allyl), 4.14 – 4.09 (m, 2H, H-6”, H-3””), 4.08 – 4.02 (m, 1H, CH_2-Allyl), 3.93 – 3.91 (m, 1H, H-1”), 3.91 – 3.83 (m, 3H, H-5, H-4’, H-2”), 3.80 – 3.75 (m, 1H, H-5””), 3.73 – 3.62 (m, 6H, H-4, H-2’, H-3’, H-6’, H-3”, H-6”), 3.24 (td, *J* = 9.6, 4.8 Hz, 1H, H-5’), 3.04 (td, *J* = 9.6, 4.8 Hz, 1H, H-5”), 1.39 (d, *J* = 6.2 Hz, 3H, H-6). ^{13}C NMR (126 MHz, $CDCl_3$) δ 166.2, 166.0, 165.7, 165.6, 165.3, 165.1, 164.9, 164.9, 138.3, 137.8, 137.6, 137.4, 135.1, 133.7, 133.2, 133.2, 133.1, 133.0, 132.9, 132.8, 132.8, 132.7, 132.7, 130.5, 130.1, 130.1, 130.0, 129.9, 129.8, 129.8, 129.8, 129.7, 129.7, 129.6, 129.5, 129.4, 129.4, 129.2, 128.9, 128.8, 128.8, 128.5, 128.5, 128.5, 128.4, 128.4, 128.3, 128.2, 128.1, 128.1, 127.9, 127.8, 127.8, 127.6, 127.5, 127.4, 126.7, 126.6, 126.3, 126.2, 125.9, 125.9, 125.7, 125.6 (C-Aromatic), 117.8 (CH_2-Allyl), 104.0 (C-1’), 101.8 (Benzylidene), 100.5 (Benzylidene), 99.8 (C-1’”), 99.2 (C-1””), 96.5 (C-1’, C-2”), 81.9 (C-4/C-2’/C-3’/C-3”), 79.4 (C-3””), 78.0 (C-4”, C-4/C-2’/C-3’/C-3”), 77.9 (C-5/C-4'/C-2"), 76.0 (C-5/C-4'/C-2"), 75.6 (C-4/C-2’/C-3’/C-3”), 75.1 (CH_2-Bn/Nap), 73.7 (CH_2-Bn/Nap), 73.6 (C-2), 73.2 (C-2””), 73.1 (C-4/C-2’/C-3’/C-3”), 72.2 (C-5””), 72.1 (CH_2-Bn/Nap), 70.7 (C-2’”), 70.4 (C-3’”, C-4””), 70.3 (C-5’”), 69.1, 68.5 (C-6’), 68.2 (C-6”, CH_2-Allyl), 68.0 (C-5’), 67.8 (C-5/C-4'/C-2"), 67.5 (C-5”), 66.7 (C-4’”), 63.6 (C-6””), 62.4 (C-6’”), 18.1 (C-6).

2,4,6-*O*-benzoyl-β-D-glucose-(1->3)-4,6-*O*-benzylidene-2-*O*-(2,3,4,6-*O*-benzoyl-α-D-mannose-(1->2))-β-D-mannose-(1->3)-2-*O*-benzyl-4,6-*O*-benzylidene-β-D-mannose-(1->3)-1-*O*-allyl-2-*O*-benzoyl-4-*O*-benzyl-α-L-rhamnose (**89**). Compound **88** (0.1 g, 0.05 mmol) was dissolved in DCM:H_2O (9:1, 6.1 mL), DDQ (0.022 g, 0.096 mmol, 2 eq.) was added and the reaction was stirred in the dark.

After stirring for 3 hours TLC indicated almost full conversion and additional DDQ (0.005 g, 0.5 eq.) was

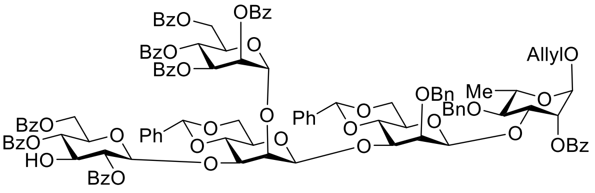

added. After stirring for an additional 30 minutes the reaction mixture was diluted with EA, the organic layer was washed with sat. aq. $NaHCO_3$, sat. aq. $Na_2S_2O_3$, brine, dried over $MgSO_4$, filtrated and concentrated in vacuo. Column chromatography (10% -> 40% EtOAc in pentane) yielded compound **89** (0.098 g, 0.05 mmol) in 100%. ^{1}H NMR (500 MHz, $CDCl_3$) δ 8.31 – 6.77 (m, 60H, H-Aromatic), 6.22 (t, *J* = 10.3 Hz, 1H, H-4'''), 6.13 (d, *J* = 3.0 Hz, 1H, H-2'''), 6.00 – 5.90 (m, 2H, CH-Allyl, H-3''')), 5.60 (s, 1H, H-Benzylidene), 5.50 – 5.46 (m, 1H, H-4''''), 5.46 – 5.45 (m, 1H, H-1'''), 5.45 – 5.43 (m, 1H, H-Benzylidene), 5.42 (dd, *J* = 3.4, 1.8 Hz, 1H, H-2), 5.36 – 5.30 (m, 2H, H-2'''', CH_2-Allyl), 5.23 (dq, *J* = 10.4, 1.3 Hz, 1H, CH_2-Allyl), 5.15 (d, *J* = 7.2 Hz, 1H, H-1''''), 4.98 (d, *J* = 1.8 Hz, 1H, H-1), 4.86 – 4.78 (m, 2H, H-5''', CH_2-Bn), 4.78 – 4.69 (m, 3H, H-1', CH_2-Bn), 4.65 (d, *J* = 12.1 Hz, 1H, CH_2-Bn), 4.55 (dd, *J* = 12.3, 2.6 Hz, 1H, H-6'''), 4.50 (d, *J* = 4.5 Hz, 2H, H-6'''' (2x)), 4.42 (t, *J* = 9.5 Hz, 1H, H-4''), 4.29 – 4.23 (m, 2H, H-3, H-6'), 4.23 – 4.13 (m, 3H, H-6'', H-6'''', CH_2-Allyl), 4.08 – 4.04 (m, 1H, CH_2-Allyl), 4.03 – 4.01 (m, 1H, H-3''''), 4.00 – 3.97 (m, 1H, H-1''), 3.93 – 3.81 (m, 4H, H-5, H-4', H-2'', H-5''''), 3.80 – 3.75 (m, 1H, H-3''), 3.74 – 3.65 (m, 5H, H-4, H-2', H-3', H-6', H-6''), 3.29 – 3.19 (m, 2H, H-5', OH''''), 3.09 (td, *J* = 9.7, 4.8 Hz, 1H, H-5''), 1.39 (d, *J* = 6.2 Hz, 3H, H-6). ^{13}C NMR (126 MHz, $CDCl_3$) δ 171.2, 166.2, 166.2, 166.0, 166.0, 165.7, 165.7, 165.5, 165.3, 138.4, 137.9, 137.6, 137.4, 133.7, 133.4, 133.3, 133.3, 133.2, 132.9, 132.9, 132.8, 132.8, 130.5, 130.1, 129.9, 129.9, 129.8, 129.8, 129.7, 129.6, 129.6, 129.4, 129.4, 129.2, 129.2, 128.9, 128.8, 128.6, 128.6, 128.5, 128.4, 128.4, 128.4, 128.3, 128.3, 128.2, 128.2, 128.2, 127.9, 127.7, 127.5, 126.7, 126.3, 126.1, 126.0 (C-Aromatic), 117.9 (CH_2-Allyl), 104.0 (C-1'), 101.8 (Benzylidene), 100.6 (Benzylidene), 99.7 (C-1'''), 99.0 (C-1''''), 96.6 (C-1''), 96.5 (C-1), 81.9 (C-4/C-2'/C-3'), 78.1 (C-3''), 78.0 (C-3, C-4''), 77.1 (C-5/C-4'/C-2''/C-5''''), 76.1 (C-5/C-4'/C-2''/C-5''''), 75.7 (C-4/C-2'/C-3'), 75.5 (C-2''''), 75.2 (CH_2-Bn (2x)), 73.9 (C-3''''), 73.9 (CH_2-Bn), 73.6 (C-2), 73.3 (C-4/C-2'/C-3'), 72.5 (C-4''''), 72.3 (C-5/C-4'/C-2''/C-5''''), 71.0 (C-2'''), 70.4 (C-3'''), 69.2 (C-5'''), 68.6 (C-6'/CH_2-Allyl), 68.5 (C-6'/CH_2-Allyl), 68.3 (C-6'), 68.1 (C-5'), 67.8 (C-5/C-4'/C-2''/C-5''''), 67.6 (C-5''), 66.6 (C-4'''), 63.8 (C-6''''), 62.4 (C-6'''), 18.2 (C-6). HRMS $[M+Na]^+$ calculated 2064.670, found 2064.674.

2,4,6-*O*-benzoyl-3-*O*-naphthyl-β-D-glucose-(1->3)-4,6-*O*-benzylidene-2-*O*-(2,3,4,6-*O*-benzoyl-α-D-mannose-(1->2))-β-D-mannose-(1->3)-2-*O*-benzyl-4,6-*O*-benzylidene-β-D-mannose-(1->3)-2-*O*-benzoyl-4-*O*-benzyl-α-L-rhamnose (**90**). Compound **88** (0.094 g, 0.043 mmol) was dissolved in freshly distilled THF (0.57 mL, 0.075 M) and degassed using Argon. {Ir(COD)[$PCH_3(C_6H_5)_2]_2$}PF_6 (1.8 mg, 2.2

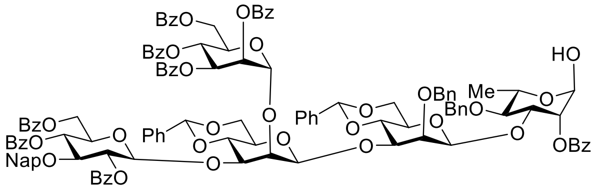

µmol, 0.05 eq.) was added and the mixture was shortly purged with Argon again. The reaction mixture was then purged with H_2 for 10 seconds and the color changed from reddish to orange. The reaction mixture was then flushed with Argon again. After 4 hours the THF was evaporated and NMR showed complete isomerization. The mixture was then taken up in THF (0.86 mL, 0.05 M), H_2O (0.13 mL) and NIS (0.015 g, 0.065 mmol, 1.5 eq.) and $NaHCO_3$ (5.4 mg, 0.065 mmol, 1.5 eq.) were added. After stirring for 4 hours TLC showed almost full conversion and additional NIS (0.015 g, 1.5 eq.), $NaHCO_3$ (5.4 mg, 1.5 eq.) were added. After stirring for an additional 1.5 h no further conversion was observed and the reaction mixture was diluted with EA, washed with sat. aq. $Na_2S_2O_3$, brine, dried over $MgSO_4$ and concentrated in vacuo. Column chromatography (20% -> 50% EtOAc in pentane) yielded compound **90** (0.061 g, 0.028 mmol) in 66%. ^{1}H NMR (600 MHz, $CDCl_3$) δ 8.27 – 6.91 (m, 67H, H-Aromatic), 6.74 (t, *J* = 7.6 Hz, 1H, H-Aromatic), 6.23 (t, *J* = 10.3 Hz, 1H, H-4'''), 6.20 – 6.14 (m, 1H, H-2'''), 5.96 (dd, *J* = 10.4, 3.2 Hz, 1H, H-3'''), 5.74 (t, *J* = 9.3 Hz, 1H, H-4''''), 5.60 (s, 1H, H-Benzylidene), 5.57 (dd, *J* = 8.9, 7.4 Hz, 1H, H-2''''), 5.51 (d, *J* = 1.9 Hz, 1HH-1'''), 5.46 – 5.43 (m, 1H, H-Benzylidene), 5.42 (dd, *J* = 3.3, 1.8 Hz, 1H, H-2), 5.37 – 5.34 (m, 1H, H-1), 5.12 (d, *J* = 7.4 Hz, 1H, H-1''''), 4.85 – 4.79 (m, 2H, H-5''', CH_2-Bn/Nap), 4.76 – 4.65 (m, 5H, H-1', CH_2-Bn/Nap), 4.63 (d, *J* = 12.2 Hz, 1H, CH_2-Bn/Nap), 4.55 (dd, *J* = 12.2, 2.5 Hz, 1H, H-6'''), 4.49 (dd, *J* = 12.0, 5.3 Hz, 1H, H-6''''), 4.44 – 4.39 (m, 2H, H-4'', H-6''''), 4.31 (dd, *J* = 9.6, 3.4 Hz, 1H, H-3), 4.27 – 4.23 (m, 1H, H-6'), 4.17 (dd, *J* = 12.5, 2.2 Hz, 1H, H-6'''), 4.14 – 4.07 (m, 3H, H-5, H-6'', H-3''''), 3.90 (s, 1H, H-1''), 3.89 – 3.82 (m, 2H, H-2'', H-4'), 3.80 – 3.73 (m, 1H, H-5''''), 3.73 – 3.66 (m, 4H, H-4, H-6', H-3'', H-6''), 3.66 – 3.61 (m, 3H, OH-1, H-2', H-3'), 3.24 (td, *J* = 9.6, 4.8 Hz, 1H, H-5'), 3.05 (td, *J* = 9.5, 4.7 Hz, 1H, H-5''), 1.37 (d, *J* = 6.1 Hz, 3H, H-6). ^{13}C NMR (151 MHz, CDCl3) δ 177.6, 171.4, 166.3,

166.1, 165.8, 165.7, 165.3, 165.2, 165.0, 165.0, 138.4, 137.8, 137.6, 137.5, 135.1, 133.3, 133.3, 133.2, 133.0, 133.0, 132.9, 132.8, 132.8, 132.8, 130.5, 130.2, 130.1, 130.1, 130.1, 130.0, 129.9, 129.9, 129.8, 129.8, 129.8, 129.7, 129.7, 129.6, 129.4, 129.2, 129.2, 129.2, 129.0, 128.8, 128.6, 128.5, 128.5, 128.5, 128.5, 128.5, 128.4, 128.4, 128.4, 128.2, 128.2, 128.2, 128.1, 128.1, 127.9, 127.9, 127.8, 127.7, 127.6, 127.5, 127.1, 126.9, 126.8, 126.7, 126.3, 126.2, 126.2, 126.1, 126.0, 125.9, 125.8, 125.7 (C-Aromatic), 104.1 (C-1’), 101.8 (Benzylidene), 100.5 (Benzylidene), 99.9 (C-1”’), 99.3 (C-1””), 96.5 (C-1”), 91.9 (C-1), 81.9 (C-4/C-3”), 79.4 (C-5/C-3””), 78.1 (C-4/C-3”), 78.0 (C-4”), 77.3 (C-3), 76.9 (C-4’/C-2”), 76.0 (C-4’/C-2”), 75.6 (C-2’/C-3’), 75.1 (CH2-Bn/Nap), 74.1 (C-2), 73.8 (CH2-Bn/Nap), 73.3 (C-2””), 73.1 (C-2’/C-3’), 72.2 (C-5””), 72.2 (CH2-Bn/Nap), 70.8 (C-2”’), 70.4 (C-3”’), 70.3 (C-4””), 69.2 (C-5”’), 68.5 (C-6”), 68.2 (C-6’), 68.1 (C-5’), 67.8 (C-5/C-3””), 67.5 (C-5”), 66.7 (C-4”’), 63.7 (C-6””), 62.4 (C-6”’), 18.3 (C-6). HRMS $[M+Na]^+$ calculated 2164.701, found 2164.705.

2,4,6-*O*-benzoyl-3-*O*-naphthyl-β-D-glucose-(1->3)-4,6-*O*-benzylidene-2-*O*-(2,3,4,6-*O*-benzoyl-α-D-mannose-(1->2))-β-D-mannose-(1->3)-2-*O*-benzyl-4,6-*O*-benzylidene-β-D-mannose-(1->3)-1-*O*-trifluoro-N-phenyl-imidate-2-*O*-benzoyl-4-*O*-benzyl-α-L-rhamnose (**91**). Compound **90** (0.06 g, 0.028 mmol) was coevaporated 3 times with toluene and dissolved in dry DCM (0.28 mL, 0.1 M) under N_2. 2,2,2-trifluoro-N-phenylacetimidoyl chloride (9.1 µL, 0.056 mmol, 2 eq) and Cs_2CO_3 (18 mg, 0.056 mmol, 2 eq.) were added. After stirring for 6 hours TLC showed full conversion and the reaction mixture was diluted with DCM and filtrated over Celite. Column chromatography (10% -> 35% EtOAc in pentane) yielded compound **91** (0.049 g, 0.021 mmol) in 76%. ^1H NMR (500 MHz, $CDCl_3$) δ 8.17 – 6.72 (m, 72H, H-Aromatic), 6.22 (t, *J* = 10.4 Hz, 1H, H-4”’), 6.18 (s, 1H, H-2”’), 5.97 (dd, *J* = 10.4, 3.2 Hz, 1H, H-3”’), 5.73 (t, *J* = 9.3 Hz, 1H, H-4””), 5.62 – 5.54 (m, 3H, H-2, H-2”, H-Benzylidene), 5.52 (d, *J* = 1.8 Hz, 1H, H-1”’), 5.44 (s, 1H, H-Benzylidene), 5.11 (s, 1H, H-1””), 4.86 – 4.78 (m, 2H, H-5”’, CH_2-Bn/Nap), 4.76 – 4.65 (m, 5H, H-1’, CH_2-Bn/Nap), 4.62 – 4.57 (m, 1H, CH_2-Bn/Nap), 4.54 (dd, *J* = 12.3, 2.6 Hz, 1H, H-6”’), 4.48 (dd, *J* = 12.1, 5.4 Hz, 1H, H-6””), 4.45 – 4.37 (m, 2H, H-4”, H-6””), 4.32 – 4.24 (m, 1H, H-3), 4.26 – 4.12 (m, 3H, H-6’, H-6”, H-6”’), 4.11 – 4.07 (m, 1H, H-3””), 4.02 – 3.96 (m, 2H, H-5, H-1”), 3.90 – 3.84 (m, 2H, H-4’, H-2”), 3.80 – 3.65 (m, 7H, H-4, H-2’, H-3’, H-6’, H-3”, H-6”, H-5””), 3.27 (td, *J* = 9.5, 5.2 Hz, 1H, H-5’), 3.06 (td, *J* = 9.6, 4.9 Hz, 1H, H-5”), 1.43 (d, *J* = 6.1 Hz, 3H, H-6). ^{13}C NMR (126 MHz, $CDCl_3$) δ 166.2, 166.1, 165.7, 165.3, 165.2, 165.0, 164.9, 143.3, 138.0, 137.7, 137.6, 137.5, 135.2, 133.5, 133.2, 133.2, 133.0, 133.0, 132.9, 132.8, 132.8, 132.7, 130.6, 130.2, 130.1, 130.0, 129.9, 129.8, 129.8, 129.8, 129.7, 129.6, 129.6, 129.4, 129.2, 129.2, 128.9, 128.8, 128.7, 128.6, 128.6, 128.5, 128.5, 128.4, 128.4, 128.3, 128.2, 128.2, 128.1, 128.1, 127.9, 127.9, 127.8, 127.7, 127.6, 127.5, 127.1, 126.7, 126.3, 126.2, 126.0, 125.9, 125.8, 125.6, 124.6, 119.5 (C-Aromatic), 104.0 (C-1’), 101.8 (Benzylidene), 100.7 (Benzylidene), 99.9 (C-1”’), 99.3 (C-1””), 96.5 (C-1”), 80.9 (C-4/C-2’/C-3’/C-3”/C-5””), 79.4 (C-3””), 78.1 (C-4”, C-4/C-2’/C-3’/C-3”/C-5””), 77.2 (C-3), 76.9 (C-4’/C-2”), 76.0 (C-4’/C-2”), 75.5 (C-4/C-2’/C-3’/C-3”/C-5””), 75.5 (CH_2-Bn/Nap), 73.9 (CH_2-Bn/Nap), 73.3 (C-2/C-2””, C-4/C-2’/C-3’/C-3”/C-5””), 72.3 (C-4/C-2’/C-3’/C-3”/C-5””), 72.2 (CH_2-Bn/Nap), 72.1 (C-2/C-2””), 70.8 (C-2”’), 70.6 (C-5), 70.4 (C-3”’), 70.4 (C4””), 69.2 (C-5”’), 68.6 (C-6”), 68.2 (C-5’), 68.1 (C-6’), 67.6 (C-5”), 66.8 (C-4”’), 63.7 (C-6””), 62.5 (C-6”’), 18.2 (C-6).

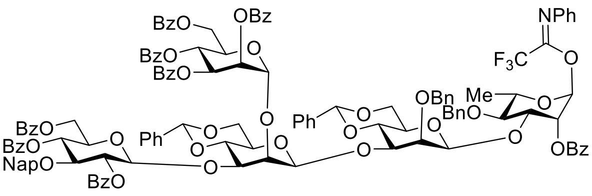

2,4,6-*O*-benzoyl-3-*O*-naphthyl-β-D-glucose-(1->3)-4,6-*O*-benzylidene-2-*O*-(2,3,4,6-*O*-benzoyl-α-D-mannose-(1->2))-β-D-mannose-(1->3)-2-*O*-benzyl-4,6-*O*-benzylidene-β-D-mannose-(1->3)-2-*O*-benzoyl-4-*O*-benzyl-α-L-rhamnose-(1->3)-2,4,6-*O*-benzoyl-β-D-glucose-(1->3)-4,6-*O*-benzylidene-2-*O*-(2,3,4,6-*O*-benzoyl-α-D-mannose-(1->2))-β-D-mannose-(1->3)-2-*O*-benzyl-4,6-*O*-benzylidene-β-D-mannose-(1->3)-1-*O*-allyl-2-*O*-benzoyl-4-*O*-benzyl-α-L-rhamnose (**92**). Donor **91** (0.049 g, 0.028 mmol, 1 eq.) and acceptor **89** (0.086 g, 0.042 mmol, 2 eq.) were mixed and co-evaporated 3 times with toluene. The mixture was dissolved in dry DCM (0.84 mL, 0.025 M) and 3 Å MS were added. After stirring for 60 min the solution was cooled to 0 °C and TfOH (0.1 mL, 0.042 M, 0.2 eq.) was added.

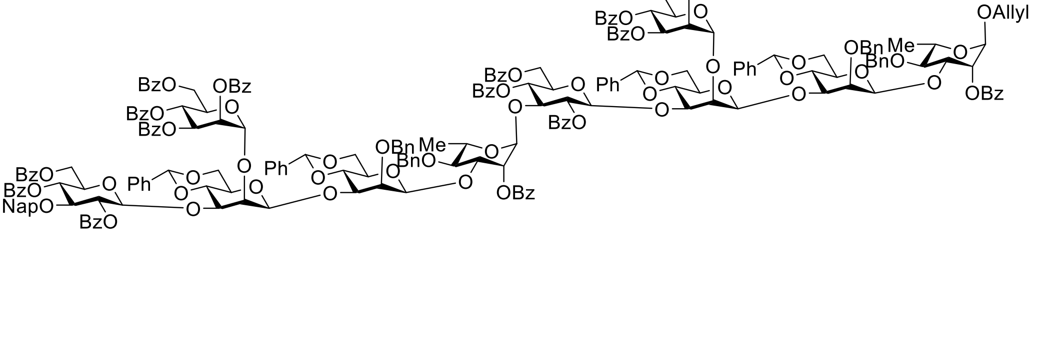

After stirring for 1.6 h TLC showed full conversion and the reaction was quenched with Et_3N. The mixture was diluted with DCM, washed with H_2O, sat. aq. $NaHCO_3$, brine, dried over $MgSO_4$, filtrated and concentrated *in vacuo*. size excl. chromatography yielded **92** (0.051 g, 0.012 mmol) in 59% and recovered acceptor **89** (0.041 g, 0.02 mmol) in 47%. ^1H NMR (850 MHz, $CDCl_3$) δ 8.22 – 6.62 (m, 127H), 6.24 – 6.18 (m, 3H), 6.17 – 6.15 (m, 1H), 5.99 – 5.91 (m, 4H), 5.75 – 5.71 (m, 2H), 5.71 – 5.66 (m, 1H), 5.59 (s, 1H), 5.57 – 5.54 (m, 2H), 5.55 – 5.50 (m, 3H), 5.49 (s, 1H), 5.46 (s, 1H), 5.42 (s, 1H), 5.42 – 5.39 (m, 2H), 5.35 – 5.30 (m, 2H), 5.25 (s, 1H), 5.25 – 5.22 (m, 1H), 5.13 – 5.08 (m, 3H), 5.09 – 5.06 (m, 1H), 5.00 – 4.96 (m, 2H), 4.85 – 4.78 (m, 3H), 4.77 – 4.64 (m, 9H), 4.65 – 4.50 (m, 8H), 4.51 – 4.45 (m, 5H), 4.45 – 4.37 (m, 6H), 4.29 (s, 1H), 4.27 – 4.21 (m, 4H), 4.22 – 4.18 (m, 4H), 4.14 – 4.09 (m, 3H), 4.10 – 4.02 (m, 6H), 3.97 (dd, J = 9.5, 3.4 Hz, 2H), 3.91 – 3.78 (m, 9H), 3.78 – 3.75 (m, 3H), 3.75 – 3.58 (m, 16H), 3.53 (d, J = 3.1 Hz, 1H), 3.50 – 3.45 (m, 2H), 3.39 – 3.34 (m, 1H), 3.26 – 3.18 (m, 2H), 3.06 – 2.97 (m, 3H), 2.60 (td, J = 9.5, 4.8 Hz, 1H), 1.39 (d, J = 6.2 Hz, 4H), 0.85 (d, J = 6.2 Hz, 3H). ^{13}C NMR (214 MHz, $CDCl_3$) δ 166.3, 166.2, 166.1, 166.0, 165.7, 165.4, 165.3, 165.3, 165.2, 165.1, 165.0, 164.9, 138.4, 138.4, 138.0, 137.8, 137.7, 137.6, 137.5, 137.4, 135.1, 133.7, 133.3, 133.3, 133.2, 133.2, 133.1, 133.0, 133.0, 132.9, 132.8, 132.8, 132.8, 130.5, 130.5, 130.2, 130.2, 130.1, 130.1, 130.0, 130.0, 129.9, 129.9, 129.9, 129.9, 129.8, 129.8, 129.8, 129.7, 129.7, 129.6, 129.4, 129.4, 129.4, 129.3, 129.2, 129.2, 129.1, 128.9, 128.8, 128.7, 128.6, 128.6, 128.6, 128.5, 128.5, 128.5, 128.5, 128.5, 128.4, 128.4, 128.3, 128.3, 128.3, 128.2, 128.2, 128.2, 128.2, 128.2, 128.1, 128.1, 127.9, 127.9, 127.9, 127.8, 127.8, 127.8, 127.6, 127.6, 127.6, 127.5, 127.5, 127.4, 126.7, 126.6, 126.3, 126.3, 126.3, 126.2, 126.2, 126.0, 126.0, 125.9, 125.8, 125.7, 117.9, 104.1, 102.8, 101.8, 101.7, 100.6, 100.3, 100.0, 99.8, 99.3, 99.0, 98.5, 96.5, 96.4, 96.4, 81.9, 81.1, 80.3, 79.4, 78.2, 78.2, 78.0, 77.9, 77.1, 76.8, 76.7, 76.0, 75.8, 75.5, 75.5, 75.2, 74.9, 74.5, 73.9, 73.9, 73.8, 73.6, 73.5, 73.2, 72.9, 72.9, 72.7, 72.2, 72.2, 72.1, 70.8, 70.7, 70.5, 70.4, 70.3, 69.2, 69.1, 68.5, 68.5, 68.2, 68.1, 67.8, 67.7, 67.5, 67.4, 66.7, 66.6, 63.6, 63.5, 62.4, 62.2, 18.2, 17.6.

β-D-glucose-(1->3)-2-*O*-(α-D-mannose-(1->2))-β-D-mannose-(1->3)-β-D-mannose-(1->3)-α-L-rhamnose-(1->3)-β-D-glucose-(1->3)-2-*O*-(α-D-mannose-(1->2))-β-D-mannose-(1->3)-β-D-mannose-(1->3)-1-*O*-propyl-α-L-rhamnose (**15**). Compound **92** was dissolved in DCM:MeOH (0.6 mL, 0.01 M, 1:1) and NaOMe (final concentration 0.05M) was added. The reaction mixture was diluted with DCM, washed with H_2O, brine, dried over $MgSO_4$, filtrated and concentrated *in vacuo*. The partially

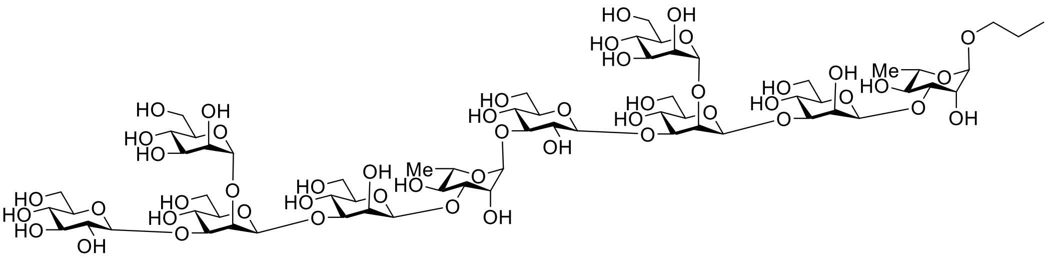

deprotected compound was then redissolved in dioxane:H_2O (1.5 mL, 0.018 M, 2:1) and 3 drops of acetic acid were added. The reaction mixture was then flushed with N_2 after which a catalytic amount of Pd black was added. The mixture was then shortly flushed with N_2, before shortly flushing with H_2 and then allowing the reaction to stir under an H_2 atmosphere. After stirring for 24 hours the naphthyl group and one benzylidene group were removed, together with reduction of the allyl to a propyl. The reaction mixture was then filtrated over a Whatman glassfiber filter and concentrated *in vacuo*. Partly reduced compound **92** was then dissolved in THF (0.4 mL, 0.015M) and t-BuOH (0.15 mL) was added. Sodium (9.9 mg, 0.43 mmol, 70 eq.) was then added to a freshly prepared NH_3 (~10 mL) solution at -60 °C. Then partially deprotected compound **92** was added, the flask was then washed with additional THF (0.2 mL) and t-BuOH (0.2 mL), still not everything was dissolved/added. The reaction mixture was stirred for one hour between -60 °C and -40 °C. The reaction mixture was quenched with AcOH and the NH_3 allowed to slowly evaporate at room temperature. Subsequently, the reaction mixture was concentrated *in vacuo*. Size exclusion yielded pure product **15** (4.16 mg, 2.5 µmol) in 41%. ^1H NMR (850 MHz, D_2O) δ 5.32 (d, J = 1.8 Hz, 1H), 5.30 (d, J = 1.8 Hz, 1H), 5.07 (d, J = 1.8 Hz, 1H), 4.80 – 4.77 (m, 4H), 4.73 (d, J = 1.8 Hz, 1H), 4.58 – 4.56 (m, 2H), 4.33 (d, J = 2.8 Hz, 1H), 4.31 (d, J = 2.9 Hz, 1H), 4.23 – 4.20 (m, 3H), 4.14 – 4.11 (m, 2H), 4.10 (dd, J = 3.3, 1.7 Hz, 1H), 4.02 – 3.97 (m, 3H), 3.95 – 3.91 (m, 2H), 3.91 – 3.78 (m, 16H), 3.73 – 3.60 (m, 16H), 3.60 – 3.54 (m, 5H), 3.53 – 3.47 (m, 3H), 3.47 – 3.41 (m, 3H), 3.41 – 3.37 (m, 4H), 3.37 – 3.30 (m, 6H), 3.28 (dd, J = 9.4, 7.9 Hz, 1H), 1.57 – 1.51 (m, 3H), 1.22 (d, J = 6.3 Hz, 3H), 1.20 – 1.17 (m, 3H), 0.84 (t, J = 7.4 Hz, 3H). ^{13}C NMR (214 MHz, D_2O) δ 101.2, 101.2, 100.9, 100.4, 100.3, 100.3, 99.2, 96.6, 82.5, 81.2, 80.7, 79.5, 79.3, 79.1, 79.1, 76.6, 76.6, 76.3,

76.3, 76.0, 76.0, 76.0, 73.6, 73.2, 73.0, 73.0, 72.2, 71.2, 71.1, 70.3, 70.2, 70.2, 70.1, 70.0, 69.6, 69.6, 68.8, 68.5, 68.1, 67.6, 66.6, 66.6, 65.6, 65.5, 65.0, 65.0, 61.0, 61.0, 60.7, 60.7, 21.9, 16.5, 16.4, 9.8. HRMS $[M+Na]^+$ calculated 1671.585, found 1671.589.

Synthesis of 18

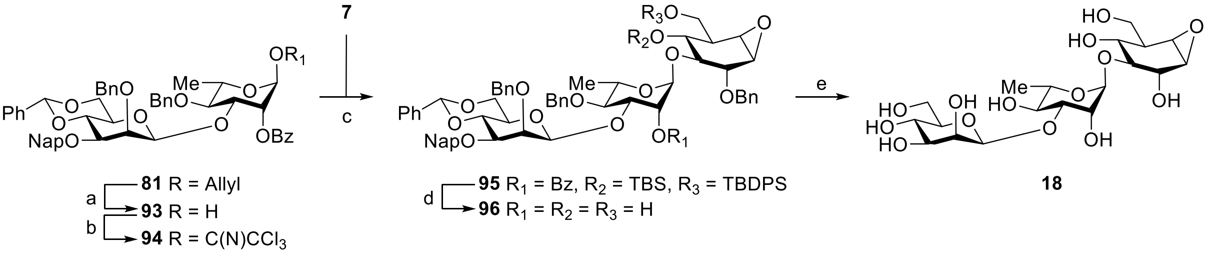

Scheme 6. a) i) $[Ir(PMePh_2)_2COD]^+$ PF_6^-, H_2, THF; ii) NIS, $NaHCO_3$, H_2O, quant; b) trichloroacetonitrile, DBU, DCM, 92%; c) TMSOTf, DCM, 3 Å MS, -40 ºC, 65%; d) i) NaOMe, DCM:MeOH, 1:1; ii) TBAF, THF; e) Na, NH_3, t-BuOH, THF, -60 °C, 52% over 3 steps.

2-*O*-benzyl-4,6-*O*-benzylidene-3-*O*-naphthyl-β-D-mannose-(1->3)-2-*O*-benzoyl-4-*O*-benzyl-α-L-rhamnose (**93**). Compound **81** was dissolved in freshly distilled THF (4.4 mL, 0.11 M) and argon was bubbled through under sonication for 20 min. $[Ir(PMePh_2)_2COD]^+$ PF_6^- (0.021 g, 0.025 mmol, 0.05 eq.) was dissolved in freshly distilled THF (2.1 mL, 0.012 M) and argon was bubbled through under sonication for 15 min. Then the catalyst was activated by bubbling through H_2 for 30 sec at which time all the red color had disappeared, subsequently the argon was bubbled through for 20-30 seconds to remove excess H_2. Then the degassed solution of compound **81** was added. After stirring for 1 hour TLC showed complete conversion and the reaction mixture was diluted with THF (3.5 mL) and H_2O (1 mL). Subsequently, $NaHCO_3$ (0.063 g, 0.75 mmol, 1.5 eq.) and NIS (0.17 g, 0.75 mmol, 1.5 eq.) were added. TLC showed full conversion after stirring for 2.5 hour and the reaction mixture was diluted with ethyl acetate. The organic layer was washed with sat. aq. $Na_2S_2O_3$, brine, dried over $MgSO_4$, filtrated and concentrated *in vacuo*. Column chromatography (30% -> 70% Et_2O in pentane) yielded compound **93** (0.43 g, 0.515 mmol) in quantitative yield. ^1H NMR (400 MHz, CDCl3) δ 8.34 – 7.19 (m, 27H, H-Aromatic), 5.68 (s, 1H, H-Benzylidene), 5.53 (dd, J = 3.4, 1.7 Hz, 1H, H-2), 5.41 (dd, J = 4.1, 1.8 Hz, 1H, H-1 α), 4.95 – 4.65 (m, 7H, CH_2-Bn/Nap, H-1'), 4.66 – 4.63 (m, 1H, 1-OH), 4.47 – 4.36 (m, 2H, H-3, H-6'), 4.27 (t, J = 9.6 Hz, 1H, H-4'), 4.23 – 4.10 (m, 1H, H-5), 3.95 (t, J = 10.3 Hz, 1H, H-6'), 3.83 (d, J = 3.1 Hz, 1H, H-2'), 3.76 (t, J = 9.5 Hz, 1H, H-4), 3.49 (dd, J = 9.9, 3.0 Hz, 1H, H-3'), 3.42 – 3.34 (m, 1H, H-5'), 1.46 (d, J = 6.2 Hz, 3H, H-6). ^{13}C NMR (101 MHz, $CDCl_3$) δ 165.9, 138.6, 138.3, 137.8, 135.9, 133.4, 133.2, 133.0, 130.3, 129.9, 128.9, 128.6, 128.6, 128.5, 128.4, 128.4, 128.4, 128.3, 128.3, 128.2, 128.2, 128.1, 128.0, 128.0, 127.9, 127.8, 127.5, 127.4, 127.2, 126.5, 126.3, 126.3, 126.2, 126.1, 125.9, 125.9, 125.6 (C-Aromatic), 103.7 (C-1'), 101.5 (C-Benzylidene), 91.7 (C-1), 81.9 (C-4), 78.4 (C-3'/C-4'), 78.3 (C-3'/C-4'), 77.5 (C-3), 75.9 (C-2'), 75.3 (CH_2-Bn/Nap), 74.4 (C-2), 74.3 (CH_2-Bn/Nap), 72.4 (CH_2-Bn/Nap), 68.6 (C-6'), 67.7 (C-5/C-5'), 67.6 (C-5\C-5'), 18.3 (C-6). HRMS $[M+Na]^+$ calculated 861.3245, found 861.3243.

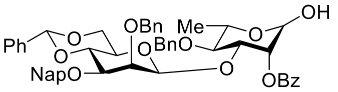

2-*O*-benzyl-4,6-*O*-benzylidene-3-*O*-naphthyl-β-D-mannose-(1->3)-2-*O*-benzoyl-4-*O*-benzyl-1-*O*-trichloroimidate-α-L-rhamnose (**94**). Compound **93** (0.42 g, 0.5 mmol) was dissolved in dry DCM (2.5 mL, 0.2 M) under a N_2 atmosphere and trichloroacetonitrile (0.2 mL, 2 mmol, 4 eq.), DBU (0.015 mL, 0.1 mmol, 0.2 eq.) were added. After stirring for 3.5 hours TLC showed full conversion and the reaction mixture was concentrated *in vacuo*. Column chromatography (10% -> 40% Et_2O in pentane) yielded compound **94** (0.45 g, 0.46 mmol) in 92%. ^1H NMR (400 MHz, CDCl3) δ 8.72 (s, 1H, Aromatic), 8.13 – 8.10 (m, 2H, Aromatic), 7.81 – 7.15 (m, 24H, Aromatic), 6.41 (d, *J* = 1.9 Hz, 1H, H-1), 5.57 (m, *J* = 2.0 Hz, 2H, H-2, Benzylidene), 4.80 – 4.54 (m, 8H, H-1´, CH_2-Bn/Nap, N-H), 4.29 (dd, *J* = 9.7, 3.3 Hz, 1H, H-3), 4.20 – 4.13 (m, 2H, H-4´, H-6´), 4.01 (ddd, *J* = 12.4, 7.8, 4.8 Hz, 1H, H-5), 3.85 (d, *J* = 10.3 Hz, 1H, H-6´), 3.78 – 3.70 34 (m, 2H, H-4, H-2´), 3.47 (dd, *J* = 9.9, 3.1 Hz, 1H, H-3´), 3.28 (dt, *J* = 9.8, 4.9 Hz, 1H, H-5´), 1.41 (d, *J* = 6.2 Hz, 3H, H-6). ^{13}C NMR (101 MHz, CDCl3) δ 165.4 (C=O), 160.0 (C=N), 138.5, 137.8, 137.6, 135.8, 133.5, 133.3, 133.0, 130.0, 129.8, 129.0, 128.6, 128.6, 128.3, 128.1, 128.1, 128.1, 128.0, 128.0, 127.8, 127.7, 127.5, 126.3, 126.2, 125.9, 125.6 (Aromatic), 103.8 (Benzylidene), 101.5 (C-1'), 94.7 (C-1), 80.7

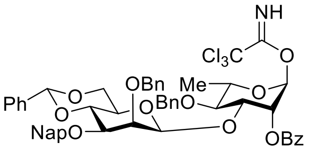

(C-2'), 78.4 (C-4'), 77.9 (C-3'), 77.4 (C-3), 75.8 (C-4), 75.8 (CH_2-Bn/Nap), 74.3 (CH_2-Bn/Nap), 72.4 (CH_2-Bn/Nap), 72.3 (C-2), 70.9 (C-5), 68.5 (C-6'), 67.8 (C-5'), 18.3 (C-6).

2-*O*-benzyl-4,6-*O*-benzylidene-3-*O*-naphthyl-β-D-mannose-(1->3)-2-*O*-benzoyl-4-*O*-benzyl-α-L-rhamnose-(1->3)-2-*O*-benzyl-6-*O*-t-butyldiphenylsilyl-3-*O*-t-butyldimethylsilyl-β-D-glucose-cyclophellitol (**95**). Donor **94** (0.18 g, 0.18 mmol, 1.8 eq.) and acceptor **7** (0.06 g, 0.1 mmol) were co-evaporated with toluene 3x and dissolved in DCM (0.9 mL, 0.11 M). 3 Å MS were added and the mixture was stirred for 60 minutes before cooling to -40 °C. TMSOTf (0.1 mL, 0.1 M in dry DCM) was added and TLC showed complete conversion after 1h45min. The reaction was quenched with Et_3N, diluted with DCM, washed with sat. aq. $NaHCO_3$, brine, dried over $MgSO_4$, filtrated and concentrated *in vacuo*. Column chromatography (10% -> 20% Et_2O in pentane) and subsequent size exclusion chromatography yielded compound **95** (0.093 g, 0.065 mmol) in 65%. ^{1}H NMR (500 MHz, $CDCl_3$) δ 8.11 – 6.97 (m, 42H, H-Aromatic), 5.68 (d, *J* = 1.6 Hz, 1H, H-1'), 5.55 (s, 1H, H-Benzylidene), 5.43 (dd, *J* = 3.7, 1.6 Hz, 1H, H-2'), 4.82 – 4.57 (m, 7H, CH_2-Bn/Nap), 4.56 – 4.54 (m, 1H, H-1"), 4.53 – 4.46 (m, 1H, CH_2-Bn/Nap), 4.34 (dd, *J* = 9.7, 3.6 Hz, 1H, H-3'), 4.23 (dt, *J* = 10.5, 5.2 Hz, 1H, H-6"), 4.09 (t, *J* = 9.6 Hz, 1H, H-4"), 4.06 – 4.01 (m, 1H, H-5'), 3.97 (dd, *J* = 9.2, 4.5 Hz, 1H, H-6), 3.89 (d, *J* = 7.7 Hz, 1H, H-2), 3.85 (t, *J* = 10.3 Hz, 1H, H-6"), 3.75 – 3.69 (m, 2H, H-3, H-7), 3.66 – 3.55 (m, 3H, H-6, H-4', H-2"), 3.35 (t, *J* = 9.5 Hz, 1H, H-4), 3.26 – 3.17 (m, 2H, H-1, H-3"), 3.09 (td, *J* = 9.7, 4.8 Hz, 1H, H-5"), 2.32 – 2.22 (m, 1H, H-5), 1.30 (d, *J* = 6.2 Hz, 3H, H-6), 1.07 (s, 9H, TBDMS/TBS), 0.67 (s, 9H, TBDMS/TBS), 0.07 (s, 3H, TBS), -0.32 (s, 3H, TBS). ^{13}C NMR (126 MHz, $CDCl_3$) δ 165.7, 138.7, 137.8, 137.5, 136.1, 135.7, 133.6, 133.5, 133.4, 133.1, 133.0, 130.4, 129.9, 129.8, 129.0, 128.5, 128.5, 128.4, 128.3, 128.3, 128.3, 128.2, 128.1, 128.1, 128.0, 128.0, 127.9, 127.9, 127.8, 127.8, 127.6, 127.5, 126.7, 126.3, 126.2, 126.1, 126.0, 125.9, 125.5 (C-Aromatic), 103.5 (C-1"), 101.5 (C-Benzylidene), 96.4 (C-1'), 82.2 (C-4'/C-2"), 81.0 (C-2), 78.5 (C-4"/C-3"), 78.4 (C-4"/C-3"), 76.5 (C-3'), 75.4 (C-3), 75.1 (CH_2-Bn/Nap), 74.2 (CH_2-Bn/Nap), 73.6 (C-2'), 72.6 (CH_2-Bn/Nap), 72.3 (CH_2-Bn/Nap), 68.7 (C-6"), 67.7 (C-5'/C-5"), 67.6 (C-5'/C-5"), 67.1 (C-4), 63.7 (C-6), 54.8 (C-7), 54.3 (C-1), 46.2 (C-5), 27.0, 26.2, 19.4 (TBS/TBDMS), 18.2 (C-6'), 18.0 (TBS/TBDMS), -2.6 (TBS), -4.1(TBS). HRMS $[M+Na]^+$ calculated 1464.640, found 1464.634.

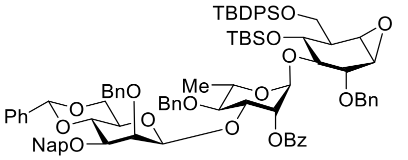

β-D-mannose-(1->3)-α-L-rhamnose-(1->3)-β-D-glucose-cyclophellitol (**18**). Compound **95** (0.026 g, 0.018 mmol) was dissolved in DCM:MeOH (1 mL, 0.018 M, 1:1) and NaOMe (0.03 mL, 4.37 M, 8 eq.) was added. After stirring for 6 hours TLC showed almost full conversion and additional NaOMe (0.015 mL, 4.37 M, 4 eq.) was added. After stirring for one additional hour, the mixture was diluted with DCM and washed with H_2O. The organic layer was dried over $MgSO_4$, filtrated and concentrated *in vacuo*. The crude product was then taken up in THF (0.6 mL, 0.03 M) and TBAF (0.07 mL, 1 M in THF, 4 eq.) was added. After stirring for 16 hours TLC showed full conversion and the reaction mixture was diluted with DCM, and washed with H_2O, sat. aq. $NaHCO_3$, brine, dried over $MgSO_4$, filtrated and concentrated *in vacuo*. The partially deprotected compound **95** was used without further purification. Na (21 mg, 0.9 mmol, 50 eq.) was dissolved in freshly condensed NH_3 (10-15 mL) at -60 °C under a nitrogen atmosphere. Partially deprotected compound **95** was co-evaporated 3 times with toluene and dissolved in dry THF (0.36 mL, 0.05M) and t-BuOH (0.09 mL, 0.9 mmol, 50 eq.) was added. The solution was then added dropwise to the NH_3 solution at -60 °C. After stirring for 1.5 hours the reaction was quenched with AcOH. Size exclusion chromatography using HOAc and a 2nd one with NH_4OAc yield pure compound **18** (4.5 mg, 9.3 µmol) in 52%. ^{1}H NMR (400 MHz, D2O) δ 5.10 (d, J = 1.8 Hz, 1H, H-1'), 4.75 (d, J = 1.1 Hz, 1H, H-1"), 4.13 (dd, J = 3.3, 1.8 Hz, 1H, H-2'), 3.98 (dd, J = 3.2, 0.9 Hz, 1H, H-2"), 3.92 – 3.78 (m, 6H, H-6, H-6", H-3', H-5', H-2), 3.70 (dd, J = 11.3, 7.2 Hz, 1H, H-6), 3.61 (dd, J = 12.3, 6.5 Hz, 1H, H-6"), 3.52 (dd, J = 9.6, 3.2 Hz, 1H, H-3"), 3.48 – 3.41 (m, 3H, H-1/H-7, H-4', H-4"), 3.38 (dd, J = 10.1, 7.9 Hz, 1H, H-3), 3.28 – 3.25 (m, 1H, H-5"), 3.25 – 3.20 (m, 1H, H-4), 3.10 (d, J = 3.9 Hz, 1H, H-1/H-7), 2.03 (dddd, J = 8.9, 7.2, 3.6, 1.6 Hz, 1H, H-5), 1.14 (d, J = 6.2 Hz, 3H, H-6'). ^{13}C NMR (101 MHz, D_2O) δ 101.3 (C-1"), 99.7 (C-1'), 82.0 (C-3), 79.1 (C-2/C-3'/C-5'), 76.2 (C-5"), 72.9 (C-3"), 71.4 (C-2/C-3'/C-5'), 71.2 (H-4'/H-4"), 70.6 (C-2'/C-2"), 70.5 (C-2'/C-2"), 68.9 (C-2/C-3'/C-5') , 66.8 (C-4'/C-4"), 64.9 (C-4), 61.1 (C-6"), 60.3 (C-6), 56.2 (C-1/C-7), 56.0 (C-1/C-7), 43.3 (C-5), 16.6 (C-6'). HRMS $[M+NH_4]^+$ calculated 502.2130, found 502.2135.

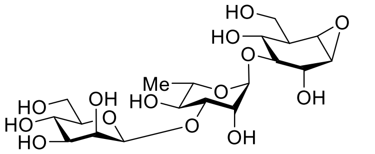

Synthesis of 19

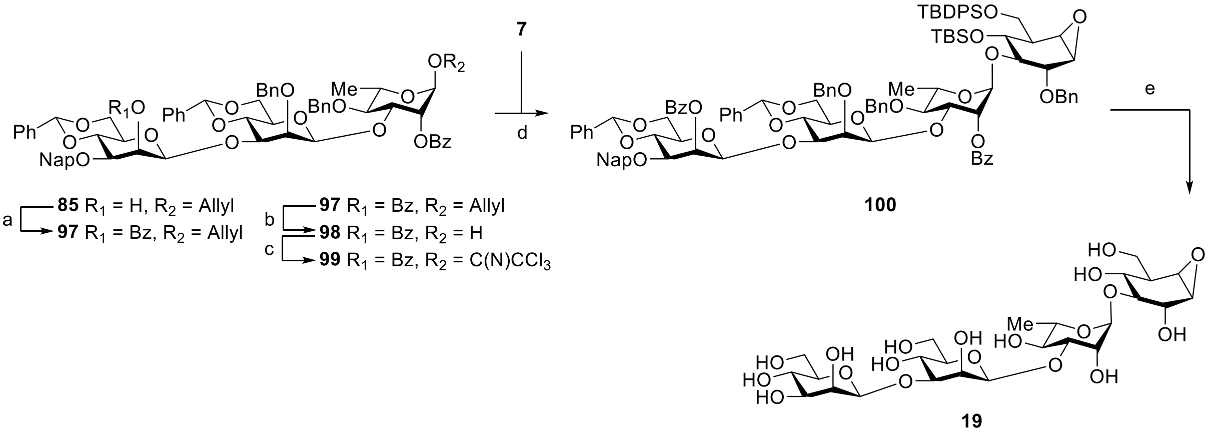

Scheme 7. a) i) HF-pyridine, pyridine, 68%; ii) BzCl, pyridine, 0 °C, 96%; b) i) $[Ir(PMePh_2)_2COD]^+$ PF_6^-, H_2, THF; ii) NIS, $NaHCO_3$, H_2O, 37%, with 46% starting material; c) trichloroacetonitrile, DBU, DCM, 80%; d) TMSOTf, DCM, 3 Å MS, -40 °C, 73%; e) i) NaOMe, DCM:MeOH, 1:1; ii) TBAF, THF; iii) Na, NH_3, t-BuOH, THF, -60 °C, 53% over 3 steps.

2-*O*-benzoyl-4,6-*O*-benzylidene-3-*O*-naphthyl-β-D-mannose-(1->3)-2-*O*-benzyl-4,6-*O*-benzylidene-β-D-mannose-(1->3)-1-*O*-allyl-2-*O*-benzoyl-4-*O*-benzyl-α-L-rhamnose (**97**). Compound **85** (0.35 g, 0.31 mmol) was dissolved in pyridine (1.55 mL, 0.2 M) and BzCl (0.07 mL, 0.62 mmol, 2 eq.) was added at 0 °C. After stirring for 2h45min the reaction was quenched with H_2O at 0 °C and dilute with Et_2O. The organic layer was then washed with 1 M HCl, sat. aq. $NaHCO_3$, brine, dried over $MgSO_4$, filtrated and concentrated *in vacuo*. Column chromatography (10% -> 30% EtOAc in pentane) yielded compound **97** (0.36 g, 0.3 mmol) in 96%. ^{1}H NMR (500 MHz, $CDCl_3$) δ 8.19 – 6.86 (m, 36H, H-Aromatic), 5.91 (dddd, *J* = 17.2, 10.4, 6.0, 5.2 Hz, 1H, Allyl), 5.75 (dd, *J* = 3.5, 1.2 Hz, 1H, H-2”), 5.60 (s, 1H, Benzylidene), 5.42 (dd, *J* = 3.5, 1.8 Hz, 1H, H-2), 5.37 (d, *J* = 4.7 Hz, 1H, H-Benzylidene), 5.33 – 5.17 (m, 2H, CH_2-Allyl), 4.95 (d, *J* = 1.7 Hz, 1H, H-1), 4.95 – 4.81 (m, 2H, CH_2-Bn/Nap), 4.75 – 4.65 (m, 3H, H-1’, CH_2-Bn/Nap), 4.57 – 4.46 (m, 2H, CH_2-Bn/Nap), 4.45 (d, *J* = 1.3 Hz, 1H, H-1”), 4.28 – 4.22 (m, 2H, H-3, H-6’), 4.19 – 4.12 (m, 3H, H-6” (2H), CH_2-Allyl (1H)), 4.12 – 4.08 (m, 1H, H-4”), 4.02 (dt, *J* = 6.0, 1.4 Hz, 1H, CH_2-Allyl (1H)), 4.01 – 3.94 (m, 1H, H-4’), 3.90 – 3.82 (m, 2H, H-5, H-6”), 3.80 – 3.63 (m, 5H, H-4, H-2', H-3', H-6', H-3"), 3.23 (td, *J* = 9.7, 4.9 Hz, 1H, H-5’), 3.16 (td, *J* = 9.7, 4.9 Hz, 1H, H-5”), 1.38 (d, *J* = 14.9 Hz, 3H, H-6). ^{13}C NMR (126 MHz, $CDCl_3$) δ 166.1, 165.7, 138.4, 138.3, 137.6, 137.5, 135.4, 133.7, 133.3, 133.1, 133.0, 133.0, 132.5, 130.2, 130.1, 130.0, 129.8, 129.8, 129.3, 129.0, 128.7, 128.6, 128.4, 128.4, 128.2, 128.1, 128.0, 127.9, 127.9, 127.8, 127.8, 127.8, 127.6, 127.5, 127.3, 127.2, 126.3, 126.2, 126.0, 125.9, 125.5 (C-Aromatic), 117.7 (CH_2-Allyl), 103.2 (C-1’), 101.6 (C-Benzylidene), 101.0 (C-Benzylidene), 98.0 (C-1”), 96.4 (C-1), 81.4 (C-4/C-2'/C-3'/C-3"), 78.3 (C-4”), 78.2 (C-3), 77.2 (C-4’), 77.0 (C-4/C-2'/C-3'/C-3"), 75.9 (C-4/C-2'/C-3'/C-3"), 75.4 (C-4/C-2'/C-3'/C-3"), 75.3 (CH_2-Bn/Nap), 74.5 (CH_2-Bn/Nap), 73.4 (C-2), 71.5 (CH_2-Bn/Nap), 69.5 (C-2”), 68.7 (C-6’), 68.4 (C-6”/CH_2-Allyl), 68.3 (C-6”/CH_2-Allyl), 67.7 (C-5), 67.5 (C-5’), 67.4 (C-5”), 18.1 (C-6). HRMS $[M+Na]^+$ calculated 1255.466, found 1255.468.

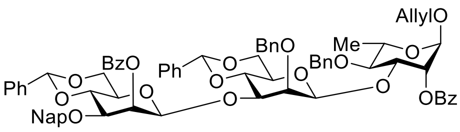

2-*O*-benzoyl-4,6-*O*-benzylidene-3-*O*-naphthyl-β-D-mannose-(1->3)-2-*O*-benzyl-4,6-*O*-benzylidene-β-D-mannose-(1->3)-2-*O*-benzoyl-4-*O*-benzyl-α-L-rhamnose (**98**). Compound **97** (0.33 g, 0.27 mmol) was dissolved in freshly distilled THF (1 mL, 0.27 M) and argon was bubbled through under sonication for 10 min. $[Ir(PMePh_2)_2COD]^+$ PF_6^- (0.011 g, 0.013 mmol, 0.05 eq.) was dissolved in freshly distilled THF (1.5 mL, 0.009 M) and argon was bubbled through under sonication for 12 min. Then the catalyst was activated by bubbling through H_2 for 30 sec/until all the red color had disappeared, subsequently N_2 was bubbled through for 20-30 seconds to remove excess H_2. Then the degassed solution of compound **97** was added. Subsequently, 1 mL of THF was added to the flask with remaining compound **97** and flushed with argon under sonication, this was then added as well to the catalyst solution. After stirring for 3 hours TLC indicated incomplete conversion and the reaction mixture was evaporated. NMR showed 50% conversion. The compound was

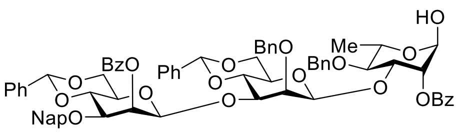

redissolved in THF (3.5 mL, 0.05M) and H_2O (1 mL). Subsequently, $NaHCO_3$ (0.034 g, 0.41 mmol, 1.5 eq.) and NIS (0.091 g, 0.41 mmol, 1.5 eq.) were added. TLC showed full conversion after stirring for 3.5 hours and the reaction mixture was diluted with ethyl acetate. The organic layer was washed with sat. aq. $Na_2S_2O_3$, brine, dried over $MgSO_4$, filtrated and concentrated *in vacuo*. Column chromatography (10% -> 40% EtOAc in pentane) yielded compound **98** (0.12 g, 0.1 mmol) in 37% yield (83% based on recovered starting material). HRMS $[M+Na]^+$ calculated 1215.435, found 1215.434.

2-*O*-benzoyl-4,6-*O*-benzylidene-3-*O*-naphthyl-β-D-mannose-(1->3)-2-*O*-benzyl-4,6-*O*-benzylidene-β-D-mannose-(1->3)-1-*O*-trichloroimidate-2-*O*-benzoyl-4-*O*-benzyl-α-L-rhamnose (**99**).

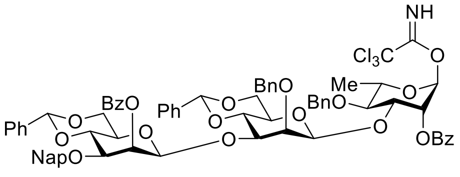

Compound **98** (0.12 g, 0.1 mmol) was dissolved in dry DCM (1 mL, 0.1 M) under a N_2 atmosphere and trichloroacetonitrile (0.04 mL, 0.4 mmol, 4 eq.), DBU (0.1 mL, 0.2 M, 0.2 eq.) were added. After stirring for 3.5 hours TLC showed full conversion and the reaction mixture was concentrated *in vacuo*. Column chromatography (10% -> 50% Et_2O in pentane) yielded compound **99** (0.11 g, 0.0.08 mmol) in 80%.

2-*O*-benzoyl-4,6-*O*-benzylidene-3-*O*-naphthyl-β-D-mannose-(1->3)-2-*O*-benzyl-4,6-*O*-benzylidene-β-D-mannose-(1->3)-2-*O*-benzoyl-4-*O*-benzyl-α-L-rhamnose-(1->3)-2-*O*-benzyl-6-*O*-t-butyldiphenylsilyl-3-*O*-t-butyldimethylsilyl-β-D-glucose-cyclophellitol (**100**).

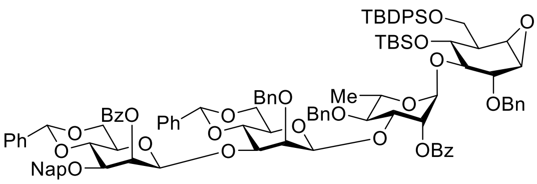

Donor **99** (0.11 g, 0.08 mmol, 1 eq.) and acceptor **7** (0.07 g, 0.11 mmol, 1.4 eq.) were coevaporated with toluene 3x and dissolved in dry DCM (0.7 mL, 0.11 M). 3 Å MS were added and the mixture was stirred for 60 minutes before cooling to -40 °C. TMSOTf (0.1 mL, 0.08 M, 0.1 eq.) was added, and TLC showed complete conversion after 2h. The reaction was quenched with Et_3N, diluted with DCM, washed with sat. aq. $NaHCO_3$, brine, dried over $MgSO_4$, filtrated and concentrated *in vacuo*. Size exclusion chromatography yielded compound **100** (0.061 mmol, 0.11 g) in 76% and impure acceptor, column chromatography (4% -> 10% Et_2O in pentane) yielded acceptor **7** (0.036 g, 0.058 mmol) slightly impure in 73%. ^1H NMR (600 MHz, $CDCl_3$) δ 8.17 – 6.79 (m, 52H, H-Aromatic), 5.69 (d, *J* = 3.4 Hz, 1H, H-2’’’), 5.66 (d, *J* = 1.6 Hz, 1H, H-1’), 5.60 (s, 1H, H-Benzylidene), 5.41 (dd, *J* = 3.7, 1.6 Hz, 1H, H-2’), 5.38 (s, 1H, H-Benzylidene), 4.94 (d, *J* = 13.0 Hz, 1H, CH_2-Bn/Nap), 4.84 (d, *J* = 13.0 Hz, 1H, CH_2-Bn/Nap), 4.78 (d, *J* = 10.9 Hz, 1H, CH_2-Bn/Nap), 4.74 (d, *J* = 11.8 Hz, 1H, CH_2-Bn/Nap), 4.70 (d, *J* = 10.8 Hz, 1H, CH_2-Bn/Nap), 4.59 (d, *J* = 11.8 Hz, 1H, CH_2-Bn/Nap), 4.57 (s, 1H, H-1”), 4.50 (d, *J* = 12.2 Hz, 1H, CH_2-Bn/Nap), 4.43 (d, *J* = 12.0 Hz, 1H, CH_2-Bn/Nap), 4.35 (dd, *J* = 9.5, 3.7 Hz, 1H, H-3’), 4.28 (s, 1H, H-1’’’), 4.20 (dq, *J* = 9.5, 4.8 Hz, 1H, H-6”), 4.09 (dd, *J* = 10.5, 4.7 Hz, 1H, H-6’’’), 4.08 – 4.01 (m, 2H, H-5’, H-4’’’), 3.97 (dd, *J* = 9.2, 4.5 Hz, 1H, H-6), 3.94 – 3.87 (m, 2H, h-2, H-4”), 3.82 (t, *J* = 10.2 Hz, 1H, H-6’’’), 3.77 – 3.69 (m, 3H, H-3, H-1/H-7, H-6”), 3.66 (dd, *J* = 9.7, 3.4 Hz, 1H, H-3’’’), 3.65 – 3.60 (m, 1H, H-6), 3.60 – 3.54 (m, 2H, H-4’, H-2”), 3.49 (dd, *J* = 10.0, 3.1 Hz, 1H, H-3’’’), 3.35 (t, *J* = 9.6 Hz, 1H, H-4), 3.22 (d, *J* = 3.9 Hz, 1H, H-1/H-7), 3.11 (td, *J* = 9.7, 4.8 Hz, 1H, H-5’’’), 3.06 (td, *J* = 9.6, 4.8 Hz, 1H,H-5”), 2.27 (td, *J* = 9.9, 3.7 Hz, 1H, H-5), 1.30 (d, *J* = 6.2 Hz, 3H, H-6’), 1.07 (s, 9H, TBS/TBDPS), 0.68 (s, 9H, TBS/TBDPS), 0.07 (s, 3H, TBS), -0.32 (s, 3H, TBS). ^{13}C NMR (151 MHz, $CDCl_3$) δ 166.2, 165.7, 138.8, 138.4, 137.5, 137.5, 135.7, 135.7, 135.4, 133.6, 133.4, 133.4, 133.1, 133.1, 130.3, 130.1, 130.1, 130.1, 129.9, 129.8, 129.8, 129.1, 128.8, 128.5, 128.5, 128.5, 128.4, 128.4, 128.4, 128.3, 128.2, 128.1, 128.1, 128.0, 128.0, 127.9, 127.9, 127.7, 127.6, 127.3, 126.7, 126.5, 126.3, 126.1, 126.0, 125.9, 125.6 (C-Aromatic), 103.1 (C-1”), 101.7 (Benzylidene), 101.1 (Benzylidene), 98.0 (C-1’’’), 96.4 (C-1’), 82.0 (C-4’/C-2”), 81.0 (C-2/C-4”), 78.3 (C-5’/C-4’’’), 77.4 (C-3), 77.2 (C-2/C-4”/C-3”), 77.2 (C-2/C-4”/C-3”), 76.5 (C-3’), 75.4 (C-3’’’), 75.3 (C-4’/C-2”), 75.0 (CH_2-Bn/Nap), 74.3 (CH_2-Bn/Nap), 73.4 (C-2’), 72.6 (CH_2-Bn/Nap), 71.5 (CH_2-Bn/Nap), 69.6 (C-2’’’), 68.7 (C-6’’’), 68.5 (C-6”), 67.6 (C-5”), 67.5 (C-5’/C-4’’’), 67.4 (C-5’’’), 67.1 (C-4), 63.7 (C-6), 54.8 (C-1/C-7), 54.2 (C-1/C-7), 46.1 (C-5), 27.0, 26.2 (CH_3 TBS/TBDS), 19.4 (t-Bu TBS/TBDPS), 18.1 (C-6’), 18,0 (t-Bu TBS/TBDPS), -2.7 (TBS), -4.1 (TBS).

β-D-mannose-(1->3)-β-D-mannose-(1->3)-α-L-rhamnose-(1->3)-β-D-glucose-cyclophellitol (**19**).

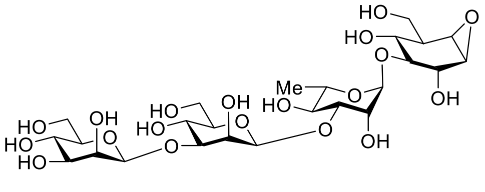

Compound **100** (0.054 g, 0.03 mmol) was dissolved in DCM:MeOH (0.86 mL, 0.035 M, 1:072) and NaOMe (0.14 mL, 0.44 M, final concentration 0.06M) was added. After stirring for 4.5 hours the reaction still wasn’t finished and additional NaOMe (0.04 mL, 0.18 mmol, 6 eq.) was added. After stirring for an additional 2 hour, TLC showed

incomplete conversion and additional MeOH (0.2 mL) was added. After stirring for an additional 17.5 hour, TLC showed complete conversion and the reaction mixture was diluted with DCM, washed with H_2O, brine, dried over $MgSO_4$, filtrated and concentrated *in vacuo*. The crude product was then taken up in THF (0.6 mL, 0.05M) and TBAF (0.12 mL, 1 M, 4 eq.) was added. After stirring for 20 hours TLC indicated full conversion and the reaction was diluted with DCM, washed with H_2O, sat. aq. $NaHCO_3$, brine, dried over $MgSO_4$, filtrated and concentrated *in vacuo*. Column chromatography (50% -> 80% ethyl acetate in pentane) yielded partially deprotected compound, the product was used immediately for the next reaction. Na (0.041 g, 1.8 mmol, 60 eq.) was dissolved in freshly condensed ammonia (10~15 mL) at -60 °C under N_2. A solution of partially deprotected compound **100** (0.037 g, 0.03 mmol) was dissolved in dry THF (0.6 mL, 0.05M) and t-BuOH (0.17 mL, 1.8 mmol, 60 eq.) was added. The compound was added dropwise to the Na-ammonia solution at -60 °C. After stirring for 1 hour the reaction was quenched with AcOH. Size exclusion chromatography yielded compound **19** (0.0104 g, 0.016 mmol) in 53.4%. ^{1}H NMR (500 MHz, D_2O) δ 5.13 (d, *J* = 1.8 Hz, 1H, H-1'), 4.77 (s, 1H, H-1#), 4.75 (s, 1H, H-1*), 4.21 (d, *J* = 3.2 Hz, 1H, H-2#), 4.15 (dd, *J* = 3.3, 1.8 Hz, 1H, H-2'), 3.98 – 3.95 (m, 1H, H-2*), 3.93 – 3.89 (m, 1H, H-5'), 3.89 – 3.80 (m, 6H, H-2, H-6, H-3', H-6*, H-3#, H-6#), 3.72 (dd, *J* = 11.3, 7.2 Hz, 1H, H-6), 3.68 – 3.59 (m, 3H, H-4#, H-6#, H-6*), 3.57 (dd, *J* = 9.6, 3.3 Hz, 1H, H-3*), 3.51 – 3.44 (m, 3H, H-7, H-4', H-4*), 3.40 (dd, *J* = 10.1, 7.8 Hz, 1H, H-3), 3.34 – 3.27 (m, 2H, H-5#, H-5*), 3.25 (t, *J* = 10.0 Hz, 1H, H-4), 3.12 (d, *J* = 3.8 Hz, 1H, H-1), 2.10 – 2.02 (m, 1H, H-5), 1.16 (d, *J* = 6.3 Hz, 3H, H-6'). ^{13}C NMR (126 MHz, D_2O) δ 101.2 (C-1#/C-1*), 99.6 (C-1'), 96.7 (C-1#/C-1*), 82.0 (C-3), 79.1 (C-2/C3'/C-3#), 78.9 (C-2/C3'/C-3#), 76.3 (C-5#/C-5*), 75.8 (C-5#/C-5*), 72.8 (C-3*), 71.4 (C-2/C3'/C-3#), 71.1 (C-4'/C-4*), 70.7 (C-2*), 70.4 (C-2'), 68.8 (C-5'), 67.6 (C-2#), 66.8 (C-4'/C-4*), 65.2 (C-4'), 64.9 (C-4), 61.0 (C-6#, C-6*), 60.3 (C-6), 56.2 (C-7), 56.0 (C-1), 43.2 (C-5) , 16.5 (C-6'). HRMS $[M+Na]^+$ calculated 669.2213, found 669.2218.

Synthesis of 20

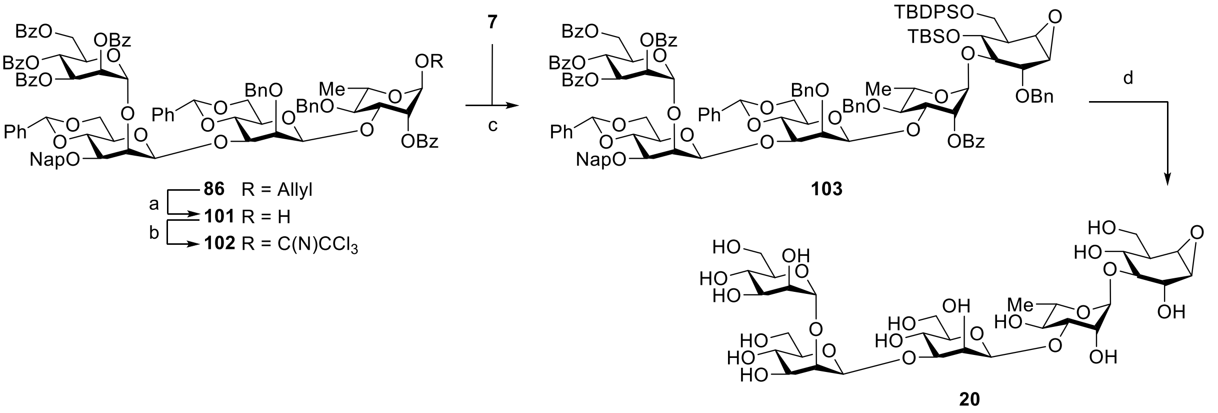

Scheme 8. a) i) $[Ir(PMePh_2)_2COD]^+$ PF_6^-, H_2, THF; ii) NIS, $NaHCO_3$, H_2O, 61%; b) trichloroacetonitrile, DBU, DCM, 85%; d) TMSOTf, DCM, 3 Å MS, -40 °C, 54%; e) i) NaOMe, DCM:MeOH, 1:1; ii) TBAF, THF; iii) Na, NH_3, t-BuOH, THF, -60 °C, 73% over 3 steps.

2,3,4,6-*O*-benzoyl-α-D-mannose-(1->2)-4,6-*O*-benzylidene-3-*O*-naphthyl-β-D-mannose-(1->3)-2-*O*-benzyl-4,6-*O*-benzylidene-β-D-mannose-(1->3)-2-*O*-benzoyl-4-*O*-benzyl-α-L-rhamnose (101). Compound **86** (0.44 g, 0.26 mmol) was dissolved in freshly distilled THF (2 mL, 0.13 M) and argon was

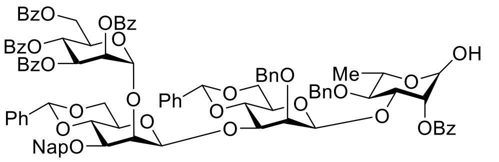

bubbled through under sonication for 10 min. $[Ir(PMePh_2)_2COD]^+PF_6^-$ (0.011 g, 0.013 mmol, 0.05 eq.) was dissolved in freshly distilled THF (1.5 mL, 0.009 M) and argon was bubbled through under sonication for 13 min. Then the catalyst was activated by bubbling through H_2 for 30 sec at which time all the red color had disappeared, subsequently N_2 was bubbled through for 20-30 seconds to remove excess H_2, or until the solution become slightly red again. Then the degassed solution of compound **86** was added. After stirring for 1h45min TLC indicated complete conversion, and the reaction mixture was diluted with THF to 0.05M and 1 mL of H_2O was added. Subsequently, $NaHCO_3$ (0.032 g, 0.39 mmol, 1.5 eq.) and NIS (0.087 g, 0.39 mmol, 1.5 eq.) were added. TLC showed full conversion after stirring for 4 hours and the reaction mixture was diluted with ethyl acetate. The organic layer was washed with sat. aq. $Na_2S_2O_3$, brine, dried over $MgSO_4$, filtrated and concentrated *in vacuo*. Column chromatography (10% -> 40% EtOAc in pentane) yielded compound **101** (0.26 g, 0.16 mmol) in 61% yield. ^1H NMR (500 MHz, $CDCl_3$) δ 8.27 – 6.85 (m, 63H), 6.20 (t, *J* = 10.3 Hz, 1H), 6.12 – 6.03 (m, 1H), 5.93 (dd, *J* = 10.3, 3.2 Hz, 1H), 5.78 (s, 1H), 5.54 – 5.49 (m, 1H), 5.47 (s, 1H), 5.44 (dd, *J* = 3.2, 1.7 Hz, 1H), 5.36 – 5.29 (m, 1H), 5.05 (d, *J* = 12.5 Hz, 1H), 4.94 – 4.84 (m, 2H), 4.79 (dd, *J* = 12.2, 7.5 Hz, 2H), 4.74 (s, 1H), 4.70 – 4.59 (m, 3H), 4.37 – 4.30 (m, 2H), 4.30 – 4.23 (m, 2H), 4.19 – 4.13 (m, 2H), 4.12 – 4.05 (m, 1H), 4.03 – 3.96 (m, 3H), 3.93 (t, *J* = 9.7 Hz, 1H), 3.76 – 3.70 (m, 2H), 3.70 – 3.63 (m, 2H), 3.54 – 3.47 (m, 1H), 3.27 (td, *J* = 9.5, 5.0 Hz, 1H), 3.13 (td, *J* = 9.6, 5.0 Hz, 1H), 1.35 (d, *J* = 6.1 Hz, 3H). ^{13}C NMR (126 MHz, $CDCl_3$) δ 178.0, 171.4, 166.9, 166.1, 165.8, 165.7, 165.5, 165.2, 138.4, 138.0, 137.8, 137.6, 135.7, 133.3, 133.3, 133.2, 133.1, 133.0, 133.0, 132.9, 130.5, 130.1, 129.9, 129.9, 129.9, 129.7, 129.6, 129.4, 129.4, 129.2, 128.7, 128.7, 128.6, 128.6, 128.6, 128.5, 128.4, 128.4, 128.3, 128.3, 128.3, 128.2, 128.2, 128.1, 127.9, 127.8, 127.7, 127.7, 126.7, 126.6, 126.3, 126.1, 126.1, 126.0, 125.9, 125.8, 104.2, 101.7, 101.7, 101.4, 100.8, 99.4, 97.0, 96.6, 93.8, 91.8, 82.0, 81.8, 81.5, 79.9, 79.3, 78.3, 77.6, 77.5, 77.4, 77.3, 77.0, 76.5, 76.2, 75.9, 75.4, 75.3, 75.2, 75.0, 74.9, 74.7, 74.5, 74.2, 74.2, 73.7, 73.2, 73.1, 72.2, 72.0, 70.7, 70.6, 70.5, 70.0, 69.6, 69.2, 68.7, 68.5, 68.3, 68.1, 67.7, 67.6, 67.0, 66.5, 62.3, 60.6, 18.3, 18.3, 18.2, 14.3. HRMS $[M+Na]^+$ calculated 1690.570, found 1690.570.

2,3,4,6-*O*-benzoyl-α-D-mannose-(1->2) 4,6-*O*-benzylidene-3-*O*-naphthyl-β-D-mannose-(1->3)-2-*O*-benzyl-4,6-*O*-benzylidene-β-D-mannose-(1->3)-1-*O*-trichloroimidate-2-*O*-benzoyl-4-*O*-benzyl-α-L-rhamnose

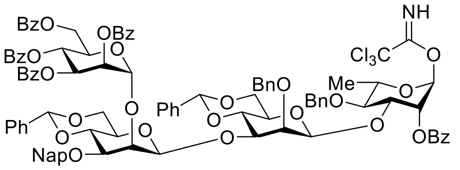

(**102**). Compound **101** (0.26 g, 0.16 mmol) was co-evaporated 3 times with toluene, dissolved in dry DCM (0.69 mL, 0.23 M) under N_2 and cooled to 0 °C. Trichloroacetonitrile (0.063 mL, 0.63 mmol, 4 eq.) and DBU (0.1 mL, 0.3 M, 0.2 eq.) were

added. After stirring for 2.5 hours TLC indicated full conversion and the reaction mixture was concentrated *in vacuo*. Column chromatography (10% -> 35% EtOAc in pentane) yielded compound **102** (0.24 g, 0.13 mmol) in 85%. ^{1}H NMR (500 MHz, $CDCl_3$) δ 8.74 (s, 1H, NH imidate), 8.17 – 6.82 (m, 52H, Aromatic), 6.41 (d, *J* = 2.0 Hz, 1H, H-1), 6.16 (t, *J* = 10.3 Hz, 1H, H-4""), 6.03 (dd, *J* = 3.3, 1.8 Hz, 1H, H-2""), 5.90 (dd, *J* = 10.4, 3.3 Hz, 1H, H-3""), 5.76 (s, 1H, Benzylidene), 5.59 (dd, *J* = 3.3, 2.0 Hz, 1H, H-2), 5.49 (d, *J* = 1.9 Hz, 1H, H-1""), 5.46 (s, 1H, Benzylidene), 5.03 (d, *J* = 12.5 Hz, 1H, CH_2-Bn/Nap), 4.86 (d, *J* = 12.6 Hz, 1H, CH_2-Bn/Nap), 4.85 – 4.79 (m, 2H, H-5"", CH_2-Bn/Nap), 4.78 – 4.69 (m, 3H, H-1*, CH_2-Bn/Nap), 4.67 – 4.59 (m, 2H, H-6"", CH_2-Bn/Nap), 4.34 – 4.27 (m, 2H, H-3, H-4#), 4.24 (dd, *J* = 10.5, 4.8 Hz, 1H, H-6#), 4.16 – 4.09 (m, 2H, H-6"", H-6*), 4.08 (s, 1H, H-1#), 4.07 – 4.03 (m, 1H, H-5), 4.00 (d, *J* = 2.8 Hz, 1H, H-2#), 3.99 – 3.94 (m, 1H, H-6#), 3.96 – 3.91 (m, 1H, H-4*), 3.82 – 3.76 (m, 2H, H-4, H-3*), 3.74 – 3.69 (m, 2H, H-2*, H-6*), 3.48 (dd, *J* = 9.7, 2.9 Hz, 1H, H-3#), 3.29 (td, *J* = 9.6, 4.8 Hz, 1H, H-5*), 3.12 (td, *J* = 9.7, 4.9 Hz, 1H H-5#), 1.43 (d, *J* = 6.1 Hz, 4H, H-6). ^{13}C NMR (126 MHz, $CDCl_3$) δ 166.1, 165.7, 165.5, 165.3, 165.1, 160.0, 138.0, 137.8, 137.7, 137.4, 135.7, 133.5, 133.3, 133.3, 133.1, 133.0, 133.0, 132.9, 130.5, 130.0, 129.9, 129.9, 129.9, 129.7, 129.7, 129.4, 129.2, 129.1, 128.9, 128.7, 128.6, 128.6, 128.5, 128.5, 128.4, 128.3, 128.3, 128.2, 128.2, 128.2, 128.2, 128.1, 127.8, 127.7, 127.6, 127.3, 126.6, 126.3, 126.2, 126.1, 125.9, 125.9, 125.8 (C-Aromatic), 104.3 (C-1*), 101.6 (Benzylidene), 100.9 (Benzylidene), 99.4 (C-1""), 96.6 (C-1#), 94.7 (C-1), 80.9 (C-2*), 79.3 (C-3/C-4#), 77.6 (C-3/C-4#, C-3#), 76.2 (C-2#, C-4*), 75.6 (CH_2-Bn/Nap), 75.2 (C-4/C-3*), 73.7 (CH_2-Bn/Nap), 73.2 (CH_2-Bn/Nap), 72.8 (C-2*), 72.3 (C-2), 70.9 (C-5), 70.6 (C-2""/C-3""), 70.5 (C-2""/C-3""), 69.1 (C-5""), 68.7 (H-6*/H-6#), 68.2 (H-6*/H-6#), 68.2 (C-5*), 67.7 (C-5#), 66.6 (C-4""), 62.3 (C-6""), 18.3 (C-6).

2,3,4,6-*O*-benzoyl-α-D-mannose-(1->2) 4,6-*O*-benzylidene-3-*O*-naphthyl-β-D-mannose-(1->3)-2-*O*-benzyl-4,6-*O*-benzylidene-β-D-mannose-(1->3)-2-*O*-benzoyl-4-*O*-benzyl-α-L-rhamnose-(1->3)-2-*O*-benzyl-6-*O*-t-butyldiphenylsilyl-3-*O*-t-butyldimethylsilyl-β-D-glucose-cyclophellitol (**103**). Donor **102** (0.19 g, 0.1 mmol, 1.3 eq.) and acceptor **7** (0.05 g, 0.08 mmol, 1 eq.) were co-evaporated with toluene 3x and dissolved in DCM (0.7 mL, 0.11 M). 3 Å MS were added and the mixture was stirred for 60 minutes before cooling to -40 °C. TMSOTf (0.1 mL, 0.08 M, 0.1 eq.) was added and TLC showed almost complete conversion after 30min. After 2.5 hours TLC still showed some starting material and additional TMSOTf (0.05 mL, 0.08M, 0.05 eq.) was added. After stirring for an additional 1-hour TLC still showed no further conversion. Thus, the reaction was quenched with Et_3N, diluted with DCM, washed with sat. aq. $NaHCO_3$, brine, dried over $MgSO_4$, filtrated and concentrated *in vacuo*. Column chromatography (10% -> 50% Et_2O in pentane) and subsequent size exclusion chromatography yielded compound **103** (0.098 g, 0.043) in 54%. ^{1}H NMR (600 MHz, $CDCl_3$) δ 8.17 – 6.86 (m, 67H, H-Aromatic), 6.19 (t, *J* = 10.3 Hz, 1H, H-4""), 6.03 (dd, *J* = 3.3, 1.8 Hz, 1H, H-2""), 5.89 (dd, *J* = 10.3, 3.2 Hz, 1H, H-3""), 5.76 (s, 1H, H-Benzylidene), 5.73 (d, *J* = 1.6 Hz, 1H, H-1'), 5.48 (d, *J* = 1.8 Hz, 1H, H-1""), 5.46 (s, 1H, H-Benzylidene), 5.44 (dd, *J* = 3.6, 1.6 Hz, 1H, H-2'), 5.04 (d, *J* = 12.5 Hz, 1H, CH_2-Bn/Nap), 4.90 – 4.84 (m, 2H, H-5"", CH_2-Bn/Nap), 4.80 (d, *J* = 10.8 Hz, 1H, CH_2-Bn/Nap), 4.79 – 4.67 (m, 4H. H-6"", CH_2-Bn/Nap), 4.64 (d, *J* = 12.4 Hz, 1H, CH_2-Bn/Nap), 4.61 (s, 1H, H-1*), 4.54 (d, *J* = 12.2 Hz, 1H, CH_2-Bn/Nap), 4.37 (dd, *J* = 9.7, 3.6 Hz, 1H, H-3'), 4.29 (t, *J* = 9.5 Hz, 1H, H-4#), 4.25 – 4.19 (m, 2H, H-6*, H-6#), 4.16 (dd, *J* = 12.5, 2.2 Hz, 1H, H-6""), 4.07 (dq, *J* = 9.7, 6.3 Hz, 1H, H-5'), 3.99 – 3.94 (m, 3H, H-6, H-63, H-2#), 3.92 (d, *J* = 7.7 Hz, 1H, H-2), 3.89 – 3.84 (m, 1H, H-4*), 3.81 (s, 1H, H-1#), 3.77 – 3.72 (m, 3H, H-3, H-7, H-6*), 3.66 – 3.57 (m, 2H, H-6, H-4'), 3.54 – 3.49 (m, 2H, H-2*, H-3*), 3.47 – 3.42 (m, 1H, H-3#), 3.36 (t, *J* = 9.6 Hz, 1H, H-4), 3.23 (d, *J* = 3.9 Hz, 1H, H-1), 3.12 (td, *J* = 9.6, 4.8 Hz, 1H, H-5*), 3.05 (td, *J* = 9.6, 4.9 Hz, 1H, H-5#), 2.27 (tdd, *J* = 9.5, 4.5, 1.4 Hz, 1H, H-5), 1.31 (d, *J* = 6.2 Hz, 3H, H-6'), 1.07 (s, 9H, TBS/TBDPS), 0.69 (s, 9H, TBS/TBDPS), 0.16 – 0.04 (m, 3H, TBS), -0.31 (s, 3H, TBS). ^{13}C NMR (151 MHz, $CDCl_3$) δ 166.1, 165.7, 165.6, 165.5, 165.1, 138.7, 137.9, 137.8, 137.5, 137.5, 137.5, 135.7, 135.7, 135.7, 135.6, 133.6, 133.4, 133.3, 133.3, 133.2, 133.0, 133.0, 132.9, 130.5, 130.1, 130.0, 129.9, 129.8, 129.8, 129.7, 129.7, 129.6, 129.4, 129.2, 129.1, 128.9, 128.9, 128.5, 128.5, 128.4, 128.4, 128.4, 128.4, 128.4, 128.3, 128.3, 128.2, 128.1, 127.9, 127.9, 127.9, 127.7, 127.7, 127.6, 126.6, 126.3, 126.1, 126.0, 125.9, 125.9 (C-Aromatic), 104.1 (C-1*), 101.6 (Benzylidene), 100.6 (Benzylidene), 99.4 (C-1""), 96.7 (C-1#), 96.3 (C-1'), 82.3 (C-4'), 81.1 (C-2), 79.3 (C-4#), 77.3 (C-3, C-3#), 76.6 (C-3', C-2#), 76.1 (C-4*), 75.4 (C-2*/C-3*), 74.8 (CH_2-Bn/Nap), 73.6 (C-2'), 73.4 (CH_2-Bn/Nap), 73.2 (CH_2-Bn/Nap), 72.6 (CH_2-Bn/Nap), 72.3 (C-2*/C-3*), 70.7 (C-3""), 70.5 (C-2""), 69.1 (C-5""), 68.7 (C-6#), 68.4 (C-6*), 68.0 (C-5*), 67.5 (C-5', C-5#), 67.0 (C-4), 66.5 (C-4""),

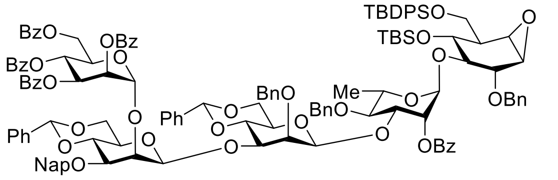

63.7 (C-6), 62.2 (C-6""), 54.8 (C-7), 54.2 (C-1), 46.1 (C-5), 27.0 (TBS/TBDPS), 26.2 (TBS/TBDPS), 19.4 (t-Bu TBS/TBDPS), 18.0 (C-6'), 18.0 (t-Bu TBS/TBDPS), -2.6 (TBS), -4.1 (TBS).

α-D-Mannose-(1->2)-β-D-mannose-(1->3)-β-D-mannose-(1->3)-α-L-rhamnose-(1->3)-β-D-glucose-cyclophellitol (**20**). Compound **103** (0.025 g, 0.011 mmol) was dissolved in THF (0.37 mL, 0.03 M) and TBAF (0.044 mL, 0.1 M, 4 eq.) was added. After stirring for 4.5 hours TLC indicated full conversion and the reaction was diluted with DCM, washed with H_2O, sat. aq. $NaHCO_3$, brine, dried over $MgSO_4$, filtrated and concentrated *in vacuo*. The crude product was take up in DCM:MeOH (0.5 mL, 0.026 M) and NaOMe (0.019 mL, 4.37 M, 6.3 eq.) was added. TLC indicated conversion to one compound and TLC-MS indicated full conversion. The reaction mixture was diluted with DCM, washed with H_2O, brine, dried over $MgSO_4$, filtrated and concentrated *in vacuo*. The crude product was used without further purification and analysis. The de-benzoylated compound (0.025 g, 0.011 mmol) was dissolved in THF (0.37 mL, 0.03 M) and TBAF (0.044 mL, 0.1 M, 4 eq.) was added. After stirring for 4.5 hours TLC indicated full conversion and the reaction was diluted with DCM, washed with H_2O, sat. aq. $NaHCO_3$, brine, dried over $MgSO_4$, filtrated and concentrated *in vacuo*. The crude product was take up in DCM:MeOH (0.5 mL, 0.026 M) and NaOMe (0.019 mL, 4.37 M, 6.3 eq.) was added. TLC indicated conversion to one compound and TLC-MS indicated full conversion. The reaction mixture was diluted with DCM, washed with H_2O, brine, dried over $MgSO_4$, filtrated and concentrated *in vacuo*. Na (0.015 g, 0.66 mmol, 60 eq.) was dissolved in freshly distilled NH_3 (10-15 mL) at -60 °C under N_2. Partially deprotected compound **103** (0.015 g, 0.011 mmol) was co-evaporated thrice with toluene and dissolved in dry THF (0.22 mL, 0.05 M) and t-BuOH (0.063 mL, 0.66 mol, 60 eq.) was added. The solution was added dropwise to the Na-NH_3 solution at -60 °C. The flask was then washed with additional THF (0.15 mL) and t-BuOH (0.05 mL) this was then added dropwise. After stirring for 1 hour the reaction had discoloured from deep blue and was quenched with AcOH. Size excl. chromatography yielded compound **20** (6.43 mg, 8 µmol) in 72%. ^{1}H NMR (600 MHz, D_2O) δ 5.13 (d, *J* = 1.8 Hz, 1H, H-1'), 5.10 (d, *J* = 1.7 Hz, 1H, H-1""), 4.79 – 4.78 (m, 1H, H-1#), 4.77 – 4.76 (m, 1H, H-1*), 4.20 (d, *J* = 3.2 Hz, 1H, H-2*), 4.16 (dd, *J* = 3.2, 1.8 Hz, 1H, H-2'), 4.14 – 4.12 (m, 1H, H-2#), 4.12 – 4.08 (m, 1H, H-5""), 4.00 (dd, *J* = 3.5, 1.7 Hz, 1H, H-2""), 3.94 – 3.91 (m, 1H, H-5'), 3.91 – 3.88 (m, 1H, H-6), 3.88 – 3.82 (m, 5H, H02, H-3', H-3"". H-6*, H-6#), 3.82 – 3.77 (m, 2H, H-3*, H-6""), 3.73 (dd, *J* = 11.3, 7.2 Hz, 1H, H-6), 3.71 – 3.67 (m, 2H, H-3#, H-6""), 3.67 – 3.62 (m, 2H, H-6*, H-6#), 3.62 – 3.58 (m, 1H, H-4""), 3.56 – 3.50 (m, 2H, H-4*, H-4#), 3.50 – 3.46 (m, 2H, H-4', H-1/H-7), 3.41 (dd, *J* = 10.1, 7.9 Hz, 1H, H-3), 3.34 – 3.28 (m, 2H, H-5*/#), 3.26 (t, *J* = 10.2 Hz, 1H, H-4), 3.13 (d, *J* = 3.9 Hz, 1H, H-1/H-7), 2.09 – 2.03 (m, 1H, H-5), 1.17 (d, *J* = 6.3 Hz, 3H, H-6'). ^{13}C NMR (151 MHz, D_2O) δ 102.1 (C-1""/C-1*), 102.1 (C-1""/C-1*), 100.6 (C-1'), 97.6 (C-1#), 82.9 (C-3), 80.2 (C-2/C-3'/C-3""), 80.0 (C-3*), 77.8 (C-5*/#), 77.2 (C-5*/#), 76.6 (C-2#), 74.6 (C-3#), 73.2 (C-5""), 72.3 (C-2/C-3'/C-3""), 72.0 (C-4'), 71.4 (C-2'), 71.1 (C-2/C-3'/C-3""), 70.9 (C-2""), 69.8 (C-5'), 68.5 (C-2*), 67.8 (C-4*/C-4#), 67.5 (C-4""), 66.0 (C-4*/C-4#), 65.9 (C-4), 62.0 (C-6*/C-6#), 61.9 (C-6*/C-6#), 61.7 (C-6""), 61.2 (C-6), 57.1 (C-1/C-7), 56.9 (C-1/C-7), 44.2 (C-5), 17.5 (C-6'). HRMS [M+Na]$^+$ calculated 831.2741, found 831.2747.

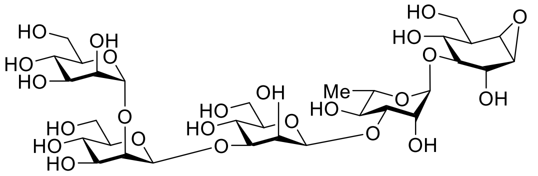

Synthesis of compound 25

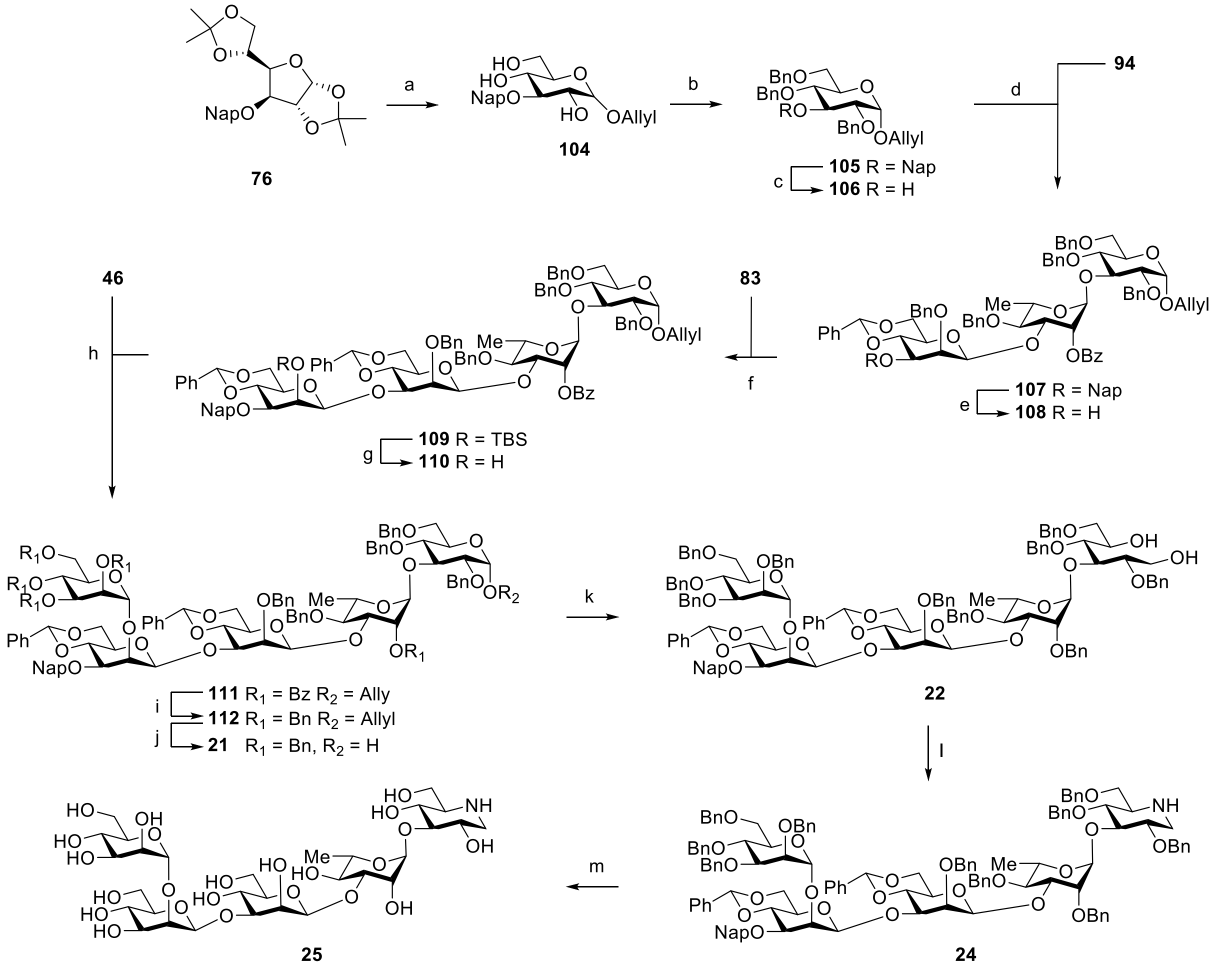

Scheme 9. a) Allyl alcohol, HCl, 90 °C, 88%; b) BnBr, NaH, TBAI, DMF,78%; c) DDQ, DCM:H_2O, 9:1, 47% α and 26% β; d) **83**, TBDMSOTf, DCM, 3 Å MS, -40 °C, 88%; e) DDQ, DCM:H_2O, 9:1, 67%; f) BSP, Tf_2O, TTBP, 3 Å MS, -60 °C, 99%; g) HF-pyridine, pyridine, 0 °C -> 50 °C -> r.t., 82%; h) TMSOTf, -20 °C -> 0 °C, 99%; i) i) NaOMe, DCM:MeOH,1:1; ii) BnBr, TBAI, NaH, DMF, 72% over 2 steps; j) i) $[Ir(PMePh_2)2COD]^+PF_6^-$, H_2, THF; ii) NIS, $NaHCO_3$, H_2O, THF, 83%; k) $LiAlH_4$, THF, 0 °C -> r.t., quant; l) i) $(COCl)_2$, DMSO, Et_3N, DCM, -78 °C to -10 °C; ii) $NaCNBH_3$, $HCOONH_4$, Na_2SO_4, MeOH, 0 °C to r.t., 54% (over 2 steps); m) NH_3, t-BuOH, THF, Na, -60 °C, 66%.

1-*O*-allyl-2,4,6-tri-*O*-benzyl-3-*O*-naphthyl-β-D-glucose (**104**). Compound **76** (9.1 mmol, 3.6 g) was dissolved in allyl alcohol (130 mL, 0.07 M) and HCl (1.8 mL, 18.2 mmol, 2 eq.) was added. The reaction mixture was heated to 90 °C, and after stirring for 1 hour TLC indicated almost complete conversion. After stirring for 1 additional hour TLC indicated no further conversion and the reaction was quenched with Et_3N (2.8 mL, 20 mmol, 2.2 eq.) and the reaction mixture was concentrated *in vacuo*. Column chromatography (50% -> 80% EtOAc in pentane) yielded **105** (2.9 g, 8 mmol) in 88%.

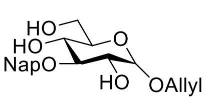

1-*O*-allyl-2,4,6-tri-*O*-benzyl-3-*O*-naphthyl-β-D-glucose (**105**). Compound **104** (2.9 g, 8 mmol) was dissolved in dry DMF (40 mL, 0.2 M) and cooled to 0 °C under N_2. Subsequently, BnBr (6.2 g, 36 mmol, 4.5 eq.), TBAI (0.3 g, 0.8 mmol, 0.1 eq.) and NaH (1.4 g, 36 mmol, 4.5 eq.) were added. After stirring for 15 hours TLC indicated full conversion and the reaction was quenched with H_2O at 0 °C. The reaction mixture was diluted with Et_2O, washed with H_2O (3x), dried over $MgSO_4$, filtrated and concentrated *in vacuo*. Column chromatography (5% ->30% Et_2O in pentane) yielded **105** (3.9 g, 6.2 mmol) in 78% in a 2:1 α:β ratio. ^1H NMR (400 MHz, $CDCl_3$) δ 7.86 – 7.04 (m, 35H), 6.04 – 5.87 (m, 1H), 5.40 – 5.29 (m, 2H), 5.26 – 5.05 (m, 3H), 5.03 – 4.91 (m, 2H), 4.91 – 4.82 (m, 2H), 4.82 – 4.72 (m, 2H), 4.72 – 4.52 (m, 4H), 4.52 – 4.41 (m, 3H), 4.22 – 4.12 (m, 2H), 4.12 – 3.98 (m, 2H), 3.88 – 3.80 (m, 1H), 3.78 – 3.59 (m, 6H), 3.59 – 3.52 (m, 1H), 3.52 – 3.42 (m, 1H). ^{13}C NMR (101 MHz, $CDCl_3$) δ 138.4, 138.3, 138.3, 138.2, 138.0, 136.5,

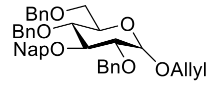

136.2, 134.2, 133.9, 133.5, 133.4, 133.1, 128.6, 128.5, 128.3, 128.2, 128.2, 128.0, 128.0, 128.0, 128.0, 127.9, 127.9, 127.8, 127.8, 127.8, 127.7, 126.6, 126.2, 126.1, 126.1, 125.9, 125.9, 118.3, 117.3, 102.9, 95.8, 84.8, 82.4, 82.3, 80.0, 78.0, 77.8, 77.6, 77.2, 76.9, 75.9, 75.8, 75.2, 75.1, 75.0, 73.6, 73.3, 70.4, 70.4, 68.6, 68.3. HRMS $[M+Na]^+$ calculated 653.2874, found 653.2874.

1-*O*-allyl-2,4,6-tri-*O*-benzyl-β-D-glucose (**106**). Compound **105** (0.31 g, 0.5 mmol) was dissolved in DCM:H_2O (10 mL, 0.05 M, 9:1) and DDQ (0.23 g, 1 mmol, 2 eq.) was added. After stirring for 1 hour in the dark, TLC indicated full conversion, and the mixture was diluted with DCM and quenched with sat. aq. $NaHCO_3$. The reaction mixture was then further diluted with DCM, washed with sat. aq. $NaHCO_3$, sat aq. $Na_2S_2O_3$, brine, dried over $MgSO_4$, filtrated and concentrated *in vacuo*. Column chromatography (10% -> 50% Et_2O in pentane) then yielded compound **106** (0.12 g, 0.24 mmol) in 47% together with the β anomer (0.063 g, 0.13 mmol) in 26%. α: ^{1}H NMR (400 MHz, $CDCl_3$) δ 7.41 – 7.07 (m, 15H, Aromatic), 5.88 (dddd, *J* = 16.9, 10.3, 6.4, 5.2 Hz, 1H, CH_2-Allyl), 5.29 (dq, *J* = 17.2, 1.6 Hz, 1H, CH_2-Allyl), 5.17 (dq, *J* = 10.3, 1.3 Hz, 1H, CH_2-Allyl), 4.87 – 4.82 (m, 2H, H-1, CH_2-Bn), 4.71 – 4.57 (m, 3H, CH_2-Bn), 4.54 – 4.41 (m, 2H, CH_2-Bn), 4.16 – 4.07 (m, 2H, H-3, CH_2-Allyl), 3.92 (ddt, *J* = 12.9, 6.4, 1.3 Hz, 1H, CH_2-Allyl), 3.77 (ddd, *J* = 9.8, 3.7, 2.0 Hz, 1H, H-5), 3.72 (dd, *J* = 10.5, 3.7 Hz, 1H, H-6), 3.63 (dd, *J* = 10.5, 2.0 Hz, 1H, H-6), 3.57 (dd, *J* = 9.9, 8.8 Hz, 1H, H-4), 3.41 (dd, *J* = 9.7, 3.5 Hz, 1H, H-2), 2.59 (d, *J* = 2.3 Hz, 1H. 3-OH).^{13}C NMR (101 MHz, $CDCl_3$) δ 138.5, 138.1, 138.0 (Aromatic), 133.8 (CH-Allyl), 128.6, 128.4, 128.4, 128.1, 128.1, 128.0, 128.0, 128.0, 127.9, 127.7, 127.7 (Aromatic), 117.9 (CH_2-Allyl), 95.4 (C-1), 79.5 (C-2), 77.5 (C-4), 74.6 (CH_2-Bn), 73.6 (C-3), 73.5 (CH_2-Bn), 72.9 (CH_2-Bn), 69.9 (C-5), 68.5 (C-6), 68.3 (CH_2-Allyl). β: ^{1}H NMR (400 MHz, $CDCl_3$) δ 7.42 – 7.16 (m, 15H, Aromatic), 5.95 (dddd, *J* = 17.3, 10.8, 6.0, 5.1 Hz, 1H, CH_2-Allyl), 5.33 (dq, *J* = 17.2, 1.6 Hz, 1H, CH_2-Allyl), 5.20 (dq, *J* = 10.5, 1.5 Hz, 1H, CH_2-Allyl), 4.97 (d, *J* = 11.4 Hz, 1H, CH_2-Bn), 4.84 (d, *J* = 11.1 Hz, 1H, CH_2-Bn), 4.66 (d, *J* = 11.4 Hz, 1H, CH_2-Bn), 4.62 (d, *J* = 12.2 Hz, 1H, CH_2-Bn), 4.58 – 4.50 (m, 2H, CH_2-Bn), 4.48 – 4.38 (m, 2H, H-1, CH_2-Allyl), 4.13 (ddt, *J* = 12.9, 5.9, 1.5 Hz, 1H, CH_2-Allyl), 3.78 – 3.63 (m, 3H, H-3, H-6), 3.55 – 3.40 (m, 2H, H-4, H-5), 3.30 (dd, *J* = 9.2, 7.8 Hz, 1H, H-2), 2.51 (d, *J* = 2.3 Hz, 1H, 3-OH). ^{13}C NMR (101 MHz, $CDCl_3$) δ 138.4, 138.4, 138.2 (Aromatic), 134.1 (CH-Allyl), 128.6, 128.5, 128.5, 128.4, 128.3, 128.1, 128.0, 127.9, 127.9, 127.9, 127.7 (Aromatic), 117.4 (CH_2-Allyl), 102.4 (C-1), 81.3 (C-2), 77.5 (C-4/C-5), 76.8 (C-3), 74.9 (C-4/C-5), 74.6 (CH_2-Bn), 74.5 (CH_2-Bn), 73.6 (CH_2-Bn), 70.3 (CH_2-Allyl), 69.0 (C-6). HRMS $[M+Na]^+$ calculated 513.2248, found 513.2248.

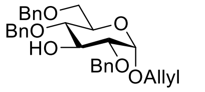

2-*O*-benzyl-4,6-*O*-benzylidene-3-*O*-naphthyl-β-D-mannose-(1->3)-2-*O*-benzoyl-4-*O*-benzyl-α-L-rhamnose-(1->3)-1-*O*-allyl-2,4,6-tri-*O*-benzyl-β-D-glucose (**107**). Compound **94** (0.49 mmol, 0.95 g, 1 eq.) and compound **106** (0.89 mmol, 0.43 g, 1.7 eq.) were co-evaporated (3x) with toluene and dissolved in dry DCM (5 mL, 0.1 M) under N_2 atmosphere. Activated Molecular Sieves (3Å) were added to the mixture and the mixture was stirred for 45 minutes. The solution was cooled to -40 °C, and TBDMSOTf (0.1 mL, 0.5M 0.1 eq.) was added dropwise. After stirring for 30 minutes, TLC showed full conversion, and the reaction was quenched using Et_3N at -40 °C. The mixture was diluted with DCM, washed with brine, the organic layer dried over $MgSO_4$, filtrated, and concentrated *in vacuo*. Size exclusion chromatography and silica gel chromatography (5% -> 25% EtOAc in pentane) yielded compound **107** (0.43 mmol, 0.56 g) in 88%. ^{1}H NMR (400 MHz, $CDCl_3$) δ 8.10 – 8.05 (m, 2H, Aromatic), 7.83 – 7.05 (m, 40H, Aromatic), 5.87 (dddd, *J* = 17.0, 10.3, 6.5, 5.2 Hz, 1H, CH-Allyl), 5.63 (d, *J* = 1.7 Hz, 1H, H-1'), 5.60 – 5.57 (m, 2H, Benzylidene, H-2'), 5.31 – 5.22 (m, 1H, CH_2-Allyl), 5.16 (dq, *J* = 10.3, 1.3 Hz, 1H, CH_2-Allyl), 4.90 (d, *J* = 12.3 Hz, 1H, CH_2-Bn/Nap), 4.82 – 4.46 (m, 13H, CH_2-Bn/Nap/ H-1, H-1"), 4.33 (dd, *J* = 10.4, 4.9 Hz, 1H, H-6"), 4.25 – 4.17 (m, 2H, H-3,H-3'), 4.15 (d, *J* = 9.6 Hz, 1H, H-4"), 4.12 – 4.02 (m, 2H, H-5´, CH_2-Allyl), 3.93 – 3.83 (m, 2H, H-6", CH_2-Allyl), 3.81 – 3.76 (m, 2H, H-5, H-6), 3.74 (d, *J* = 3.1 Hz, 1H, H-2"), 3.65 – 3.59 (m, 3H, H-4, H-6, H-4'), 3.57 (dd, *J* = 9.6, 3.5 Hz, 1H, H-2), 3.37 (dd, *J* = 9.9, 3.1 Hz, 1H, H-3"), 3.26 (dt, *J* = 9.6, 4.9 Hz, 1H, H-5"), 1.14 (d, *J* = 6.2 Hz, 3H, H-6').^{13}C NMR (101 MHz, $CDCl_3$) δ 165.4 (C=O), 138.7, 138.5, 138.2, 137.8, 137.8, 136.0 (Aromatic), 133.9 (CH_2-Allyl), 133.4, 133.1, 133.0, 130.5, 129.9, 129.0, 128.7, 128.6, 128.5, 128.5, 128.4, 128.4, 128.3, 128.2, 128.1, 128.1, 128.0, 128.0, 128.0, 127.8, 127.7, 127.5, 127.2, 126.3, 126.1, 125.9, 125.6 (Aromatic), 118.1 (CH_2-Allyl), 103.6 (C-1''), 101.5 (Benzylidene), 97.5 (C-1'), 95.8 (C-1), 82.3 (C-4/C-4'), 80.9 (C-2), 78.5 (C-4"), 78.2 (C-3'), 77.8 (C-3/C-3'), 76.8 (C-4/C-4'), 75.9 (C-2"), 75.7 (C-3/C-3'), 75.4, 75.1, 74.3, 73.8 (CH2-Bn/Nap), 73.5 (C-2'), 73.4, 72.3 (CH2-Bn/Nap), 70.3 (C-5), 68.7 (C-6), 68.6 (CH_2-Allyl), 68.5 (C-6"), 67.9 (C-5'), 67.8 (C-5"), 18.2 (C-6'). HRMS $[M+NH_4]^+$ calculated 1328.59, found 1328.59.

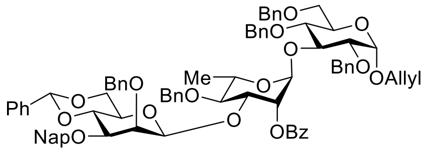

2-*O*-benzyl-4,6-*O*-benzylidene-β-D-mannose-(1->3)-2-*O*-benzoyl-4-*O*-benzyl-α-L-rhamnose-(1->3)-1-*O*-allyl-2,4,6-tri-*O*-benzyl-β-D-glucose (**108**). Compound **107** (1.8 mmol, 2.4 g, 1 eq.) was dissolved

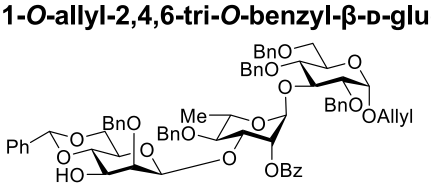

in DCM:H_2O (36 mL, 0.05 M, 9:1) and DDQ (2.7 mmol, 0.61 g, 1.5 eq.) was added. After stirring for 1.5 hours additional DDQ (0.15 g, 0.37 eq.) was added. After stirring for an additional 30 minutes TLC indicated no further conversion and the mixture was further diluted with DCM and quenched with sat. aq. $NaHCO_3$. The organic layer was washed with sat. aq. $NaHCO_3$ (x2), sat. aq. Na_2SO_3, brine dried over $MgSO_4$, filtrated and concentrated *in vacuo*. Silica gel chromatography (20% -> 50% Et_2O in pentane) yielded compound **108** (1.2 mmol, 1.4 g) in 67%. ^{1}H NMR (400 MHz, $CDCl_3$) δ 8.05 –7.04 (m, 35H, Aromatic), 5.87 (dddd, *J* = 17.0, 10.3, 6.5, 5.2 Hz, 1H, CH-Allyl), 5.65 – 5.63 (m, 2H, Benzylidene, H-2'), 5.43 (s, 1H, H-1'), 5.27 (dq, *J* = 17.2, 1.6 Hz, 1H, CH_2-Allyl), 5.19 – 5.14 (m, 1H, CH_2-Allyl), 4.91 – 4.77 (m, 4H, H-1", CH_2-Bn), 4.72 – 4.61 (m, 4H, H-1, CH_2-Bn), 4.55 (dd, *J* = 11.4, 5.2 Hz, 2H, CH_2-Bn), 4.47 (t, *J* = 11.4 Hz, 2H, CH_2-Bn), 4.35 – 4.22 (m, 3H, H-3, H-3', H-6"), 4.16 – 4.04 (m, 2H, H-5', CH_2-Allyl), 3.90 – 3.75 (m, 4H, H-5, H-6, H-6", CH_2-Allyl), 3.75 – 3.52 (m, 7H, H-2, H-4, H-6, H-4', H-2", H-3", H-4"), 3.24 (td, *J* = 9.6, 4.9 Hz, 1H, H-5"), 2.53 (d, *J* = 8.7 Hz, 1H, 3-OH"), 1.18 (t, *J* = 7.0 Hz, 3H, H-6'). ^{13}C NMR (101 MHz, $CDCl_3$) δ 165.3 (C=O), 138.2, 138.0, 137.6, 137.6, 137.3 (Aromatic), 133.7 (CH-Allyl), 132.9, 130.2, 129.7, 129.0, 128.5, 128.5, 128.4, 128.3, 128.2, 128.2, 128.2, 128.0, 128.0, 127.9, 127.8, 127.8, 127.8, 127.7, 127.6, 127.6, 127.3, 126.3 (Aromatic), 118.0 (CH_2-Allyl) , 103.3 (C-1"), 101.9 (C-1'), 97.4 (Benzylidene), 95.5 (C-1), 82.0 (C-2/C-4/C-4'/C-2"/C-3"/C-4"), 80.7 (C-2/C-4/C-4'/C-2"/C-3"/C-4"), 79.1 (C-2/C-4/C-4'/C-2"/C-3"/C-4"), 78.3 (C-2/C-4/C-4'/C-2"/C-3"/C-4"), 77.7 (C-3, C-3'), 76.6 (C-2/C-4/C-4'/C-2"/C-3"/C-4""), 75.6 (C-3/C-3'), 75.4, 75.0, 74.9, 73.6, 73.2 (CH_2-Bn), 73.2 (C-2'), 70.6 (C-2/C-4/C-4'/C-2"/C-3"/C-4"), 70.2 (C-5), 68.5 (C-6"), 68.4 (C-6), 68.3 (CH-Allyl), 67.70 (C-5'), 67.0 (C-5"), 18.0 (C-6'). HRMS $[M+Na]^+$ calculated 1193.487, found 1193.489.

4,6-*O*-benzylidene-2-*O*-tert-butyldimethylsilyl-3-*O*-naphthyl-β-D-mannose-(1->3)-2-*O*-benzyl-4,6-*O*-benzylidene-β-D-mannose-(1->3)-2-*O*-benzoyl-4-*O*-benzyl-α-L-rhamnose-(1->3)-1-*O*-allyl-2,4,6-tri-*O*-benzyl-β-D-glucose (**109**). Compound **46** (2.0

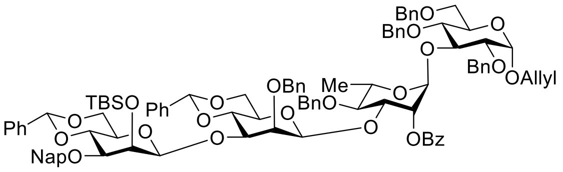

mmol, 1.27 g, 1.7 eq.), TTBP (3.6 mmol, 0.89 g, 3 eq.) and BSP (2.15 mmol, 0.45 g, 1.8eq.) were co-evaporated (3x) with toluene and dissolved in dry DCM (24 mL, 0.05 M). 3Å MS were added and the solution was stirred for 1 hour under N_2. The mixture was cooled to -70 °C and Tf_2O (2.15 mmol, 0.36 mL, 1.8 eq.) was added dropwise. After stirring for 1 hour, the solution had warmed to -55 °C and was re-cooled to -60 °C before adding compound **108** (1.2 mmol, 1.4 g, 1 eq., in 2 mL DCM), which was co-evaporated 3 times with toluene, in 10 minutes. After stirring for 2.5 hours TLC indicated full conversion and the reaction was quenched with pyridine at -60 °C. The mixture was diluted with DCM and washed with sat. aq. $NaHCO_3$, brine, dried over $MgSO_4$, filtrated, and concentrated *in vacuo*. Silica gel chromatography (5% -> 30% EtOAc in pentane) yielded compound **109** (1.97 g, 1.17 mmol) in 99%. ^{1}H NMR (400 MHz, $CDCl_3$) δ 8.37 – 7.36 (d, *J* = 1.6 Hz, 47H, Aromatic), 6.19 (dddd, *J* = 17.0, 10.3, 6.6, 5.3 Hz, 1H, CH-Allyl), 5.96 – 5.91 (m, 2H, H-2', Benzylidene), 5.89 (s, 1H, H-1'), 5.78 (s, 1H, Benzylidene), 5.61 (q, *J* = 1.6 Hz, 1H, CH_2-Allyl), 5.48 (dq, *J* = 10.3, 1.2 Hz, 1H, CH_2-Allyl), 5.21 (d, *J* = 12.4 Hz, 1H, CH_2-Bn/Nap), 5.15 (d, *J* = 3.5 Hz, 2H, CH_2-Bn/Nap), 5.11 (d, *J* = 8.0 Hz, 2H, H-1", CH_2-Bn/Nap), 5.07 – 5.03 (m, 2H, CH_2-Bn/Nap), 5.03 – 4.93 (m, 3H, H-1, CH_2-Bn/Nap), 4.90 – 4.78 (m, 4H, CH_2-Bn/Nap), 4.66 (dd, *J* = 10.4, 4.7 Hz, 1H, H-6"), 4.62 – 4.55 (m, 1H, H-3'), 4.53 (d, *J* = 9.4 Hz, 1H, H-3), 4.49 – 4.33 (m, 4H, H-5', H-4"', H-6", CH_2-Allyl), 4.30 – 4.24 (m, 2H, H-4", H-1"'), 4.23 – 4.15 (m, 3H, H-2", H-6", CH_2-Allyl), 4.15 – 4.05 (m, 5H, H-5, H-6, H-2"', H-3"', H-6"'), 4.00 – 3.92 (m, 3H, H-4, H-6, H-4') 3.90 (dd, *J* = 9.5, 3.6 Hz, 1H, H-2), 3.65 (dt, *J* = 9.5, 4.8 Hz, 1H, H-5"), 3.59 (dd, *J* = 9.6, 2.7 Hz, 1H, H-3"), 3.34 (td, *J* = 9.6, 4.8 Hz, 1H, H-5"'), 1.64 – 1.54 (m, 6H, CH_3-TBS), 1.48 (d, *J* = 6.1 Hz, 3H, H-6'), 1.11 (s, 9H, CH_3-TBS). ^{13}C NMR (101 MHz, $CDCl_3$) δ 165.3 (C=O), 138.4, 138.4, 138.2, 137.8, 137.7, 137.7, 137.6, 136.1 (Aromatic), 133.8 (CH-Allyl), 133.4, 133.0, 133.0, 130.4, 129.8, 129.1, 129.0, 128.9, 128.7, 128.6, 128.5, 128.4, 128.4, 128.3, 128.2, 128.2, 128.1, 128.6, 128.0, 127.9, 127.9, 127.8, 127.8, 127.7, 127.7, 127.6, 127.5, 127.0, 126.6, 126.5, 126.3, 126.3, 126.1, 125.9, 125.9, 125.7 (Aromatic), 118.2 (CH_2-Allyl), 103.8 (C-1"), 101.8 (Benzylidene), 101.6 (C-1'), 97.5 (Benzylidene), 96.6 (C-1"'), 95.7 (C-1), 82.1 (C-4/C-4'), 80.9 (C-2), 78.9 (C-5'/C-4"'), 77.9 (C-3'), 77.7 (C-3"), 76.7 (C-4/C-4'), 76.6 (C-4"), 75.7 (C-3), 75.3 (CH_2-Bn/Nap), 75.6 (CH_2-Bn/Nap), 74.2 (C-5/C-2"'/C-3"'), 74.1 (CH_2-Bn/Nap), 73.9 (C-5/C-2"'/C-3"'), 73.8 (CH_2-Bn/Nap), 73.4 (CH_2-Bn/Nap), 73.3 (C-2'), 71.9 (CH_2-Bn/Nap),

71.0 (C-2”), 70.3 (C-5/C-2”’/C-3”’), 68.9 (C-6”’), 68.7 (C-6”), 68.5 (C-6), 68.5 (CH_2-Allyl), 68.0 (C-5’/C-4”’), 67.8 (C-5”), 67.7 (C-5”’), 29.8 (CH_3-TBS), 25.9 (CH_3-TBS), 25.8 (CH_3-TBS), 18.5 (C-TBS), 18.1 (C-6’).

4,6-*O*-benzylidene-3-*O*-naphthyl-β-ᴅ-mannose-(1->3)-2-*O*-benzyl-4,6-*O*-benzylidene-β-ᴅ-mannose-(1->3)-2-*O*-benzoyl-4-*O*-benzyl-α-ʟ-rhamnose-(1->3)-1-*O*-allyl-2,4,6-tri-*O*-benzyl-β-ᴅ-glucose (**110**). Compound **109** (0.88 mmol, 1.48 g, 1 eq.) was co-evaporated (3x) with toluene and dissolved in dry pyridine (22.2 mL, 0.04 M,). The mixture was cooled to 0 °C and HF-pyridine (11.5 mL, 70% HF in pyridine) was added dropwise. The reaction mixture was heated to 50 °C and stirred for 3.5 hours. TLC showed that there was still starting material, and the reaction was left overnight at r.t. After 18 hours, TLC indicated that there was a very small amount of starting material, and the reaction was worked up. The crude product was diluted with EtOAc (60 mL) washed with sat. aq. $NaHCO_3$, 1M HCl (2x), brine, dried over $MgSO_4$, filtrated and concentrated *in vacuo*. Silica gel chromatography (10% -> 40% EtOAc in pentane) yielded compound **110** (0.73 mmol, 1.14 g) in 82%, while 3% of compound **108** (0.03 mmol, 52 mg) was recovered. ^{1}H NMR (400 MHz, $CDCl_3$) δ 8.11 – 6.96 (m, 47H, Aromatic), 5.87 (dddd, *J* = 17.0, 10.3, 6.6, 5.2 Hz, 1H, CH-Allyl), 5.64 (d, *J* = 1.7 Hz, 1H, H-1’), 5.60 (dd, *J* = 3.4, 1.7 Hz, 1H, H-2’), 5.50 (s, 1H, Benzylidene), 5.38 (s, 1H, Benzylidene), 5.31 – 5.24 (m, 1H, CH_2-Allyl), 5.16 (dt, *J* = 10.3, 1.4 Hz, 1H, CH_2-Allyl), 4.93 – 4.87 (m, 3H, CH_2-Bn/Nap), 4.80 (s, 1H, H-1”), 4.78 – 4.69 (m, 3H, CH_2-Bn/Nap), 4.68 (d, *J* = 3.5 Hz, 1H, H-1), 4.66 – 4.45 (m, 6H, CH_2-Bn/Nap), 4.34 (dd, *J* = 10.5, 4.8 Hz, 1H, H-6”), 4.29 – 4.25 (m, 1H, H-3’), 4.21 (d, *J* = 9.4 Hz, 1H, H-3), 4.15 – 4.00 (m, 7H, H-5’, H-4”, H-1”’, H-4”’, H-6”’, CH_2-Allyl), 3.91 – 3.78 (m, 6H, H-6, H-2”, H-6”, CH_2-Allyl), 3.71 – 3.61 (m, 5H, H-4, H-4’, H-2”’, H-6”’), 3.58 (dd, *J* = 9.5, 3.6 Hz, 1H, H-2), 3.45 (dd, *J* = 9.3, 3.7 Hz, 1H, H-3”), 3.29 (td, *J* = 9.7, 4.8 Hz, 1H, H-5”), 3.05 (td, *J* = 9.8, 4.9 Hz, 1H, H-5”’), 1.16 (d, *J* = 6.2 Hz, 3H, H-6’). ^{13}C NMR (101 MHz, $CDCl_3$) δ 165.4 (C=O), 138.4, 138.2, 138.1, 137.8, 137.7, 137.6, 137.4, 135.7, 133.8, 133.3, 133.1, 133.1, 130.4, 129.8, 129.1, 129.0, 128.7, 128.6, 128.5, 128.5, 128.4, 128.4, 128.3, 128.3, 128.2, 128.1, 128.0, 128.0, 128.0, 127.9, 127.8, 127.7, 127.7, 127.1, 126.8, 126.3, 126.2, 126.2, 126.1, 125.9 (Aromatic), 118.1 (CH-Allyl), 103.4 (C-1”), 101.7 (Benzylidene), 101.4 (Benzylidene), 97.5 (C-1’), 96.8 (C-1”’), 95.7 (C-1), 82.2 (C-4/C-4’/C-2”’), 80.8 (C-2), 78.4 (C-5’/C-4”/C-4”’), 77.6 (C-3’), 76.7 (C-5’/C-4”/C-4”’), 76.7 (C-4/C-4’/C-2”’), 75.7 (C-3) 75.7 (C-3”), 75.30, 75.0 (CH_2-Bn/Nap), 74.7 (C-2”, C-4/C-4’/C-2”’), 74.6, 74.2, 73.8, 73.4 (CH_2-Bn/Nap), 73.3 (C-2’), 72.2 (CH_2-Bn/Nap), 70.2, 69.7, 68.6 (C-6”, CH_2-Allyl), 68.5 (C-6”’), 68.5 (C-6), 67.8 (C-5”), 66.9 (C-5”’), 18.1 (C-6’). HRMS $[M+NH_4]^+$ calculated 1579.68, found 1579.68.

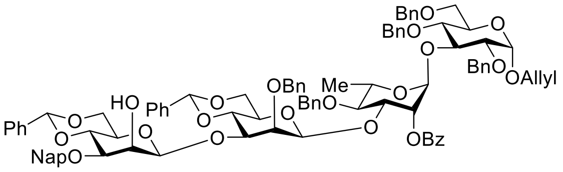

2,3,4,6-tetra-*O*-benzoyl-α-ᴅ-mannose-(1->2)-4,6-*O*-benzylidene-3-*O*-naphthyl-β-ᴅ-mannose-(1->3)-2-*O*-benzyl-4,6-*O*-benzylidene-β-ᴅ-mannose-(1->3)-2-*O*-benzoyl-4-*O*-benzyl-α-ʟ-rhamnose-(1->3)-1-*O*-allyl-2,4,6-tri-*O*-benzyl-β-ᴅ-glucose (**111**). Compound **110** (0.22 mmol, 0.34 g, 1 eq.) and compound **46** (0.82 mmol, 0.63 g, 3.75 eq.) were co-evaporated (3x) with toluene, and dissolved in dry DCM (2.2 mL, 0.1 M) under N_2. Activated MS (3Å) were added, and the solution was stirred for 1 hour. The mixture was cooled to -20 °C, and TMSOTf (0.1 mL, 0.22 M in dry DCM, 0.1 eq.) was added dropwise. The solution was warmed to 0 °C using an ice bath. After stirring for 2 hours at 0 °C, TLC indicated complete conversion, and the reaction was quenched with Et_3N. Subsequently, the reaction mixture was diluted with DCM, washed with sat. aq. $NaHCO_3$, brine, dried over $MgSO_4$, filtrated and concentrated *in vacuo*. Size exclusion chromatography and silica gel chromatography (25% -> 35% EtOAc in pentane) yielded compound **111** (0.21 mmol, 0.34 g) as a white solid in 99%. ^{1}H NMR (400 MHz, $CDCl_3$) δ 8.15 – 6.87 (m, 67H, Aromatic), 6.18 (t, *J* = 10.3 Hz, 1H, H-4””), 6.03 (dd, *J* = 3.2, 1.7 Hz, 1H, H-2””), 5.94 – 5.81 (m, 2H, H-3””, CH-Allyl), 5.76 (s, 1H, Benzylidene), 5.65 (d, *J* = 1.7 Hz, 1H, H-1’), 5.60 (dd, *J* = 3.4, 1.7 Hz, 1H, H-2’), 5.50 – 5.45 (m, 2H, H-1””, Benzylidene), 5.30 – 5.24 (m, 1H, CH_2Allyl), 5.16 (dq, *J* = 10.3, 1.3 Hz, 1H, CH_2-Allyl), 5.02 (d, *J* = 12.5 Hz, 1H, CH_2-Bn/Nap), 4.92 – 4.83 (m, 3H, H-5””, CH_2-Bn/Nap), 4.81 – 4.71 (m, 4H, H-1”, CH_2-Bn/Nap), 4.70 – 4.61 (m, 5H, H-1, H-6”” CH_2-Bn/Nap), 4.59 – 4.47 (m, 4H, CH_2-Bn/Nap), 4.33 – 4.20 (m, 5H, H-3, H-3’), 4.16 – 4.04 (m, 3H, H-5’, H-6”” CH_2-Allyl), 3.99 – 3.93 (m, 3H, H-H-1”’), 3.92 – 3.83 (m, 2H, CH_2-Allyl (1H)), 3.83 – 3.77 (m, 3H), 3.75 – 3.57 (m, 6H, H-2, H-4’), 3.45 (dd, *J* = 9.7, 2.9 Hz, 1H), 3.27 (td, *J* = 9.6, 4.8 Hz, 1H, H-5”/”’), 3.08 (td, *J* = 9.6, 4.9 Hz, 1H, H-5”/”’), 1.15 (d, *J* = 6.1 Hz, 3H, H-6’). ^{13}C NMR (101 MHz, $CDCl_3$) δ 166.0, 165.7, 165.5, 165.3,

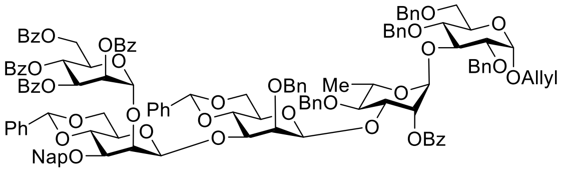

165.1, 138.5, 138.2, 138.0, 137.8, 137.6, 135.7 (Aromatic), 133.8 (CH-Allyl), 133.3, 133.2, 133.1, 133.0, 133.0, 132.9, 130.5, 130.3, 130.0, 129.9, 129.8, 129.8, 129.7, 129.4, 129.2, 129.2, 129.1, 128.9, 128.8, 128.7, 128.6, 128.6, 128.5, 128.5, 128.5, 128.4, 128.4, 128.4, 128.3, 128.3, 128.2, 128.1, 128.0, 128.0, 128.0, 127.8, 127.7, 127.7, 126.7, 126.6, 126.3, 126.1, 126.0, 125.9, 125.8, 125.4 (Aromatic), 118.2 (CH_2-Allyl), 104.1 (C-1''), 101.6 (Benzylidene), 100.9 (C-1''''/Benzylidene), 99.4(C-1''''/Benzylidene), 97.5 (C-1'), 96.6 (C-1), 95.7 (C-1'''), 82.3, 80.9, 79.3, 77.9, 77.4, 76.7, 76.2, 75.6, 75.4, 75.1, 75.0, 73.8, 73.7, 73.4, 73.2, 73.0, 70.6, 70.5, 70.3, 69.1, 68.7, 68.6, 68.5, 68.2, 67.8, 67.5, 66.5, 62.3, 18.1.

2,3,4,6-tetra-*O*-benzyl-α-D-mannose-(1->2)-4,6-*O*-benzylidene-3-*O*-naphthyl-β-D-mannose-(1->3)-2-*O*-benzyl-4,6-*O*-benzylidene-β-D-mannose-(1->3)-2-*O*-benzyl-4-*O*-benzyl-α-L-rhamnose-(1->3)-1-*O*-allyl-2,4,6-tri-*O*-benzyl-β-D-glucose (**112**). Compound **111** (0.21 mmol, 0.46 g, 1eq.) was suspended in DCM:MeOH (2.1 mL, 0.1 M, 1:1), and NaOMe (4.377 M) was added until pH 13 (total of 8 drops). After 24 hours TLC showed full conversion and the mixture was diluted with DCM, washed with sat. aq. $NaHCO_3$, brine, dried over $MgSO_4$, filtrated, and concentrated *in vacuo*. Silica gel column chromatography (0% -> 8% MeOH in DCM) yielded debenzoylated compound **111** (0.21 mmol, 0.33 g) in 97%, however NMR analysis then indicated a small amount of compound still contained one benzoyl and the reaction was restarted in MeOH (2 mL) and DCM (0.6 mL) and 10 drops of NaOMe (4.377 M) were added. After 5 hours the reaction was quenched with AcOH and column chromatography (0% -> 8% MeOH in DCM) yielded pure debenzoylated compound **111** (0.21 mmol, 0.33 g) in 96%. ^{1}H NMR (400 MHz, $CDCl_3$) δ 7.79 –6.97 (m, 42H), 5.88 (dddd, *J* = 17.0, 10.3, 6.6, 5.2 Hz, 1H), 5.60 – 5.45 (m, 3H), 5.32 – 5.25 (m, 2H), 5.19 (dq, *J* = 10.4, 1.3 Hz, 1H), 4.92 – 4.73 (m, 5H), 4.70 – 4.58 (m, 5H), 4.56 – 4.46 (m, 4H), 4.21 – 4.14 (m, 3H), 4.15 – 4.00 (m, 6H), 3.96 – 3.89 (m, 3H), 3.88 – 3.73 (m, 10H), 3.70 (d, *J* = 3.1 Hz, 1H), 3.66 – 3.56 (m, 3H), 3.54 – 3.44 (m, 2H), 3.37 (d, *J* = 9.8 Hz, 1H), 3.29 (td, *J* = 9.5, 4.9 Hz, 1H), 3.03 (td, *J* = 9.4, 5.1 Hz, 1H), 1.10 (d, *J* = 6.2 Hz, 3H). ^{13}C NMR (101 MHz, $CDCl_3$) δ 138.8, 137.8, 137.8, 137.7, 137.6, 137.6, 135.6, 133.8, 133.3, 133.1, 129.2, 129.1, 128.9, 128.7, 128.6, 128.6, 128.4, 128.4, 128.4, 128.3, 128.3, 128.2, 128.2, 128.2, 128.1, 128.0, 127.8, 127.7, 127.7, 126.6, 126.4, 126.4, 126.3, 126.1, 125.6, 118.3, 103.6, 101.6, 100.8, 99.8, 95.8, 95.2, 82.3, 81.1, 80.6, 78.9, 77.5, 77.2, 76.9, 76.8, 76.2, 76.1, 75.0, 74.8, 74.0, 73.8, 73.5, 72.7, 72.1, 71.6, 71.0, 70.9, 70.3, 68.6, 68.4, 67.9, 67.6, 67.4, 62.3, 29.8, 18.0.

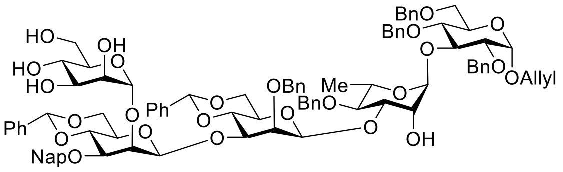

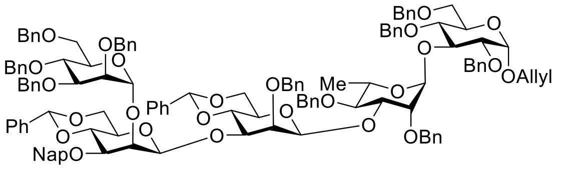

Debenzoylated compound **111** (0.063 mmol, 0.1 g) was co-evaporated thrice with toluene and dissolved in dry DMF (0.63 mL, 0.1 M) under N_2 and cooled to 0 °C. Subsequently, BnBr (0.056 mL, 0.47 mmol, 7.5 eq.), TBAI (1.5 mg, 4 μmol, 0.07 eq.) and NaH (19 mg, 0.47 mmol, 7.5 eq., 60% wt.) were added and the reaction was allowed to reach room temperature. After stirring for 20 hours additional BnBr (0.019 mL, 0.016 mmol, 2.5 eq.) and NaH (6.3 mg, 0.016 mmol, 2.5 eq., 60% wt.) were added. After stirring for an additional 3 hours TLC indicated complete conversion and the reaction was quenched with MeOH at 0 °C. The reaction mixture was diluted with Et_2O, washed with H_2O (2x), brine, dried over $MgSO_4$, filtrated and concentrated *in vacuo*. Column chromatography (5% -> 30% EtOAc in pentane) yielded compound **112** (0.094 g, 0.046 mmol) in 72%. ^{1}H NMR (400 MHz, $CDCl_3$) δ 7.91 – 6.94 (m, 67H), 5.89 (dddd, *J* = 17.1, 10.3, 6.6, 5.2 Hz, 1H), 5.72 – 5.61 (m, 1H), 5.47 – 5.40 (m, 2H), 5.33 – 5.26 (m, 2H), 5.25 – 5.15 (m, 2H), 5.00 – 4.83 (m, 3H), 4.83 – 4.31 (m, 22H), 4.28 – 3.73 (m, 23H), 3.73 – 3.54 (m, 8H), 3.54 – 3.45 (m, 2H), 3.20 (td, *J* = 9.7, 5.0 Hz, 1H), 3.12 – 3.02 (m, 1H), 1.09 (d, *J* = 6.2 Hz, 3H). ^{13}C NMR (101 MHz, $CDCl_3$) δ 139.2, 138.9, 138.8, 138.8, 138.2, 138.0, 137.8, 137.8, 137.7, 133.8, 133.3, 133.2, 129.1, 129.1, 128.6, 128.6, 128.6, 128.5, 128.4, 128.4, 128.3, 128.3, 128.2, 128.2, 128.2, 128.1, 128.1, 128.0, 127.9, 127.9, 127.8, 127.8, 127.8, 127.7, 127.6, 127.5, 127.5, 127.4, 127.4, 127.3, 127.2, 127.1, 126.9, 126.7, 126.5, 126.4, 126.3, 126.2, 126.2, 126.1, 126.1, 118.3, 104.4, 101.6, 100.6, 98.8, 98.7, 96.3, 95.4, 81.9, 81.0, 80.6, 80.4, 79.4, 78.6, 77.5, 77.2, 76.8, 76.8, 76.3, 76.1, 75.7, 75.1, 74.8, 74.0, 73.8, 73.4, 72.7, 72.5, 72.0, 71.8, 70.3, 69.1, 68.8, 68.5, 68.4, 68.3, 68.1. HRMS $[M+NH_4]^+$ calculated 2087.943, found 2087.749.

2,3,4,6-tetra-*O*-benzyl-α-D-mannose-(1->2)-4,6-*O*-benzylidene-3-*O*-naphthyl-β-D-mannose-(1->3)-2-*O*-benzyl-4,6-*O*-benzylidene-β-D-mannose-(1->3)-2-*O*-benzyl-4-*O*-benzyl-α-L-rhamnose-(1->3)-2,4,6-tri-*O*-benzyl-β-D-glucose (**21**). Compound **112** (0.15 g, 0.074 mmol) was dissolved in THF (2 mL) and sonicated under argon for 10 minutes. $[Ir(PMePh_2)_2COD]^+PF_6^-$ (3 mg, 3.7 μmol, 0.05 eq.) was

dissolved in THF (2 mL) and sonicated for 10 minutes under Argon. The solution was then flushed with

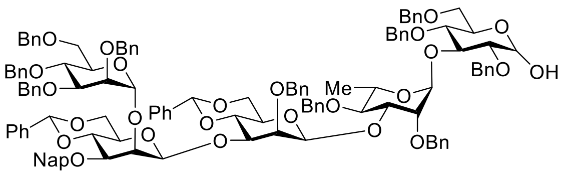

H_2 for 1 minute or until all color disappeared, subsequently the solution was flushed with argon for ~1-2 minute or until starting to turn reddish again, to remove excess H_2. The solution containing compound **112** was then added to the Iridium catalyst. After stirring for 1.15 hours, additional THF (0.4 mL), H_2O (1 mL), $NaHCO_3$ (14 mg, 0.17 mmol, 2.3 eq.) and NIS (26 mg, 0.11 mmol, 1.5 eq.) were added. After stirring for 4 hours TLC indicated complete conversion and the reaction mixture was diluted with EtOAc, washed with sat. aq. $Na_2S_2O_3$, brine, dried over $MgSO_4$, filtrated and concentrated *in vacuo*. Column chromatography (20% -> 40% EtOAc in pentane) yielded compound **21** (0.13 g, 0.062 mmol) in 83% as an α/β mixture. ^{1}H NMR (400 MHz, $CDCl_3$) δ 7.90 – 6.94 (m, 67H), 5.66 (d, J = 11.3 Hz, 1H), 5.48 – 5.42 (m, 1H), 5.40 (d, J = 1.7 Hz, 1H), 5.23 (s, 5H), 5.16 (d, J = 3.4 Hz, 1H), 5.04 – 4.80 (m, 5H), 4.80 – 4.63 (m, 6H), 4.63 – 4.31 (m, 20H), 4.27 – 4.21 (m, 1H), 4.20 – 3.45 (m, 39H), 3.34 (dd, J = 9.1, 7.7 Hz, 1H), 3.27 (s, 1H), 3.23 – 3.12 (m, 1H), 3.08 (s, 1H), 1.13 – 1.06 (m, 4H). ^{13}C NMR (101 MHz, $CDCl_3$) δ 139.1, 138.8, 138.8, 138.7, 138.3, 138.2, 137.9, 137.8, 137.7, 137.7, 137.5, 137.5, 135.6, 135.6, 133.3, 133.2, 133.1, 129.1, 129.0, 128.8, 128.7, 128.7, 128.6, 128.5, 128.5, 128.4, 128.4, 128.3, 128.3, 128.2, 128.2, 128.1, 128.1, 128.0, 128.0, 127.9, 127.9, 127.8, 127.8, 127.7, 127.7, 127.5, 127.5, 127.4, 127.4, 127.3, 127.3, 127.2, 127.1, 126.9, 126.6, 126.5, 126.4, 126.3, 126.3, 126.2, 126.2, 126.1, 126.0, 125.9, 104.4, 101.7, 101.5, 101.5, 100.6, 98.8, 98.6, 97.5, 96.3, 90.7, 83.6, 81.8, 81.1, 80.5, 80.3, 80.2, 79.3, 79.3, 78.8, 78.5, 78.0, 77.5, 77.2, 76.9, 76.8, 76.7, 76.0, 75.7, 75.1, 75.0, 74.9, 74.8, 74.1, 74.0, 73.7, 73.3, 73.1, 72.6, 72.6, 72.0, 72.0, 71.8, 70.4, 69.0, 68.8, 68.6, 68.4, 68.3, 68.0, 67.1, 60.5, 17.9.

2,3,4,6-tetra-*O*-benzyl-α-ᴅ-mannose-(1->2)-4,6-*O*-benzylidene-3-*O*-naphthyl-β-ᴅ-mannose-(1->3)-2-*O*-benzyl-4,6-*O*-benzylidene-β-ᴅ-mannose-(1->3)-2-*O*-benzyl-4-*O*-benzyl-α-ʟ-rhamnose-(1->3)-2,4,6-tri-*O*-benzyl-β-ᴅ-glucitol (**22**). Compound **21** (0.06 mmol, 0.12 g) was coevaporated with toluene

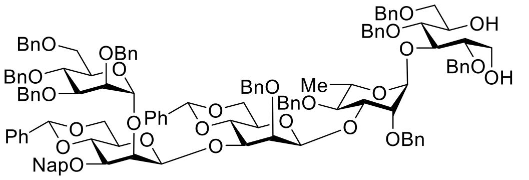

(3x) and dissolved in dry THF (0.6 mL, 0.1 M) under argon. The solution was cooled to 0 °C and $LiAlH_4$ (0.18 mL, 1 M, 3 eq.) was added slowly. After stirring for 10 minutes the ice bath was removed and the reaction mixture was allowed to stir at room temperature. After stirring for 3 hours TLC indicated complete conversion and the reaction mixture was cooled to 0 °C and quenched with H_2O. Sat. aq. sodium tartrate was added, and the reaction was stirred until the aluminum salts had dissolved. The mixture was then diluted with ethyl acetate, washed with H_2O, brine, dried over $MgSO_4$, filtrated and concentrated *in vacuo*. Compound **22** (0.12 g, 0.06 mmol) was yielded pure without any further purification quantitatively. ^{1}H NMR (400 MHz, $CDCl_3$) δ 7.87 – 6.96 (m, 77H), 5.74 – 5.63 (m, 1H), 5.50 – 5.40 (m, 1H), 5.20 (s, 1H), 5.02 – 4.80 (m, 5H), 4.80 – 4.29 (m, 22H), 4.29 – 4.21 (m, 1H), 4.21 – 3.90 (m, 14H), 3.90 – 3.44 (m, 19H), 3.14 (qt, J = 13.0, 5.9 Hz, 3H), 1.30 (d, J = 6.2 Hz, 3H). ^{13}C NMR (101 MHz, $CDCl_3$) δ 139.1, 138.8, 138.7, 138.7, 138.7, 138.6, 138.3, 138.2, 138.1, 138.0, 138.0, 137.7, 135.6, 133.3, 133.2, 129.1, 128.9, 128.8, 128.7, 128.7, 128.6, 128.6, 128.5, 128.4, 128.4, 128.3, 128.3, 128.3, 128.2, 128.1, 128.0, 128.0, 127.9, 127.9, 127.8, 127.8, 127.8, 127.7, 127.6, 127.5, 127.5, 127.5, 127.4, 127.3, 127.3, 127.2, 127.1, 126.9, 126.7, 126.4, 126.3, 126.2, 126.2, 126.1, 126.0, 104.1, 101.6, 100.9, 100.6, 98.8, 96.3, 81.1, 80.3, 80.0, 79.5, 79.4, 79.0, 78.5, 77.6, 77.5, 77.2, 76.8, 76.0, 75.1, 74.9, 74.9, 74.0, 73.6, 73.4, 73.4, 73.2, 73.0, 72.0, 71.8, 71.0, 70.8, 69.6, 69.1, 68.8, 68.3, 67.9, 61.5, 18.0. HRMS [M+Na]$^+$ calculated 2054.882, found 2054.885.

2,3,4,6-tetra-*O*-benzyl-α-ᴅ-mannose-(1->2)-4,6-*O*-benzylidene-3-*O*-naphthyl-β-ᴅ-mannose-(1->3)-2-*O*-benzyl-4,6-*O*-benzylidene-β-ᴅ-mannose-(1->3)-2-*O*-benzyl-4-*O*-benzyl-α-ʟ-rhamnose-(1->3)-2,4,6-tri-*O*-benzyl-1-deoxynojirimycin (**24**). A freshly prepared solution of $(COCl)_2$ (0.1 mL, 1.35 M in

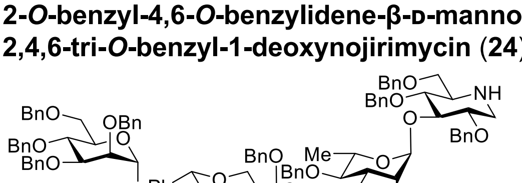

dry DCM) was dissolved in dry DCM (1 mL, 0.12 M) under Argon and immediately cooled to -80 °C. DMSO (0.1 mL, 1.35 M) was added slowly, in eight minutes, and the mixture was stirred for 1 hour while allowing it to react -67 °C. Subsequently, compound **23** (0.039 mmol, 0.079 g) in DMSO (0.3 mL, 0.13 M) was added dropwise, the flask was then washed with additional DMSO (0.2 mL) and added to the reaction dropwise. The reaction was kept between -60 °C and -67 °C, after stirring for 2 hours, the mixture was cooled to -70 °C and Et_3N

(0.06 mL, 0.43 mmol, 11 eq.) was added dropwise. The reaction was stirred for an additional 2.5 hours while gradually reaching -10 °C and then transferred to a mixture containing cold MeOH (2.3 mL, 0.017 M, 0 °C), Na_2SO_4 (0.15 mmol, 24 mg, 4 eq.), CH_3COONH_4 (0.9 mmol, 57 mg, 23.5 eq.) and $NaCNBH_4$ (0.17 mmol, 12 mg, 4.35 eq.). The reaction mixture was then concentrated *in vacuo*, redissolved in EtOAc, washed with H_2O, sat. aq. $NaHCO_3$, brine, dried over $MgSO_4$, filtrated and concentrated *in vacuo*. Size exclusion chromatography yielded compound **24** (0.036 g, 0.018 mmol) in 47%. ^1H NMR (600 MHz, $CDCl_3$) δ 7.86 – 6.89 (m, 67H), 5.65 (d, *J* = 20.8 Hz, 1H), 5.50 (s, 1H), 5.43 (d, *J* = 14.6 Hz, 1H), 5.23 (s, 1H), 4.97 (dd, *J* = 12.0, 2.5 Hz, 1H), 4.93 – 4.83 (m, 2H), 4.82 – 4.74 (m, 2H), 4.68 (d, *J* = 11.0 Hz, 2H), 4.63 – 4.31 (m, 15H), 4.27 – 3.55 (m, 24H), 3.54 – 3.46 (m, 2H), 3.36 (tt, *J* = 9.7, 4.8 Hz, 1H), 3.27 (dt, *J* = 12.3, 4.0 Hz, 1H, H-1), 3.16 (d, *J* = 11.6 Hz, 1H), 3.11 – 3.03 (m, 1H), 2.76 (ddd, *J* = 9.0, 5.7, 3.0 Hz, 1H), 2.47 (t, *J* = 11.5 Hz, 1H, H-1), 1.13 (dd, *J* = 6.4, 2.6 Hz, 3H). ^{13}C NMR (151 MHz, $CDCl_3$) δ 139.2, 138.8, 138.8, 138.8, 138.8, 138.5, 138.5, 138.3, 138.2, 138.1, 138.1, 138.0, 137.8, 137.8, 137.8, 137.7, 137.4, 135.7, 135.6, 133.4, 133.3, 133.3, 133.2, 133.1, 133.0, 130.7, 129.1, 129.0, 129.0, 128.8, 128.6, 128.6, 128.6, 128.5, 128.5, 128.4, 128.4, 128.4, 128.3, 128.3, 128.3, 128.2, 128.2, 128.2, 128.2, 128.1, 128.1, 128.1, 128.0, 128.0, 127.9, 127.9, 127.9, 127.8, 127.8, 127.8, 127.7, 127.6, 127.5, 127.5, 127.5, 127.4, 127.4, 127.4, 127.3, 127.3, 127.2, 127.1, 127.0, 126.9, 126.7, 126.6, 126.5, 126.4, 126.4, 126.3, 126.2, 126.2, 126.2, 126.1, 126.0, 126.0, 125.9, 125.8, 104.4, 101.6, 101.6, 100.6, 98.8, 98.6, 96.3, 81.9, 81.2, 80.5, 80.4, 79.4, 78.9, 78.8, 77.4, 77.2, 76.9, 76.1, 76.0, 75.9, 75.7, 75.5, 75.4, 75.4, 75.3, 75.2, 75.1, 74.9, 74.8, 74.1, 74.0, 73.9, 73.6, 73.6, 73.4, 72.6, 72.2, 72.1, 72.1, 72.0, 71.8, 69.6, 69.1, 69.0, 68.8, 68.5, 68.4, 68.3, 68.1, 67.1, 59.8, 47.2 (C-1), 18.0. HRMS $[M+H]^+$ calculated 2013.906, found 2013.909.

α-D-mannose-(1->2)-β-D-mannose-(1->3)-β-D-mannose-(1->3)-α-L-rhamnose-(1->3)-1-deoxynojirimycin (**25**). Compound **24** (16 mg, 8 µmol) was co-evaporated with toluene (3x) and dissolved in dry THF (0.32 mL, 0.025 M) under N_2 and t-BuOH (0.1 mL, 1.1 mmol, 132 eq.) was added. Ammonia (±10 mL) was condensed at -60 °C while maintaining a dry atmosphere and Na (35 mg, 1.5 mmol, 192 eq.) was added, while using a glass stirring bar. After stirring for 10 min the Na was completely dissolved and compound **24** was added dropwise, the flask that contained **24** was washed with additional THF (0.2 mL). After stirring for 1 hour, the reaction mixture was quenched with AcOH and the ammonia was allowed to evaporate at room temperature, and the remaining solution was evaporated *in vacuo*. Size exclusion chromatography (HW40, 150 mM NH_4CO_3 in H_2O:MeCN, 9:1) then yielded pure compound **25** (4.2 mg, 5.3 µmol) in 66% as a salt with H_2CO_3-Et_3N. ^1H NMR (850 MHz, D_2O) δ 5.12 (d, *J* = 1.8 Hz, 1H), 5.10 (d, *J* = 1.8 Hz, 1H), 4.80 (dd, *J* = 2.4, 1.0 Hz, 2H), 4.23 (dd, *J* = 3.2, 2.0 Hz, 2H), 4.15 (dd, *J* = 3.0, 1.1 Hz, 1H), 4.13 (ddd, *J* = 10.2, 5.2, 2.4 Hz, 1H), 4.02 (dd, *J* = 3.4, 1.7 Hz, 1H), 3.99 (dq, *J* = 9.6, 6.2 Hz, 1H, H-5'), 3.89 (dd, *J* = 4.7, 3.3 Hz, 1H), 3.89 – 3.86 (m, 3H), 3.85 (dd, *J* = 2.3, 1.0 Hz, 2H), 3.84 – 3.83 (m, 1H), 3.83 – 3.81 (m, 3H), 3.81 – 3.79 (m, 1H), 3.73 – 3.69 (m, 3H), 3.68 – 3.64 (m, 3H), 3.62 (dt, *J* = 12.2, 10.2 Hz, 3H), 3.59 – 3.53 (m, 4H), 3.52 (t, *J* = 8.7 Hz, 1H, H-4'), 3.42 (dd, *J* = 12.6, 5.2 Hz, 1H, H-1), 3.35 – 3.30 (m, 3H), 3.15 (ddd, *J* = 10.4, 5.2, 3.1 Hz, 1H), 3.12 (q, *J* = 7.3 Hz, 6H, Et_3N-H^+), 2.94 (dd, *J* = 12.6, 11.6 Hz, 1H, H-1), 1.20 (t, *J* = 7.3 Hz, 12H, H-6', Et_3N-H^+). ^{13}C NMR (214 MHz, D_2O) δ 162.9, 117.0, 115.6, 101.2, 101.1, 100.8, 96.6, 82.0, 79.3, 79.1, 76.9, 76.3, 75.6, 73.6, 72.3, 71.0, 70.3, 70.2, 70.0, 68.9, 67.6, 67.5, 66.9, 66.6, 66.3, 65.1, 61.1, 61.0, 60.8, 60.0, 57.6, 46.7, 45.7 (C-1), 16.5, 8.2. HRMS $[M+H]^+$ calculated 796.3081, found 796.3087.

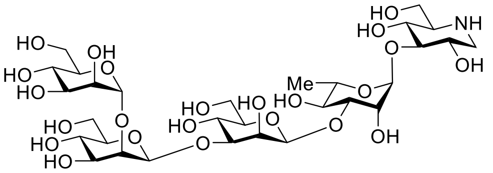

References

(1) McNicholas, S., Potterson, E., Wilson, K.S., Noble, M.E.M. Presenting your structures: the CCP4mg molecular-graphics software. *Acta Cryst.* **2011**, D67, 386-394

(2) Jumper, J.; Evans, R.; Pritzel, A.; Green, T.; Figurnov, M.; Ronneberger, O.; Tunyasuvunakool, K.; Bates, R.; Žídek, A.; Potapenko, A.; Bridgland, A.; Meyer, C.; Kohl, S. A. A.; Ballard, A. J.; Cowie, A.; Romera-Paredes, B.; Nikolov, S.; Jain, R.; Adler, J.; Back, T.; Petersen, S.; Reiman, D.; Clancy, E.; Zielinski, M.; Steinegger, M.; Pacholska, M.; Berghammer, T.; Bodenstein, S.; Silver, D.; Vinyals, O.; Senior, A. W.; Kavukcuoglu, K.; Kohli, P.; Hassabis, D. Highly accurate protein structure prediction with AlphaFold. *Nature* **2021**, *596*, 583-589

(3) Winter, G.; Waterman, D. G.; Parkhurst, J. M.; Brewster, A. S.; Gildea, R. J.; Gerstel, M.; Fuentes-Montero, L.; Vollmar, M.; Michels-Clark, T.; Young, I. D.; Sauter, N. K.; Evans, G. *DIALS*:

implementation and evaluation of a new integration package. *Acta Crystallogr. Sect. Struct. Biol.* **2018**, *74*, 85-97.

(4) Gildea, R. J.; Beilsten-Edmands, J.; Axford, D.; Horrell, S.; Aller, P.; Sandy, J.; Sanchez-Weatherby, J.; Owen, C. D.; Lukacik, P.; Strain-Damerell, C.; Owen, R. L.; Walsh, M. A.; Winter, G. Xia2.multiplex: a multi-crystal data-analysis pipeline. *Acta Crystallogr. Sect. Struct. Biol.* **2022**, *78*, 752-769.

(5) Evans, P. R.; Murshudov, G. N. How good are my data and what is the resolution? *Acta Crystallogr. D Biol. Crystallogr.* **2013**, *69*, 1204-1214.

(6) McCoy, A. J.; Grosse-Kunstleve, R. W.; Adams, P. D.; Winn, M. D.; Storoni, L. C.; Read, R. J. Phaser crystallographic software. *J. Appl. Crystallogr.* **2007**, *40*, 658-674.

(7) Emsley, P.; Cowtan, K. Coot: model-building tools for molecular graphics. *Acta Crystallogr. D Biol. Crystallogr.* **2004**, *60*, 2126-2132.

(8) Murshudov, G. N.; Skubák, P.; Lebedev, A. A.; Pannu, N. S.; Steiner, R. A.; Nicholls, R. A.; Winn, M. D.; Long, F.; Vagin, A. A. *REFMAC* 5 for the refinement of macromolecular crystal structures. *Acta Crystallogr. D Biol. Crystallogr.* **2011**, *67*, 355-367.

(9) Krissinel, E.; Lebedev, A. A.; Uski, V.; Ballard, C. B.; Keegan, R. M.; Kovalevskiy, O.; Nicholls, R. A.; Pannu, N. S.; Skubák, P.; Berrisford, J.; Fando, M.; Lohkamp, B.; Wojdyr, M.; Simpkin, A. J.; Thomas, J. M. H.; Oliver, C.; Vonrhein, C.; Chojnowski, G.; Basle, A.; Purkiss, A.; Isupov, M. N.; McNicholas, S.; Lowe, E.; Triviño, J.; Cowtan, K.; Agirre, J.; Rigden, D. J.; Uson, I.; Lamzin, V.; Tews, I.; Bricogne, G.; Leslie, A. G. W.; Brown, D. G. CCP 4 Cloud for structure determination and project management in macromolecular crystallography. *Acta Crystallogr. Sect. Struct. Biol.* **2022**, *78*, 1079-1089.

(10) Mori, M.; Ito, Y.; Ogawa, T. Total synthesis of the mollu-series glycosyl ceramides α-D-Manp-(1→3)-β-D-Manp-(1→4)-β-D-Glcp-(1→1)-Cer and α-D-Manp-(1→3)-[β-D-Xylp-(1→2)]-β-D-Manp-(1→4)-β-D-Glcp-(1→1)-Cer. *Carbohydr. Res.* **1990**, *195*, 199-224.

(11) Heuckendorff, M., Bendix, J., Pedersen, C. M., Bols, M. β-Selective mannosylation with a 4,6-silylene-tethered thiomannosyl donor. *Org. Lett.* **2024**, *16*, 1116-1119.

(12) Armstrong, Z.; Kuo, C.-L.; Lahav, D.; Liu, B.; Johnson, R.; Beenakker, T. J. M.; de Boer, C.; Wong, C.-S.; van Rijssel, E. R.; Debets, M. F.; Florea, B. I.; Hissink, C.; Boot, R. G.; Geurink, P. P.; Ovaa, H.; van der Stelt, M.; van der Marel, G. M.; Codée, J. D. C.; Aerts, J. M. F. G.; Wu, L.; Overkleeft, H. S.; Davies, G. J. Manno-epi-cyclophellitols enable activity-based protein profiling of human α-mannosidases and discovery of new Golgi mannosidase II inhibitors. *J. Am. Chem. Soc.* **2020**, *142*, 13021-13029.

(13) Crich, D., Li, W.; Li, H. Direct chemical synthesis of the β-mannans: linear and block syntheses of the alternating β-(1→3)-β-(1→4)-mannan common to *Rhodotorula glutinis*, *Rhodotorula mucilaginosa*, and *Leptospira biflexa*. *J. Am. Chem. Soc.* **2004**, *126*, 15081-15086.

(14) Ofman, T. P.; Küllmer, F.; van der Marel, G. A.; Codée, J. D. C.; Overkleeft, H. S. An orthogonally protected cyclitol for the construction of nigerose- and dextran-mimetic cyclophellitols. *Org. Lett.* **2021**, *23*, 9516-9519.

(15) Li, H.; Mo, K.-F.; Wang, Q.; Stover, C. K.; DiGiandomenico, A.; Boons, G.-J. Epitope mapping of monoclonal antibodies using synthetic oligosaccharides uncovers novel aspects of immune recognition of the Psl exopolysaccharide of *Pseudomonas aeruginosa*. *Chem. Eur. J.* **2013**, *19*, 17425-17431.

(16) Demeter, F.; Chang, M. D.-T.; Lee, Y.-C.; Borbás, A.; Herczeg, M. An efficient synthesis of the pentasaccharide repeating unit of Pseudomonas aeruginosa Psl exopolysaccharide. *Synlett* **2020**, *31*, 469-474.

NMR Spectra

Phenyl 3-(8-azidooctyl)-4,6-O-ditertbutylsilyl-1-thio-α-d-mannopyranose (26)

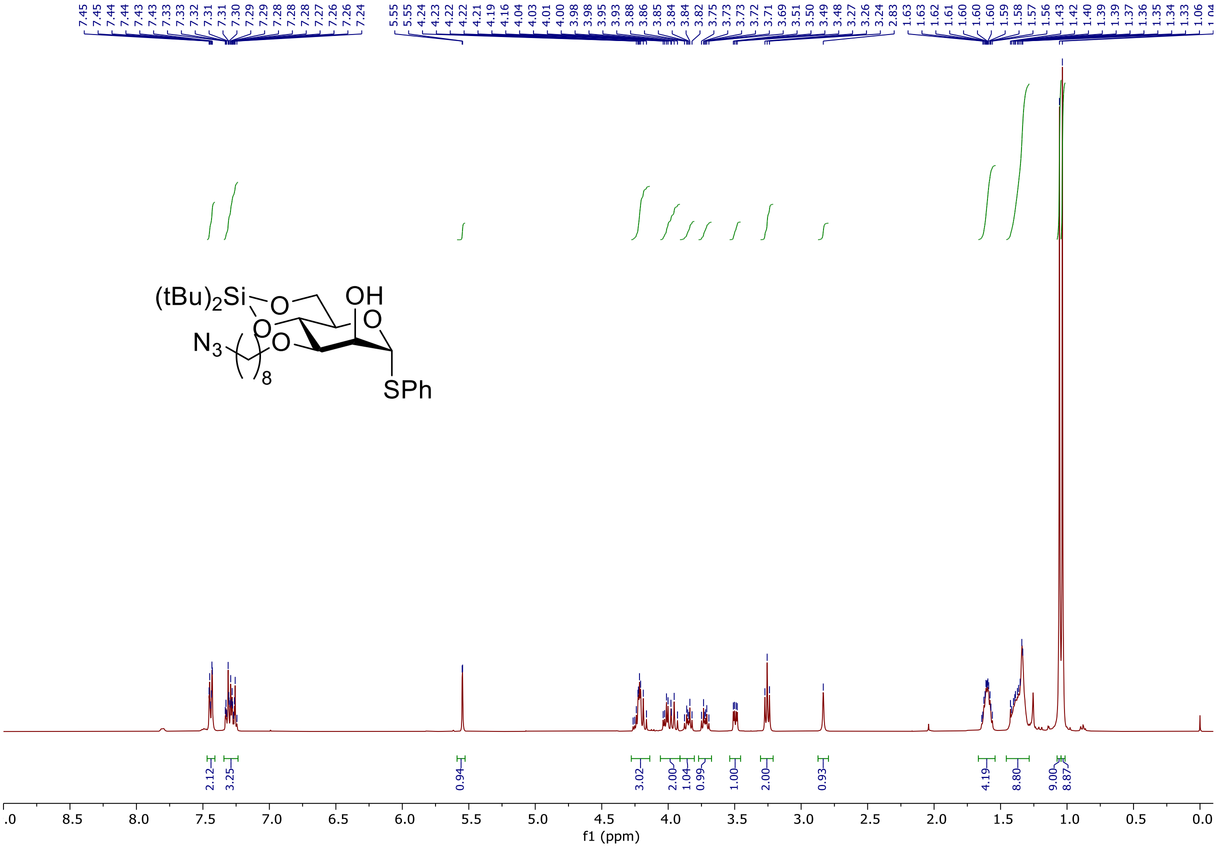

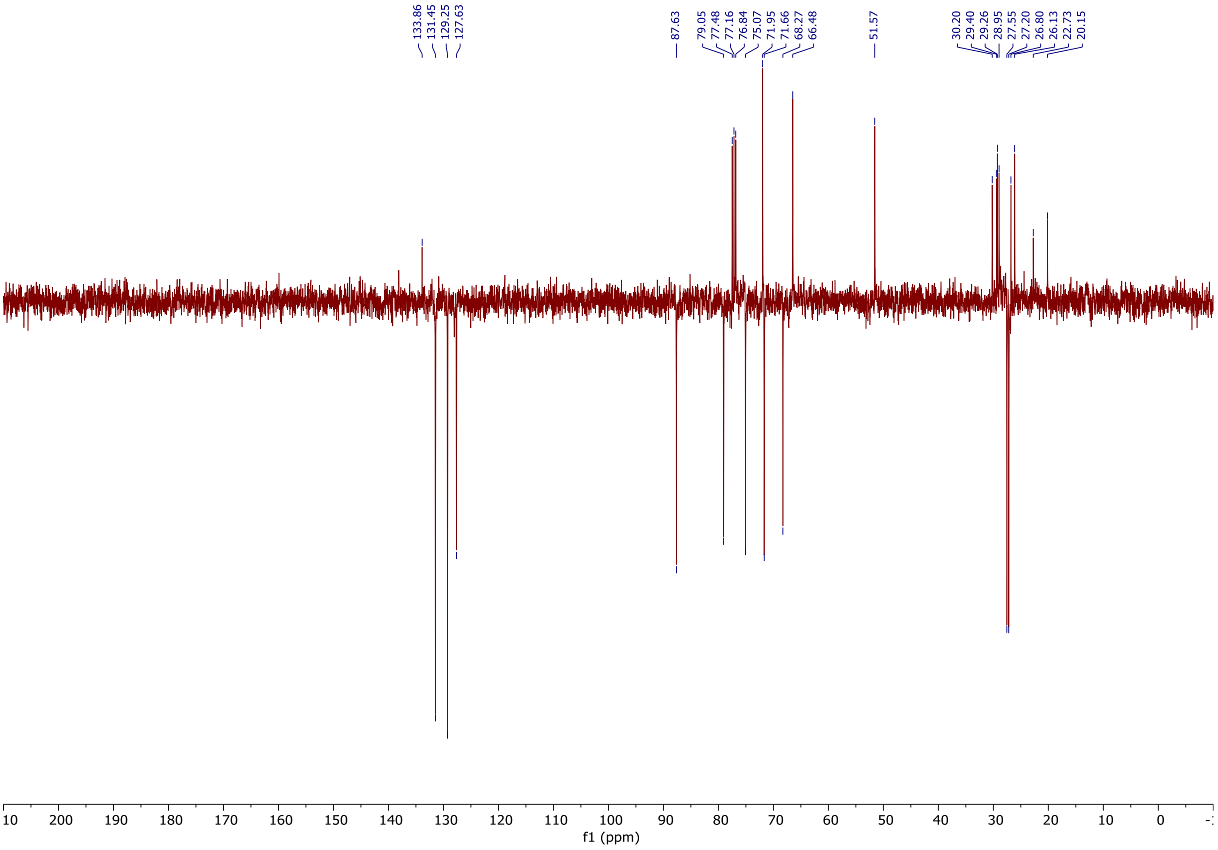

Phenyl 2-*O*-(2,3,4,6-tetra-*O*-acetyl-β-D-mannopyranosyl)-3-*O*-(8-azidooctyl)-4,6-*O*-ditertbutylsilyl-1-thio-α-D-mannopyranose (27)

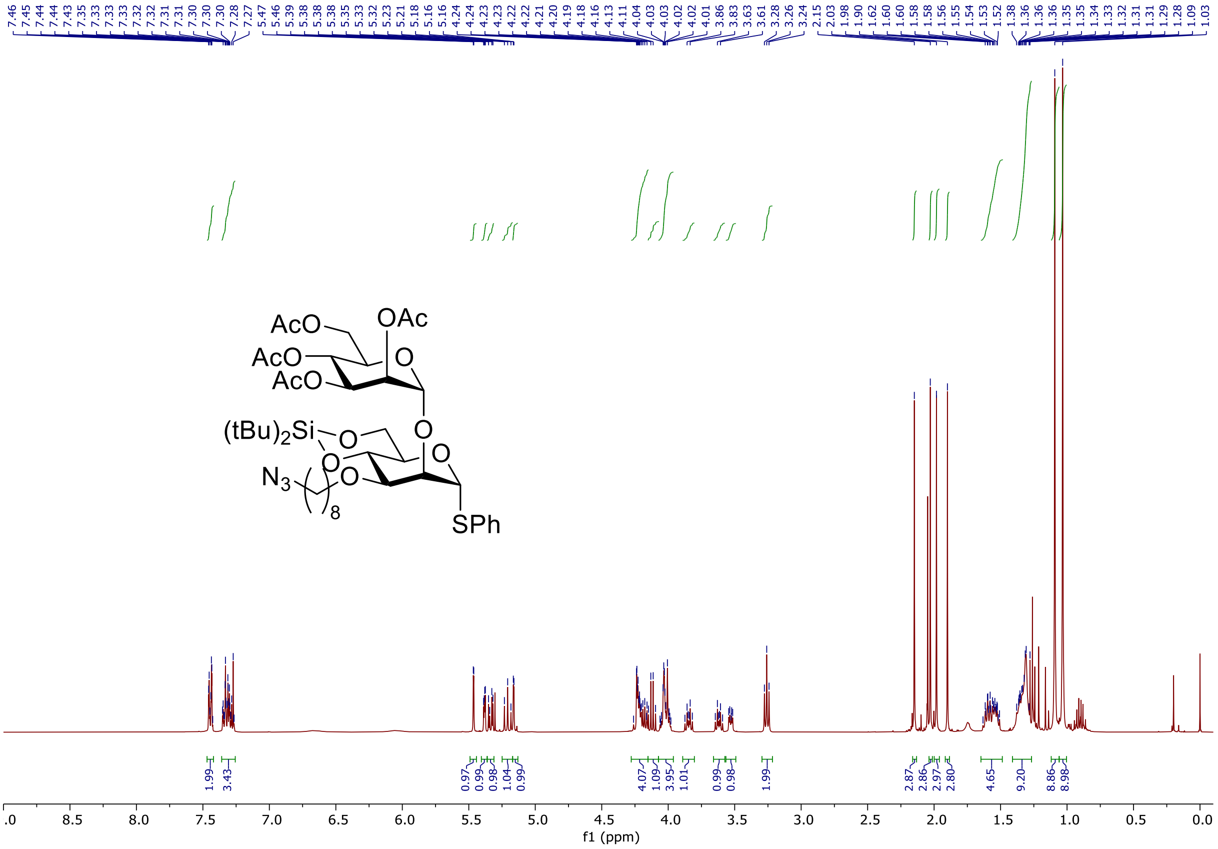

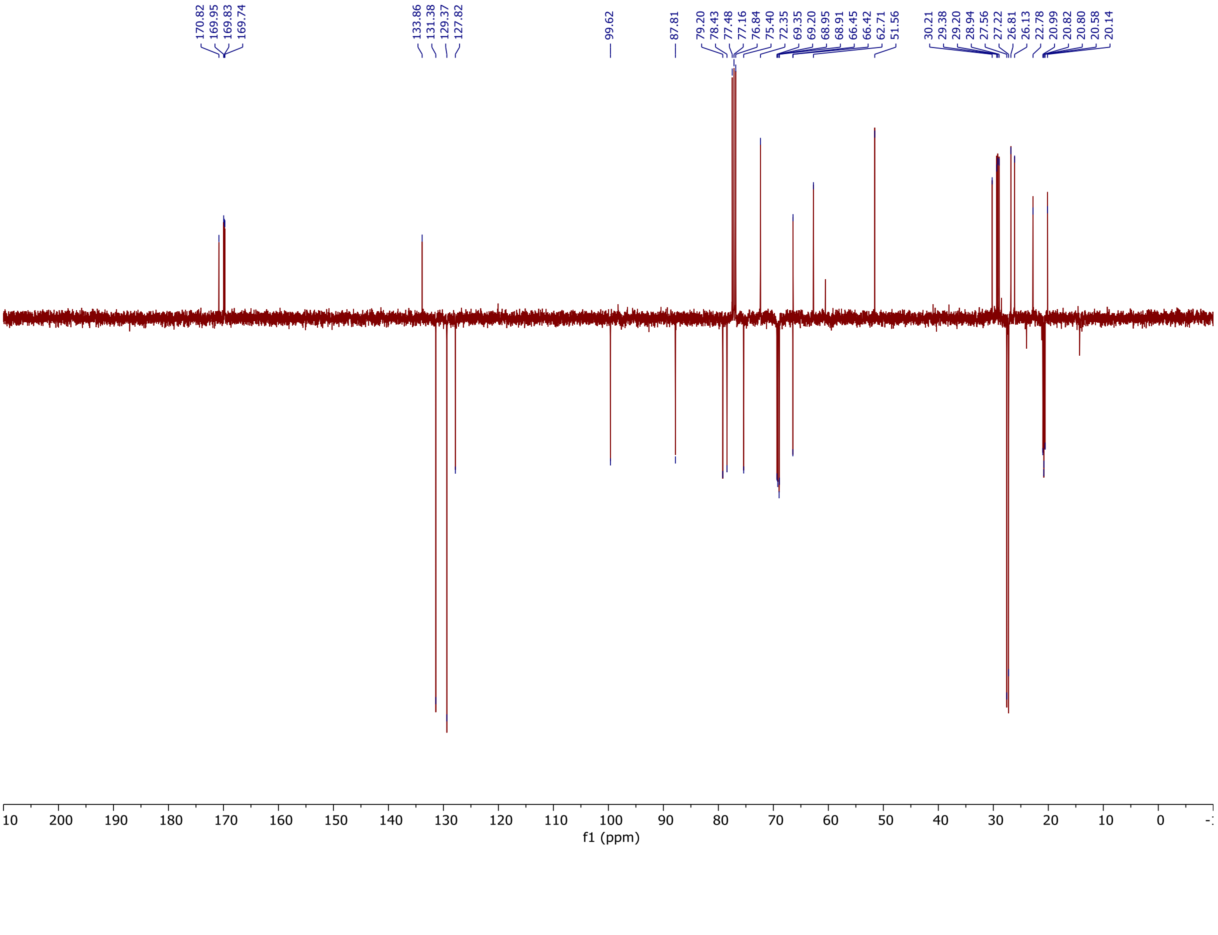

2,3-*O*-benzyl-4,6-*O*-ditertbutylsilyl-mannosecyclophellitolalkene (29)

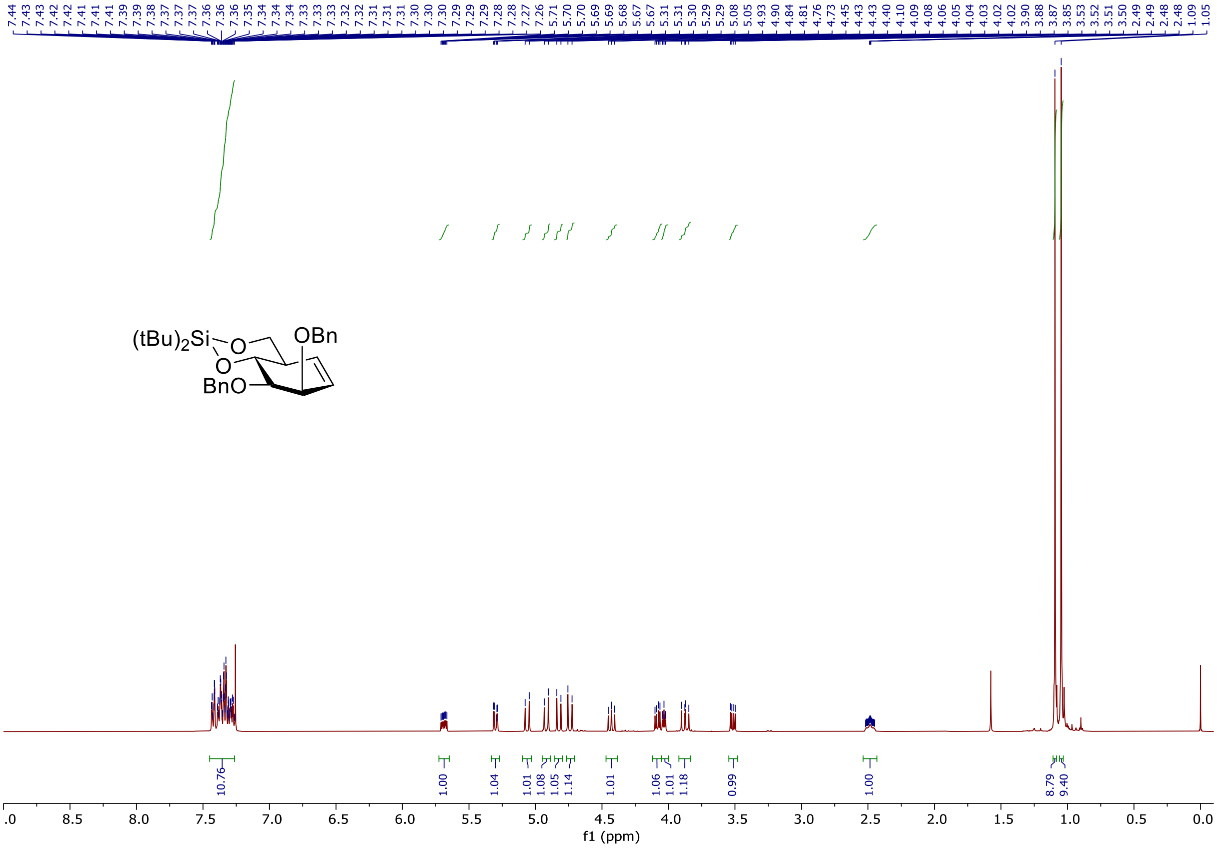

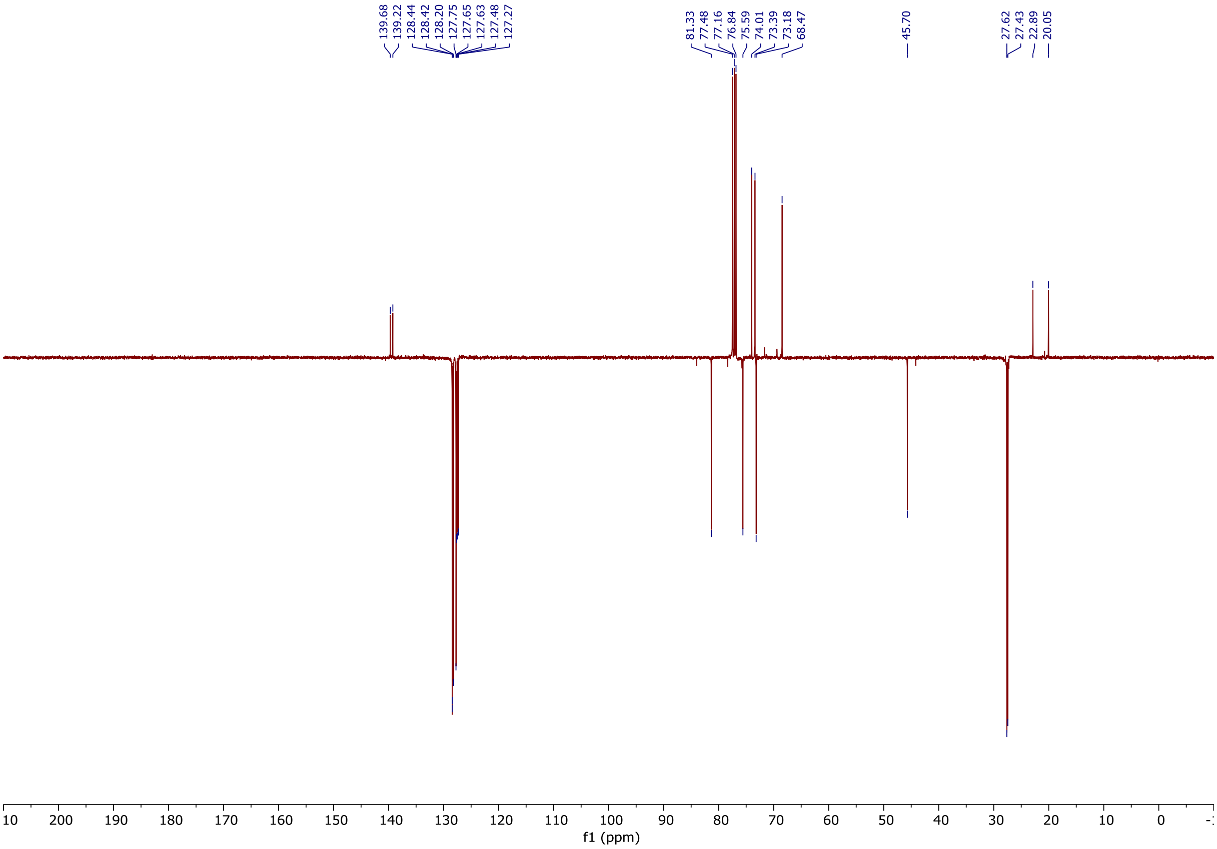

4,6-*O*-ditertbutylsilyl-mannocyclophellitolalkene (30)

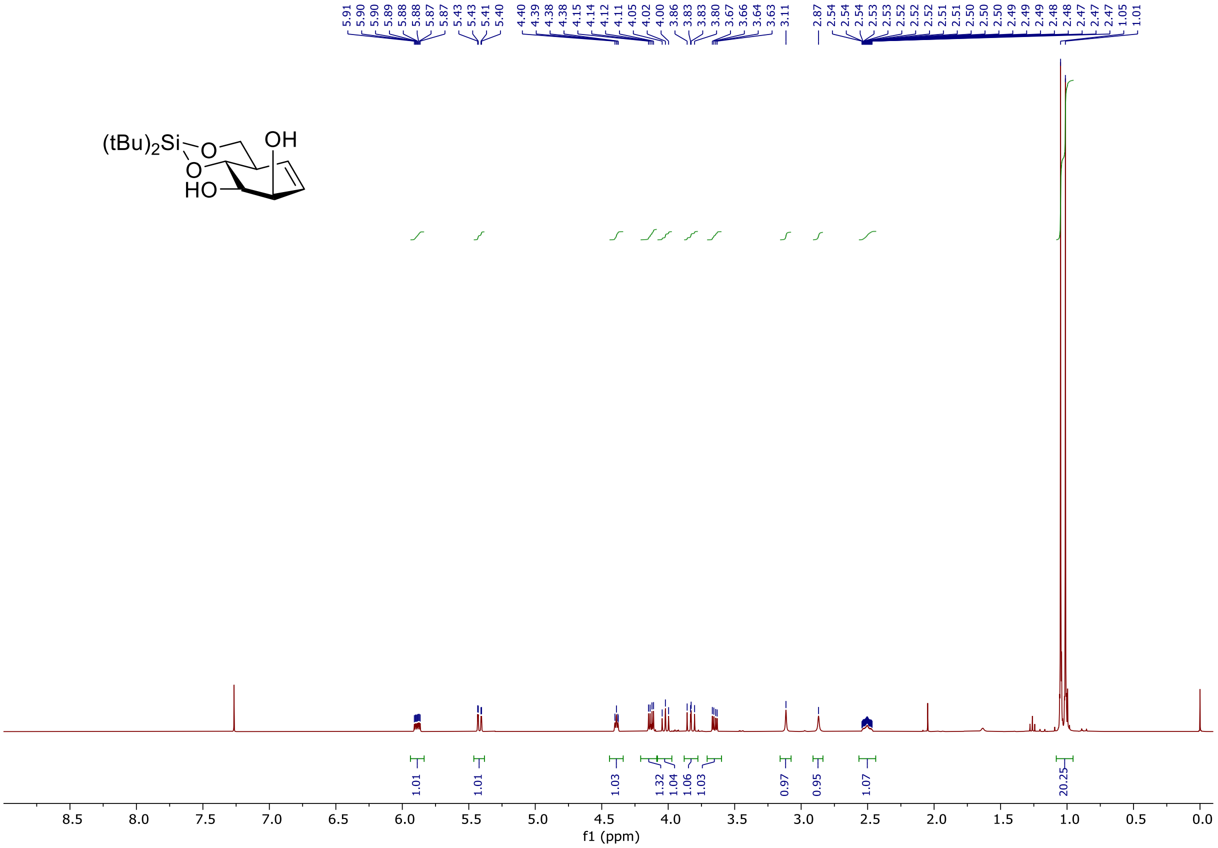

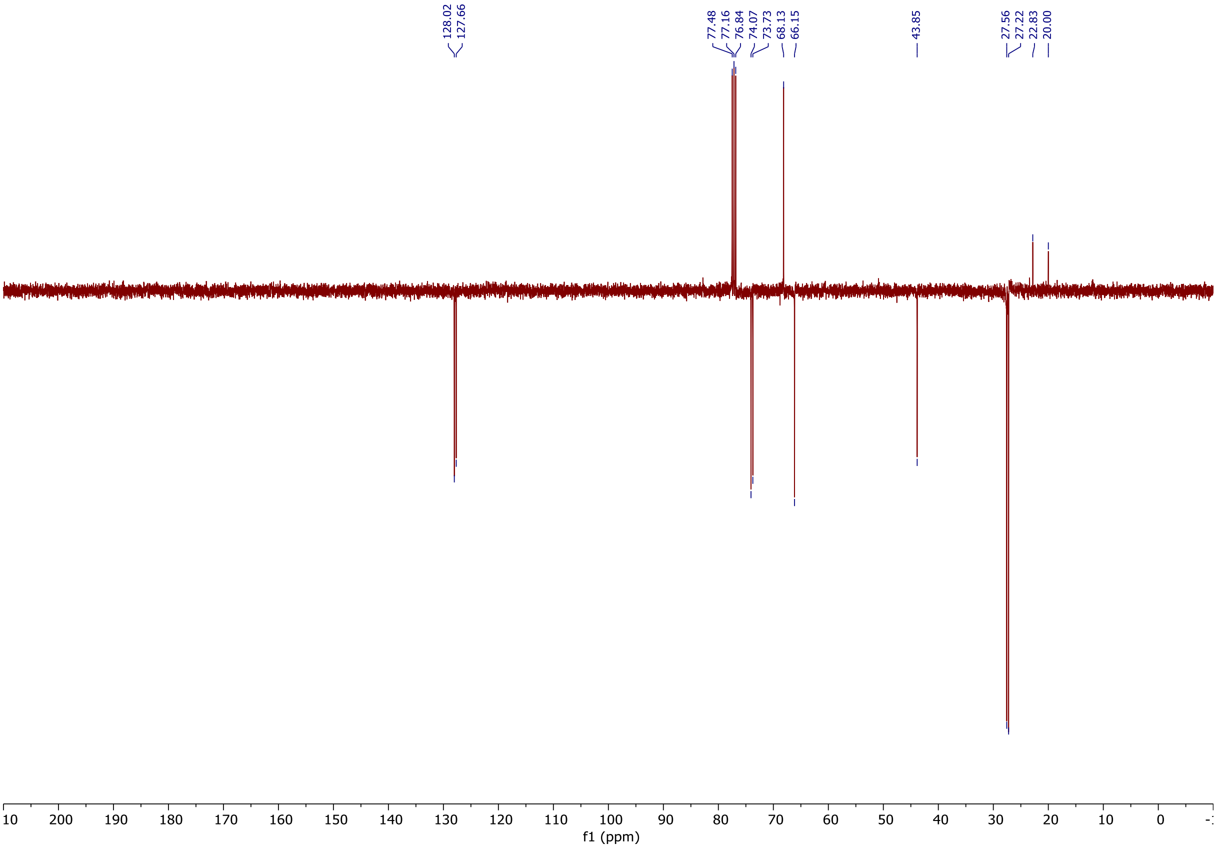

2-*O*-benzoyl-4,6-*O*-ditertbutylsilyl-mannocyclophellitolalkene (31)

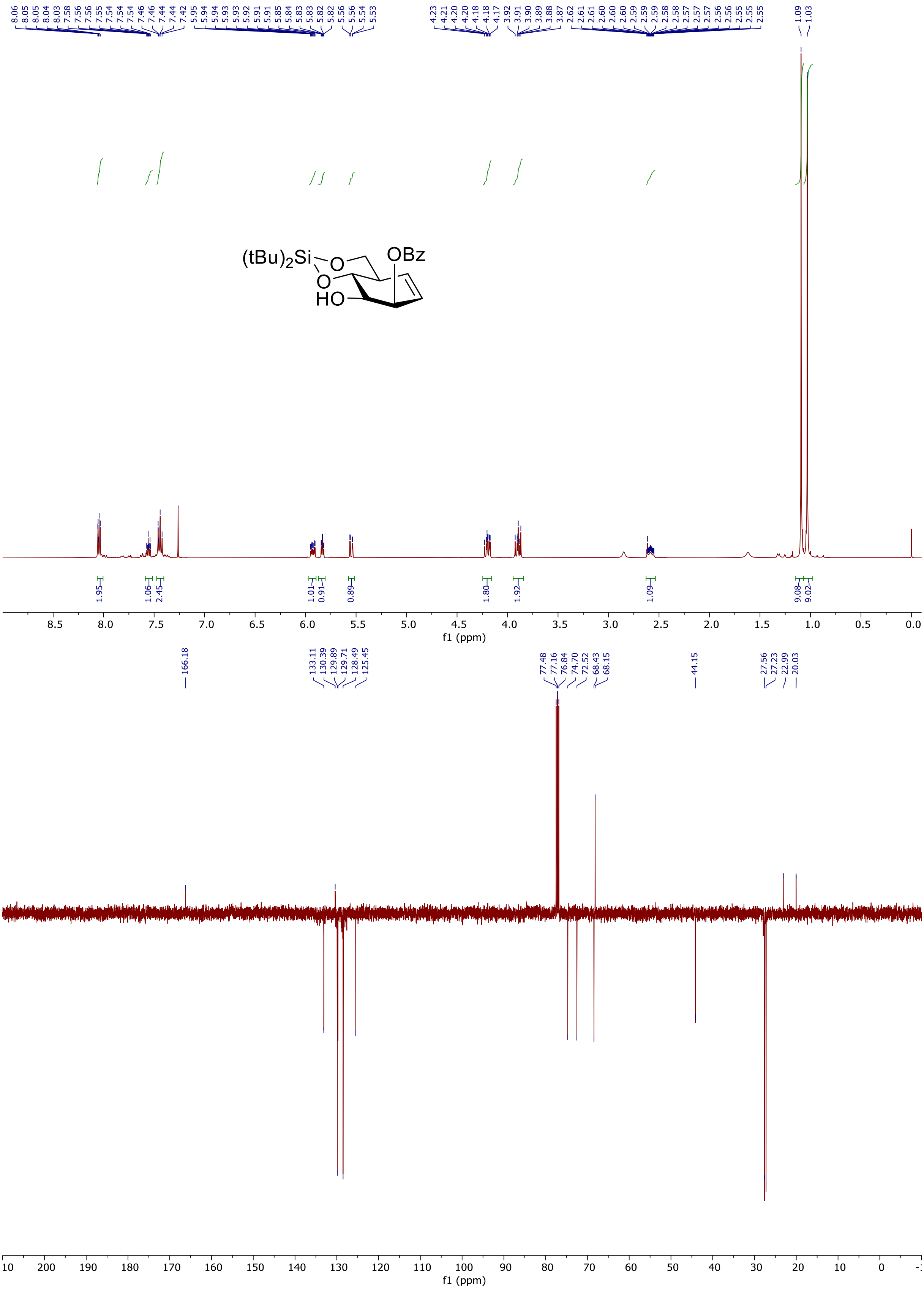

2-*O*-benzoyl-3-*O*-(2-*O*-(2,3,4,6-tetra-*O*-acetyl-α-D-mannopyranosyl)-3-*O*-(8-azidooctyl)-β-D-mannopyranosyl)-4,6-*O*-ditertbutylsilyl-mannocyclophellitolalkene (30)

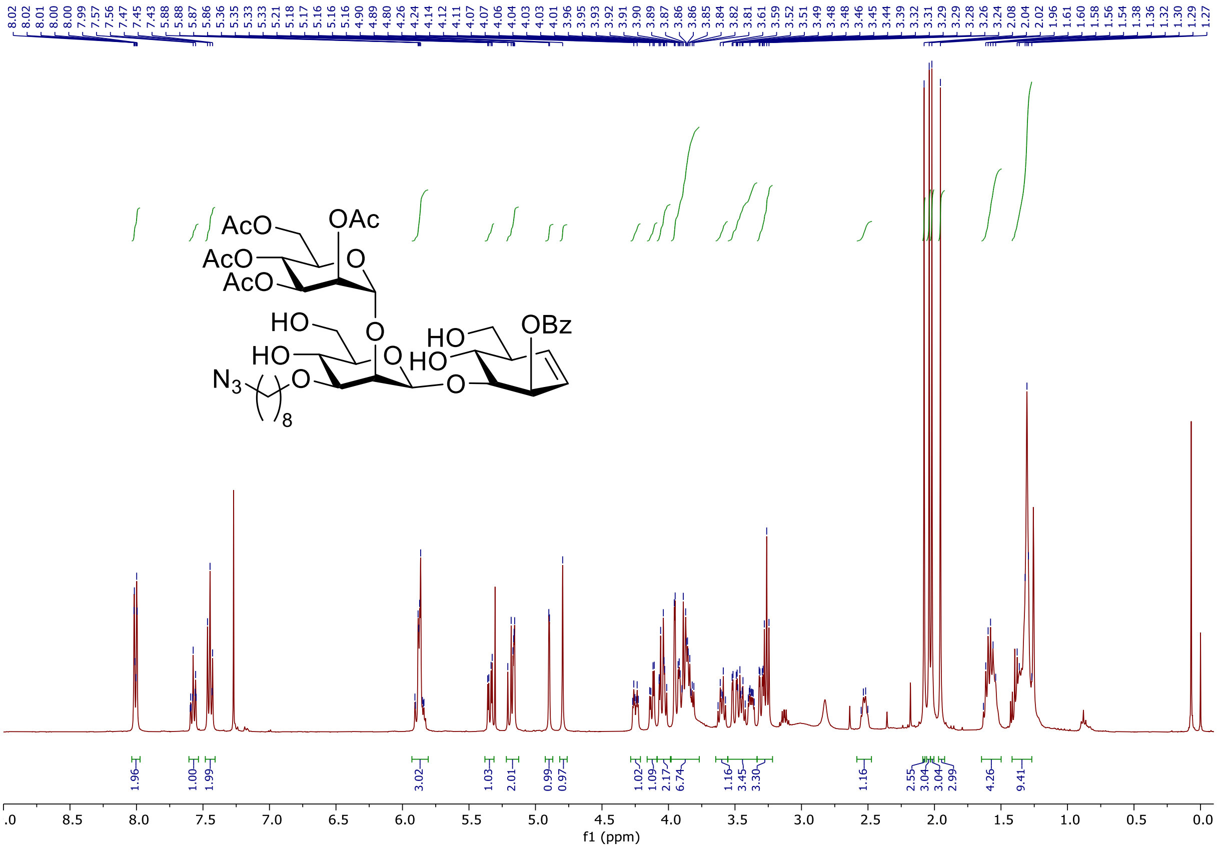

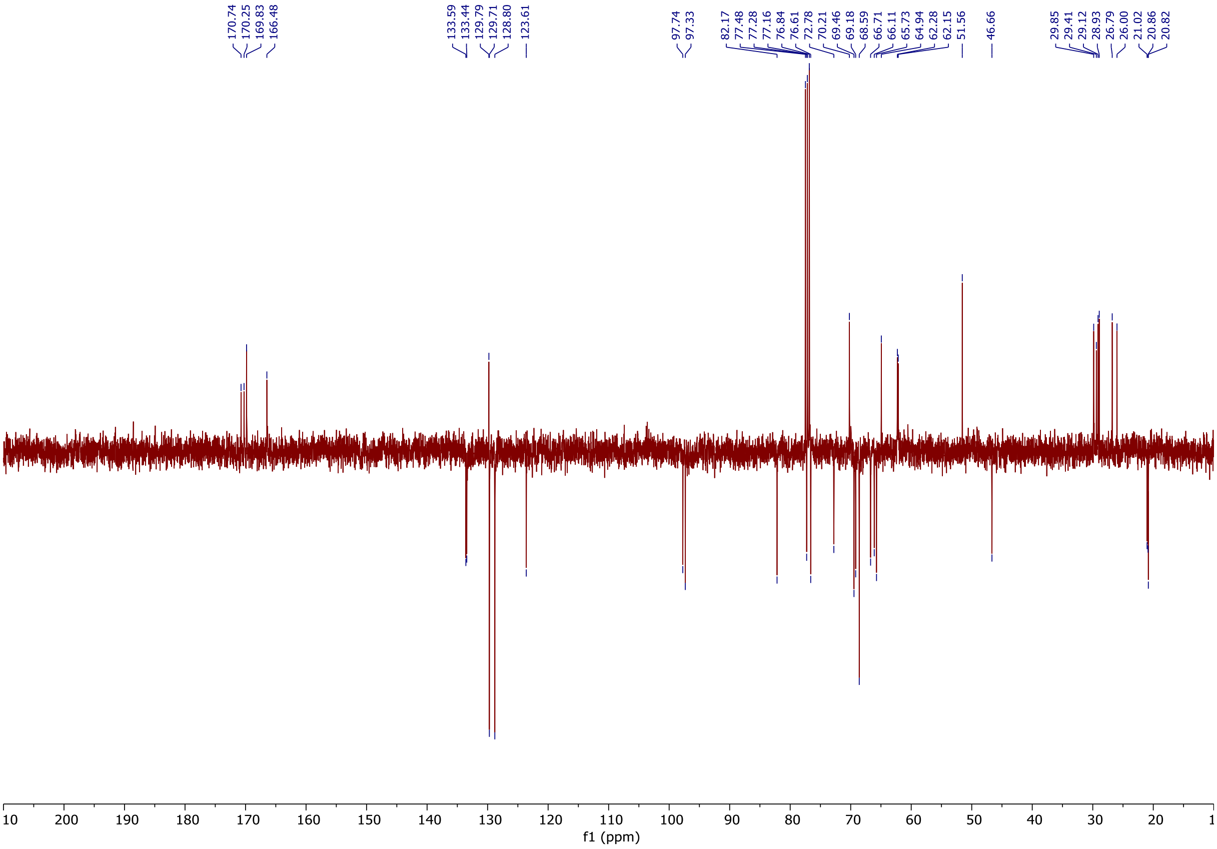

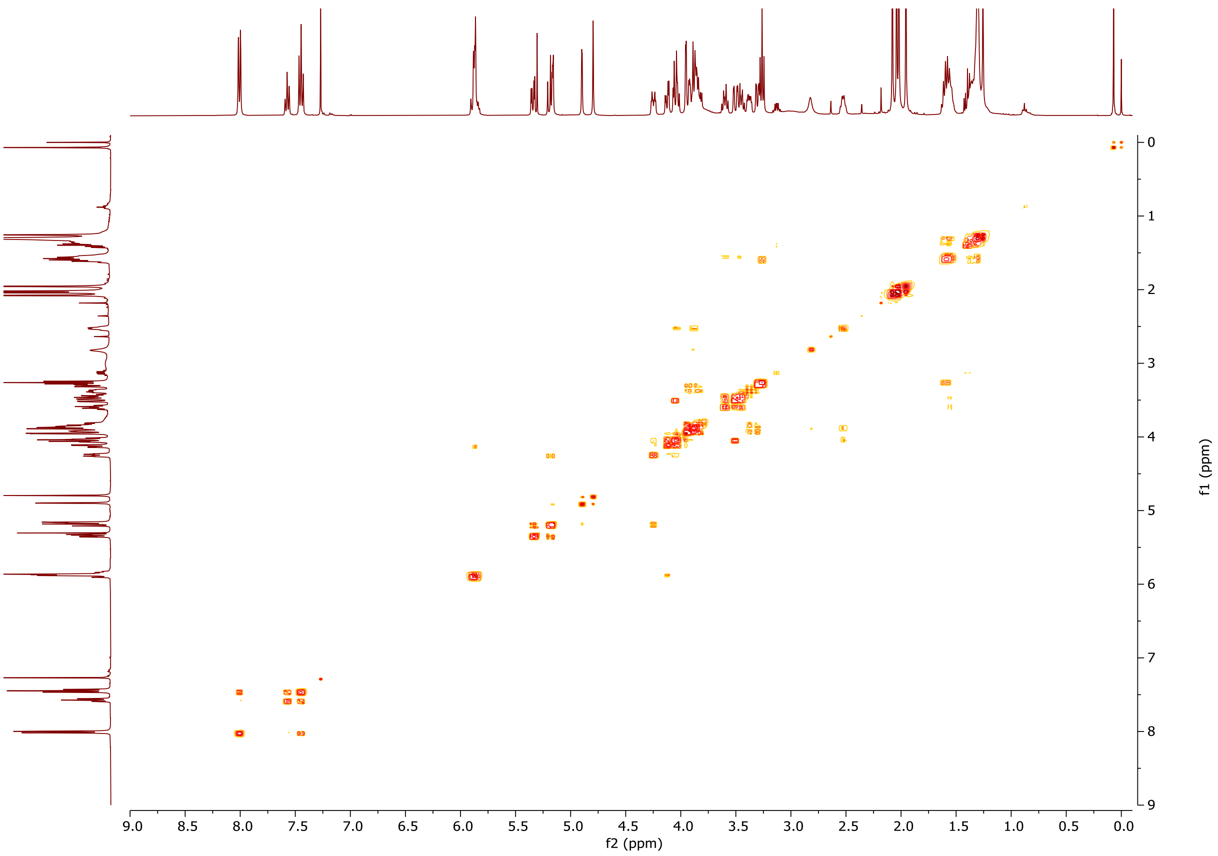
f2 (ppm)
f1 (ppm)

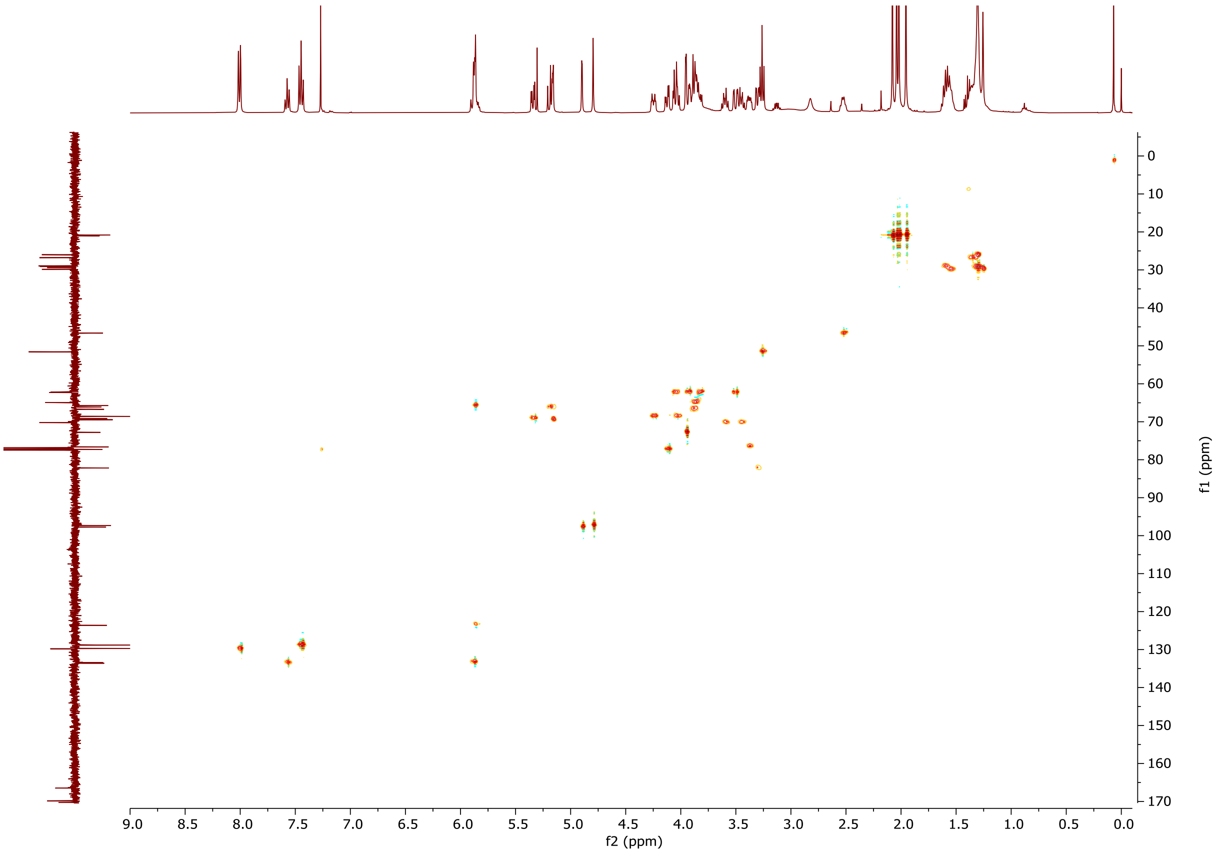
f2 (ppm)
f1 (ppm)

3-*O*-(2-*O*-(α-D-mannopyranosyl)-3-*O*-(8-azidooctyl)-β-D-mannopyranosyl)-β-mannocyclophellitol (1)

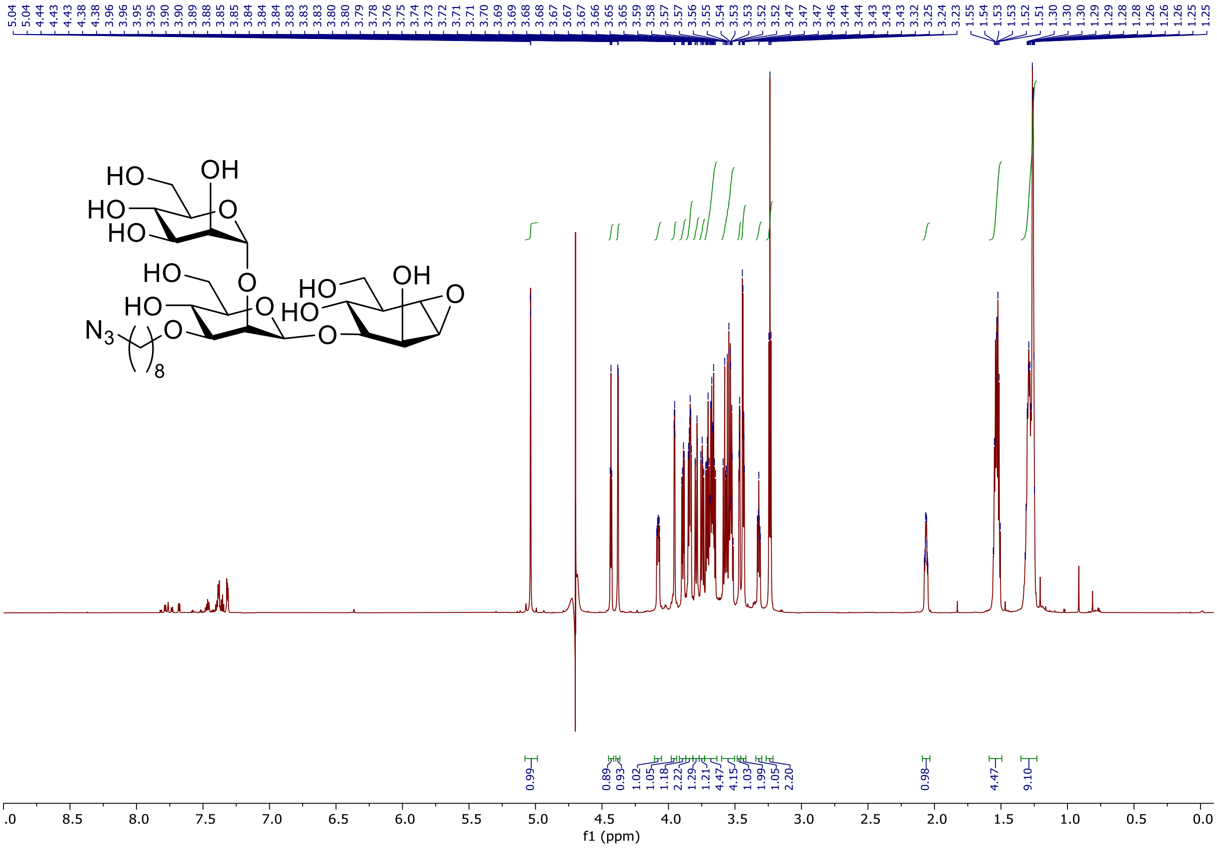

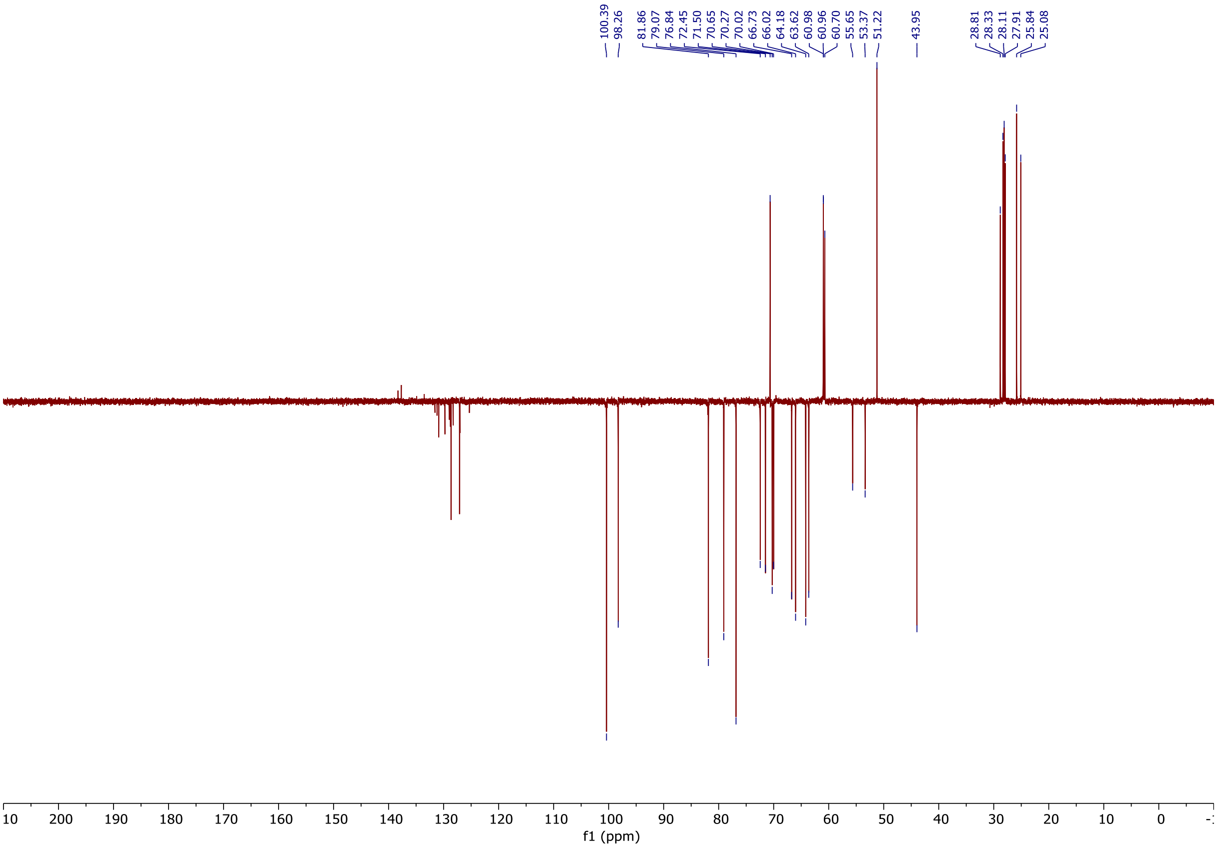

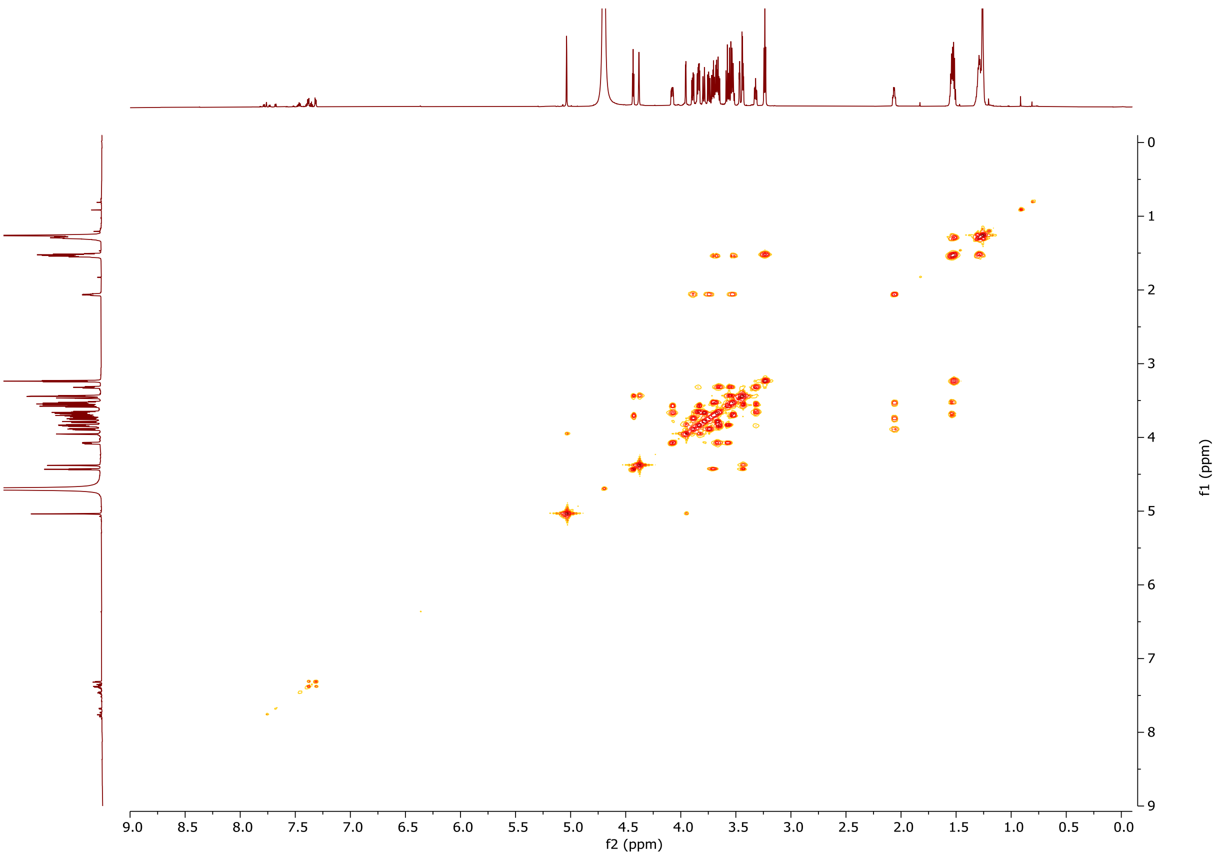
f1 (ppm)
f2 (ppm)

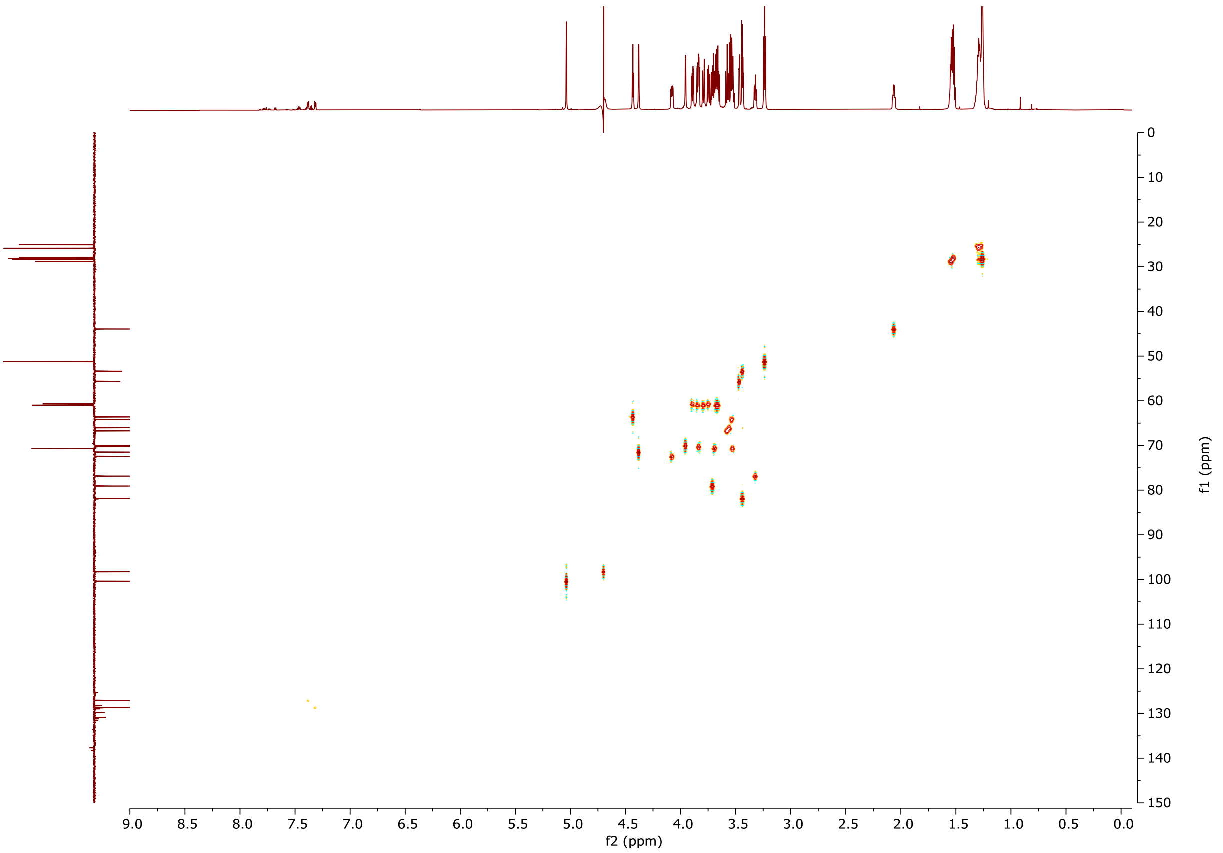
f1 (ppm)
f2 (ppm)

1,2:5,6-do-*O*-isopropylidene-3-*O*-(8-azido-octyl) α-D-glucofuranose (35)

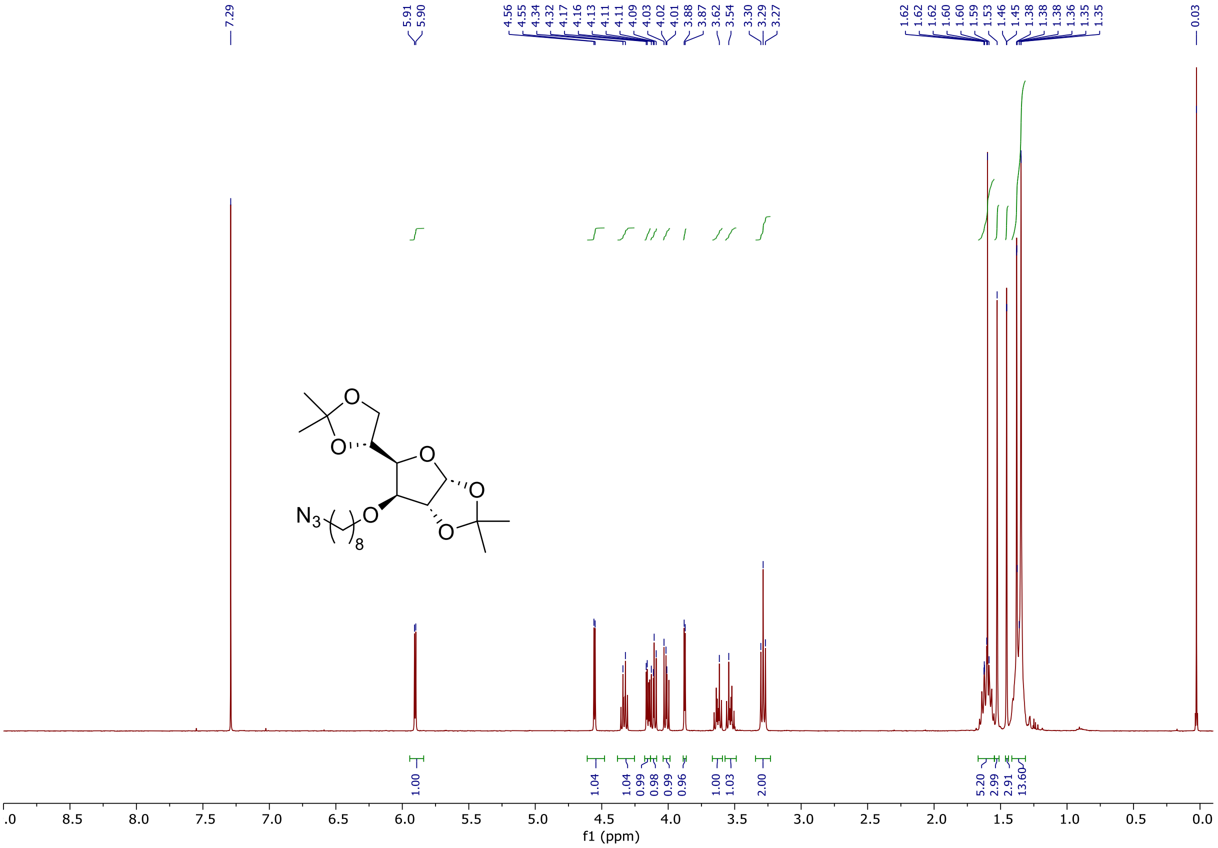

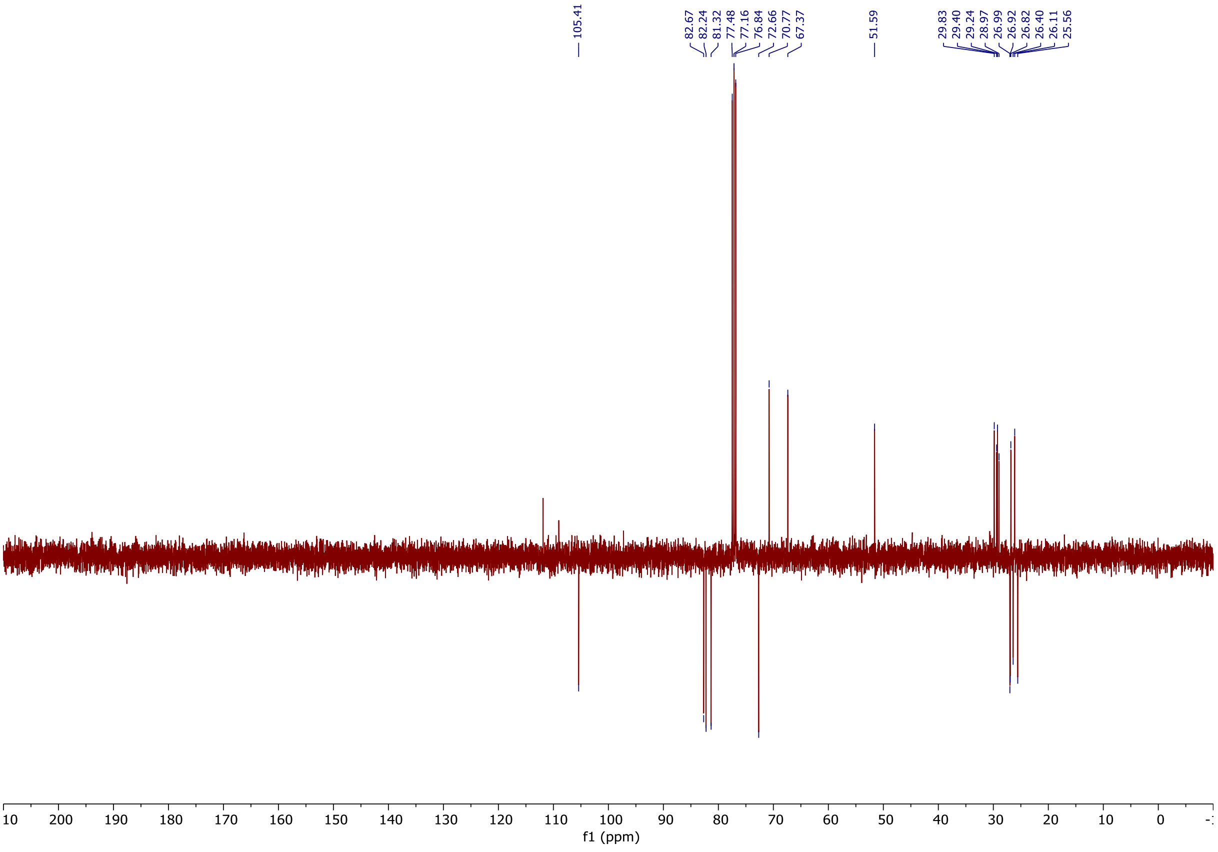

1,2,4,6-*O*-benzoyl-3-*O*-(8-azido-octyl)-D-glucopyranose (36)

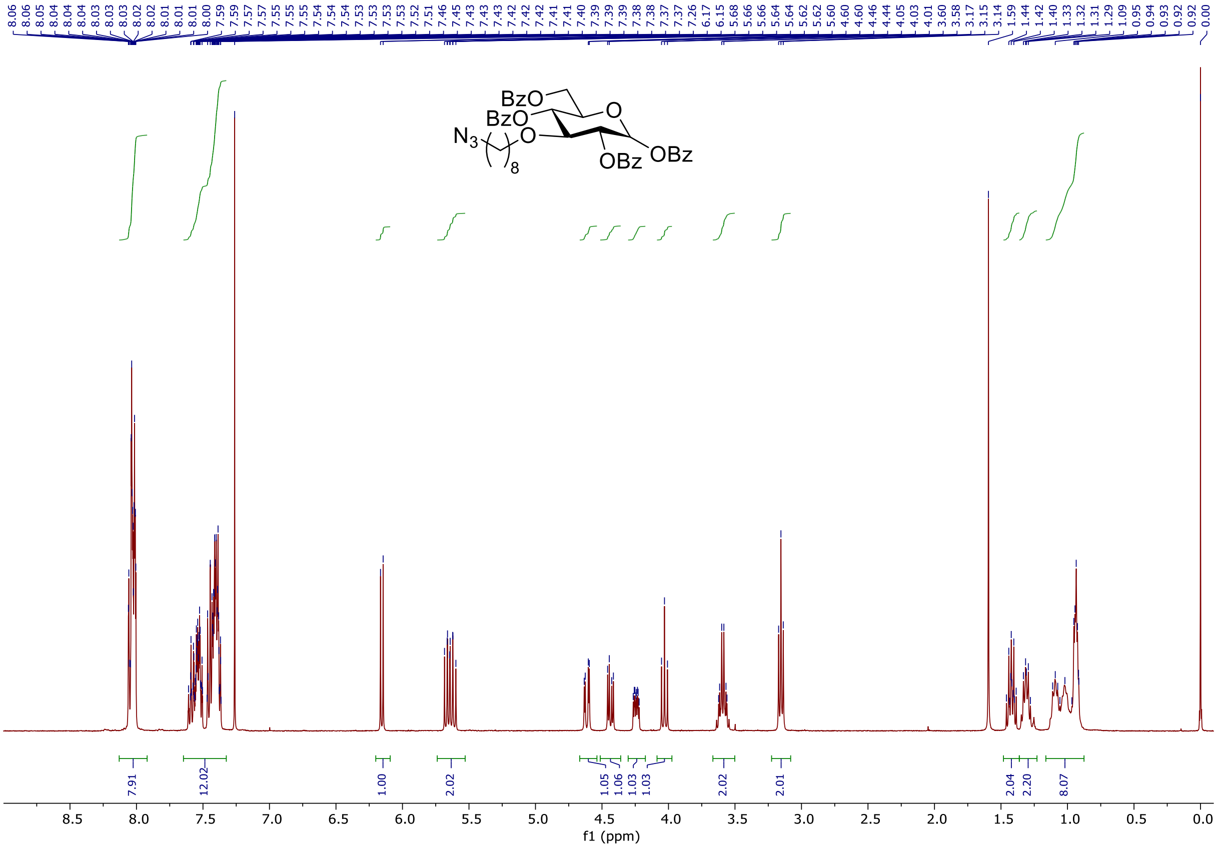

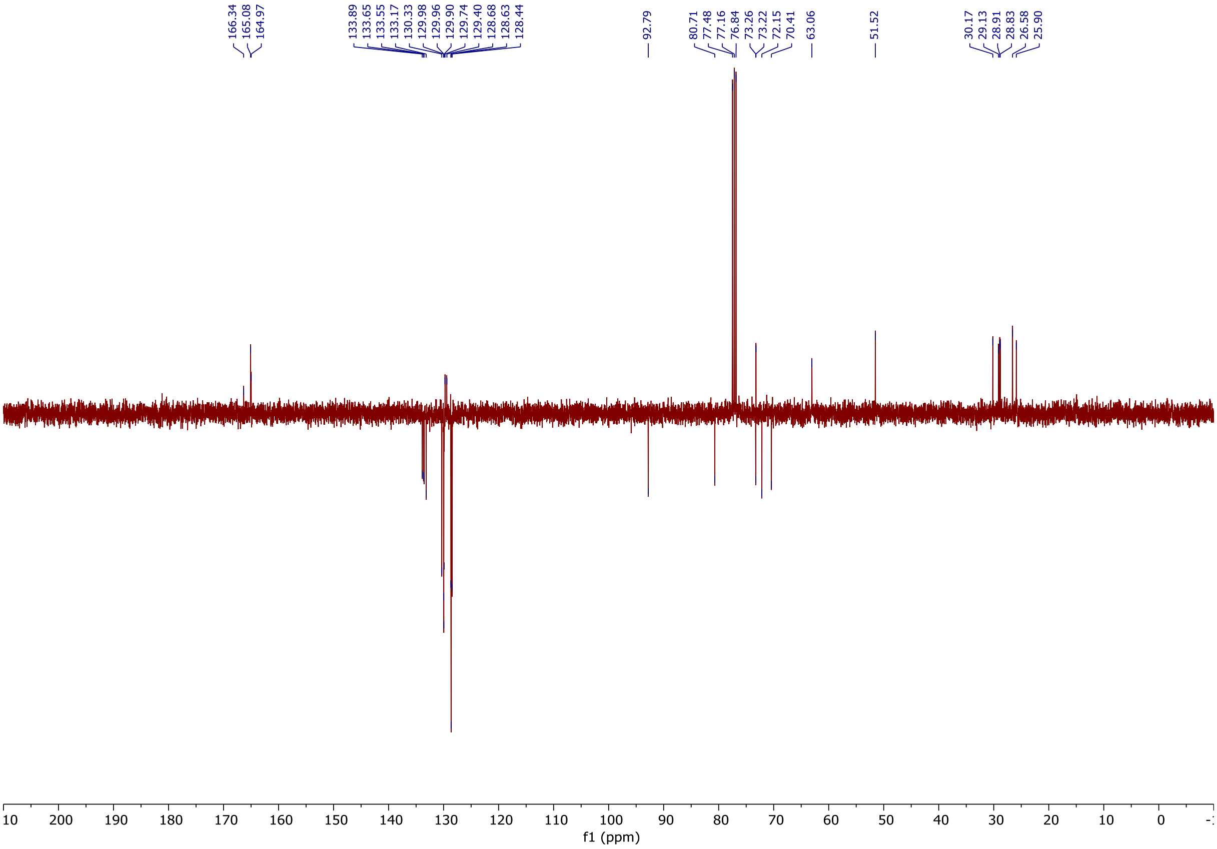

2,4,6-*O*-benzoyl-3-*O*-(8-azido-octyl)-D-glucopyranose (36a)

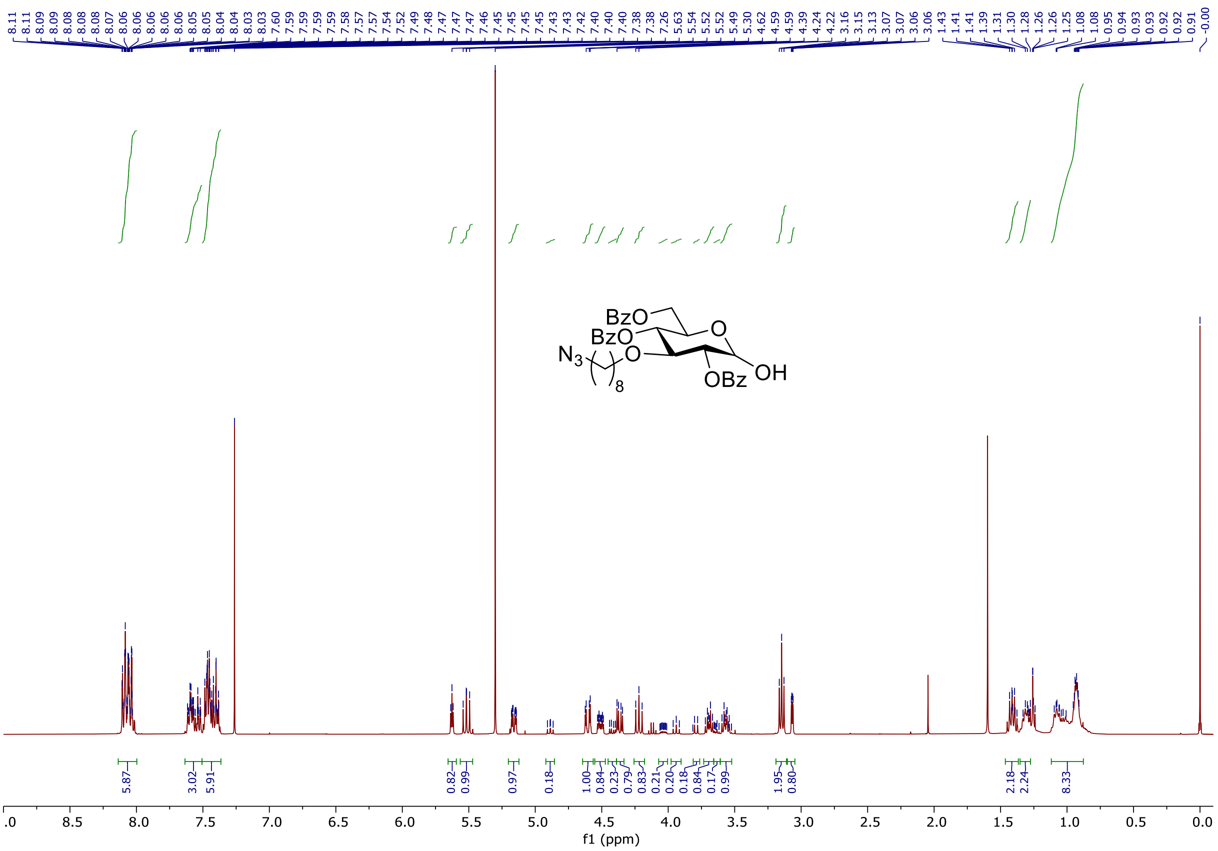

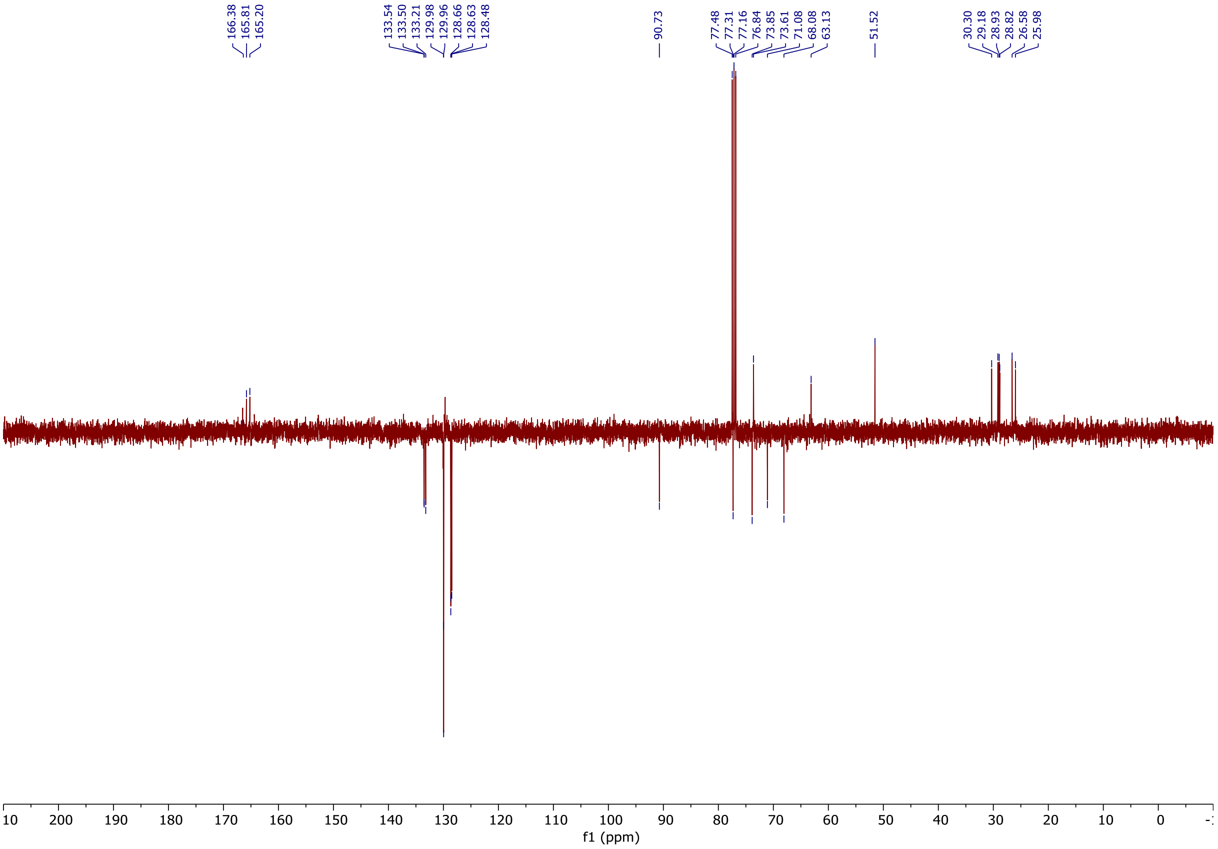

2,4,6-*O*-benzoyl-3-*O*-(8-azido-octyl)-D-glucopyranose (37)

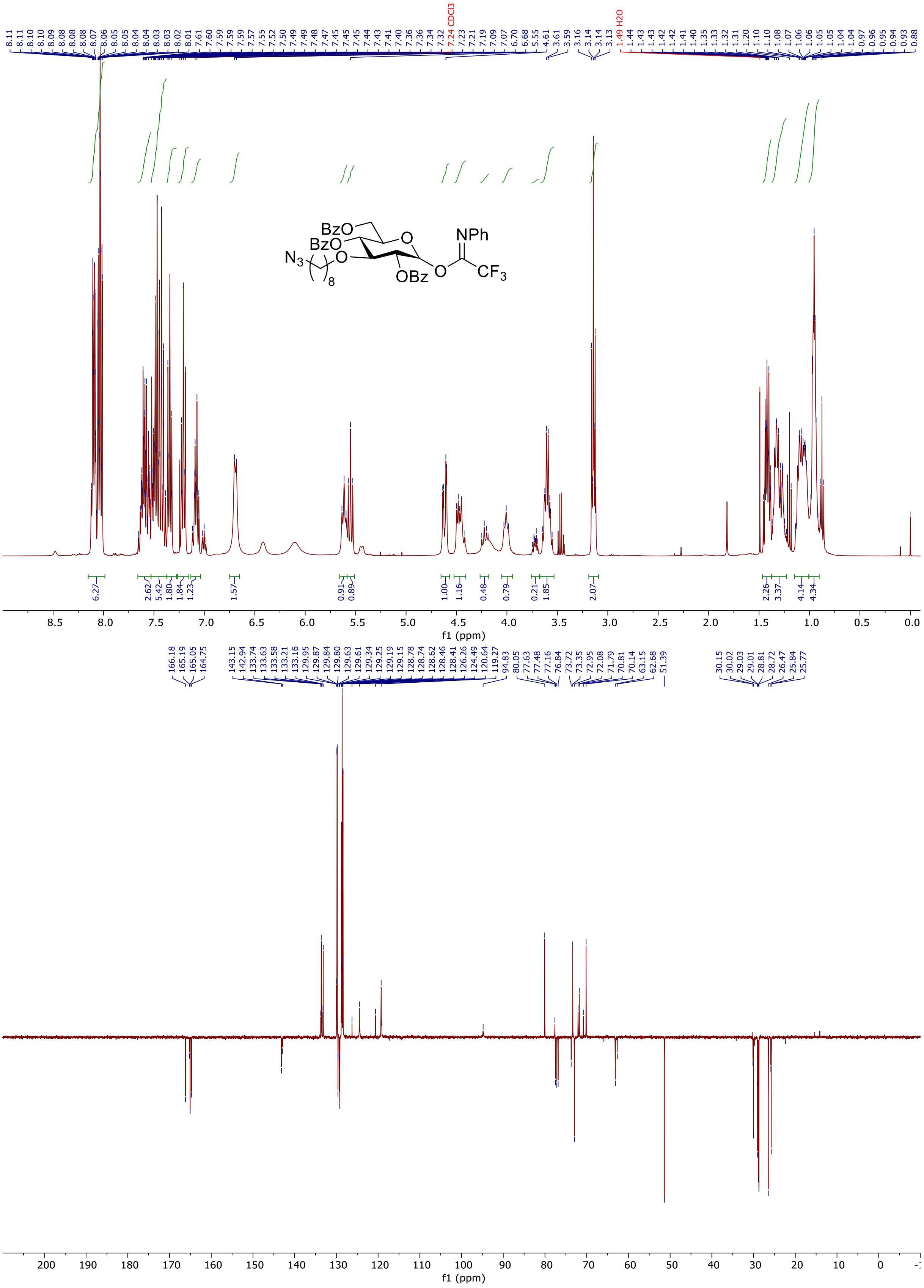

4,6-*O*-di*tert*butylsilyl-3-*O*-naphthyl-D-mannose-cyclohexene (38)

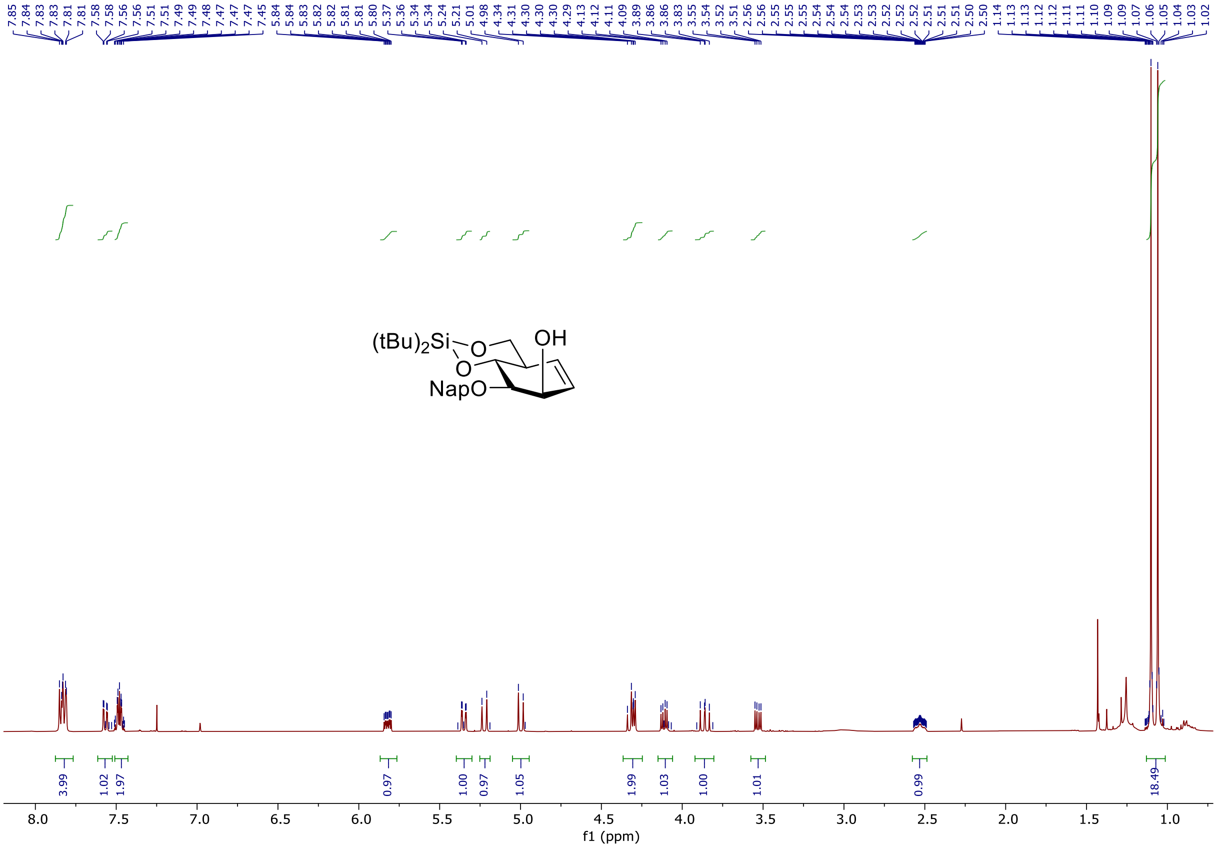

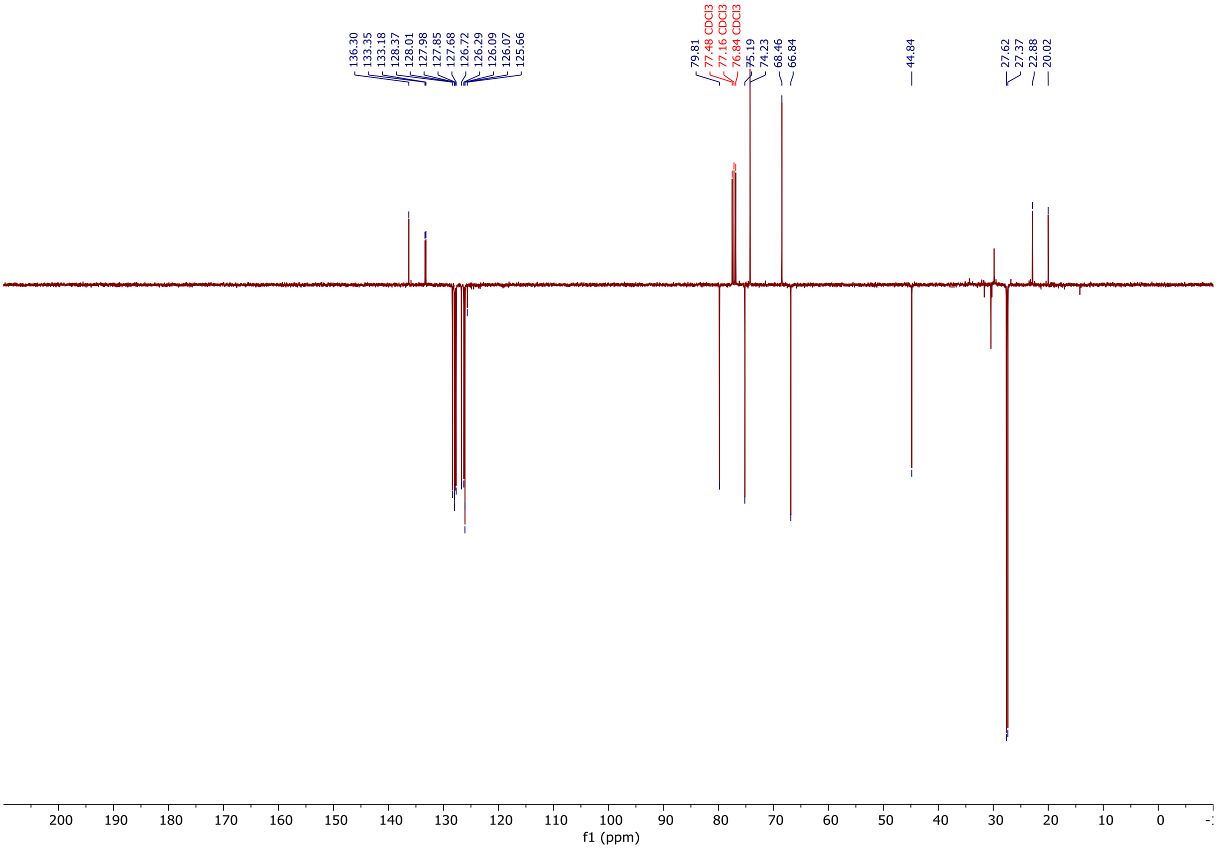

2,3,4,6-tetra-*O*-benzoyl-α-D-Mannose-(1->2)-4,6-*O*-ditertbutylsilyl-3-*O*-naphthyl-D-mannose-cyclohexene (39)

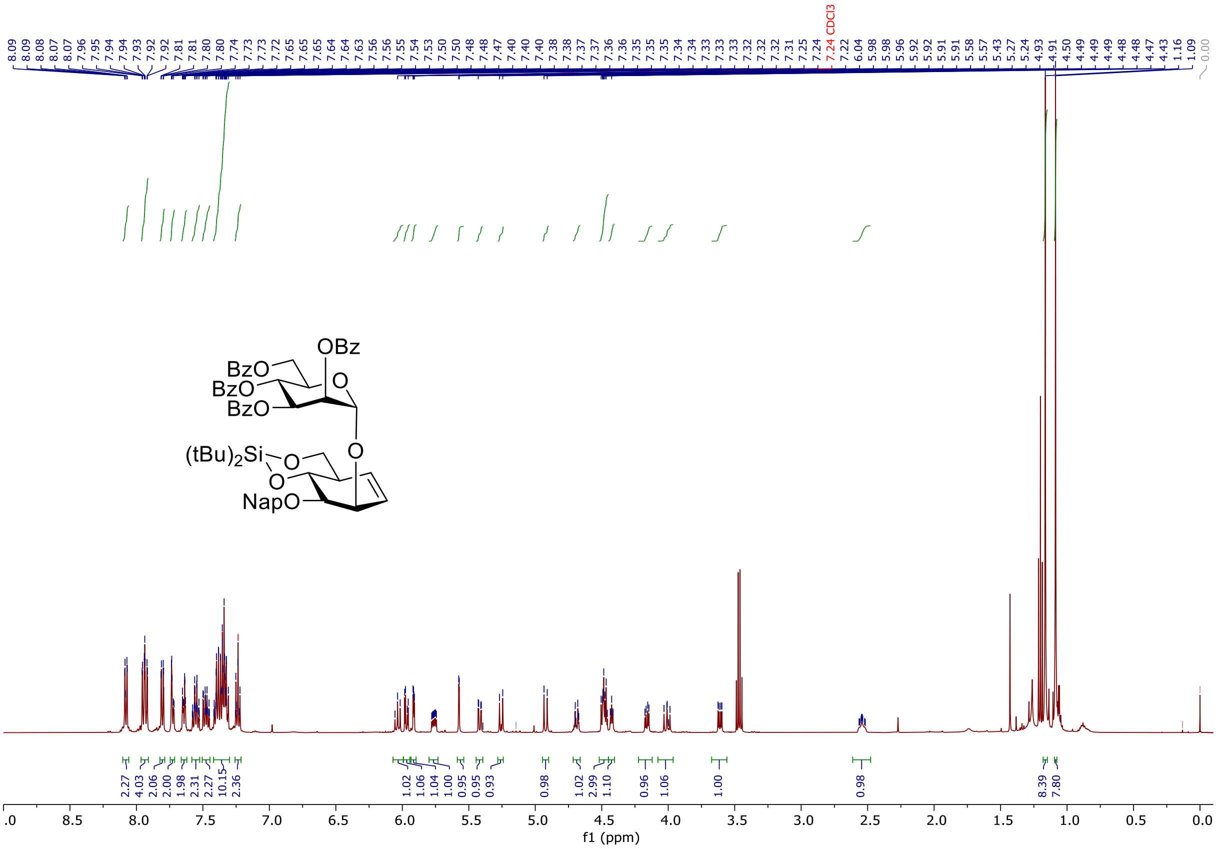

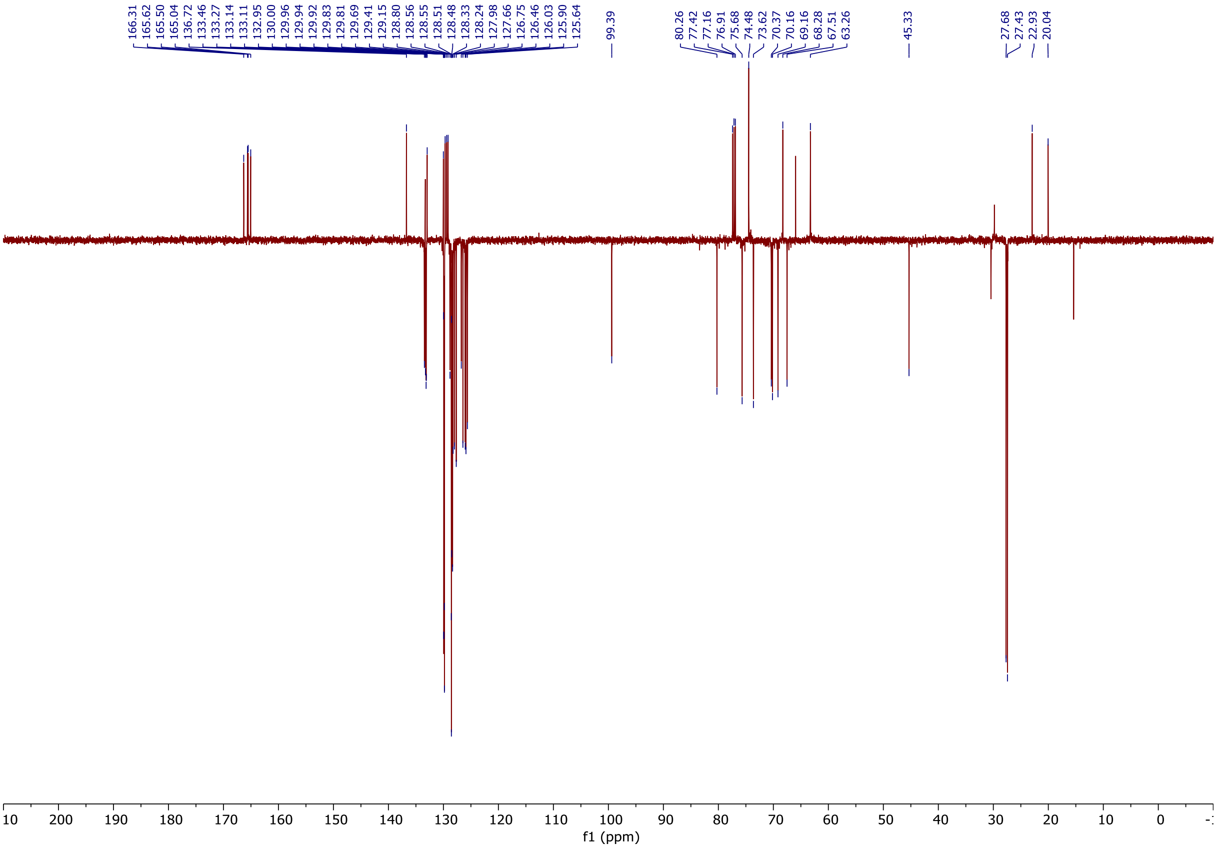

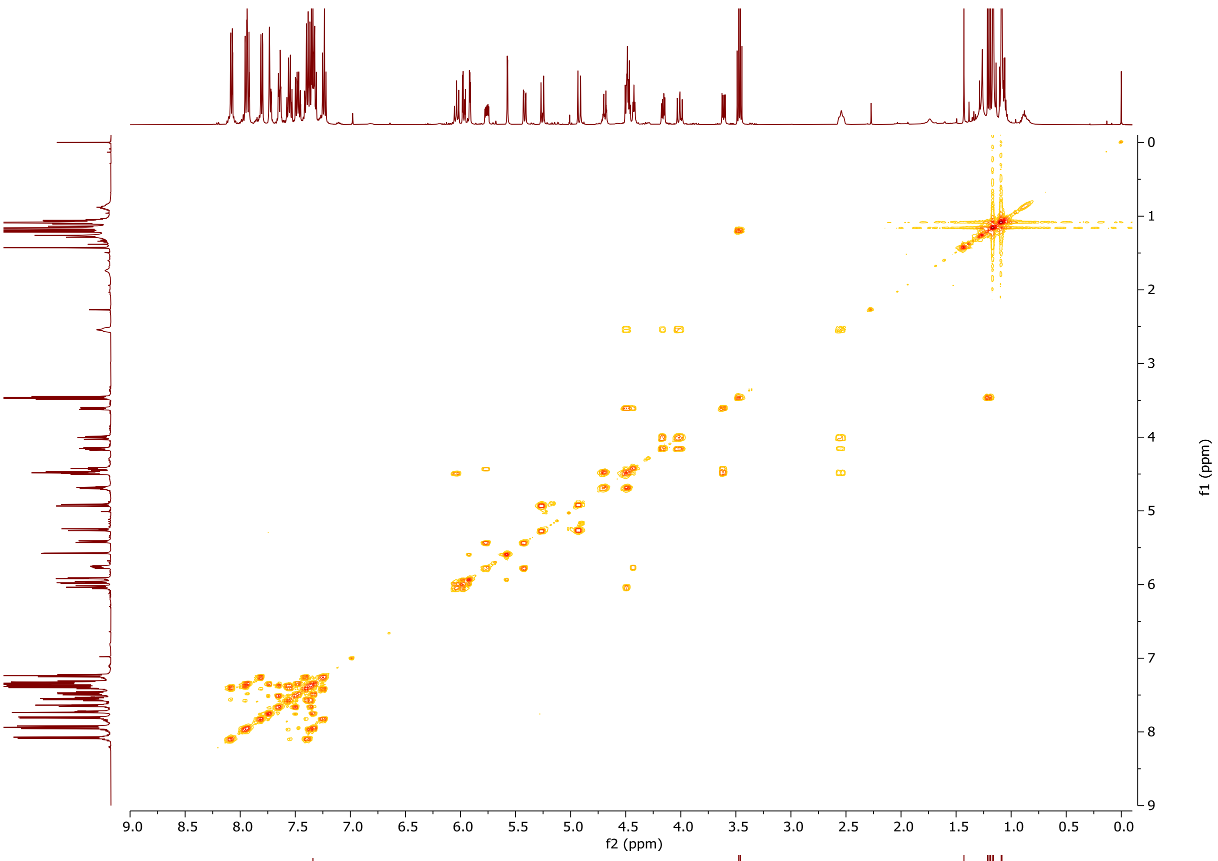

9.0
8.5
8.0
7.5
7.0
6.5
6.0
5.5
5.0
4.5
4.0
3.5
3.0
2.5
2.0
1.5
1.0
0.5
0.0
f2 (ppm)
f1 (ppm)

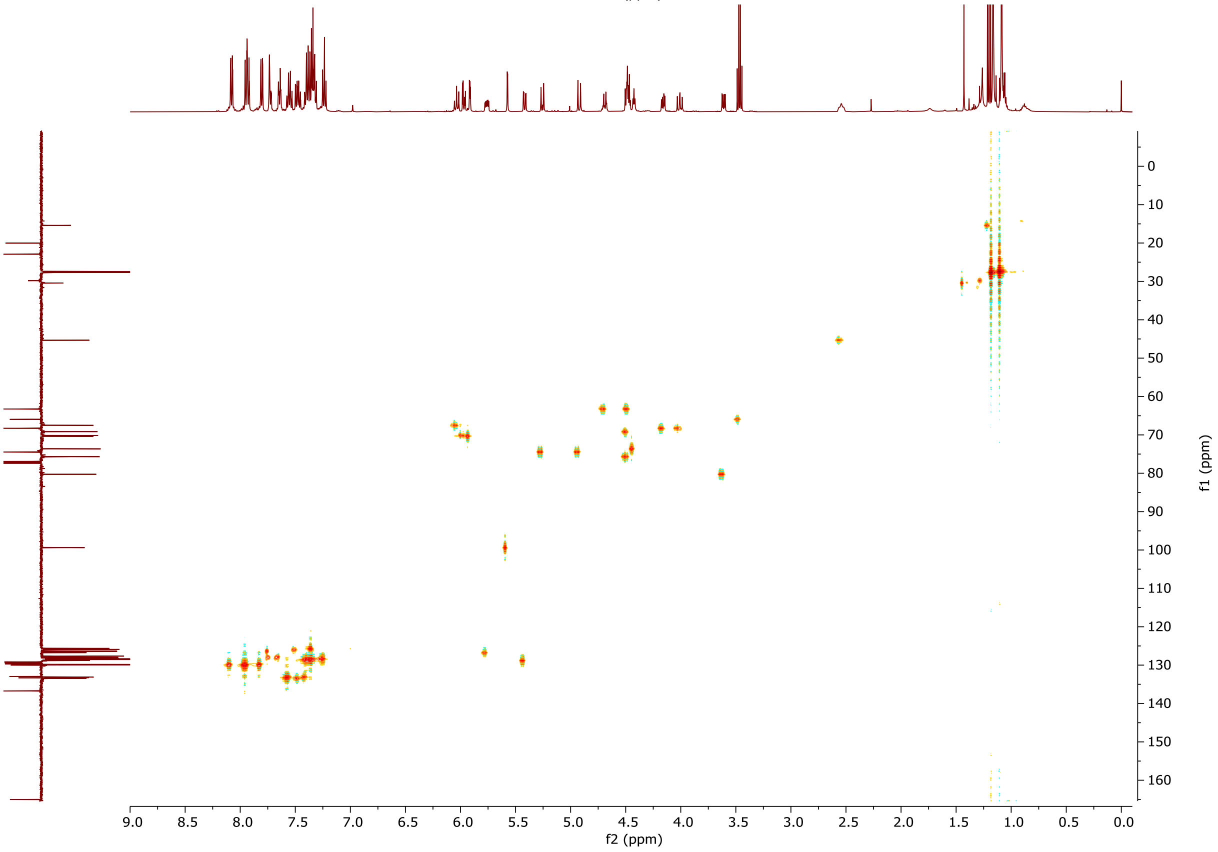

9.0
8.5
8.0
7.5
7.0
6.5
6.0
5.5
5.0
4.5
4.0
3.5
3.0
2.5
2.0
1.5
1.0
0.5
0.0
f2 (ppm)
f1 (ppm)

2,3,4,6-tetra-*O*-benzoyl-α-D-mannose-(1->2)-4,6-*O*-di*tert*butylsilyl-D-mannose-cyclohexene (40)

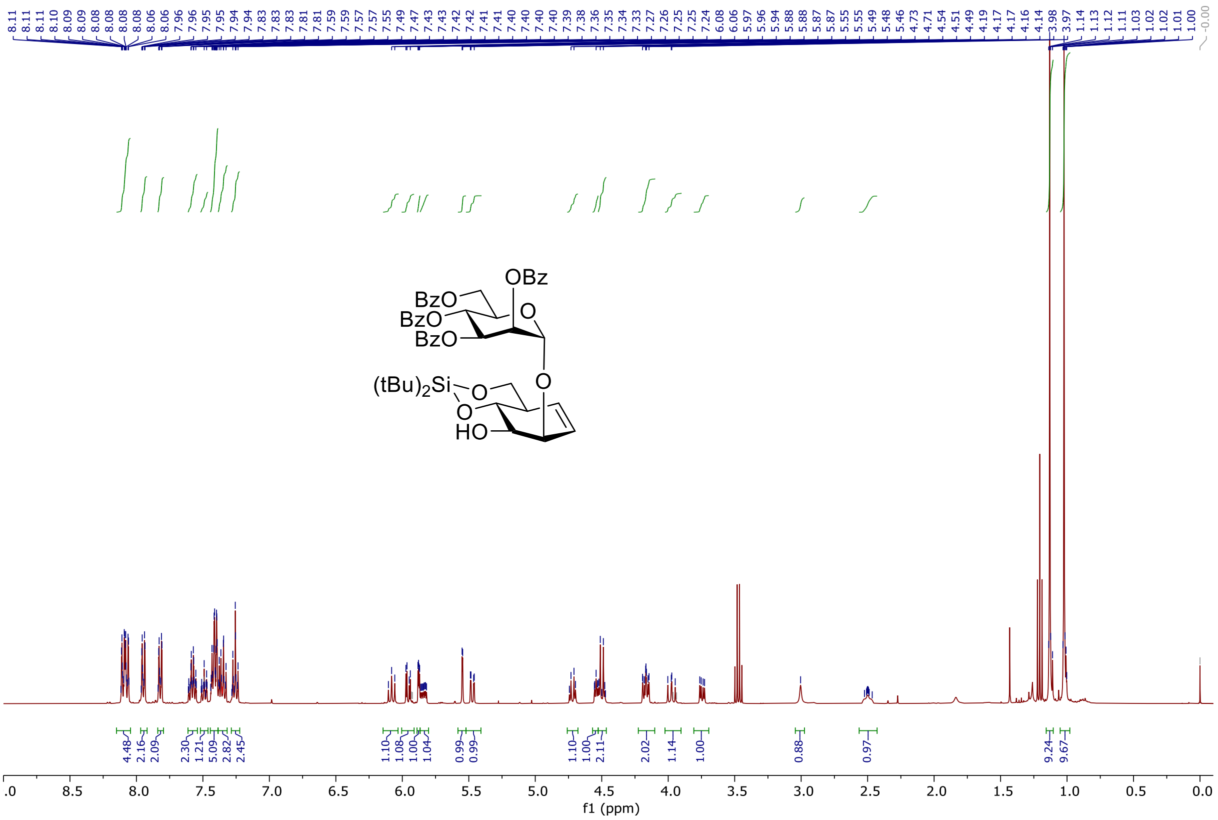

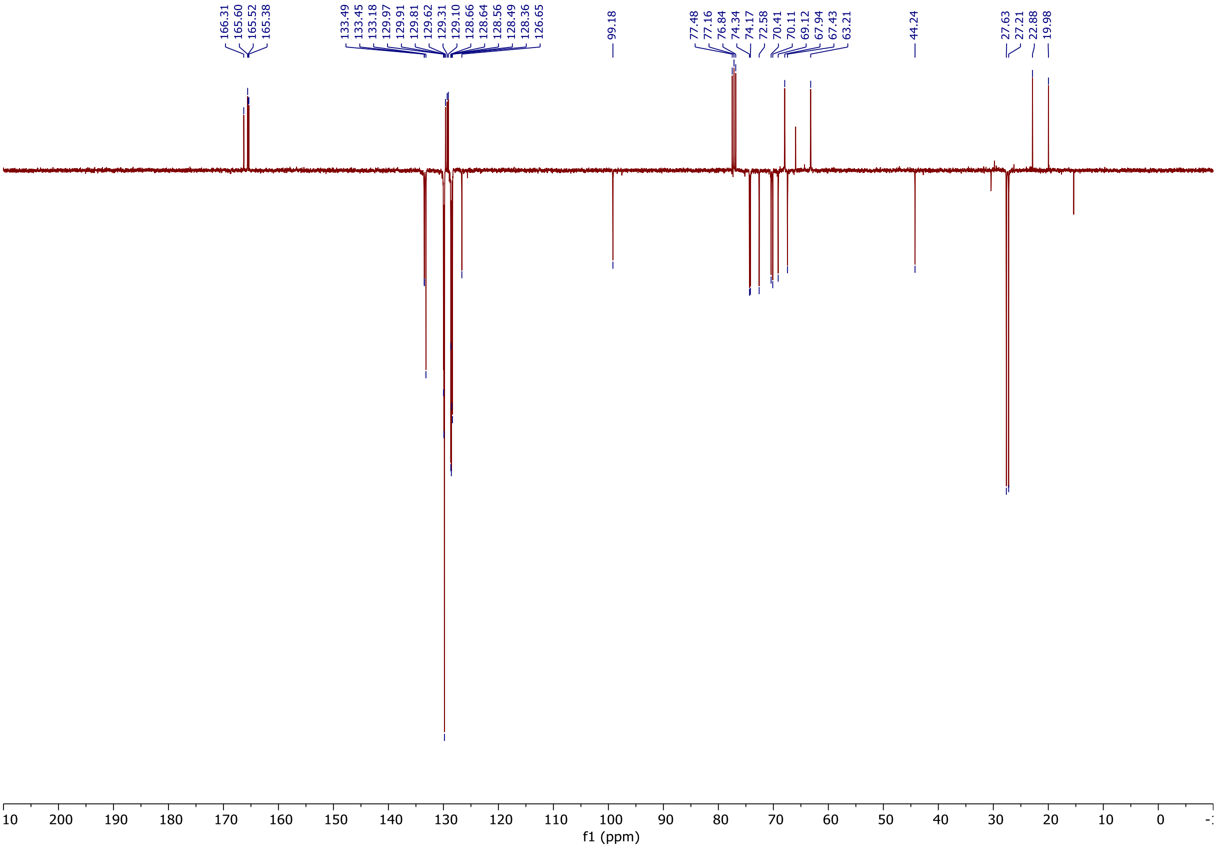

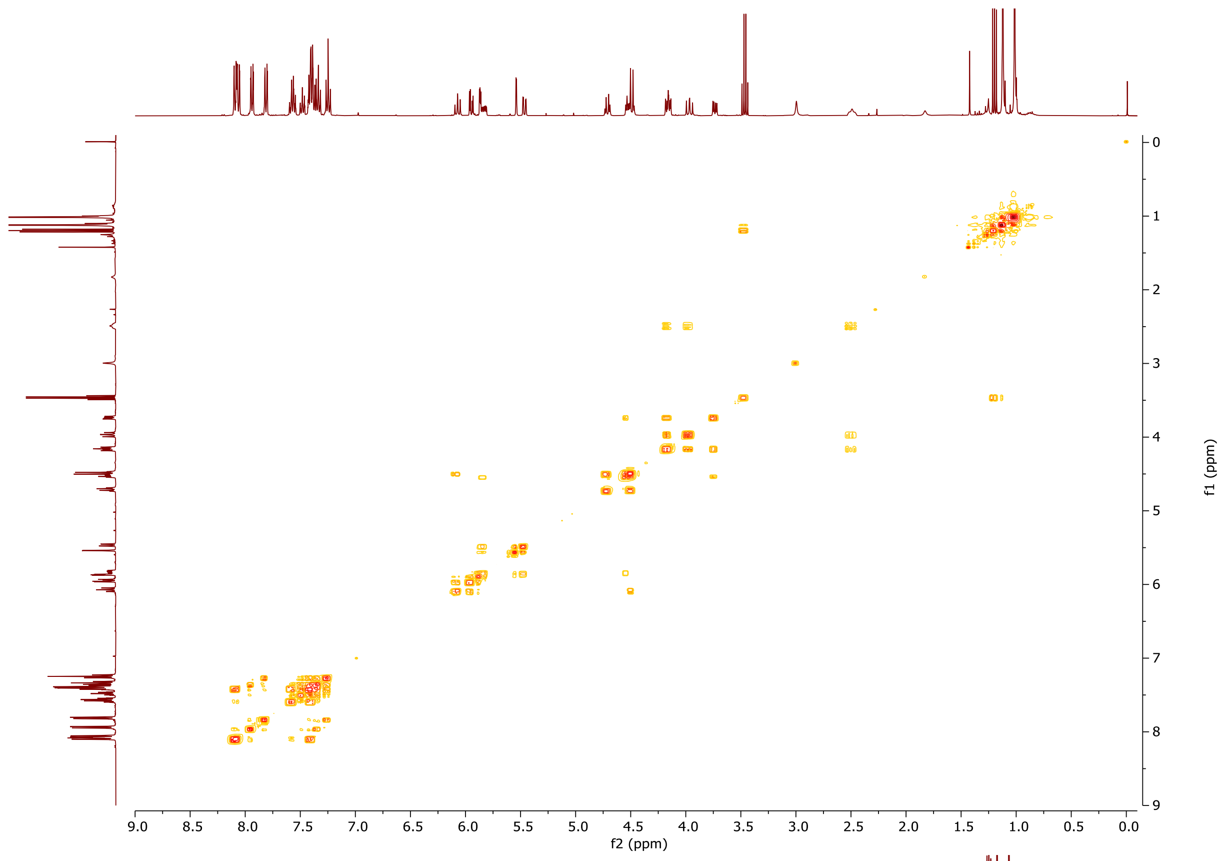

9.0 8.5 8.0 7.5 7.0 6.5 6.0 5.5 5.0 4.5 4.0 3.5 3.0 2.5 2.0 1.5 1.0 0.5 0.0
f2 (ppm)
0 1 2 3 4 5 6 7 8 9
f1 (ppm)

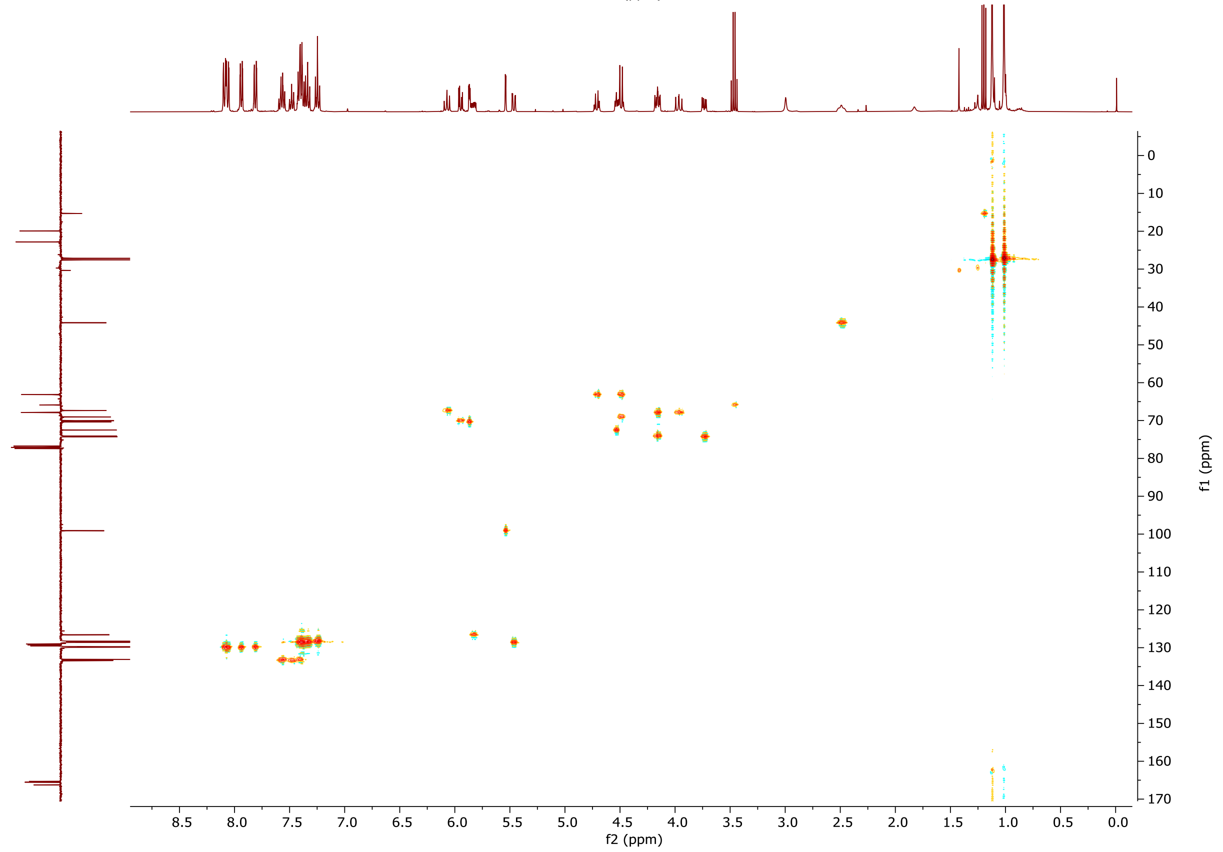

8.5 8.0 7.5 7.0 6.5 6.0 5.5 5.0 4.5 4.0 3.5 3.0 2.5 2.0 1.5 1.0 0.5 0.0
f2 (ppm)
0 10 20 30 40 50 60 70 80 90 100 110 120 130 140 150 160 170
f1 (ppm)

[2,3,4,6-tetra-*O*-benzoyl-α-D-mannose-(1->2)]-[2,4,6-*O*-benzoyl-3-*O*-(8-azido-octane)-β-D-glucose-(1->3)]-4,6-*O*-di*tert*butylsilyl-D-mannose-cyclohexene (41)

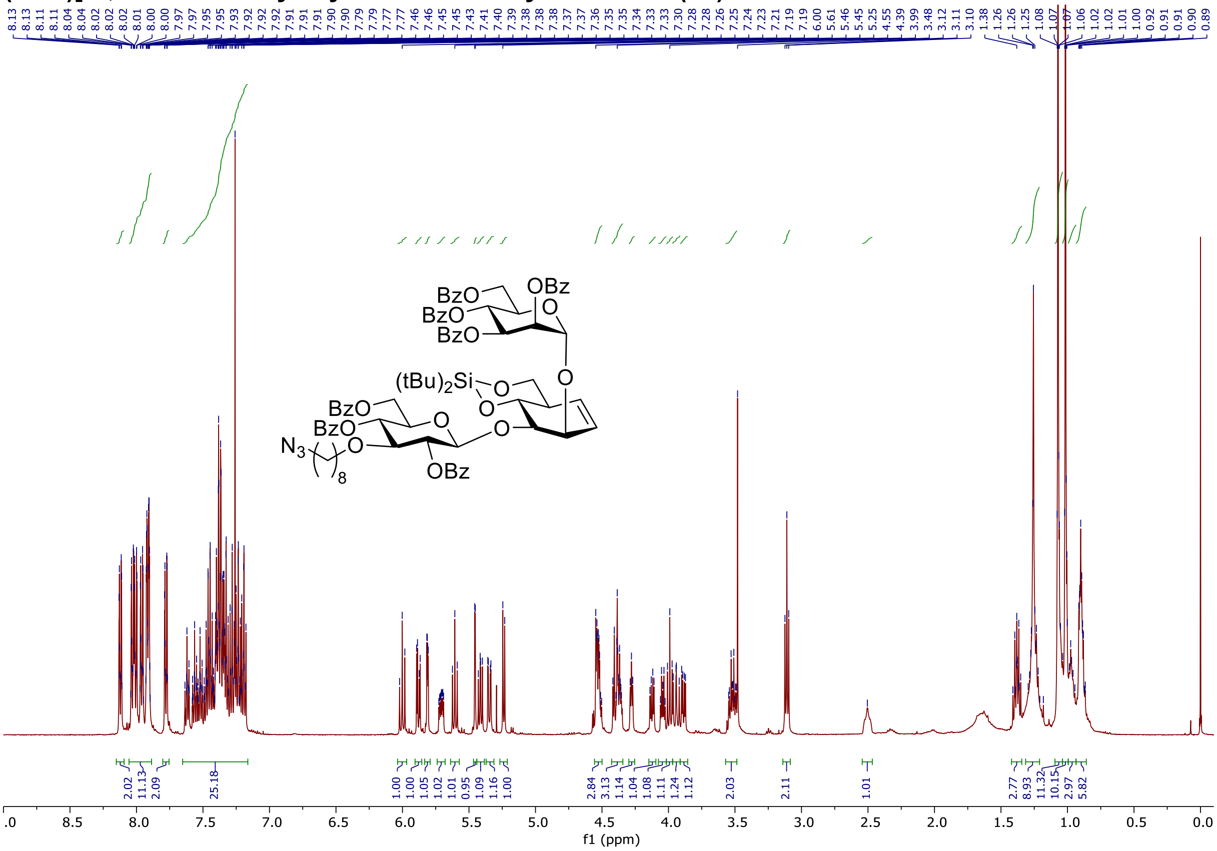

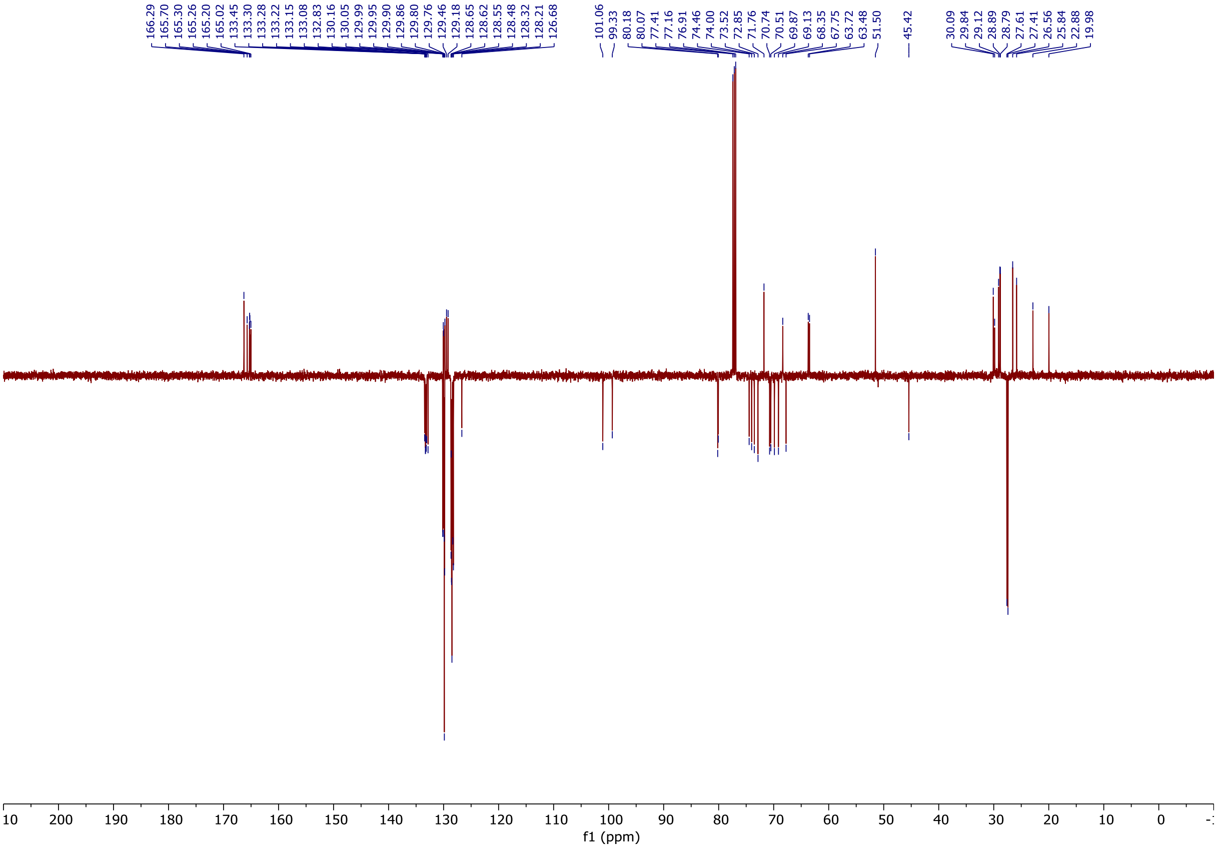

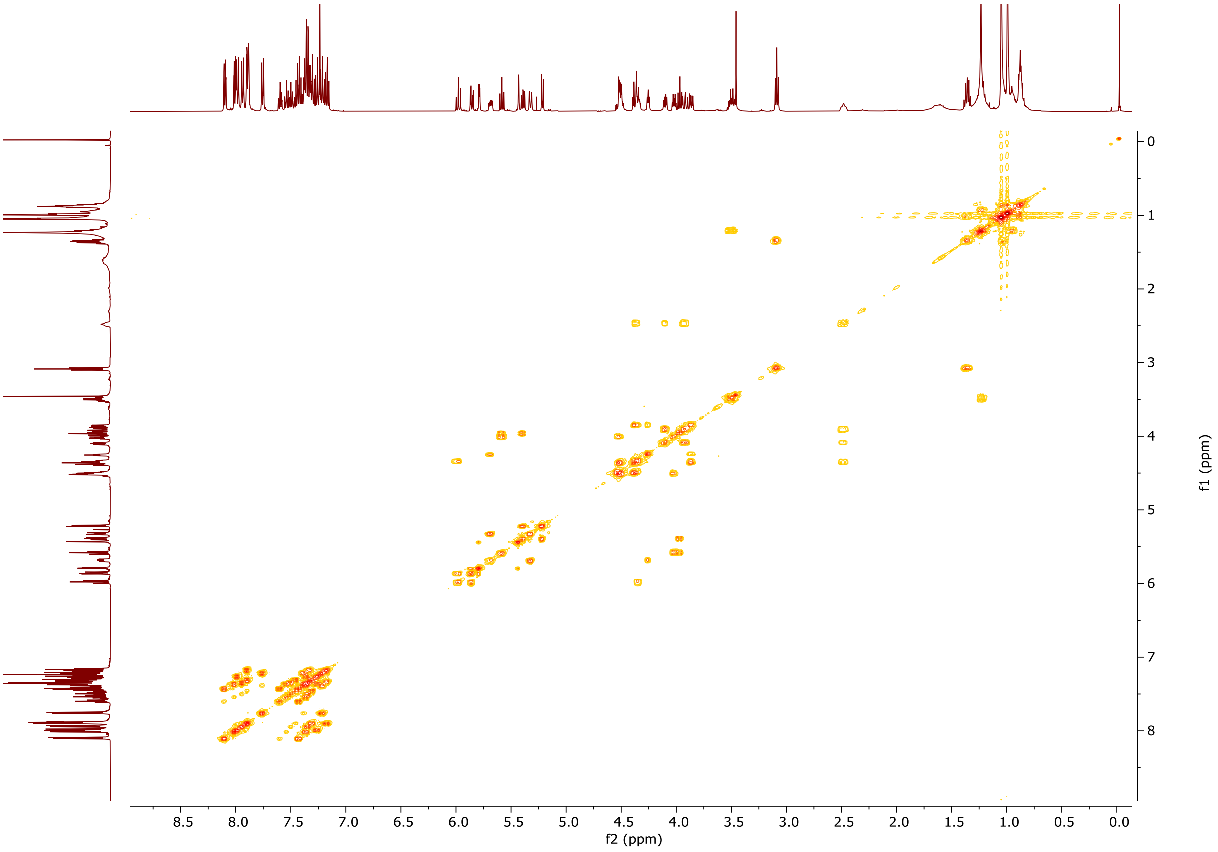
f2 (ppm)
f1 (ppm)

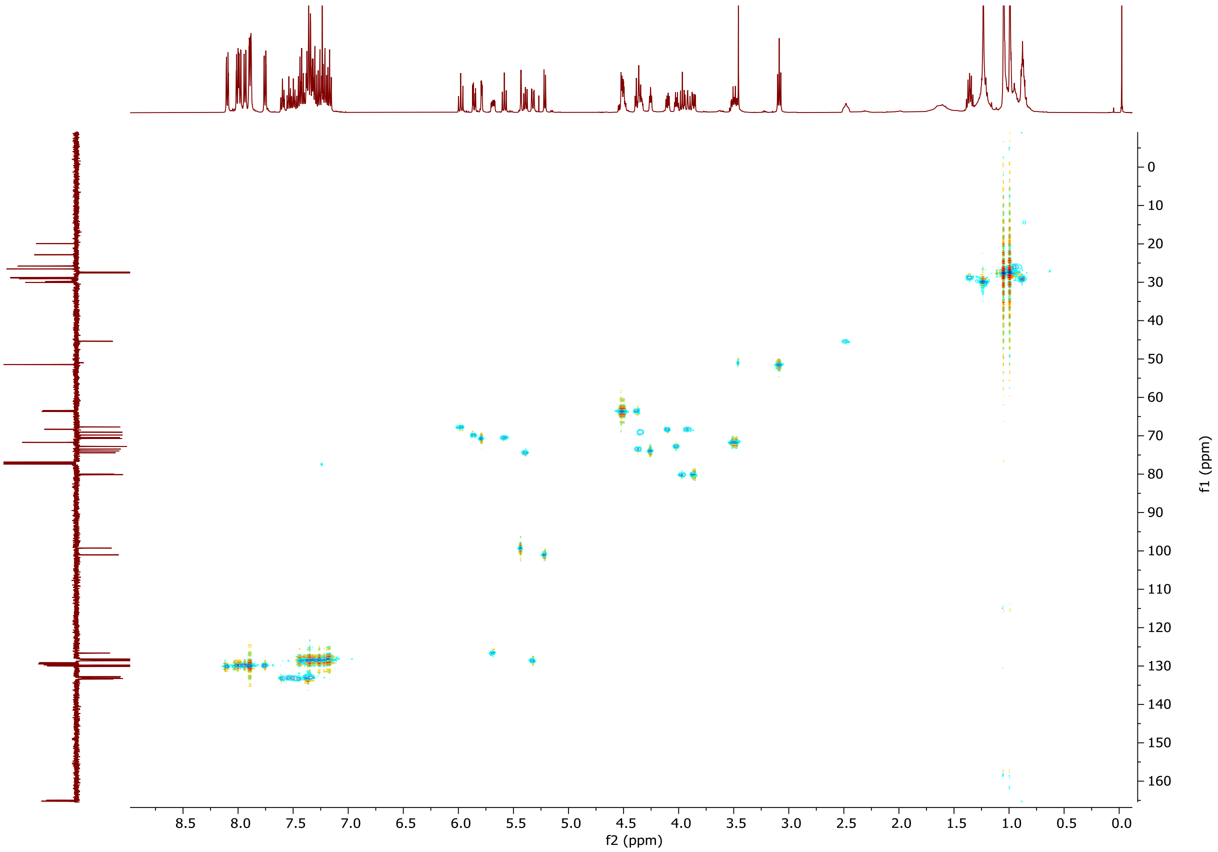
f2 (ppm)
f1 (ppm)

[2,3,4,6-tetra-*O*-benzoyl-α-D-mannose-(1->2)]-[2,4,6-*O*-benzoyl-3-*O*-(8-azido-octyl)-β-D-glucose-(1->3)]-D-mannose-cyclohexene (42)

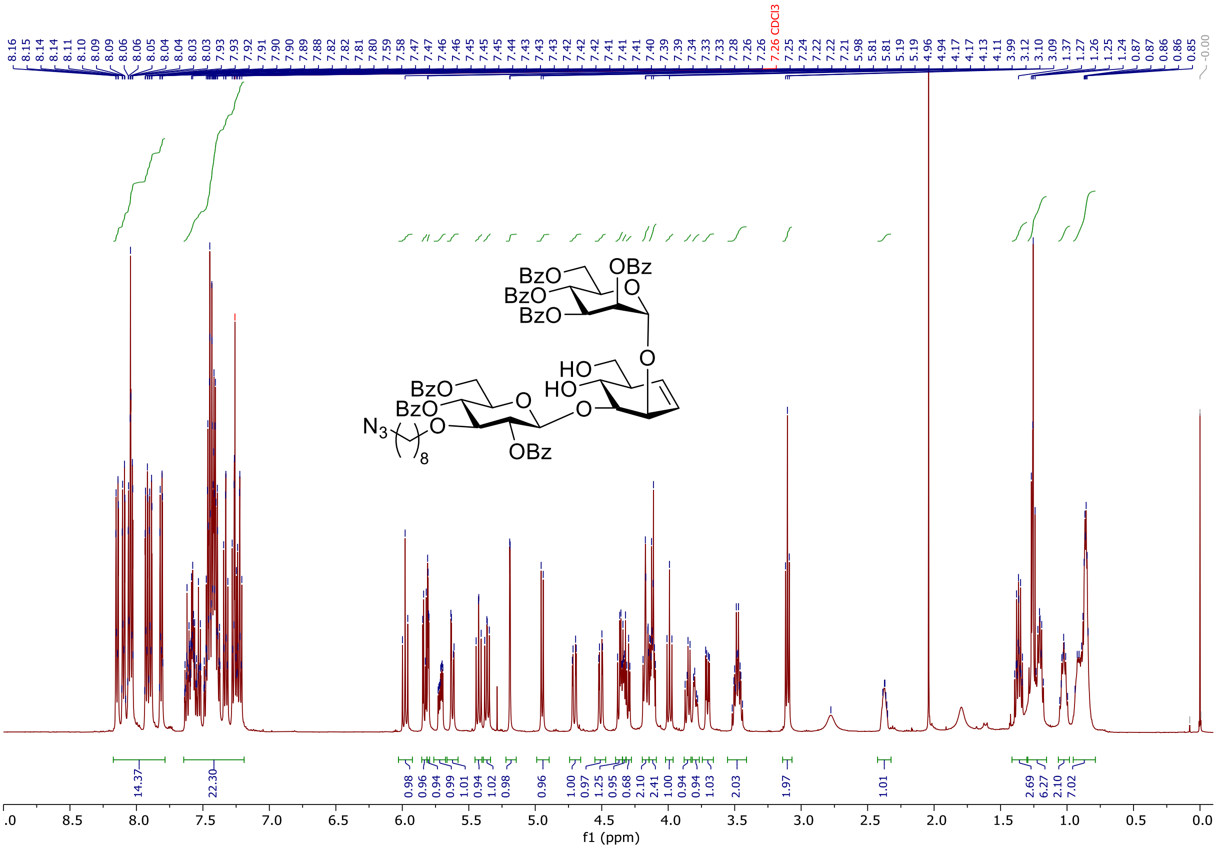

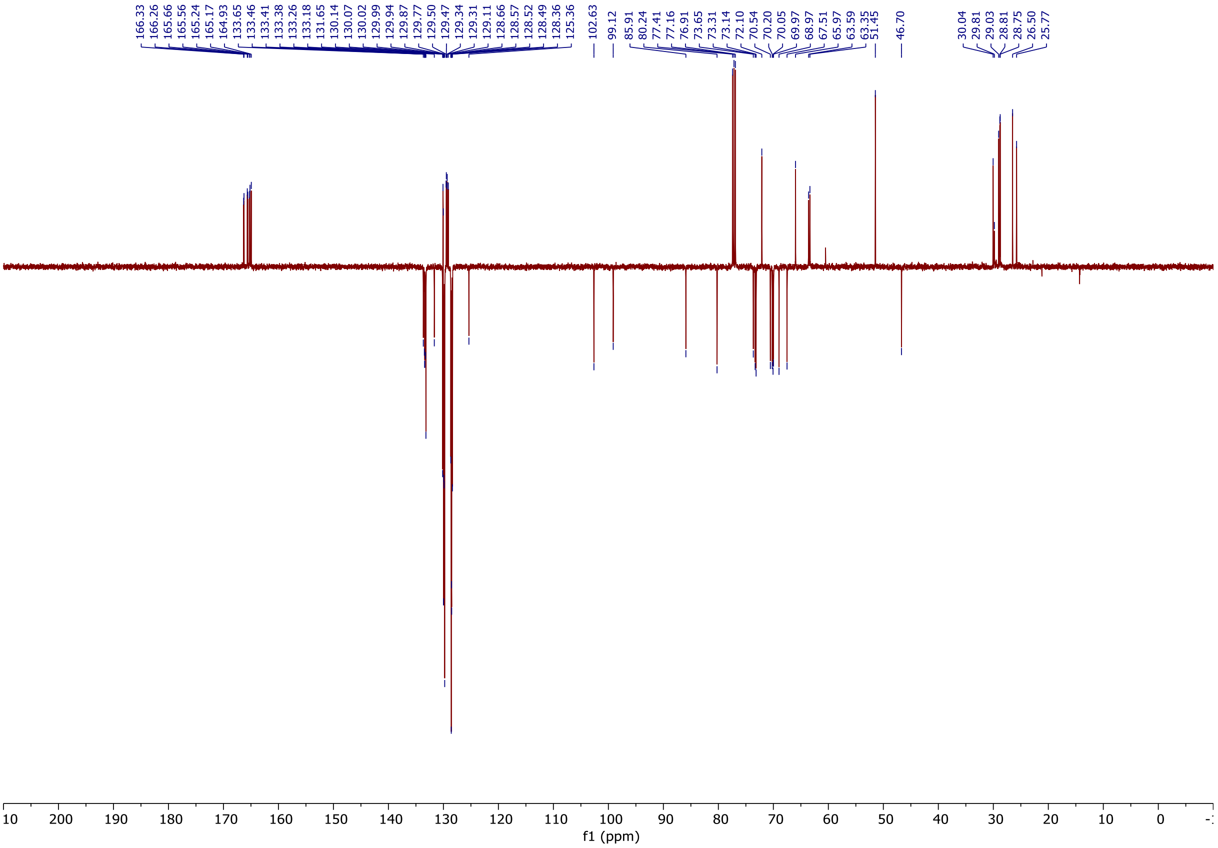

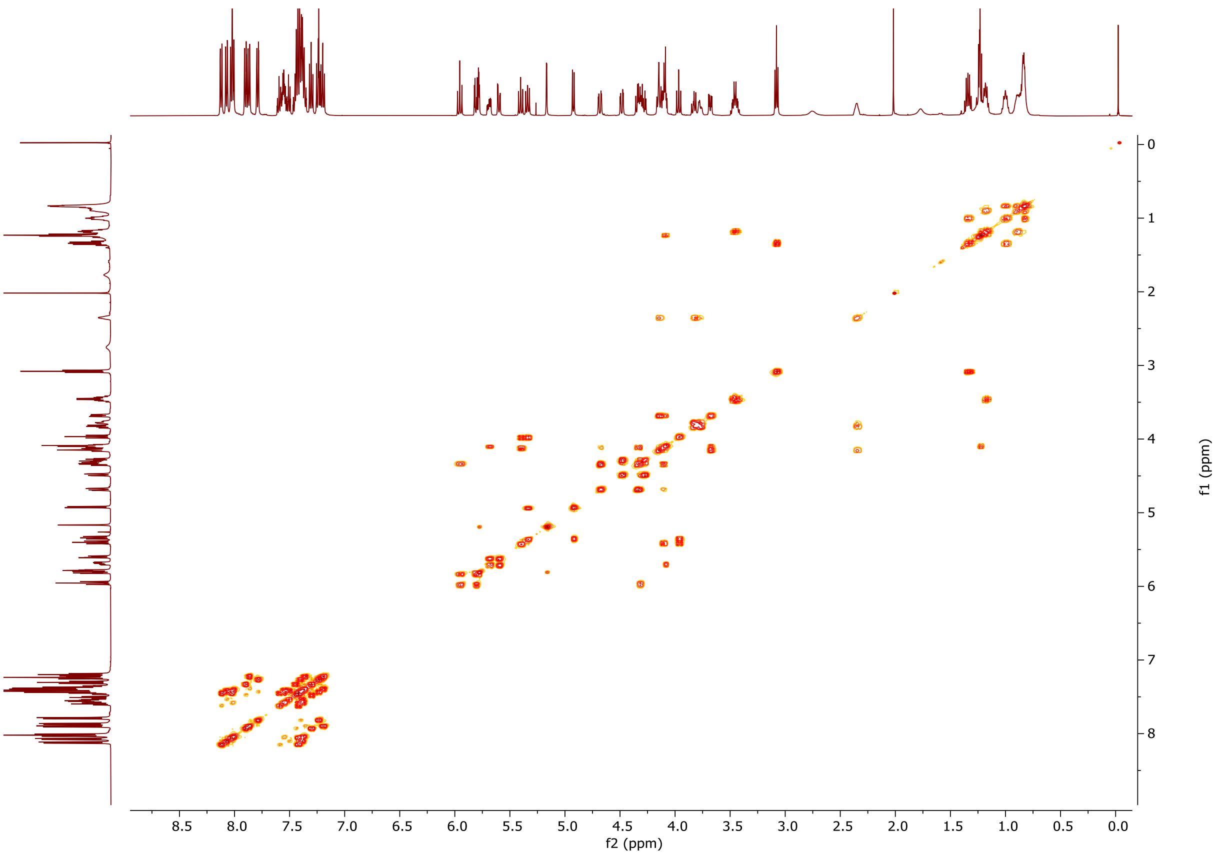
8.5 8.0 7.5 7.0 6.5 6.0 5.5 5.0 4.5 4.0 3.5 3.0 2.5 2.0 1.5 1.0 0.5 0.0
f2 (ppm)
0 1 2 3 4 5 6 7 8
f1 (ppm)

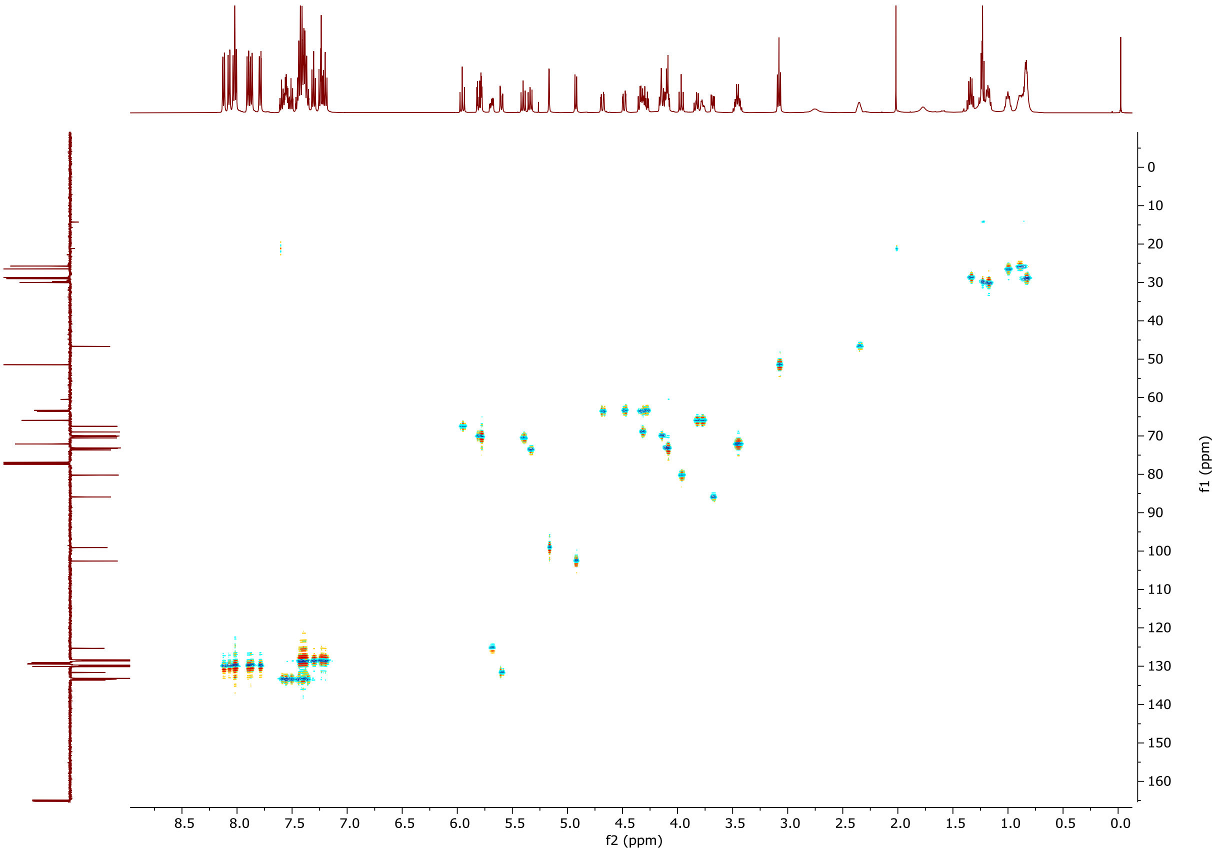
8.5 8.0 7.5 7.0 6.5 6.0 5.5 5.0 4.5 4.0 3.5 3.0 2.5 2.0 1.5 1.0 0.5 0.0
f2 (ppm)
0 10 20 30 40 50 60 70 80 90 100 110 120 130 140 150 160
f1 (ppm)

6-O-acetyl-[2,3,4,6-tetra-*O*-benzoyl-α-D-mannose-(1->2)]-[2,4,6-*O*-benzoyl-3-*O*-(8-azido-octyl)-β-D-glucose-(1->3)]-β-D-mannose-cyclophellitol (44)

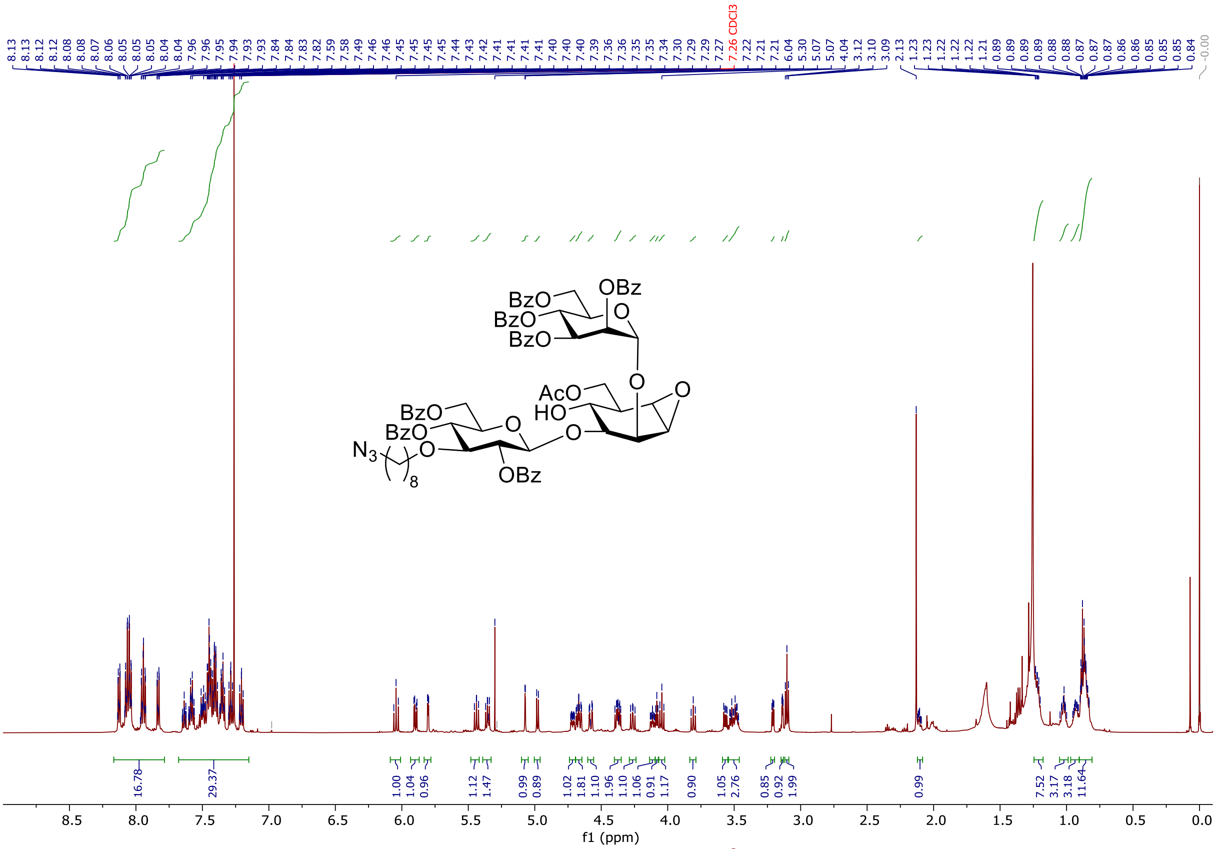

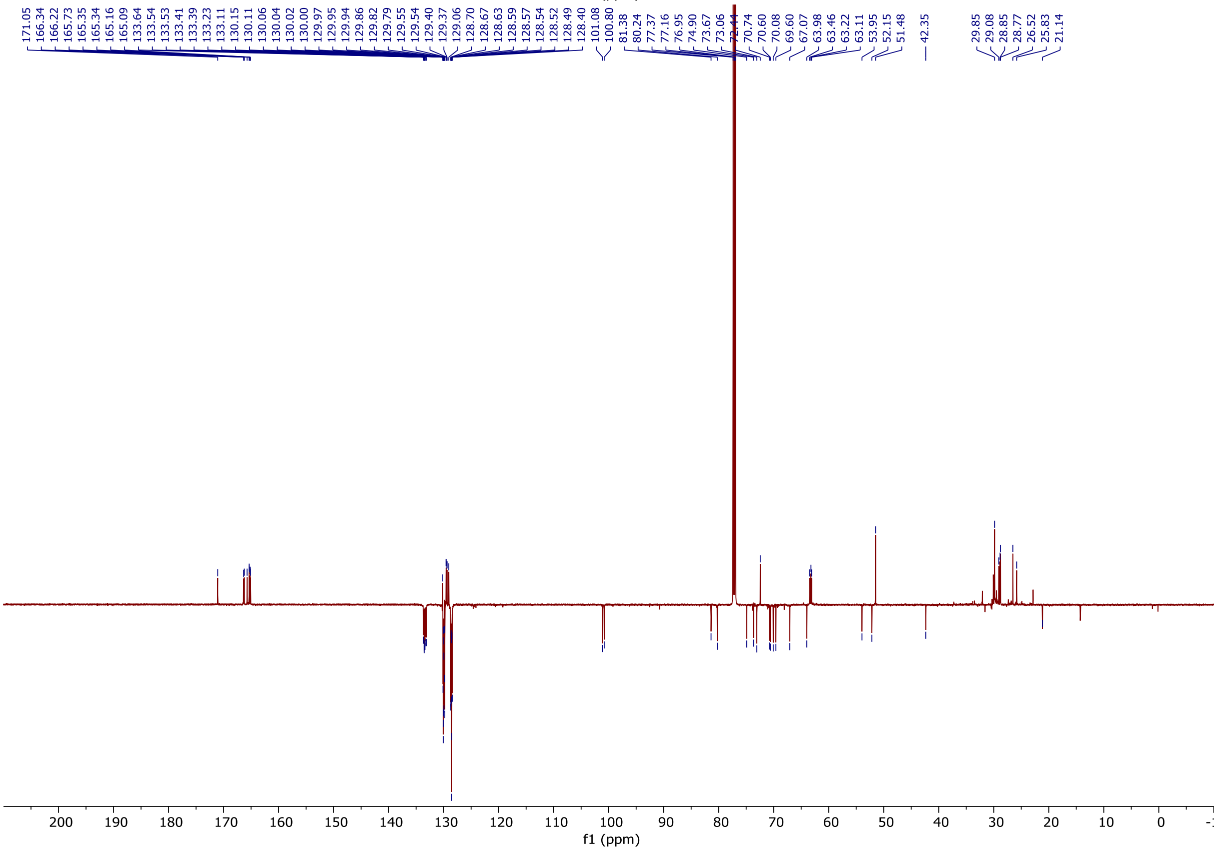

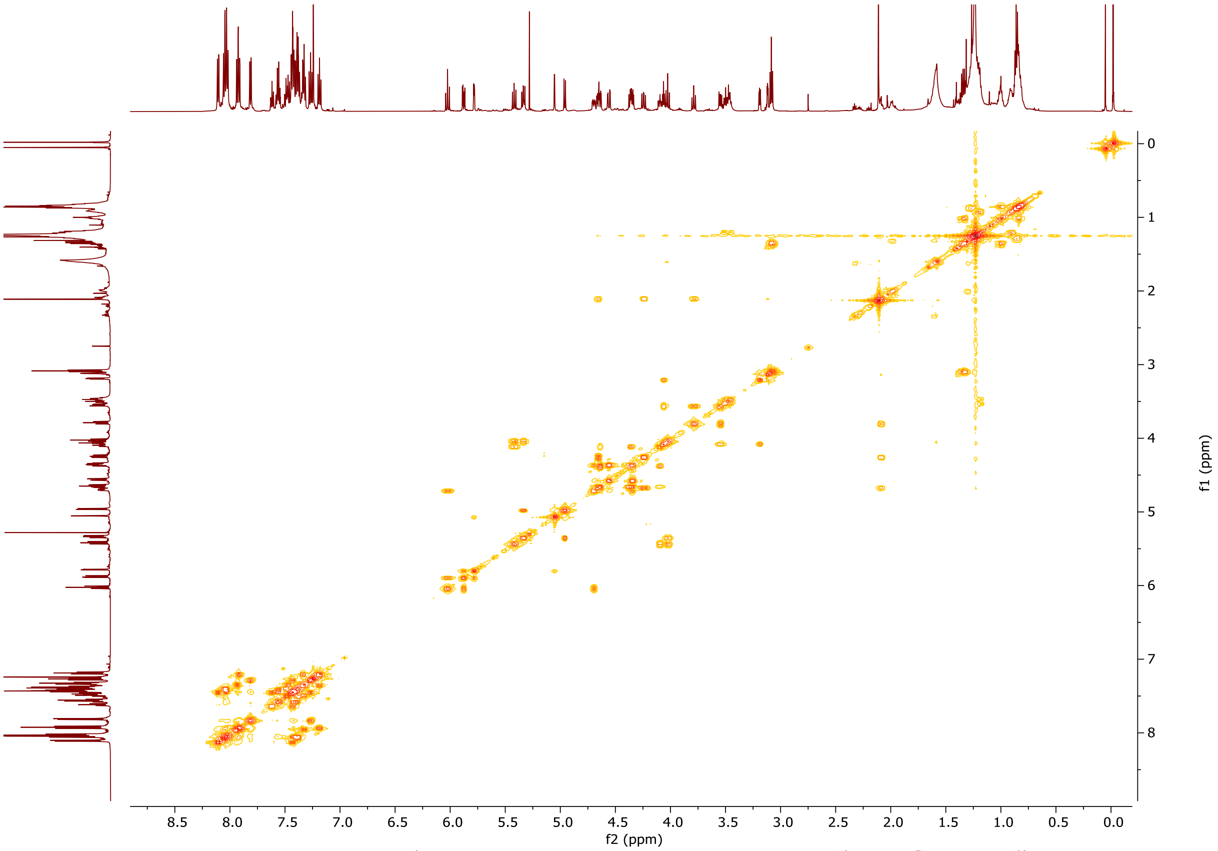
f2 (ppm)
f1 (ppm)

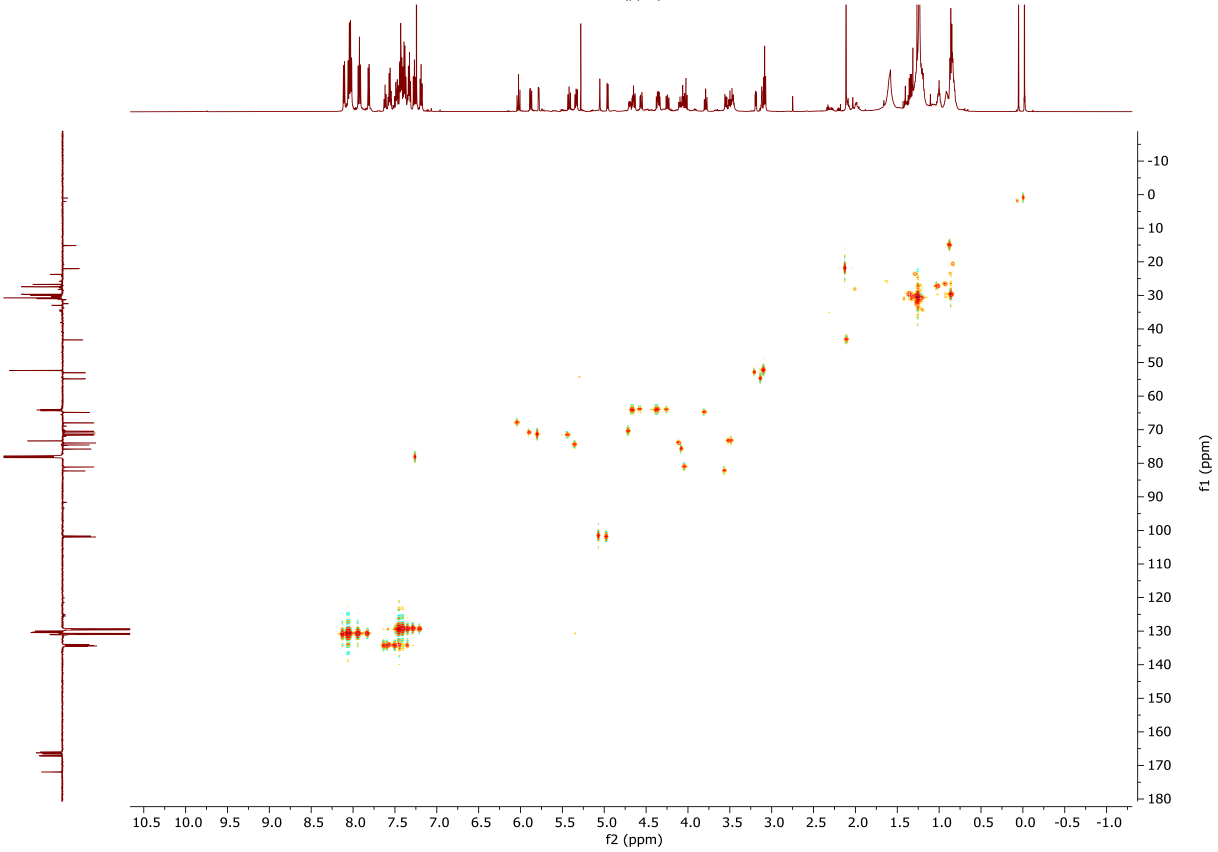
f2 (ppm)
f1 (ppm)

[α-D-mannose-(1->2)]-[3-O-(8-azido-octyl)-β-D-glucose-(1->3)]-β-D-mannose-cyclophellitol (45)

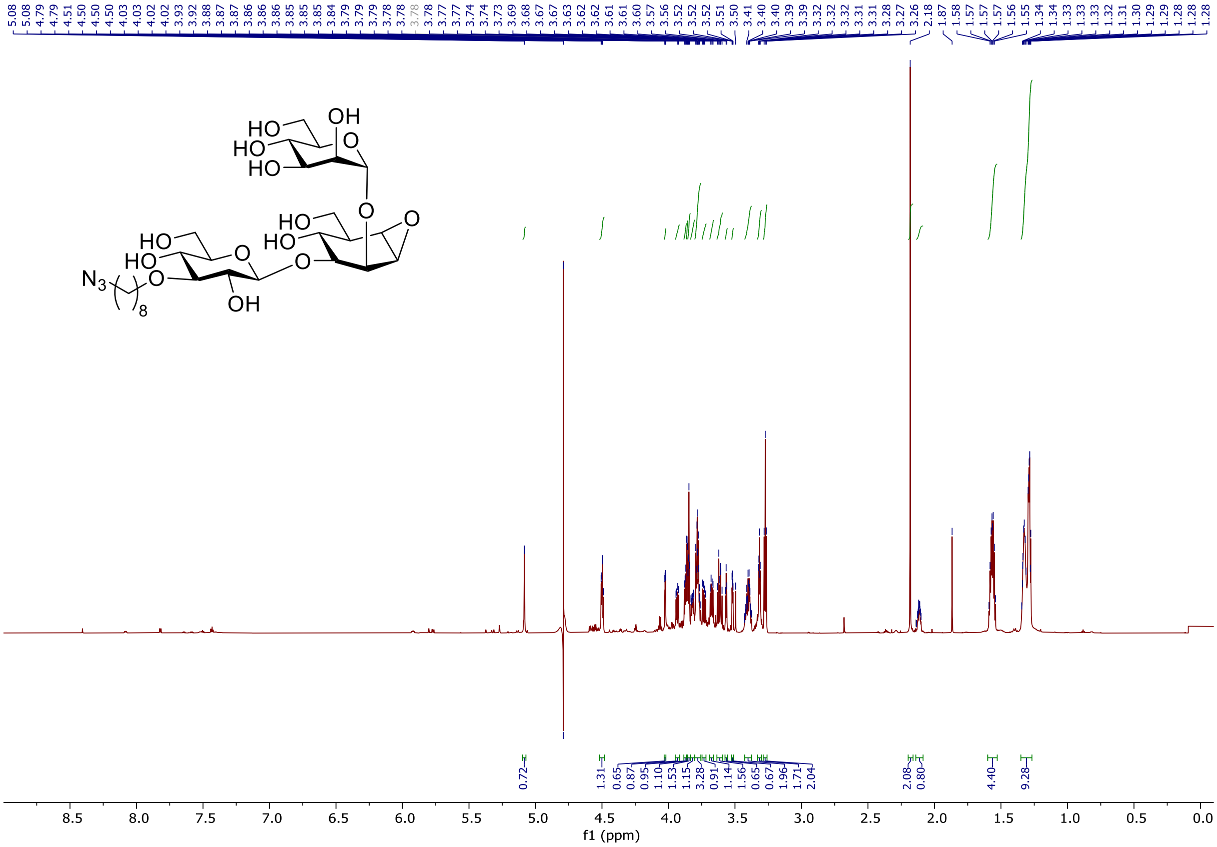

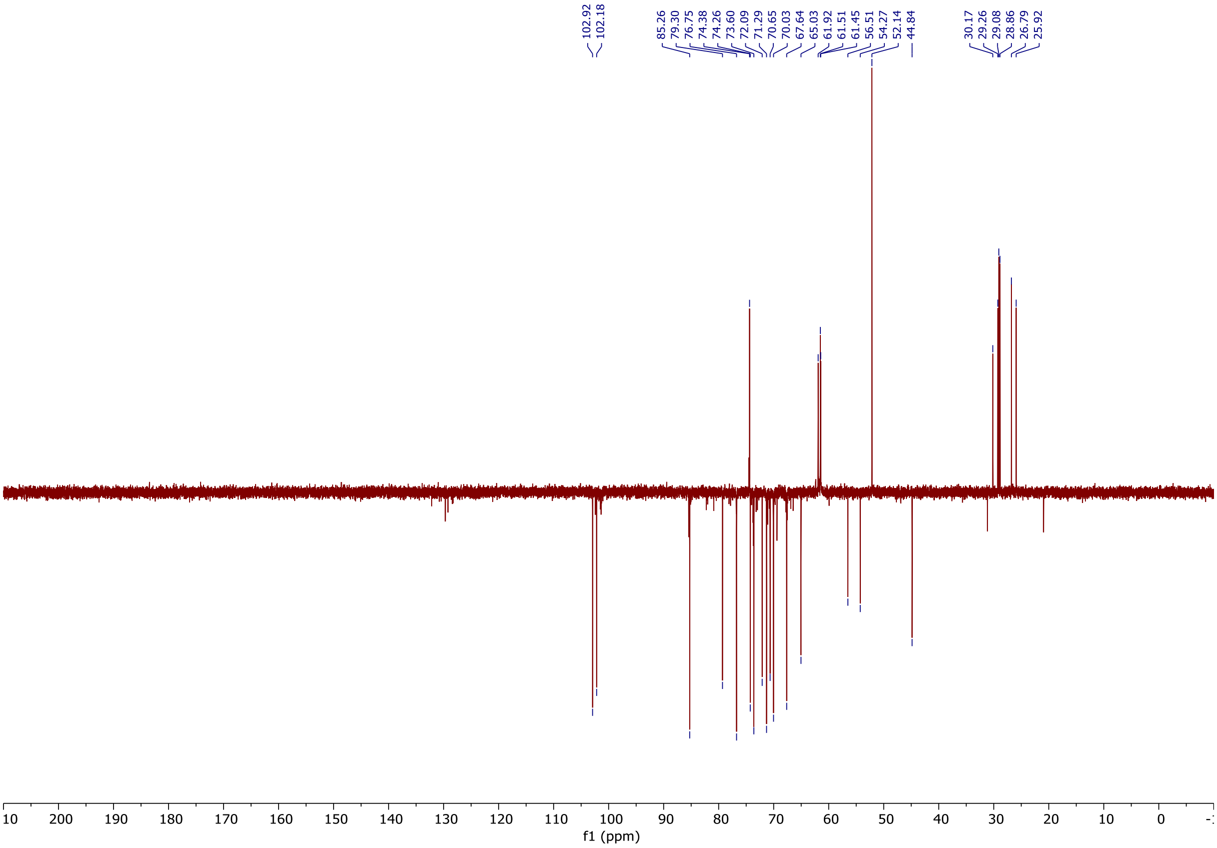

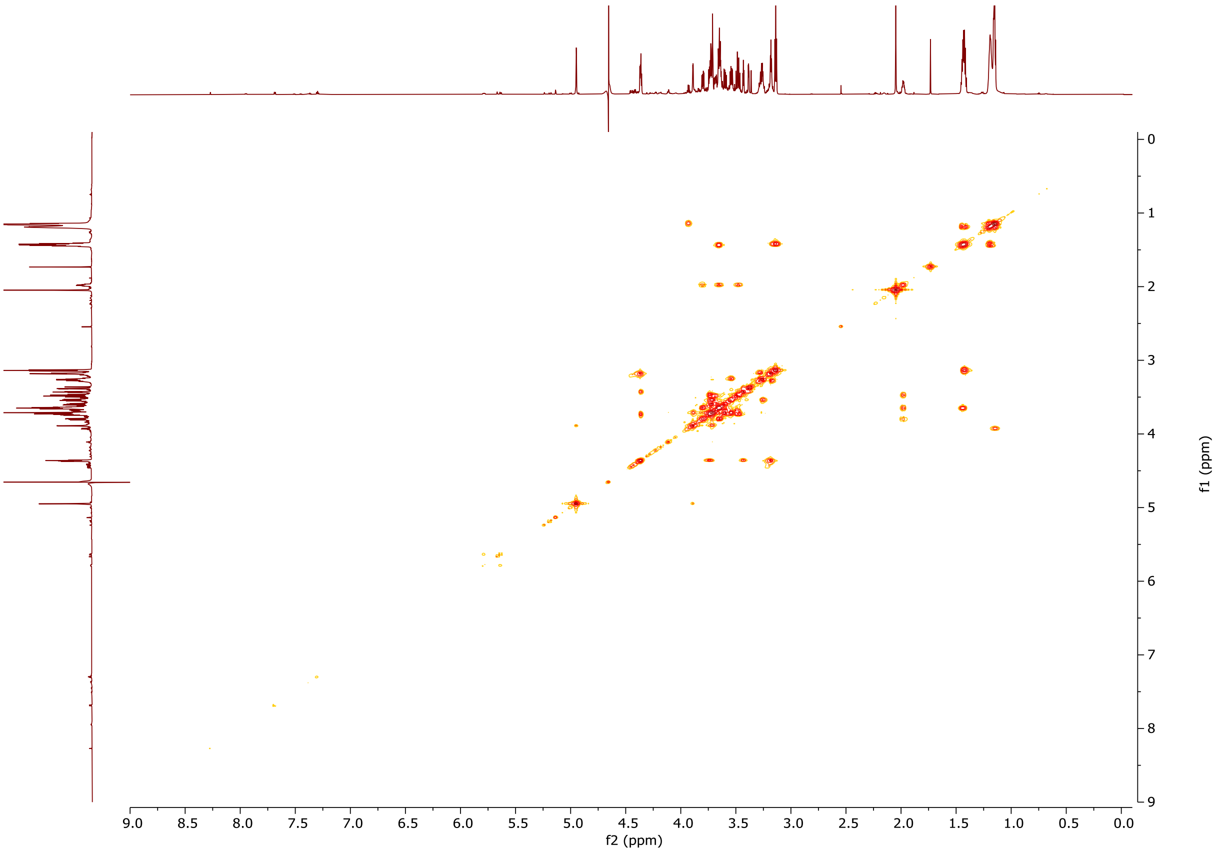
9.0 8.5 8.0 7.5 7.0 6.5 6.0 5.5 5.0 4.5 4.0 3.5 3.0 2.5 2.0 1.5 1.0 0.5 0.0
f2 (ppm)
0 1 2 3 4 5 6 7 8 9
f1 (ppm)

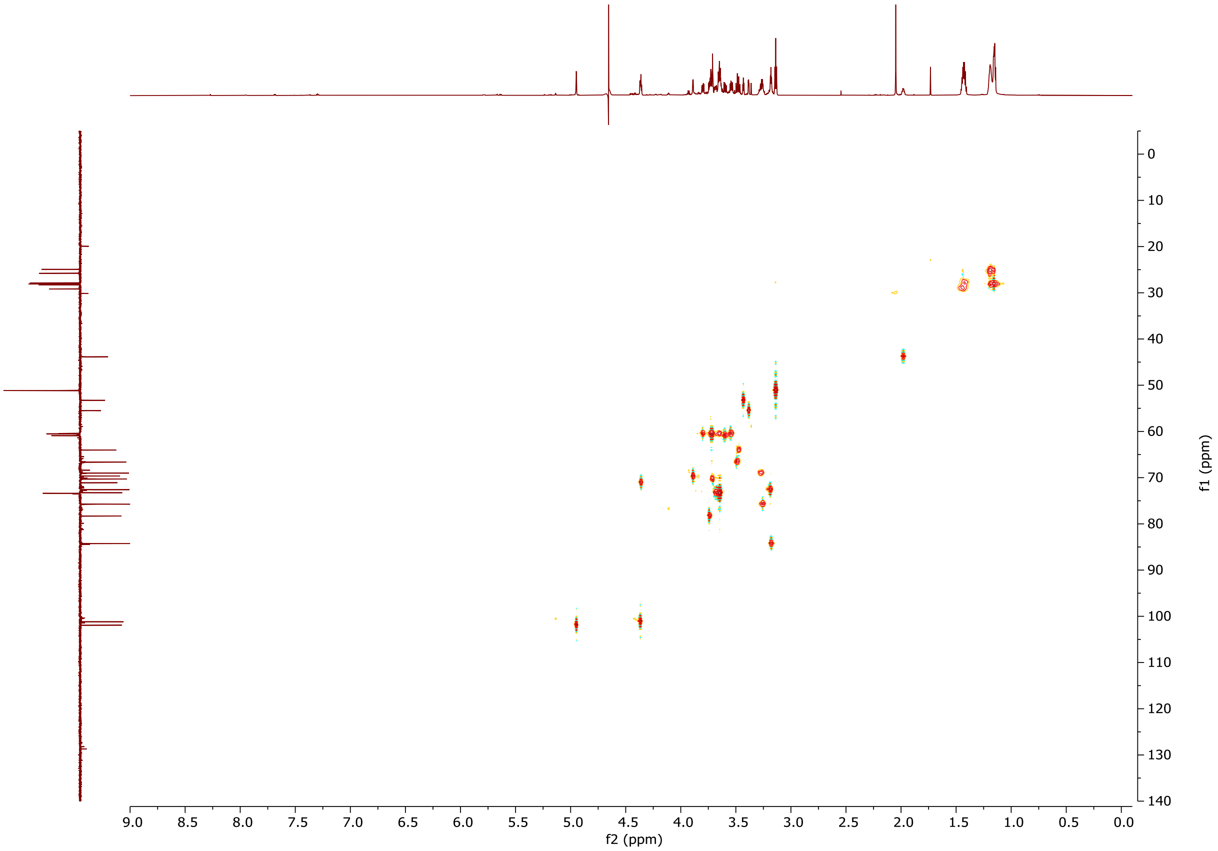
9.0 8.5 8.0 7.5 7.0 6.5 6.0 5.5 5.0 4.5 4.0 3.5 3.0 2.5 2.0 1.5 1.0 0.5 0.0
f2 (ppm)
0 10 20 30 40 50 60 70 80 90 100 110 120 130 140
f1 (ppm)

[α-D-mannose-(1->2)]-[3-*O*-(8-triazole-Cy5-octane)-β-D-glucose-(1->3)]-β-D-mannose-cyclophelllitol (2)

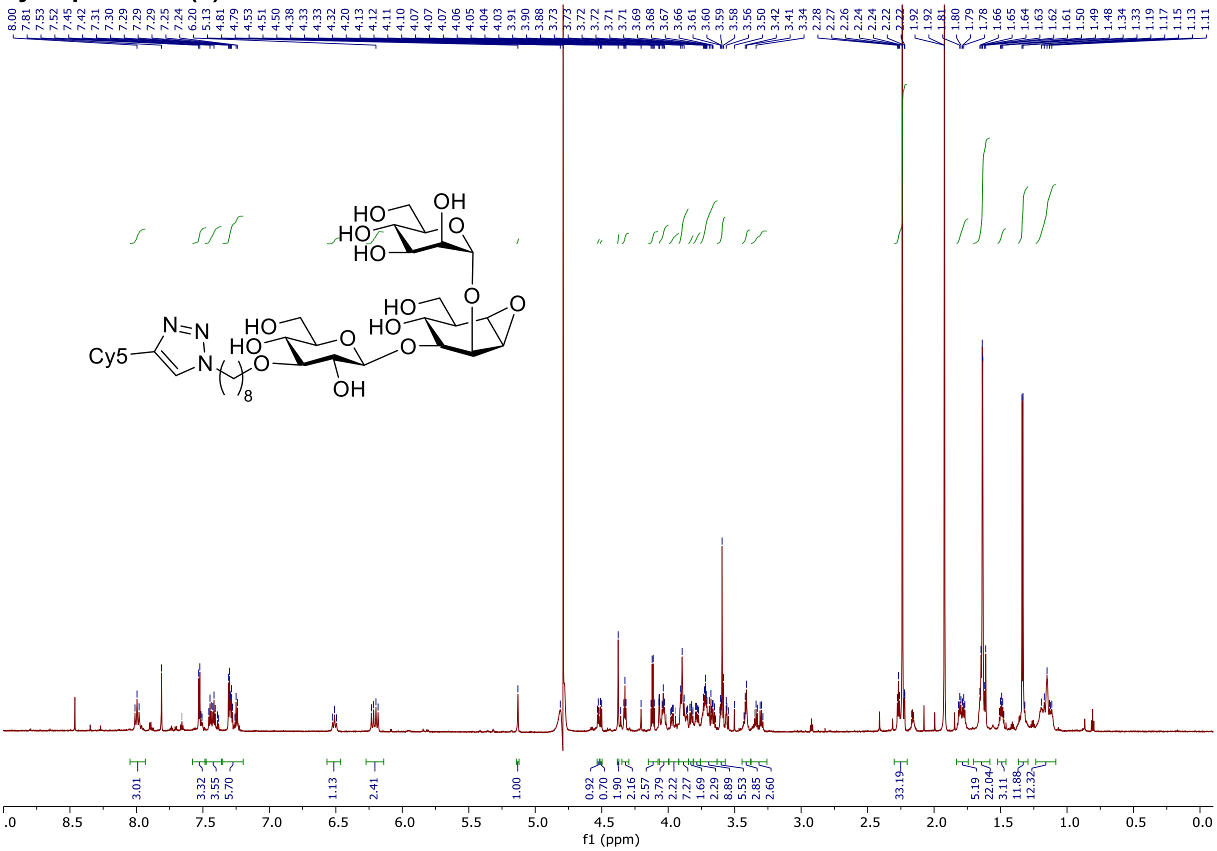

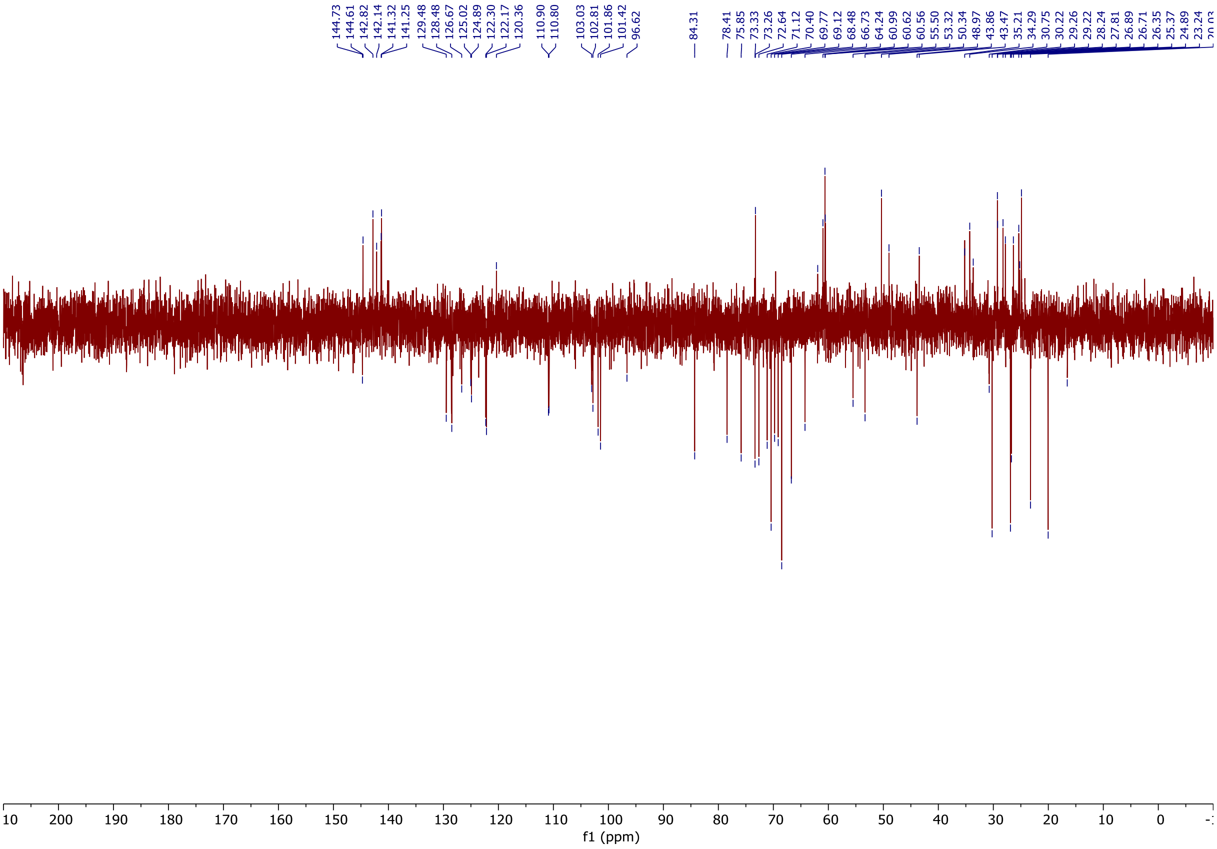

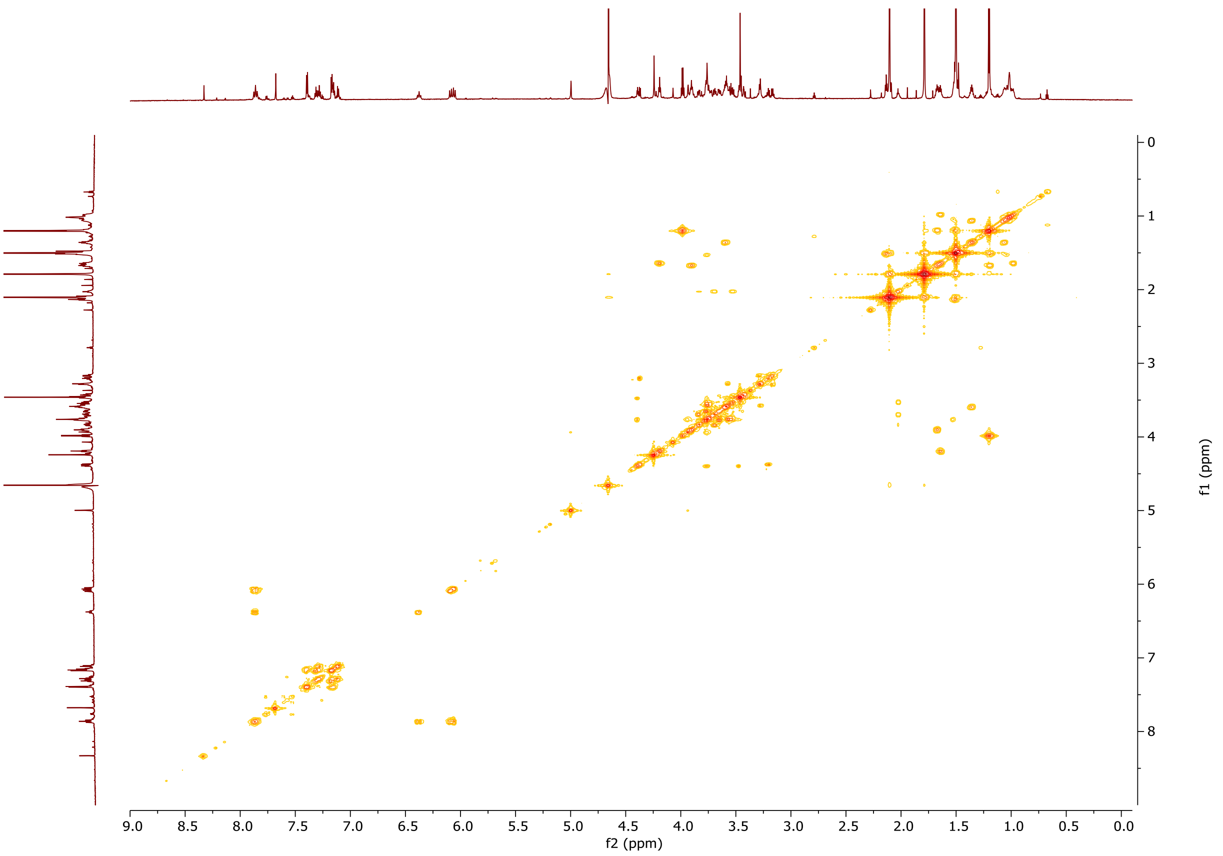

9.0 8.5 8.0 7.5 7.0 6.5 6.0 5.5 5.0 4.5 4.0 3.5 3.0 2.5 2.0 1.5 1.0 0.5 0.0
f2 (ppm)
0 1 2 3 4 5 6 7 8
f1 (ppm)

Phenyl 3-(8-azidooctyl)-4,6-*O*-ditertbutylsilyl-1-thio-α-D-mannopyranose (50a)

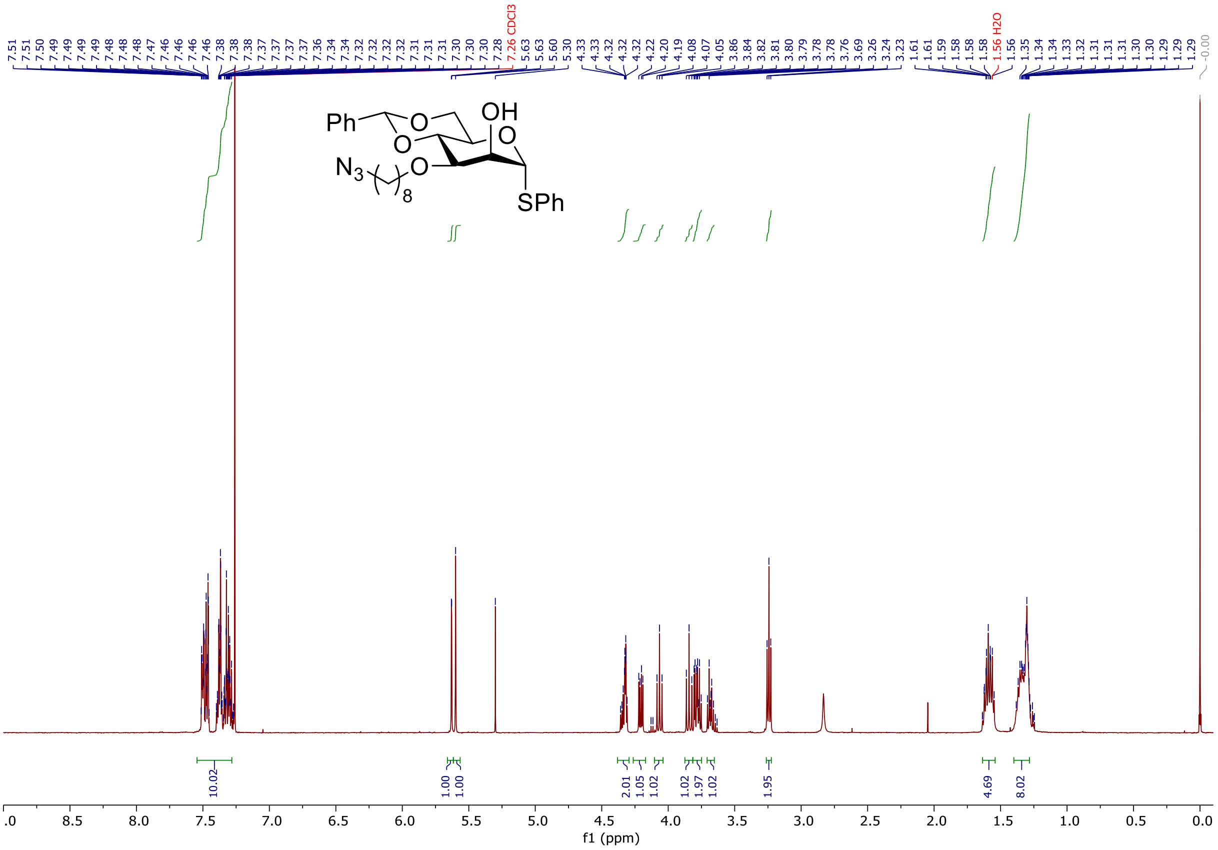

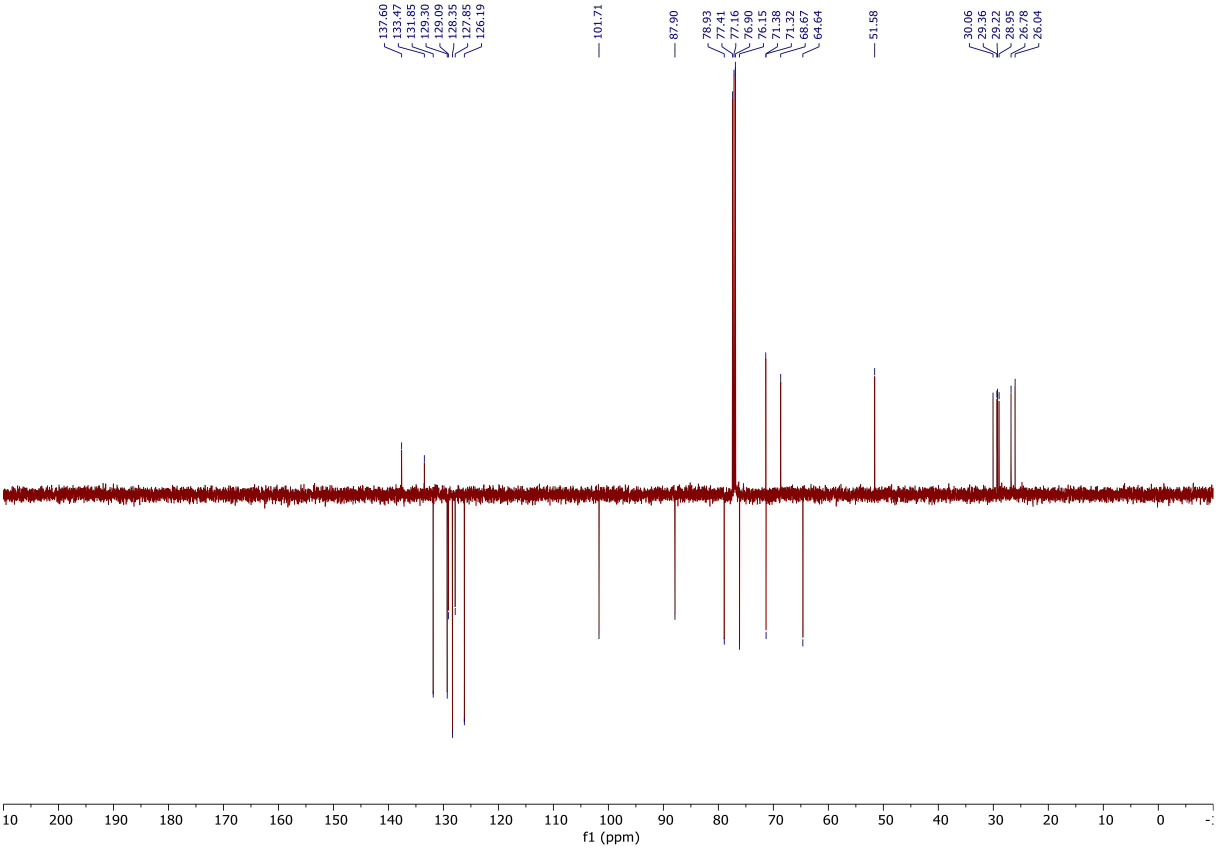

2-*O*-benzyl-4,6-*O*-benzylidene-3-*O*-(8-azido-octane)-1-deoxy-1-thiophenol-α-D-mannose (50)

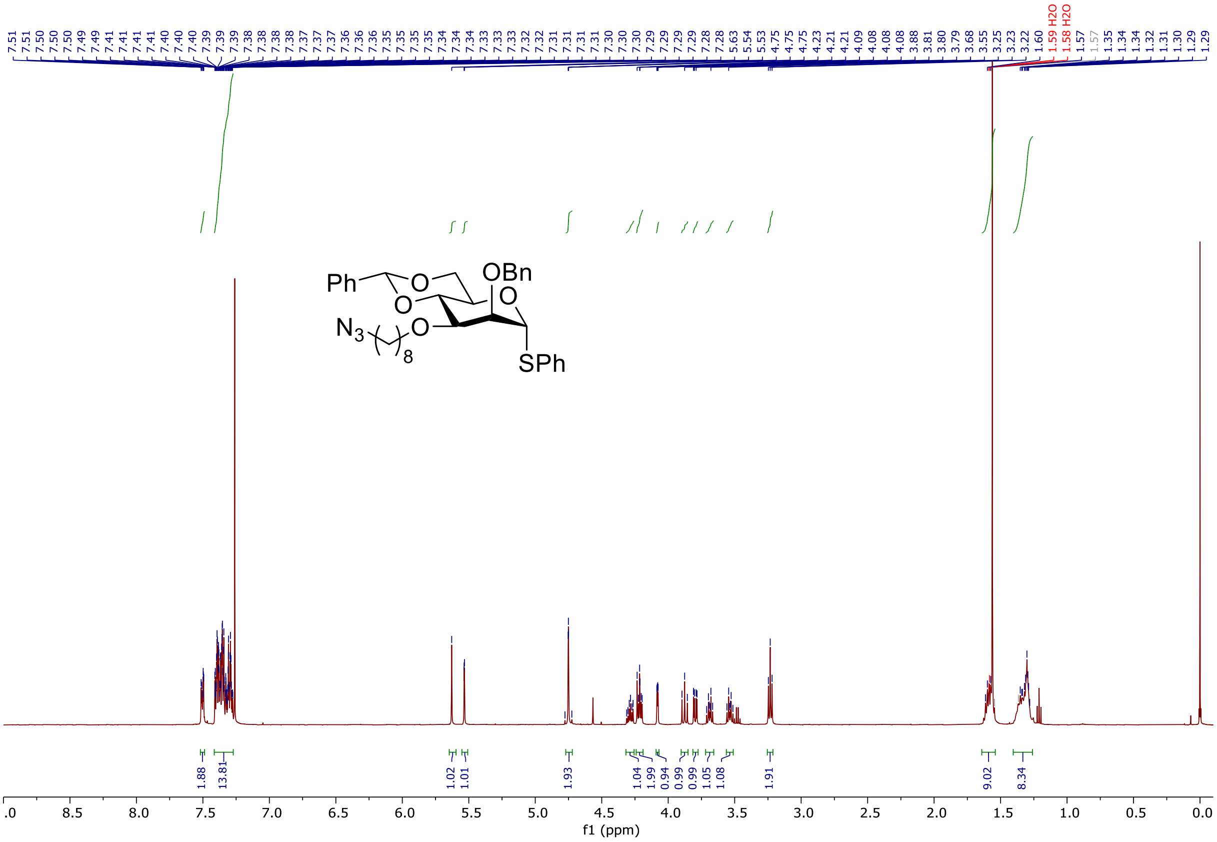

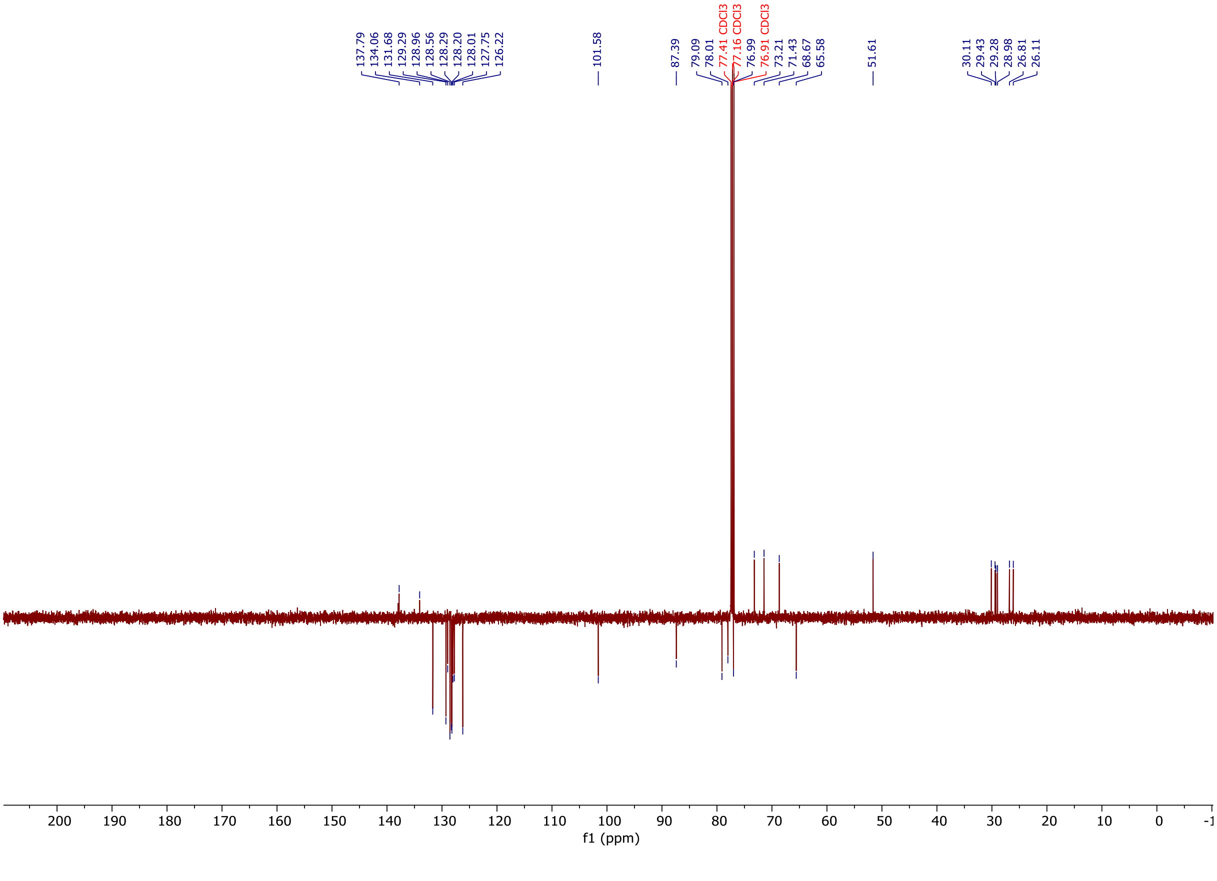

2-*O*-benzyl-4,6-*O*-benzylidene-3-*O*-(8-azido-octane)-D-mannose (51)

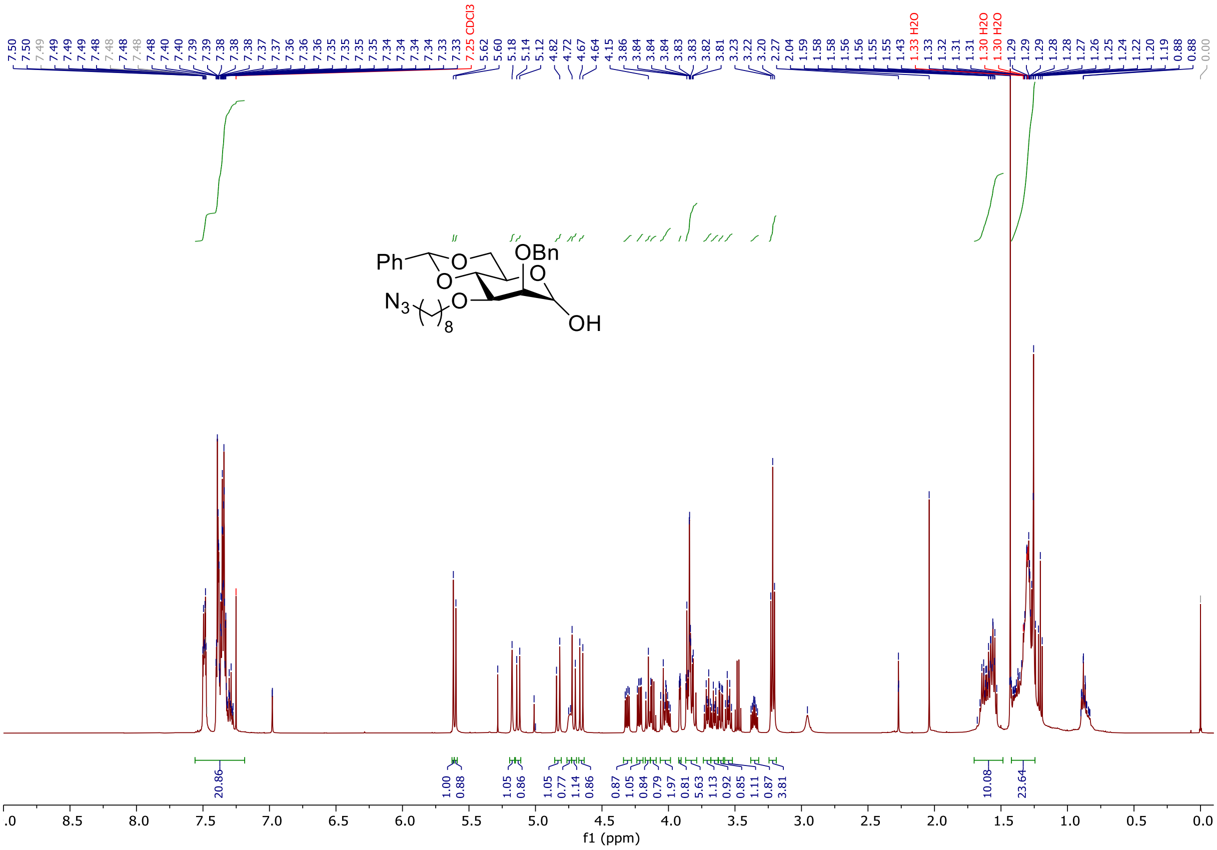

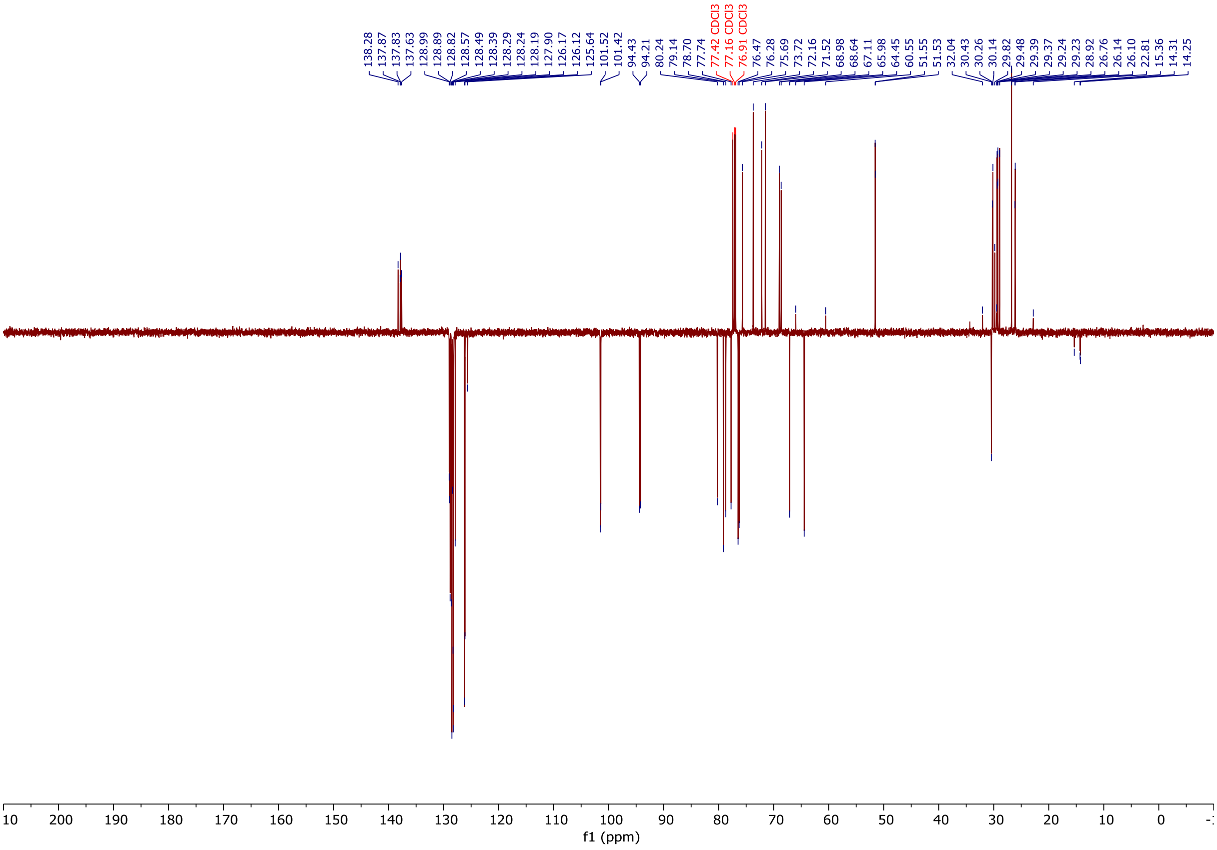

2-*O*-benzyl-4,6-*O*-benzylidene-3-*O*-(8-azido-octane)-1-*O*-(trifluoro-N-phenyl-imidate)-D-mannose (52)

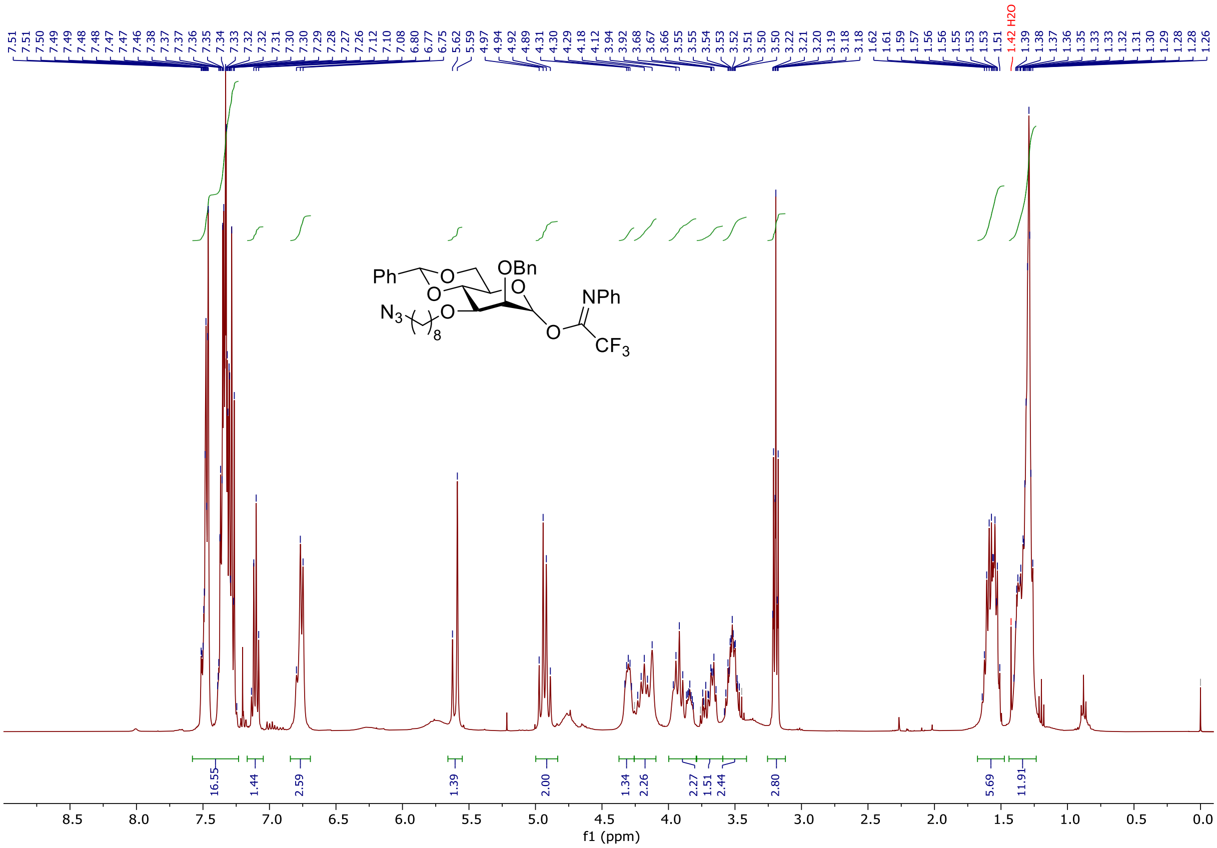

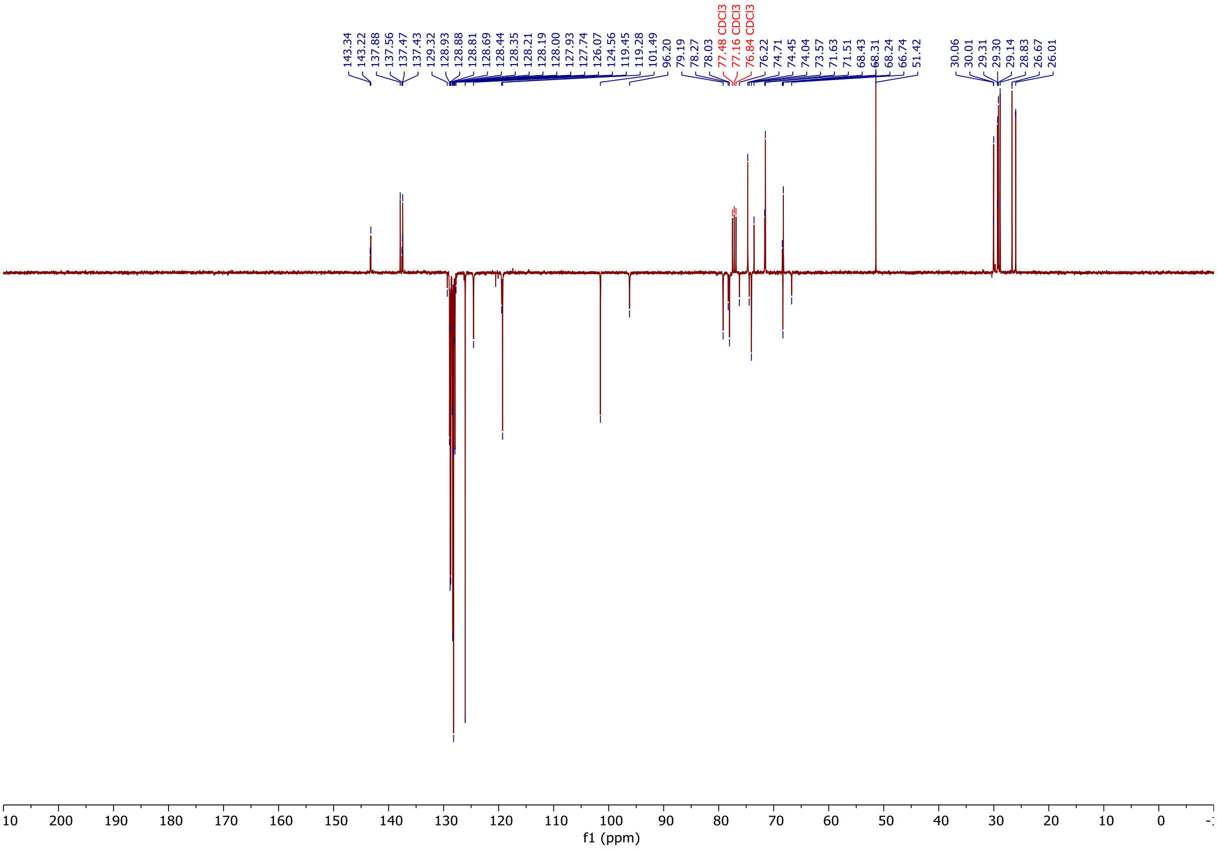

1-*O*-methyl-5-*O*-trityl-L-ribofuranose (53)

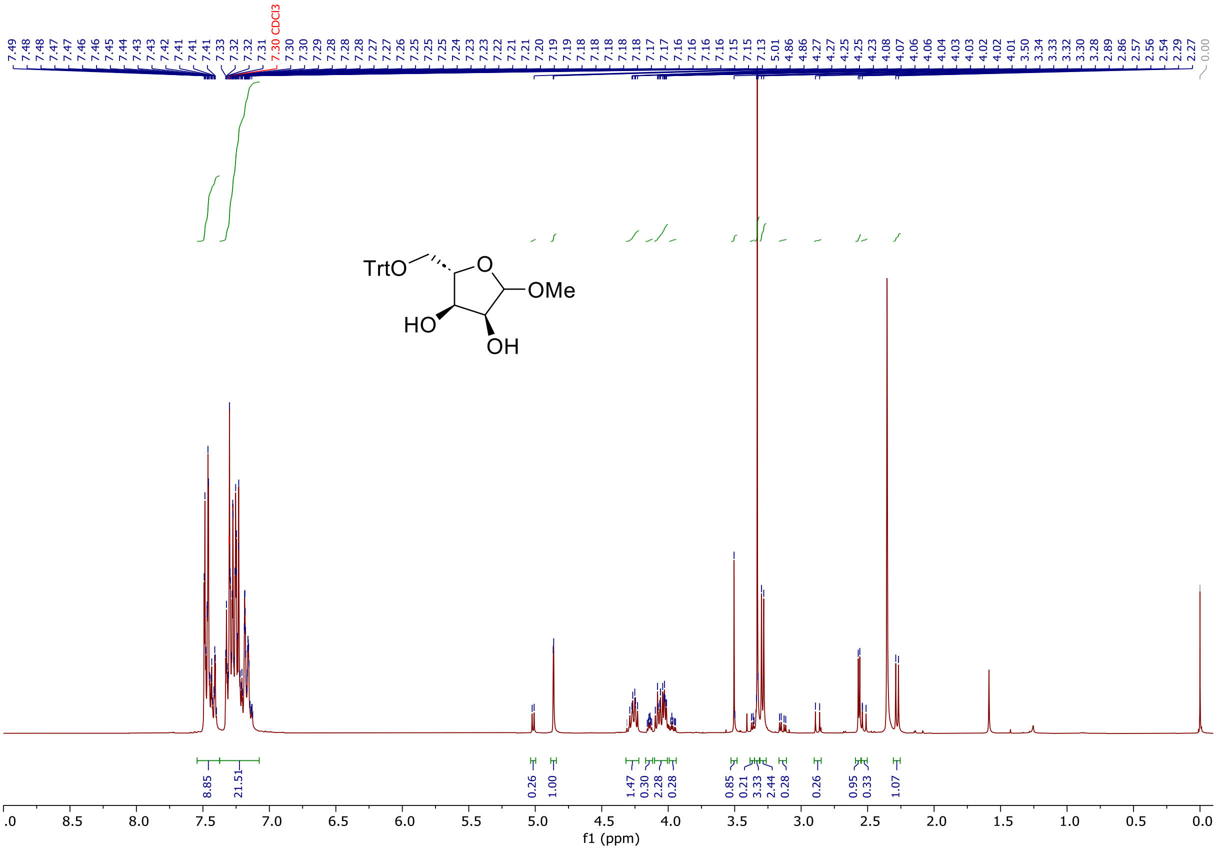

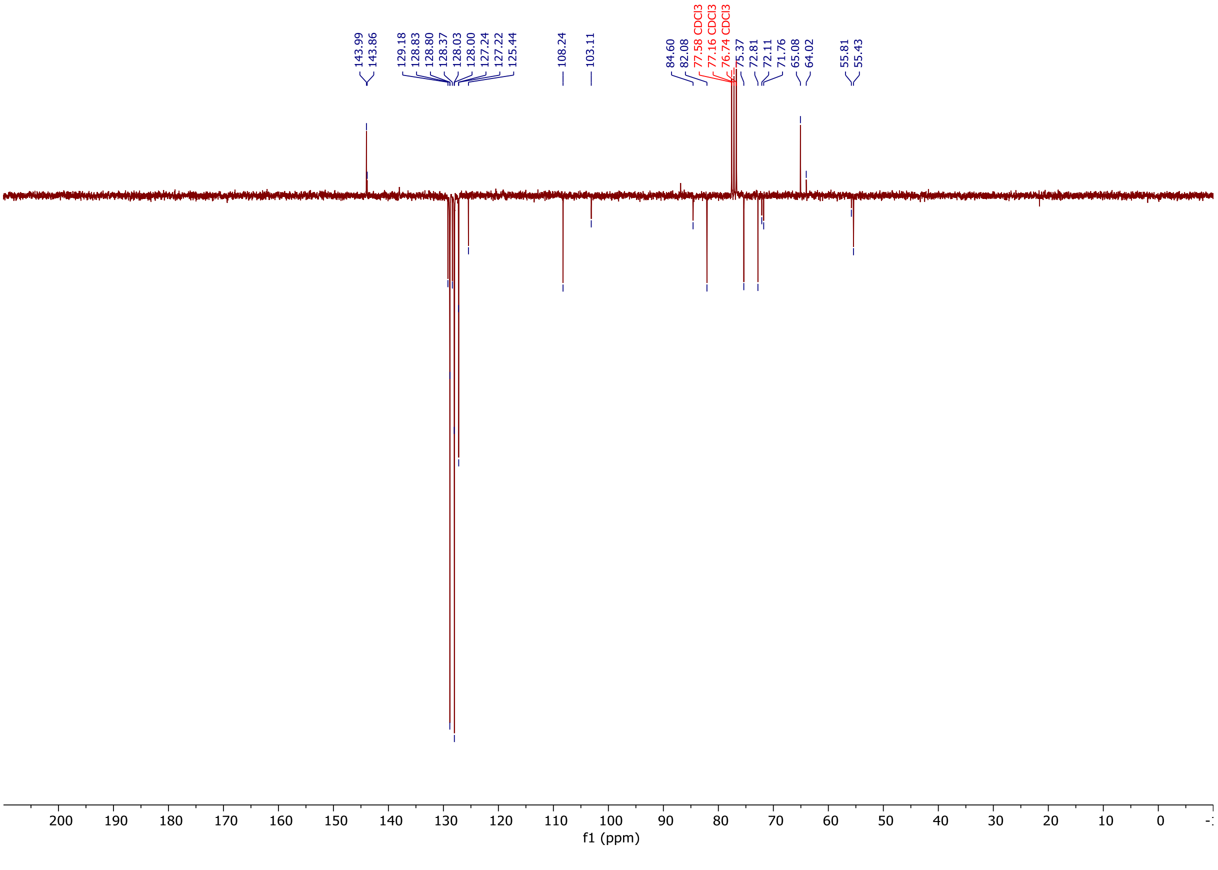

1-*O*-methyl-2,3-di-*O*-benzyl-5-*O*-trityl-L-ribofuranose (54)

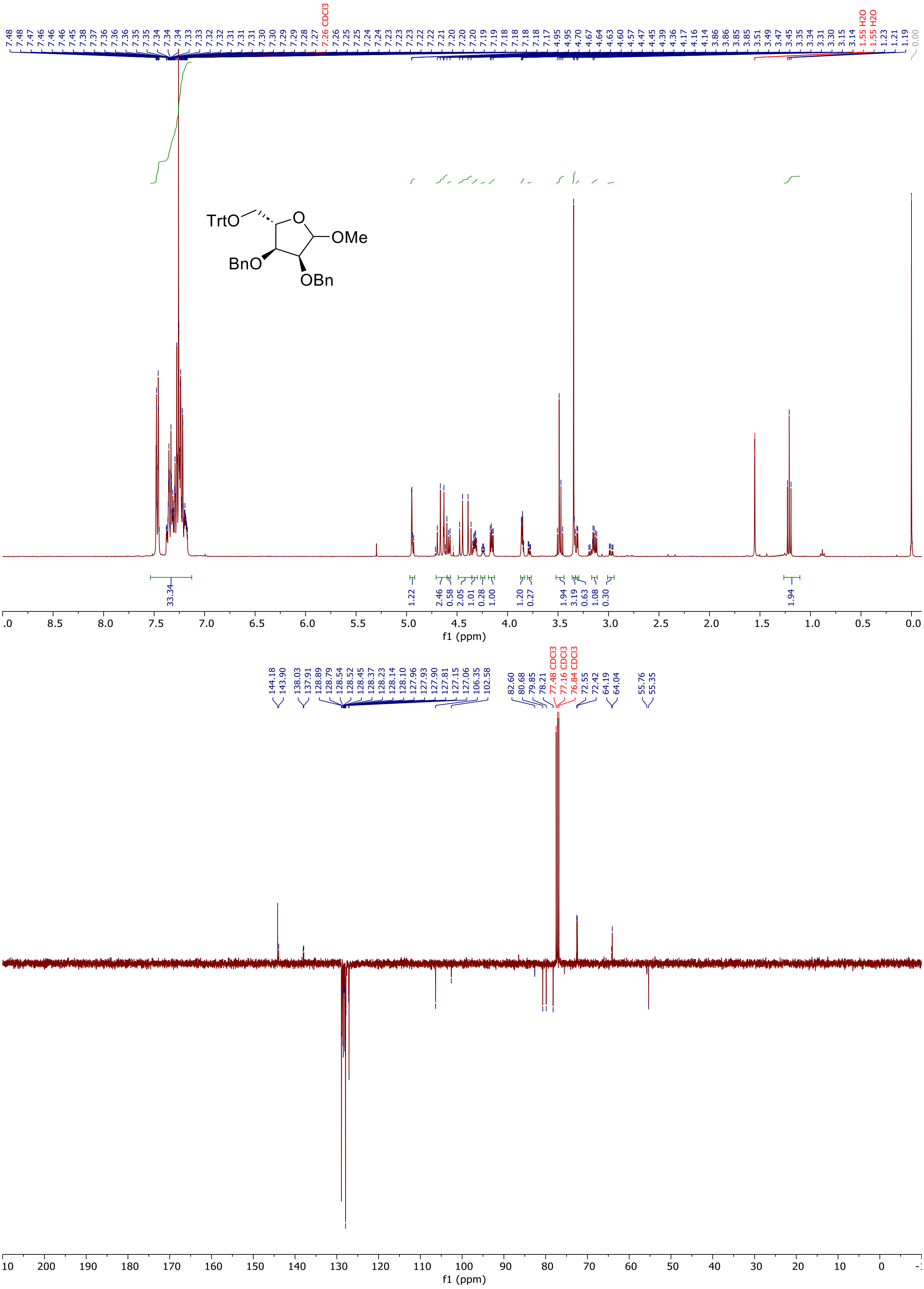

1-*O*-methyl-2,3-di-*O*-benzyl-L-Ribofuranose (55)

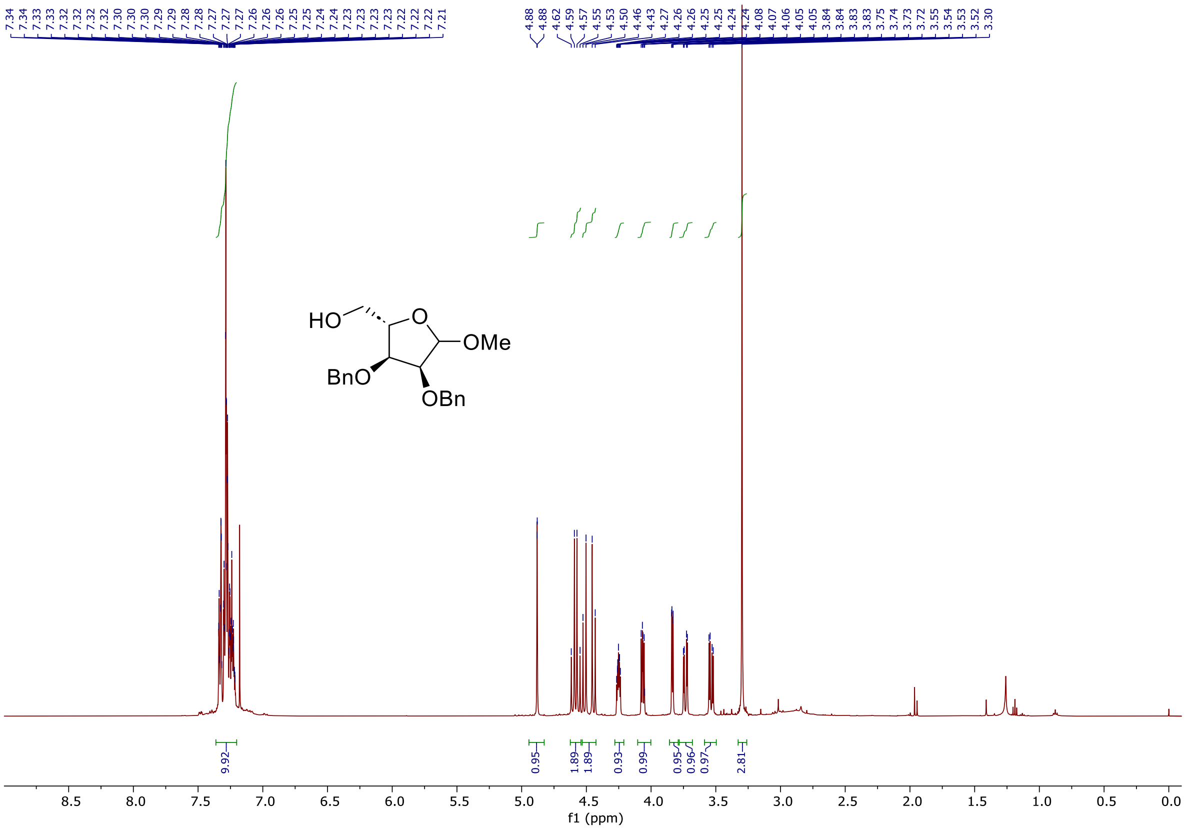

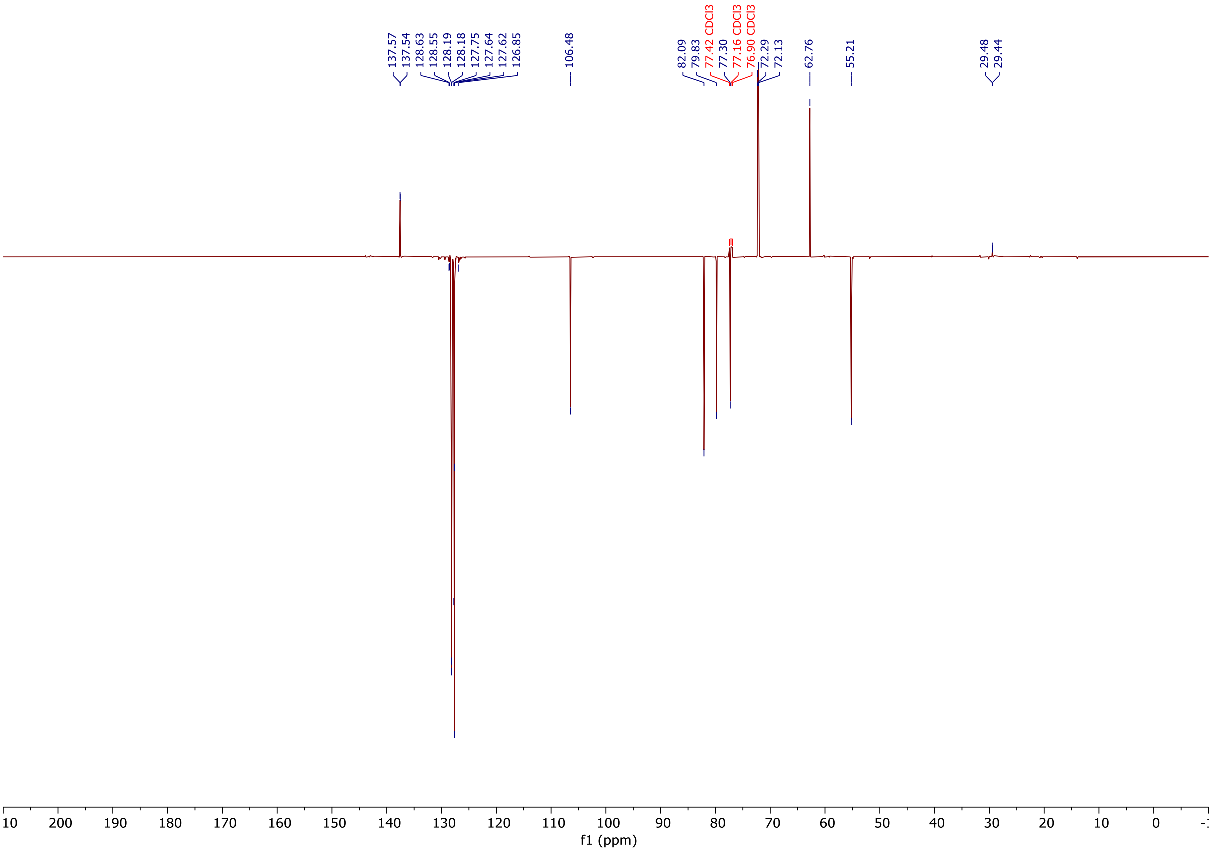

1-*O*-methyl-2,3-di-*O*-benzyl-5-deoxy-5-iodo-L-ribofuranose (56)

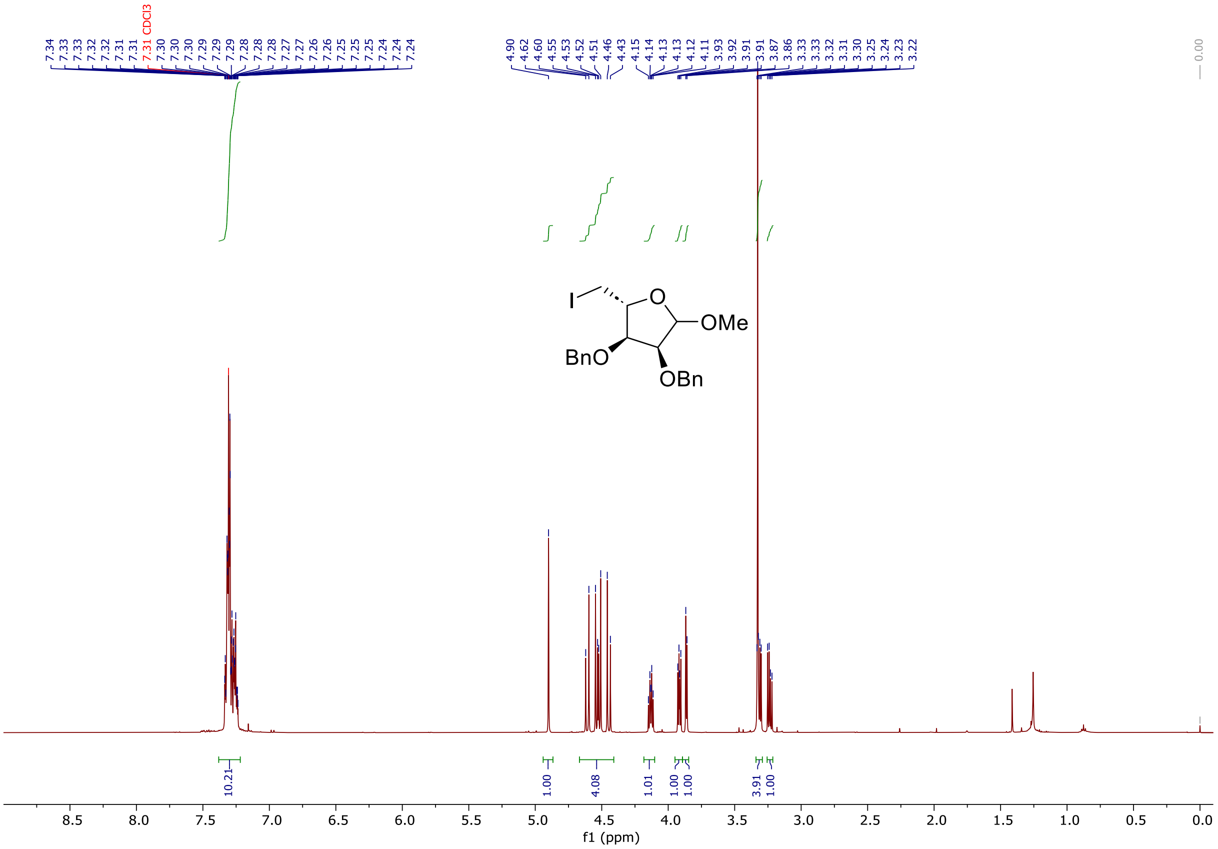

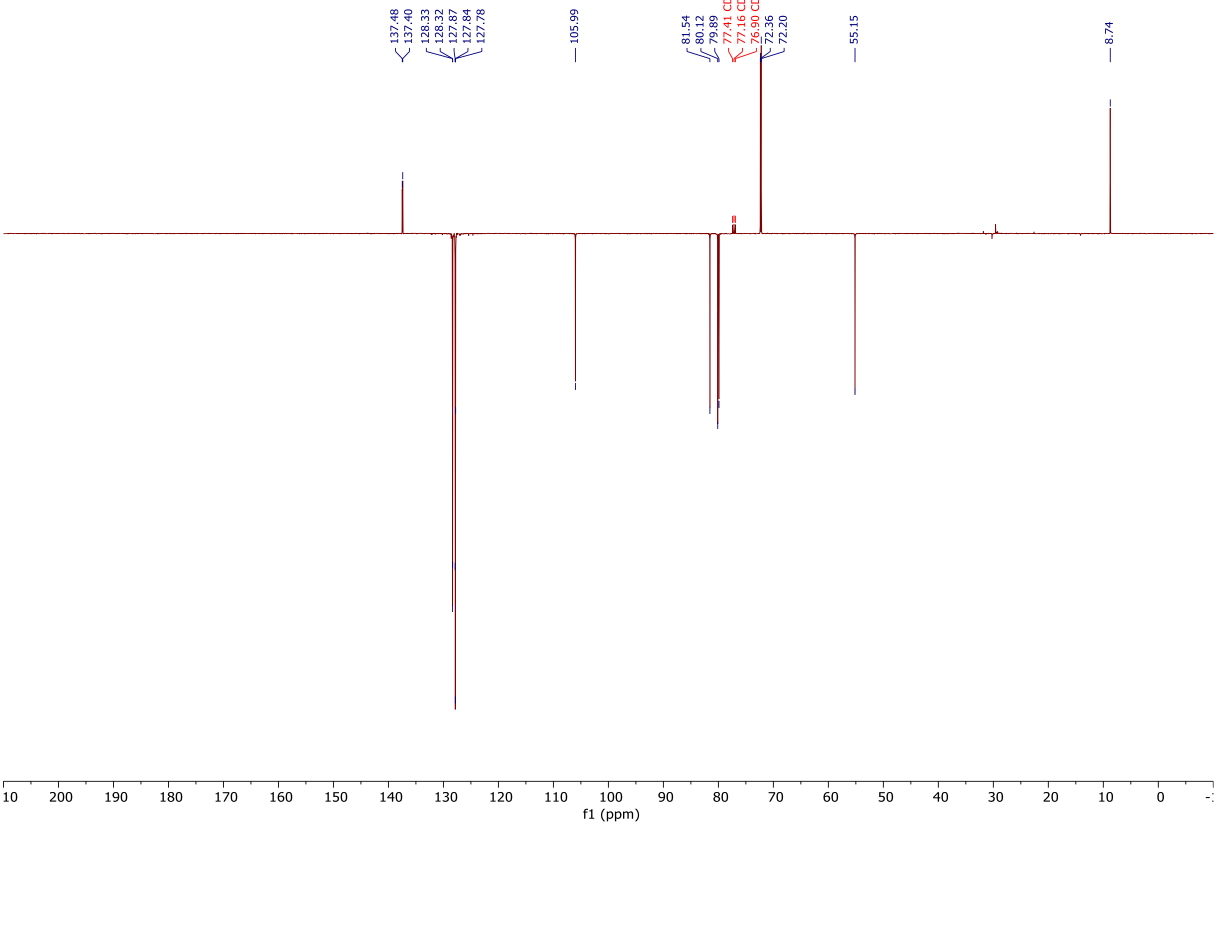

(2S,3S)-2,3-bis(benzyloxy)pent-4-enal (57)

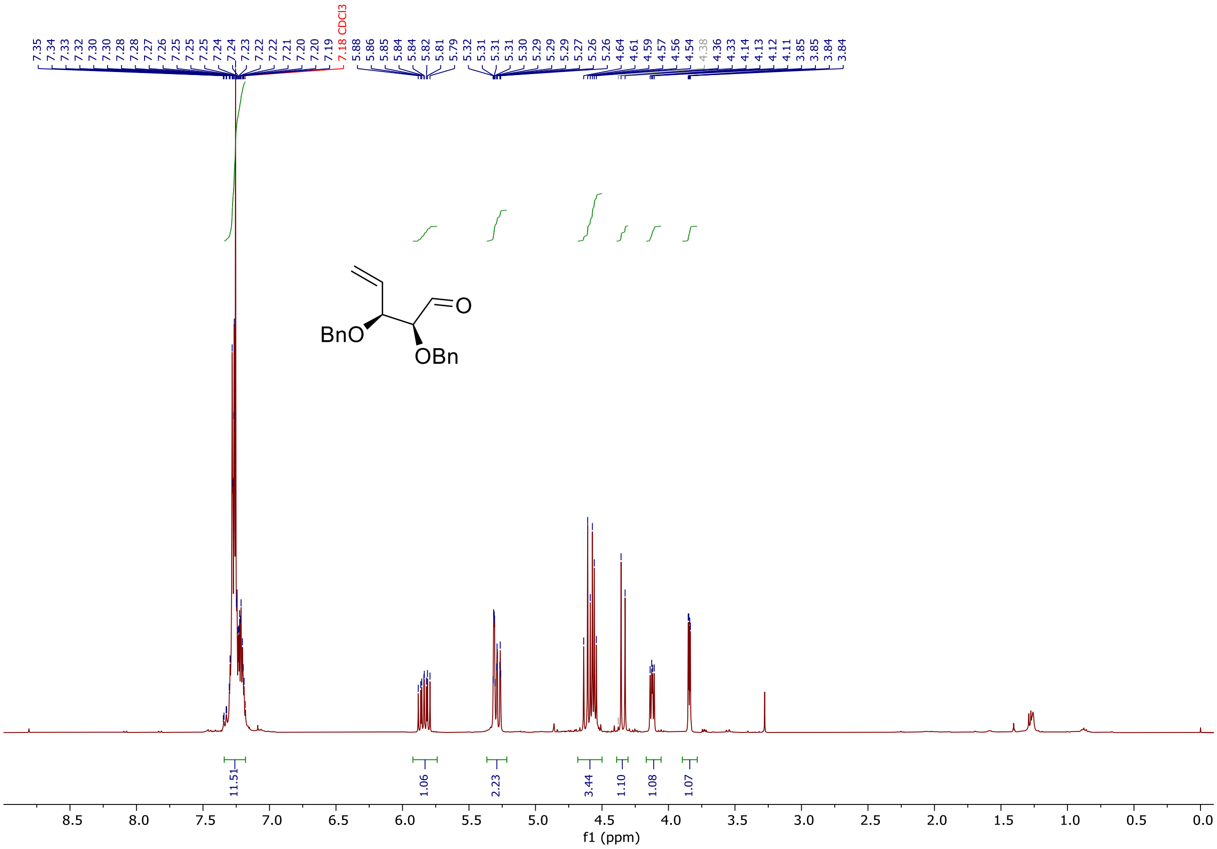

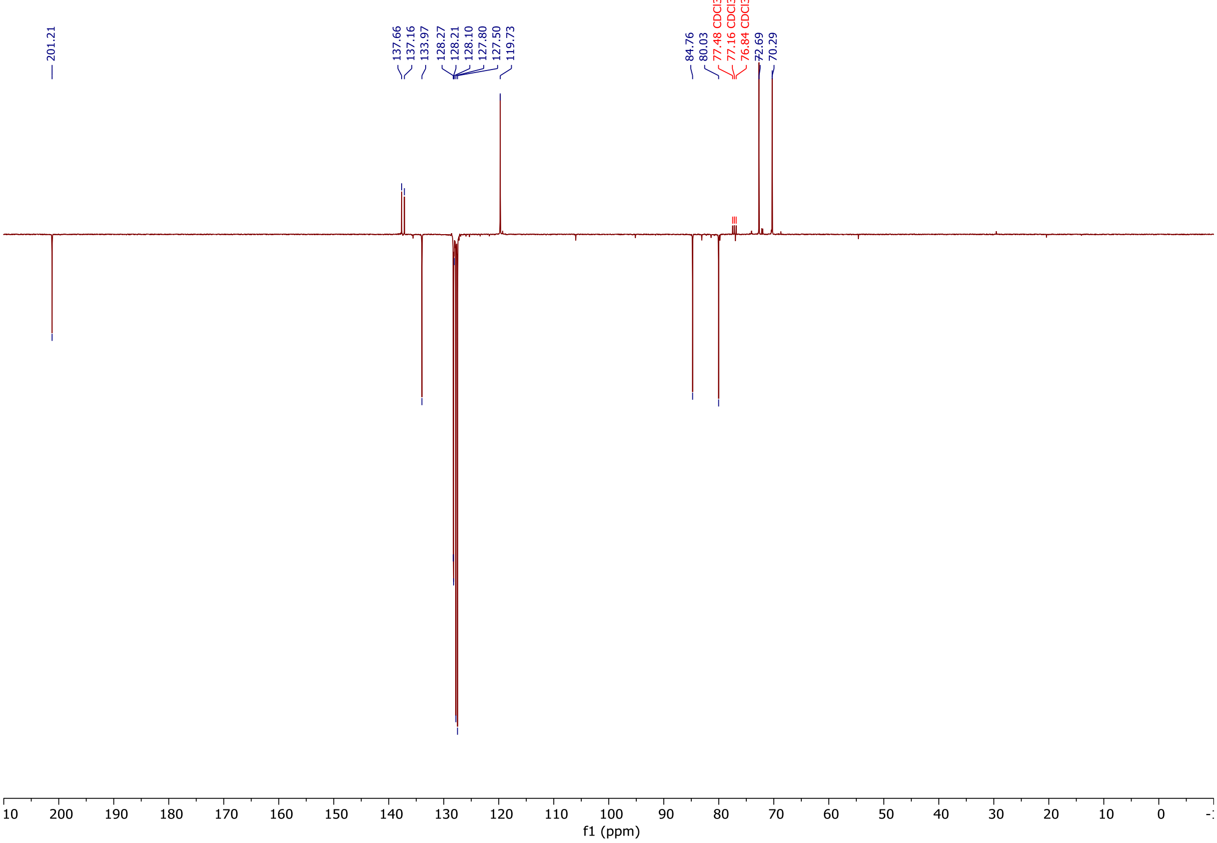

Alkene (58)

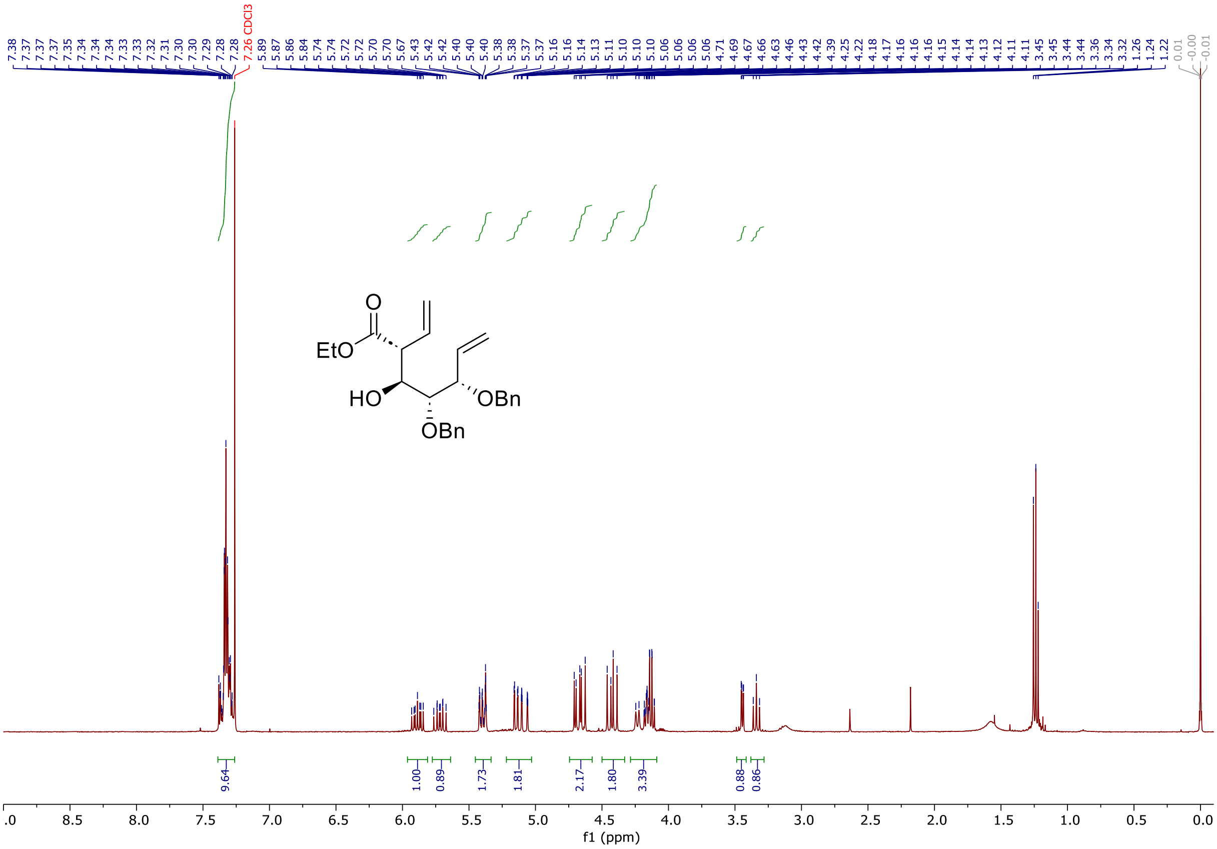
7.38 7.37 7.37 7.35 7.34 7.34 7.33 7.33 7.32 7.31 7.30 7.30 7.29 7.28 7.28
7.26 CDCl3
5.89 5.87 5.86 5.84 5.74 5.74 5.72 5.72 5.70 5.70 5.67 5.43 5.42 5.42 5.40 5.40 5.38 5.38 5.37 5.37 5.16 5.16 5.14 5.13 5.11 5.10 5.10 5.10 5.06 5.06 5.06 5.06 4.71 4.69 4.67 4.66 4.63 4.46 4.43 4.42 4.39 4.25 4.22 4.18 4.17 4.16 4.16 4.16 4.15 4.14 4.14 4.13 4.12 4.11 4.11 3.45 3.45 3.44 3.36 3.34 3.32 1.26 1.24 1.22
0.01 -0.00 -0.01
O
EtO
HO
OBn
OBn
9.64 1.00 0.89 1.73 1.81 2.17 1.80 3.39 0.88 0.86
8.5 8.0 7.5 7.0 6.5 6.0 5.5 5.0 4.5 4.0 3.5 3.0 2.5 2.0 1.5 1.0 0.5 0.0
f1 (ppm)

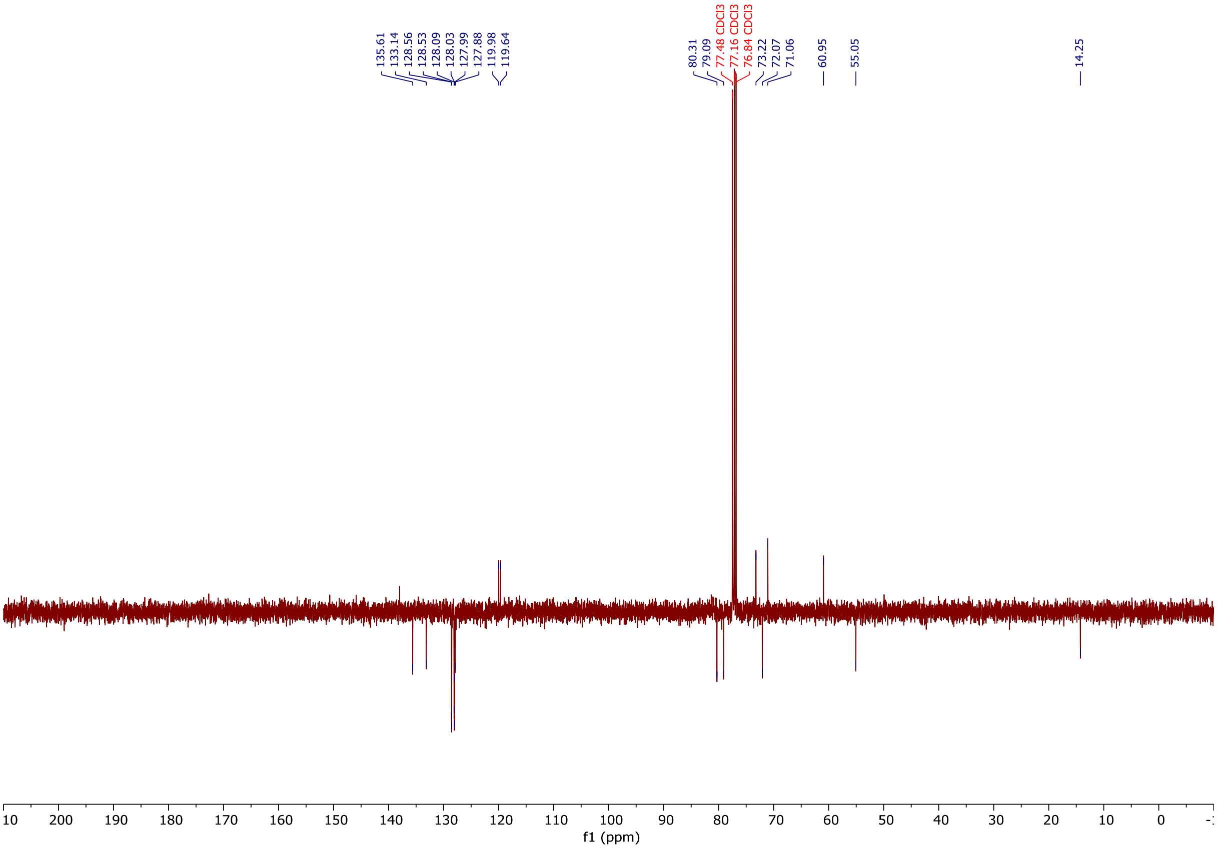
135.61 133.14 128.56 128.53 128.09 128.03 127.99 127.88 119.98 119.64
80.31 79.09
77.48 CDCl3 77.16 CDCl3 76.84 CDCl3
73.22 72.07 71.06
60.95
55.05
14.25
200 190 180 170 160 150 140 130 120 110 100 90 80 70 60 50 40 30 20 10 0
f1 (ppm)

2,3-di-*O*-benzyl-6-ethanoate-L-mannose-cyclohexene (58a)

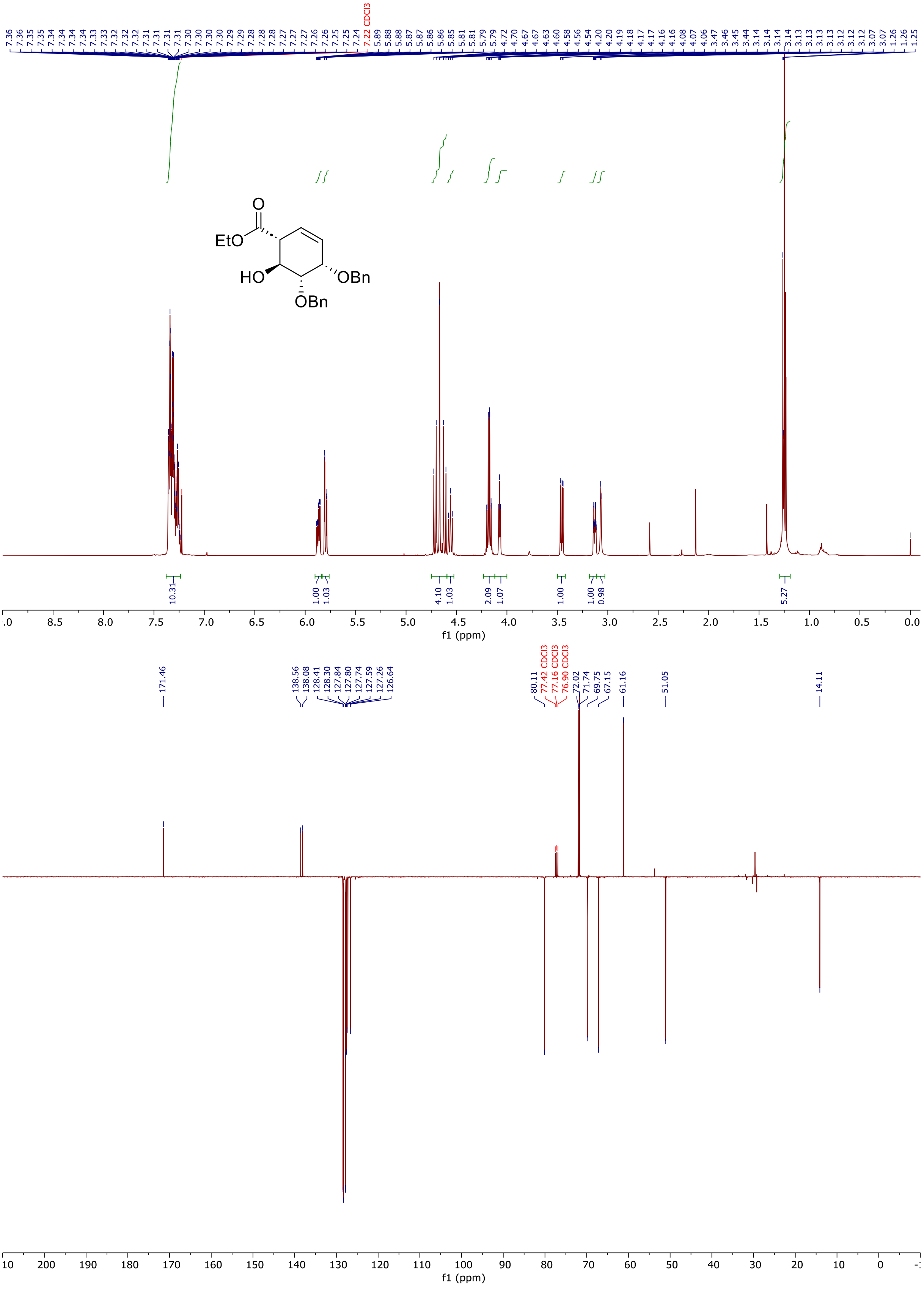

2,3-di-*O*-benzyl-L-mannose-cyclohexene (59)

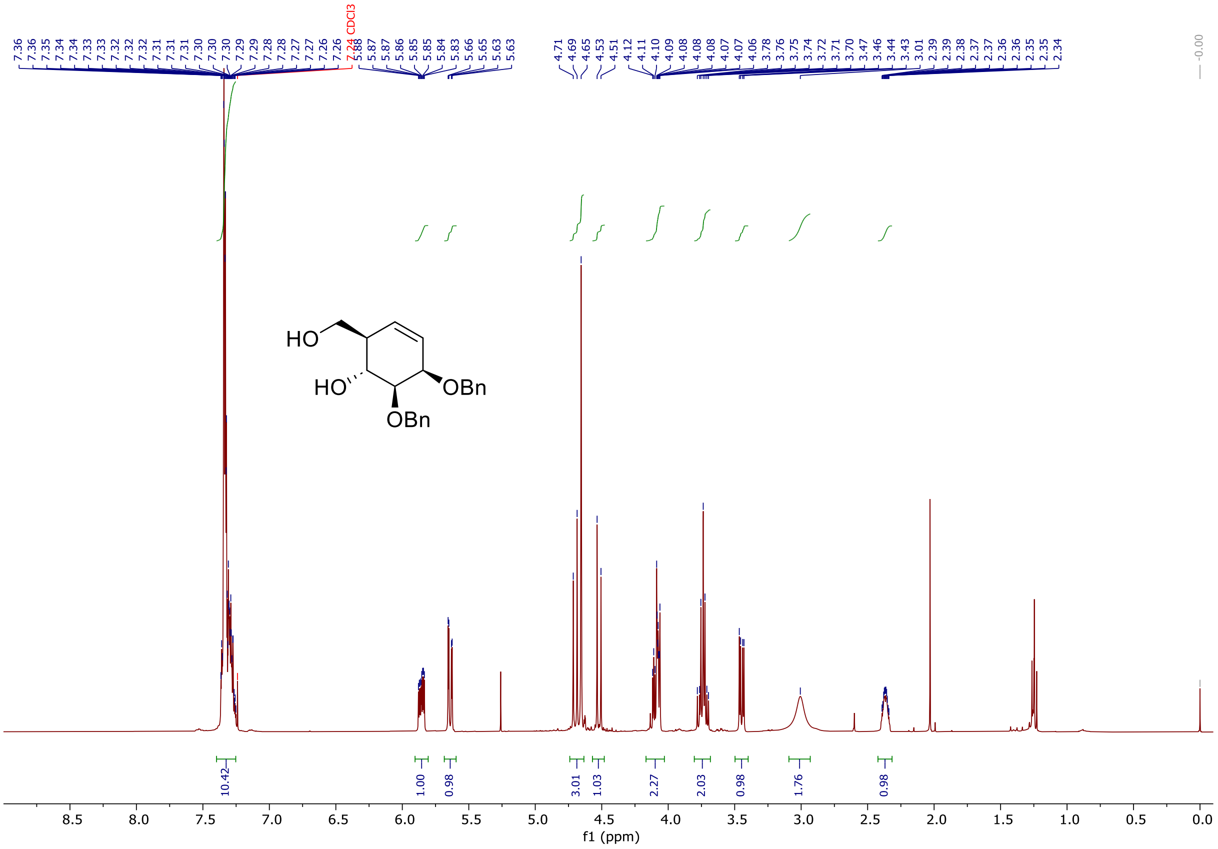

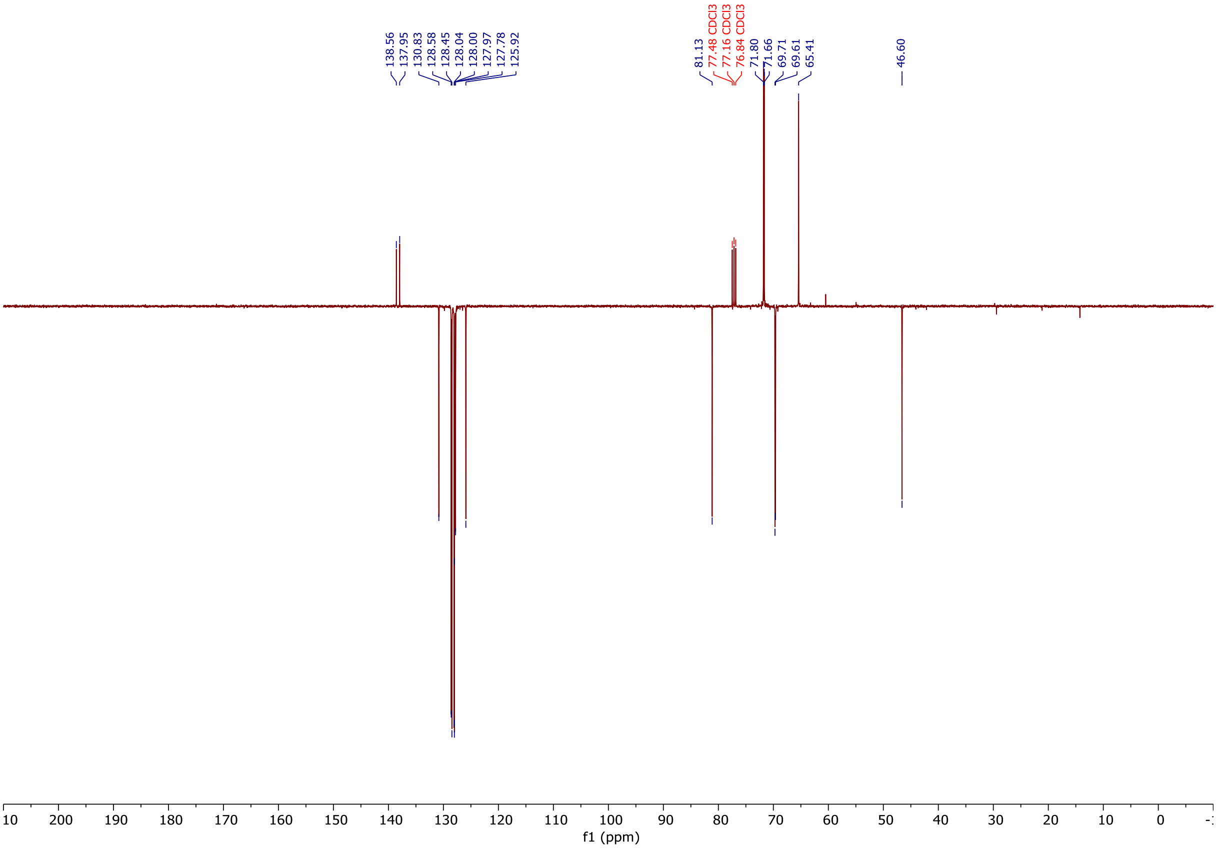

2,3-di-*O*-benzyl-6-*O*-tosyl-L-mannose-cyclohexene (59a)

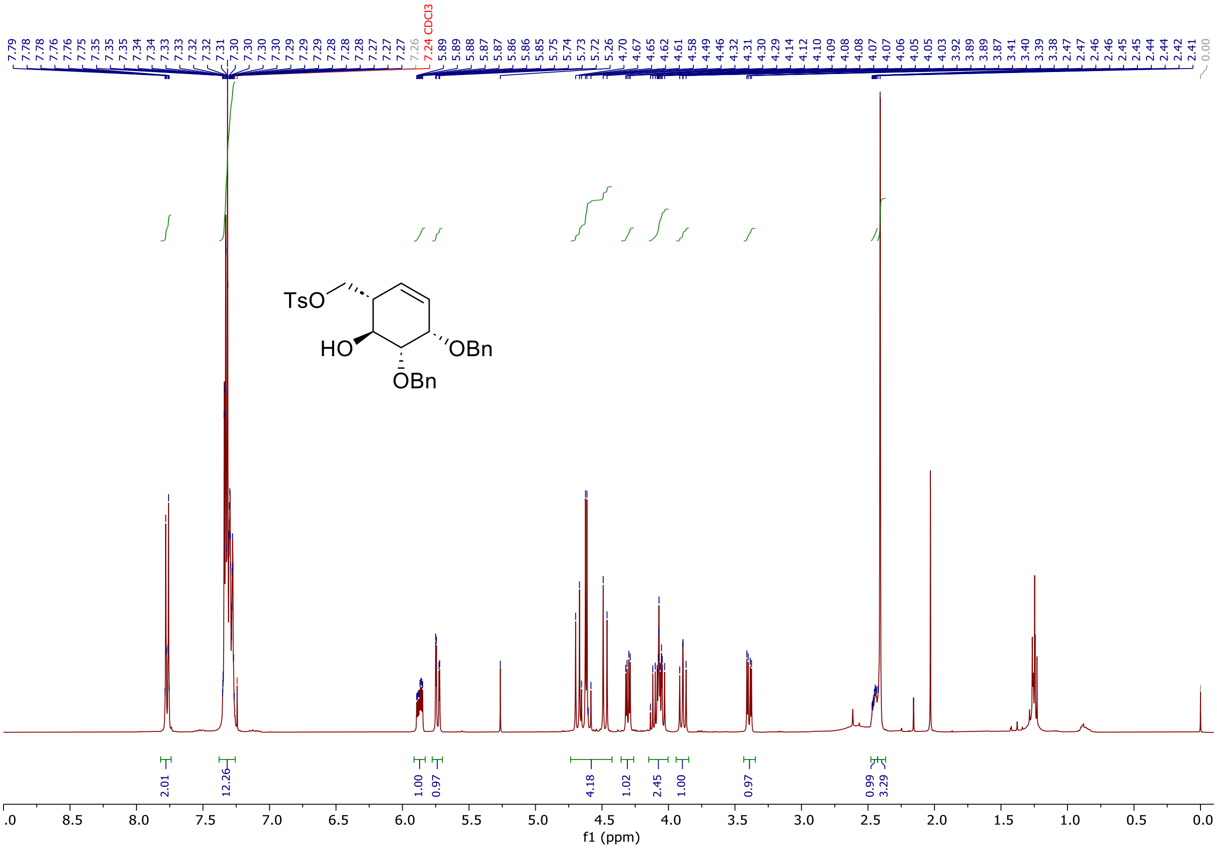

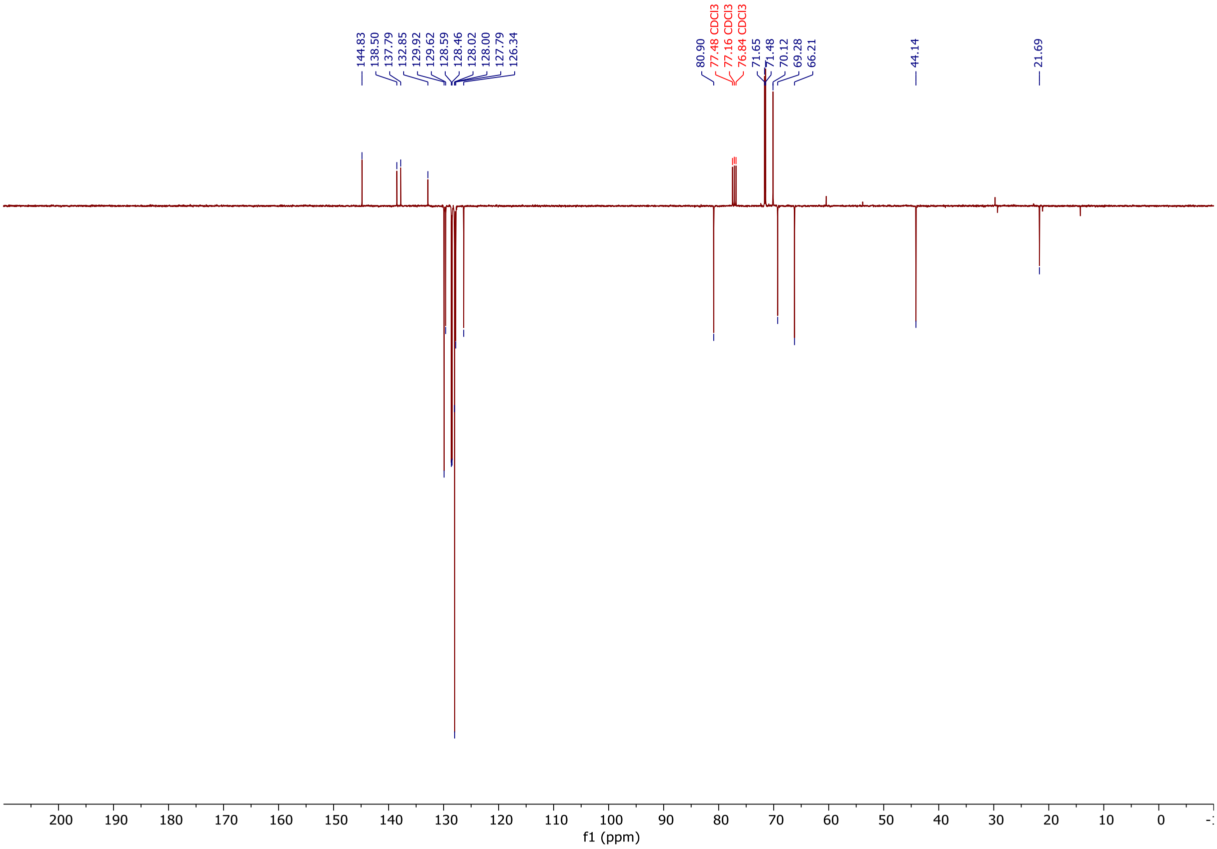

2,3-di-*O*-benzyl-L-rhamnose-cyclohexene (60)

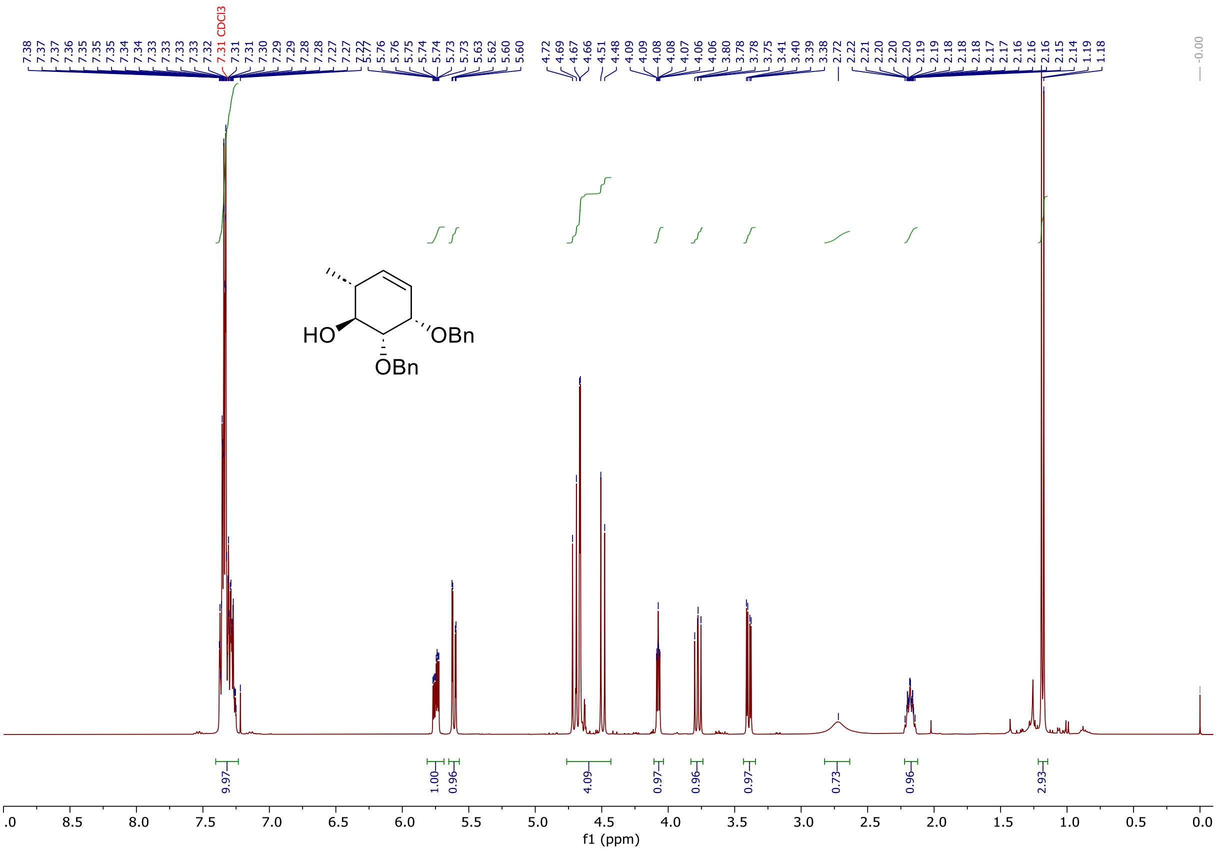

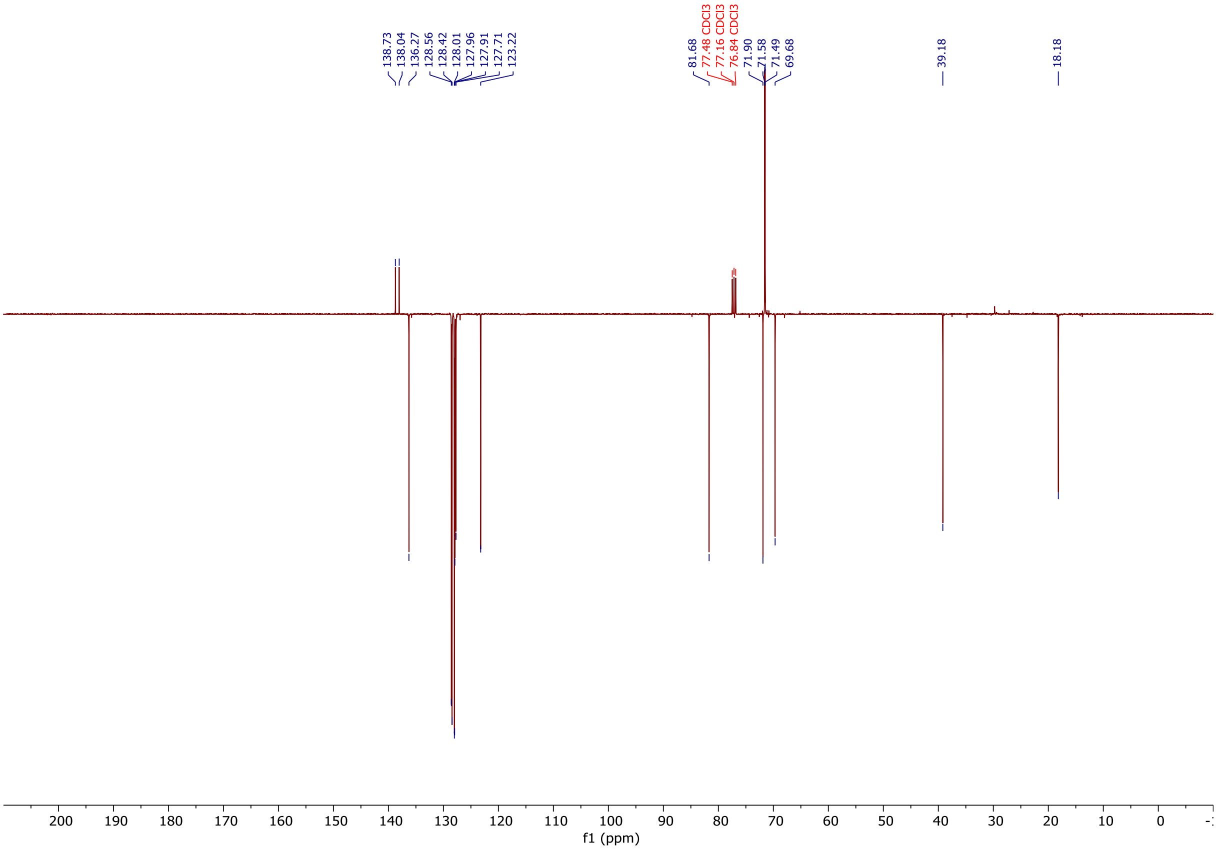

2,3-di-*O*-benzyl-4-*O*-TIPS-L-rhamnose-cyclohexene (61)

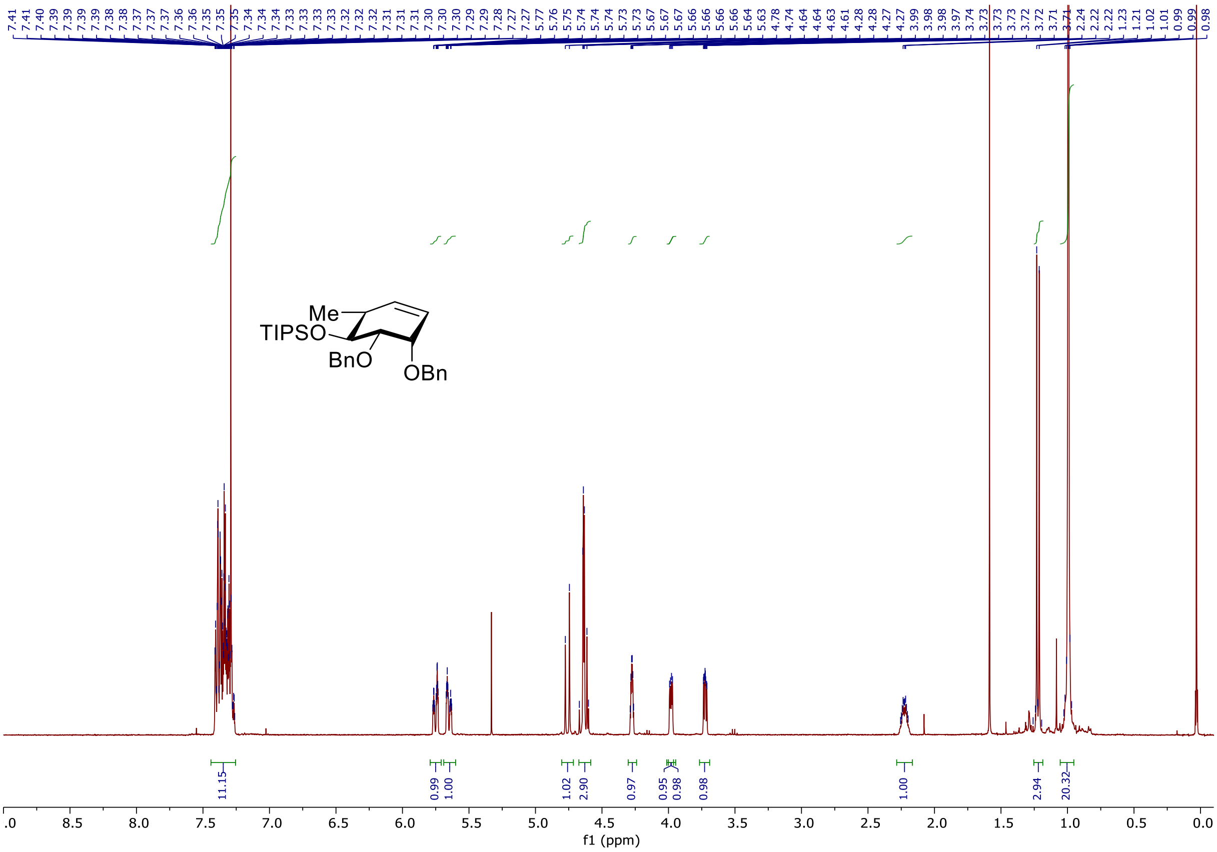

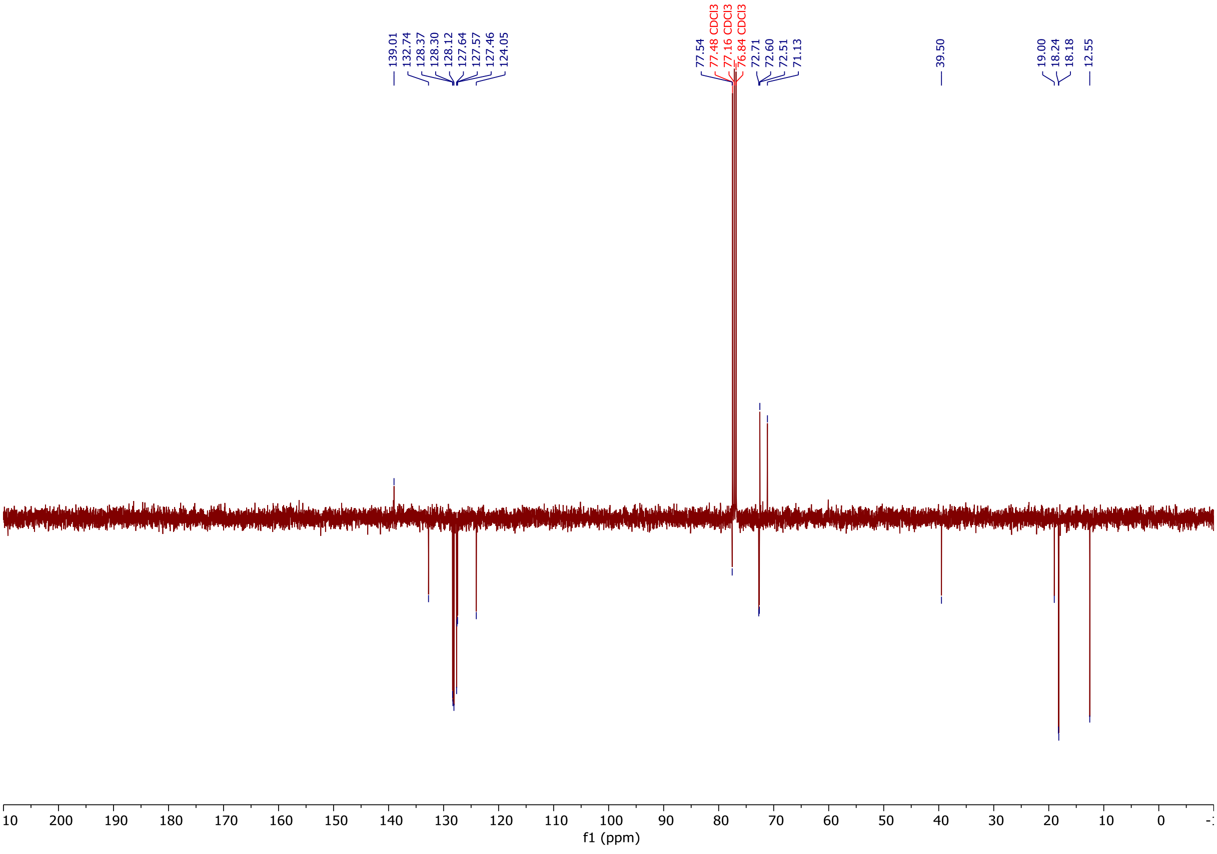

4-*O*-TIPS-rhamnose-L-cyclohexene (62)

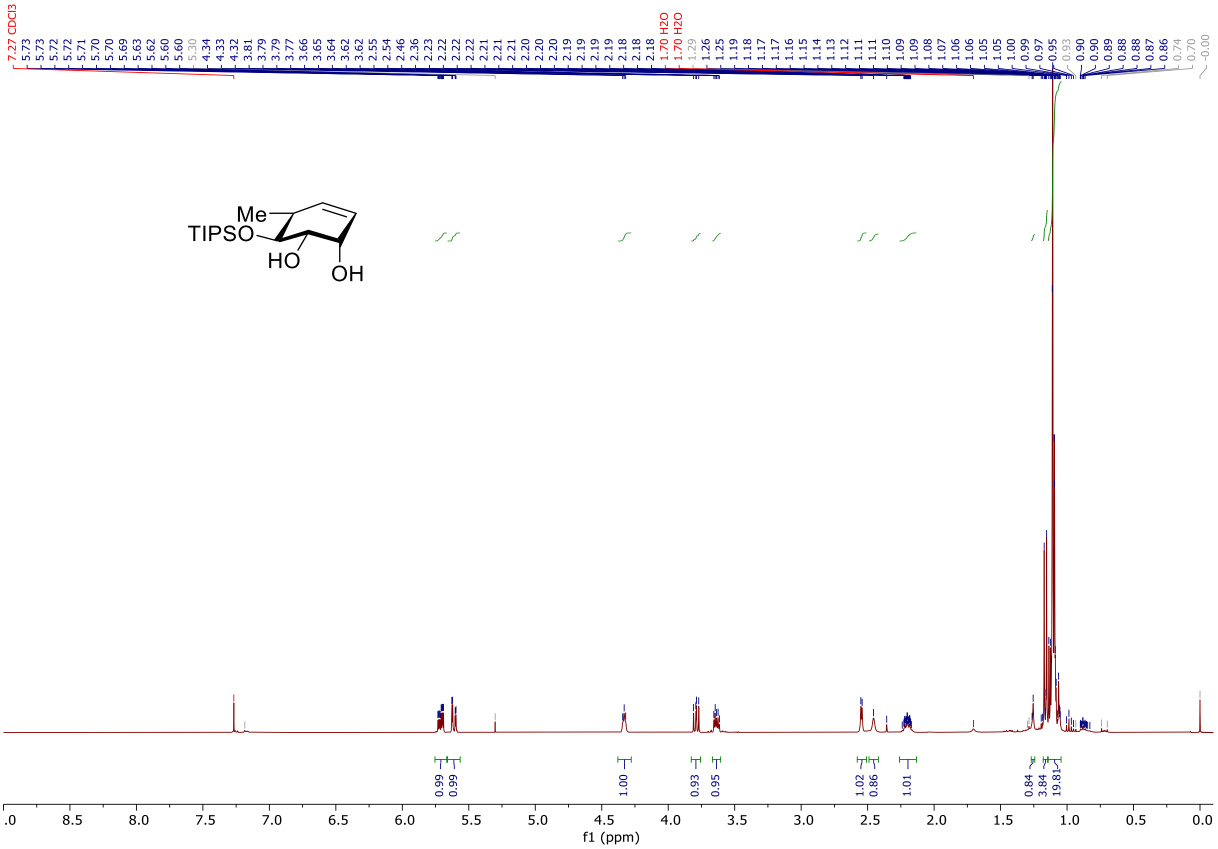

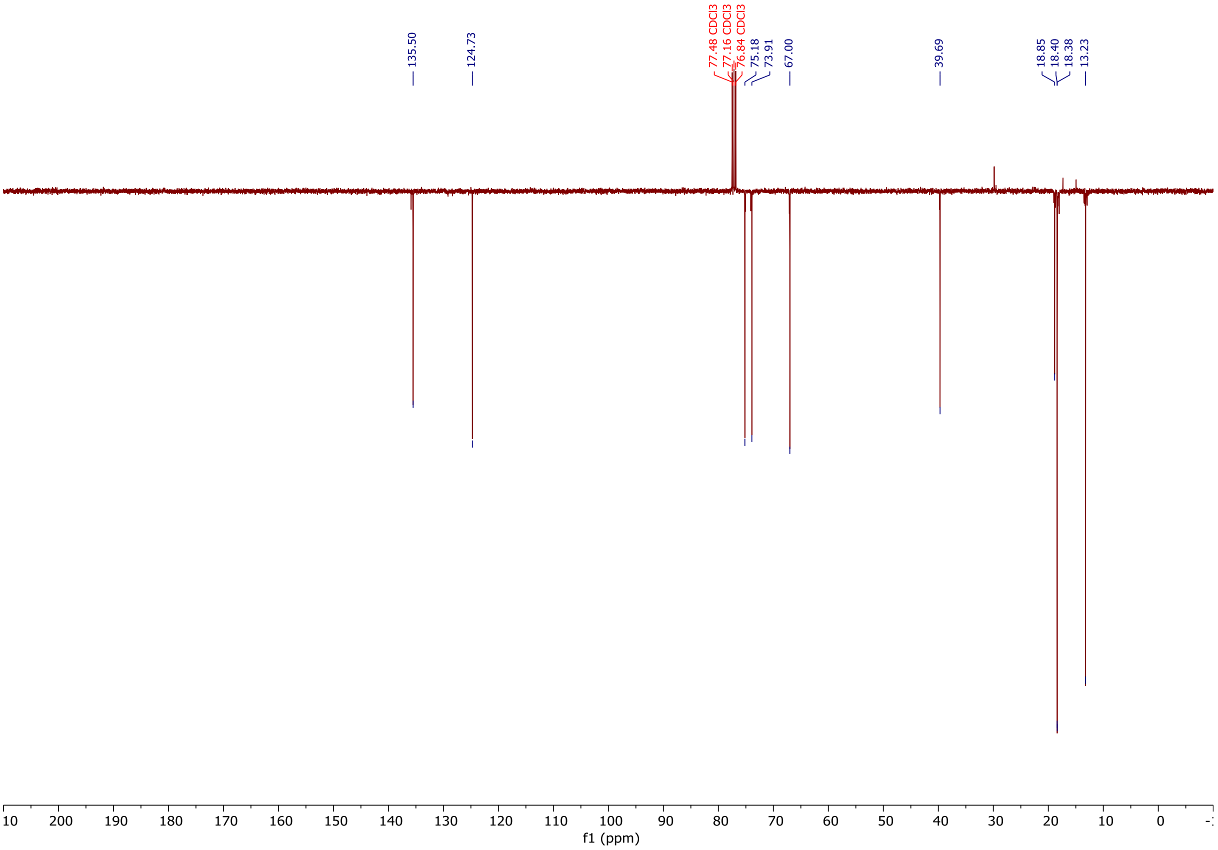

2-*O*-benzoyl-4-*O*-TIPS-L-rhamnose-cyclohexene (63)

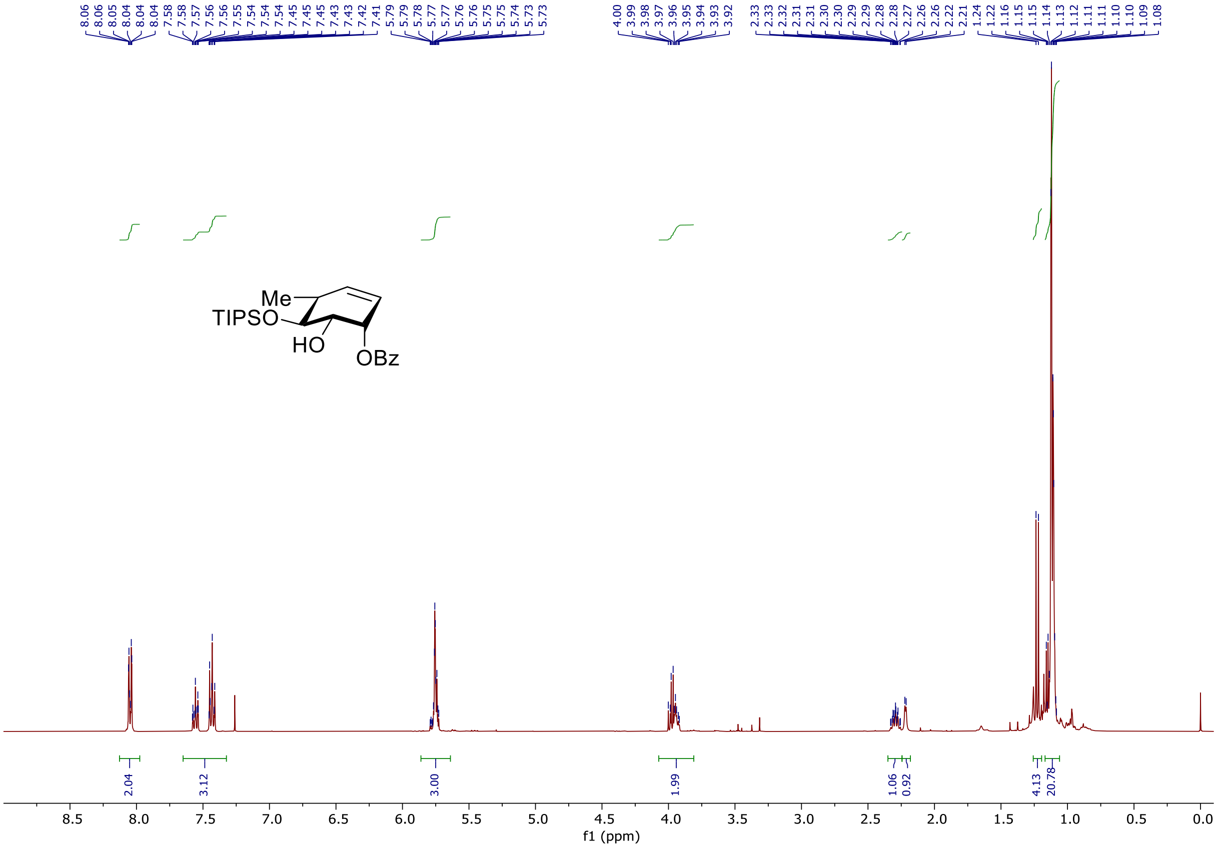

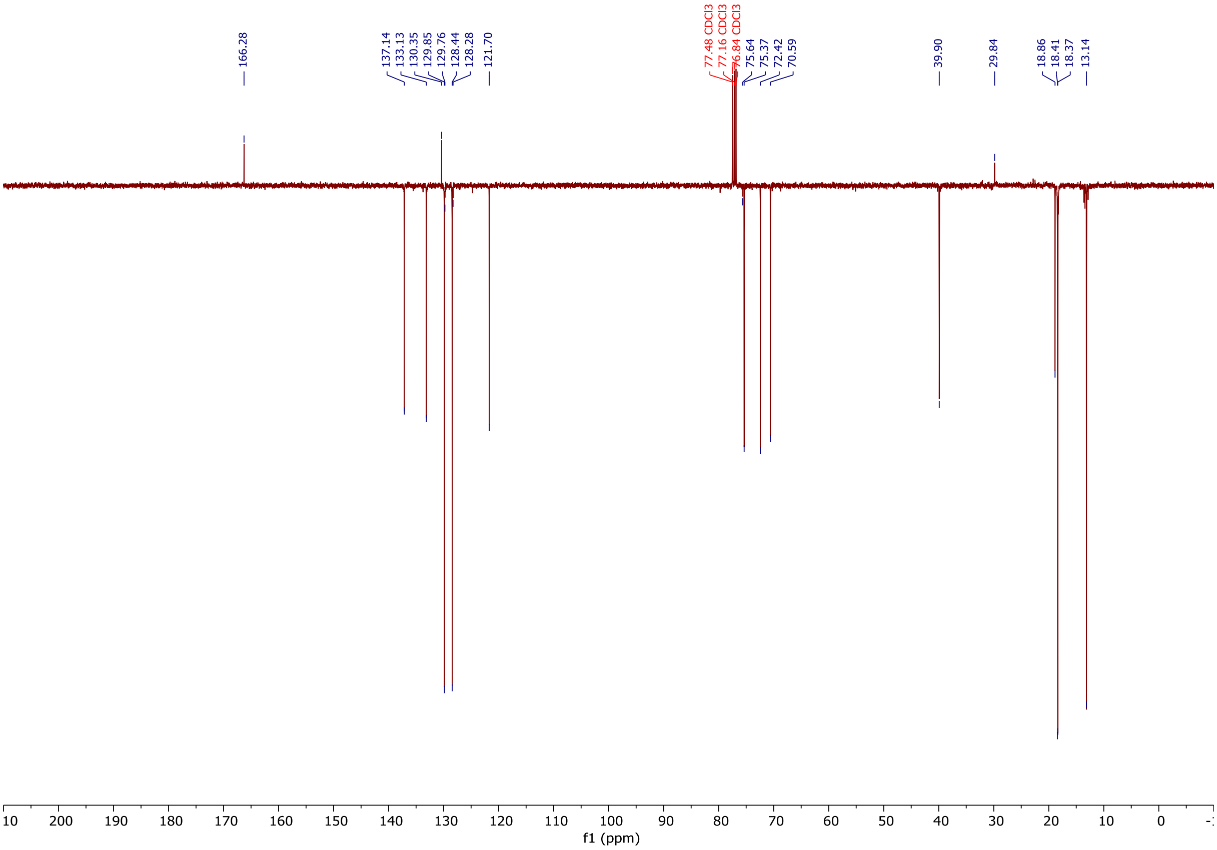

2-O-benzyl-4,6-O-benzylidene-3-O-naphthyl-β-D-mannose-(1->3)-2-O-benzoyl-4-O-TIPS-l-rhamnose-cyclohexene (64)

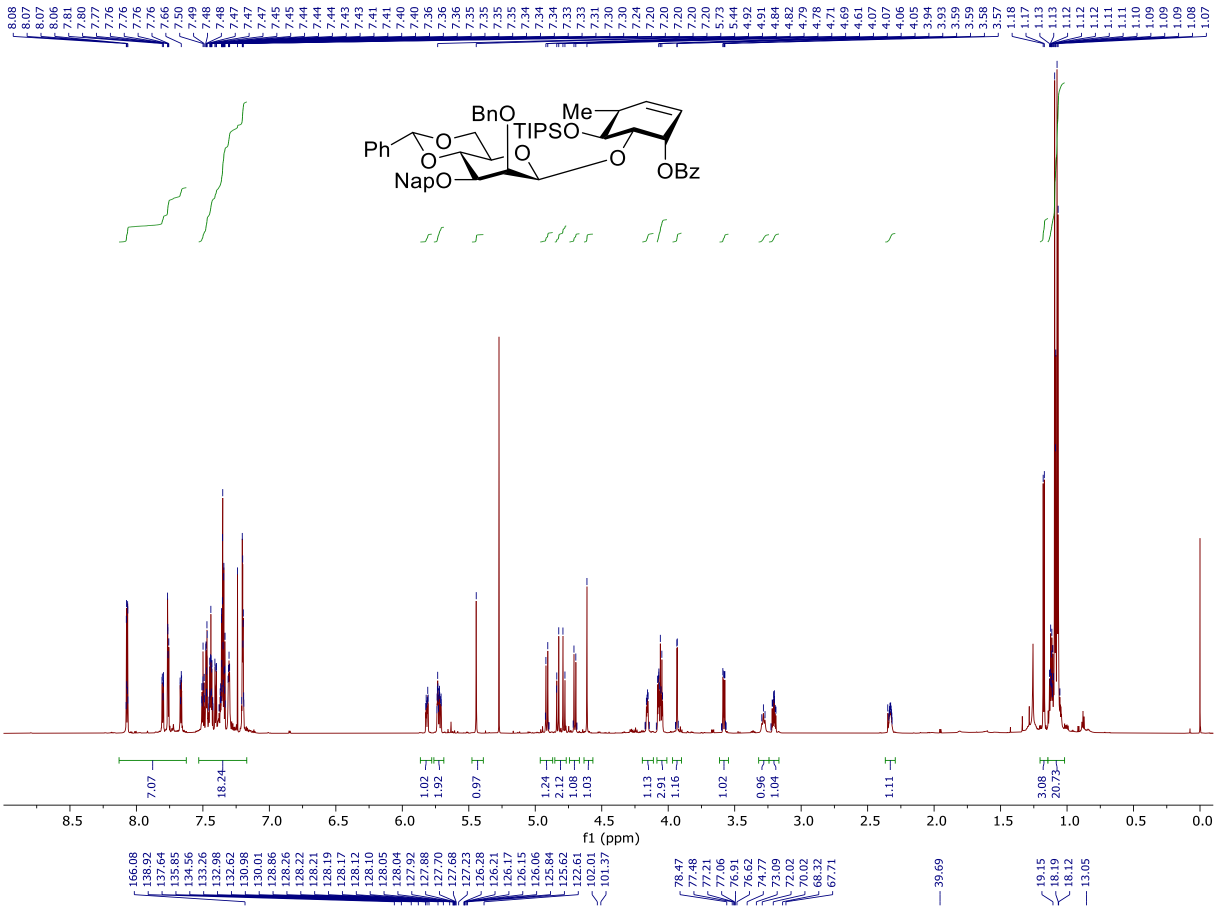

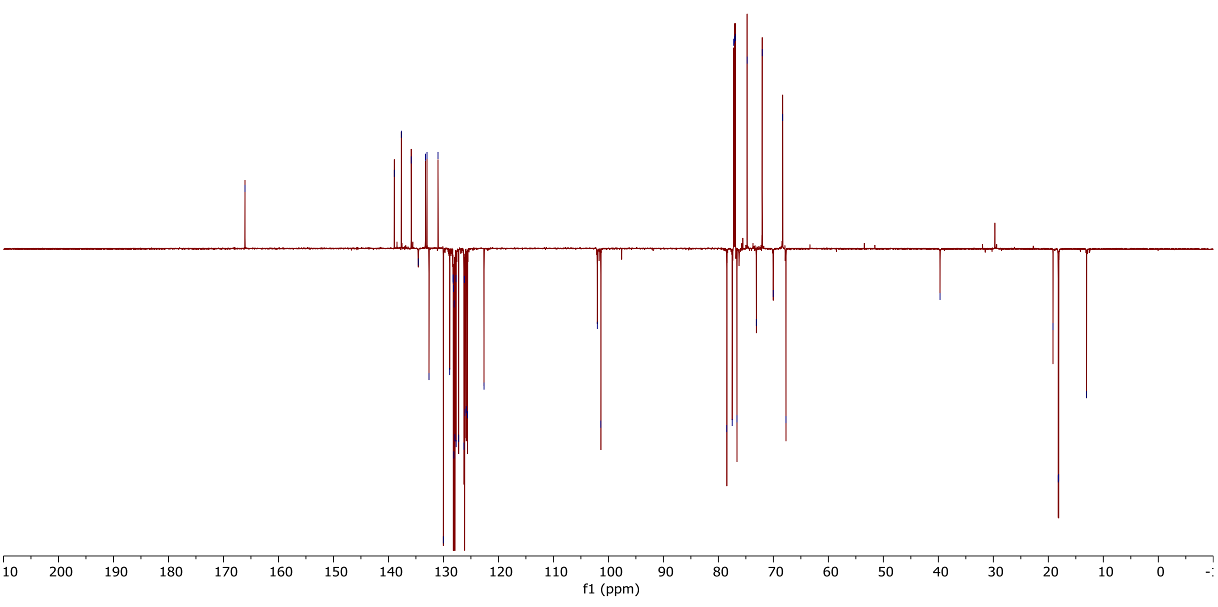

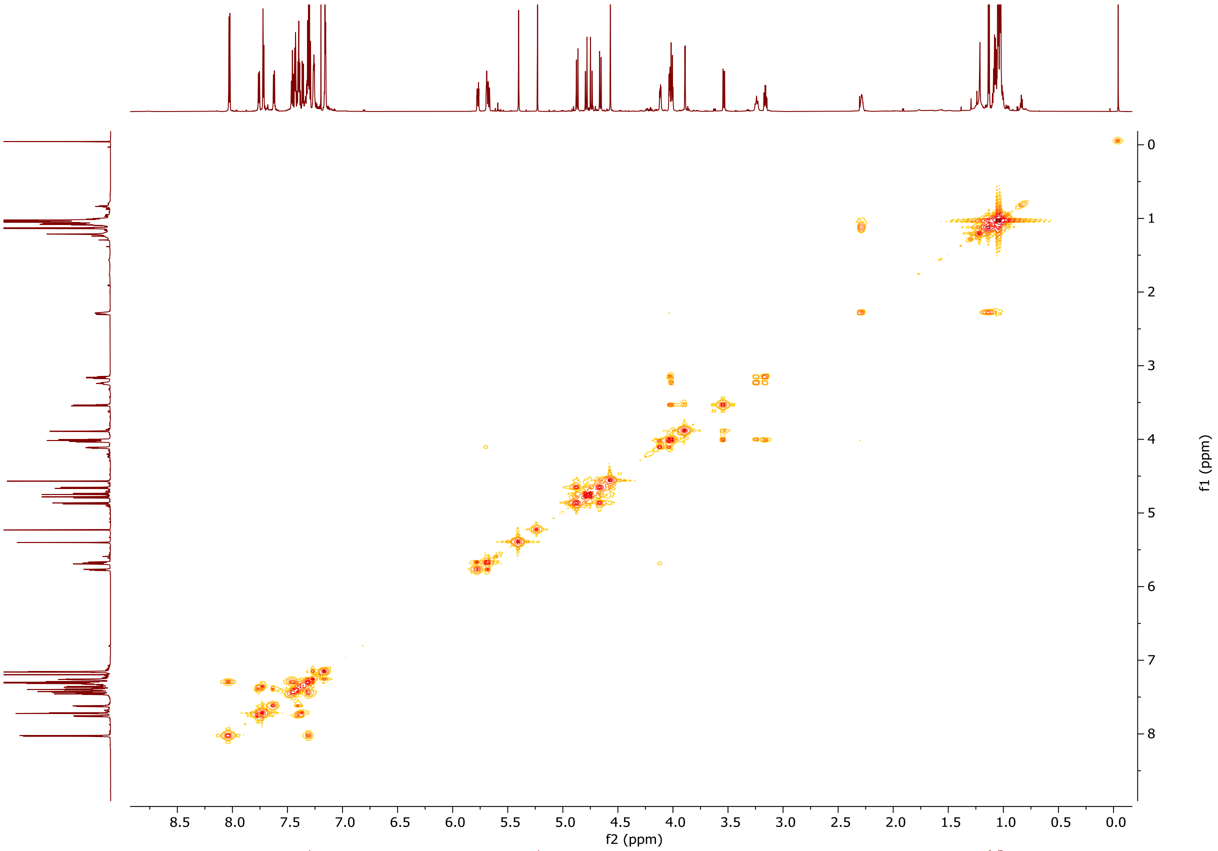
8.5 8.0 7.5 7.0 6.5 6.0 5.5 5.0 4.5 4.0 3.5 3.0 2.5 2.0 1.5 1.0 0.5 0.0
f2 (ppm)
0 1 2 3 4 5 6 7 8
f1 (ppm)

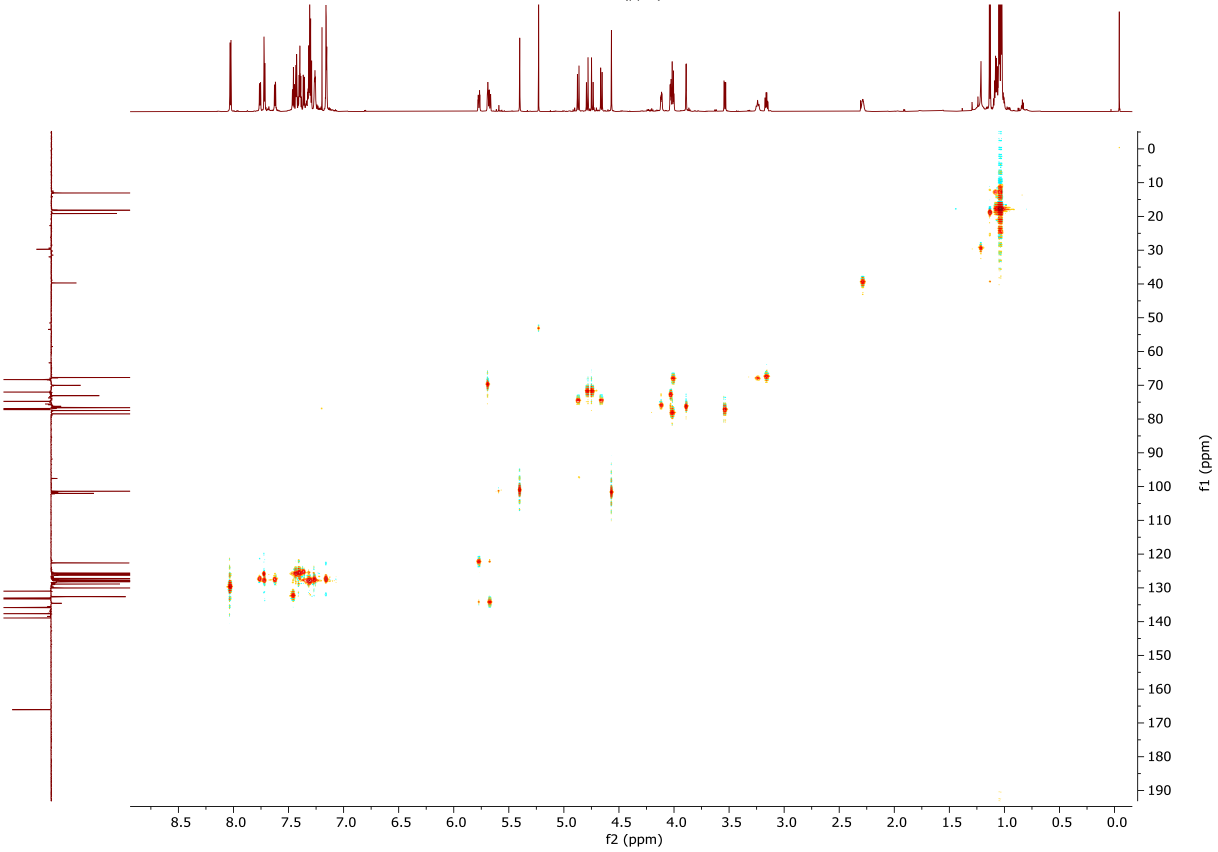
8.5 8.0 7.5 7.0 6.5 6.0 5.5 5.0 4.5 4.0 3.5 3.0 2.5 2.0 1.5 1.0 0.5 0.0
f2 (ppm)
0 10 20 30 40 50 60 70 80 90 100 110 120 130 140 150 160 170 180 190
f1 (ppm)

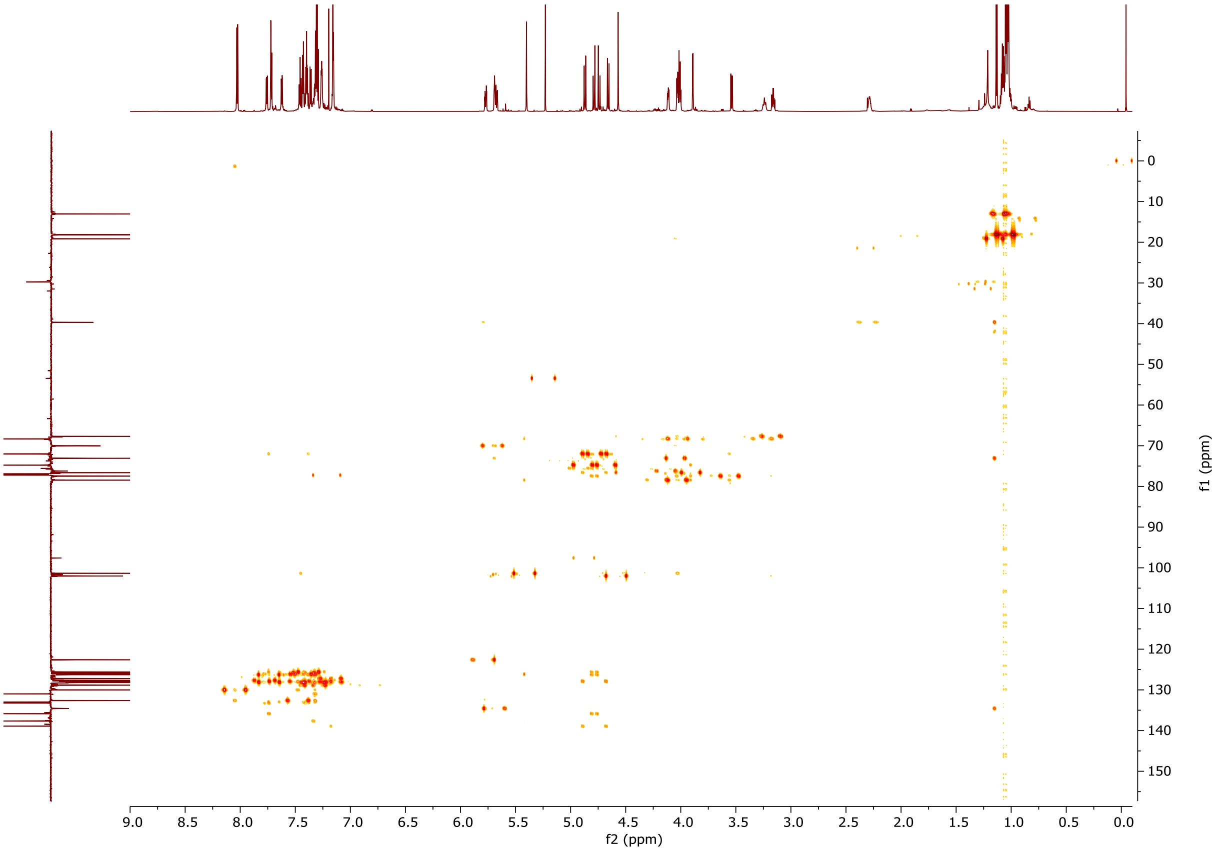

2-*O*-benzyl-3-*O*-naphthyl-β-D-mannose-(1->3)-2-*O*-benzoyl-4-O-TIPS-L-rhamnose-cyclohexene (65)

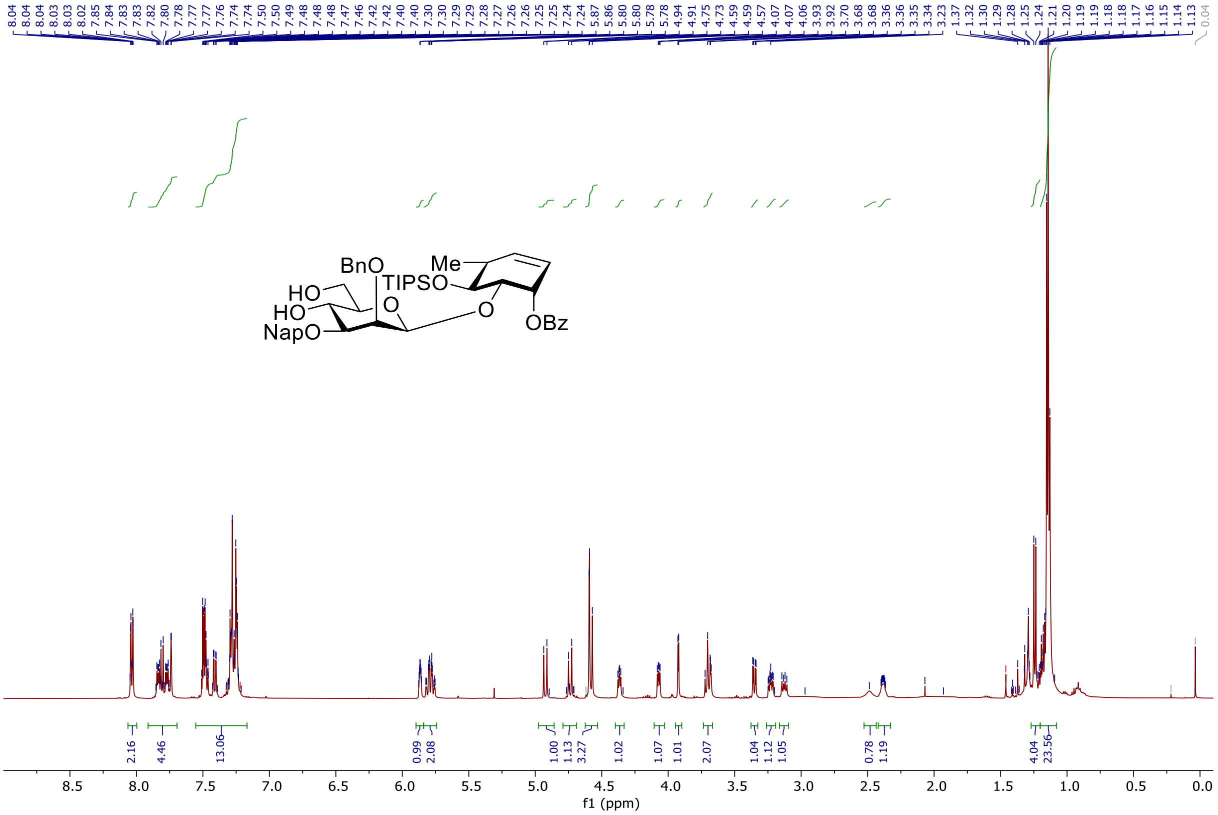

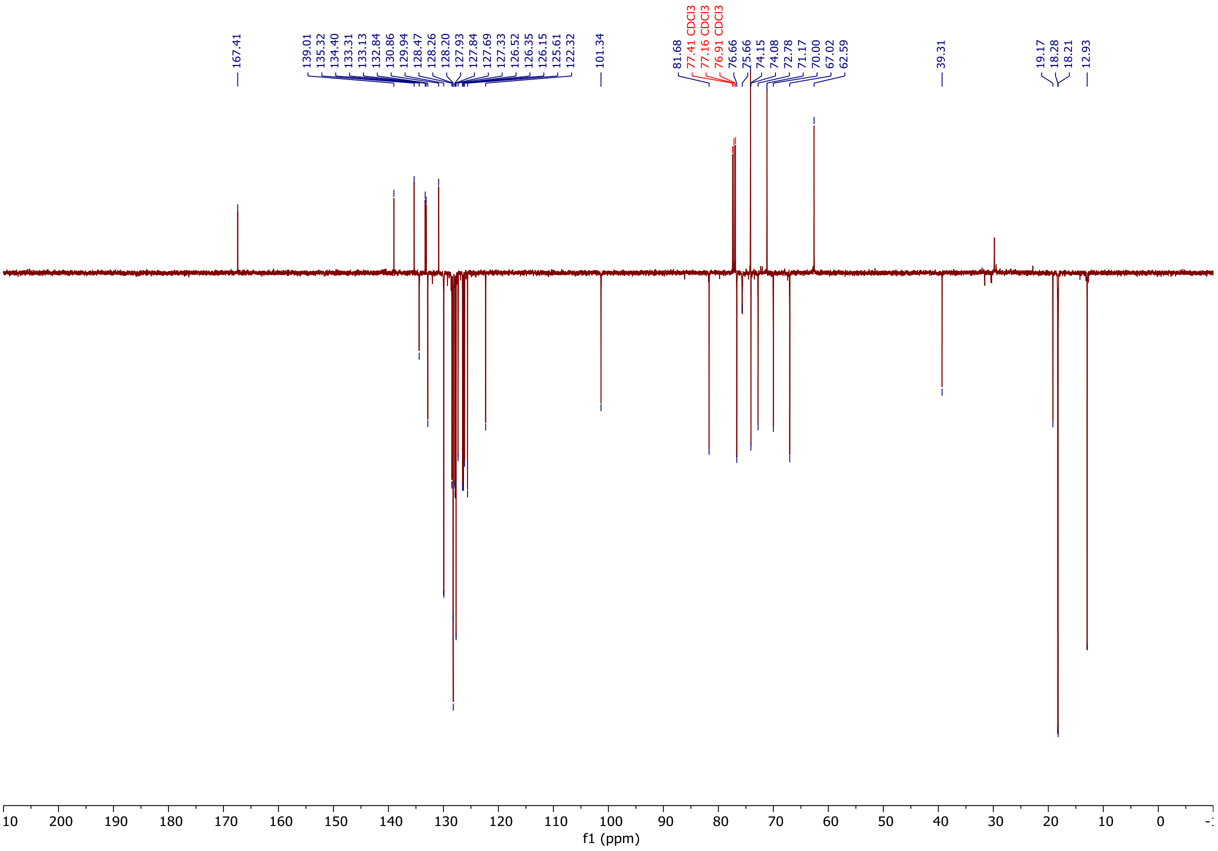
167.41
139.01
135.32
134.40
133.31
133.13
132.84
130.86
129.94
128.47
128.26
128.20
127.93
127.84
127.69
127.33
126.52
126.35
126.15
125.61
122.32
101.34
81.68
77.41 CDCl3
77.16 CDCl3
76.91 CDCl3
76.66
75.66
74.15
74.08
72.78
71.17
70.00
67.02
62.59
39.31
19.17
18.28
18.21
12.93
f1 (ppm)

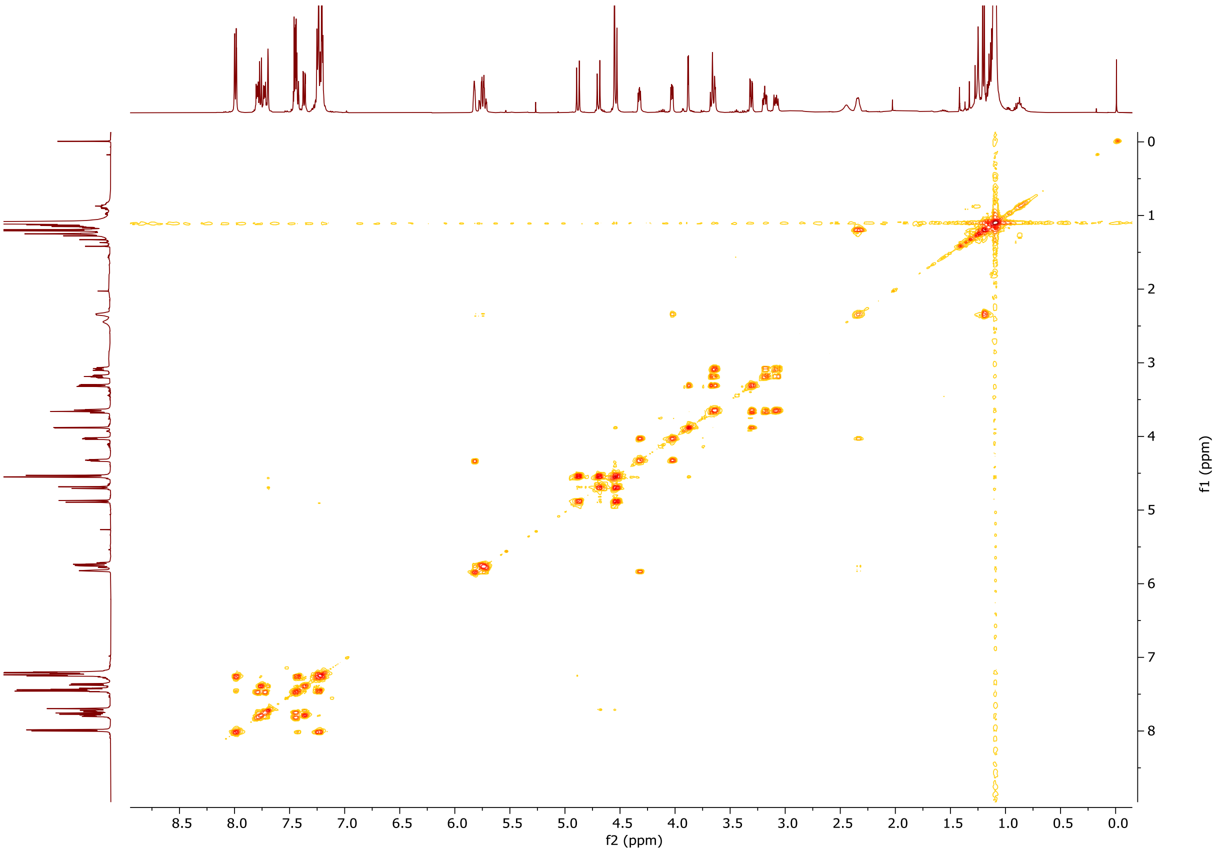
f2 (ppm)
f1 (ppm)

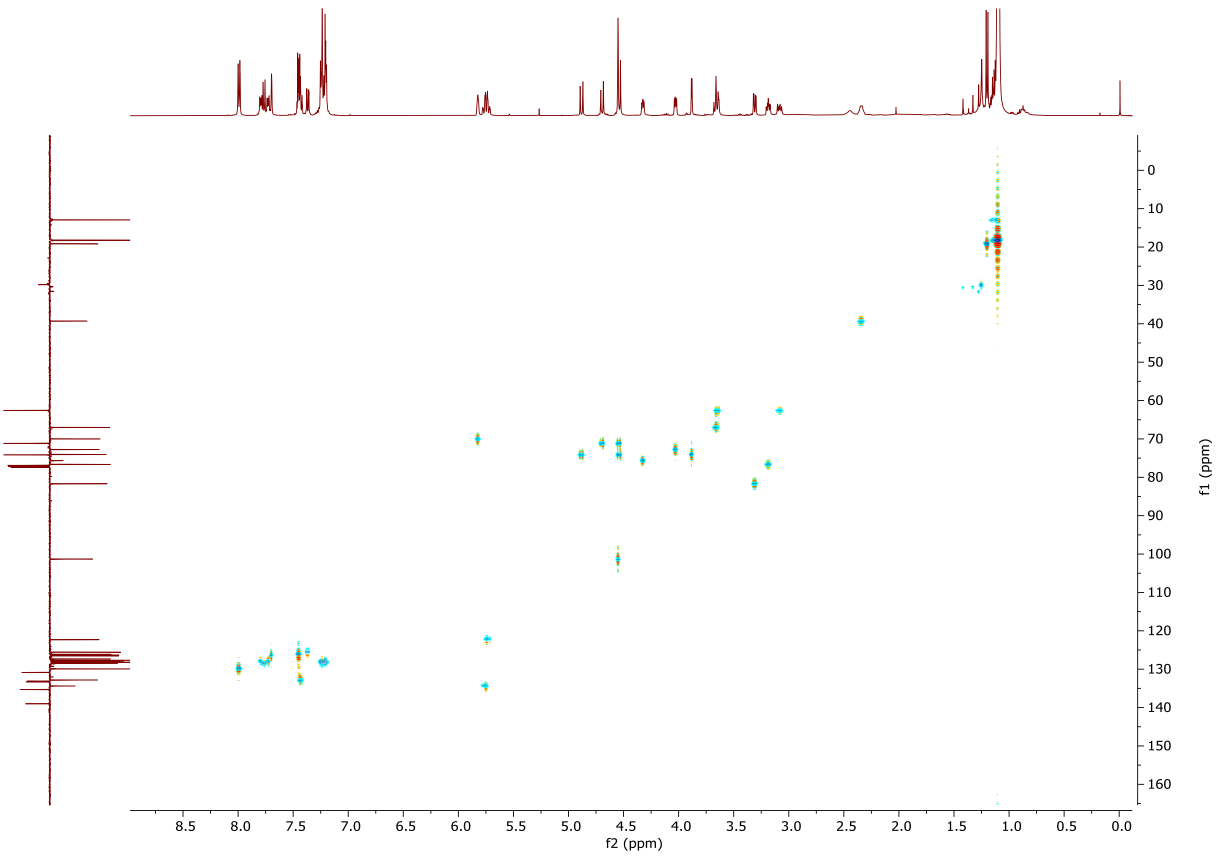

2-*O*-benzyl-3-*O*-naphthyl-β-D-mannose-(1->3)-4-*O*-TIPS-L-rhamnose-cyclohexene (66)

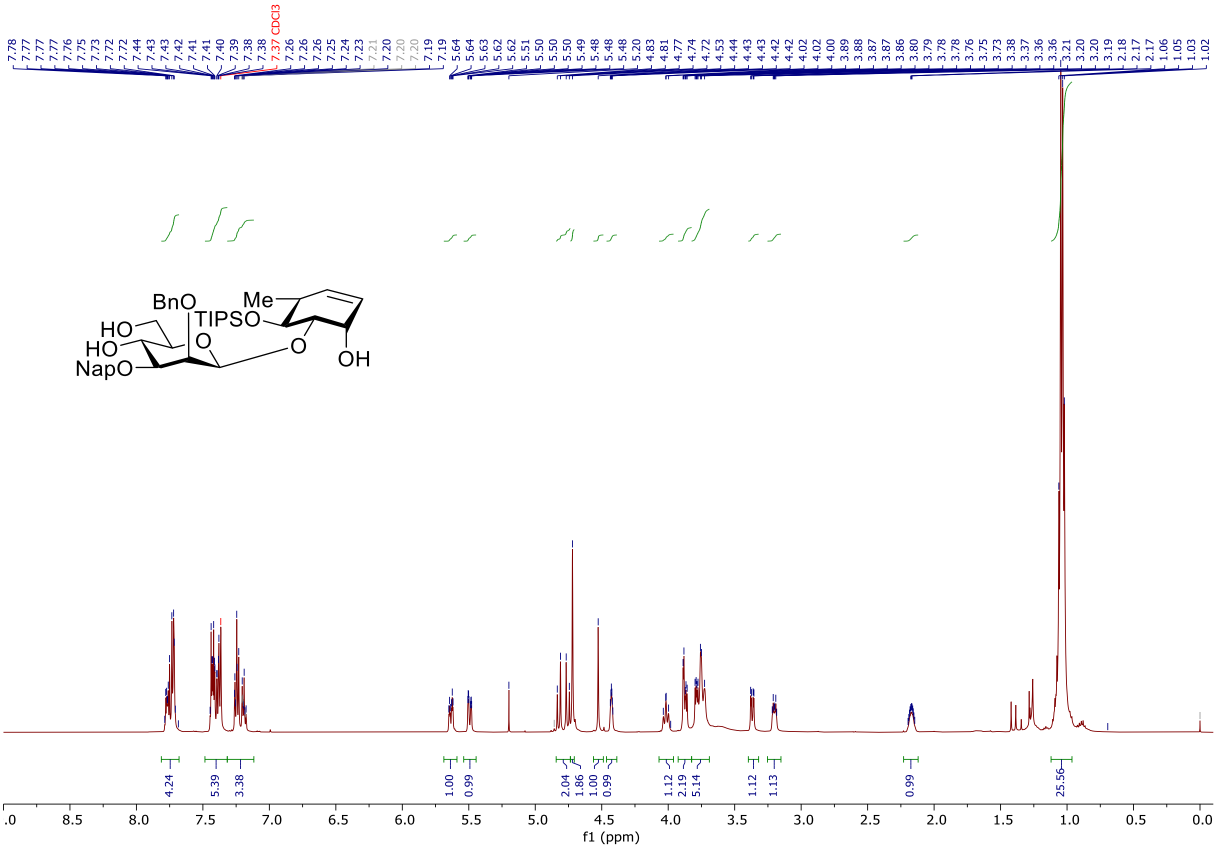

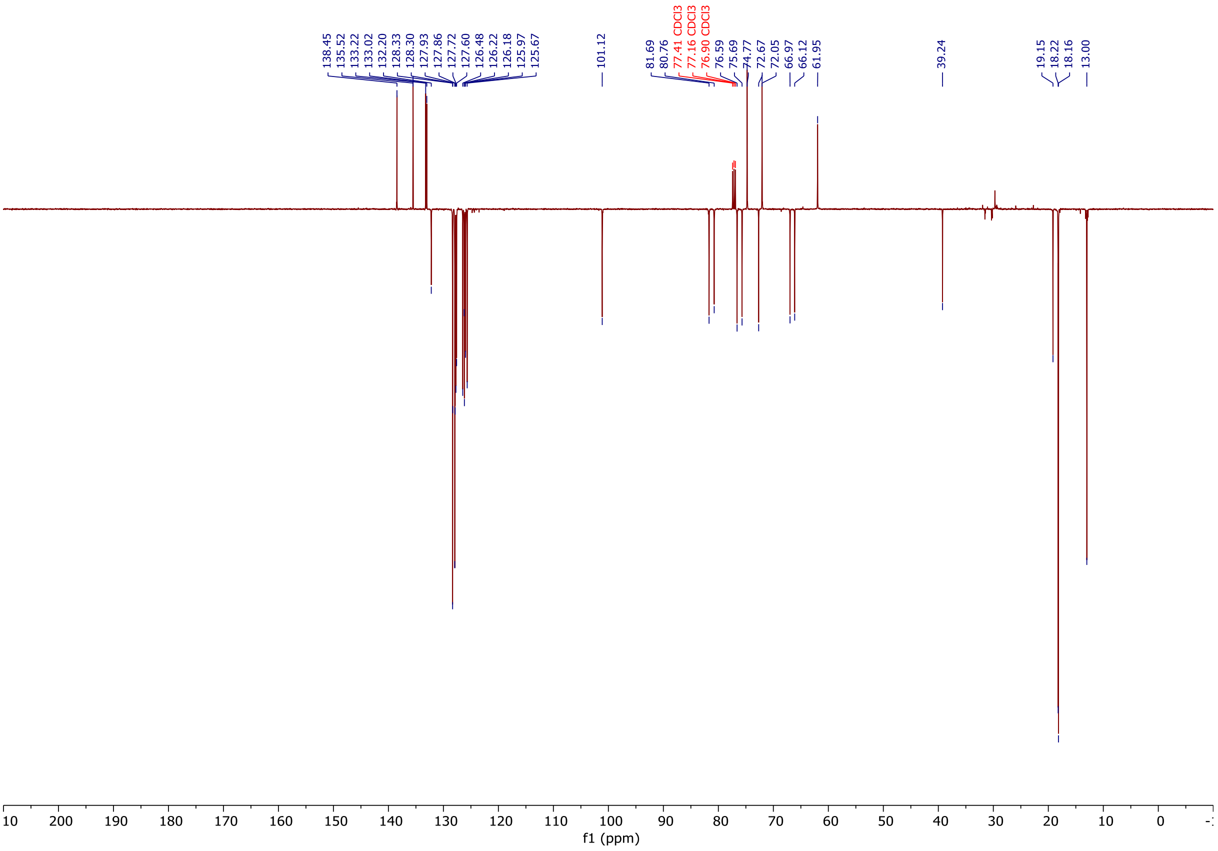
138.45
135.52
133.22
133.02
132.20
128.33
128.30
127.93
127.86
127.72
127.60
126.48
126.22
126.18
125.97
125.67
101.12
81.69
80.76
77.41 CDCl3
77.16 CDCl3
76.90 CDCl3
76.59
75.69
74.77
72.67
72.05
66.97
66.12
61.95
39.24
19.15
18.22
18.16
13.00
f1 (ppm)

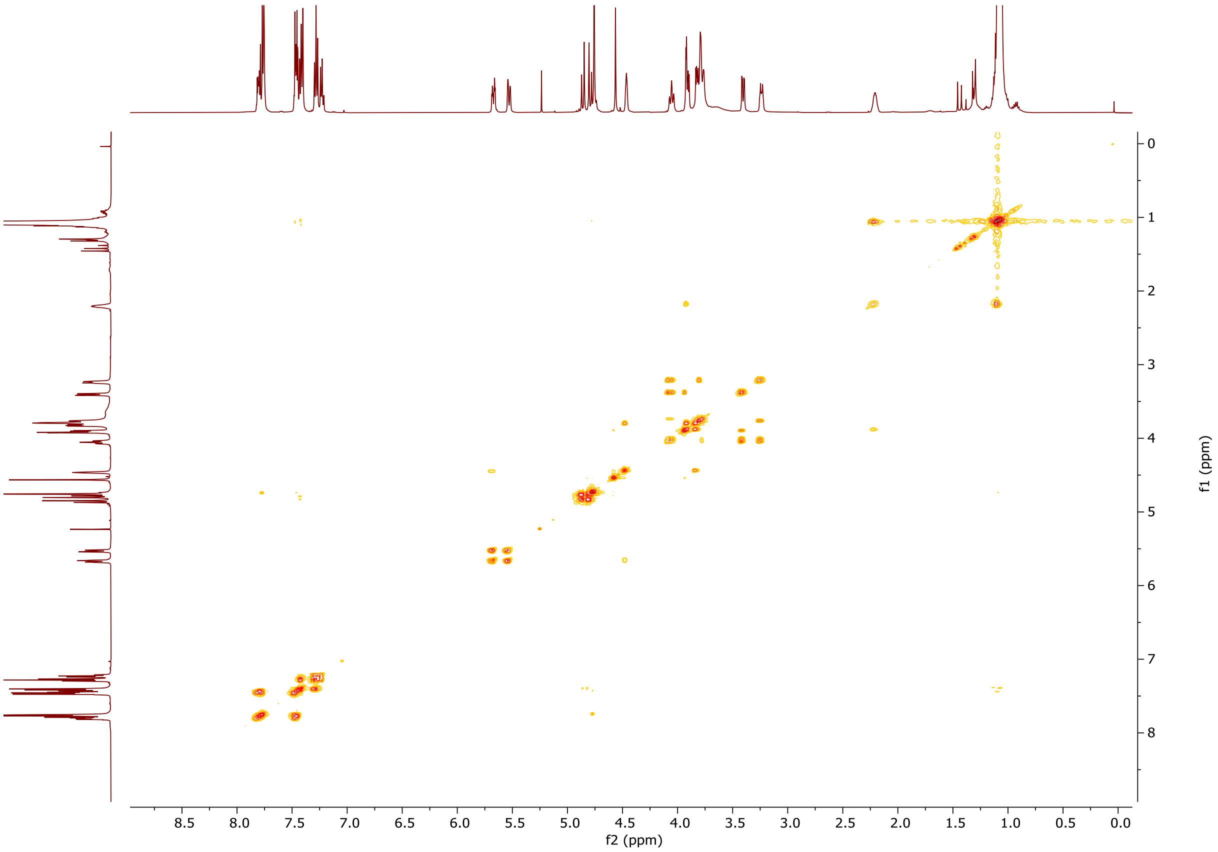
f2 (ppm)
f1 (ppm)

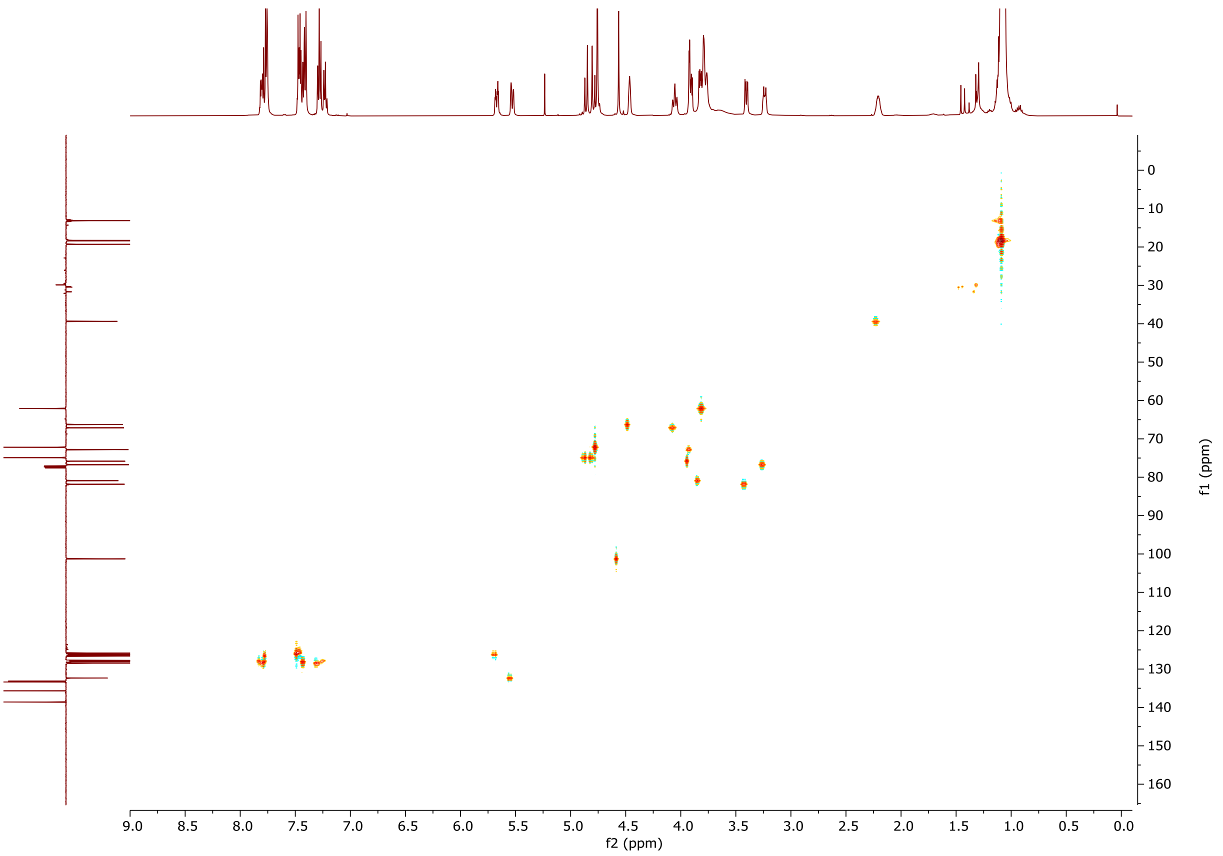

2,4,6-tri-*O*-benzyl-3-*O*-naphthyl-β-D-mannose-(1->3)-2-*O*-benzyl-4-*O*-TIPS-L-rhamnose-cyclohexene (67)

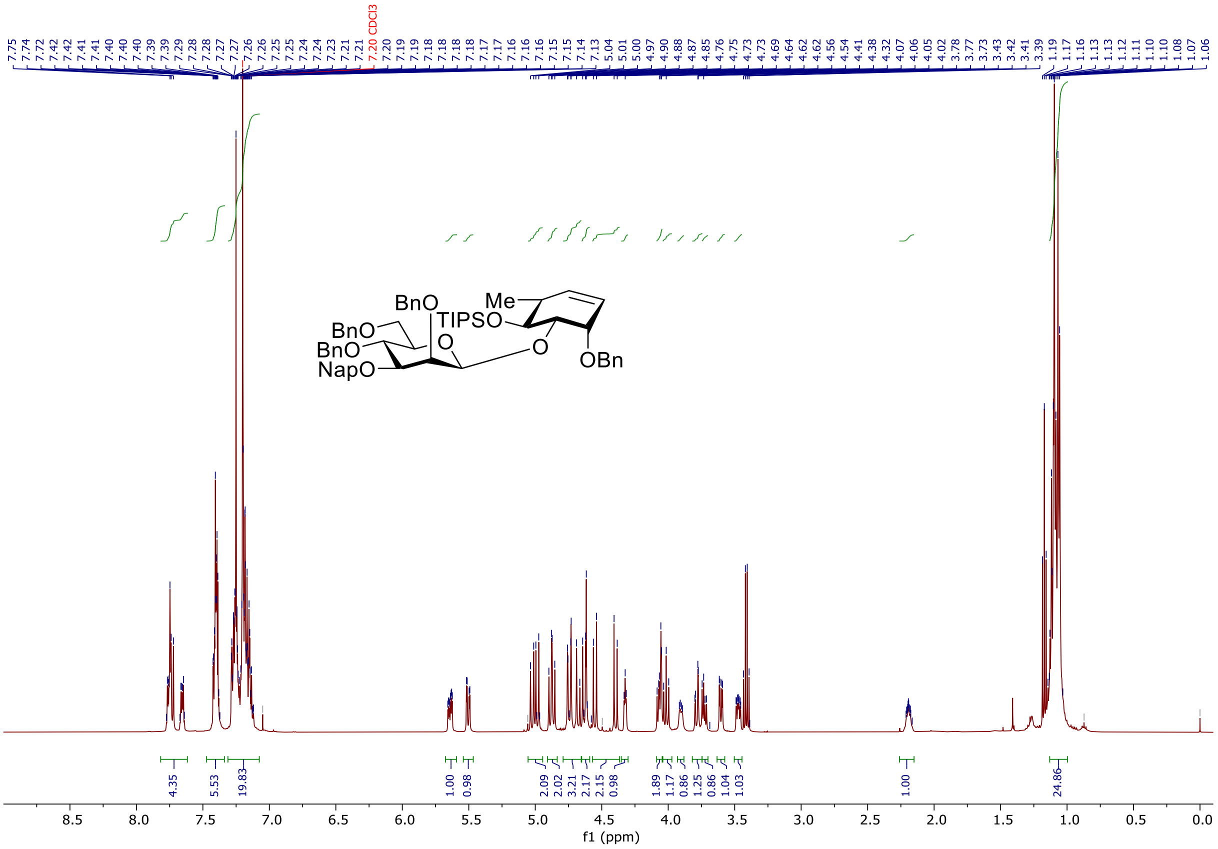

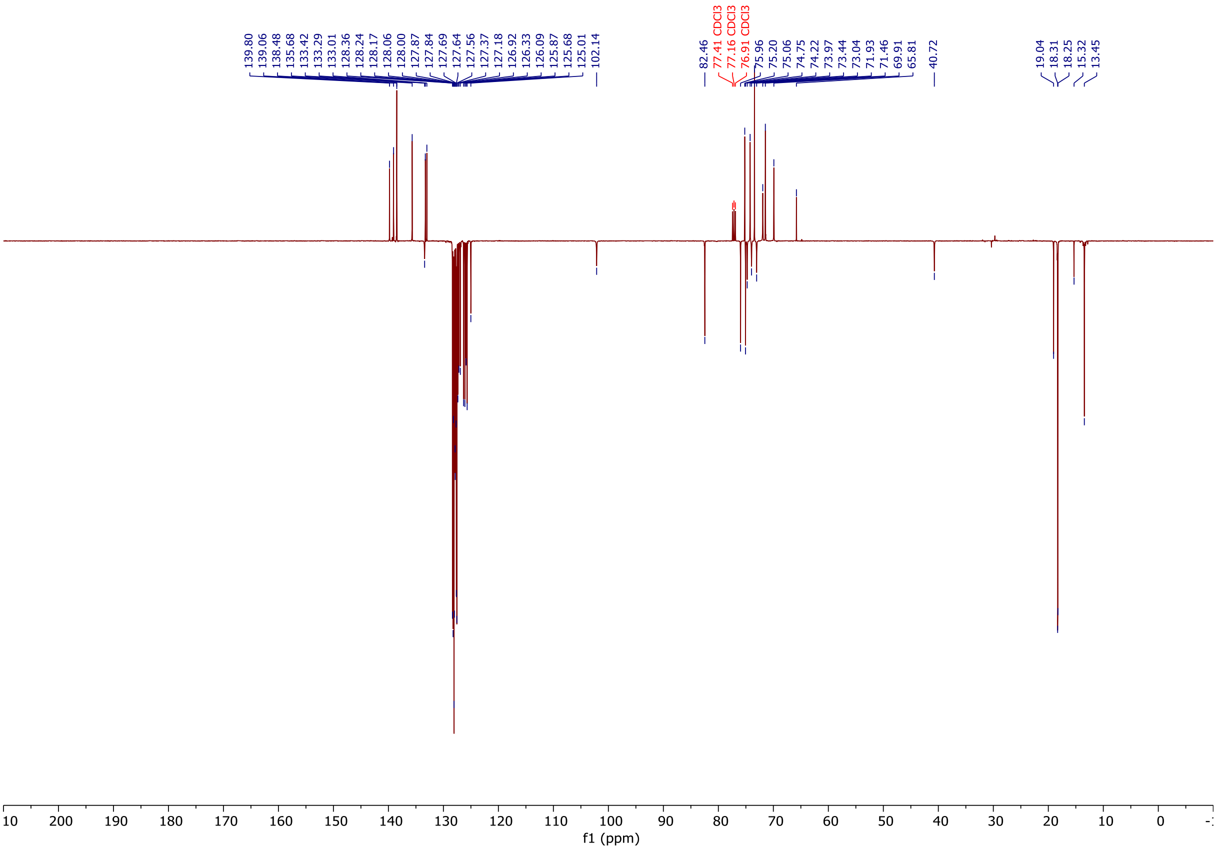
139.80
139.06
138.48
135.68
133.42
133.29
133.01
128.36
128.24
128.17
128.06
128.00
127.87
127.84
127.69
127.64
127.56
127.37
127.18
126.92
126.33
126.09
125.87
125.68
125.01
102.14
82.46
77.41 CDCl3
77.16 CDCl3
76.91 CDCl3
75.96
75.20
75.06
74.75
74.22
73.97
73.44
73.04
71.93
71.46
69.91
65.81
40.72
19.04
18.31
18.25
15.32
13.45
f1 (ppm)

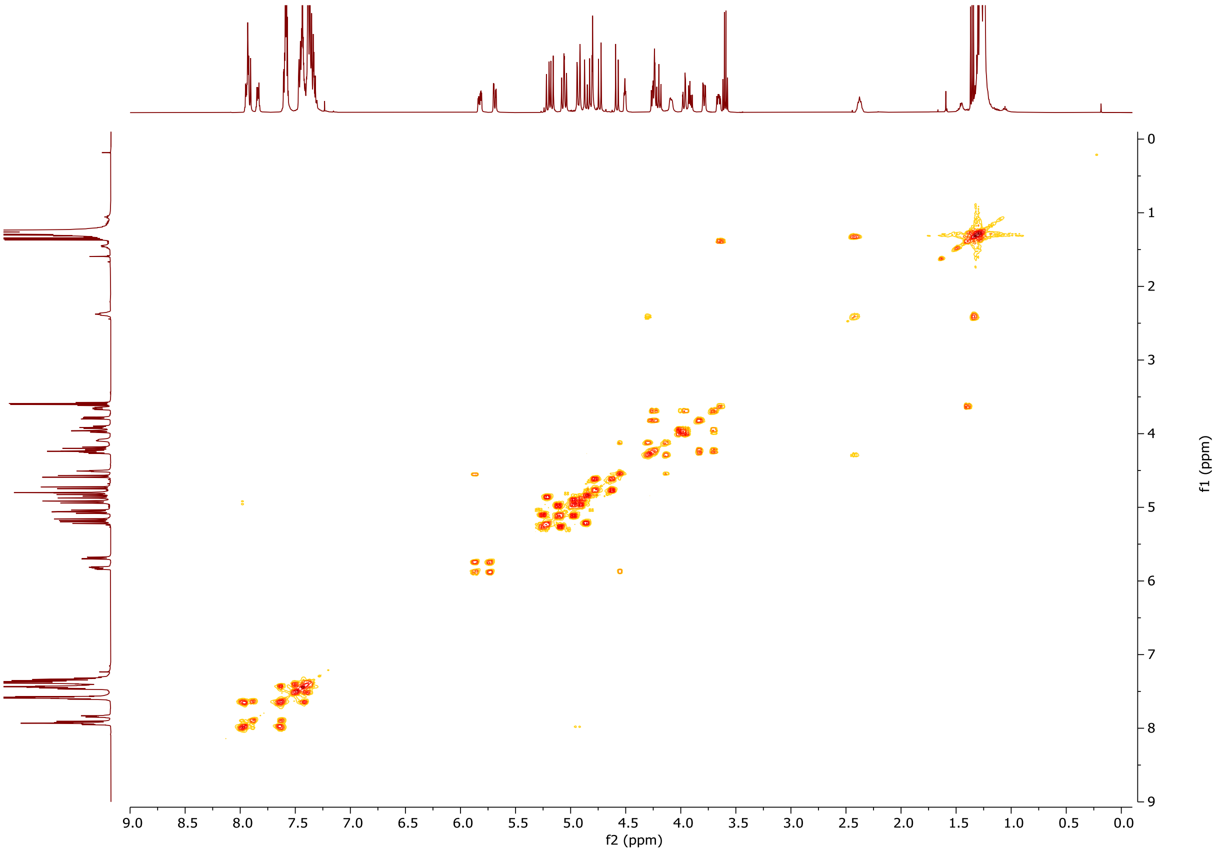
f2 (ppm)
f1 (ppm)

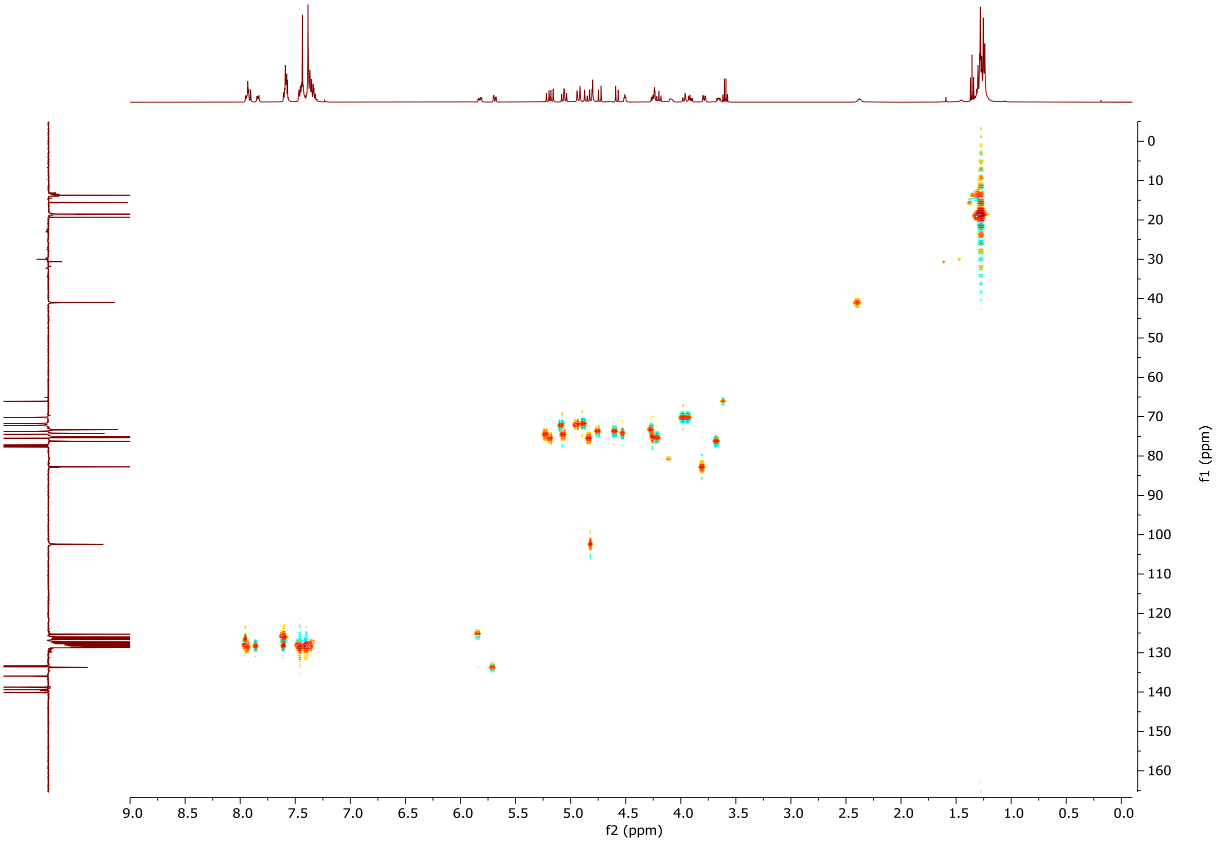

2,4,6-tri-*O*-benzyl-β-D-mannose-(1->3)-2-*O*-benzyl-4-*O*-TIPS-L-rhamnose-cyclohexene (68)

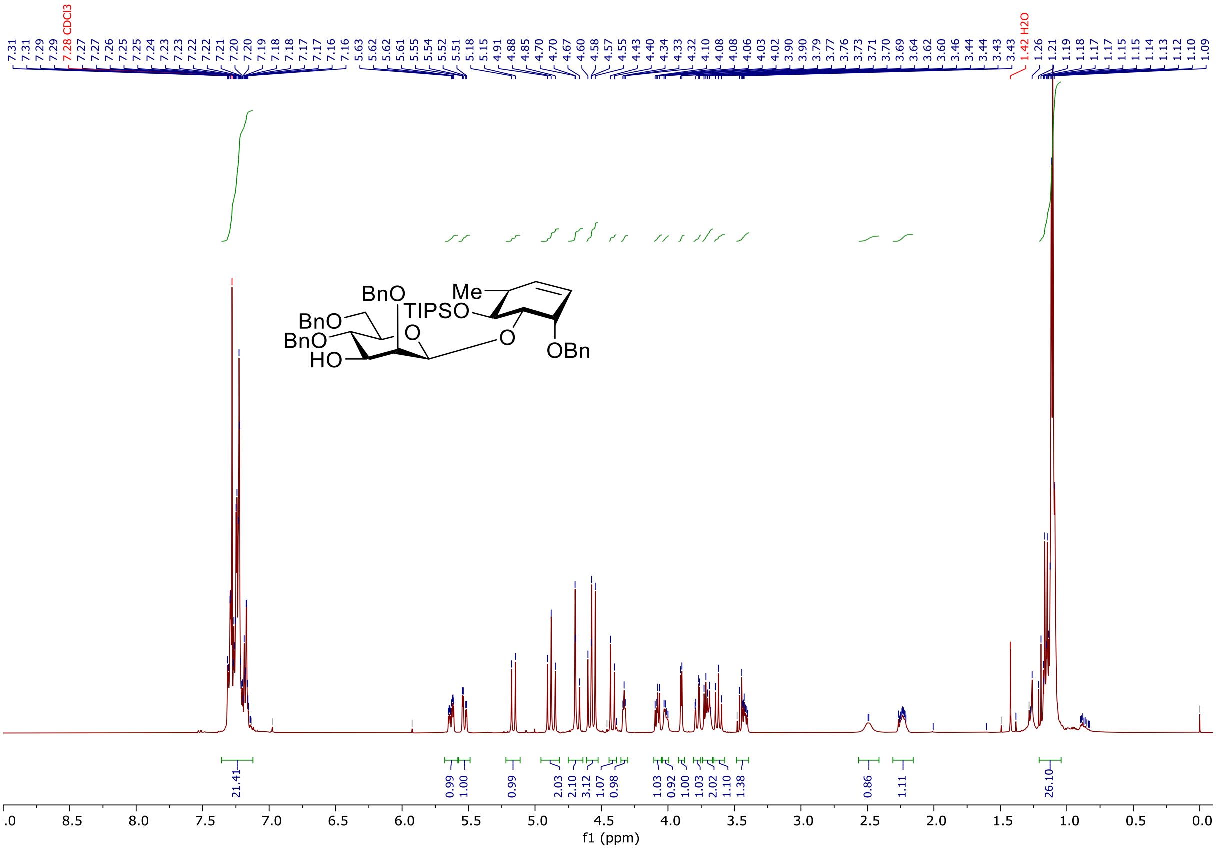

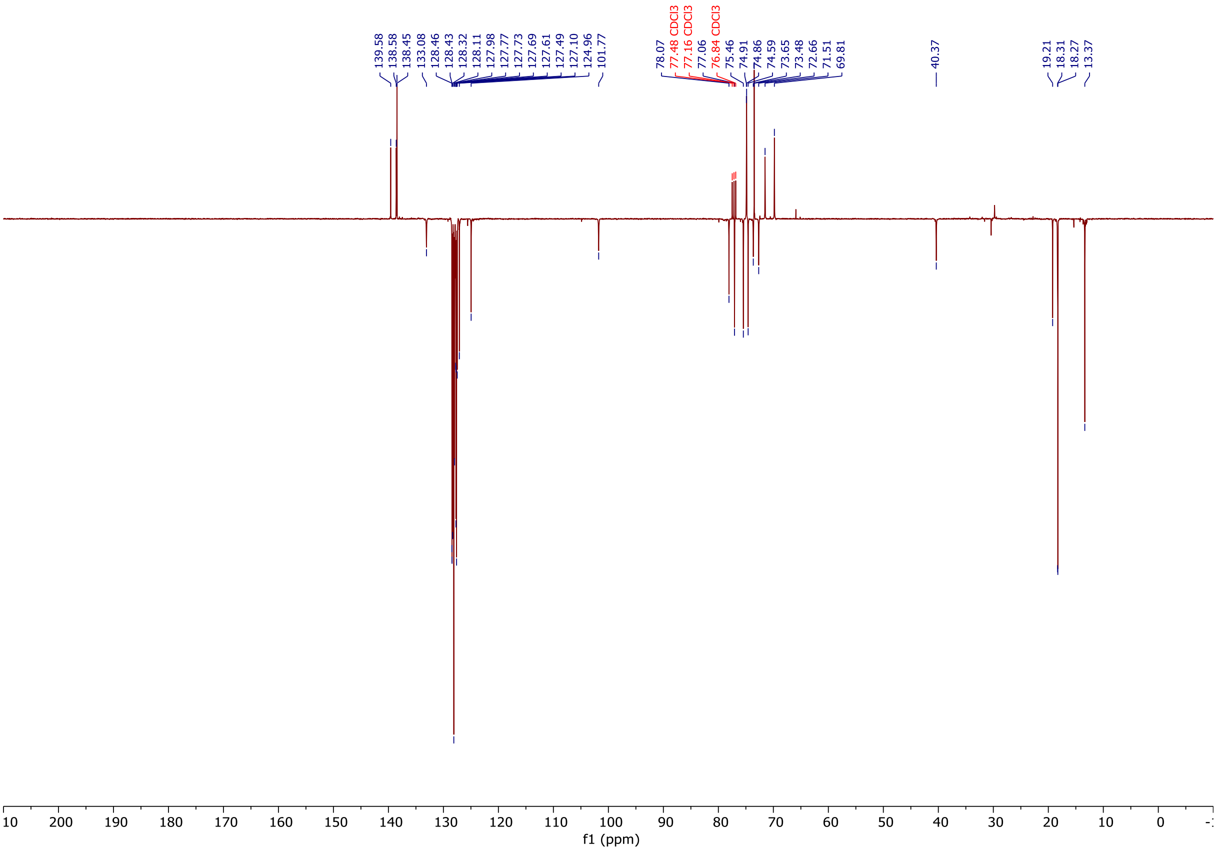
139.58
138.58
138.45
133.08
128.46
128.43
128.32
128.11
127.98
127.77
127.73
127.69
127.61
127.49
127.10
124.96
101.77
78.07
77.48 CDCl3
77.16 CDCl3
77.06
76.84 CDCl3
75.46
74.91
74.86
74.59
73.65
73.48
72.66
71.51
69.81
40.37
19.21
18.31
18.27
13.37
f1 (ppm)

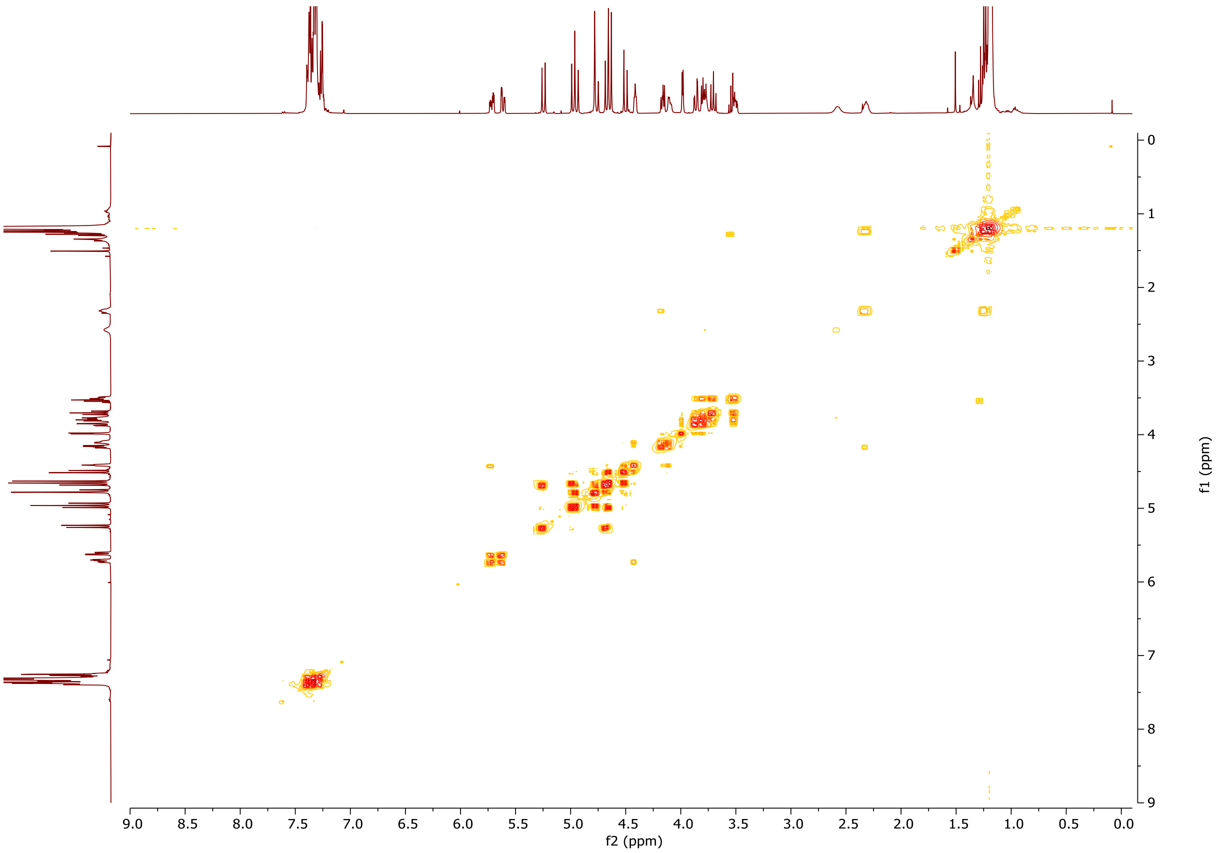
f2 (ppm)
f1 (ppm)

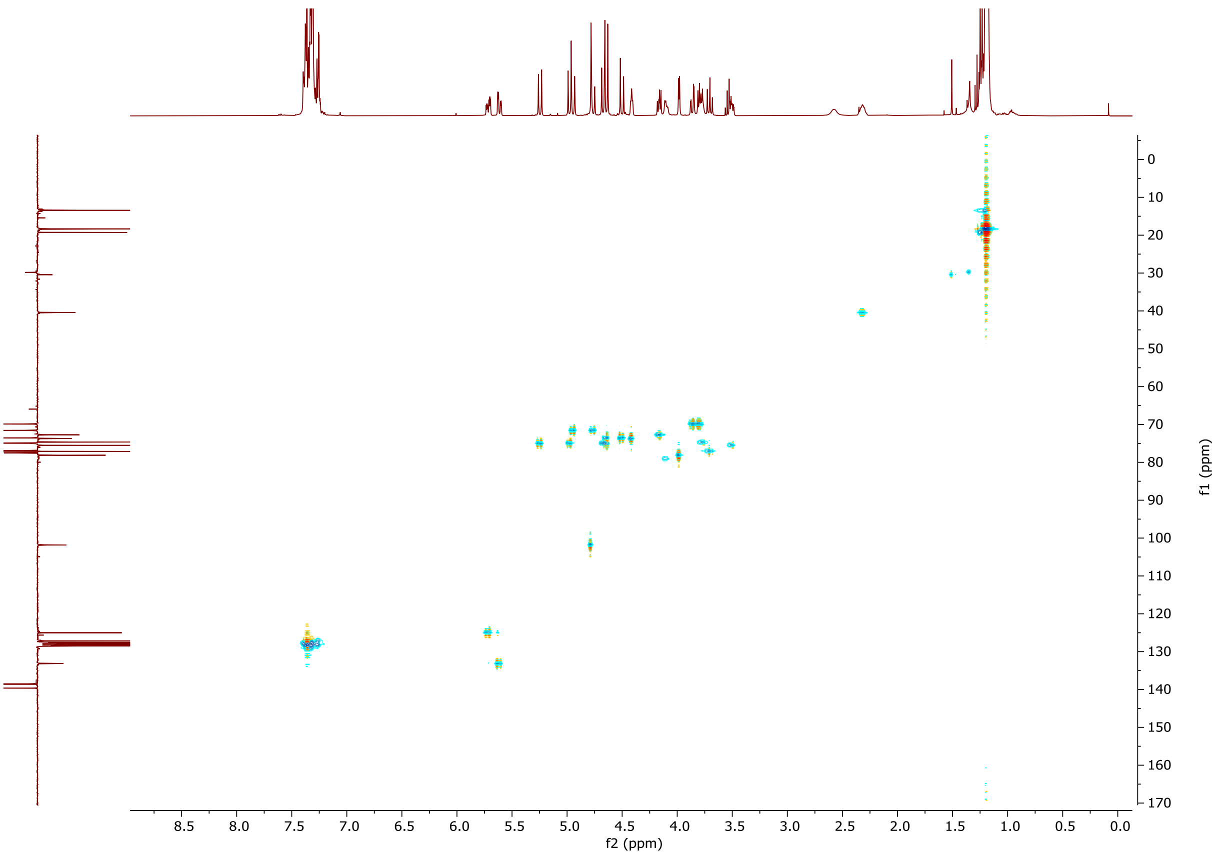

2-*O*-benzyl-4,6-*O*-benzylidene-3-*O*-(8-azido-octane)-β-D-mannose-(1->3)-2,4,6-tri-*O*-benzyl-β-D-mannose-(1->3)-2-*O*-benzyl-4-*O*-TIPS-L-rhamnose-cyclohexene (69)

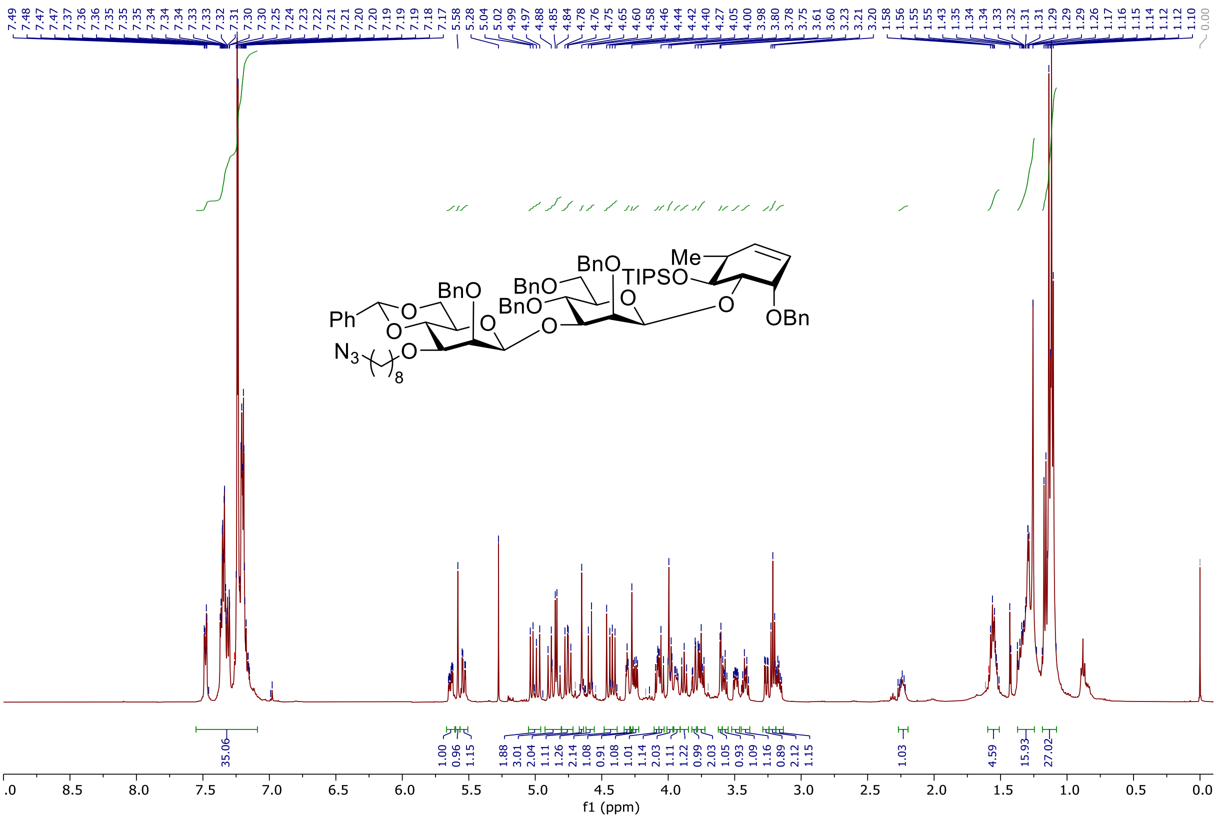

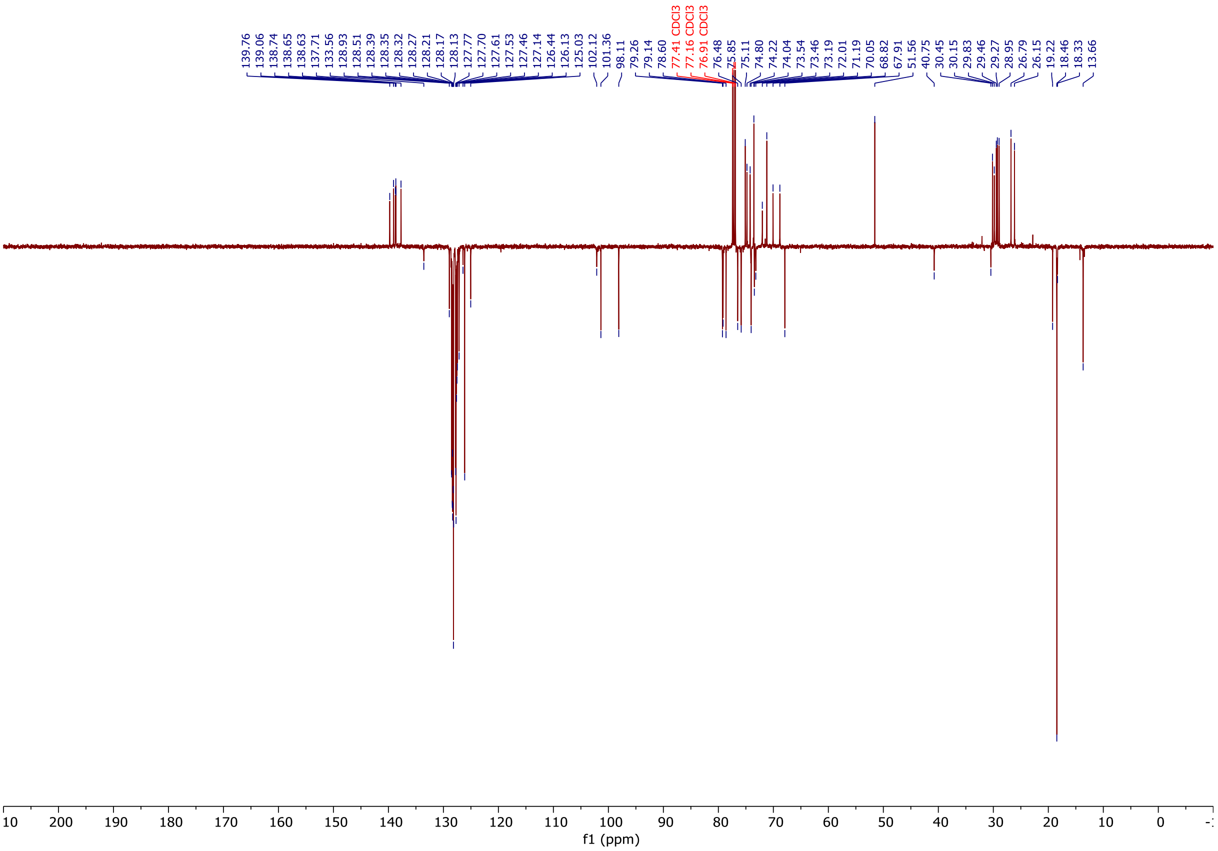
139.76
139.06
138.74
138.65
138.63
137.71
133.56
128.93
128.51
128.39
128.35
128.32
128.27
128.21
128.17
128.13
127.77
127.70
127.61
127.53
127.46
127.14
126.44
126.13
125.03
102.12
101.36
98.11
79.26
79.14
78.60
77.41 CDCl3
77.16 CDCl3
76.91 CDCl3
76.48
75.85
75.11
74.80
74.22
74.04
73.54
73.46
73.19
72.01
71.19
70.05
68.82
67.91
51.56
40.75
30.45
30.15
29.83
29.46
29.27
28.95
26.79
26.15
19.22
18.46
18.33
13.66
f1 (ppm)

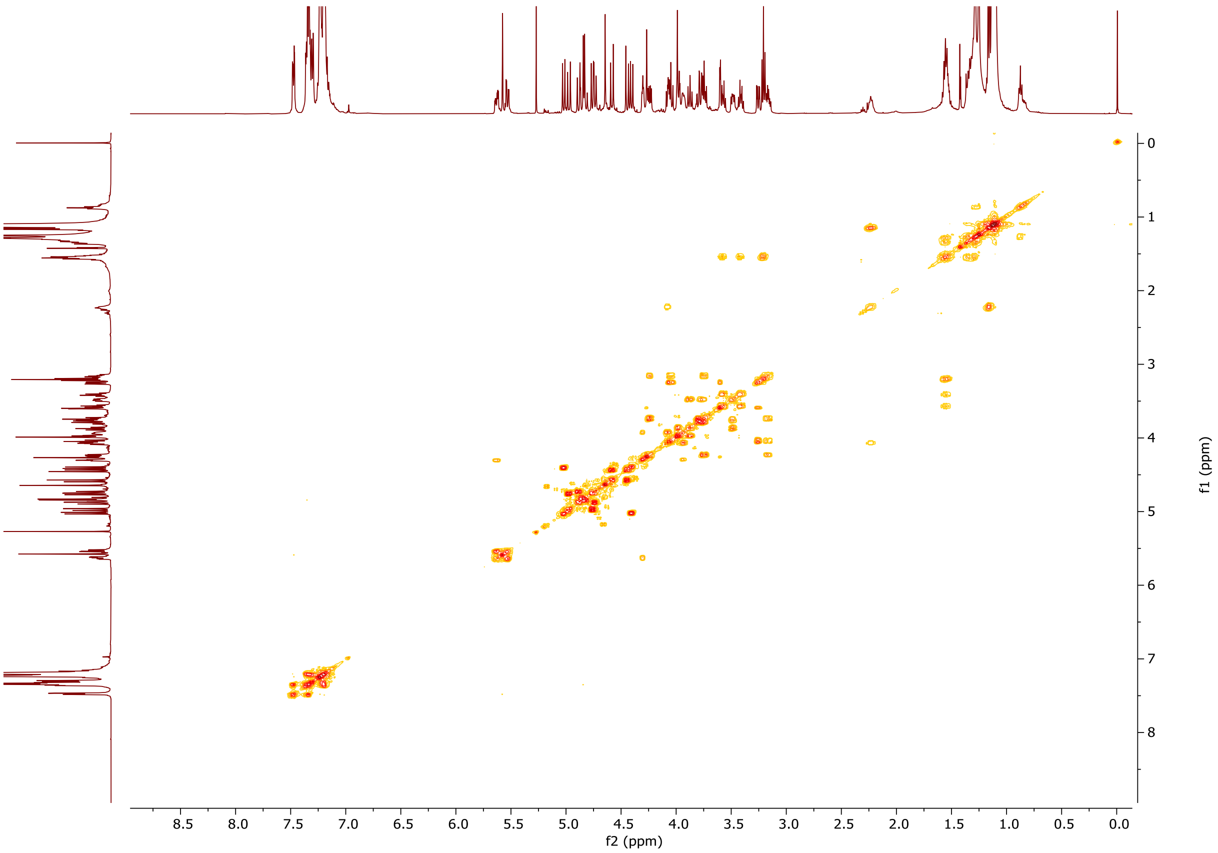
f2 (ppm)
f1 (ppm)

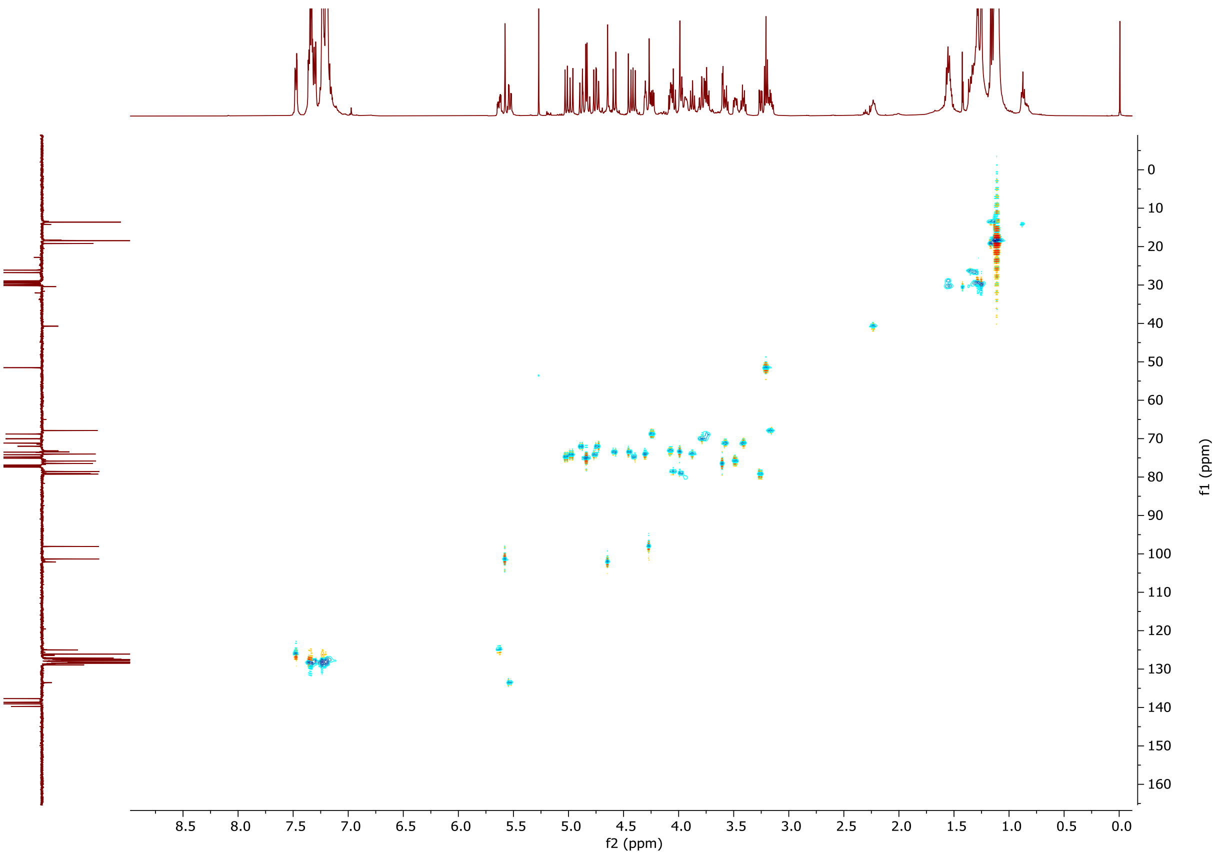

f1 (ppm)
f2 (ppm)

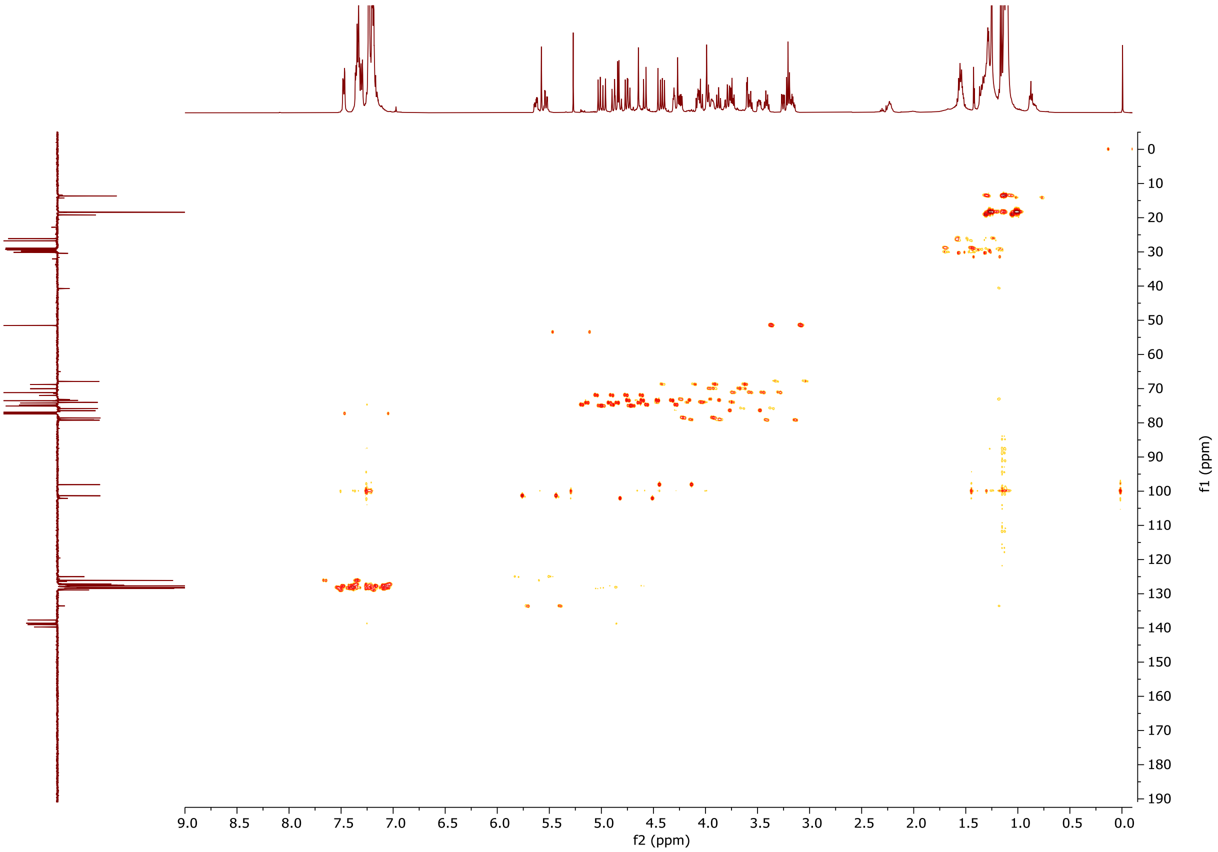

f1 (ppm)
f2 (ppm)

2-*O*-benzyl-4,6-*O*-benzylidene-3-*O*-(8-azido-octane)-β-D-mannose-(1->3)-2,4,6-tri-*O*-benzyl-β-D-mannose-(1->3)-2-*O*-benzyl-4-*O*-TIPS-α-L-rhamnose-cyclophellitol (70)

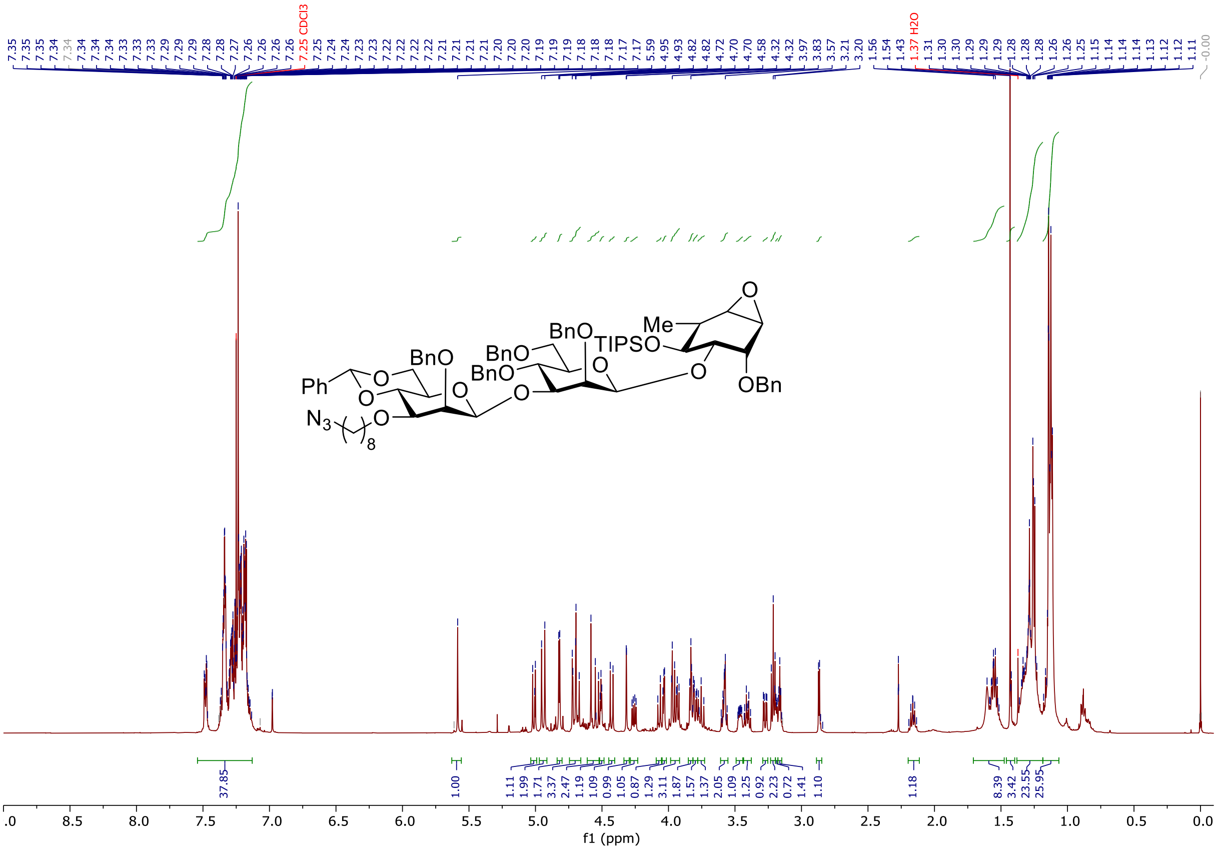

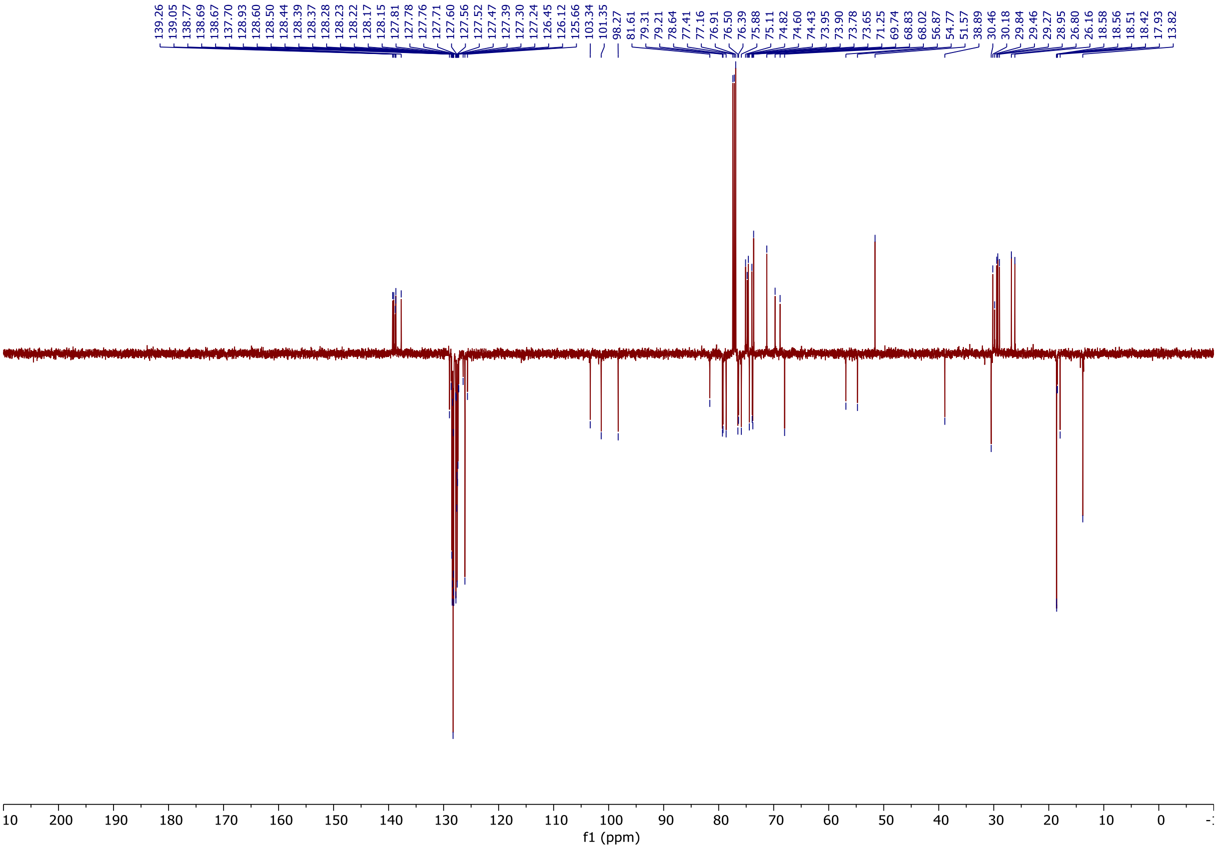

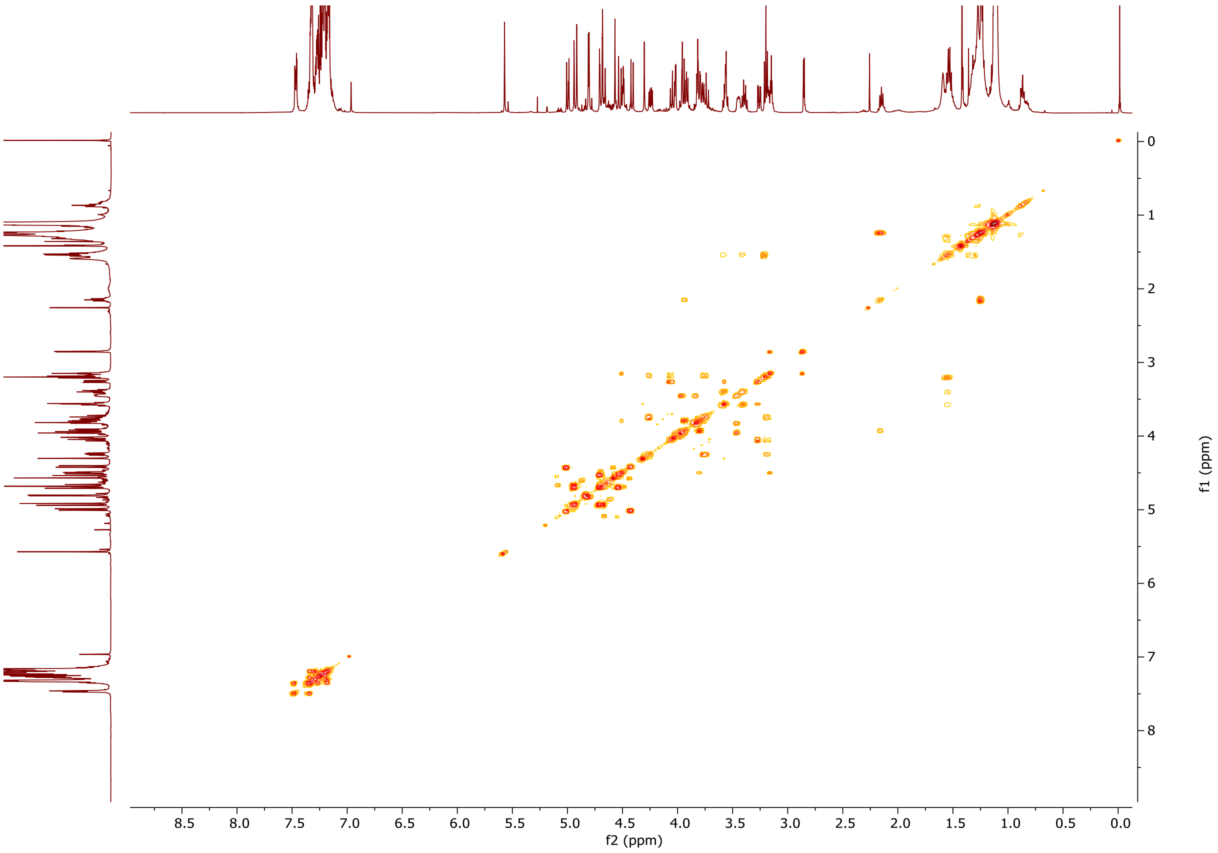

f2 (ppm)
f1 (ppm)

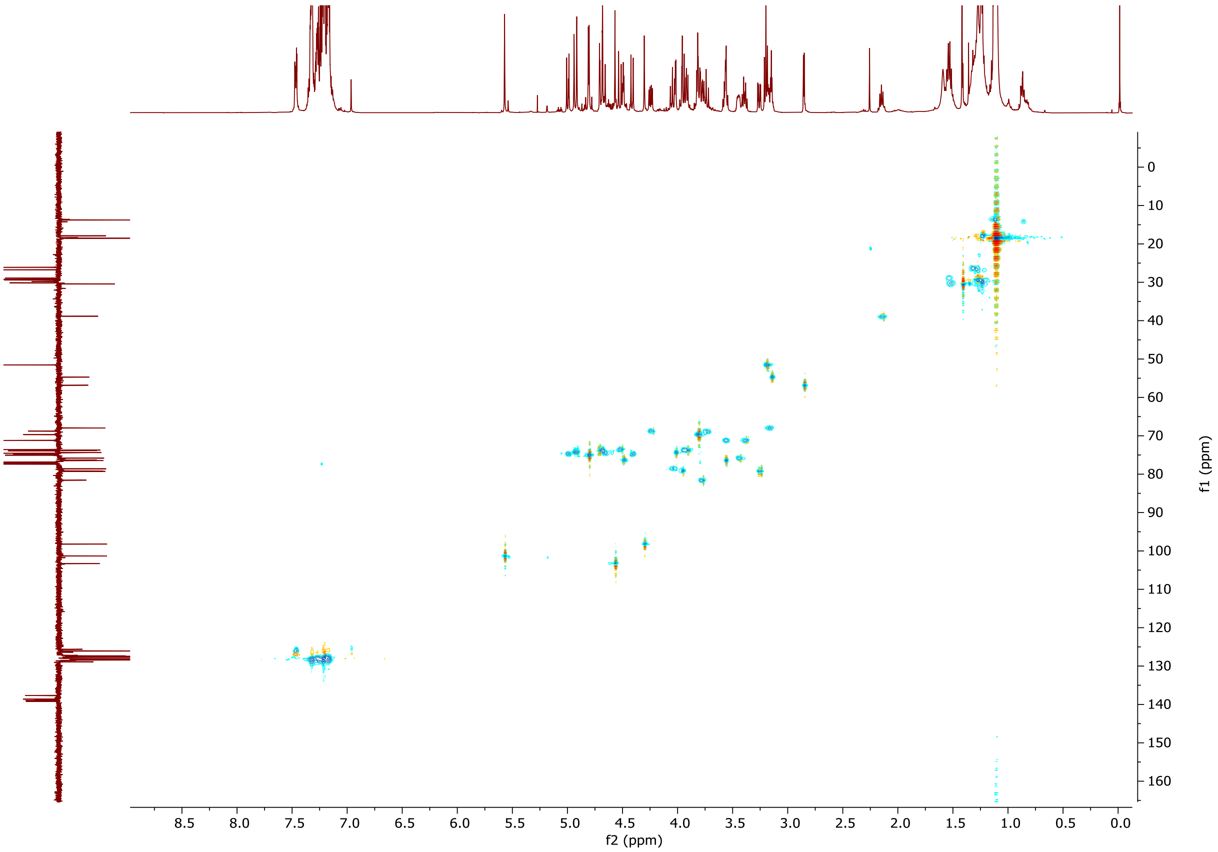

f2 (ppm)
f1 (ppm)

3-*O*-(8-amine-octane)-β-D-mannose-(1->3)-β-D-mannose-(1->3)-α-L-rhamnose-cyclophellitol (71)

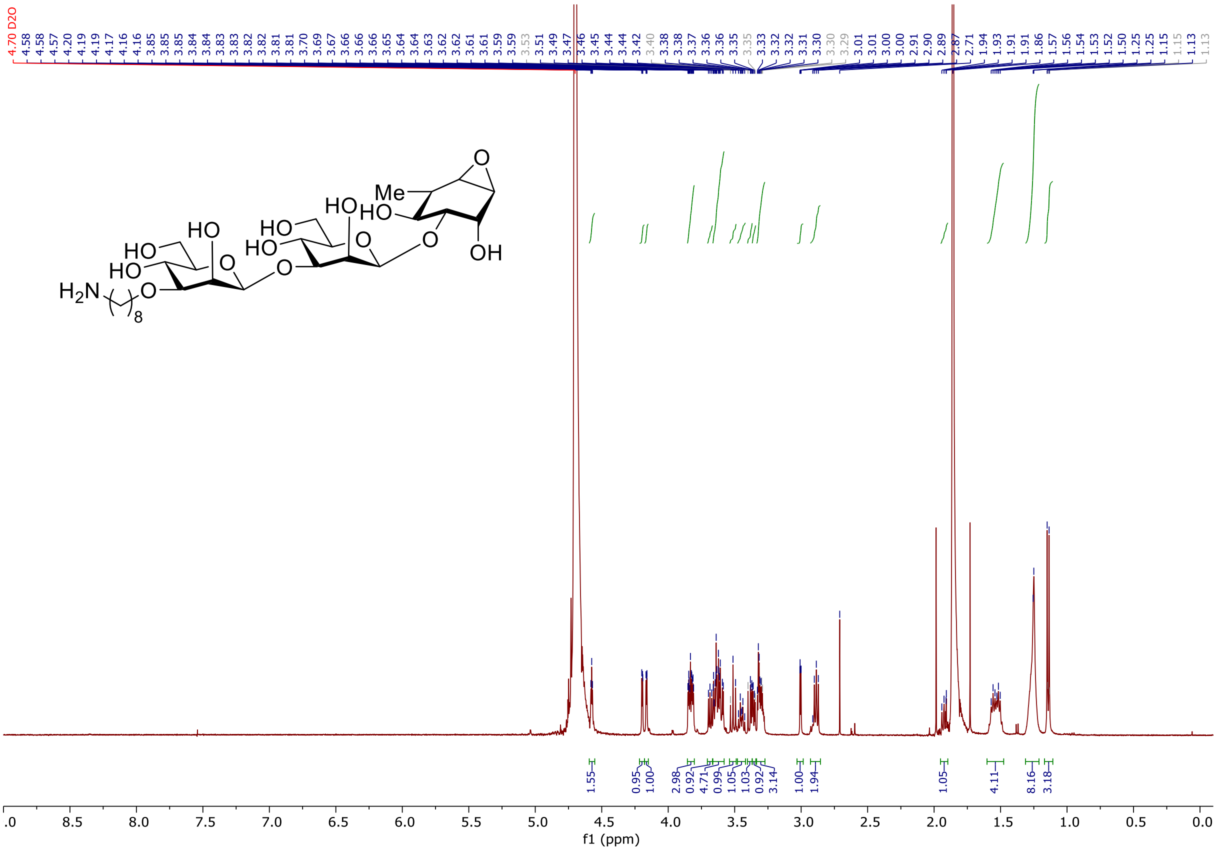

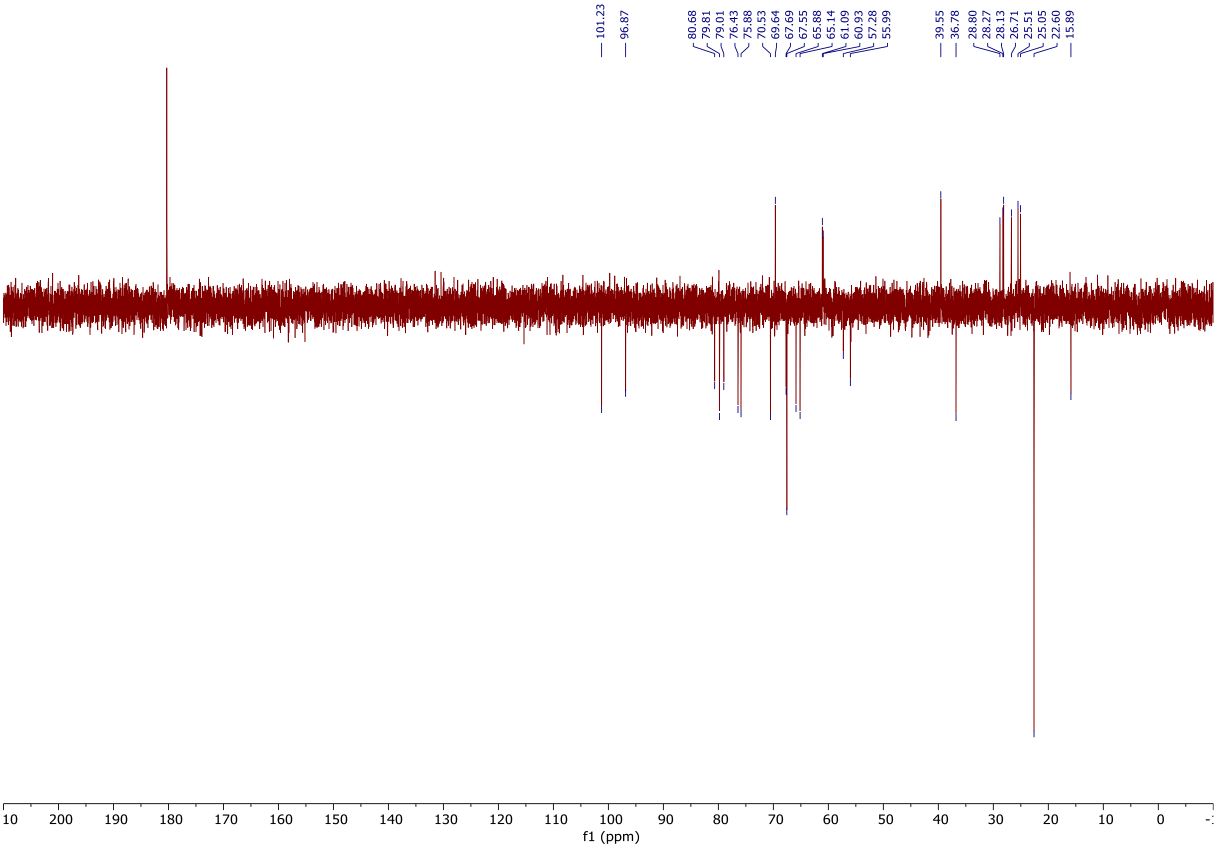

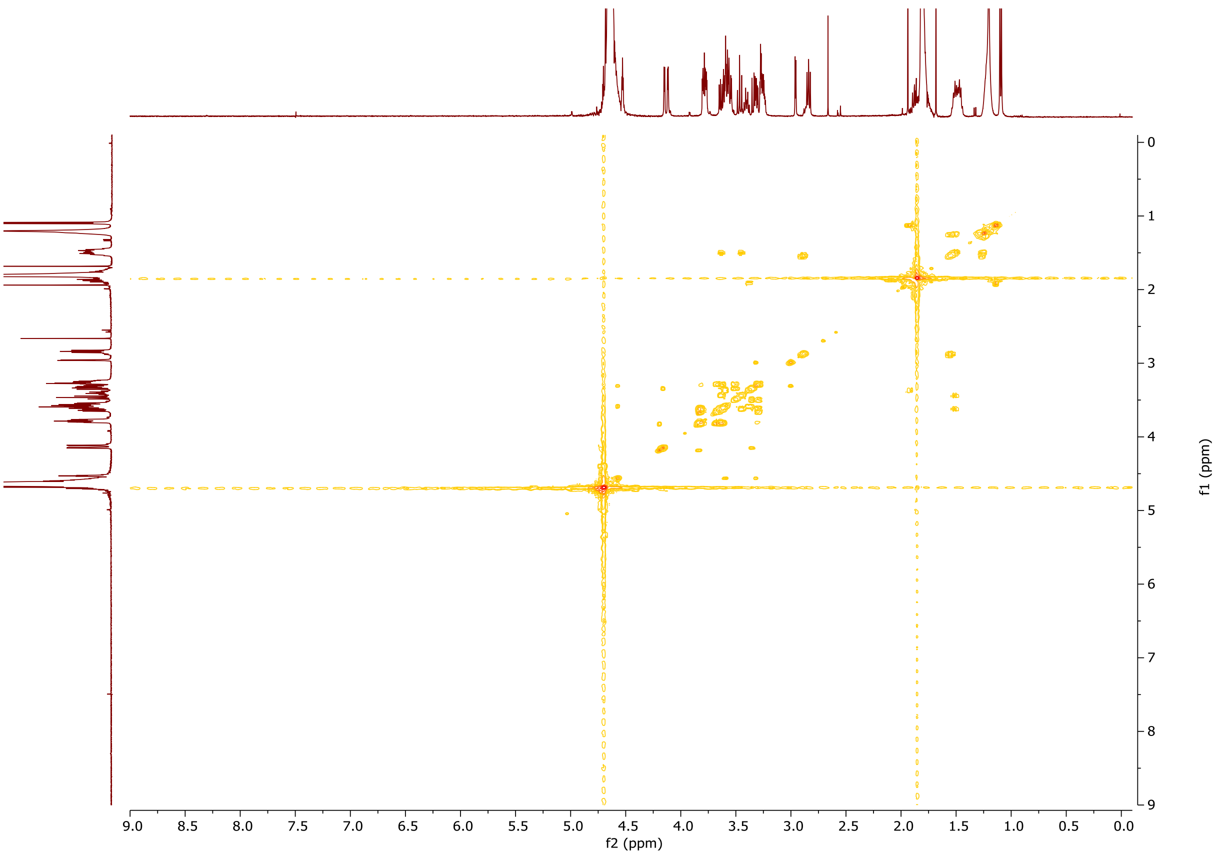
f2 (ppm)
f1 (ppm)

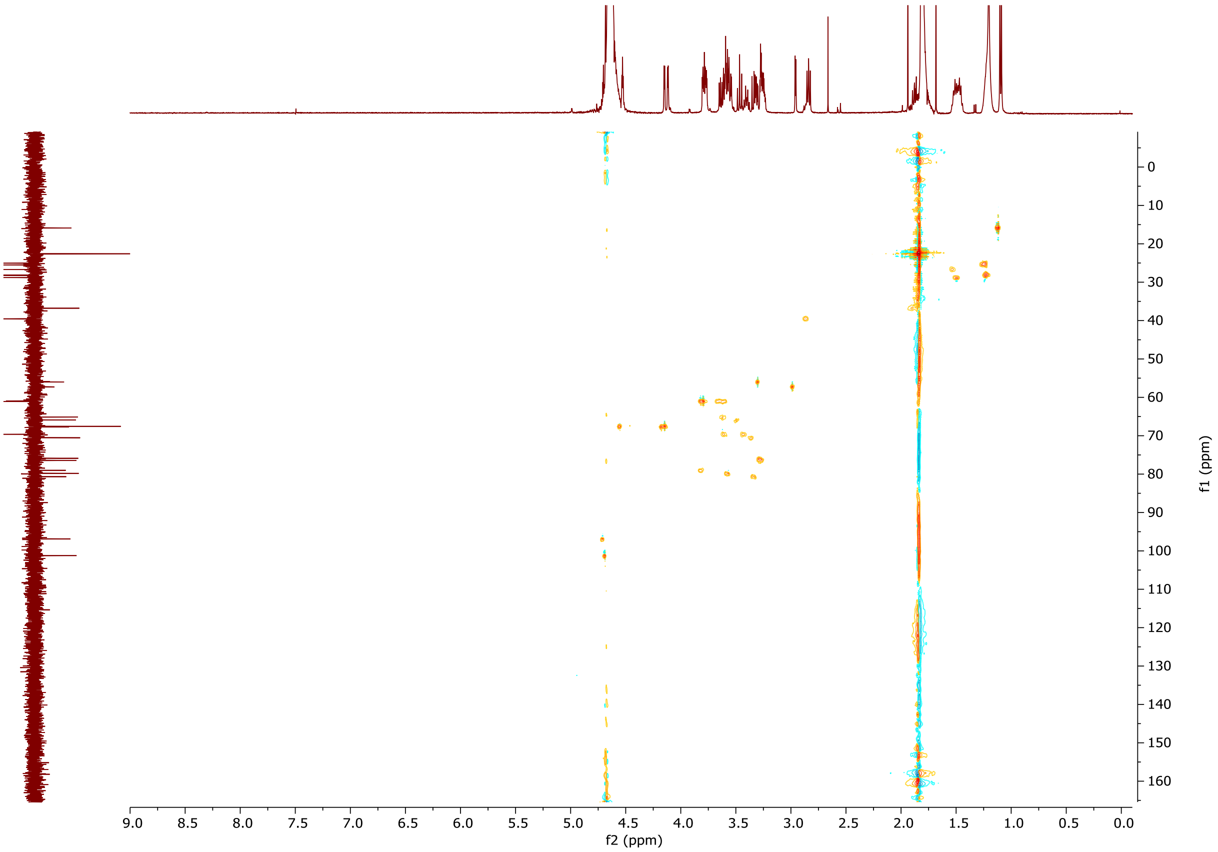
f2 (ppm)
f1 (ppm)

3-*O*-(8-amide Cy5-octane)-β-D-mannose-(1->3)-β-D-mannose-(1->3)-α-L-rhamnose-cyclophellitol (3)

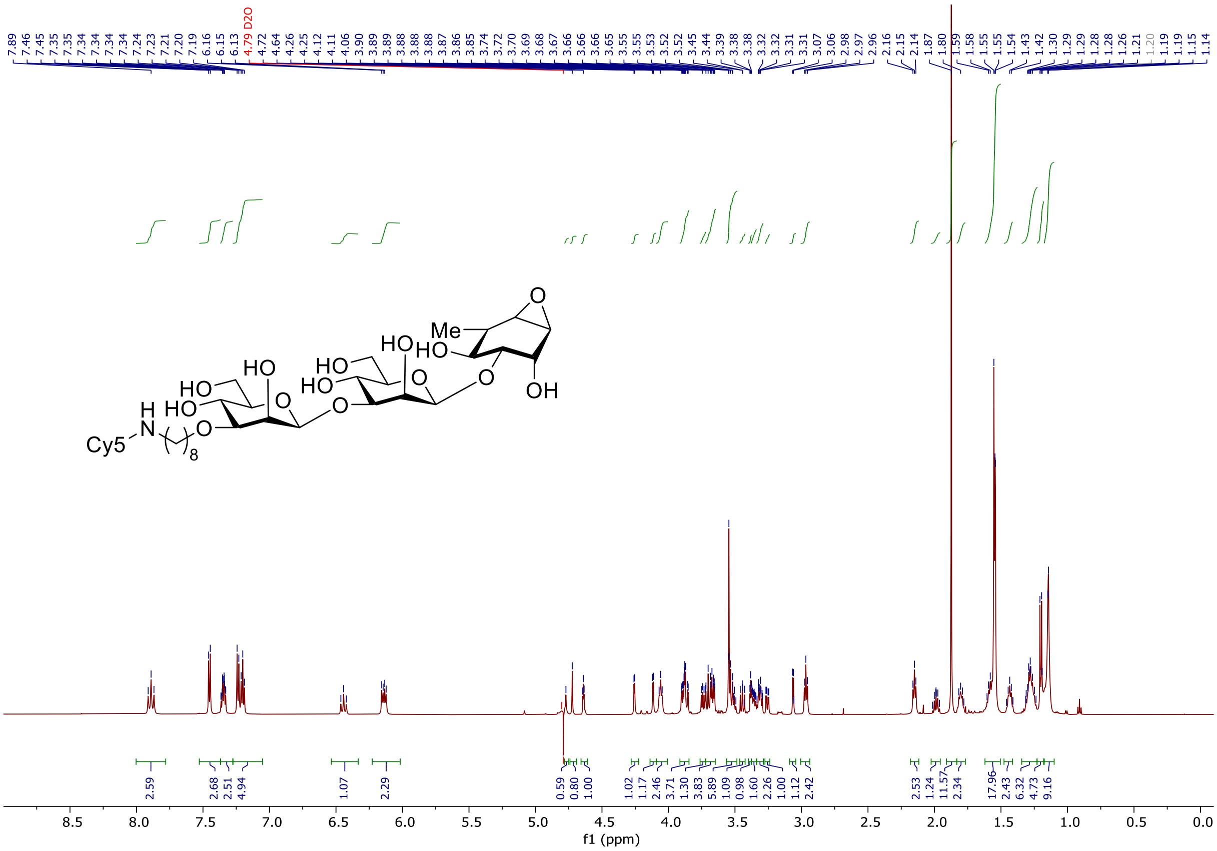

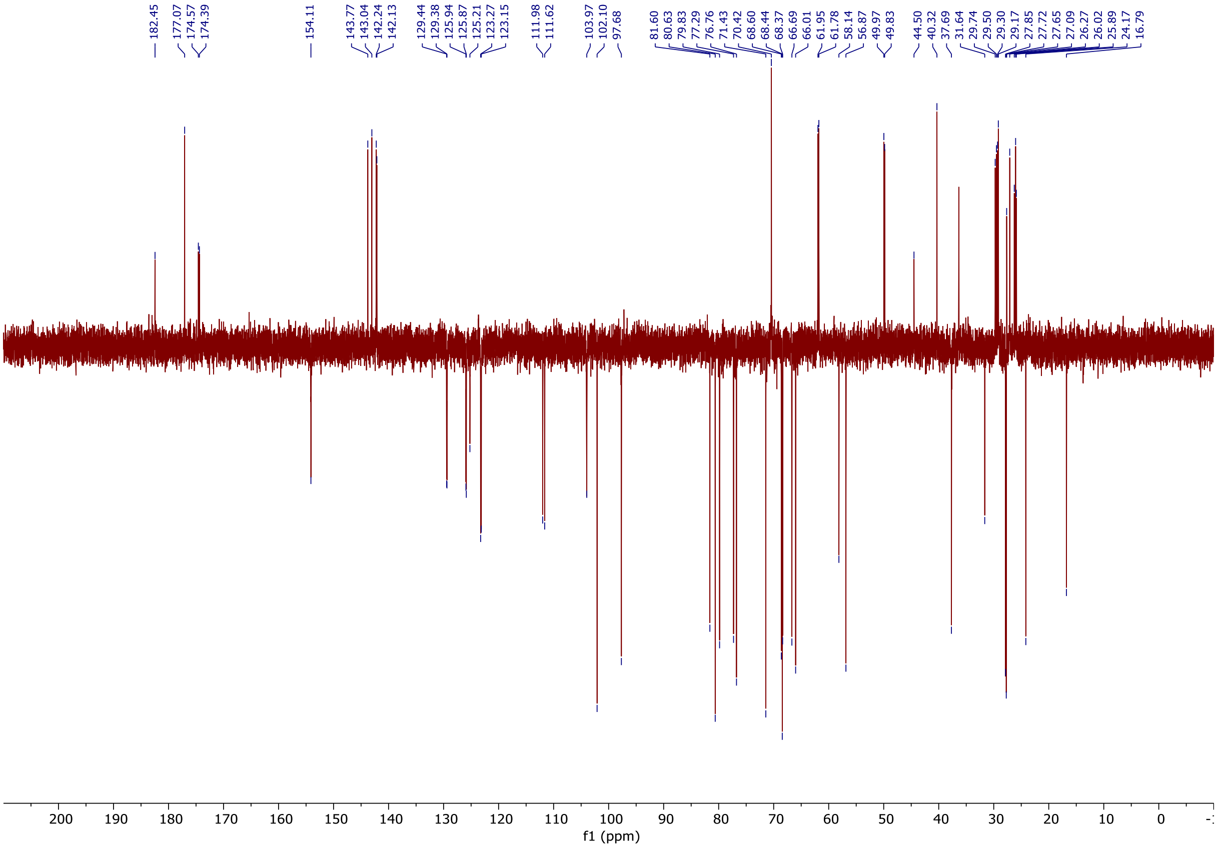

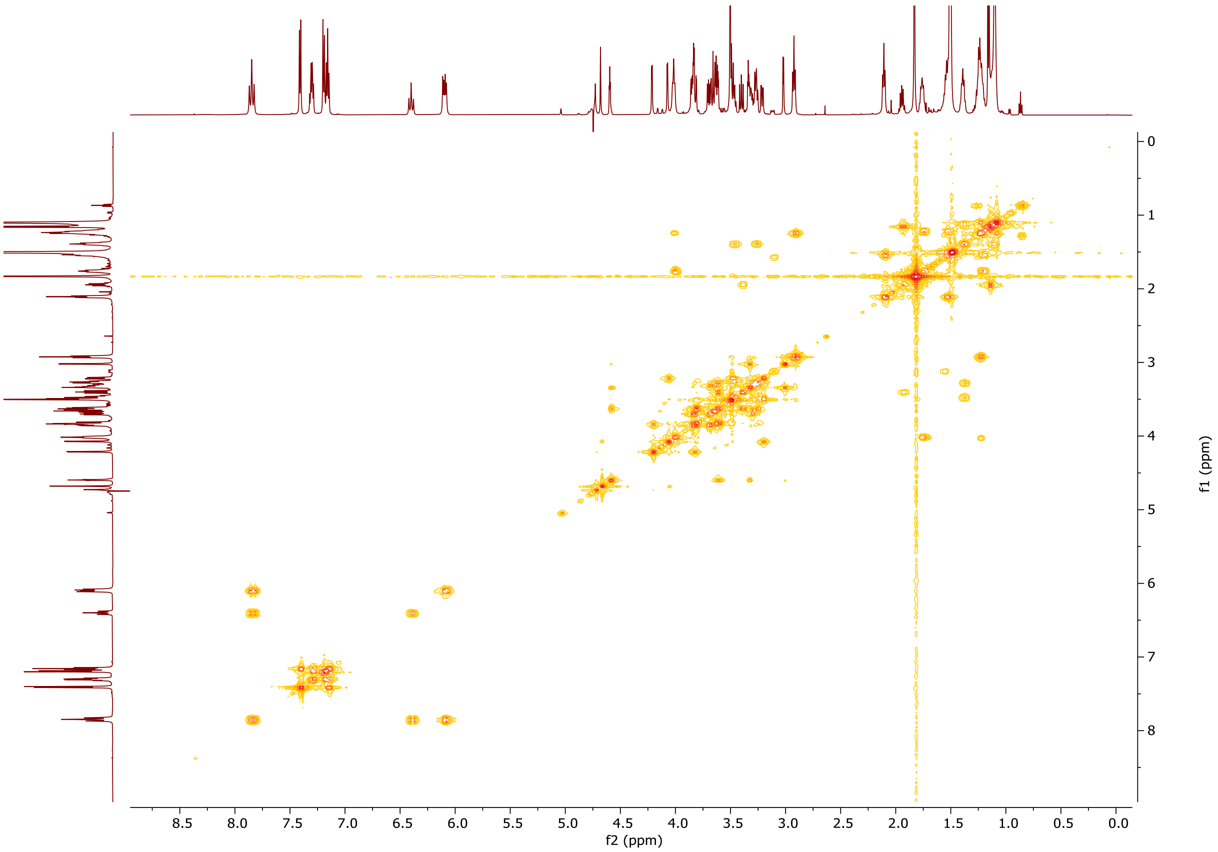
8.5 8.0 7.5 7.0 6.5 6.0 5.5 5.0 4.5 4.0 3.5 3.0 2.5 2.0 1.5 1.0 0.5 0.0
f2 (ppm)
0 1 2 3 4 5 6 7 8
f1 (ppm)

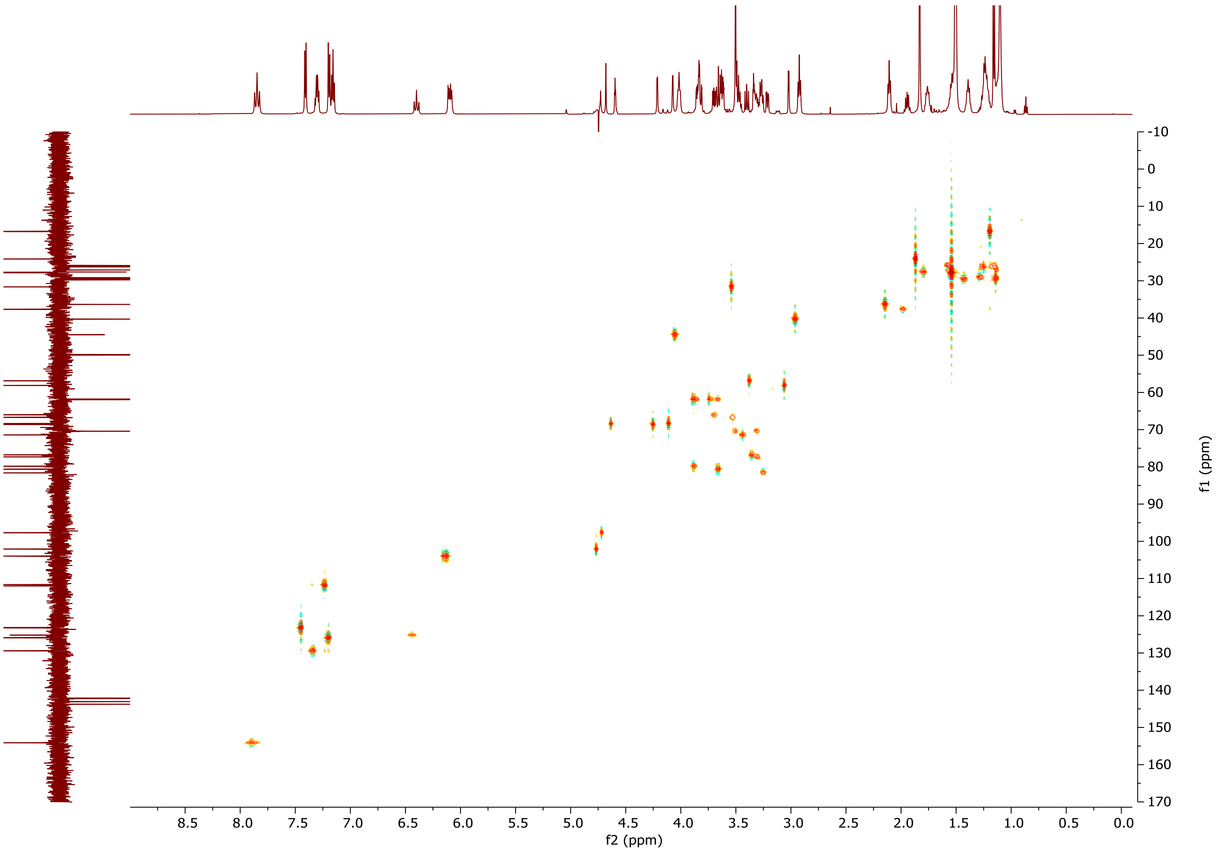
8.5 8.0 7.5 7.0 6.5 6.0 5.5 5.0 4.5 4.0 3.5 3.0 2.5 2.0 1.5 1.0 0.5 0.0
f2 (ppm)
-10 0 10 20 30 40 50 60 70 80 90 100 110 120 130 140 150 160 170
f1 (ppm)

1-*O*-allyl-2-*O*-benzoyl-4-*O*-benzyl-α-L-rhamnose (10)

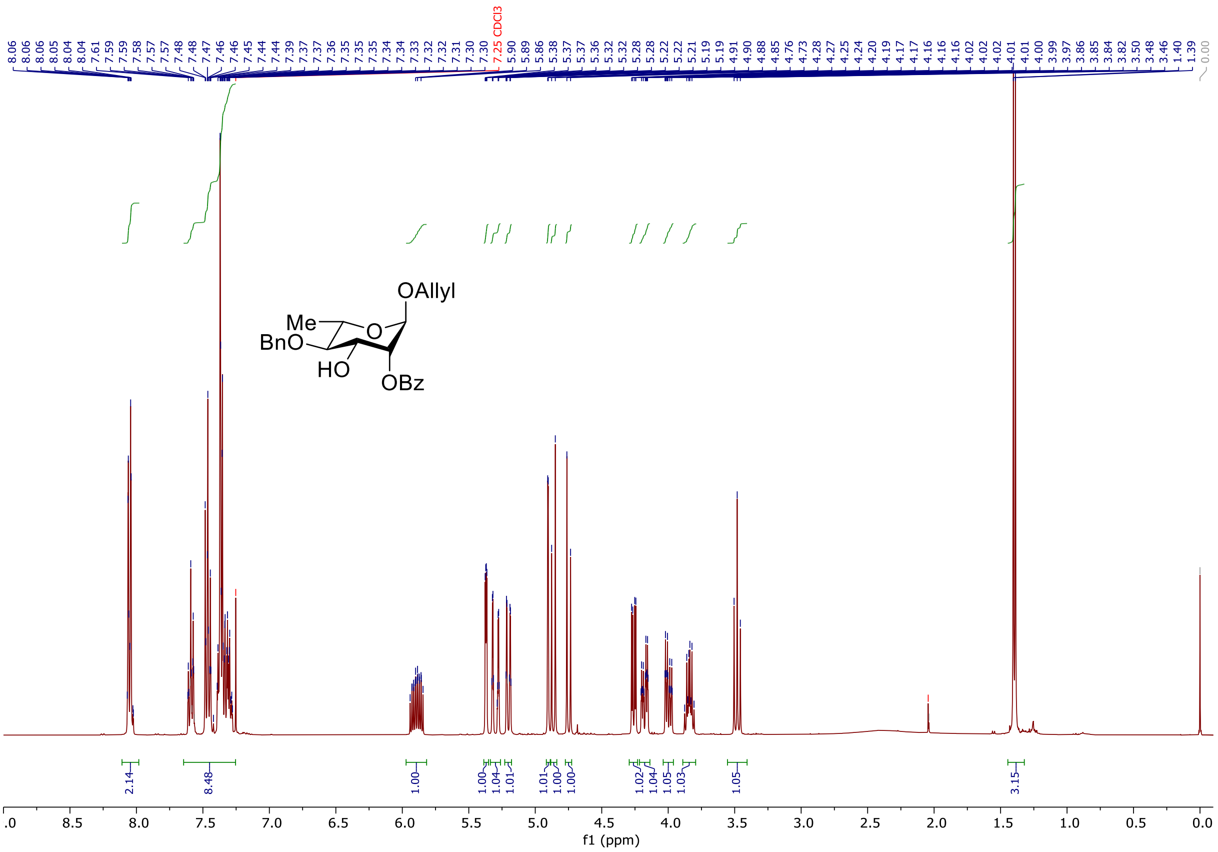

2-*O*-benzyl-4,6-*O*-benzylidene-3-*O*-(8-azido-octane)-1-*O*-trichloroimidate-α-D-mannose (9)

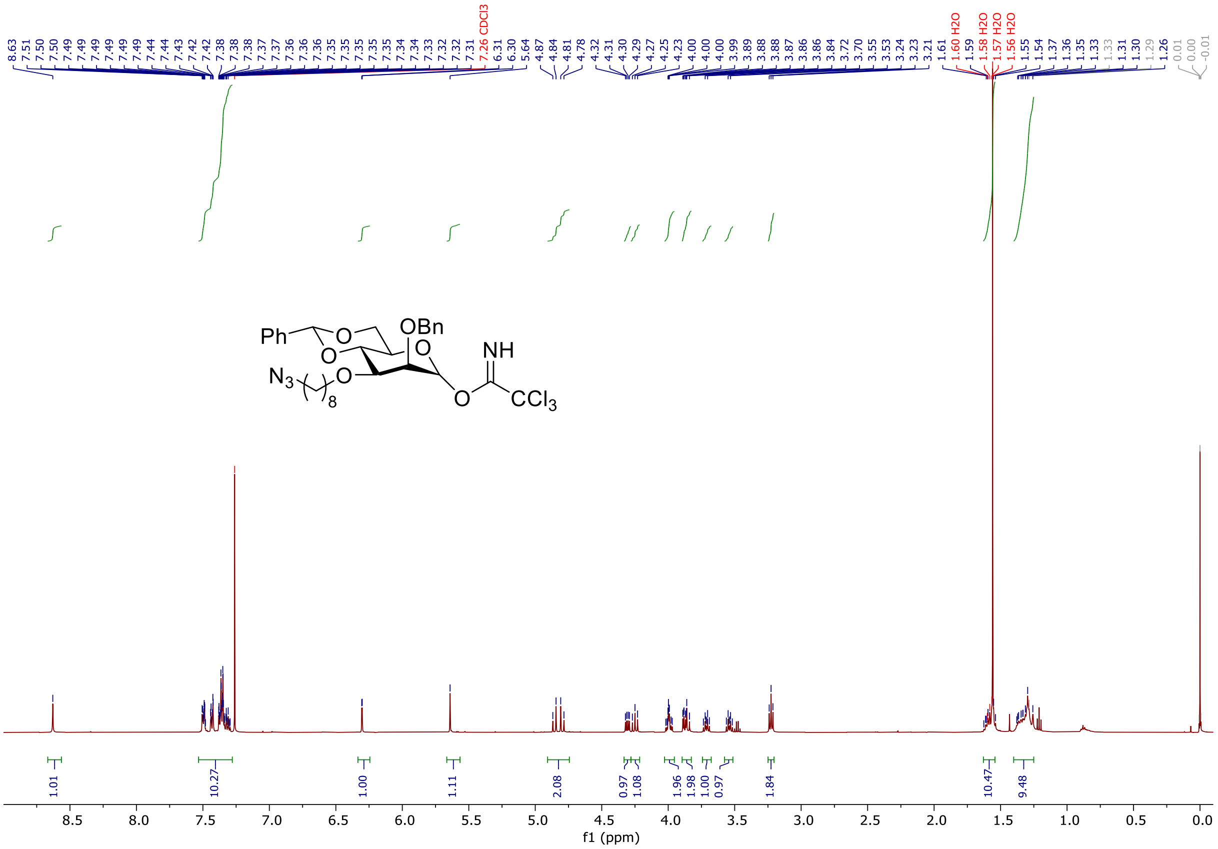

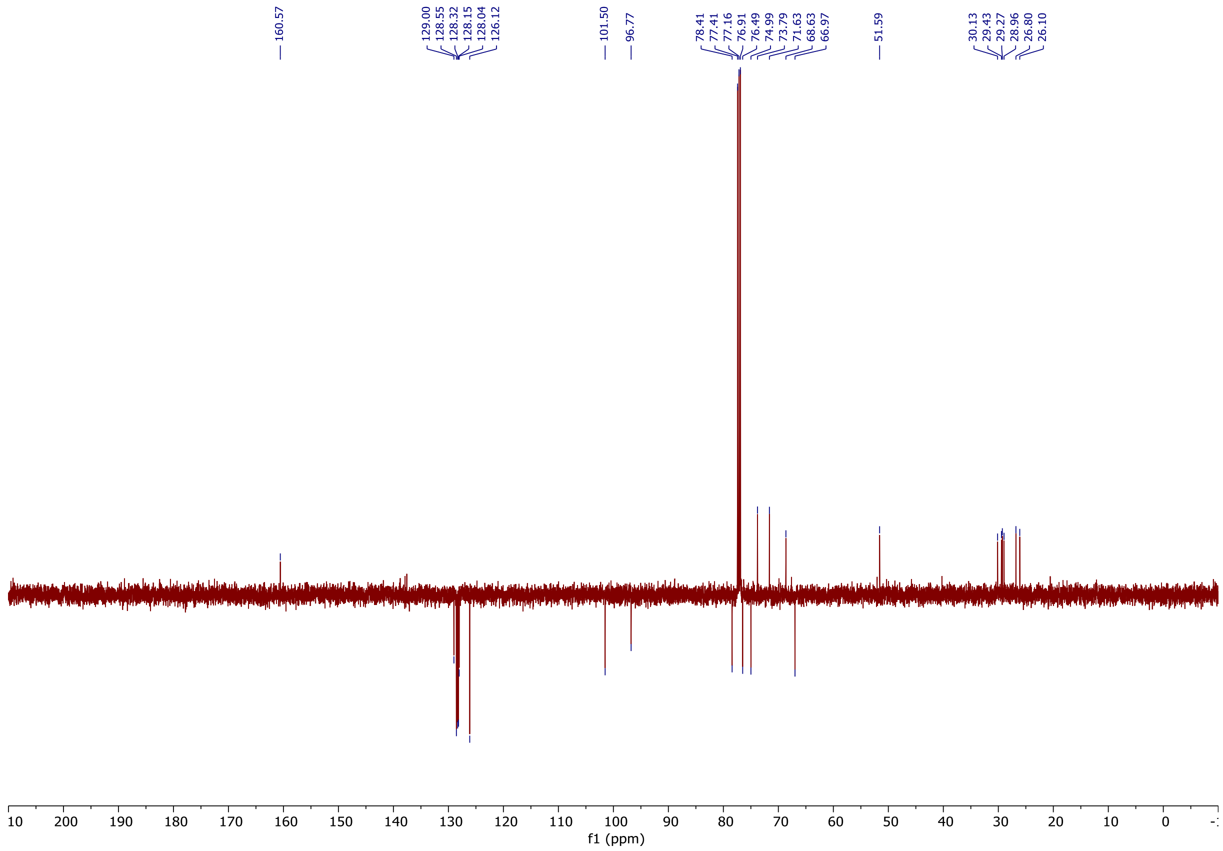

2-*O*-benzyl-3-*O*-*tert*-butyldimethylsilyl-β-glucose-cyclohexene (73)

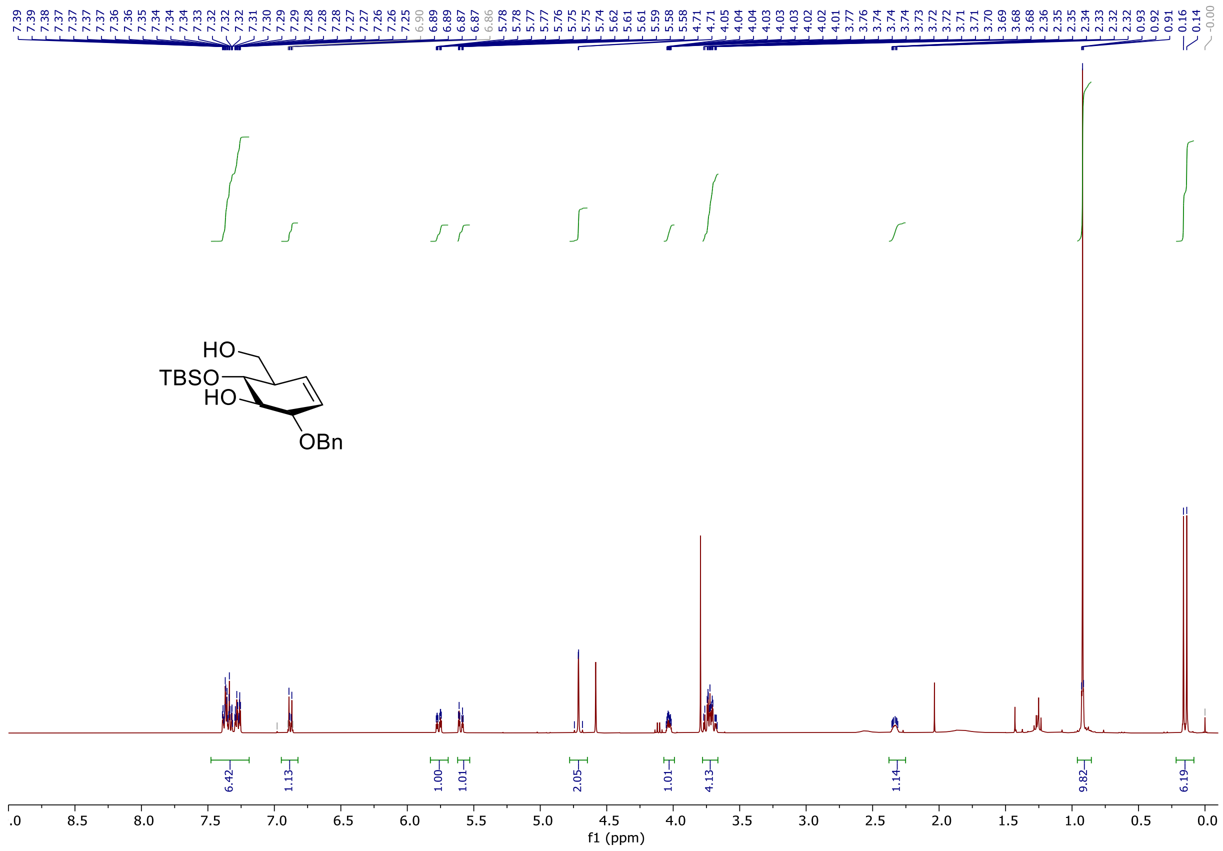

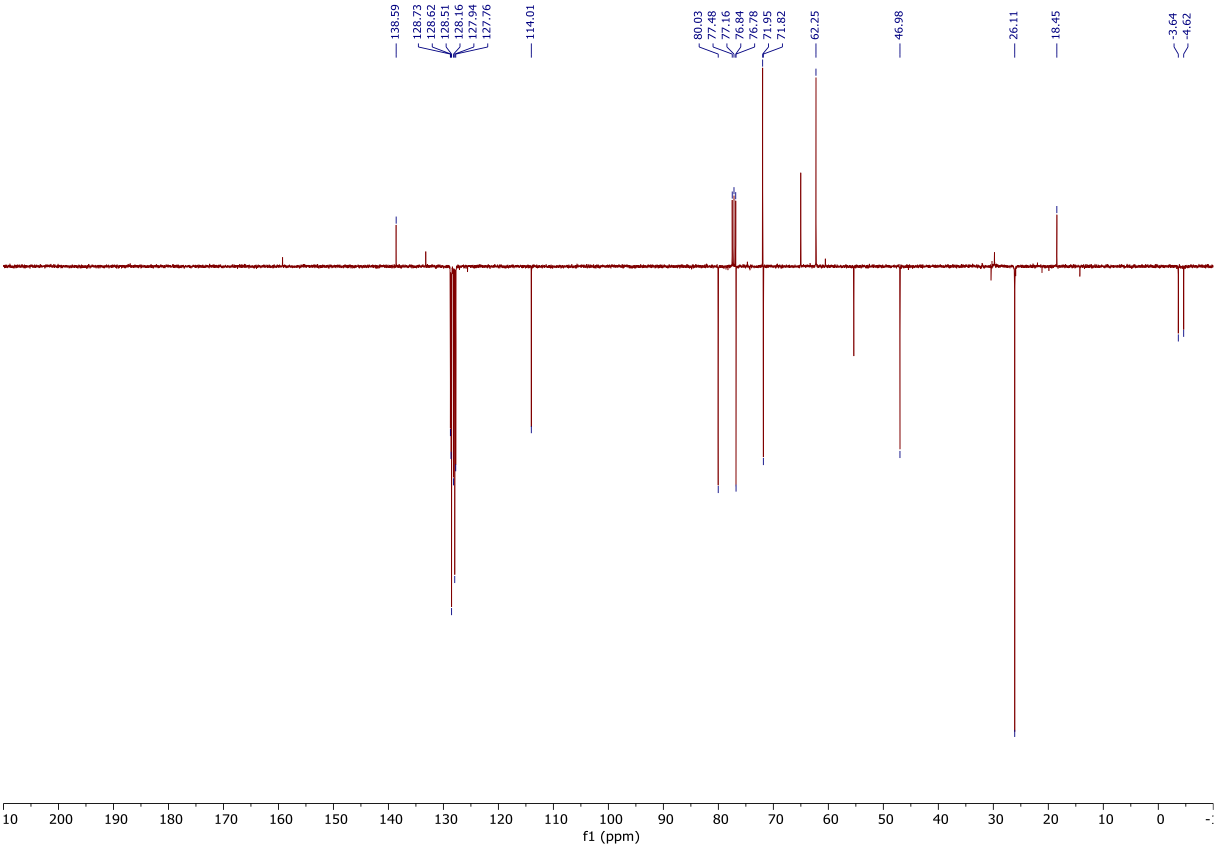

2-*O*-benzyl-6-*O*-TBDPS -3-*O*-TBS-β-glucose-cyclophellitol (7)

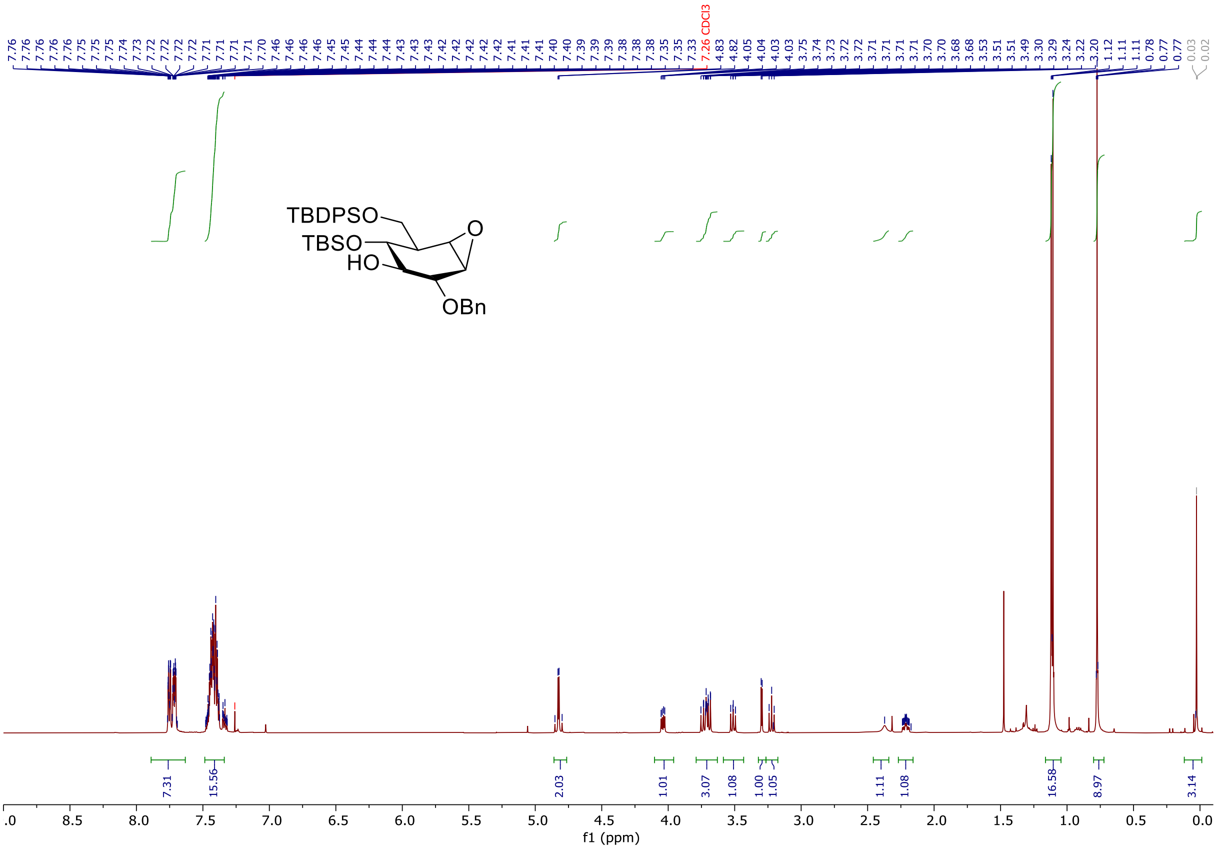

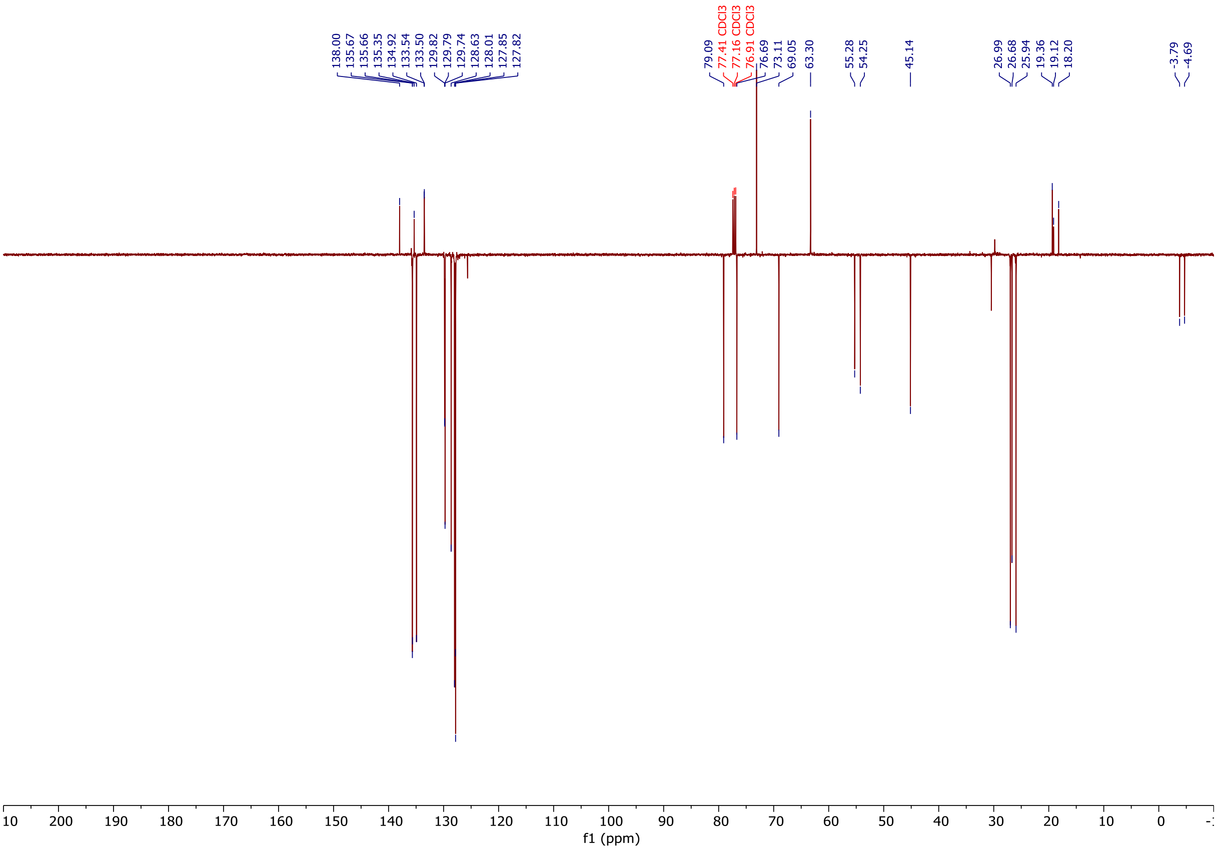

2-*O*-benzyl-4,6-*O*-benzylidene-3-*O*-(8-azido-octane)-β-D-mannose-(1->3)-1-*O*-allyl-2-*O*-benzoyl-4-*O*-benzyl-α-L-rhamnose (75)

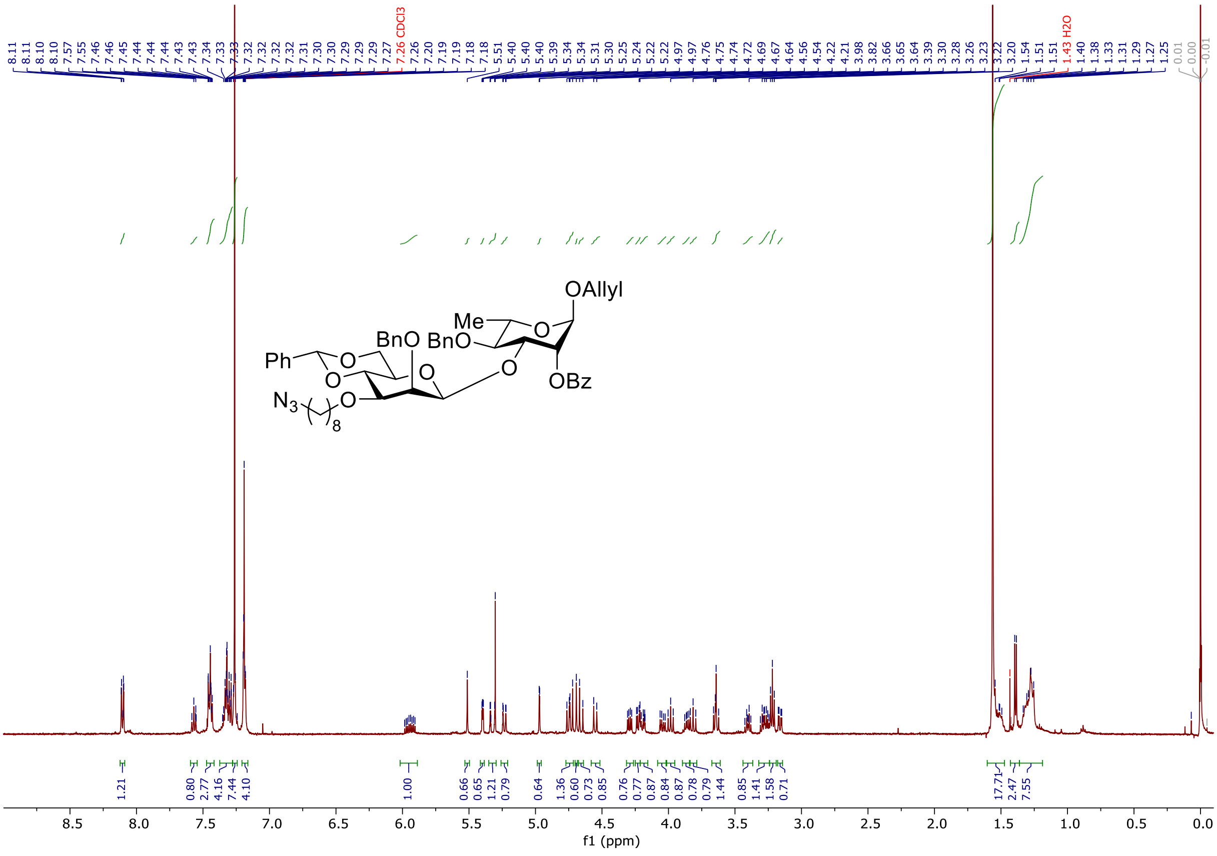

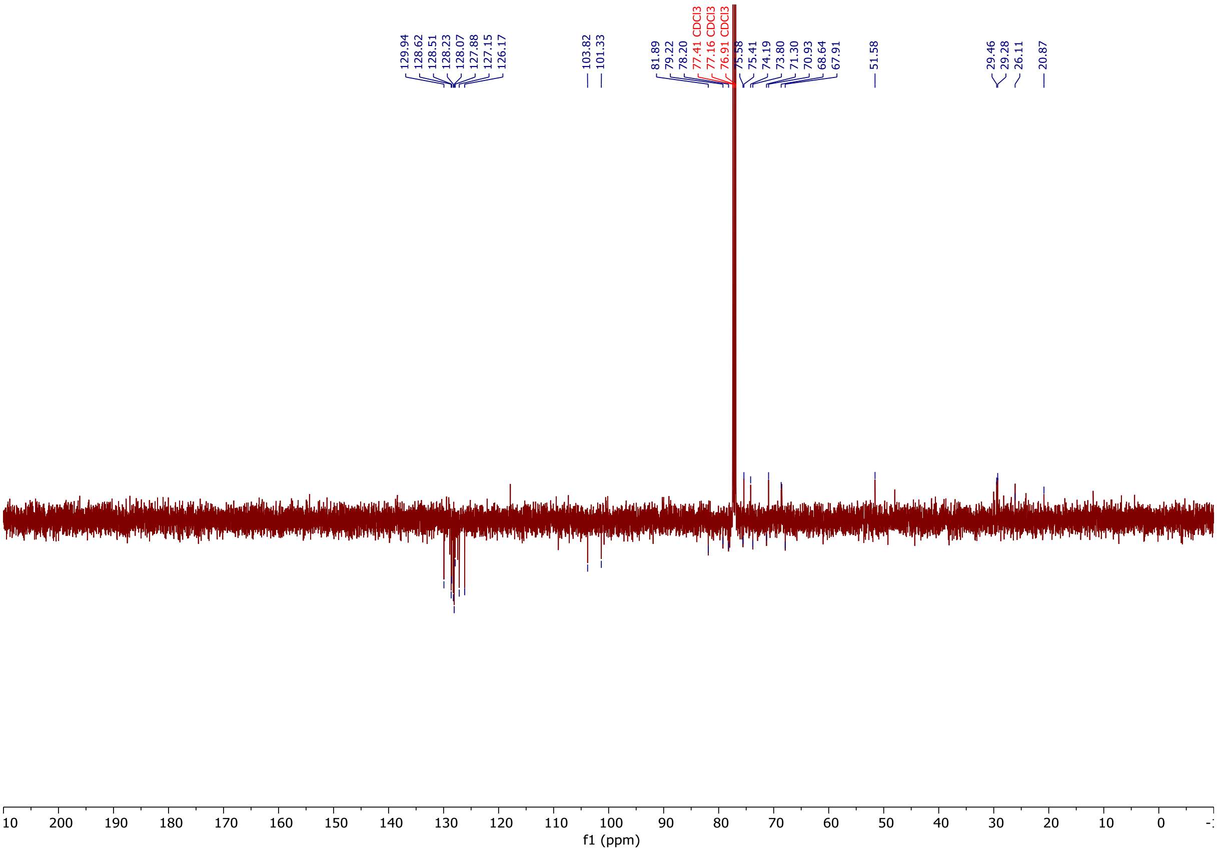
129.94
128.62
128.51
128.23
128.07
127.88
127.15
126.17
103.82
101.33
81.89
79.22
78.20
77.41 CDCl3
77.16 CDCl3
76.91 CDCl3
75.58
75.41
74.19
73.80
71.30
70.93
68.64
67.91
51.58
29.46
29.28
26.11
20.87
f1 (ppm)

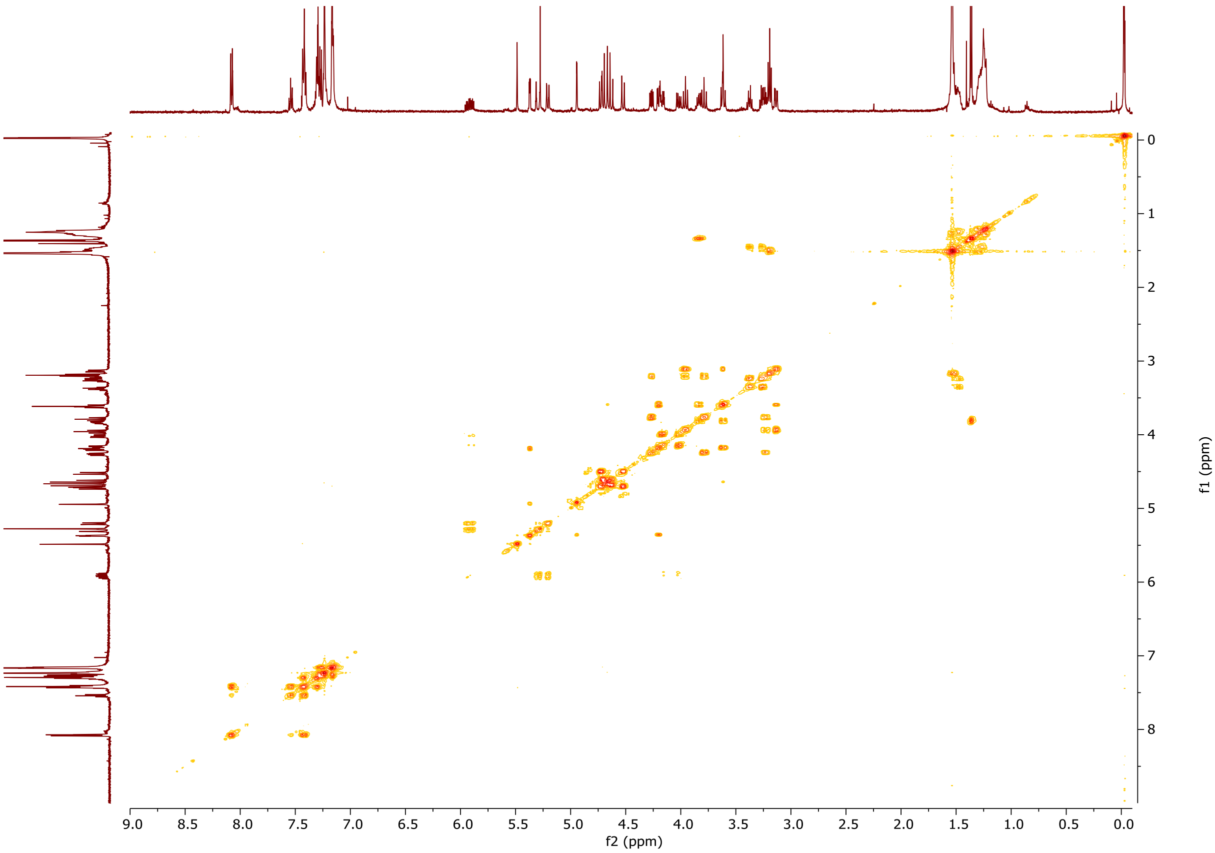
f2 (ppm)
f1 (ppm)

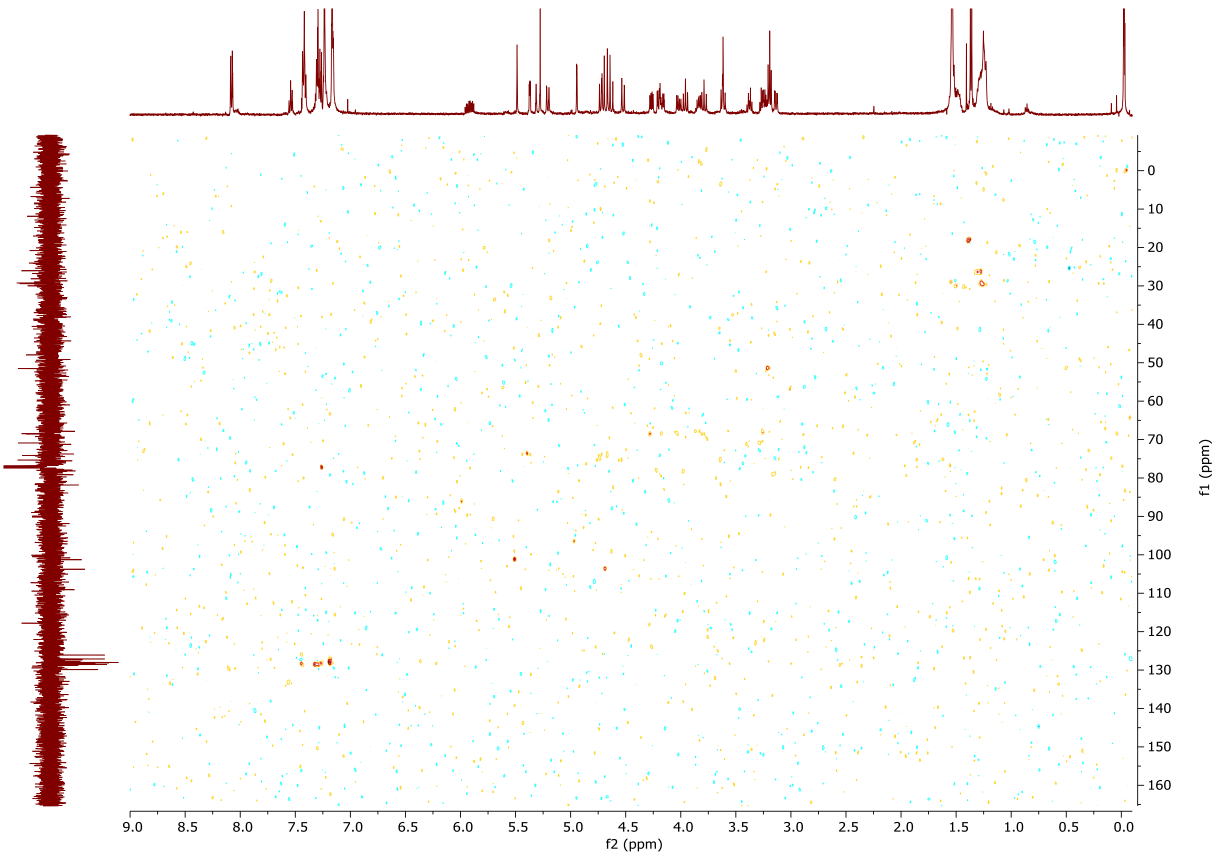

2-*O*-benzyl-4,6-*O*-benzylidene-3-*O*-(8-azido-octane)-β-D-mannose-(1->3)-2-*O*-benzoyl-4-*O*-benzyl-L-rhamnose (75a)

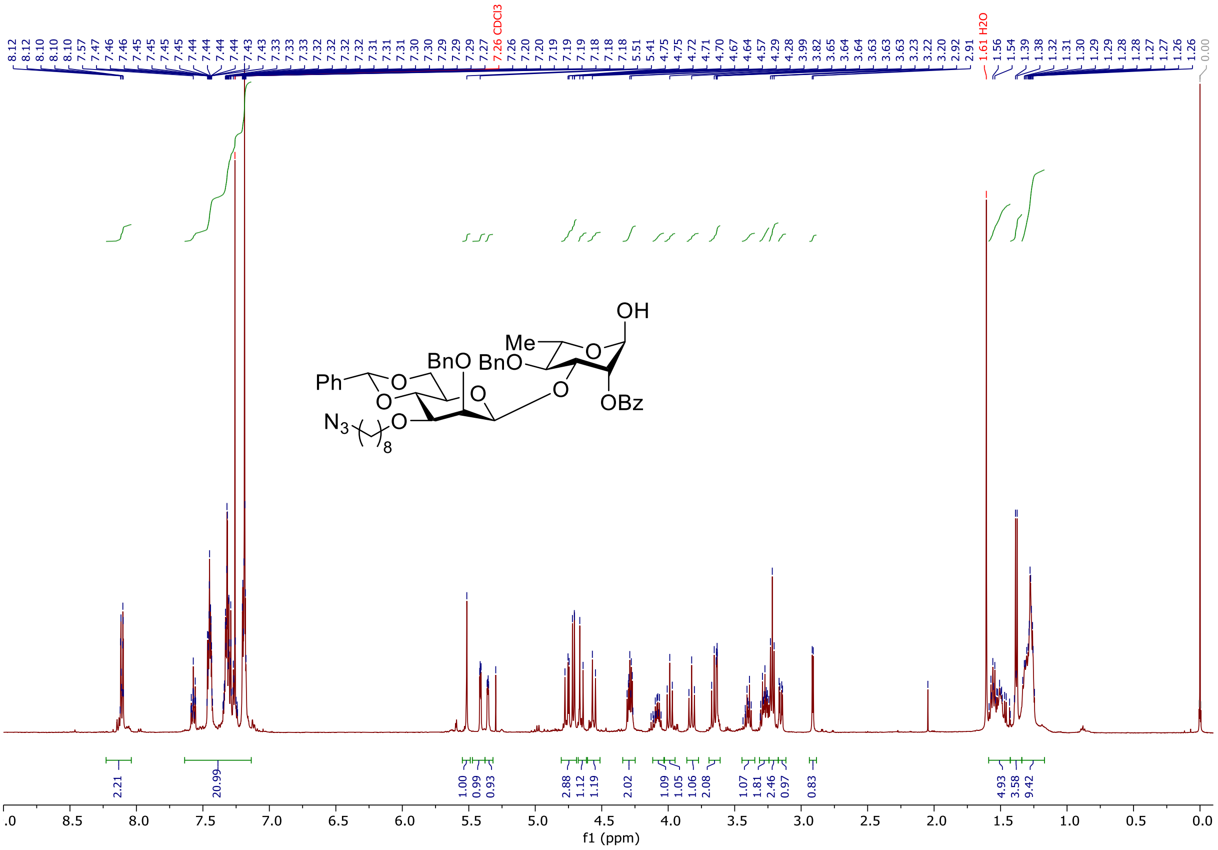

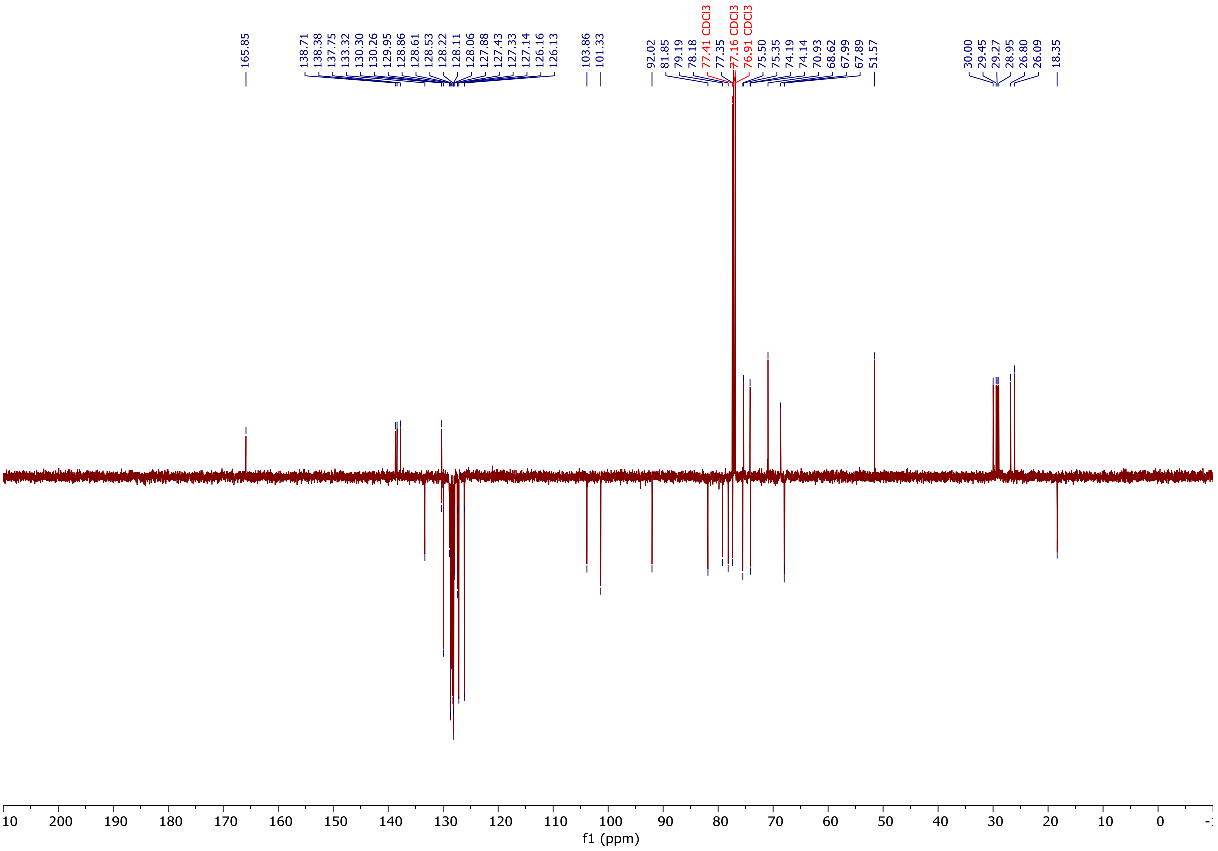
165.85
138.71
138.38
137.75
133.32
130.30
130.26
129.95
128.86
128.61
128.53
128.22
128.11
128.06
127.88
127.43
127.33
127.14
126.16
126.13
103.86
101.33
92.02
81.85
79.19
78.18
77.41 CDCl3
77.35
77.16 CDCl3
76.91 CDCl3
75.50
75.35
74.19
74.14
70.93
68.62
67.99
67.89
51.57
30.00
29.45
29.27
28.95
26.80
26.09
18.35
f1 (ppm)

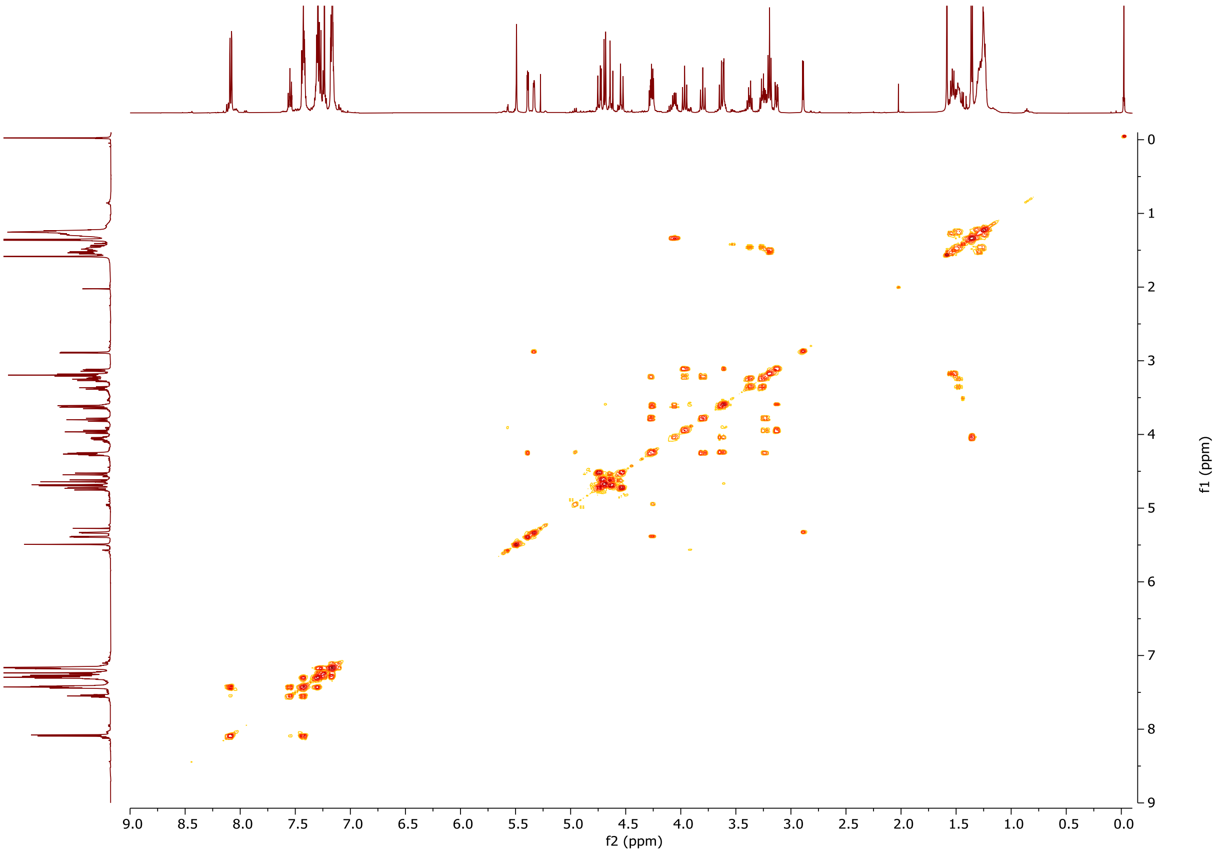
f2 (ppm)
f1 (ppm)

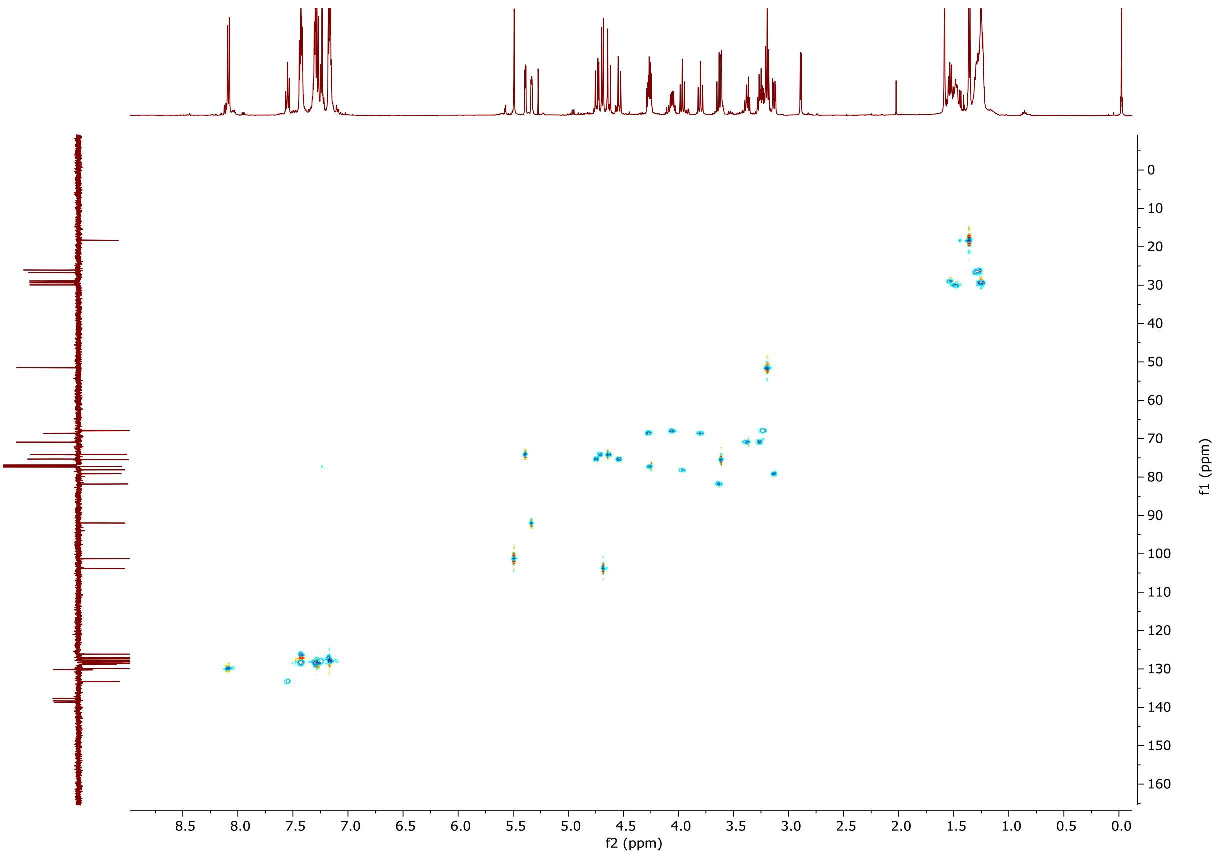

8.5 8.0 7.5 7.0 6.5 6.0 5.5 5.0 4.5 4.0 3.5 3.0 2.5 2.0 1.5 1.0 0.5 0.0
f2 (ppm)
0 10 20 30 40 50 60 70 80 90 100 110 120 130 140 150 160
f1 (ppm)

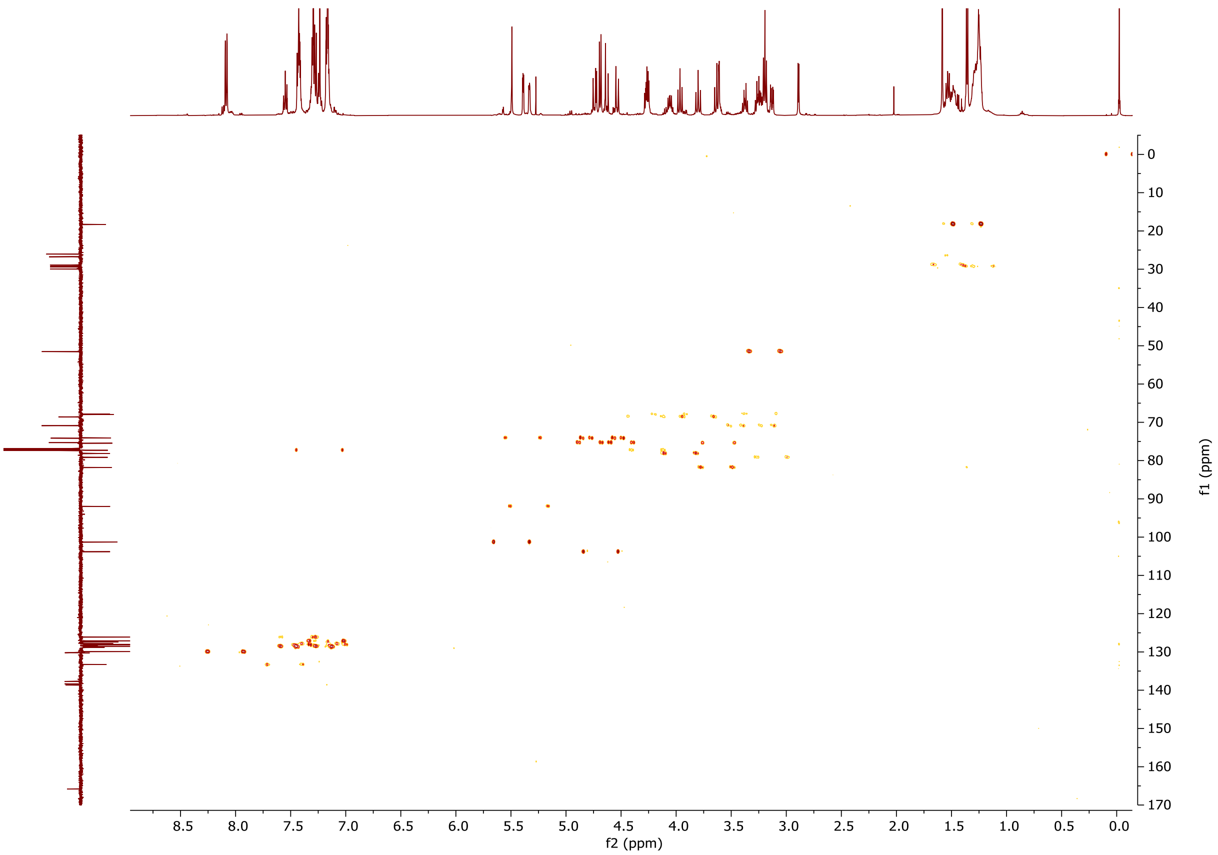

8.5 8.0 7.5 7.0 6.5 6.0 5.5 5.0 4.5 4.0 3.5 3.0 2.5 2.0 1.5 1.0 0.5 0.0
f2 (ppm)
0 10 20 30 40 50 60 70 80 90 100 110 120 130 140 150 160 170
f1 (ppm)

2-*O*-benzyl-4,6-*O*-benzylidene-3-*O*-(8-azido-octane)-β-D-mannose-(1->3)-2-*O*-benzoyl-4-*O*-benzyl-1-*O*-trichloroimidate-α-L-rhamnose (11)

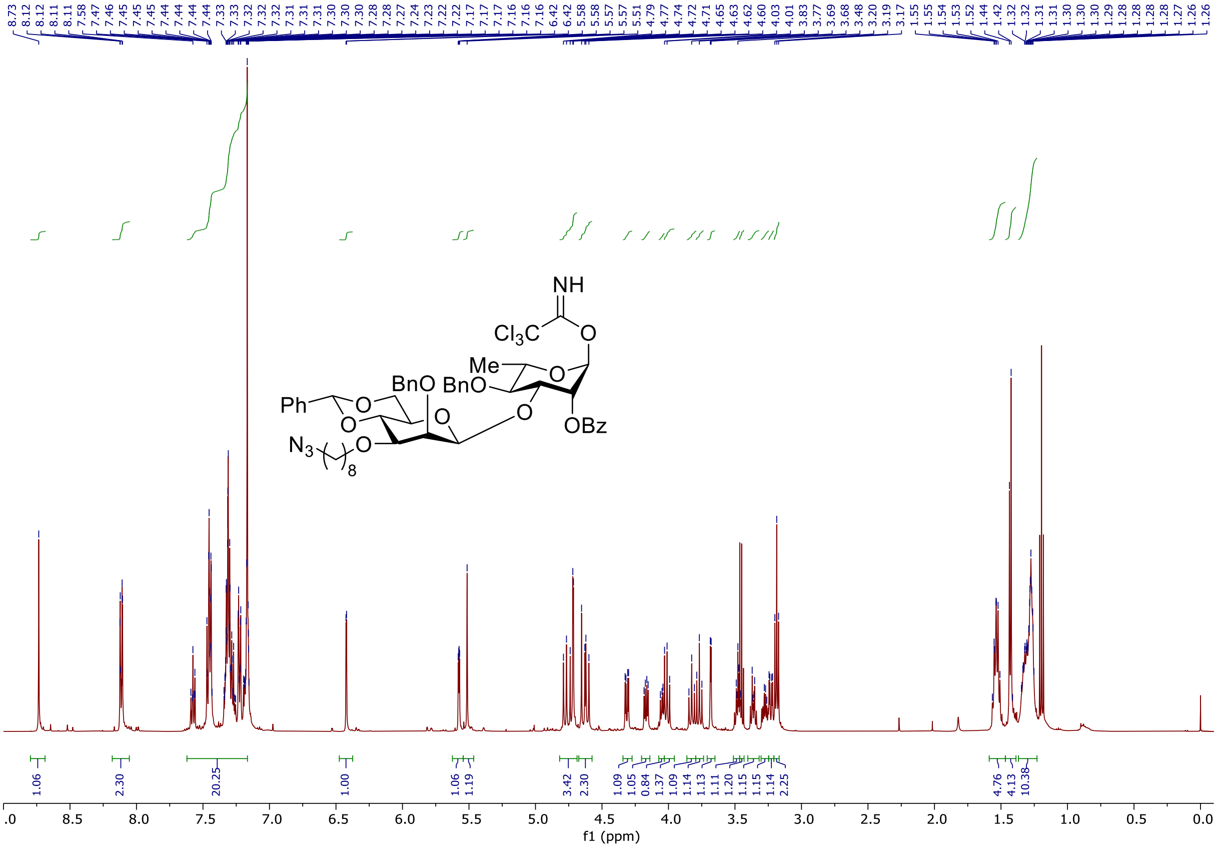

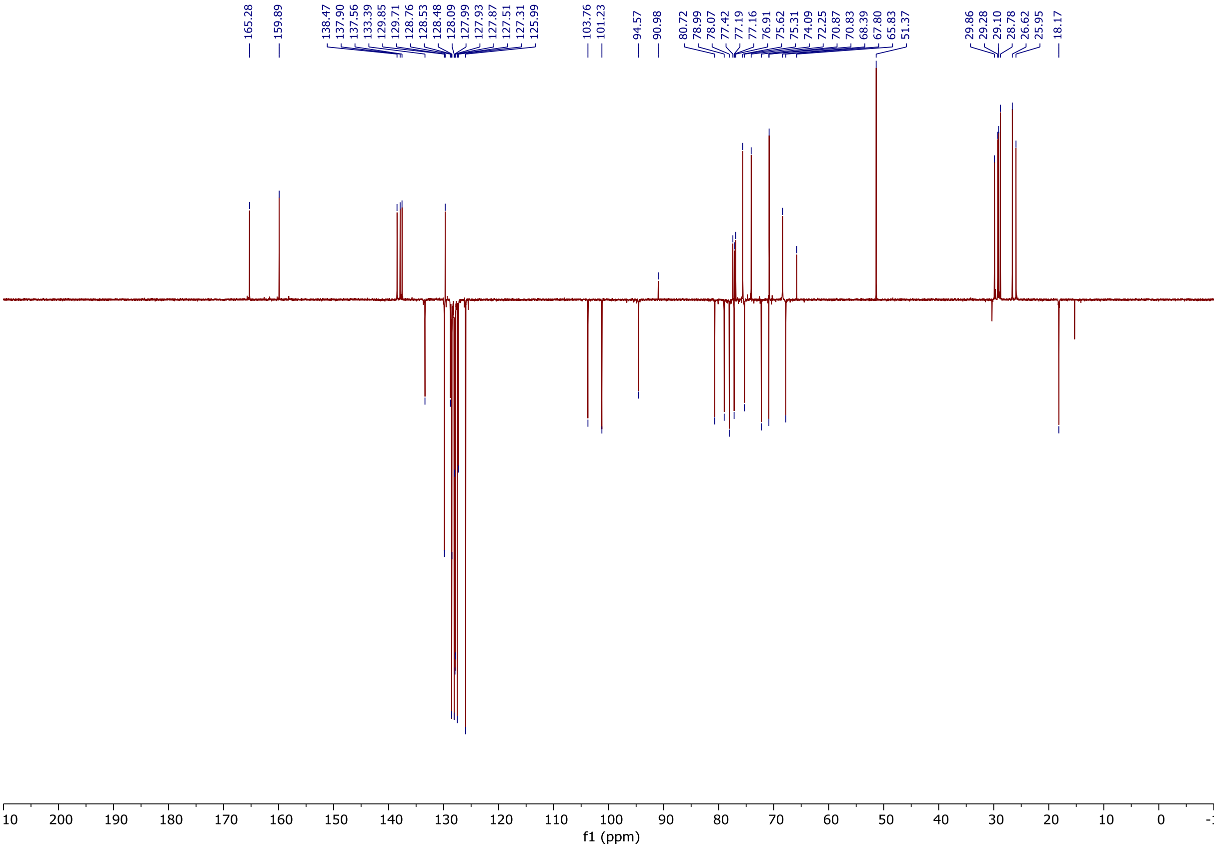

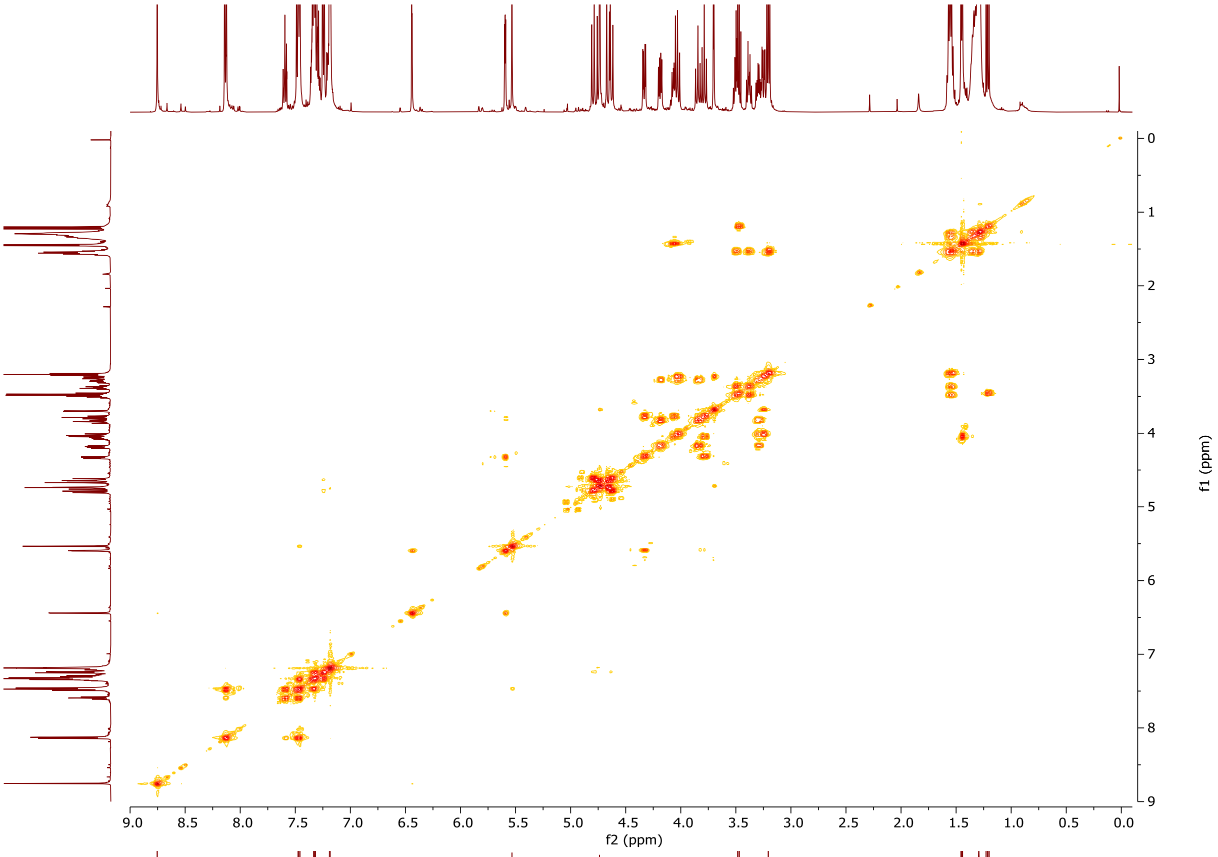
f2 (ppm)
f1 (ppm)

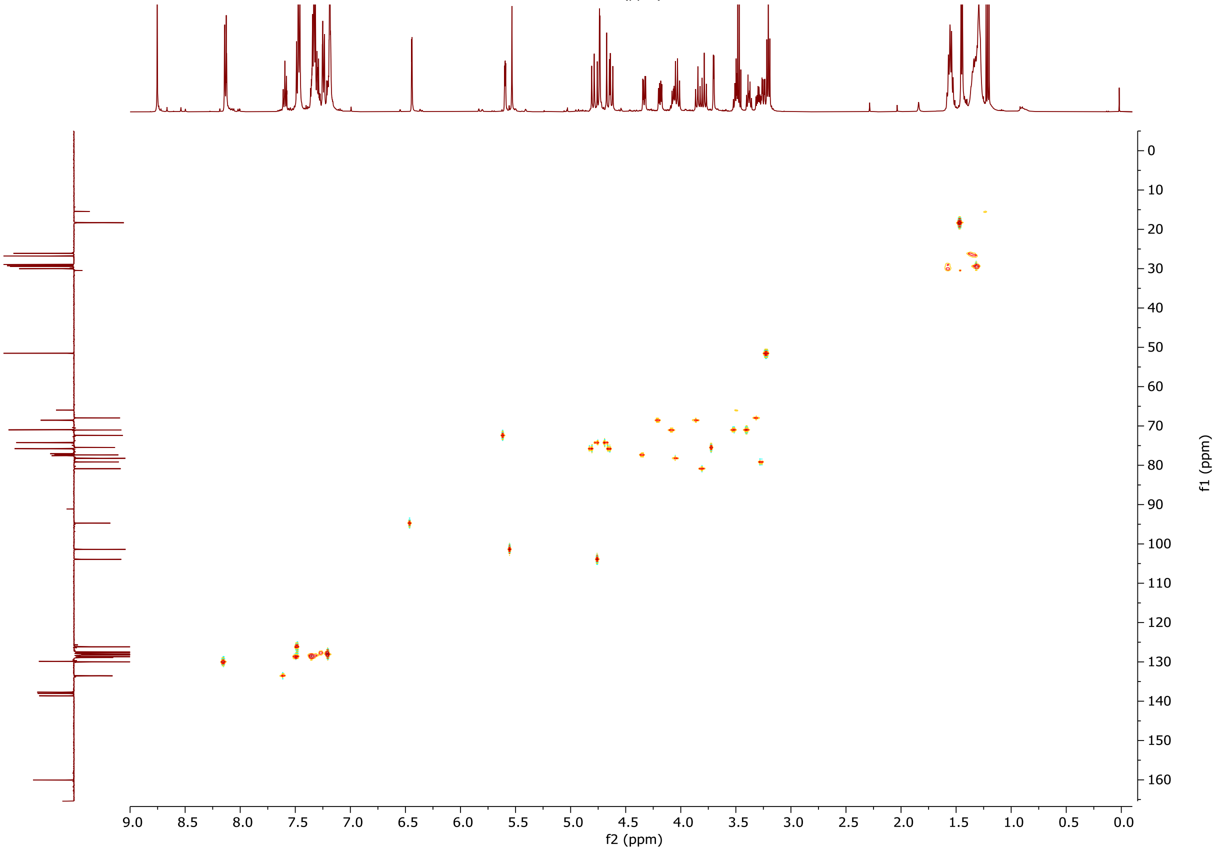
f2 (ppm)
f1 (ppm)

2-*O*-benzyl-4,6-*O*-benzylidene-3-*O*-(8-azido-octane)-β-D-mannose-(1->3)-2-*O*-benzoyl-4-*O*-benzyl-α-L-rhamnose-(1->3)-2-*O*-benzyl-6-*O*-TBDPS-3-*O*-TBS-β-glucose-cyclophellitol (12)

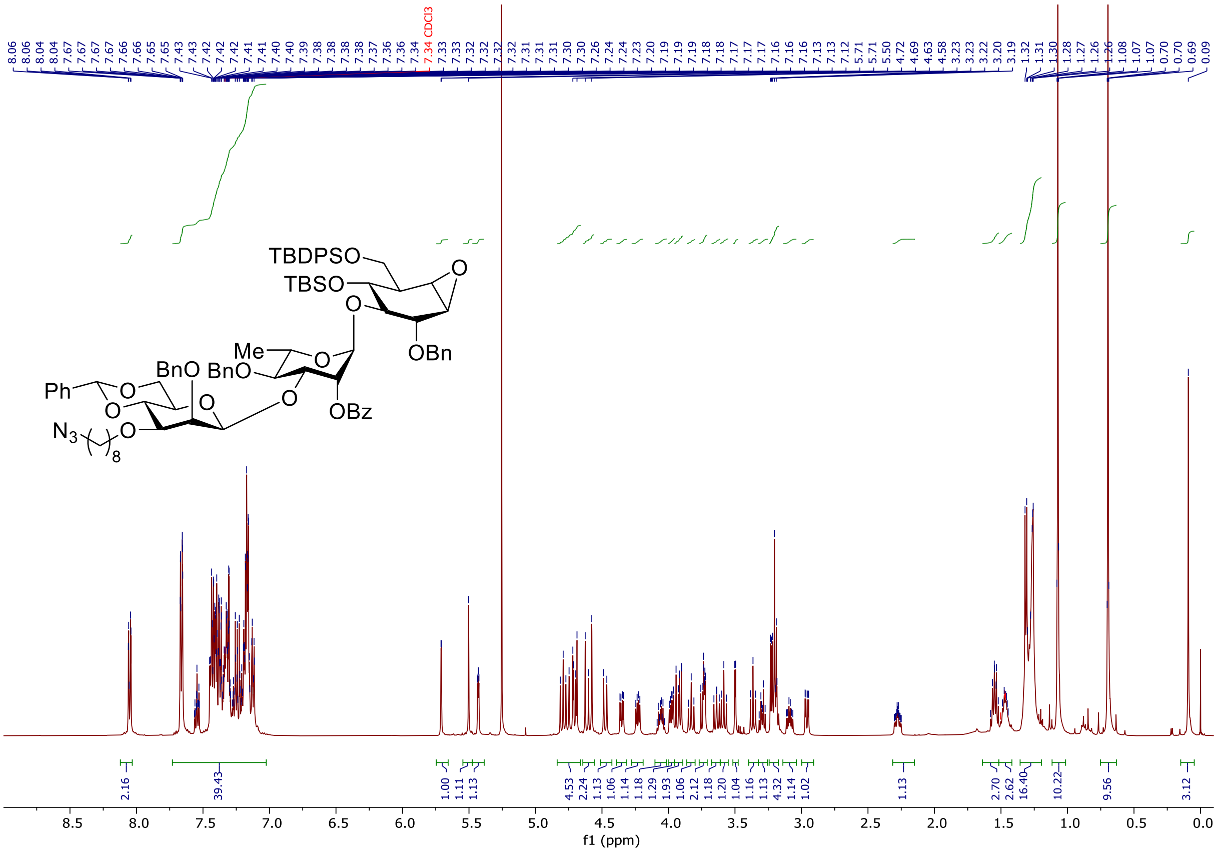

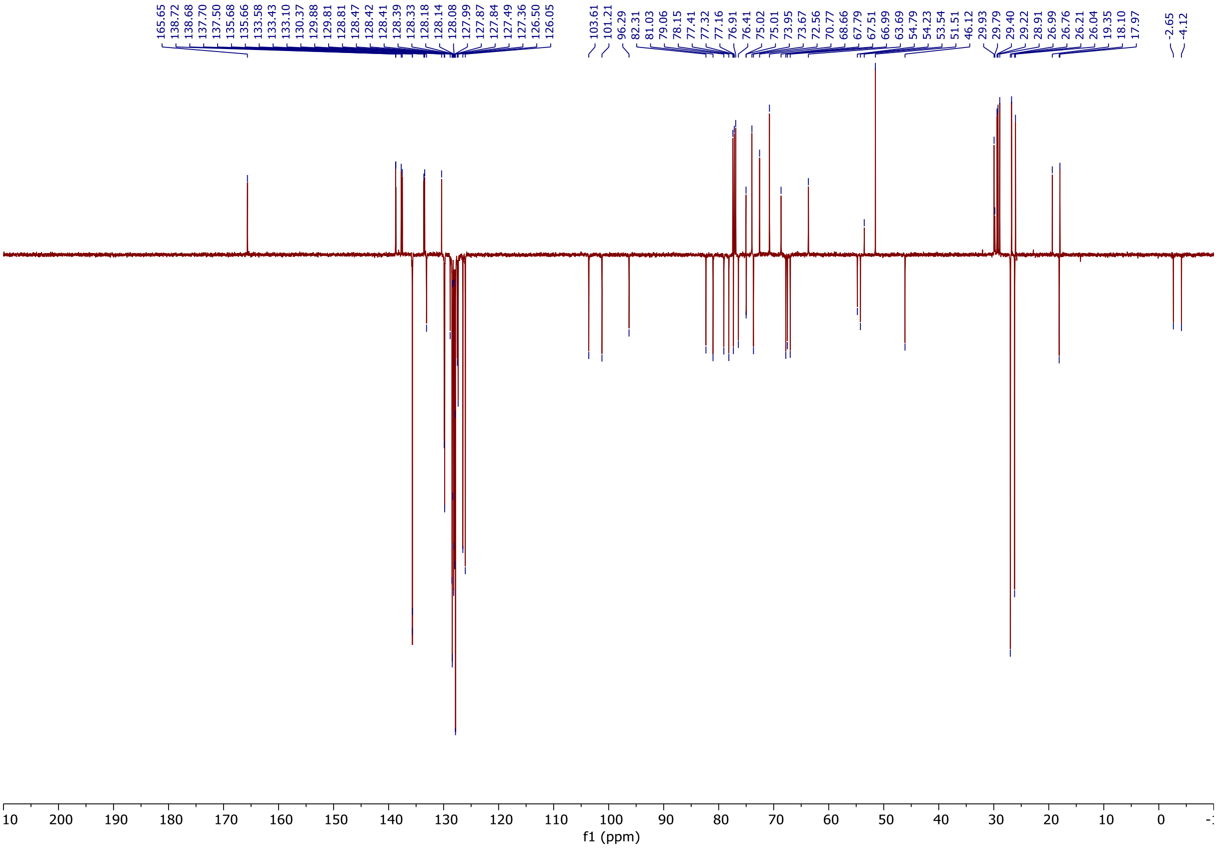

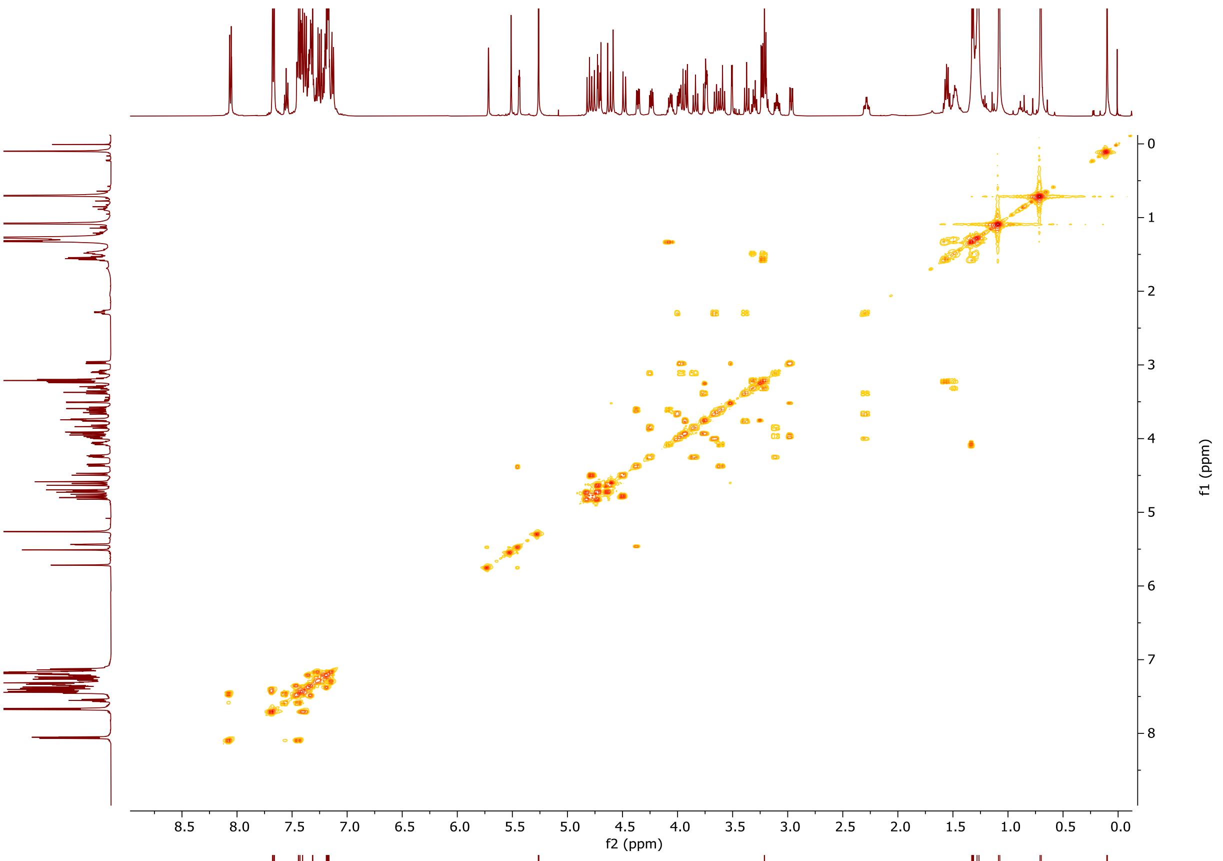
f2 (ppm)
f1 (ppm)

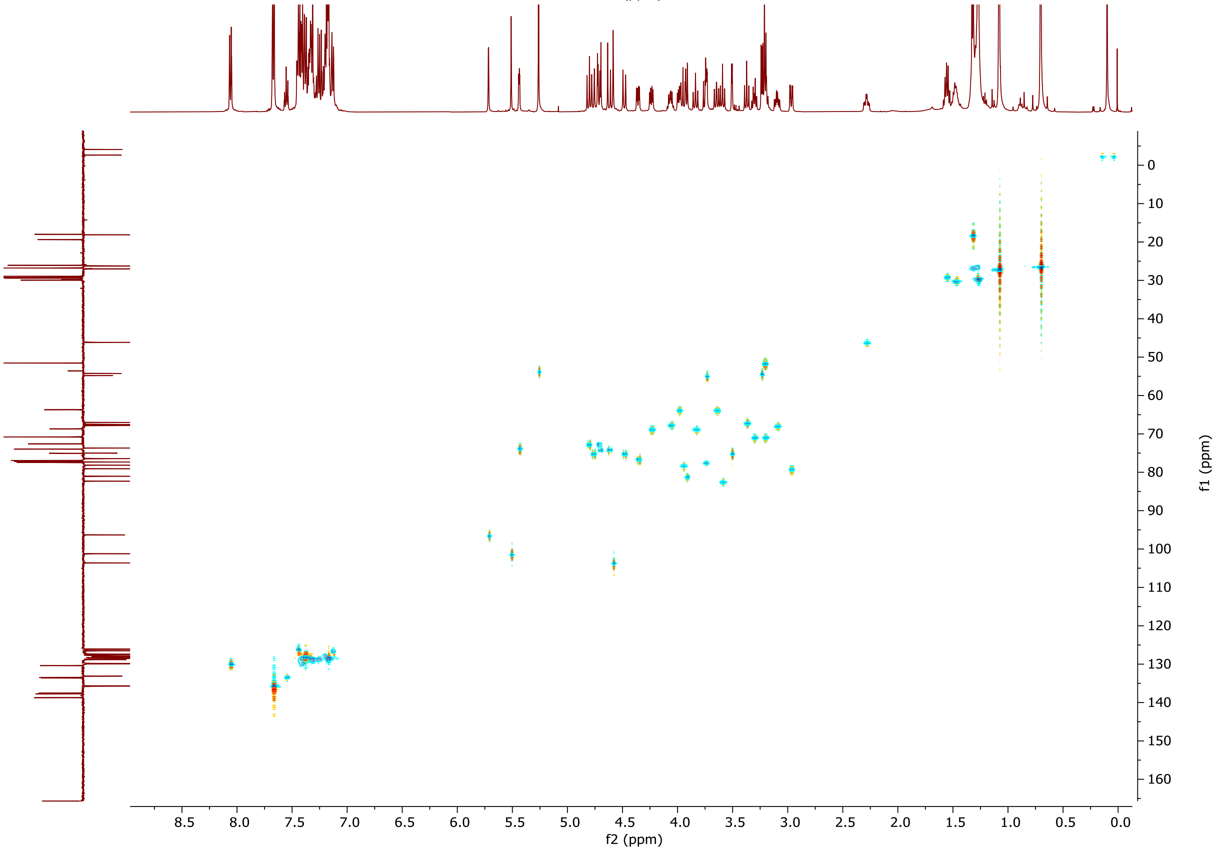
f2 (ppm)
f1 (ppm)

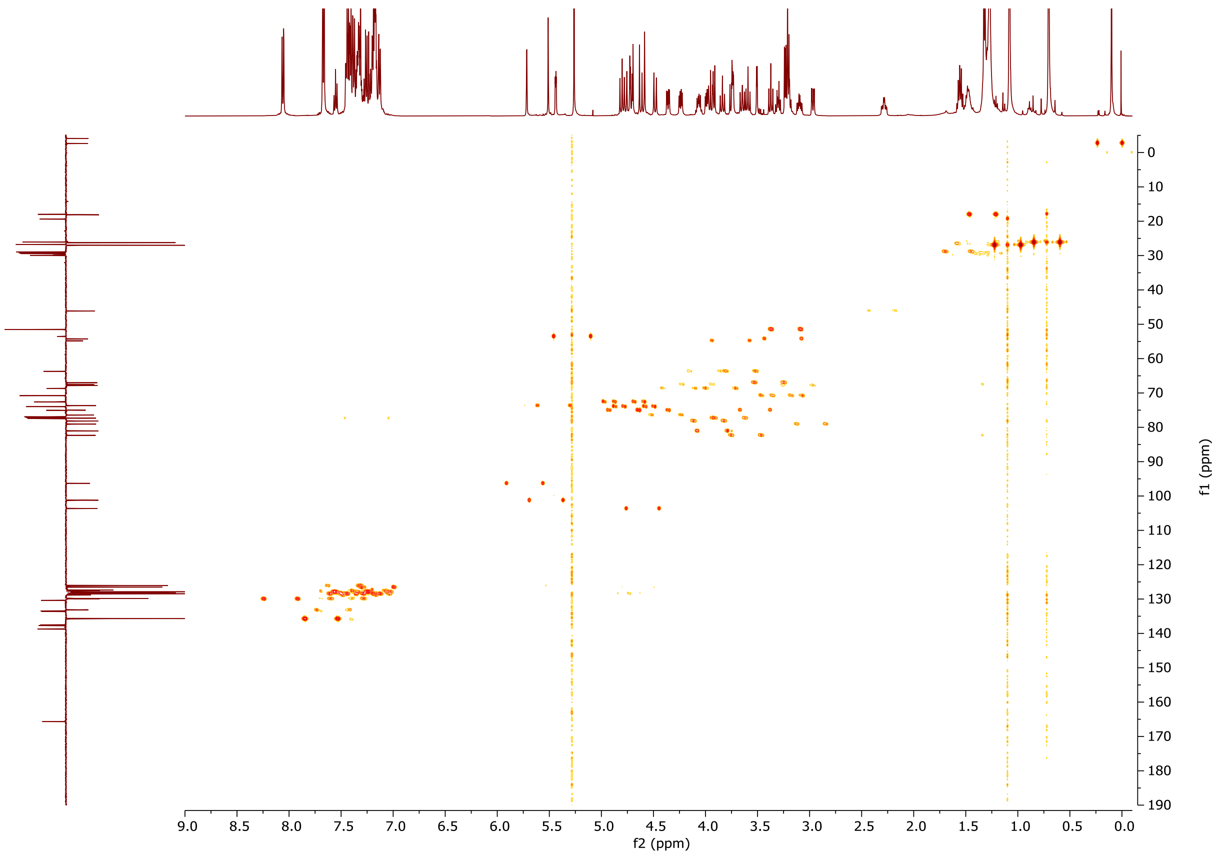

3-*O*-(8-amino-octane)-β-D-mannose-(1->3)-α-L-rhamnose-(1->3)-β-glucose-cyclophellitol (13)

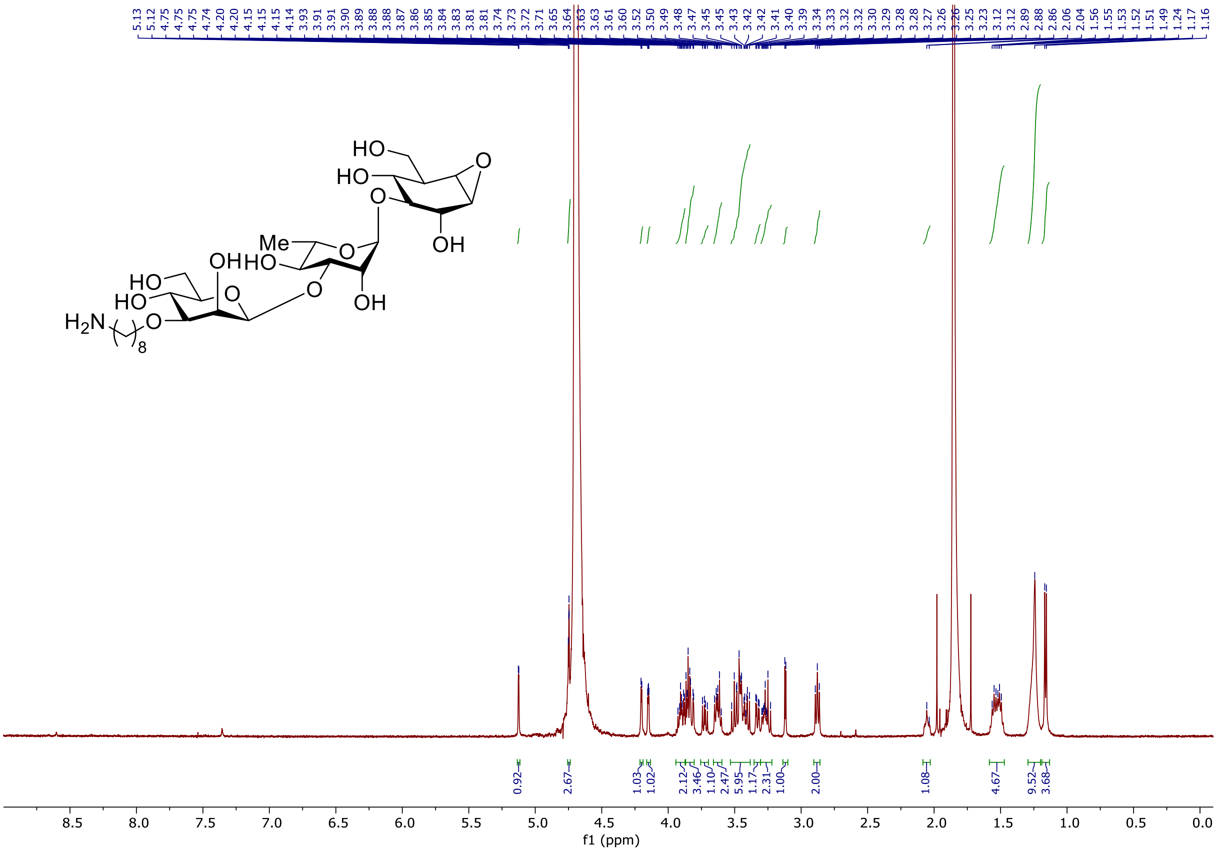

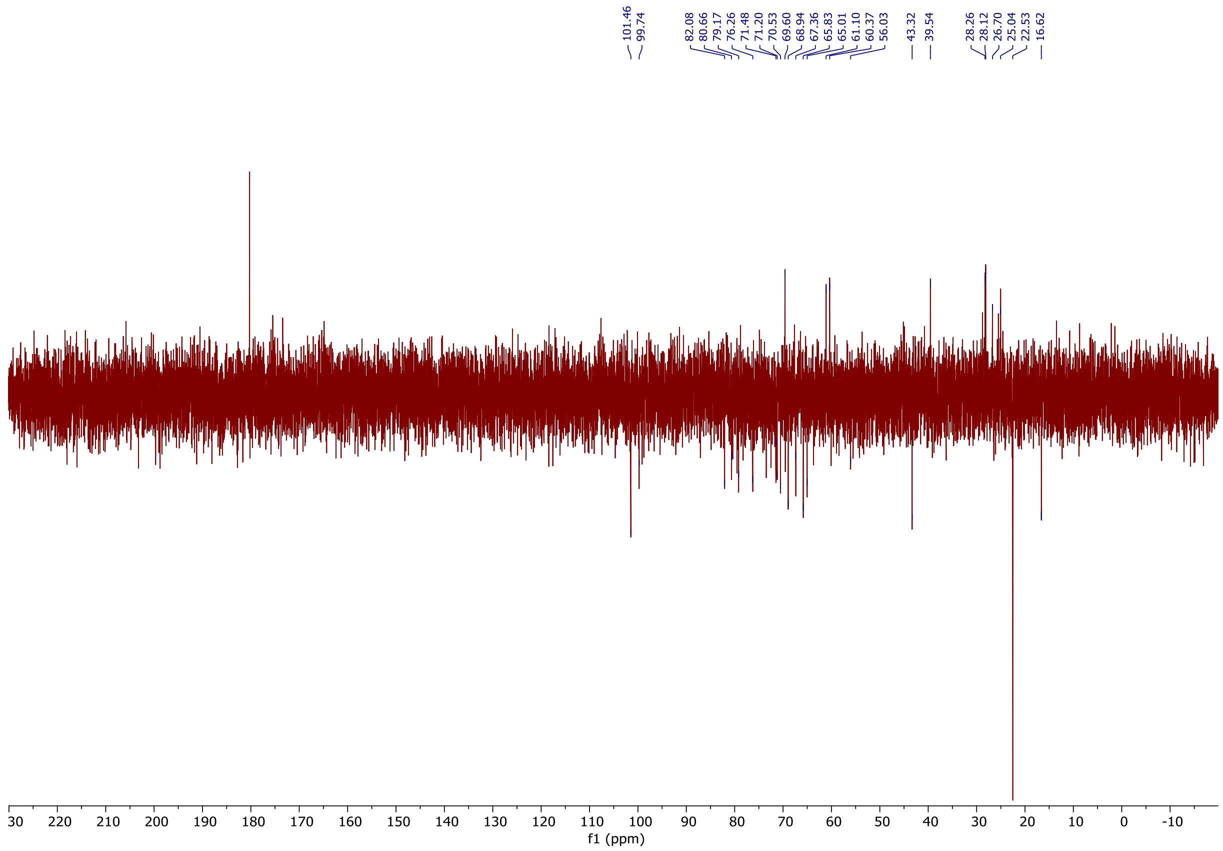
101.46
99.74
82.08
80.66
79.17
76.26
71.48
71.20
70.53
69.60
68.94
67.36
65.83
65.01
61.10
60.37
56.03
43.32
39.54
28.26
28.12
26.70
25.04
22.53
16.62
f1 (ppm)

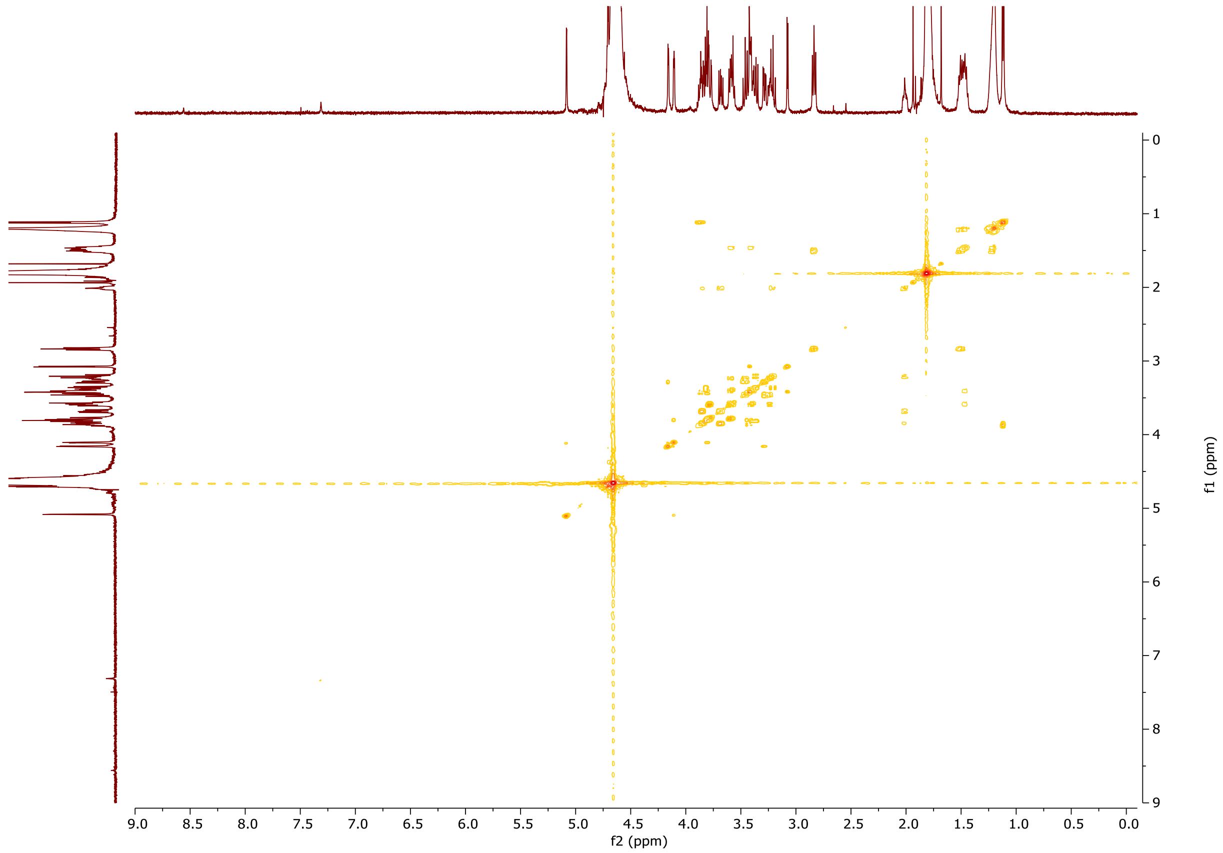
f2 (ppm)
f1 (ppm)

3-*O*-(8-amide Cy5-octane)-β-D-mannose-(1->3)-α-L-rhamnose-(1->3)-β-glucose-cyclophellitol (4)

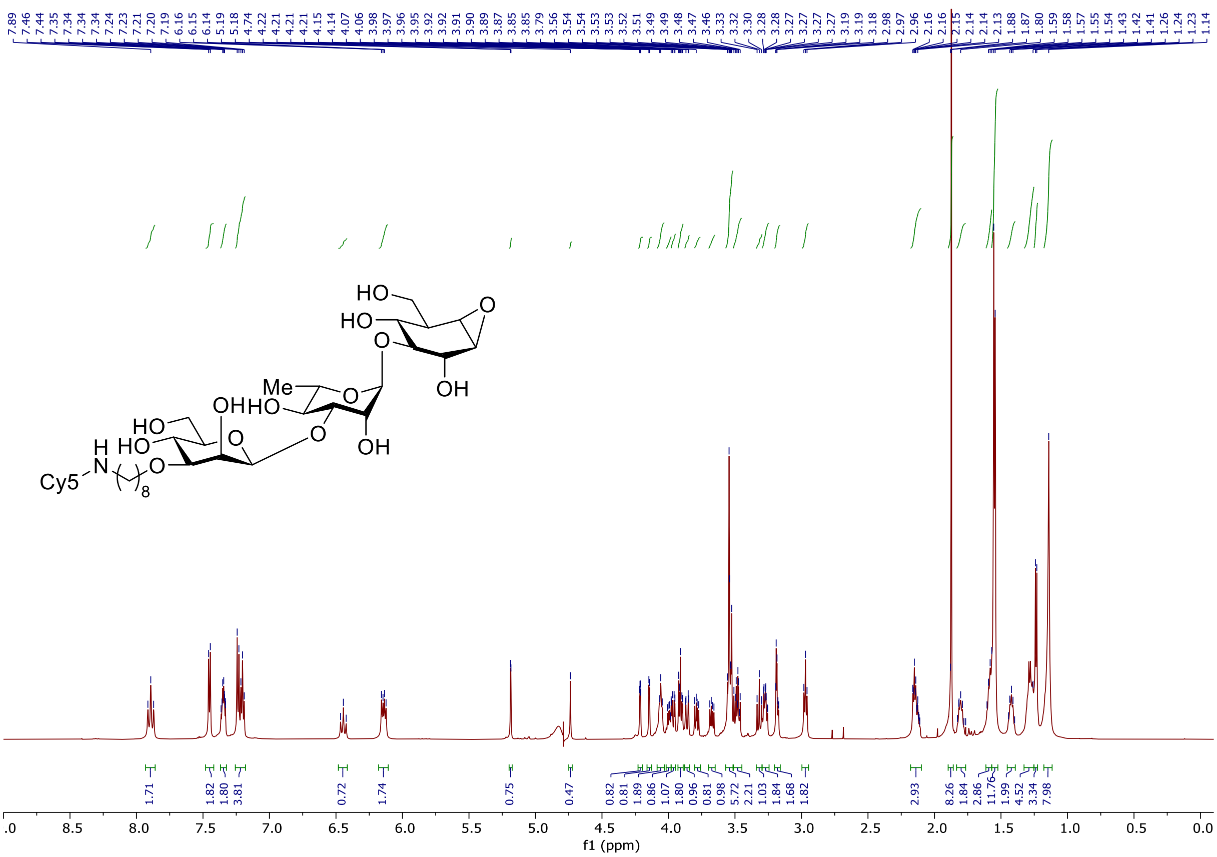

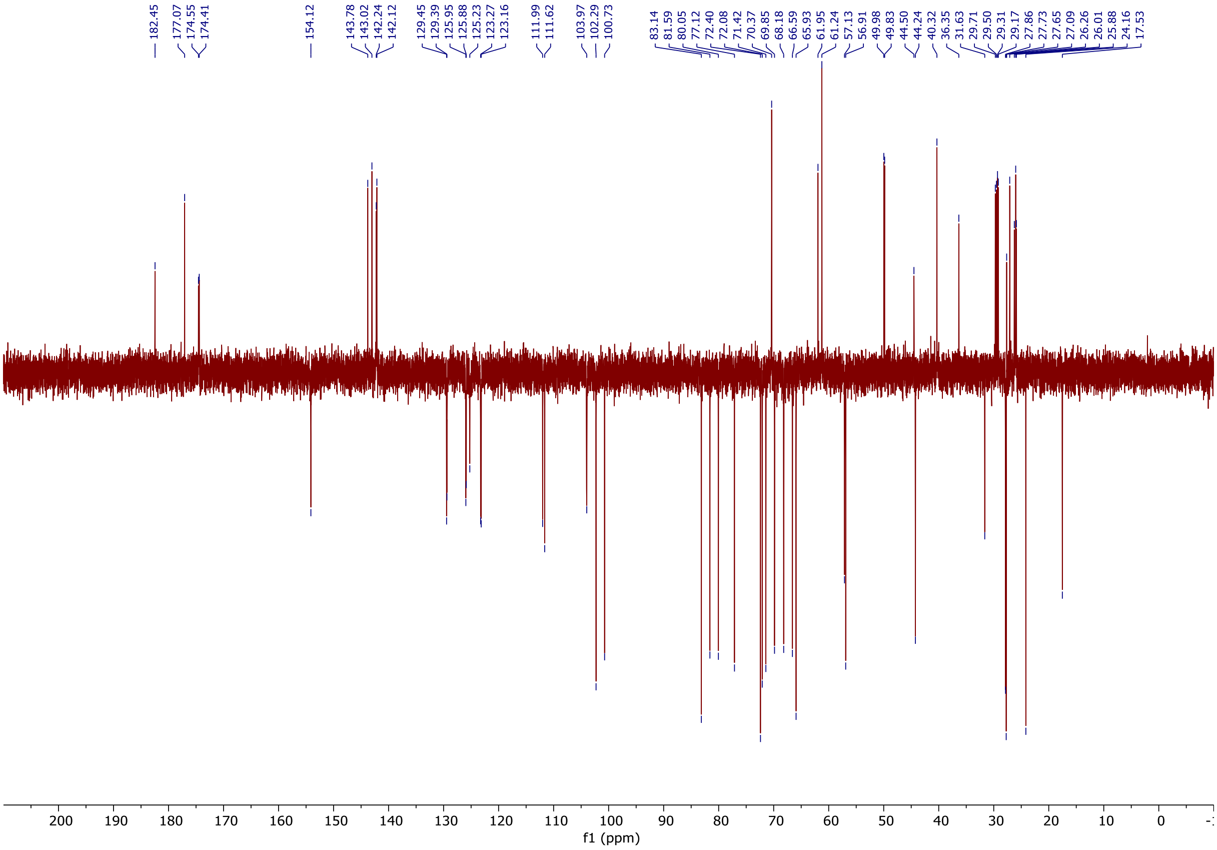

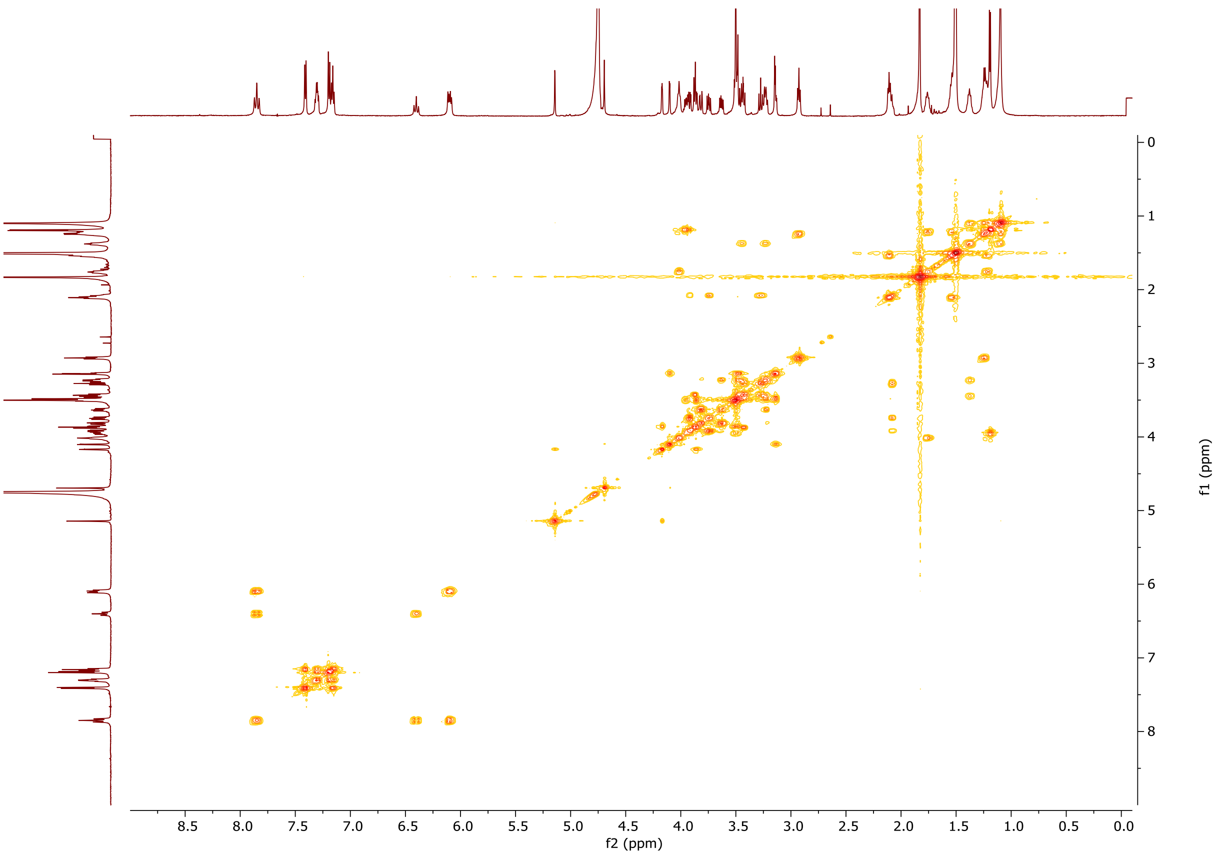
8.5 8.0 7.5 7.0 6.5 6.0 5.5 5.0 4.5 4.0 3.5 3.0 2.5 2.0 1.5 1.0 0.5 0.0
f2 (ppm)
0 1 2 3 4 5 6 7 8
f1 (ppm)

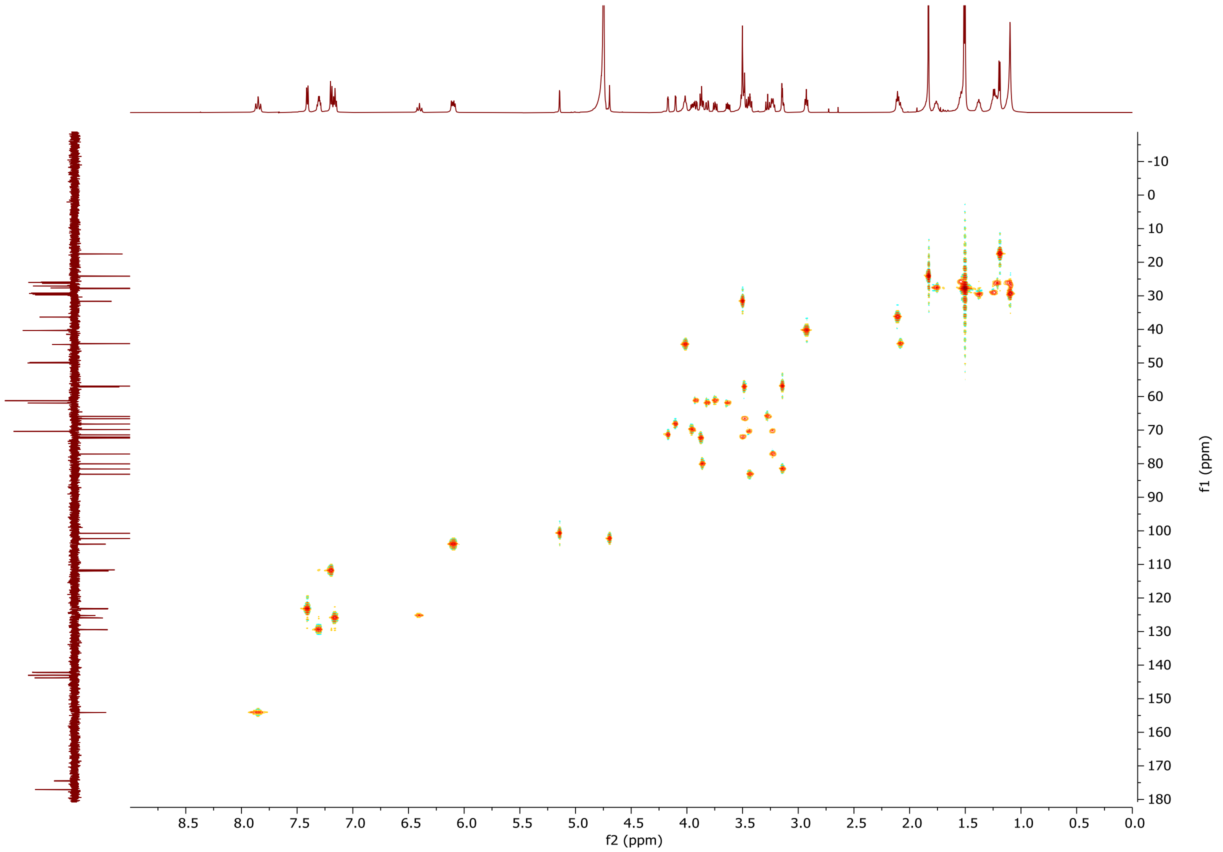
8.5 8.0 7.5 7.0 6.5 6.0 5.5 5.0 4.5 4.0 3.5 3.0 2.5 2.0 1.5 1.0 0.5 0.0
f2 (ppm)
-10 0 10 20 30 40 50 60 70 80 90 100 110 120 130 140 150 160 170 180
f1 (ppm)

1,2,4,6-tetra-*O*-benzoyl-3-*O*-naphthyl-β-D-glucopyranose (77)

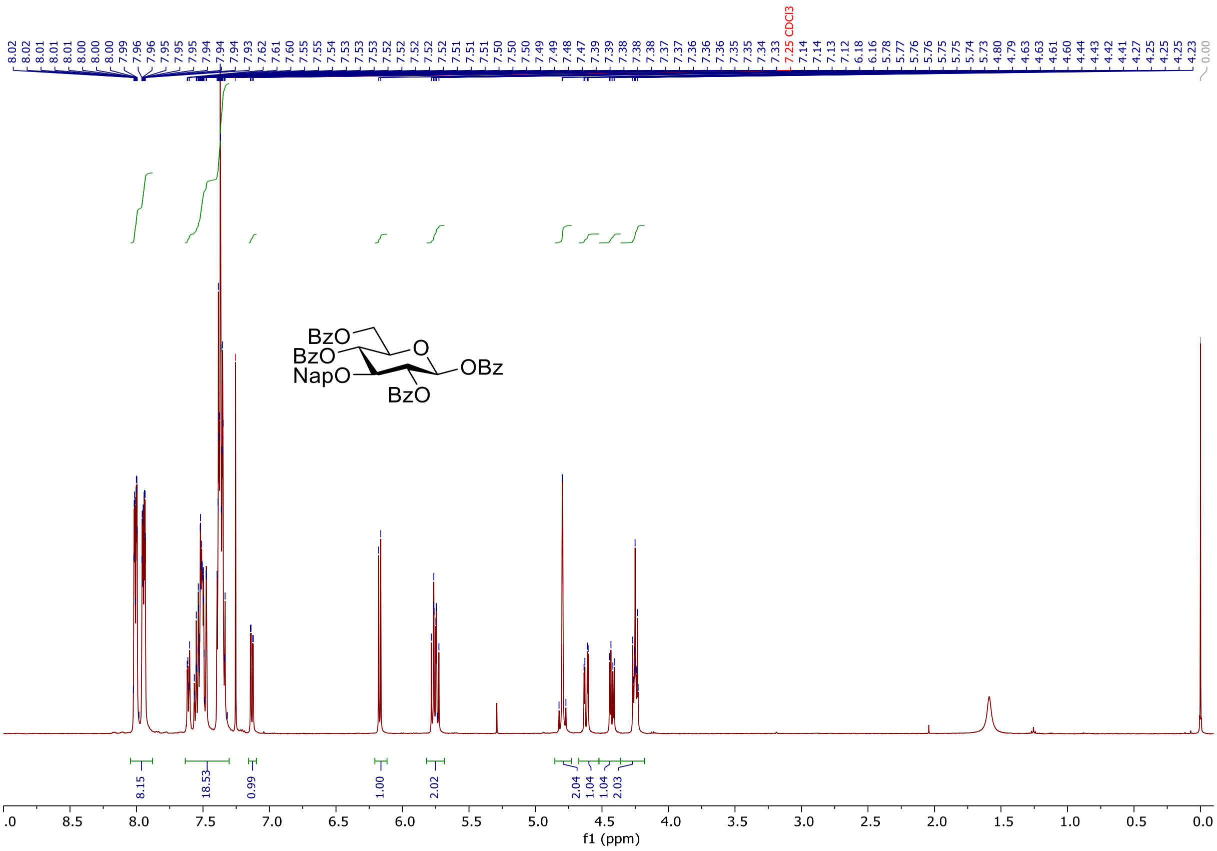

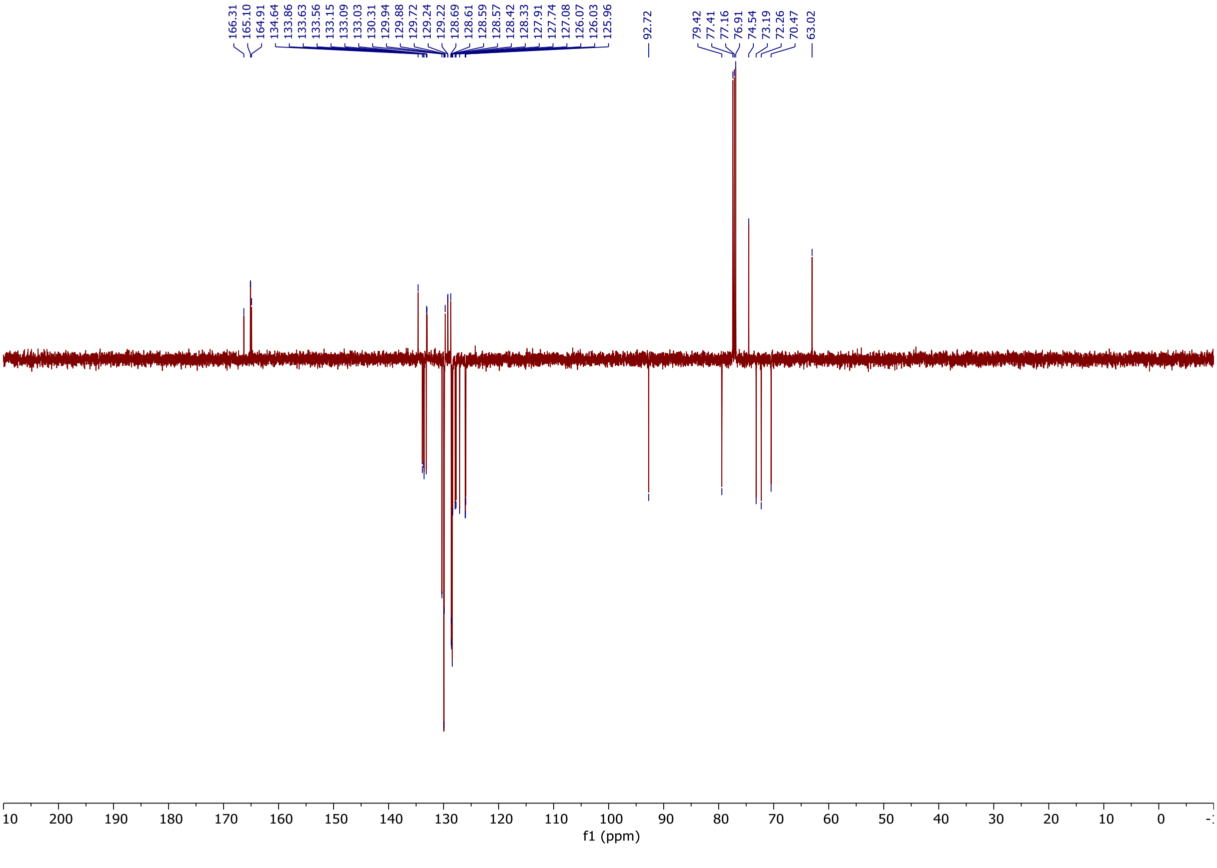

2,4,6-tri-*O*-benzoyl-3-*O*-naphthyl-D-glucopyranose (78)

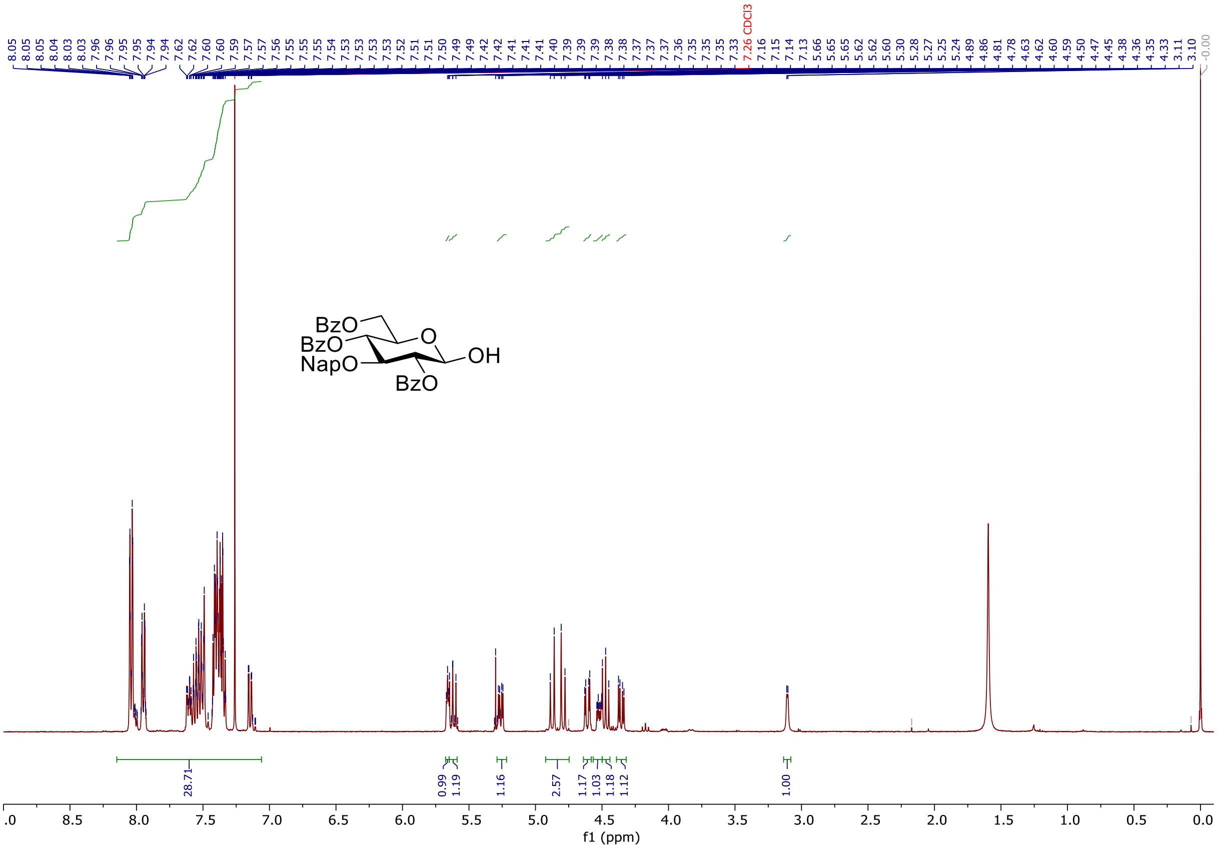

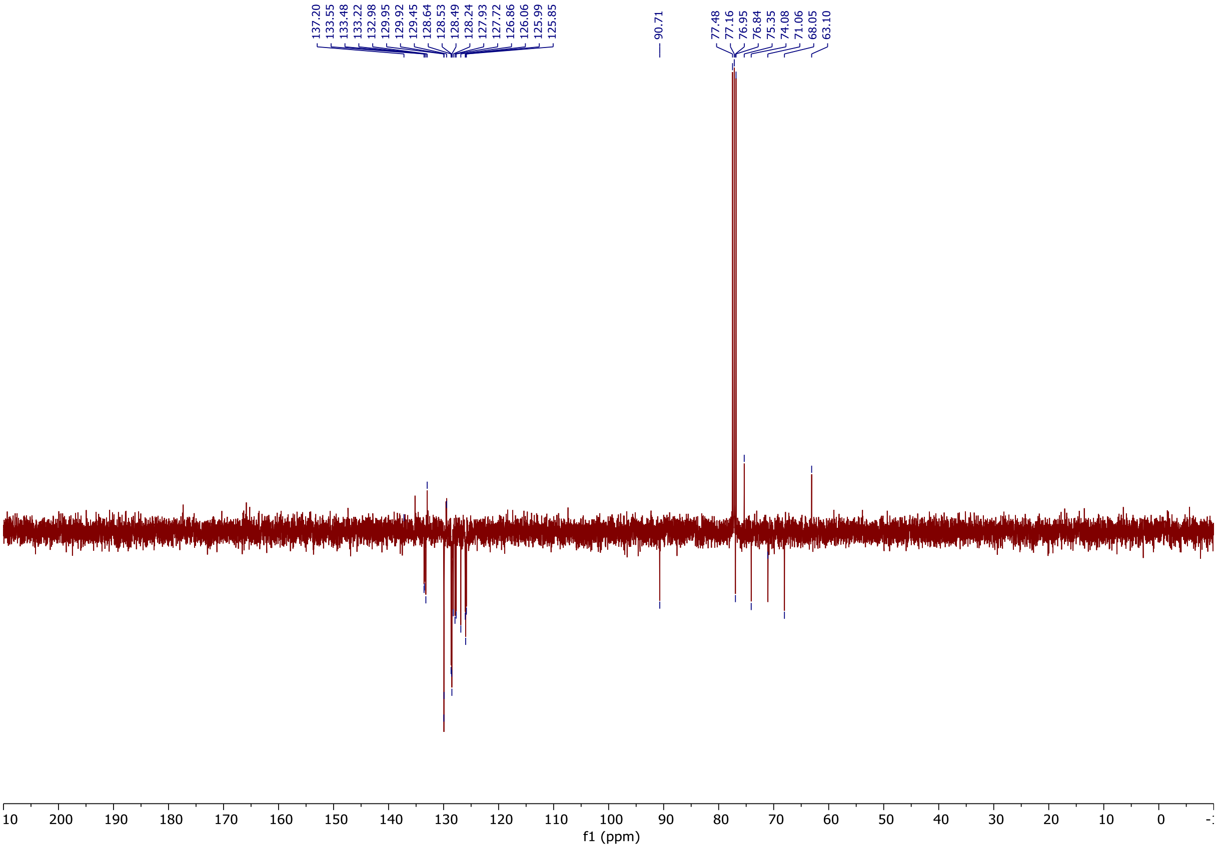

2,4,6-tri-*O*-benzoyl-3-*O*-naphthyl-1-*O*-trifluoro-N-phenyl-imidate-D-glucopyranose (79)

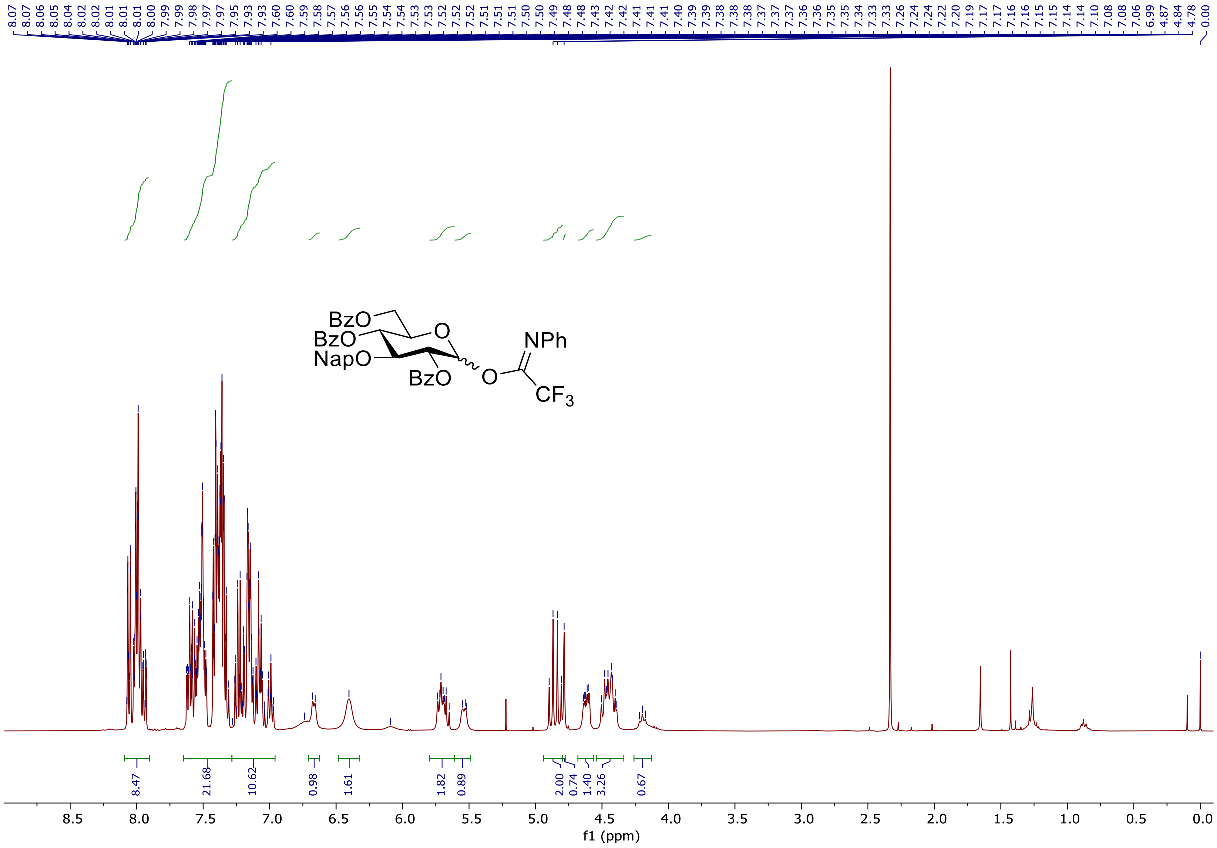

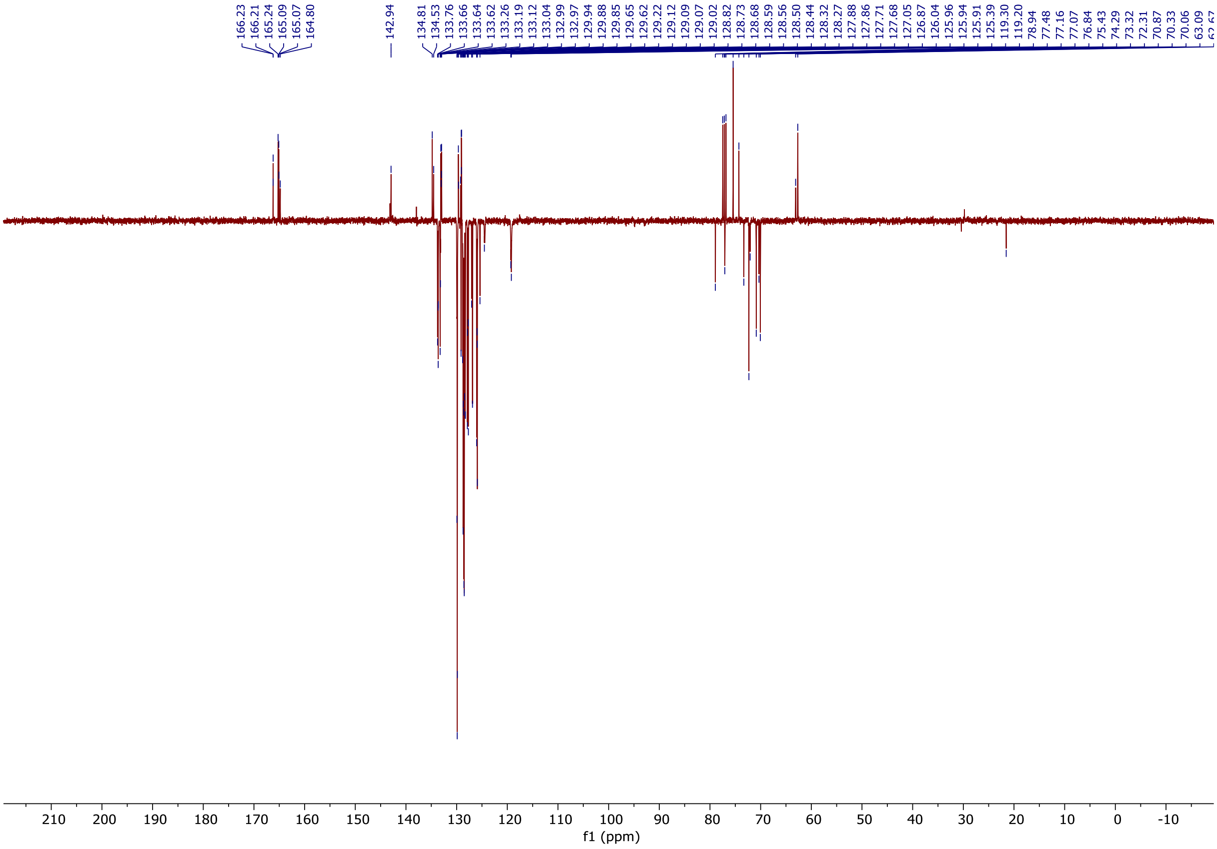

2-*O*-benzyl-4,6-*O*-benzylidene-3-*O*-naphthyl-β-D-mannose-(1->3)-1-*O*-allyl-2-*O*-benzoyl-4-*O*-benzyl-α-L-rhamnose (81)

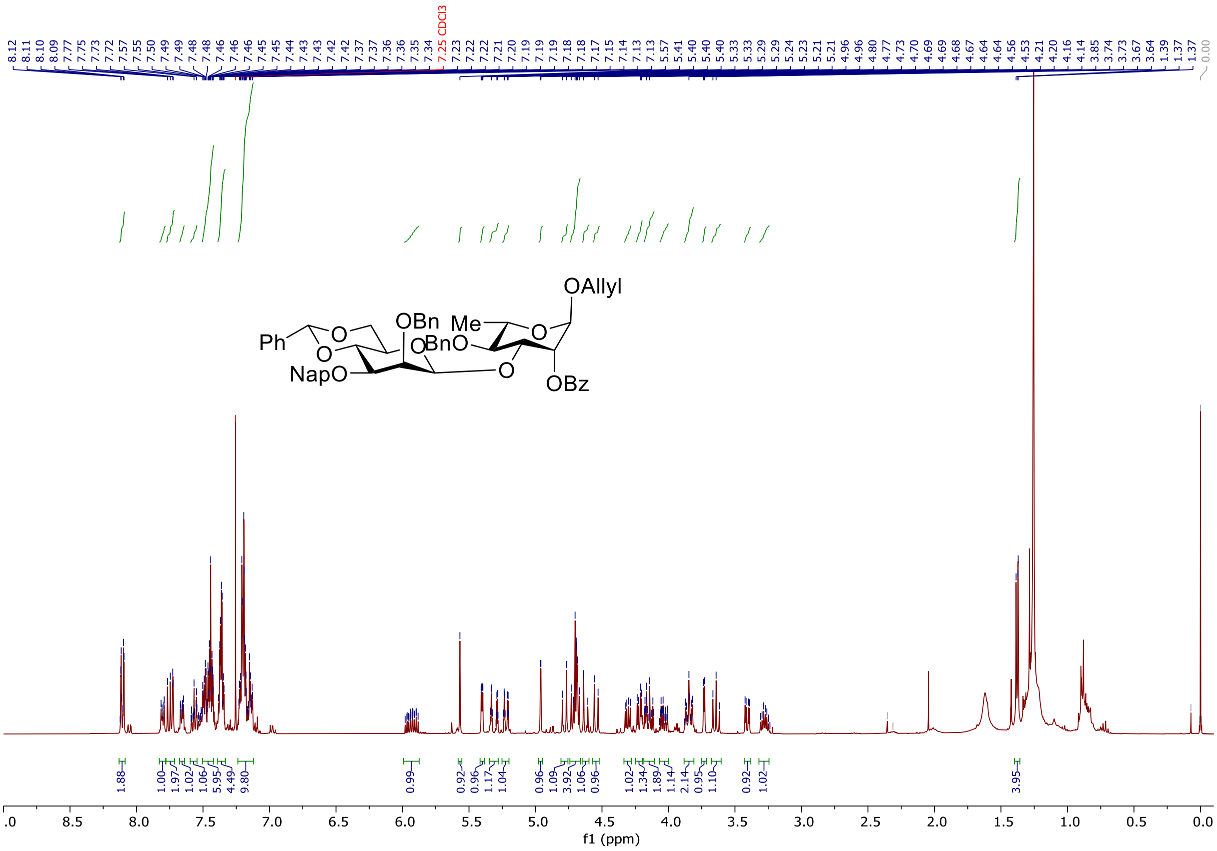

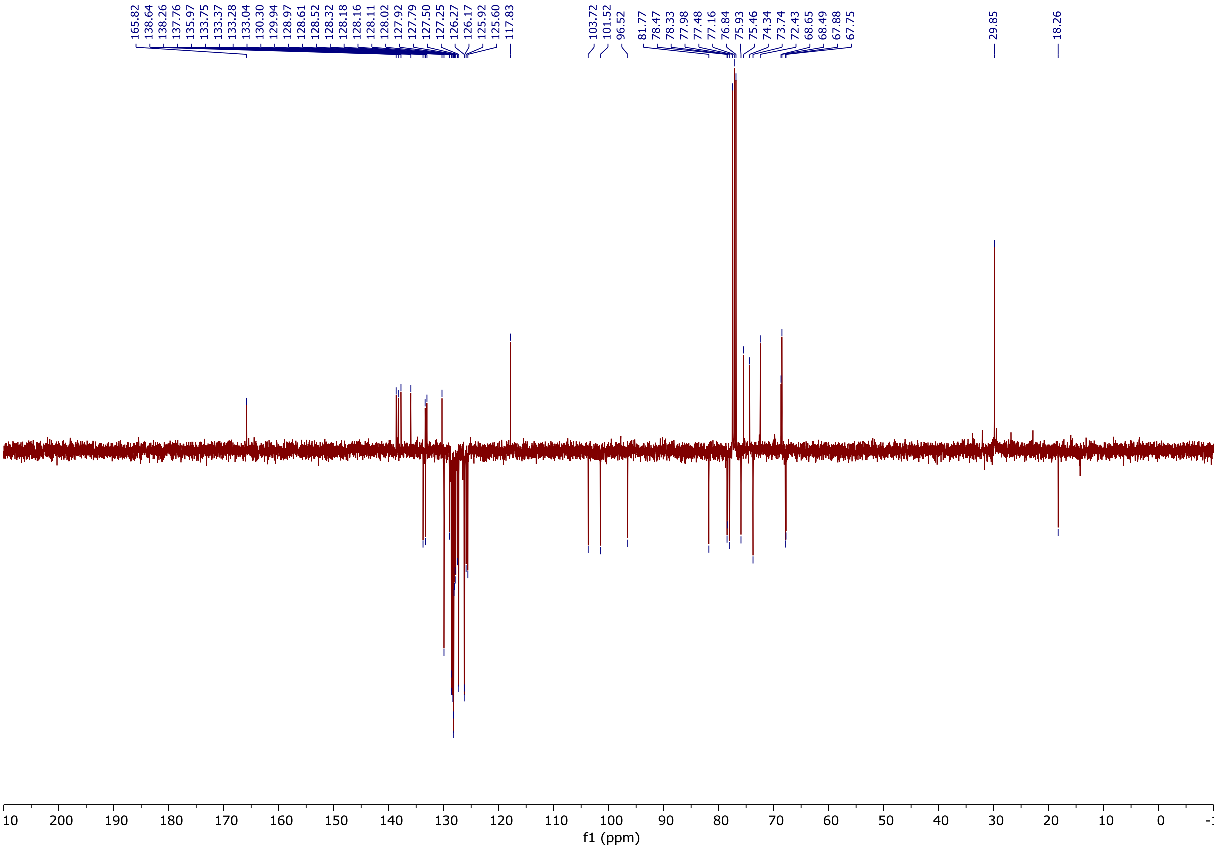

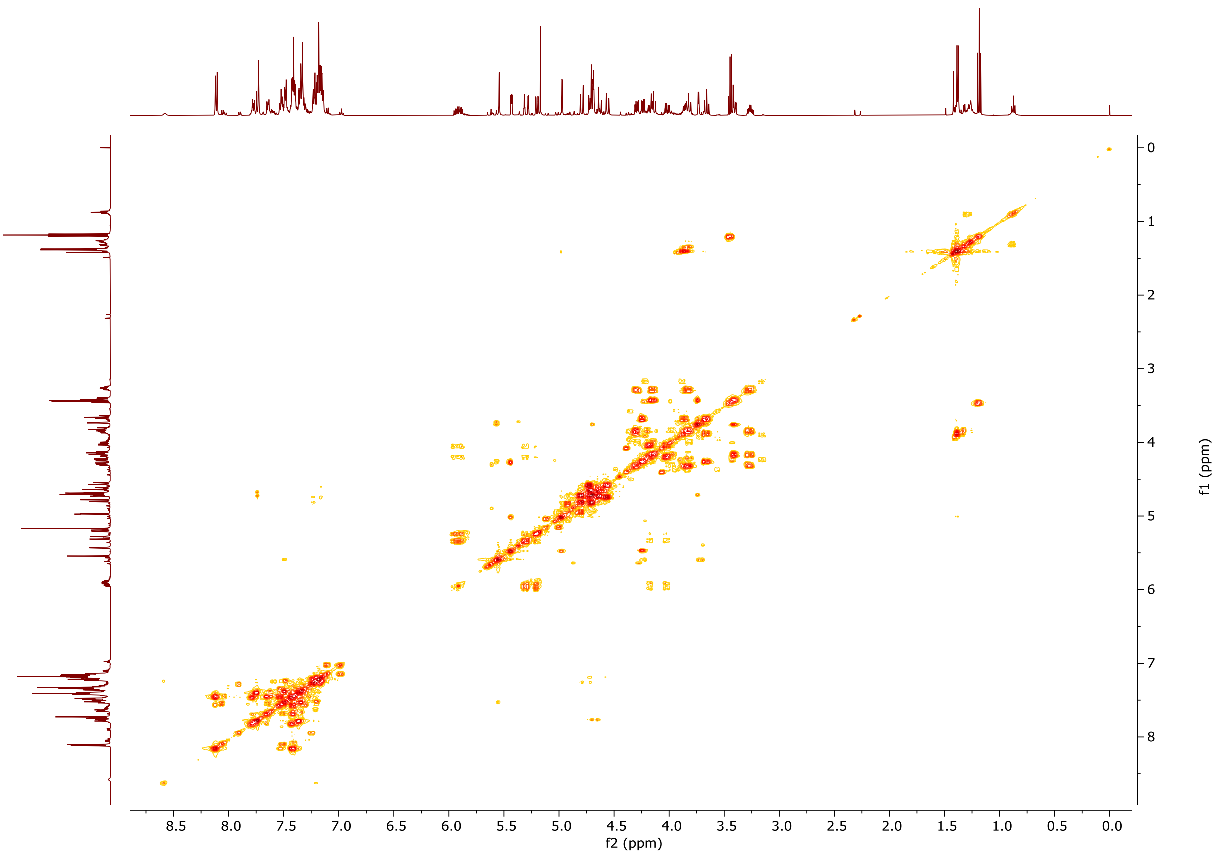
f2 (ppm)
f1 (ppm)

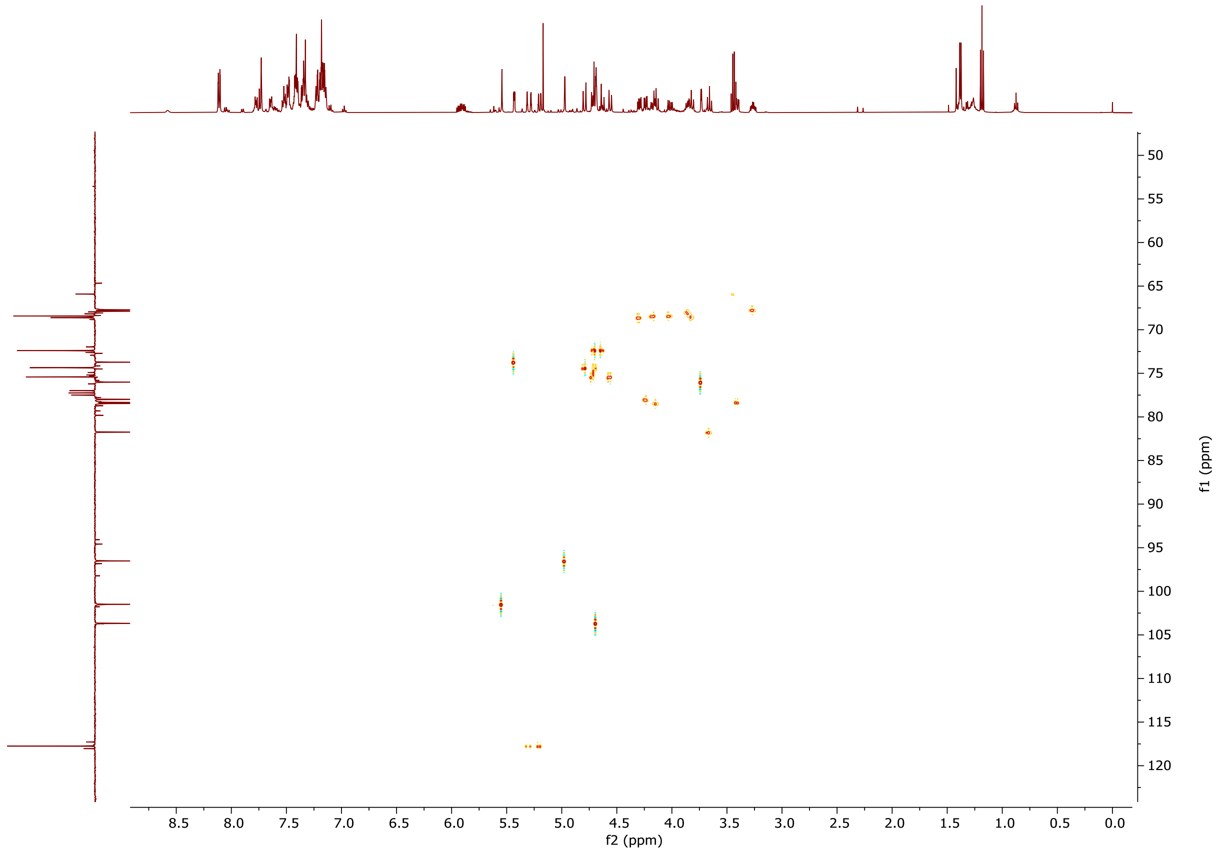
f2 (ppm)
f1 (ppm)

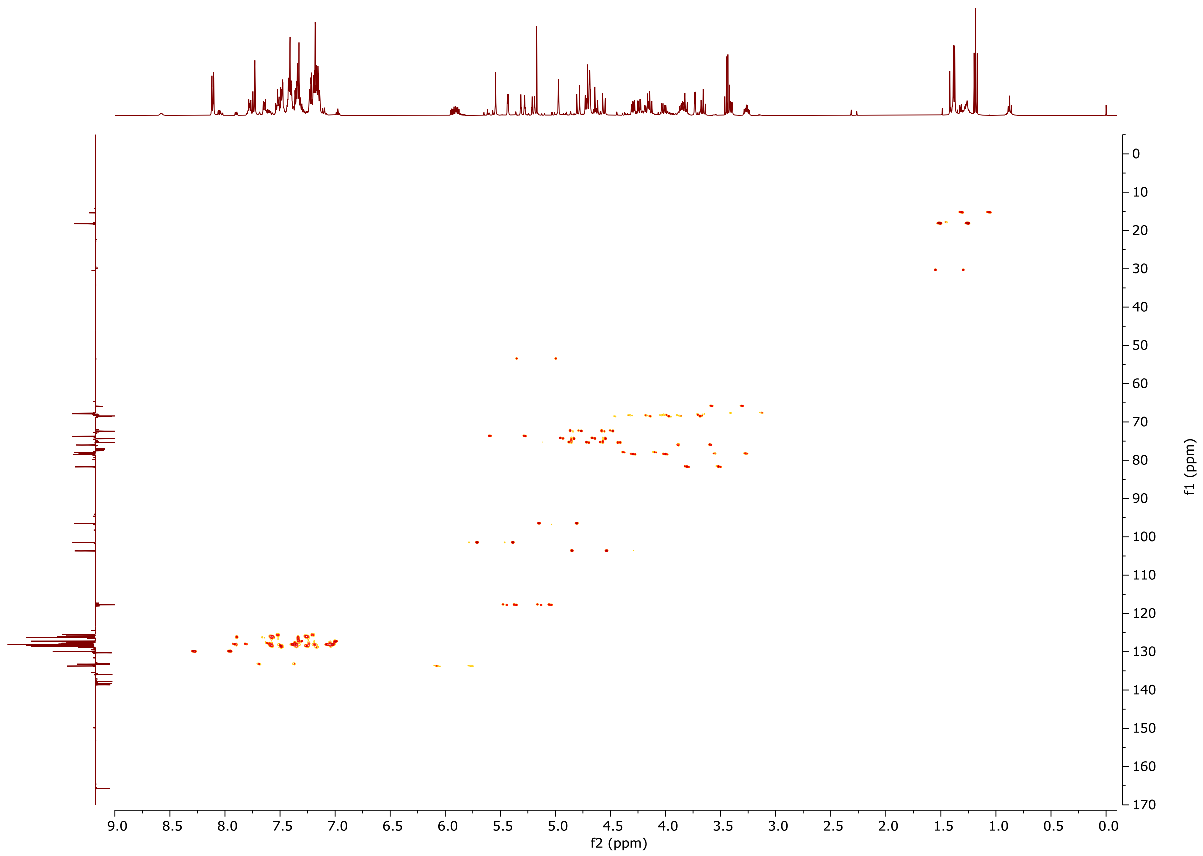

2-*O*-benzyl-4,6-*O*-benzylidene-β-D-mannose-(1->3)-1-*O*-allyl-2-*O*-benzoyl-4-*O*-benzyl-α-L-rhamnose (82)

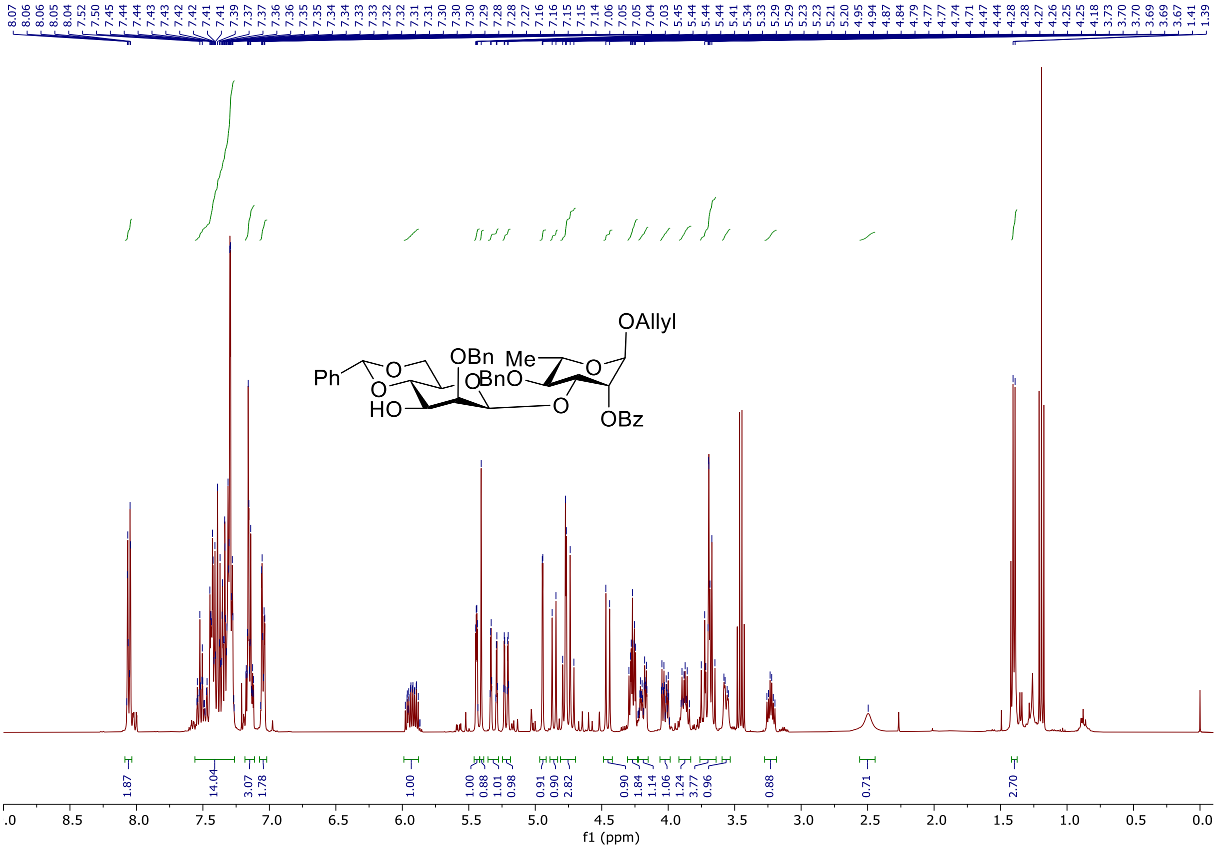

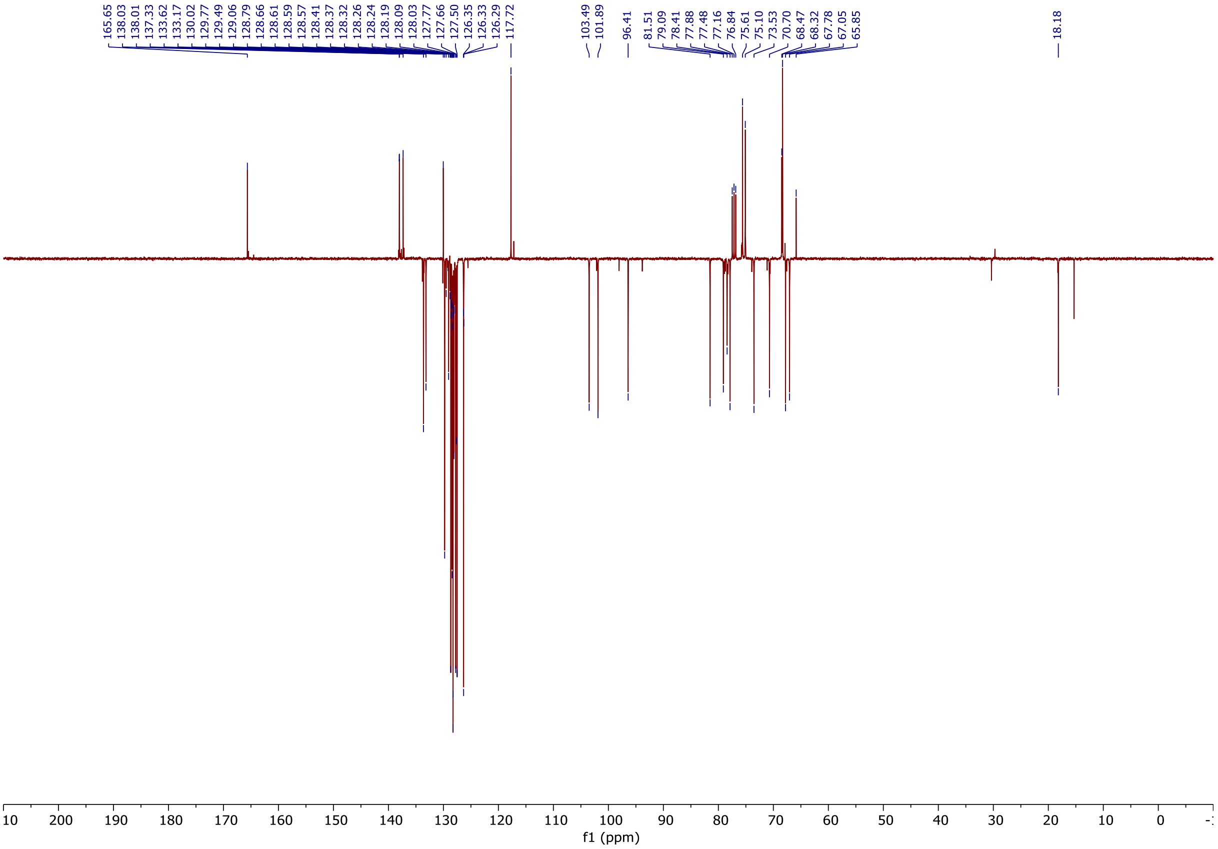
165.65
138.03
138.01
137.33
133.62
133.17
130.02
129.77
129.49
129.06
128.79
128.66
128.61
128.59
128.57
128.41
128.37
128.32
128.26
128.24
128.19
128.09
128.03
127.77
127.66
127.50
126.35
126.33
126.29
117.72
103.49
101.89
96.41
81.51
79.09
78.41
77.88
77.48
77.16
76.84
75.61
75.10
73.53
70.70
68.47
68.32
67.78
67.05
65.85
18.18
f1 (ppm)

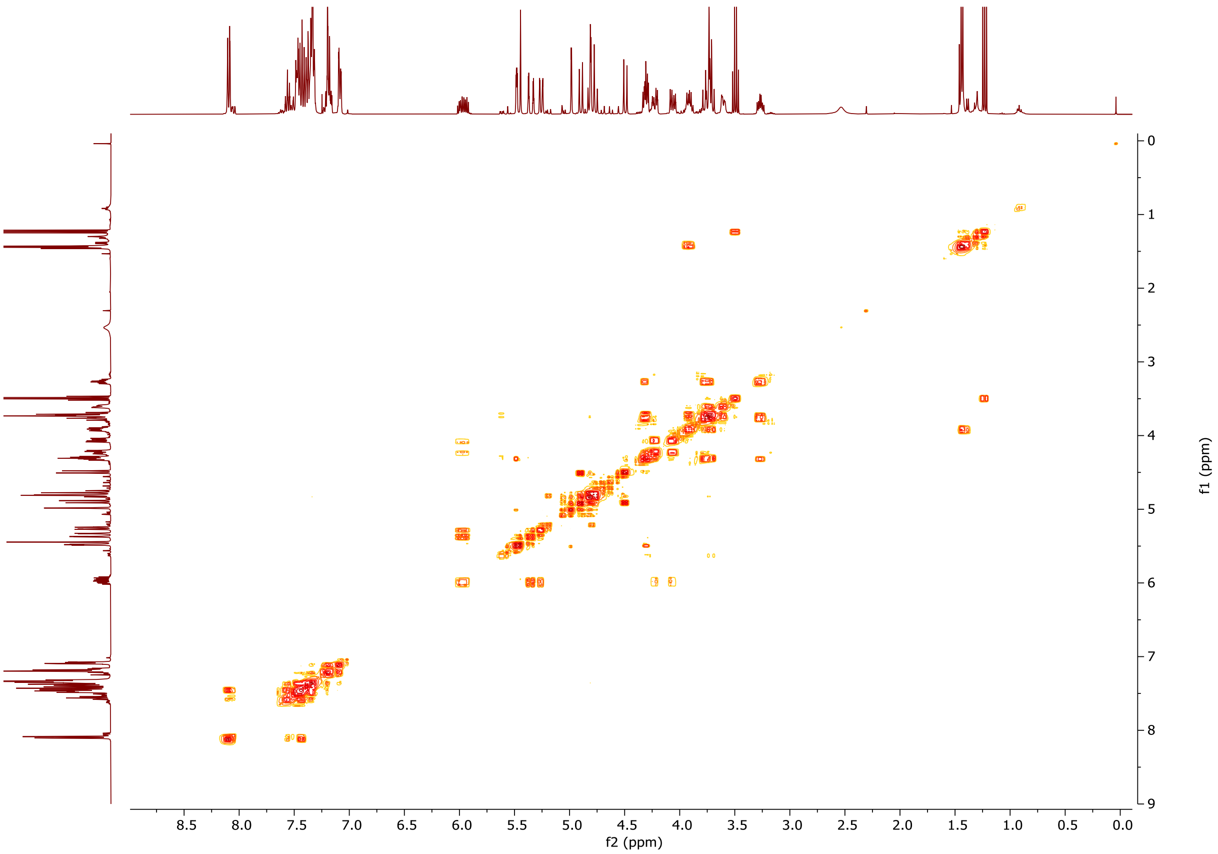
f2 (ppm)
f1 (ppm)

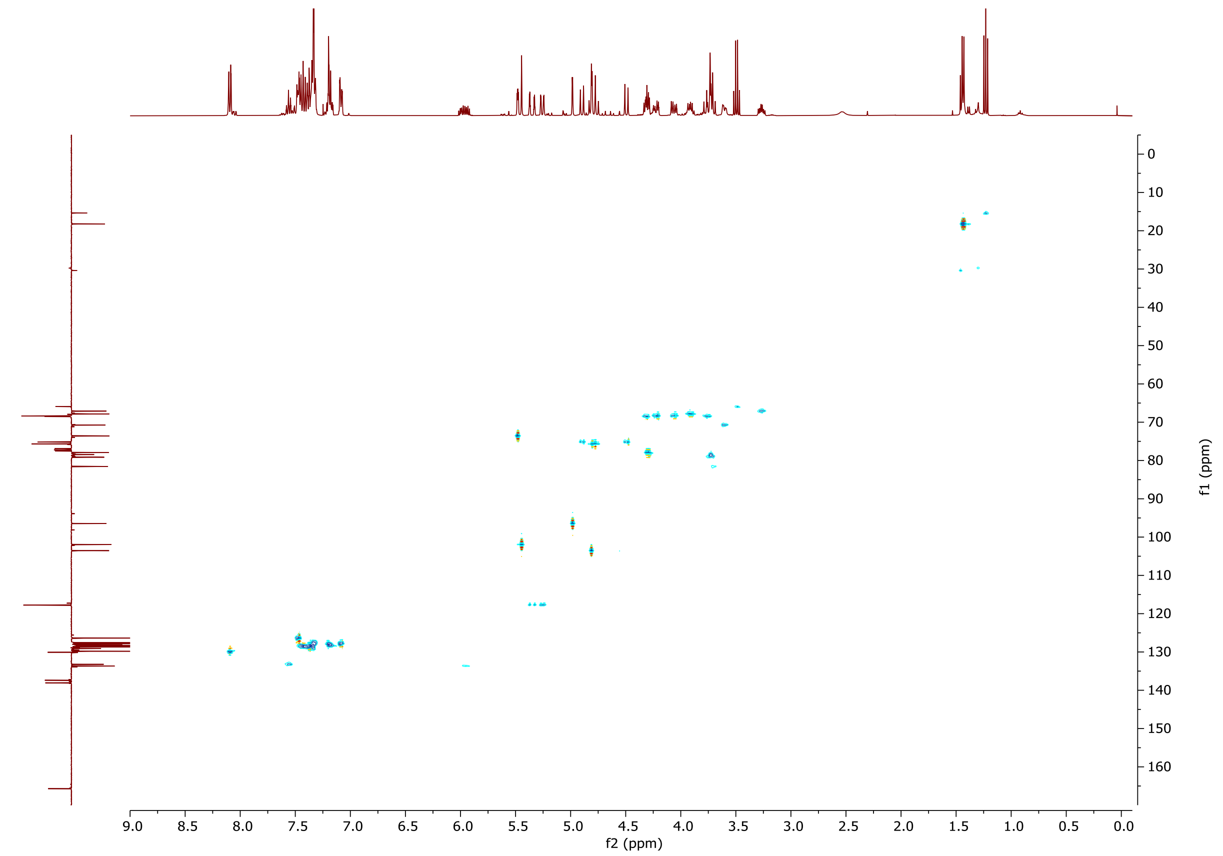

2-*O*-t-butyldimethylsilyl-4,6-*O*-benzylidene-3-*O*-naphthyl-β-D-mannose-(1->3)-2-*O*-benzyl-4,6-*O*-benzylidene-β-D-mannose-(1->3)-1-*O*-allyl-2-*O*-benzoyl-4-*O*-benzyl-α-L-rhamnose (84)

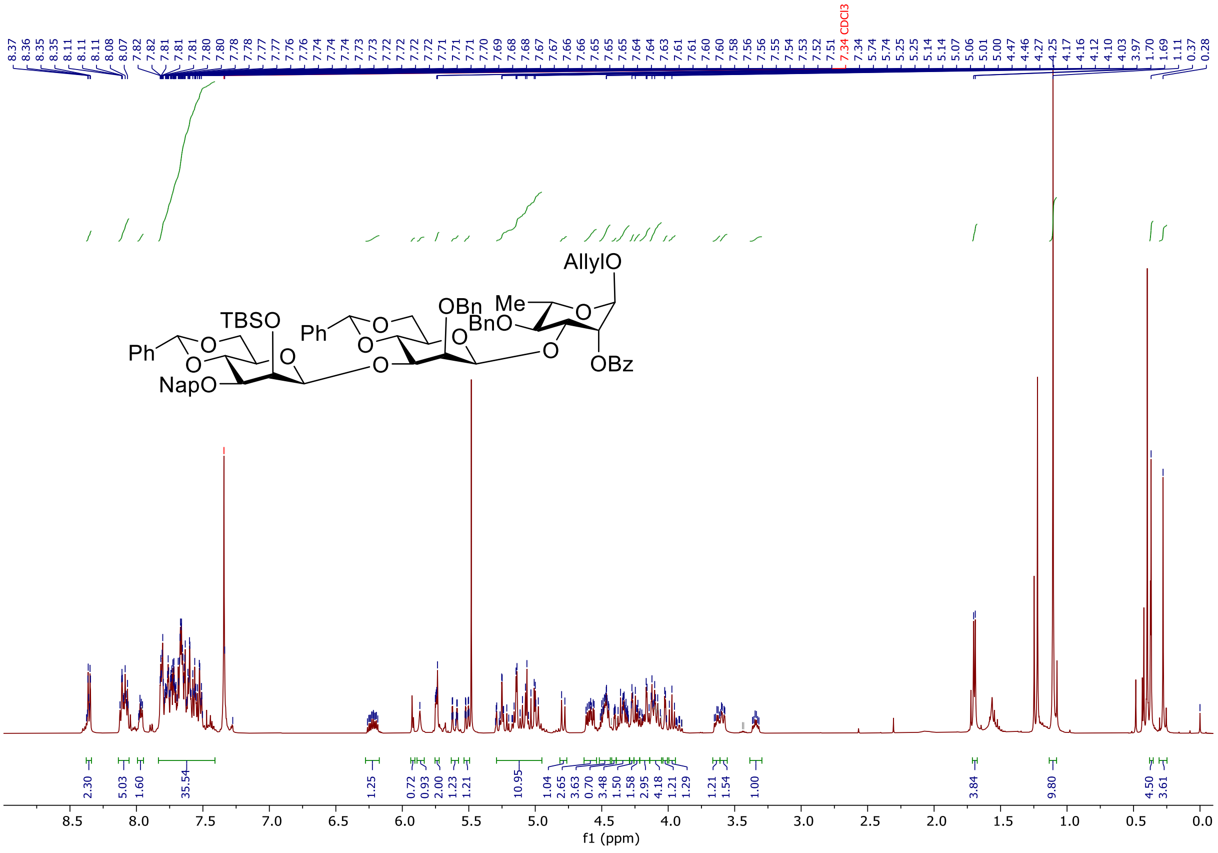

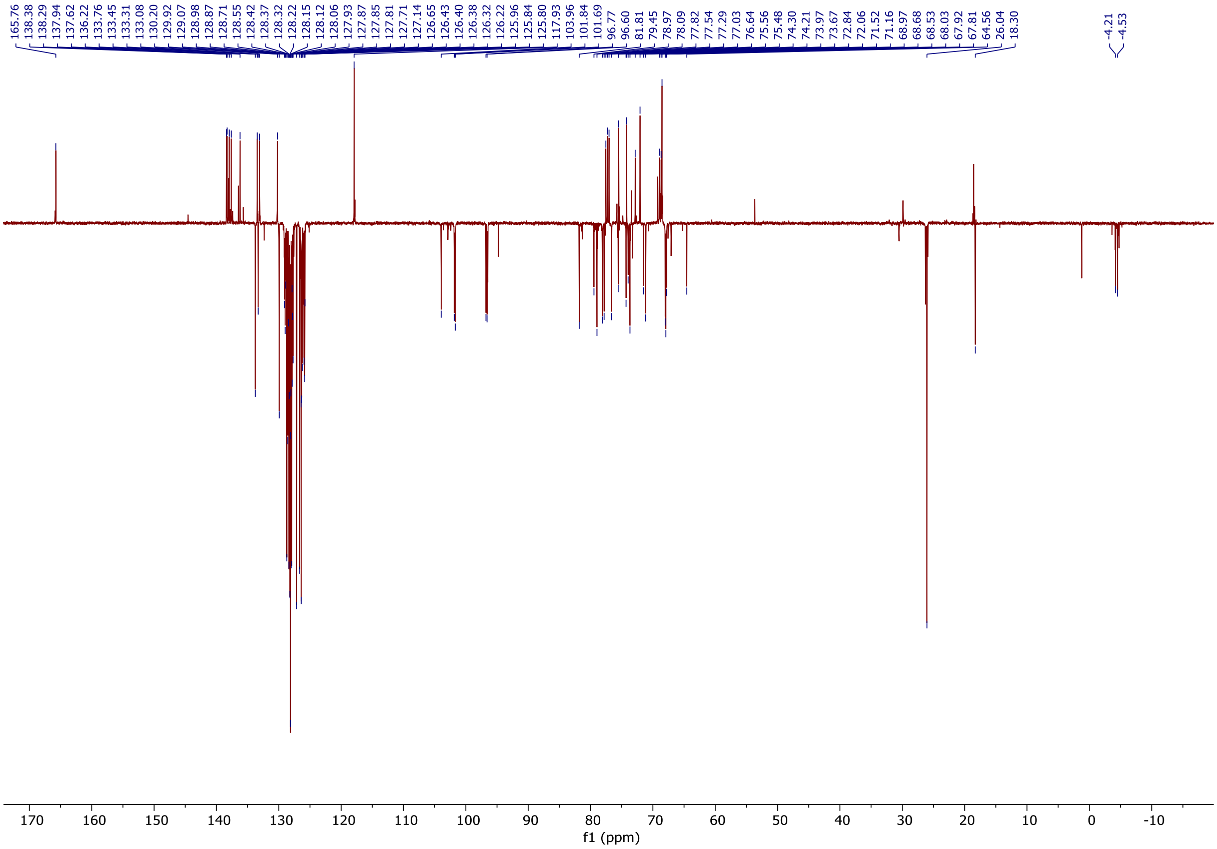
165.76
138.38
138.29
137.94
137.62
136.22
133.76
133.45
133.31
133.08
130.20
129.92
129.07
128.98
128.87
128.71
128.55
128.42
128.37
128.32
128.22
128.15
128.12
128.06
127.93
127.87
127.85
127.81
127.71
127.14
126.65
126.43
126.40
126.38
126.32
126.22
125.96
125.84
125.80
117.93
103.96
101.84
101.69
96.77
96.60
81.81
79.45
78.97
78.09
77.82
77.54
77.29
77.03
76.64
75.56
75.48
74.30
74.21
73.97
73.67
72.84
72.06
71.52
71.16
68.97
68.68
68.53
68.03
67.92
67.81
64.56
26.04
18.30
-4.21
-4.53
f1 (ppm)

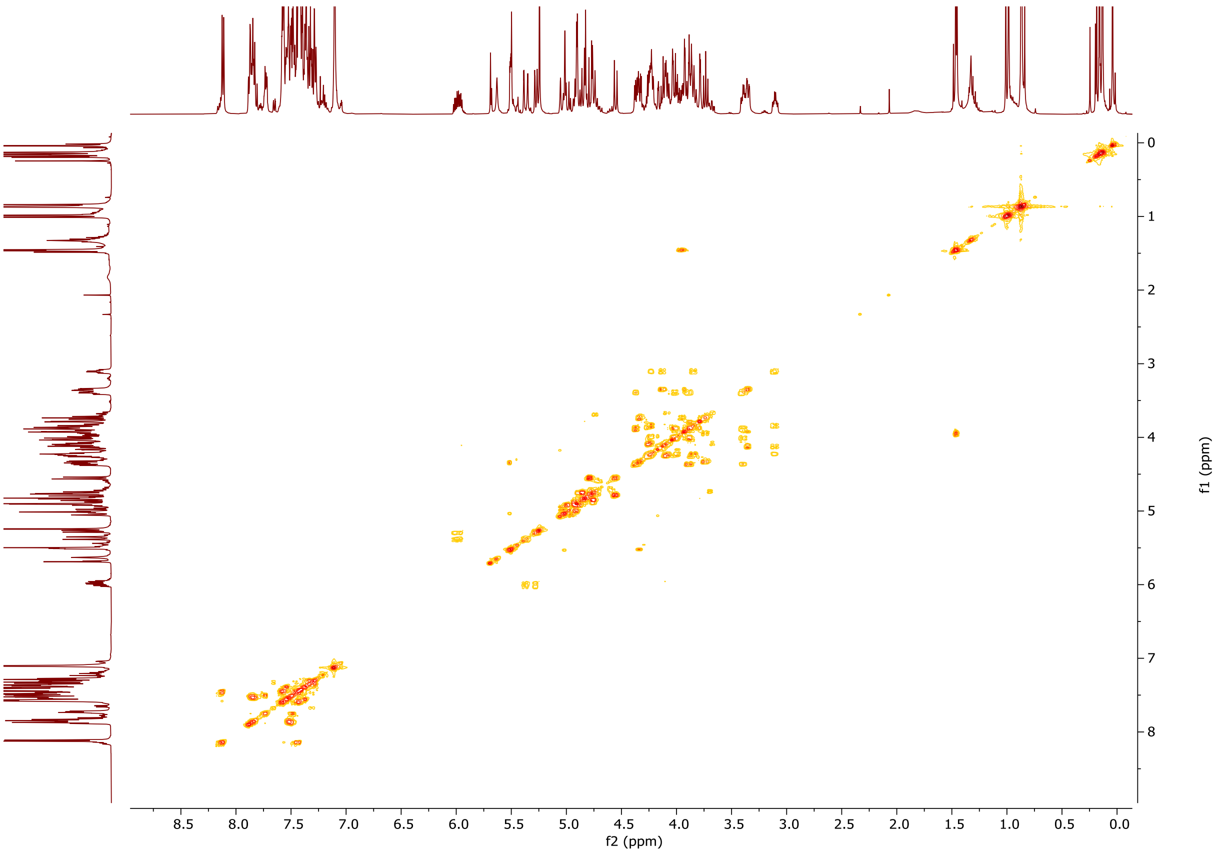
f2 (ppm)
f1 (ppm)

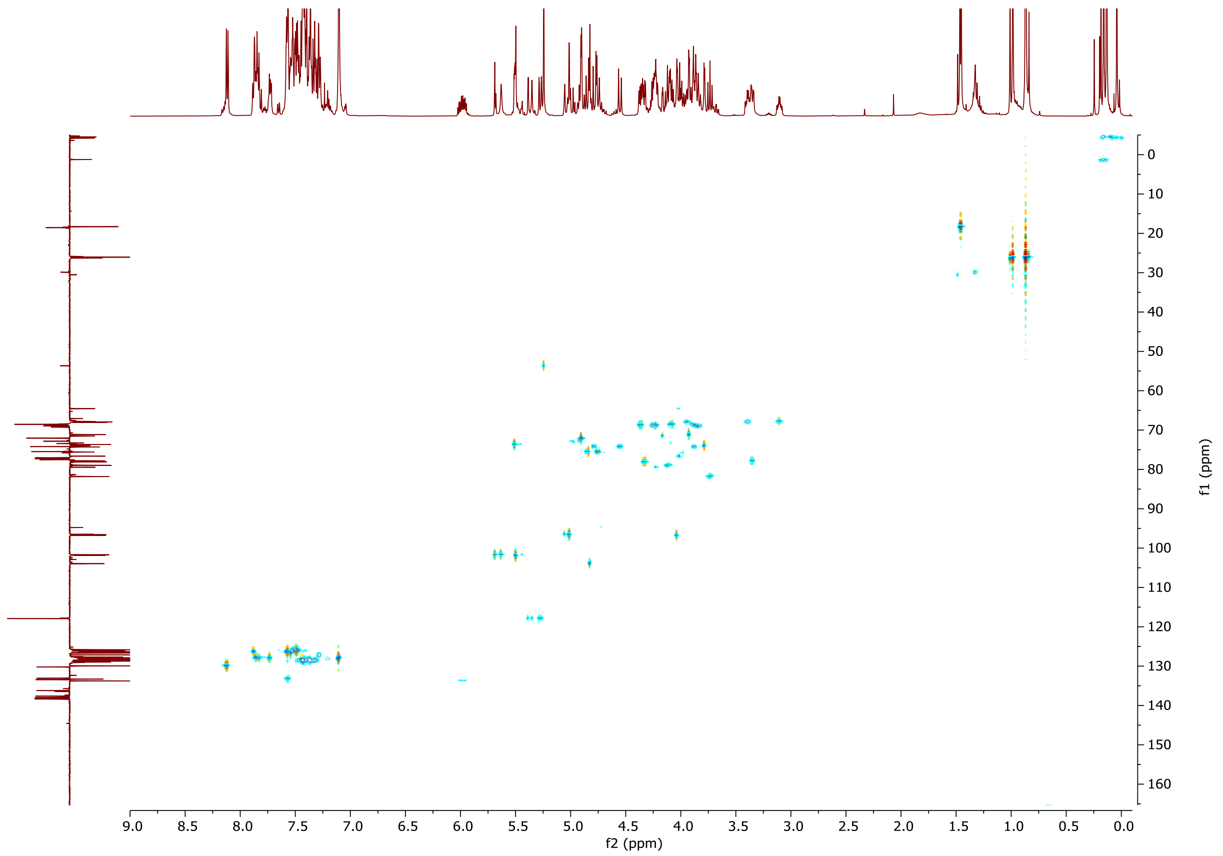

4,6-*O*-benzylidene-3-*O*-naphthyl-β-D-mannose-(1->3)-2-*O*-benzyl-4,6-*O*-benzylidene-β-D-mannose-(1->3)-1-*O*-allyl-2-*O*-benzoyl-4-*O*-benzyl-α-L-rhamnose (85)

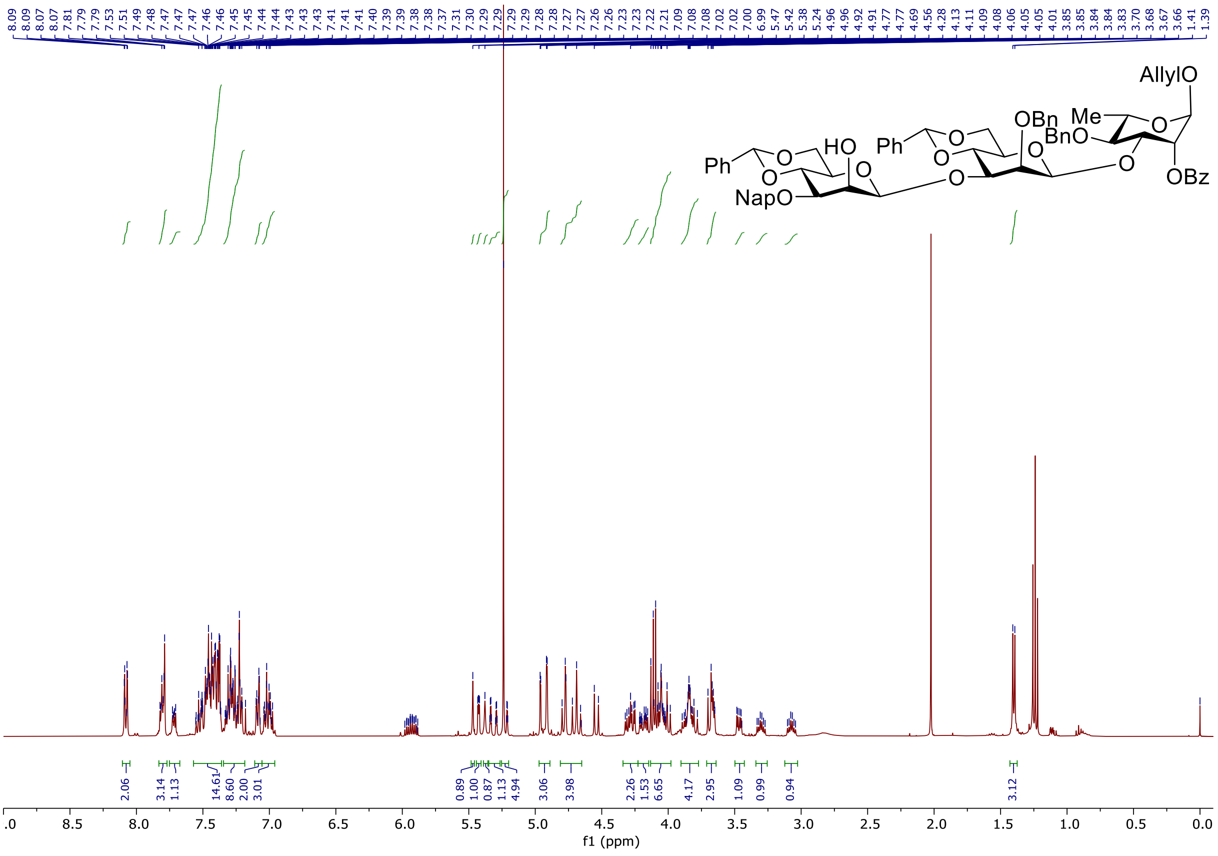

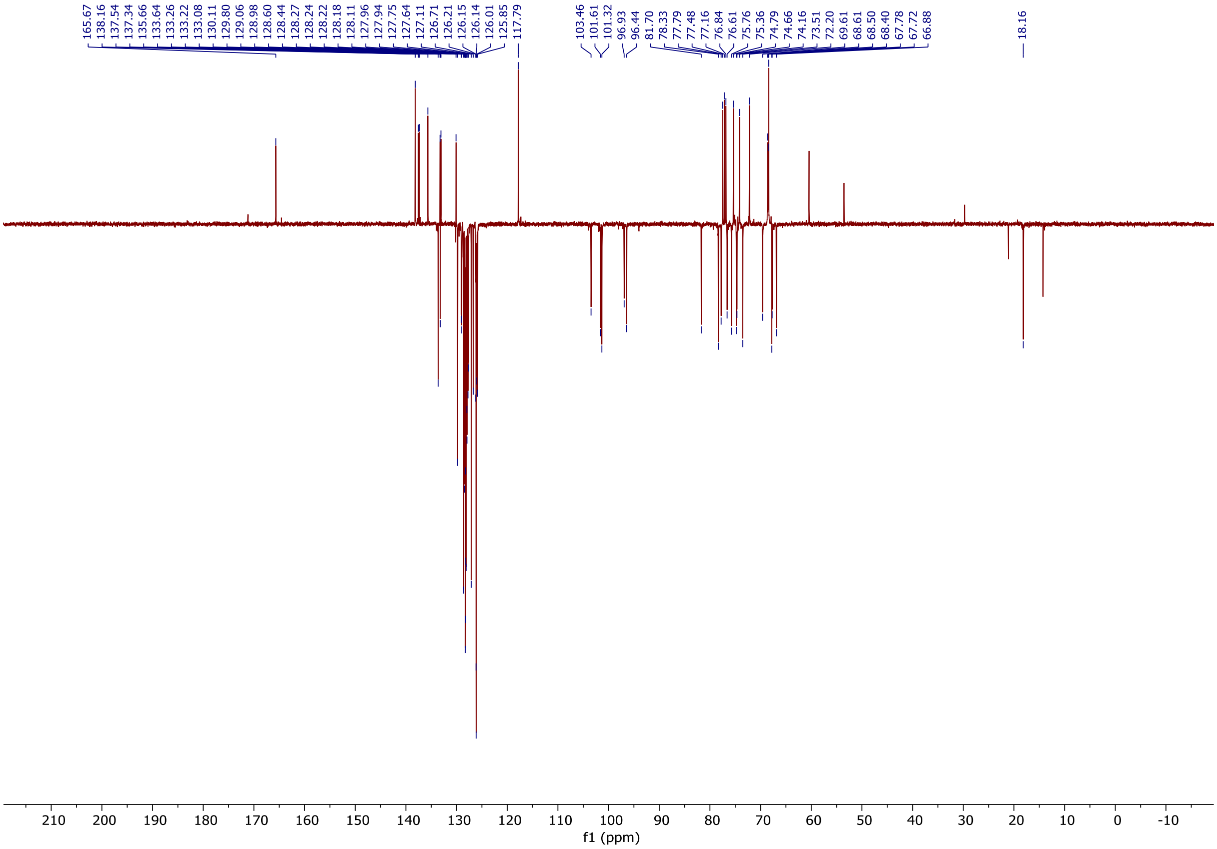
165.67
138.16
137.54
137.34
135.66
133.64
133.26
133.22
133.08
130.11
129.80
129.06
128.98
128.60
128.44
128.27
128.24
128.22
128.18
128.11
127.96
127.94
127.75
127.64
127.11
126.71
126.21
126.15
126.14
126.01
125.85
117.79
103.46
101.61
101.32
96.93
96.44
81.70
78.33
77.79
77.48
77.16
76.84
76.61
75.76
75.36
74.79
74.66
74.16
73.51
72.20
69.61
68.61
68.50
68.40
67.78
67.72
66.88
18.16
f1 (ppm)

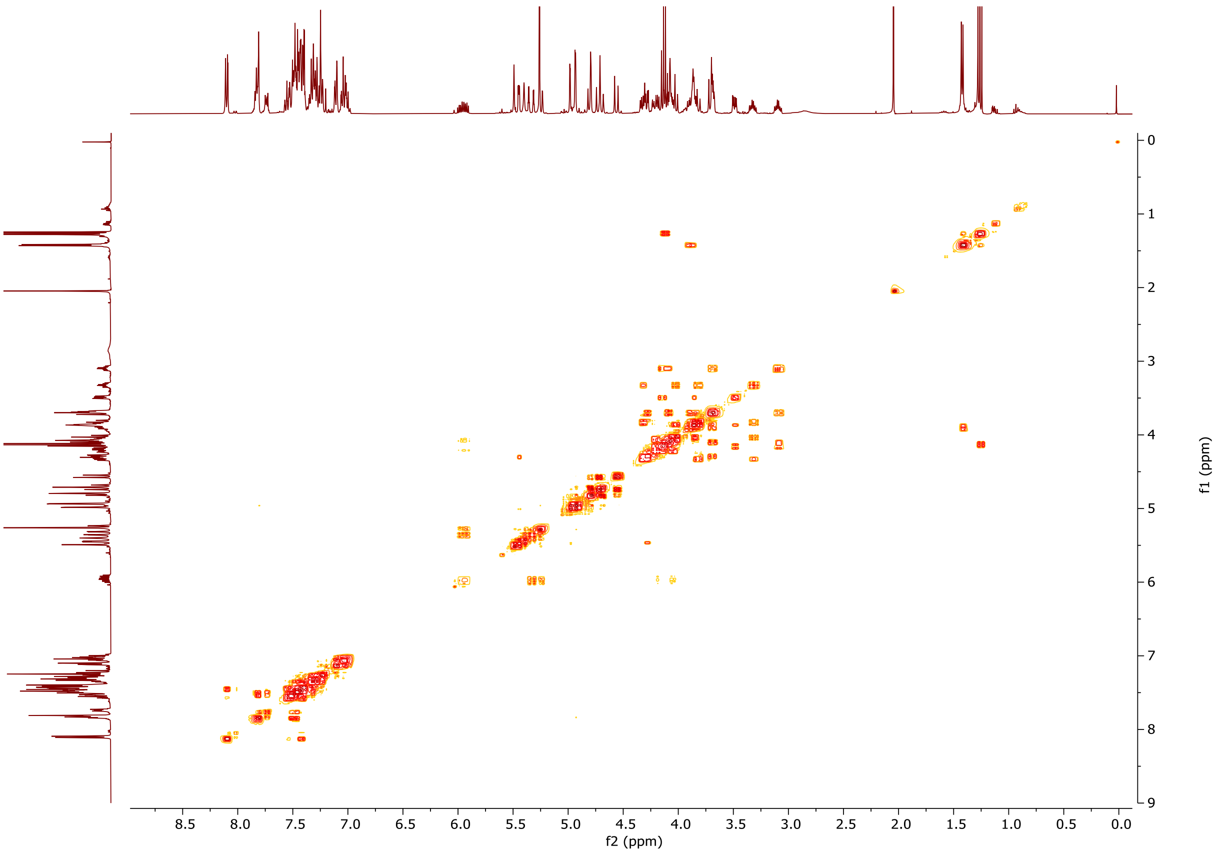
f2 (ppm)
f1 (ppm)

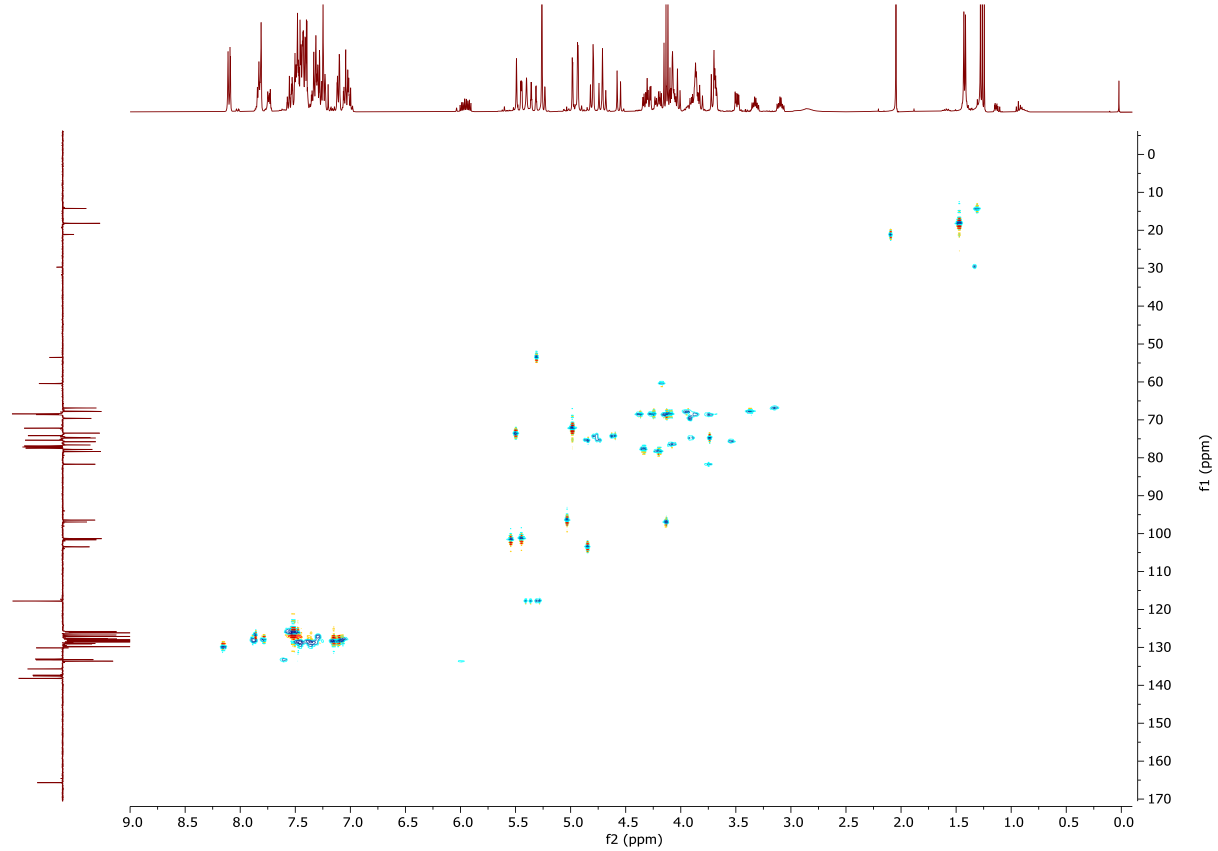
f1 (ppm)
f2 (ppm)

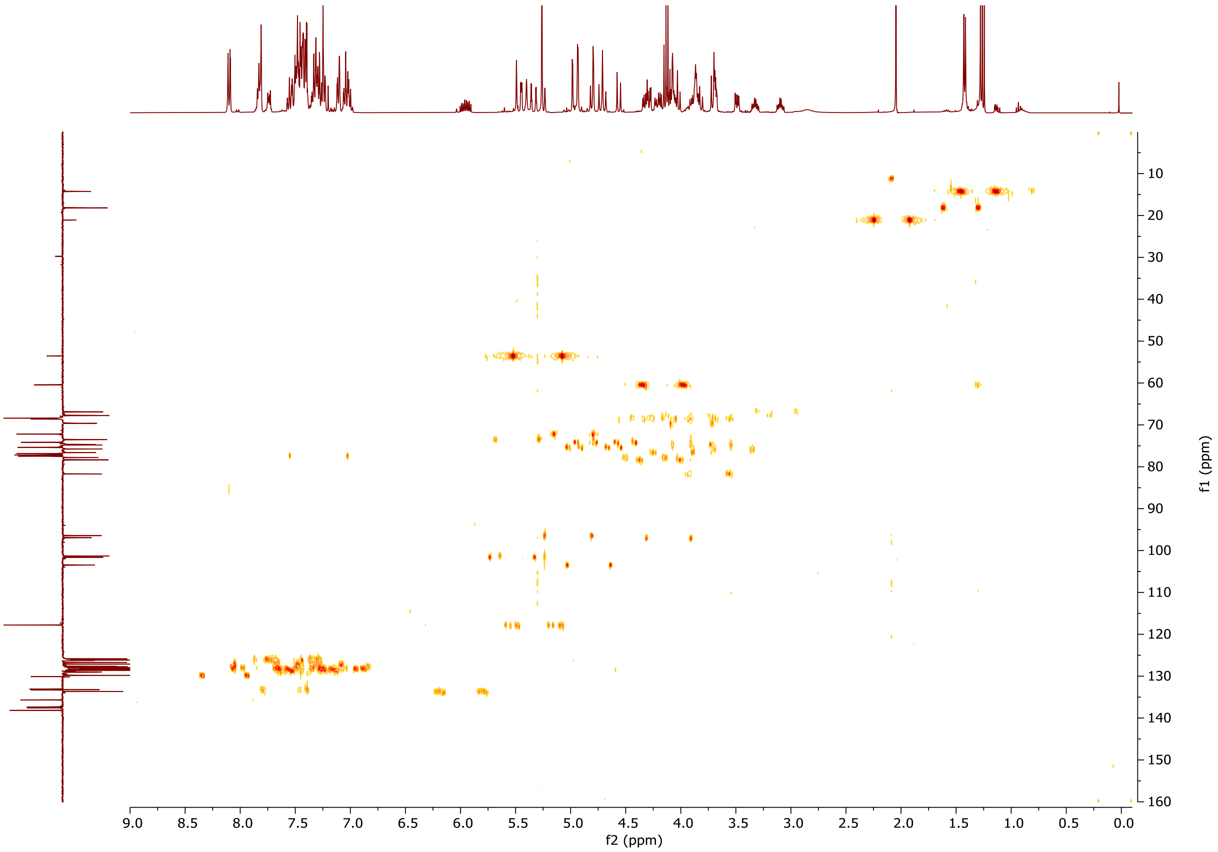
f1 (ppm)
f2 (ppm)

2,3,4,6-*O*-benzoyl-α-D-mannose-(1->2)-4,6-*O*-benzylidene-3-*O*-naphthyl-β-D-mannose-(1->3)-2-*O*-benzyl-4,6-*O*-benzylidene-β-D-mannose-(1->3)-1-*O*-allyl-2-*O*-benzoyl-4-*O*-benzyl-α-L-rhamnose (86)

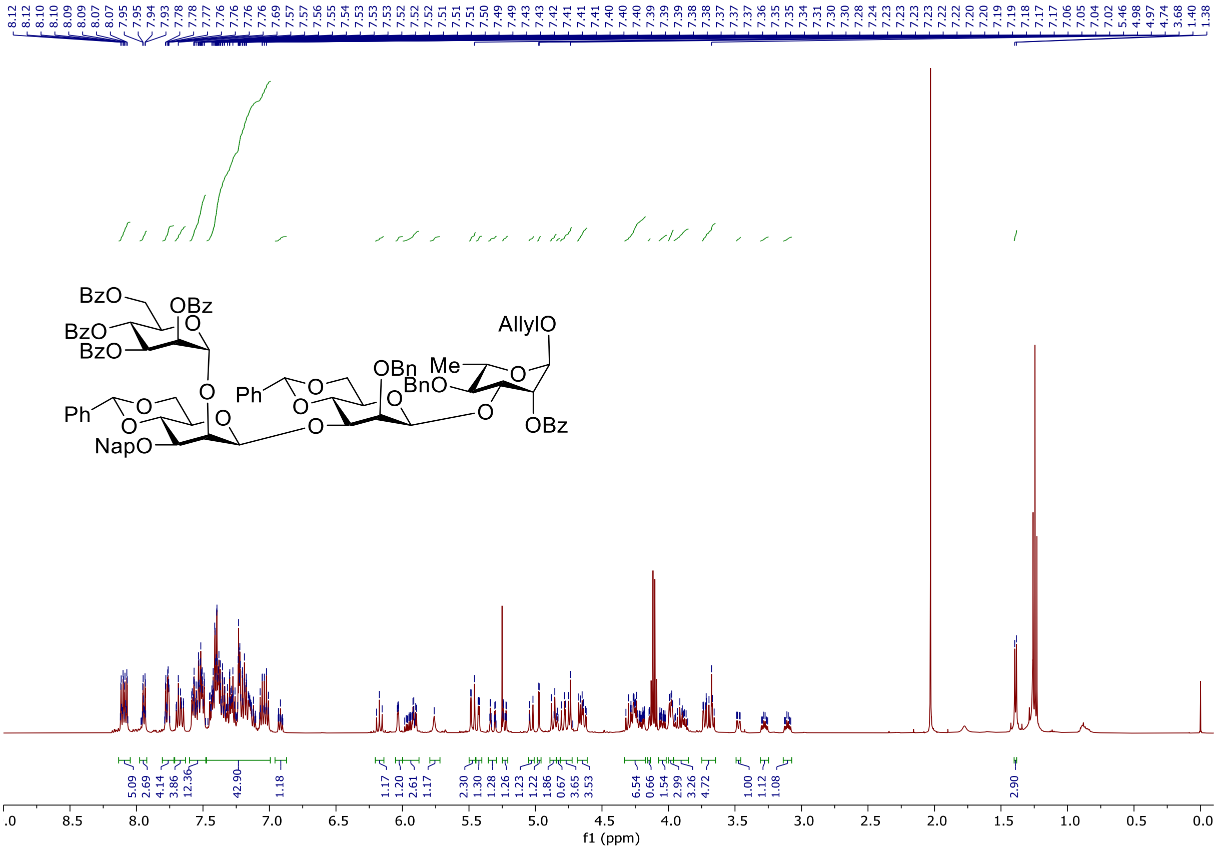

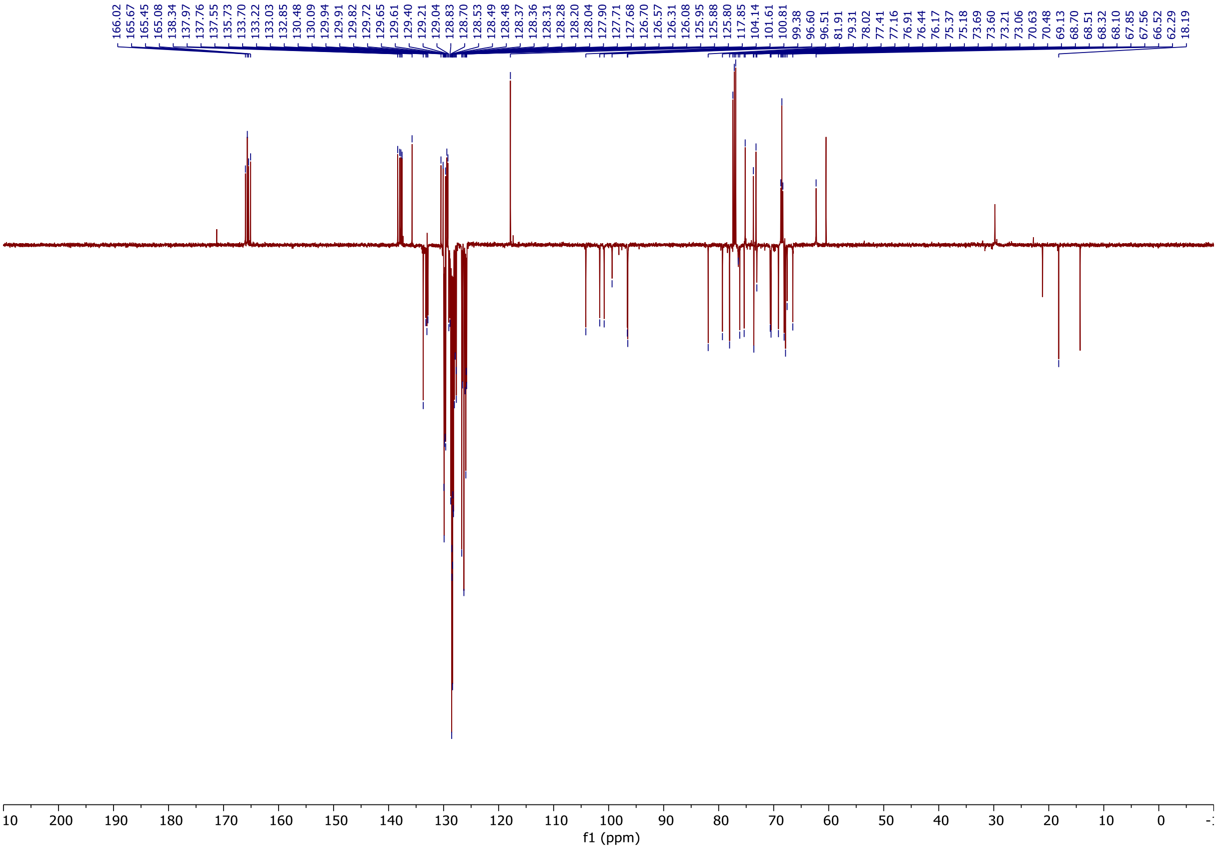

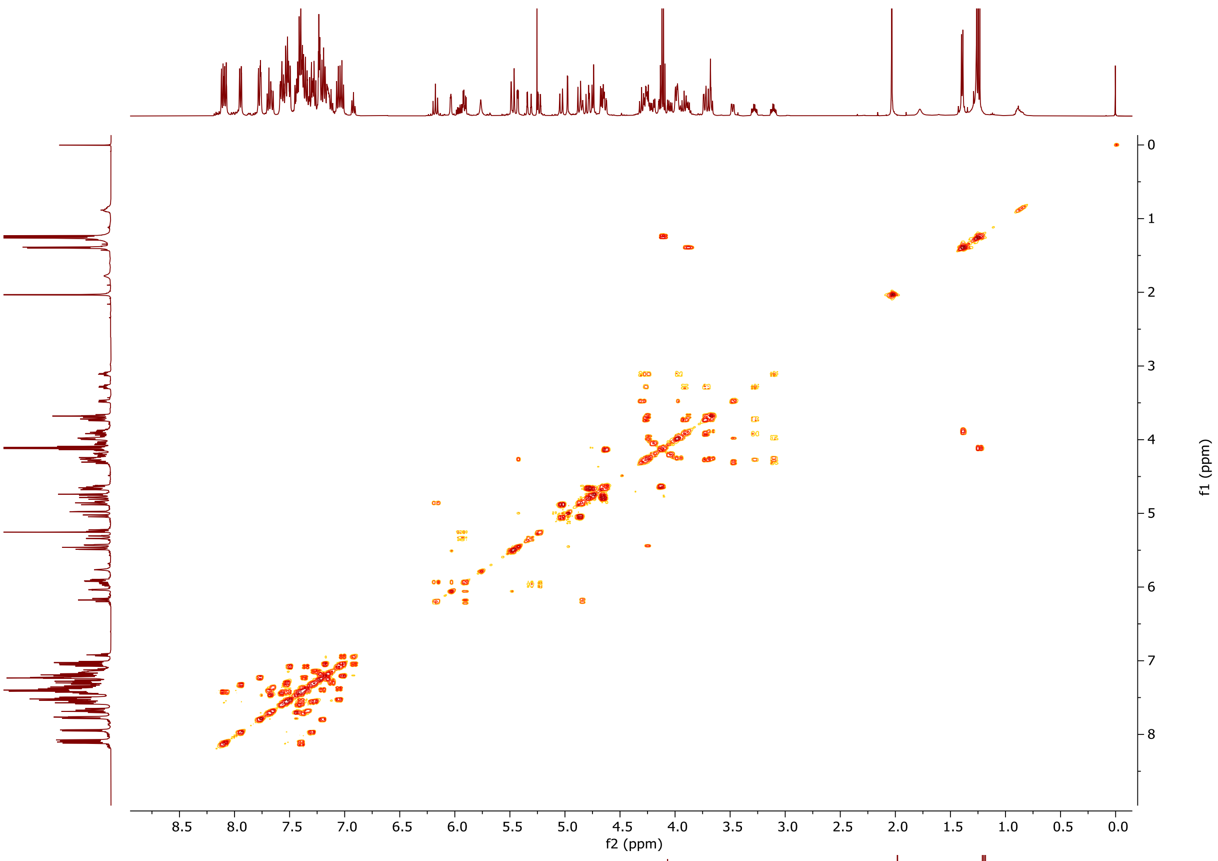
f2 (ppm)
f1 (ppm)

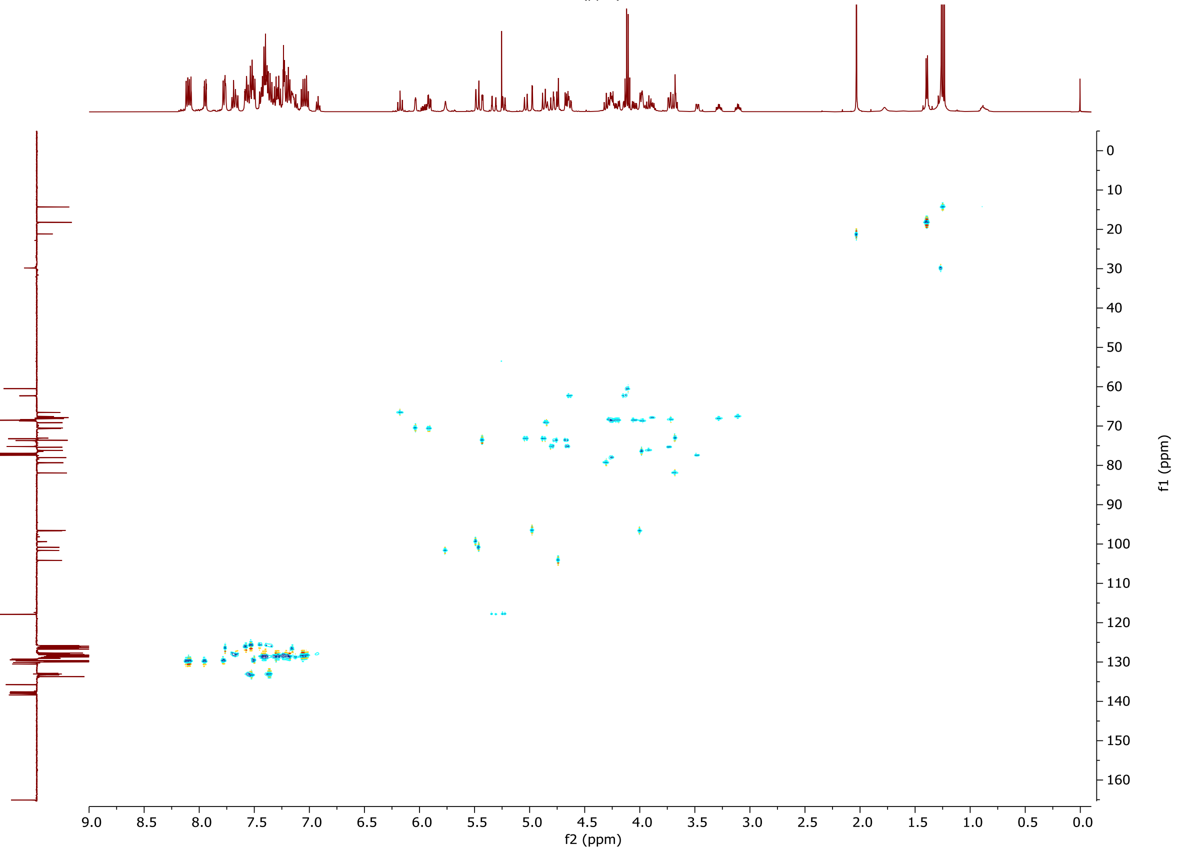
f2 (ppm)
f1 (ppm)

2,3,4,6-*O*-benzoyl-α-D-mannose-(1->2)-4,6-*O*-benzylidene-β-D-mannose-(1->3)-2-*O*-benzyl-4,6-*O*-benzylidene-β-D-mannose-(1->3)-1-*O*-allyl-2-*O*-benzoyl-4-*O*-benzyl-α-L-rhamnose (87)

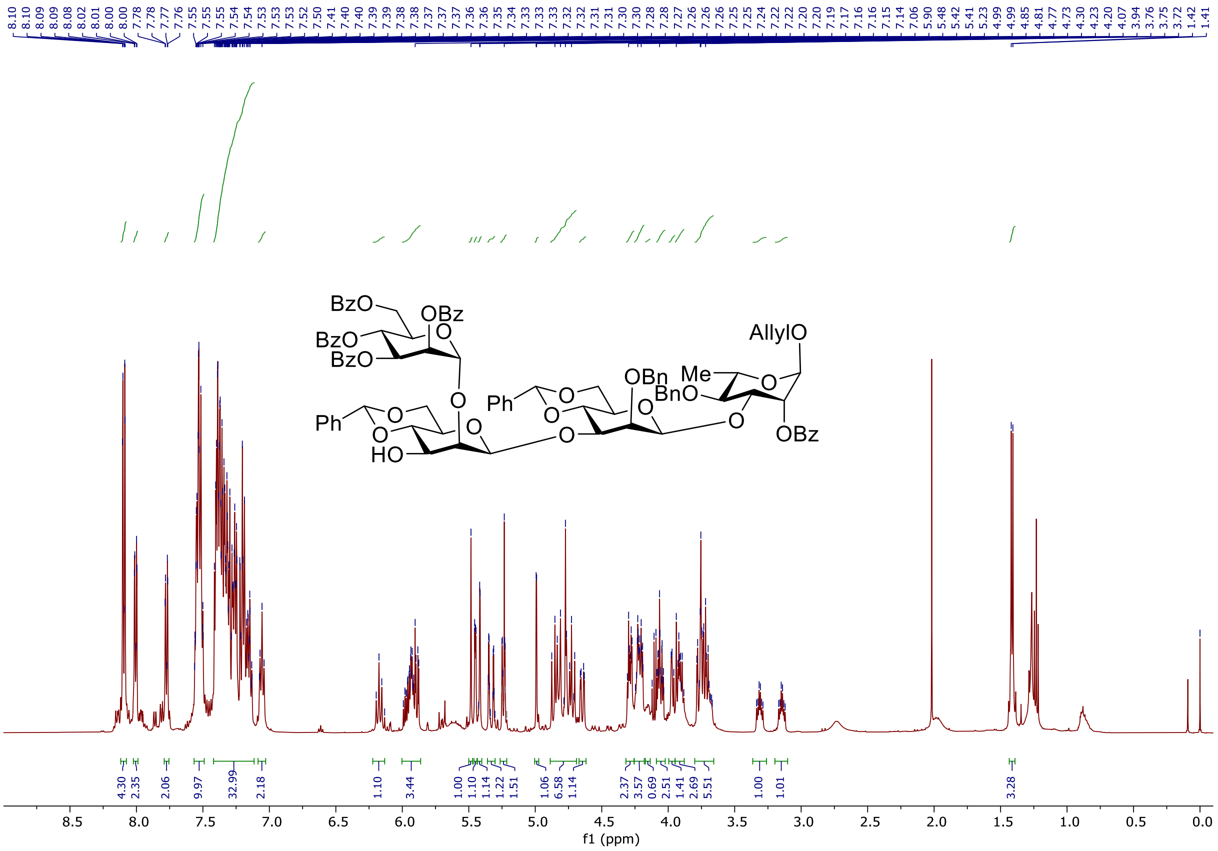

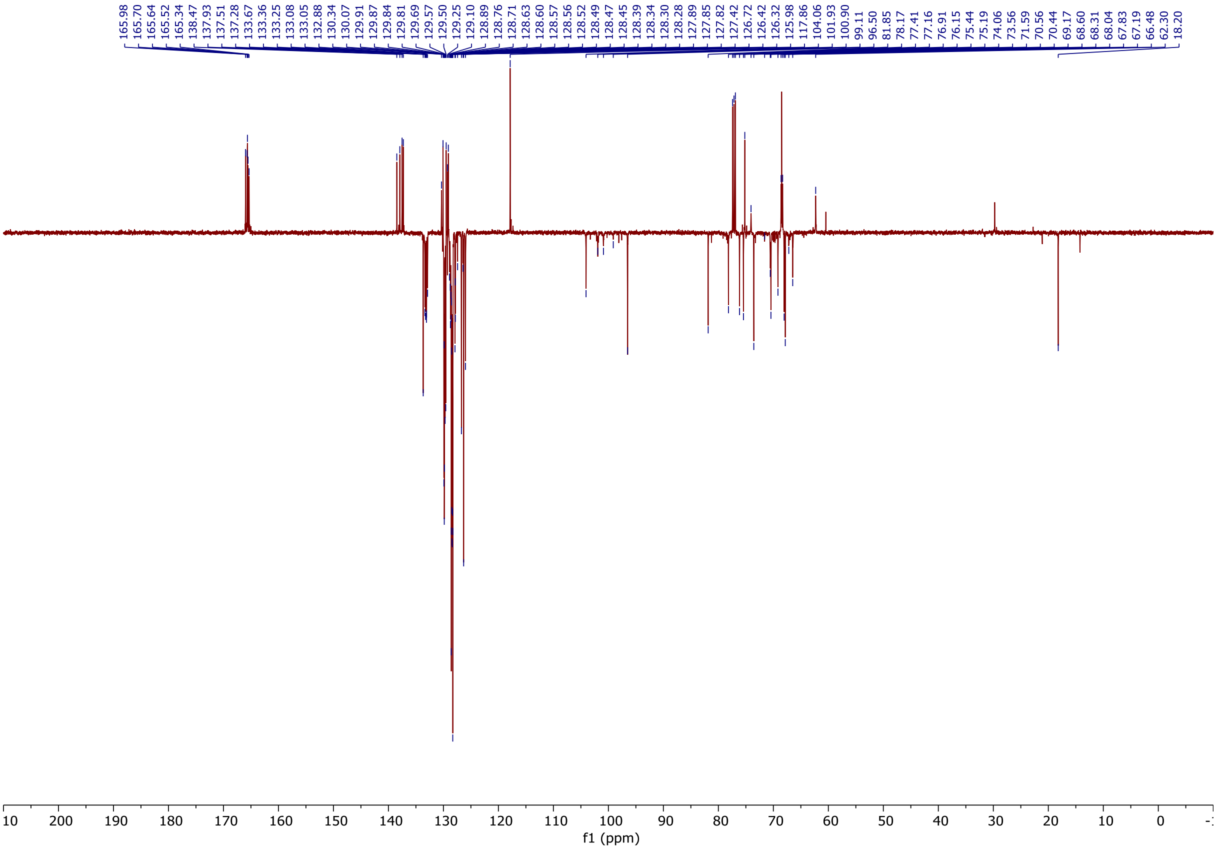

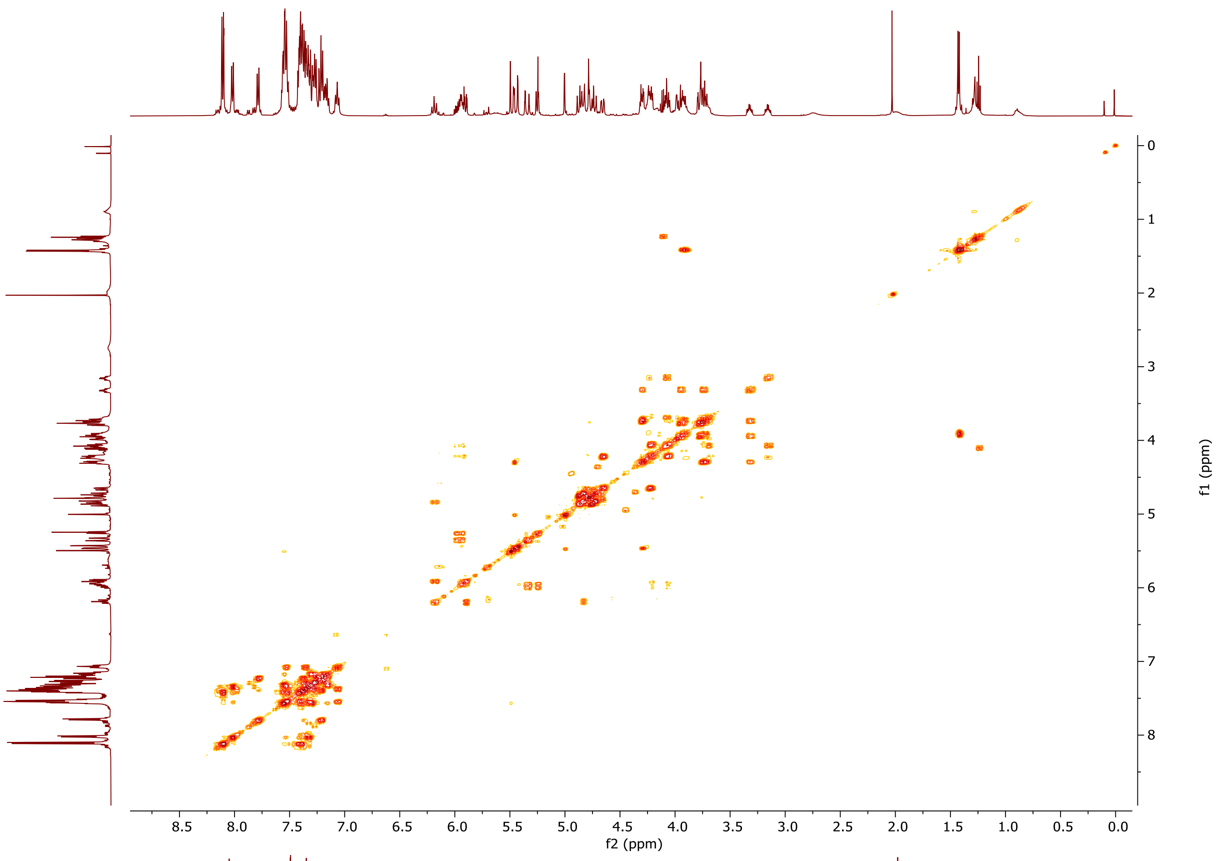

f1 (ppm)
f2 (ppm)

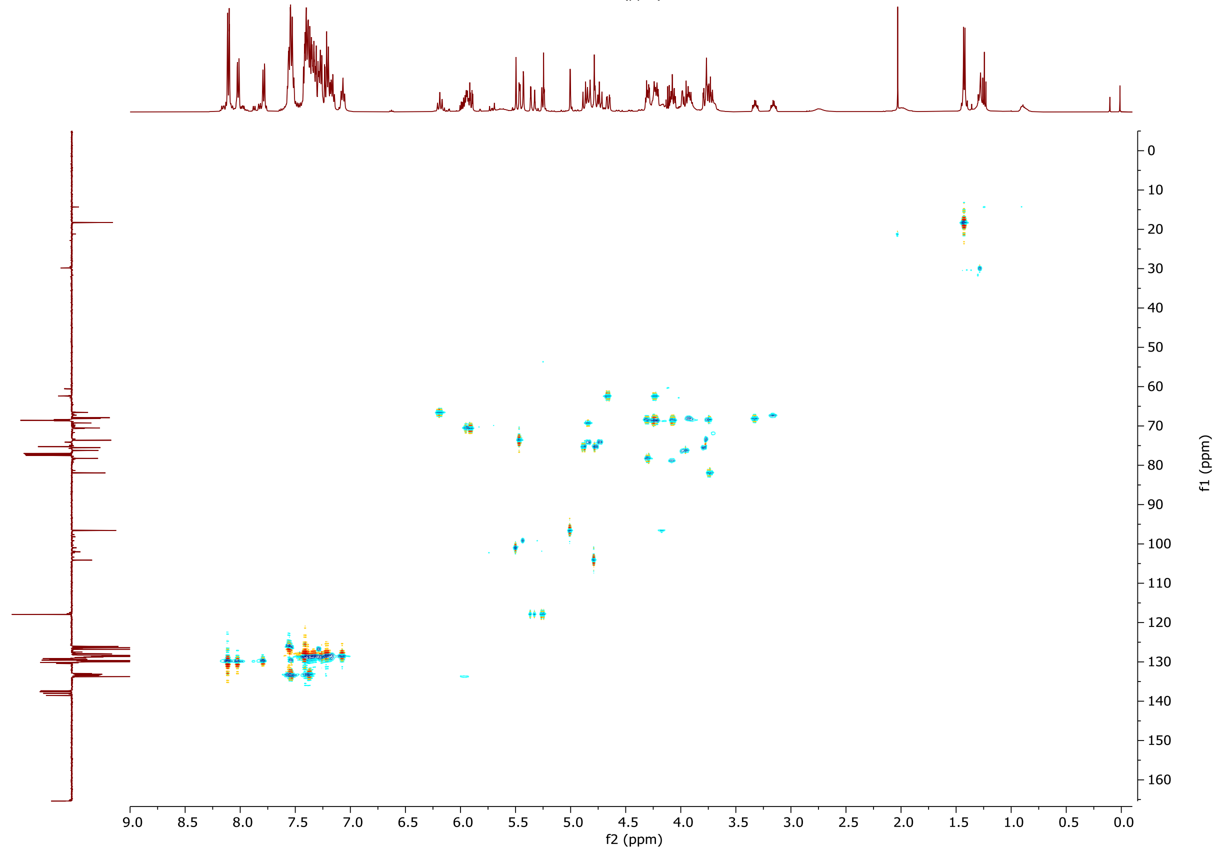

f1 (ppm)
f2 (ppm)

2,4,6-*O*-benzoyl-3-*O*-naphthyl-β-D-glucose-(1->3)-4,6-*O*-benzylidene-2-*O*-(2,3,4,6-*O*-benzoyl-α-D-mannose-(1->2))-β-D-mannose-(1->3)-2-*O*-benzyl-4,6-*O*-benzylidene-β-D-mannose-(1->3)-1-*O*-allyl-2-*O*-benzoyl-4-*O*-benzyl-α-L-rhamnose (88)

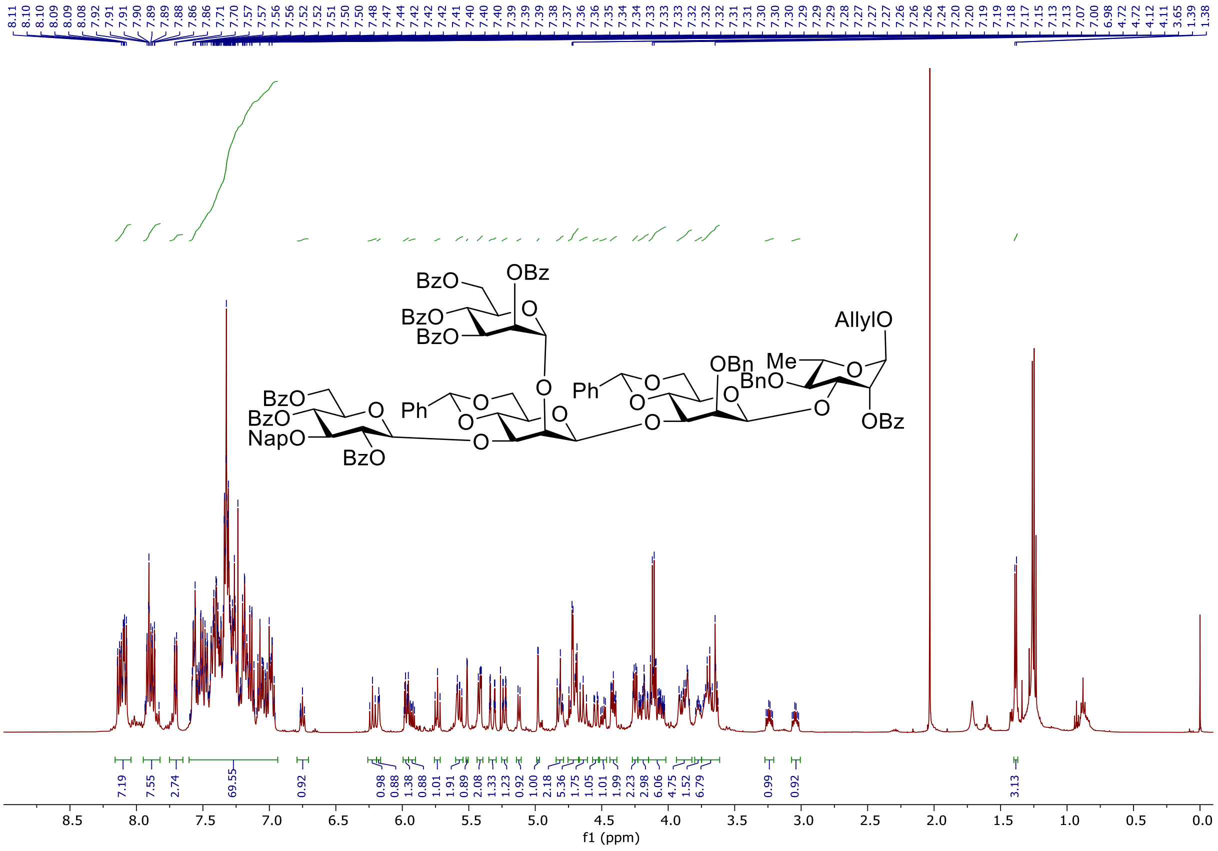

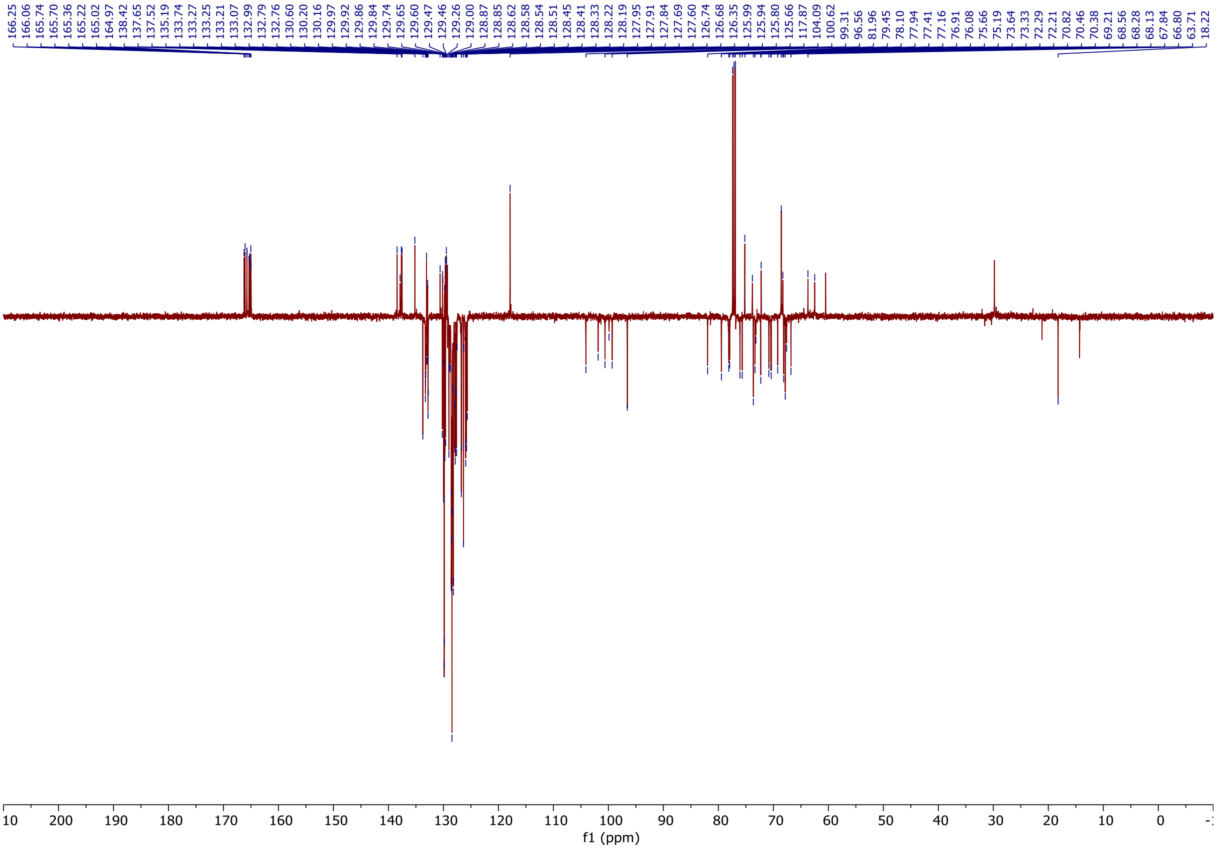

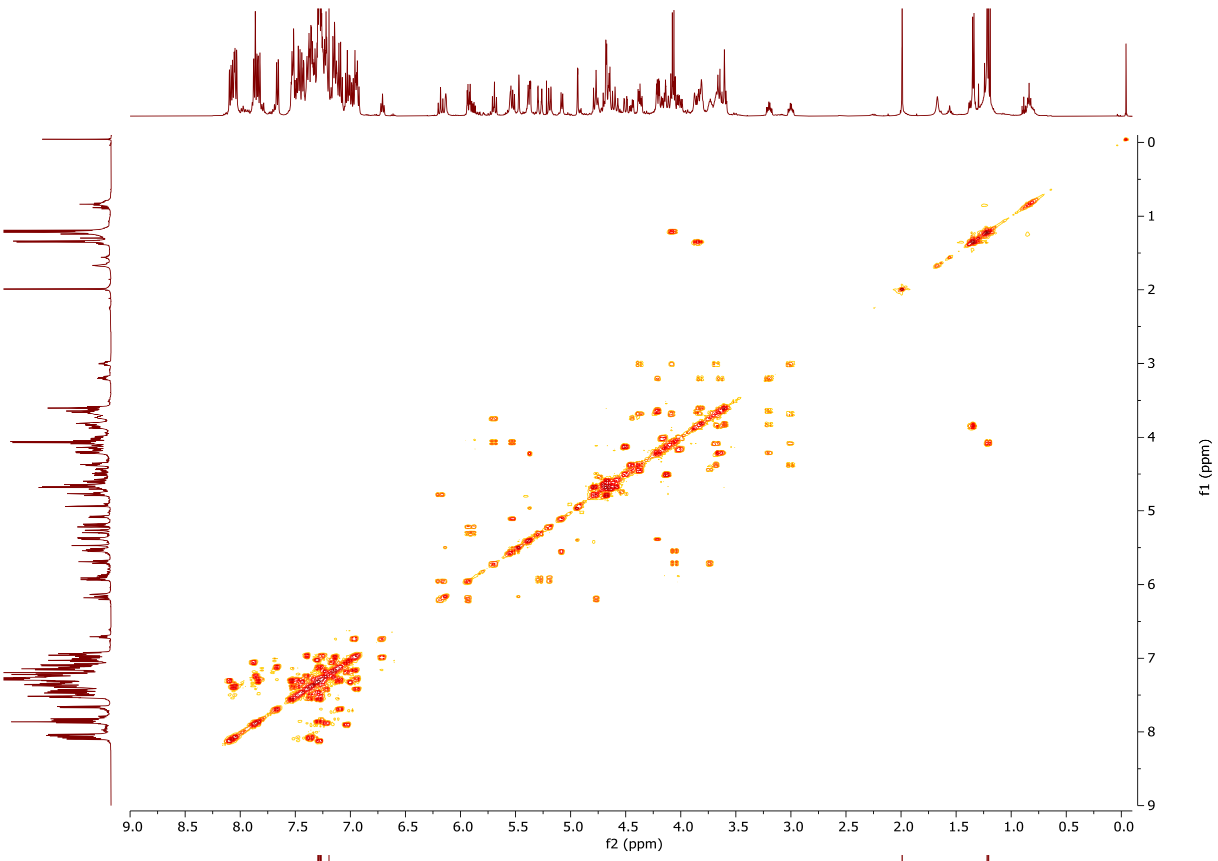
f2 (ppm)
f1 (ppm)

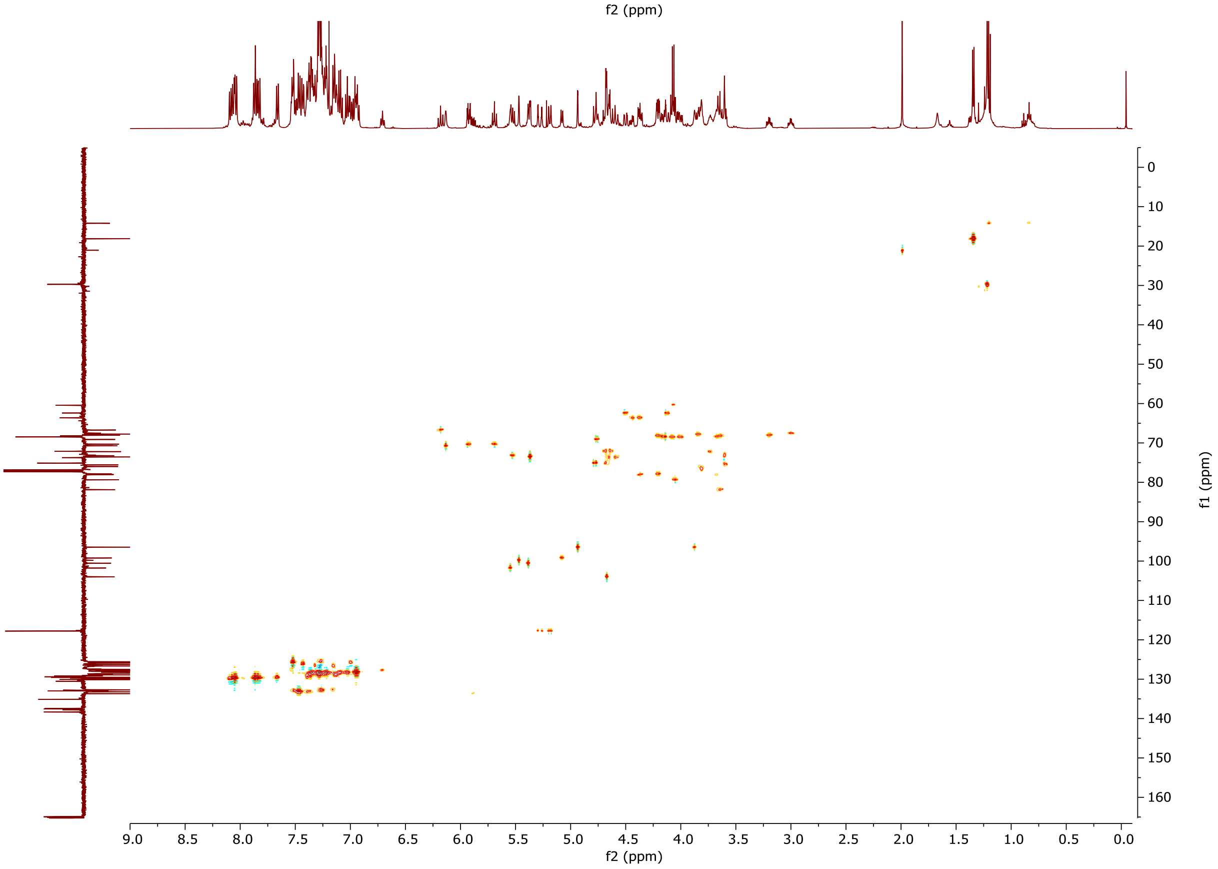
f2 (ppm)
f1 (ppm)

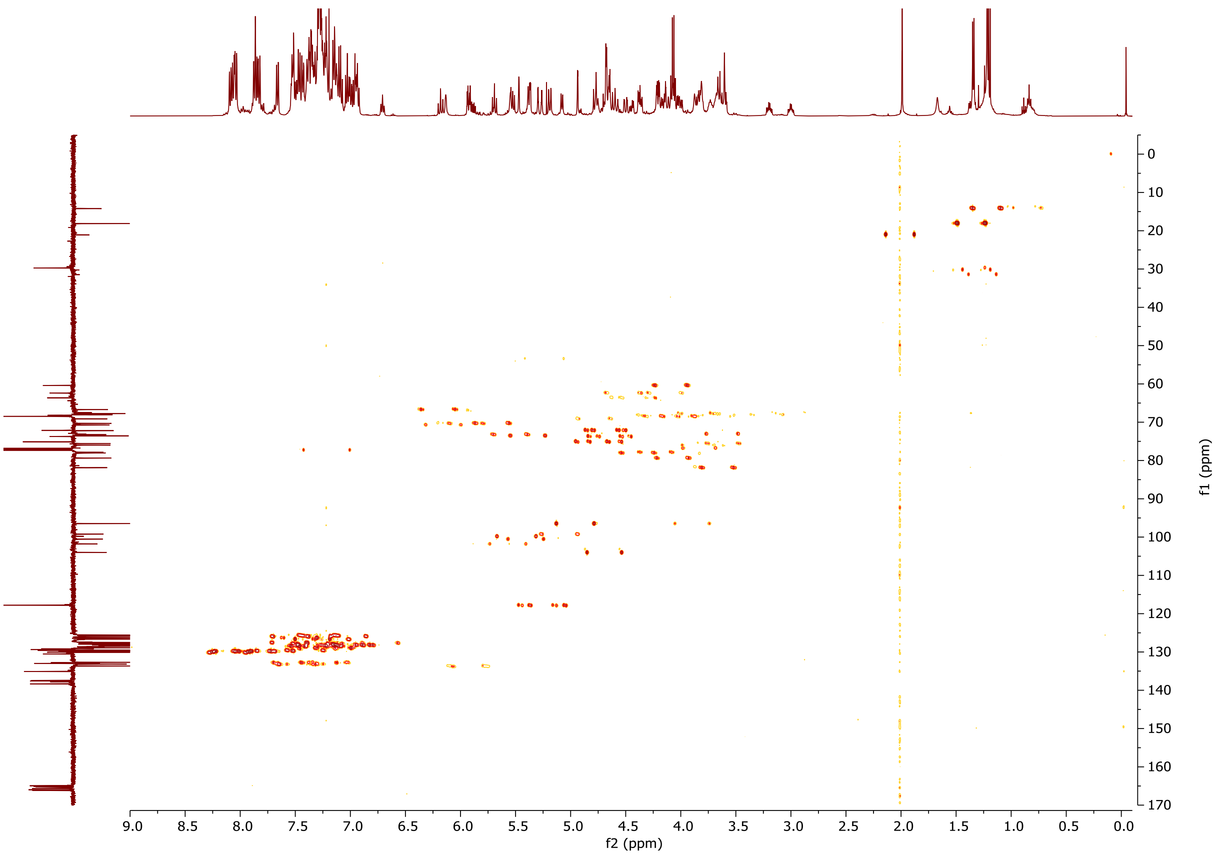

2,4,6-*O*-benzoyl-β-D-glucose-(1->3)-4,6-*O*-benzylidene-2-*O*-(2,3,4,6-*O*-benzoyl-α-D-mannose-(1->2))-β-D-mannose-(1->3)-2-*O*-benzyl-4,6-*O*-benzylidene-β-D-mannose-(1->3)-1-*O*-allyl-2-*O*-benzoyl-4-*O*-benzyl-α-L-rhamnose (89)

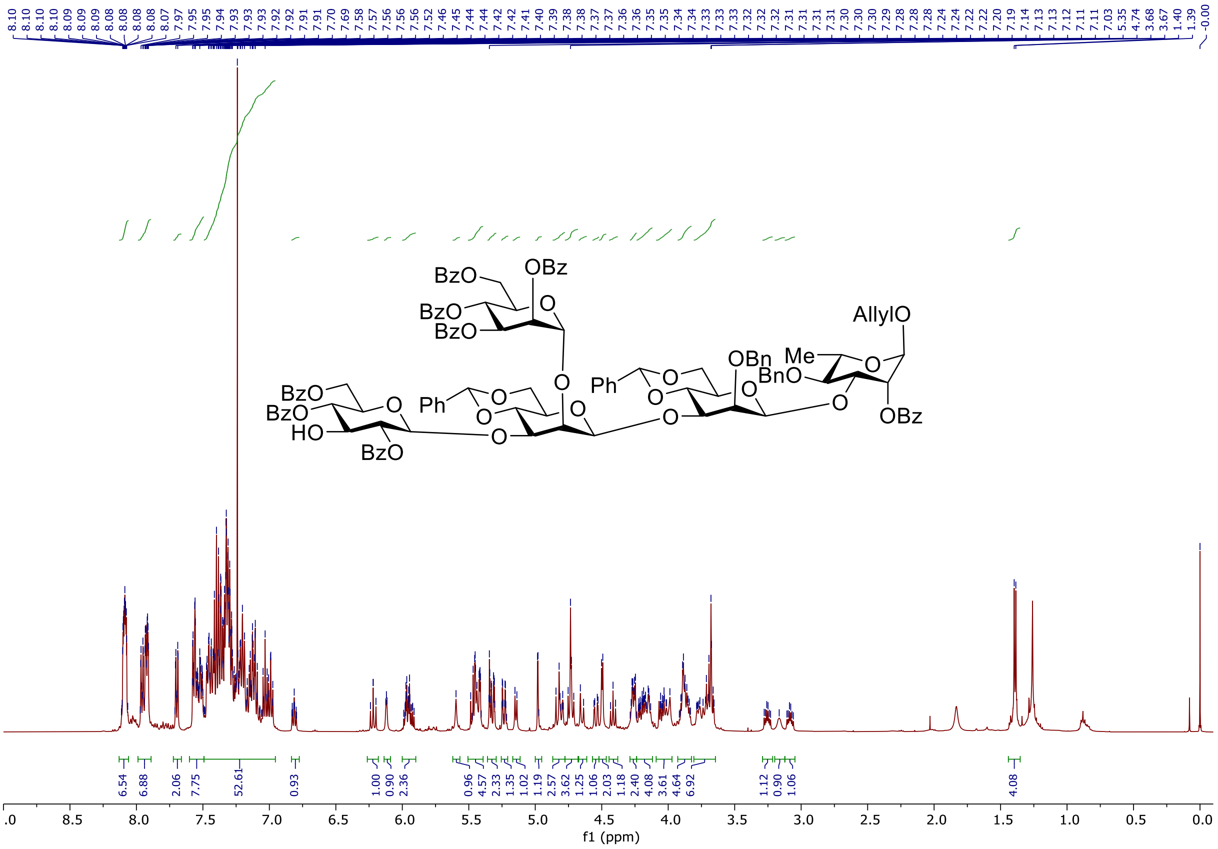

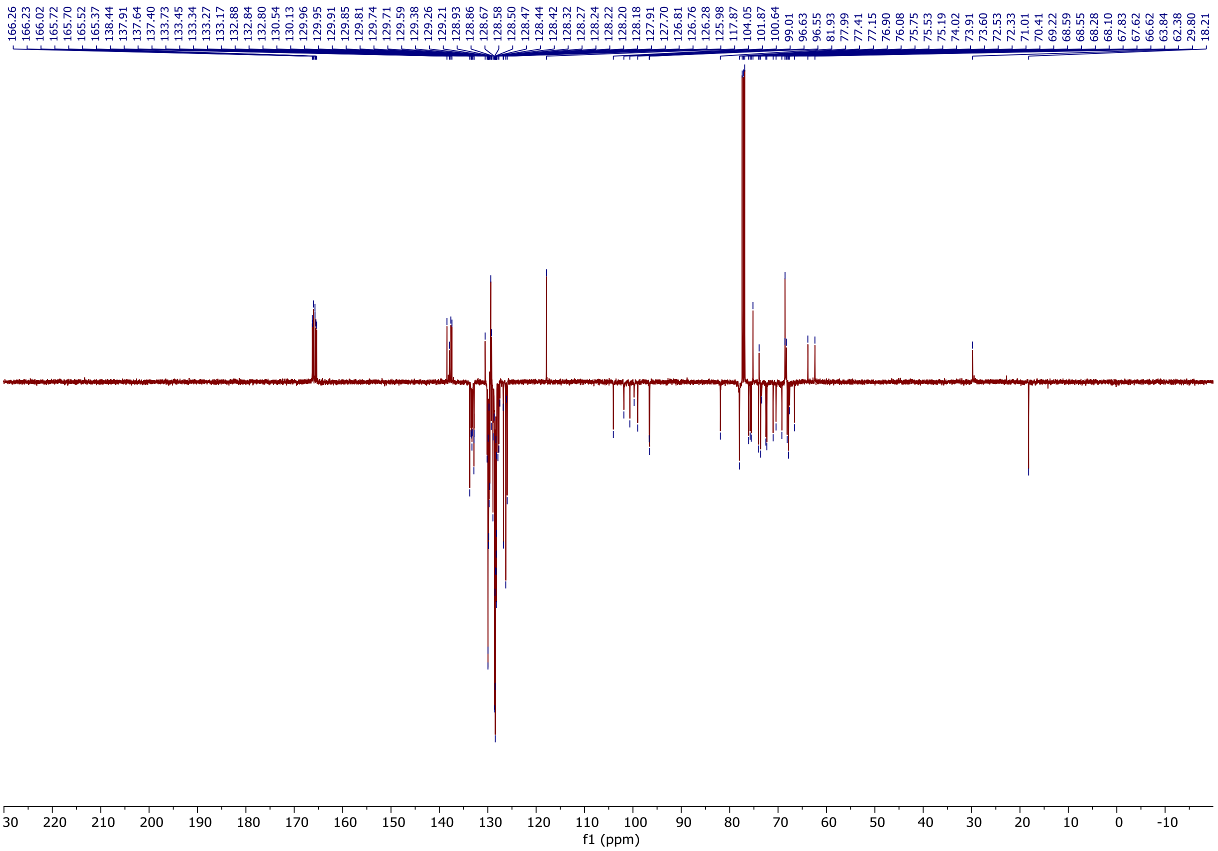
166.26
166.23
166.02
165.72
165.70
165.52
165.37
138.44
137.91
137.64
137.40
133.73
133.45
133.34
133.27
133.17
132.88
132.84
132.80
130.54
130.13
129.96
129.95
129.91
129.85
129.81
129.74
129.71
129.59
129.38
129.26
129.21
128.93
128.86
128.67
128.58
128.50
128.47
128.44
128.42
128.32
128.27
128.24
128.22
128.20
128.18
127.91
127.70
126.81
126.76
126.28
125.98
117.87
104.05
101.87
100.64
99.01
96.63
96.55
81.93
77.99
77.41
77.15
76.90
76.08
75.75
75.53
75.19
74.02
73.91
73.60
72.53
72.33
71.01
70.41
69.22
68.59
68.55
68.28
68.10
67.83
67.62
66.62
63.84
62.38
29.80
18.21
f1 (ppm)

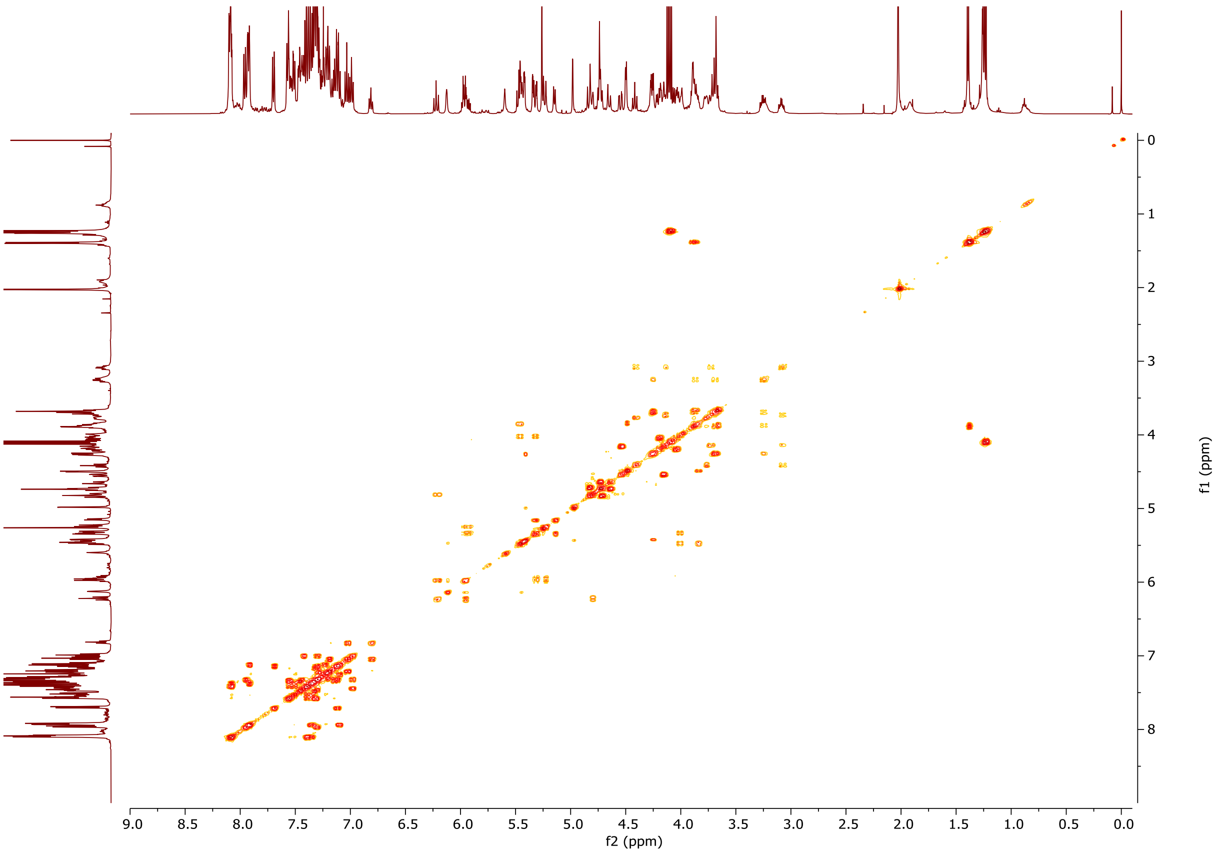
f2 (ppm)
f1 (ppm)

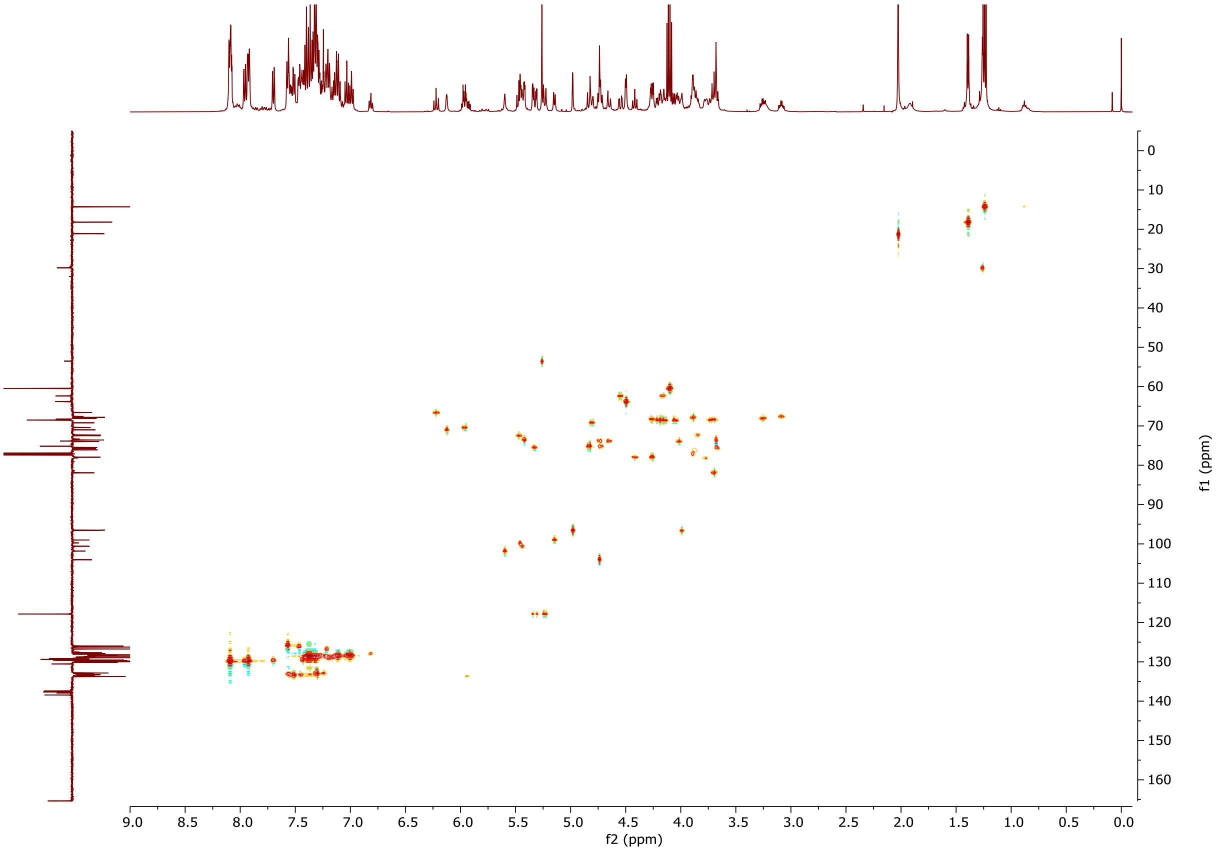

2,4,6-*O*-benzoyl-3-*O*-naphthyl-β-D-glucose-(1->3)-4,6-*O*-benzylidene-2-*O*-(2,3,4,6-*O*-benzoyl-α-D-mannose-(1->2))-β-D-mannose-(1->3)-2-*O*-benzyl-4,6-*O*-benzylidene-β-D-mannose-(1->3)-2-*O*-benzoyl-4-*O*-benzyl-α-L-rhamnose (90)

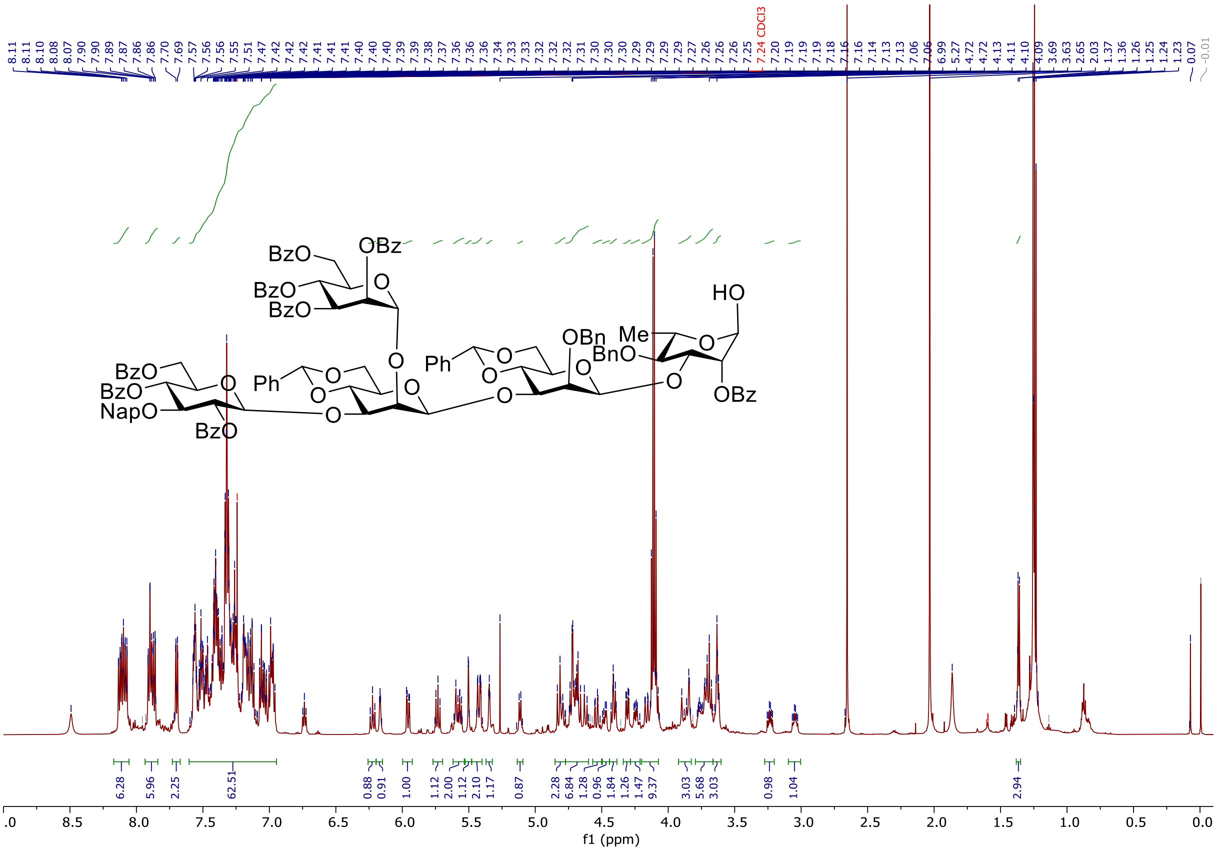

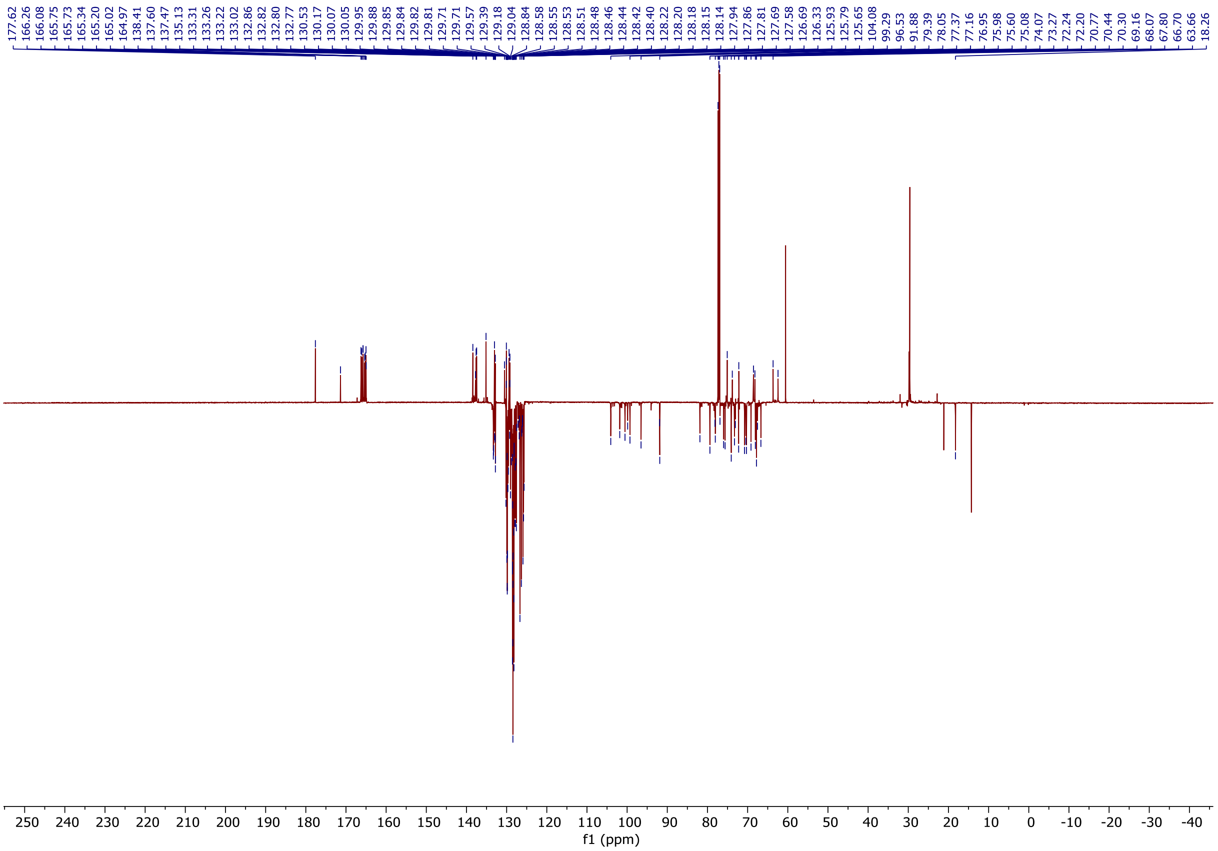

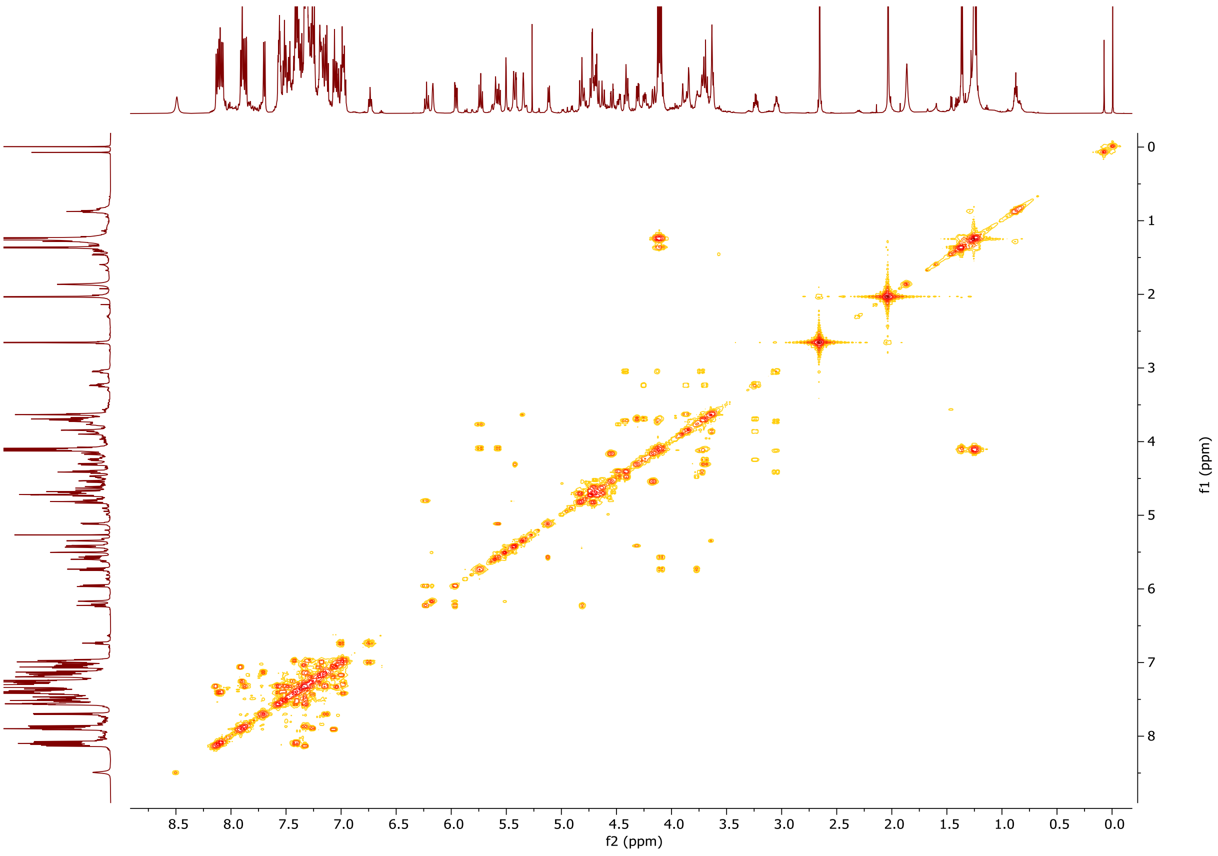

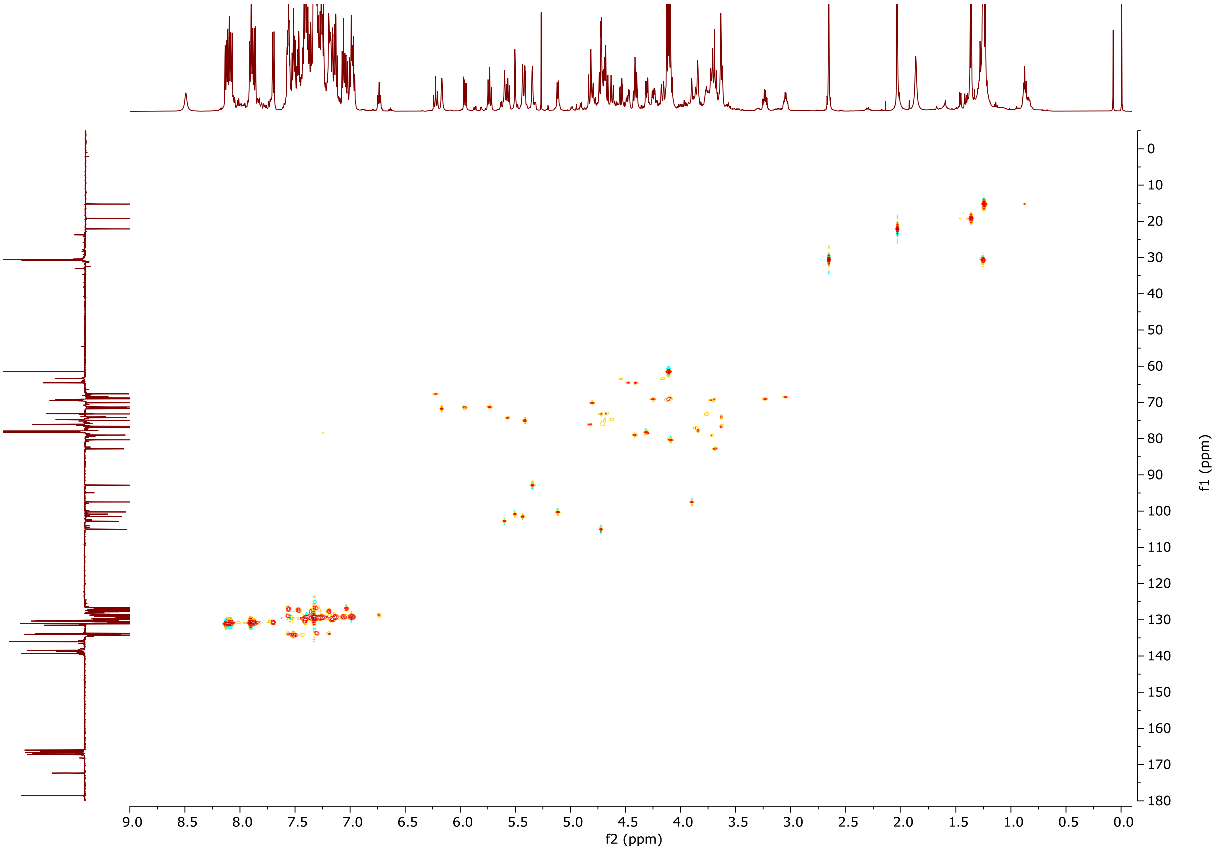

2,4,6-*O*-benzoyl-3-*O*-naphthyl-β-D-glucose-(1->3)-4,6-*O*-benzylidene-2-*O*-(2,3,4,6-*O*-benzoyl-α-D-mannose-(1->2))-β-D-mannose-(1->3)-2-*O*-benzyl-4,6-*O*-benzylidene-β-D-mannose-(1->3)-1-*O*-trifluoro-N-phenyl-imidate-2-*O*-benzoyl-4-*O*-benzyl-α-L-rhamnose (91)

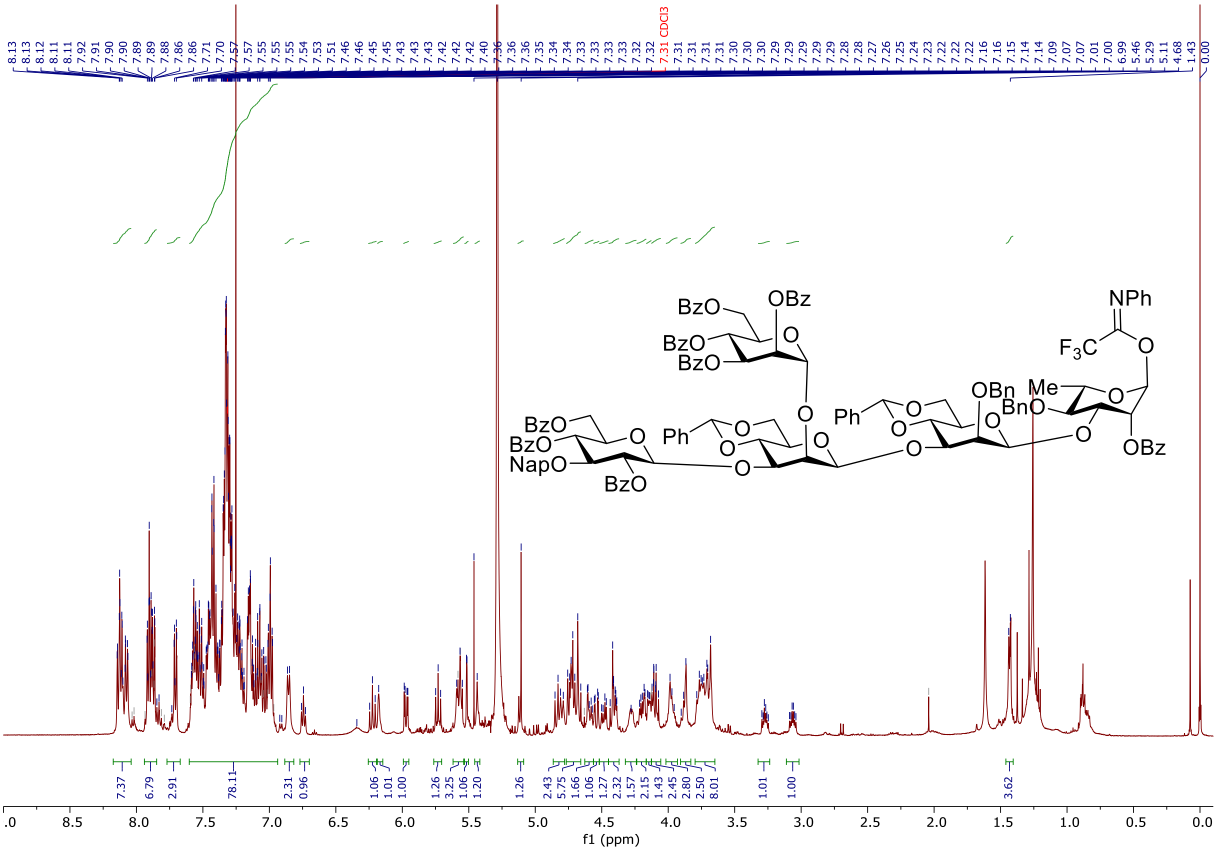

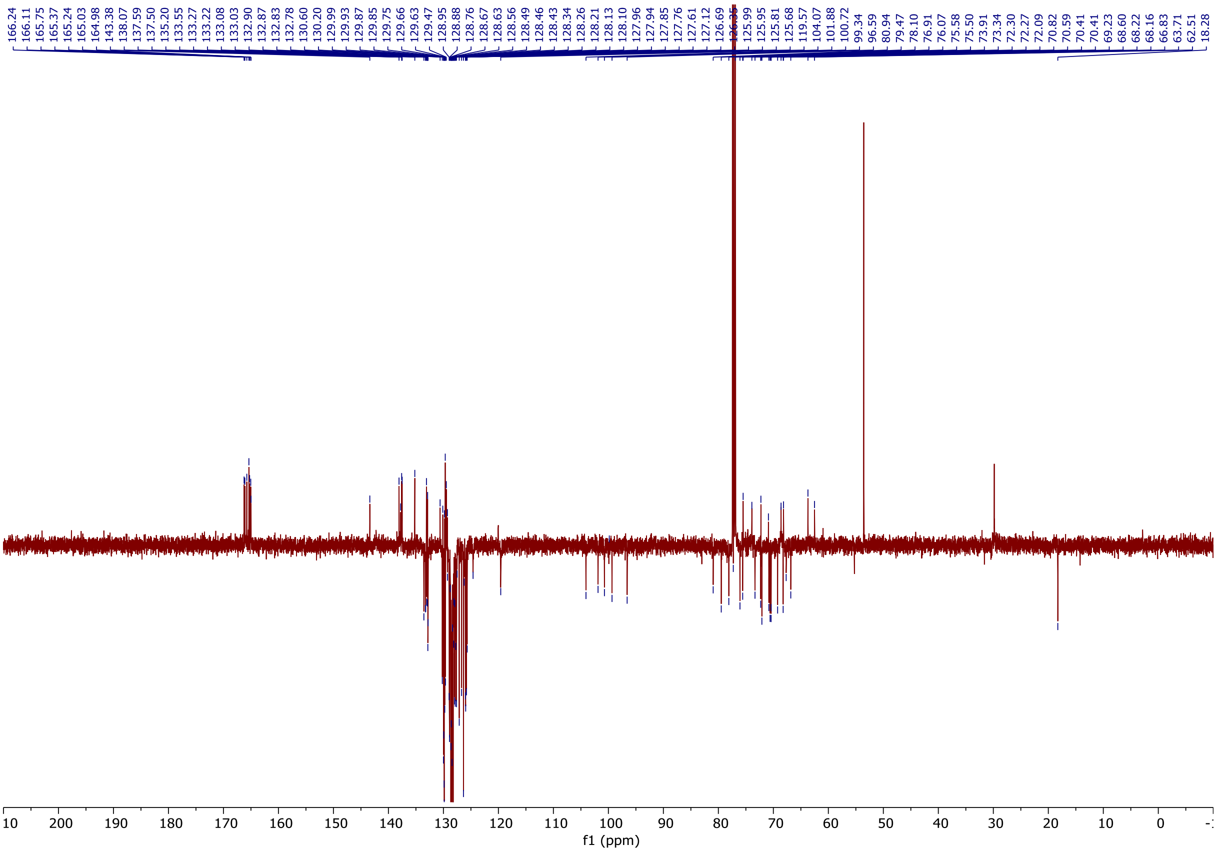
166.24
166.11
165.75
165.37
165.24
165.03
164.98
143.38
138.07
137.59
137.50
135.20
133.55
133.27
133.22
133.08
133.03
132.90
132.87
132.83
132.78
130.60
130.20
129.99
129.93
129.87
129.85
129.75
129.66
129.63
129.47
128.95
128.88
128.76
128.67
128.63
128.56
128.49
128.46
128.43
128.34
128.26
128.21
128.13
128.10
127.96
127.94
127.85
127.76
127.61
127.12
126.69
125.99
125.95
125.81
125.68
119.57
104.07
101.88
100.72
99.34
96.59
80.94
79.47
78.10
76.07
76.91
75.58
75.50
73.91
73.34
72.30
72.27
72.09
70.82
70.59
70.41
70.41
69.23
68.60
68.22
68.16
66.83
63.71
62.51
18.28
f1 (ppm)

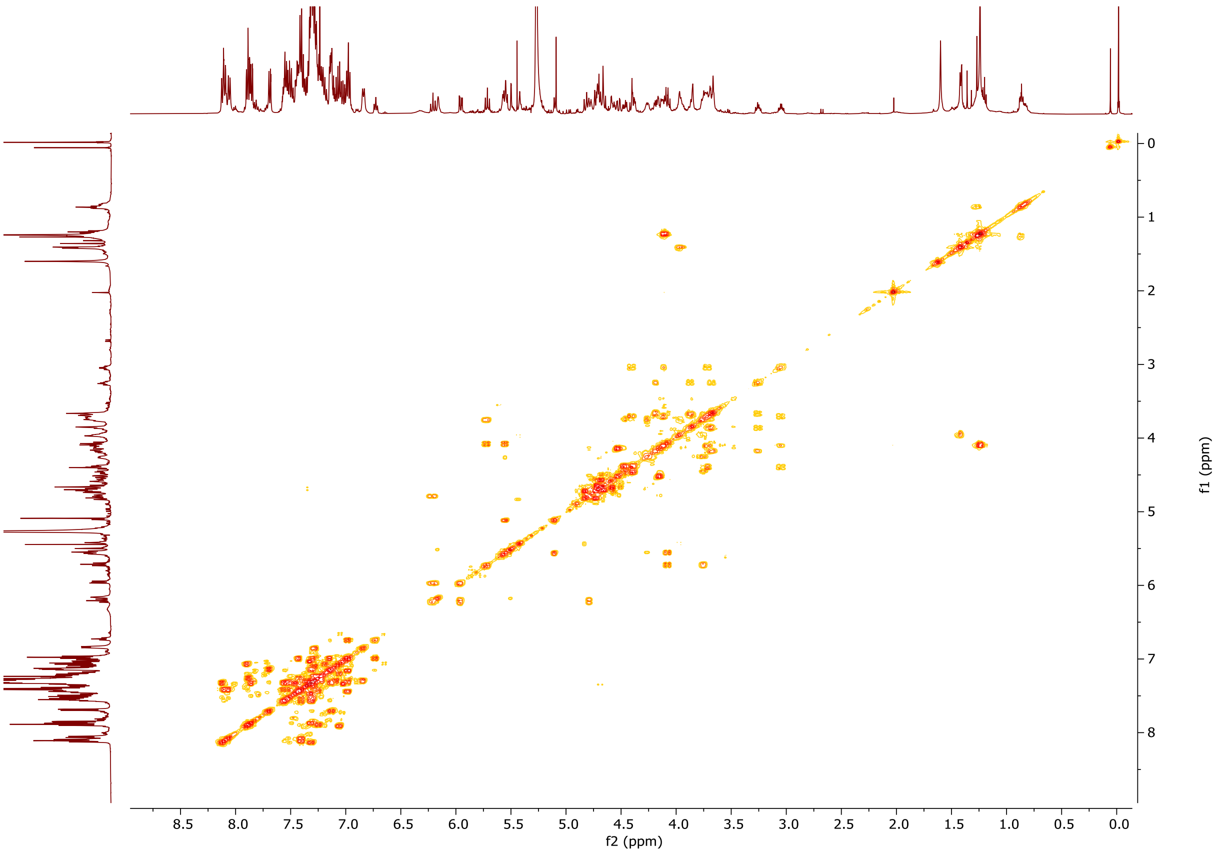
f2 (ppm)
f1 (ppm)

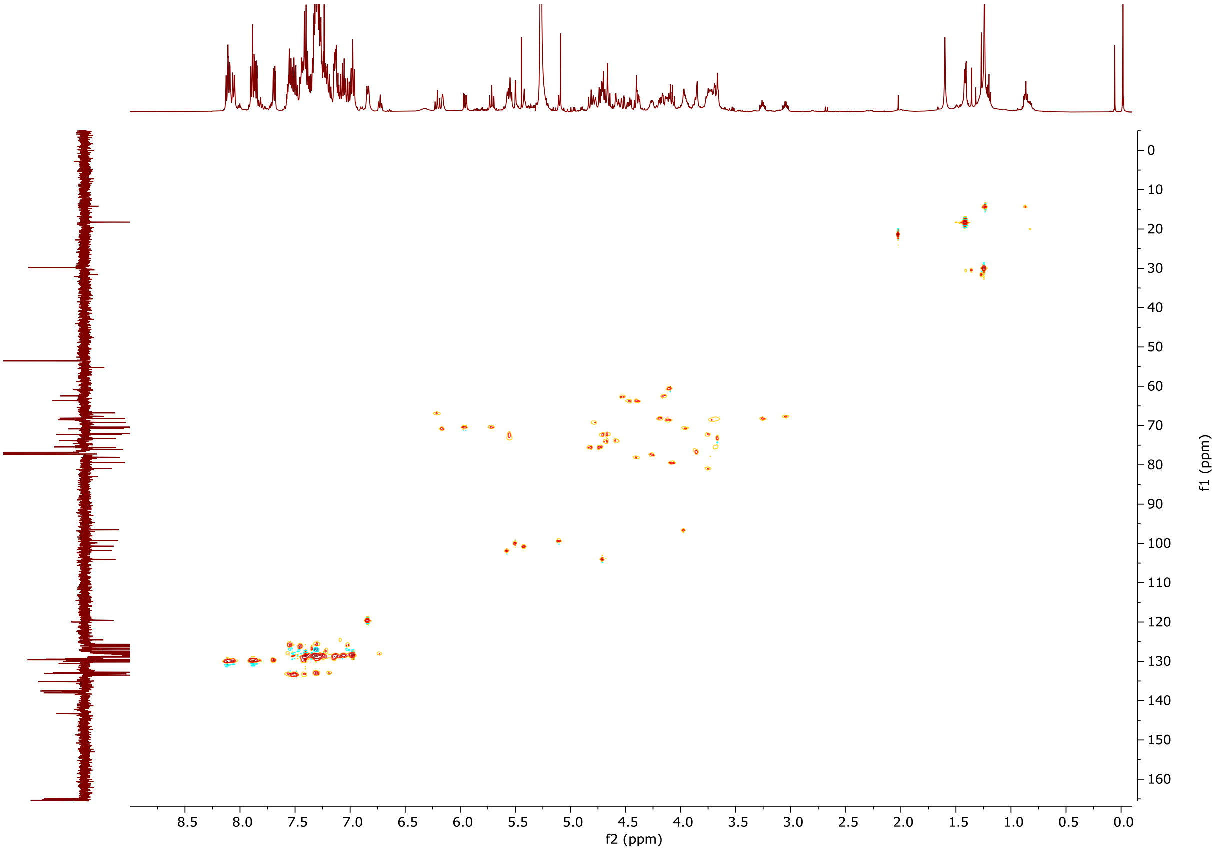

2,4,6-*O*-benzoyl-3-*O*-naphthyl-β-D-glucose-(1->3)-4,6-*O*-benzylidene-2-*O*-(2,3,4,6-*O*-benzoyl-α-D-mannose-(1->2))-β-D-mannose-(1->3)-2-*O*-benzyl-4,6-*O*-benzylidene-β-D-mannose-(1->3)-2-*O*-benzoyl-4-*O*-benzyl-α-L-rhamnose-(1->3)-2,4,6-*O*-benzoyl-β-D-glucose-(1->3)-4,6-*O*-benzylidene-2-*O*-(2,3,4,6-*O*-benzoyl-α-D-mannose-(1->2))-β-D-mannose-(1->3)-2-*O*-benzyl-4,6-*O*-benzylidene-β-D-mannose-(1->3)-1-*O*-allyl-2-*O*-benzoyl-4-*O*-benzyl-α-L-rhamnose (92)

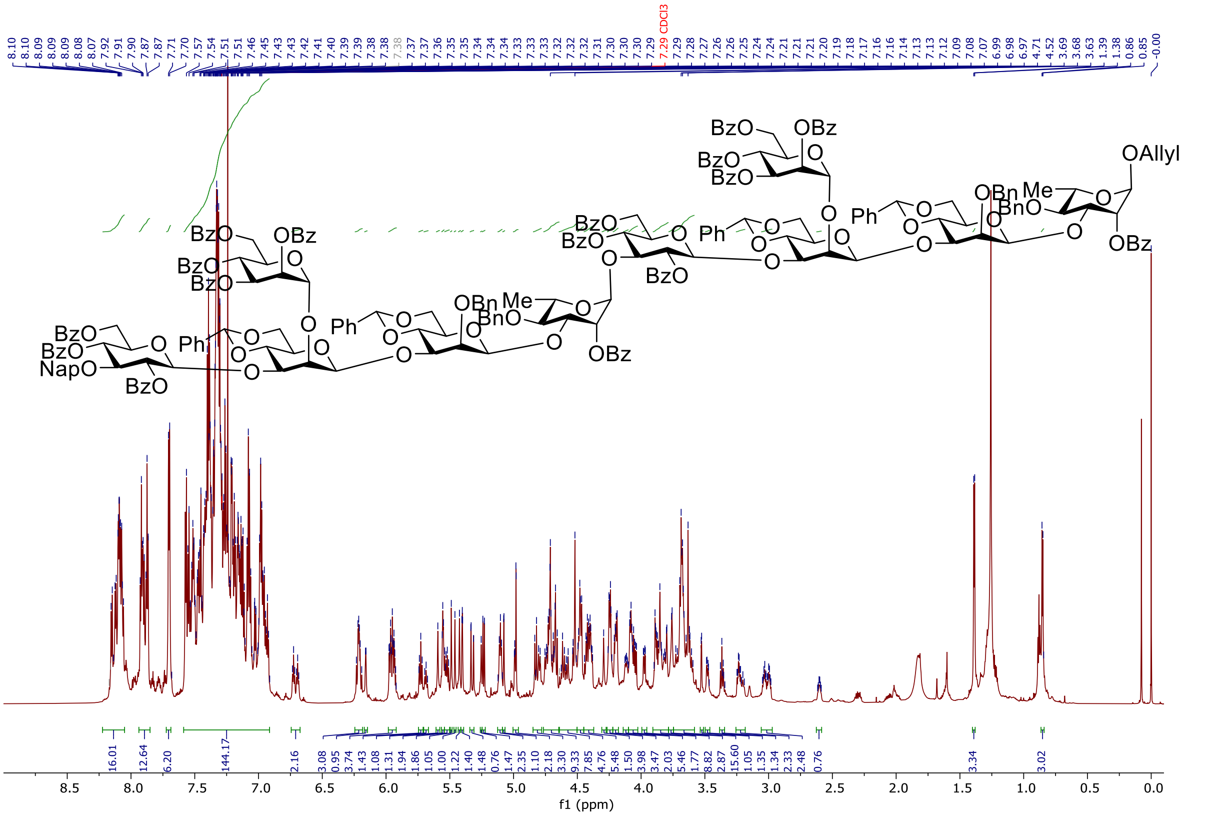

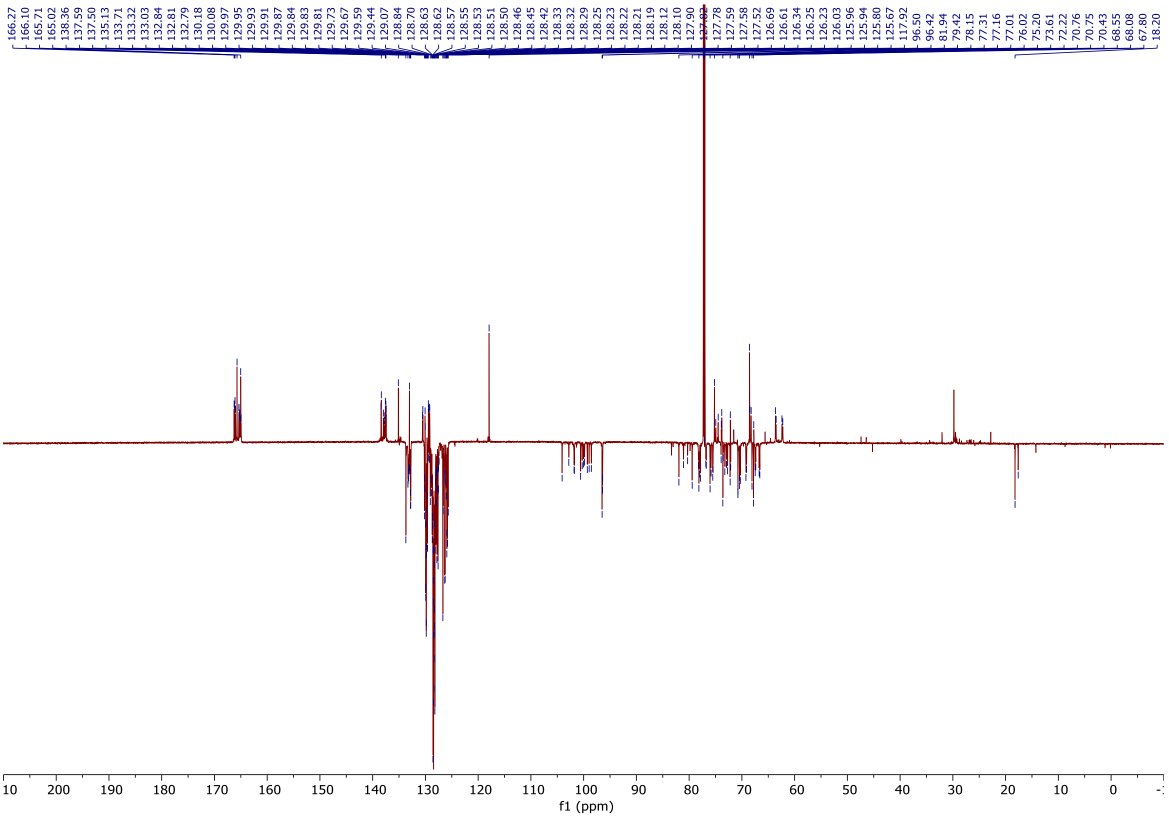
166.27
166.10
165.71
165.02
138.36
137.59
137.50
135.13
133.71
133.32
133.03
132.84
132.81
132.79
130.18
130.08
129.97
129.95
129.93
129.91
129.87
129.84
129.83
129.81
129.73
129.67
129.59
129.44
129.07
128.84
128.70
128.63
128.62
128.57
128.55
128.53
128.51
128.50
128.46
128.45
128.42
128.33
128.32
128.29
128.25
128.23
128.22
128.21
128.19
128.12
128.10
127.90
[illegible]
127.78
127.59
127.58
127.52
126.69
126.61
126.34
126.25
126.23
126.03
125.96
125.94
125.80
125.67
117.92
96.50
96.42
81.94
79.42
78.15
77.31
77.16
77.01
76.02
75.20
73.61
72.22
70.76
70.75
70.43
68.55
68.08
67.80
18.20
f1 (ppm)

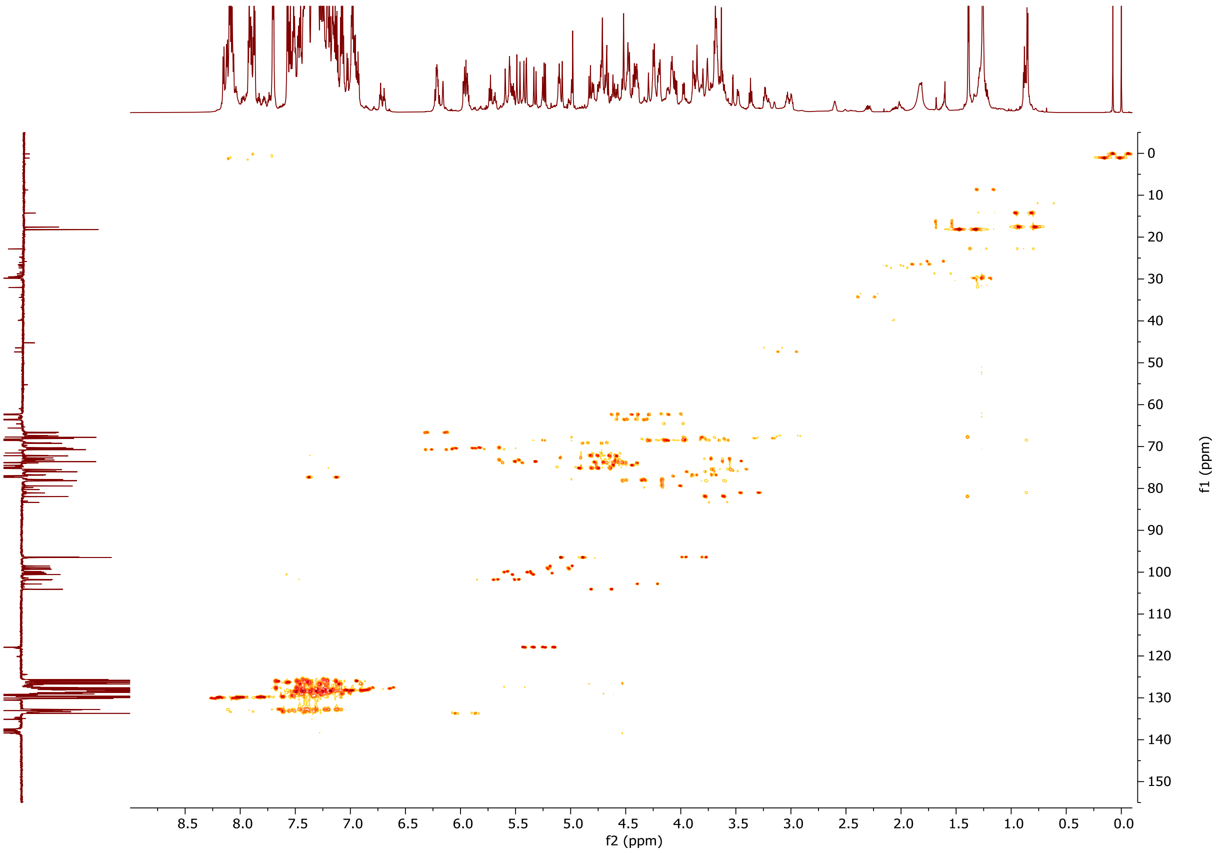
f2 (ppm)
f1 (ppm)

β-D-glucose-(1->3)-2-*O*-(α-D-mannose-(1->2))-β-D-mannose-(1->3)-β-D-mannose-(1->3)-α-L-rhamnose-(1->3)-β-D-glucose-(1->3)-2-*O*-(α-D-mannose-(1->2))-β-D-mannose-(1->3)-β-D-mannose-(1->3)-1-*O*-propyl-α-L-rhamnose (15)

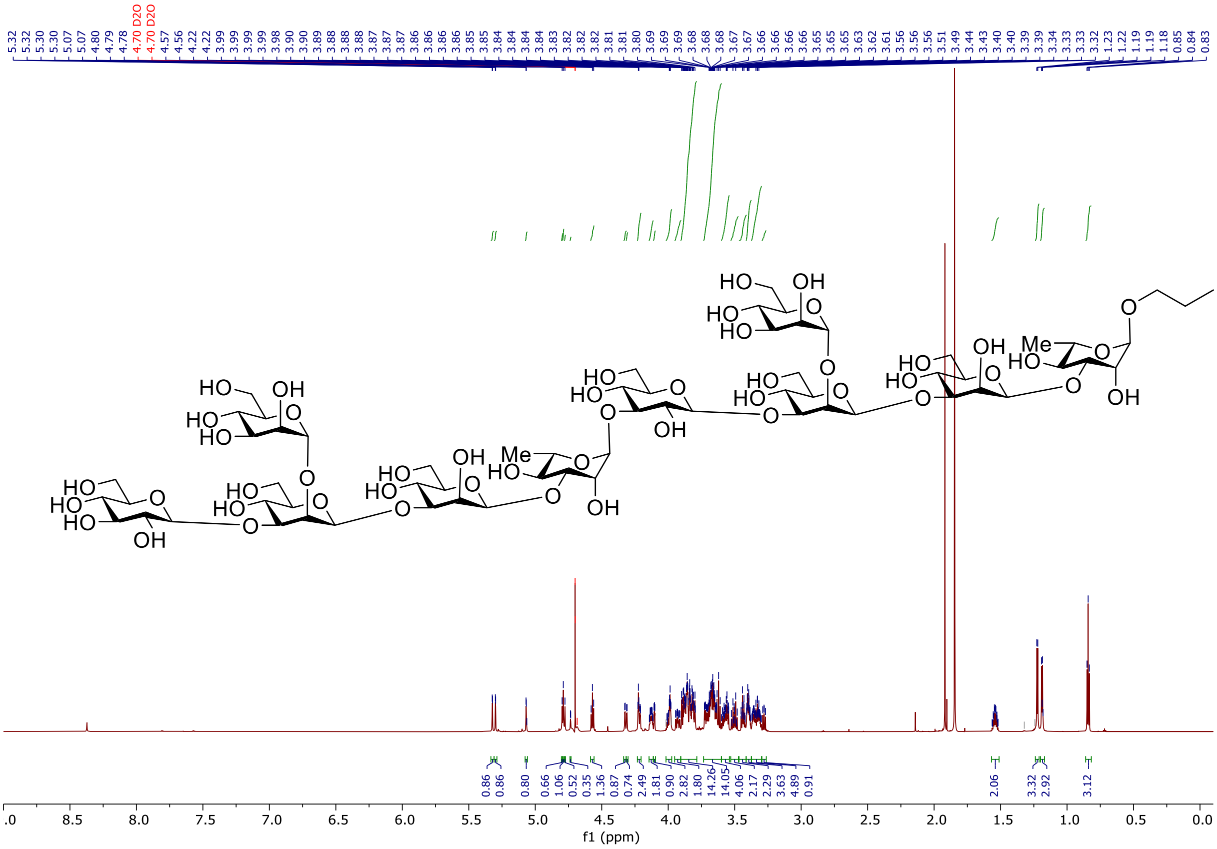

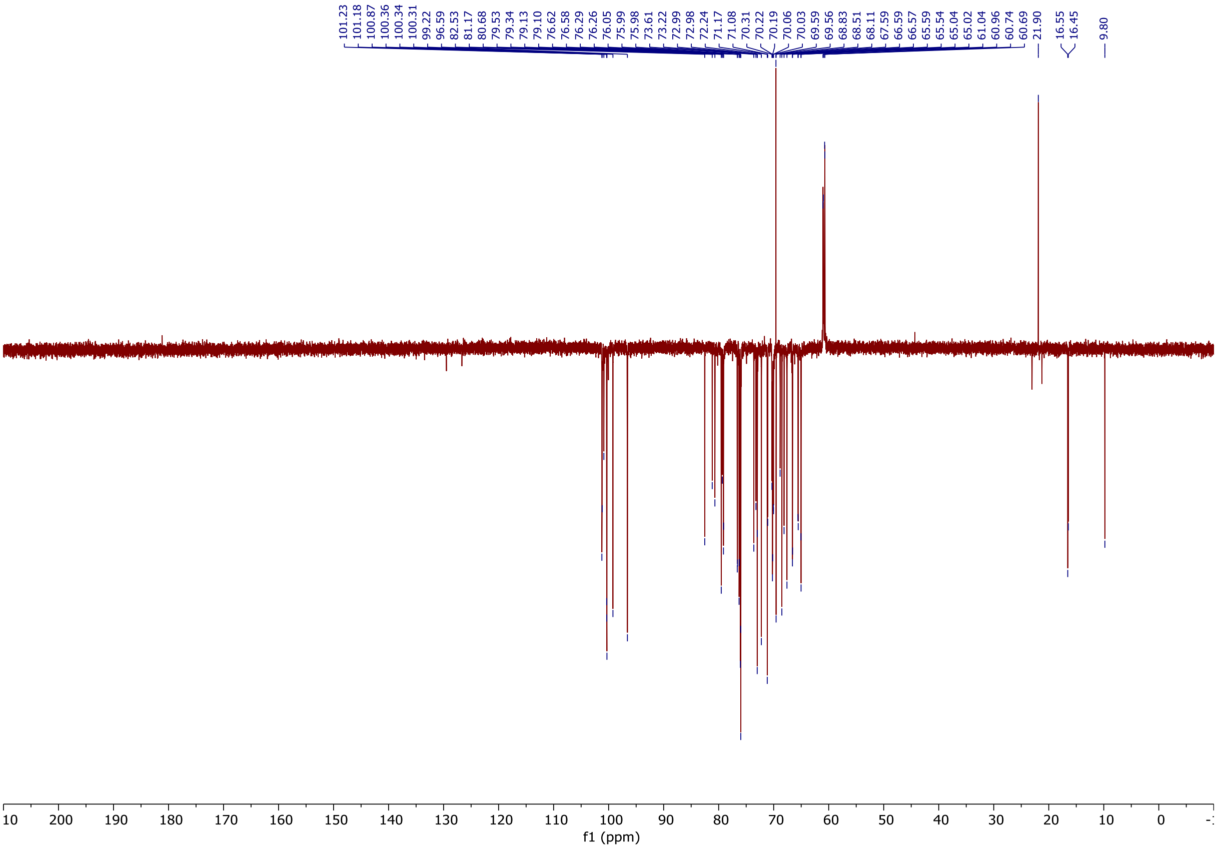

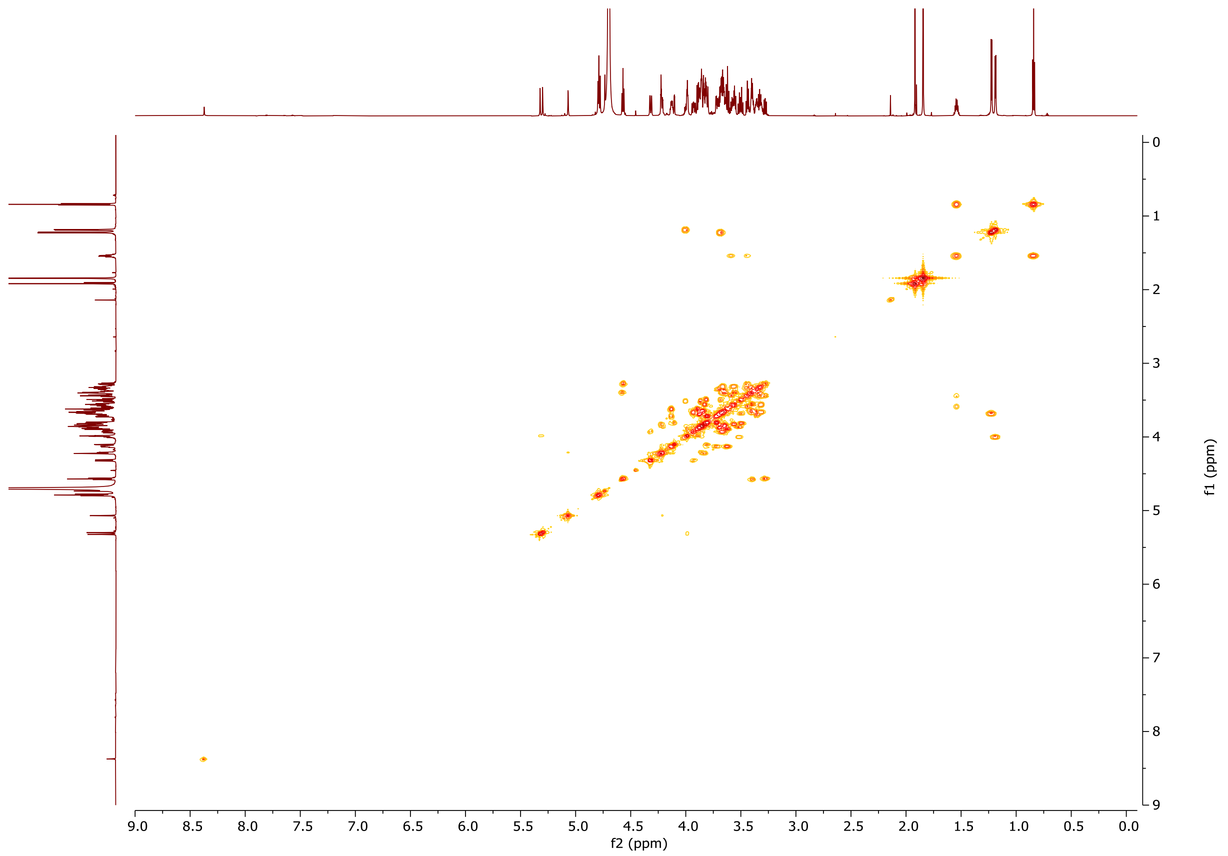

f2 (ppm)
f1 (ppm)

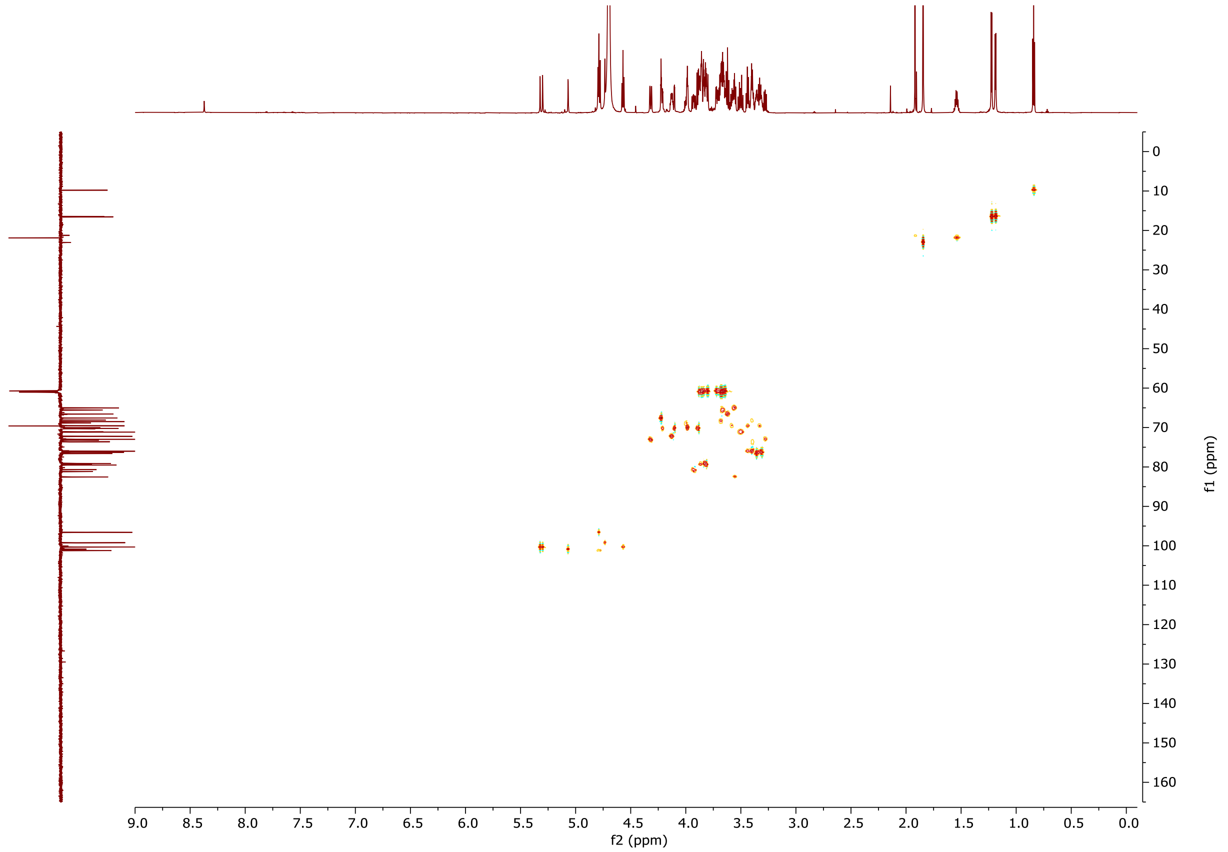

f2 (ppm)
f1 (ppm)

2-*O*-benzyl-4,6-*O*-benzylidene-3-*O*-naphthyl-β-ᴅ-mannose-(1->3)-2-*O*-benzoyl-4-*O*-benzyl-α-ʟ-rhamnose (93)

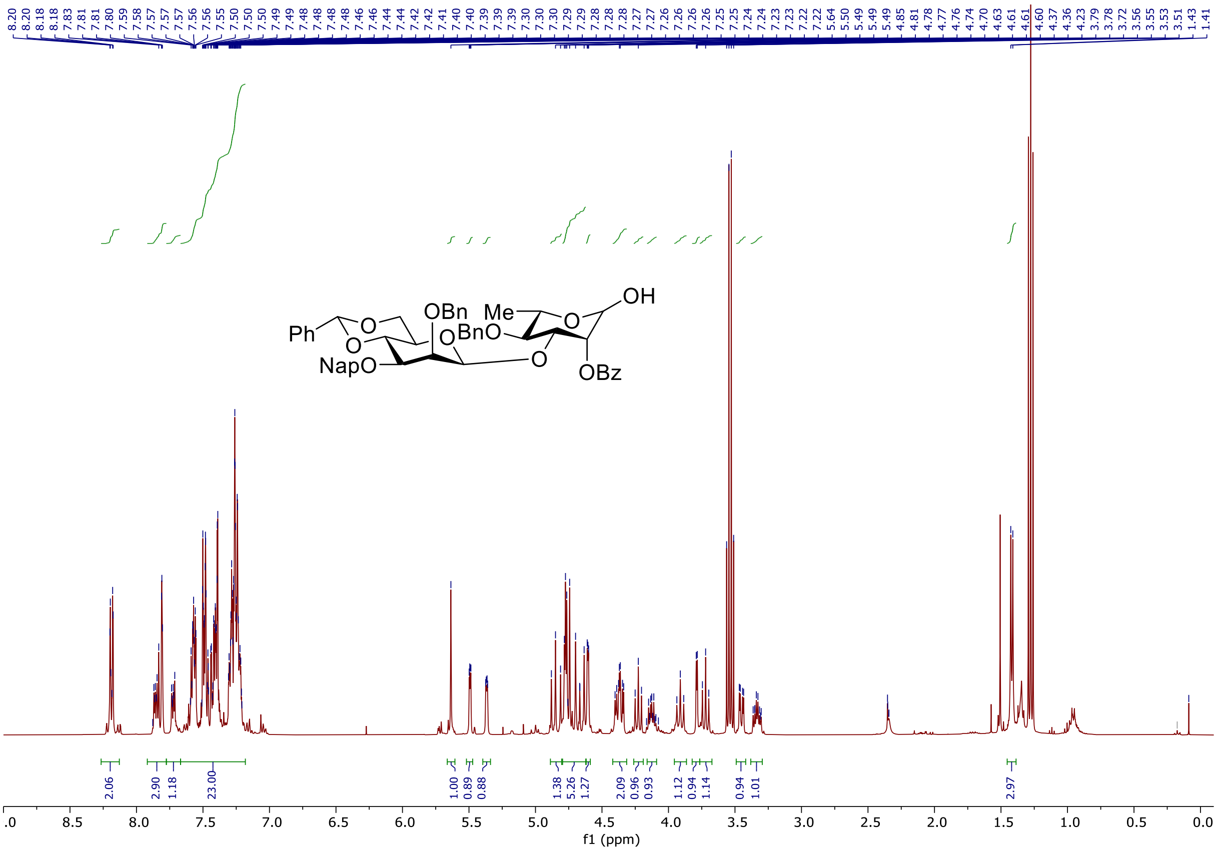

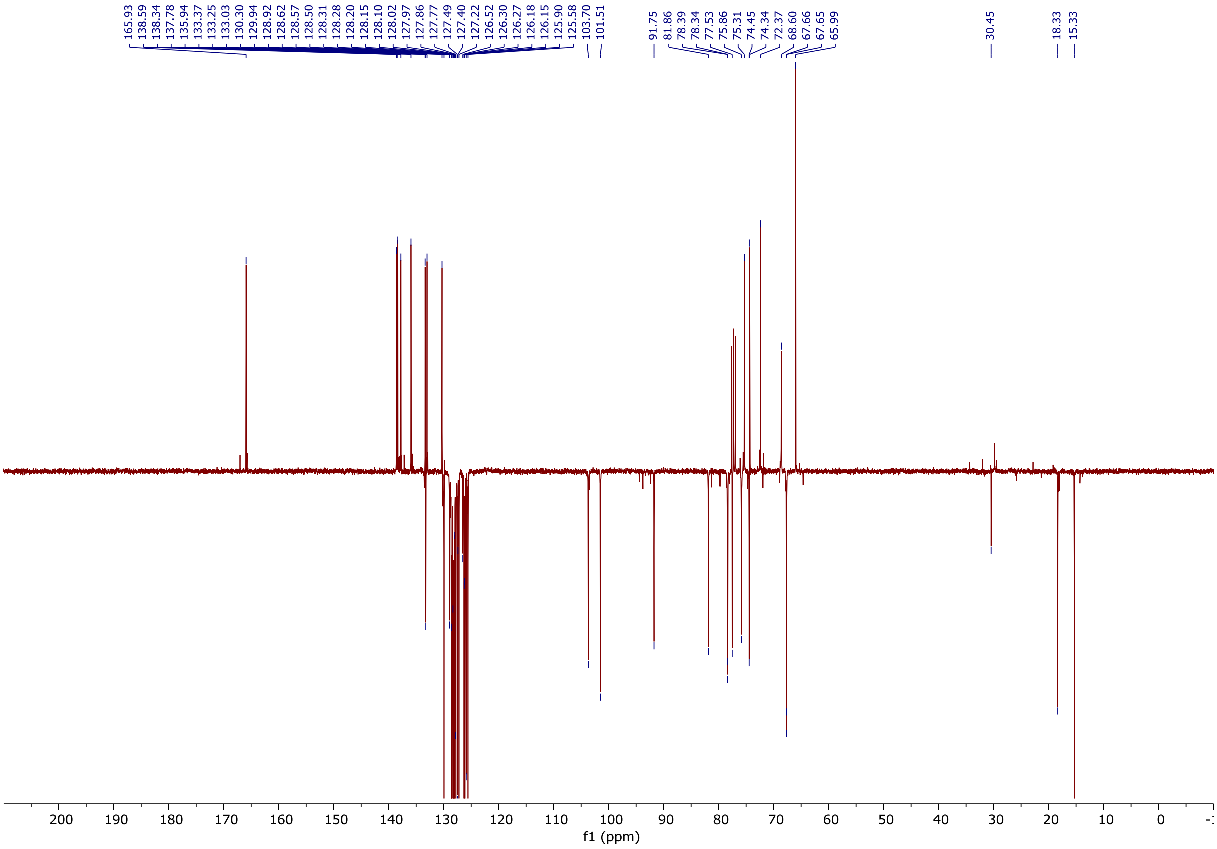

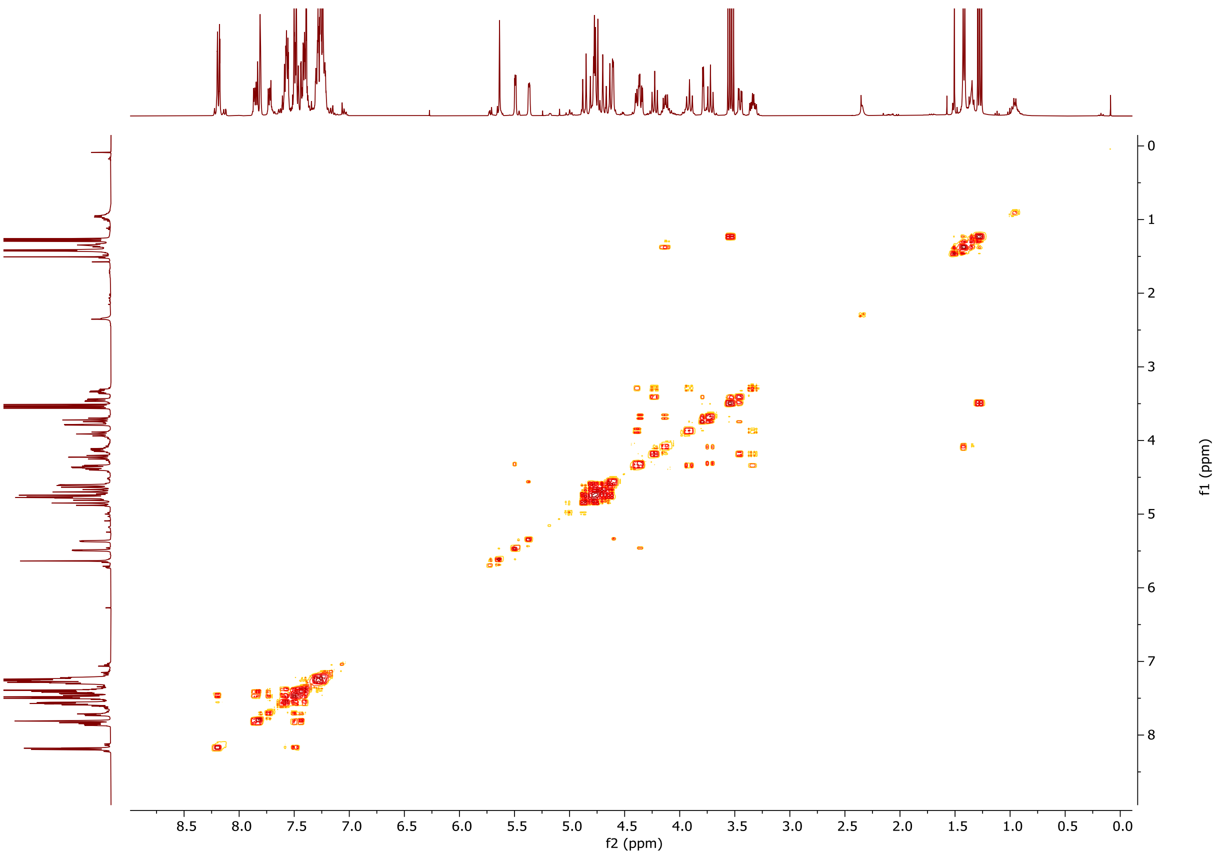

0
1
2
3
4
5
6
7
8
f1 (ppm)
8.5 8.0 7.5 7.0 6.5 6.0 5.5 5.0 4.5 4.0 3.5 3.0 2.5 2.0 1.5 1.0 0.5 0.0
f2 (ppm)

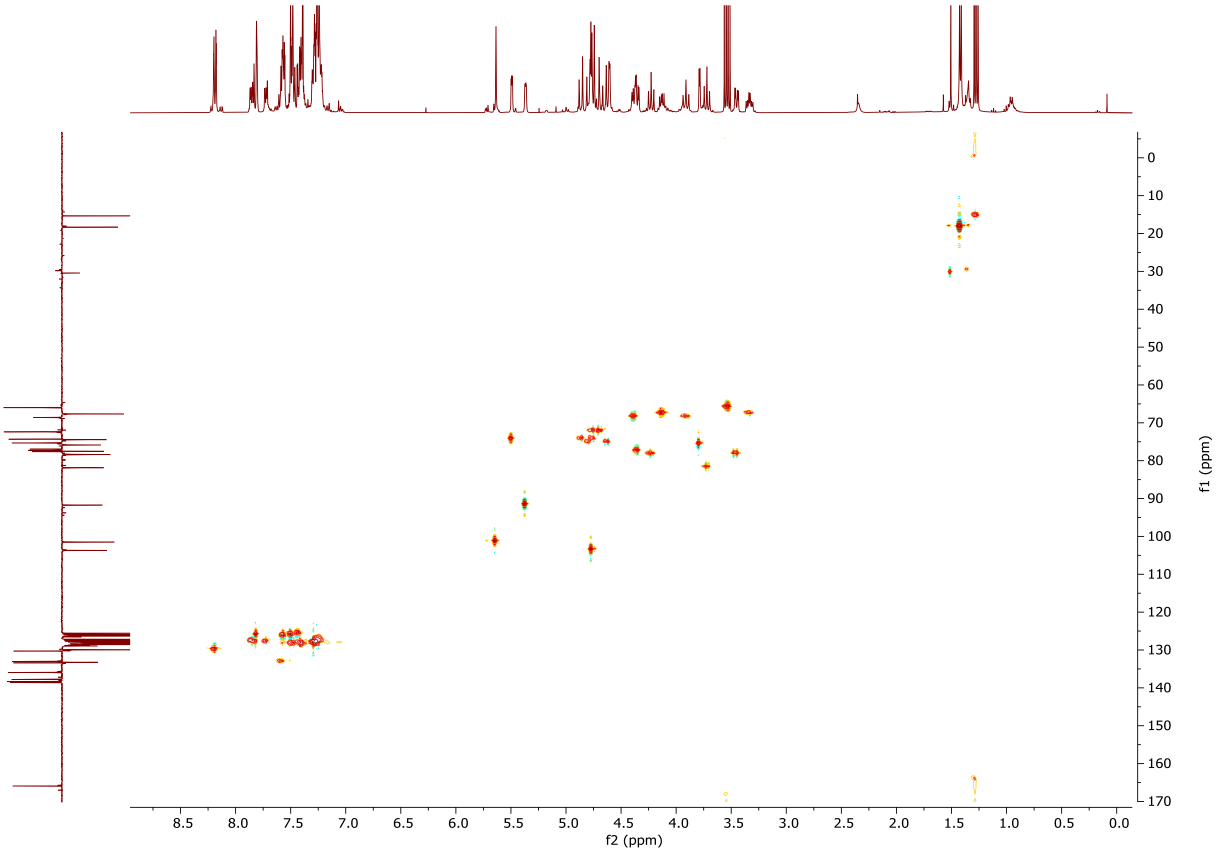

0
10
20
30
40
50
60
70
80
90
100
110
120
130
140
150
160
170
f1 (ppm)
8.5 8.0 7.5 7.0 6.5 6.0 5.5 5.0 4.5 4.0 3.5 3.0 2.5 2.0 1.5 1.0 0.5 0.0
f2 (ppm)

2-*O*-benzyl-4,6-*O*-benzylidene-3-*O*-naphthyl-β-D-mannose-(1->3)-2-*O*-benzoyl-4-*O*-benzyl-1-*O*-trichloroimidate-α-L-rhamnose (94)

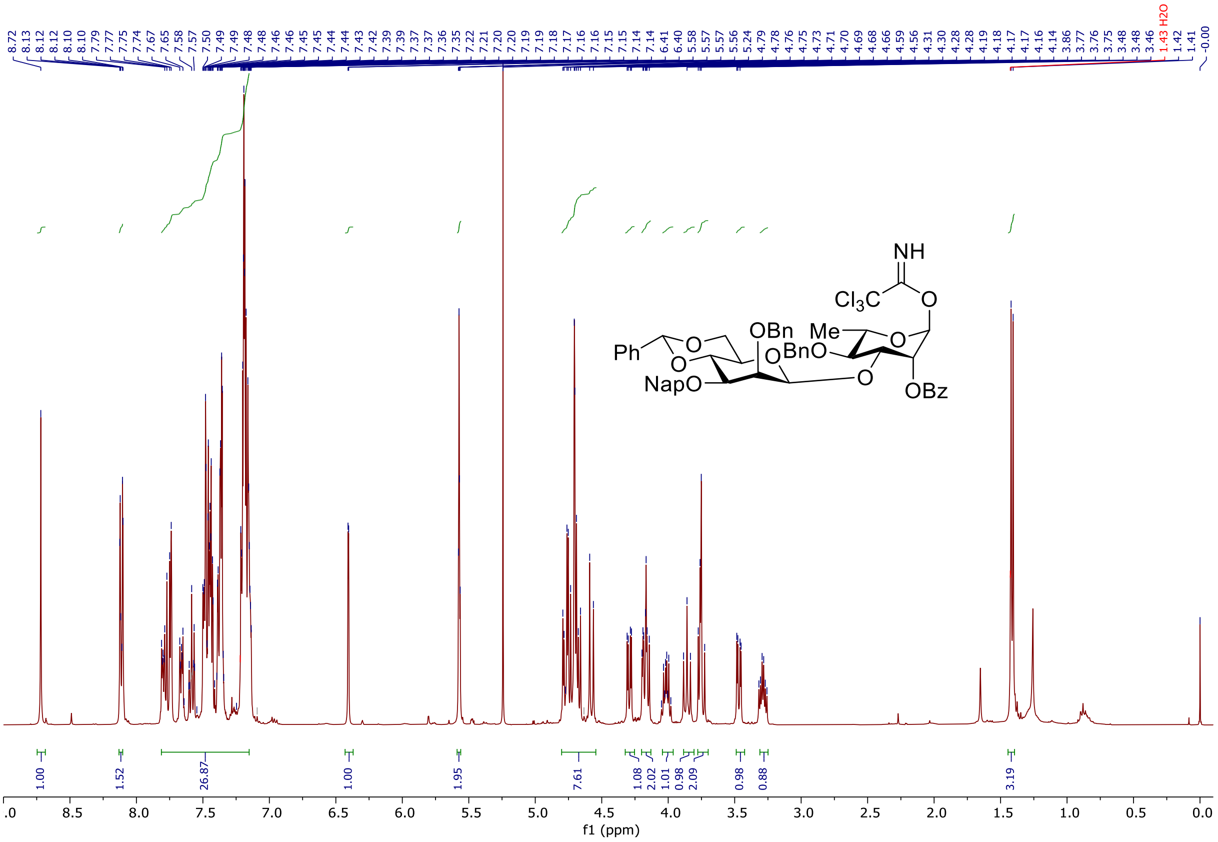

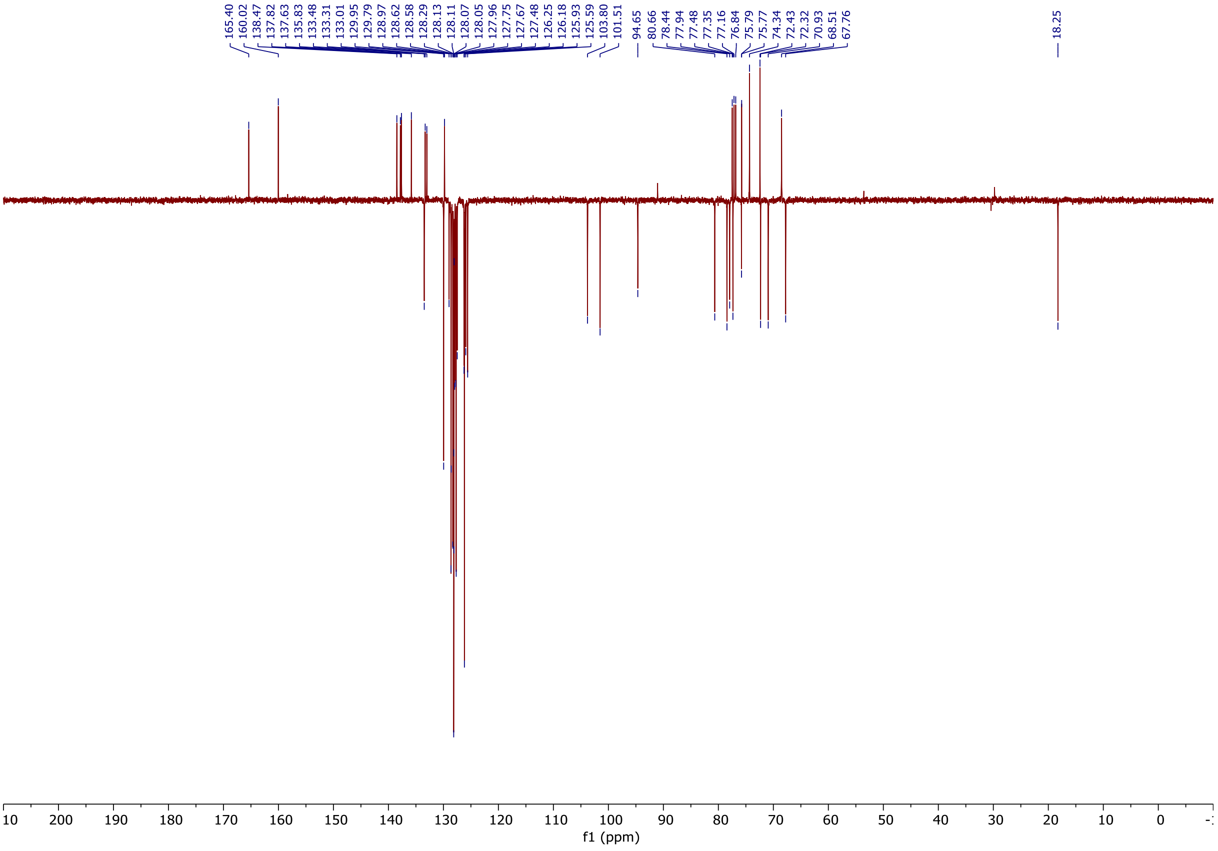

2-*O*-benzyl-4,6-*O*-benzylidene-3-*O*-naphthyl-β-D-mannose-(1->3)-2-*O*-benzoyl-4-*O*-benzyl-α-L-rhamnose-(1->3)-2-*O*-benzyl-6-*O*-t-butyldiphenylsilyl-3-*O*-t-butyldimethylsilyl-β-D-glucose-cyclophellitol (95)

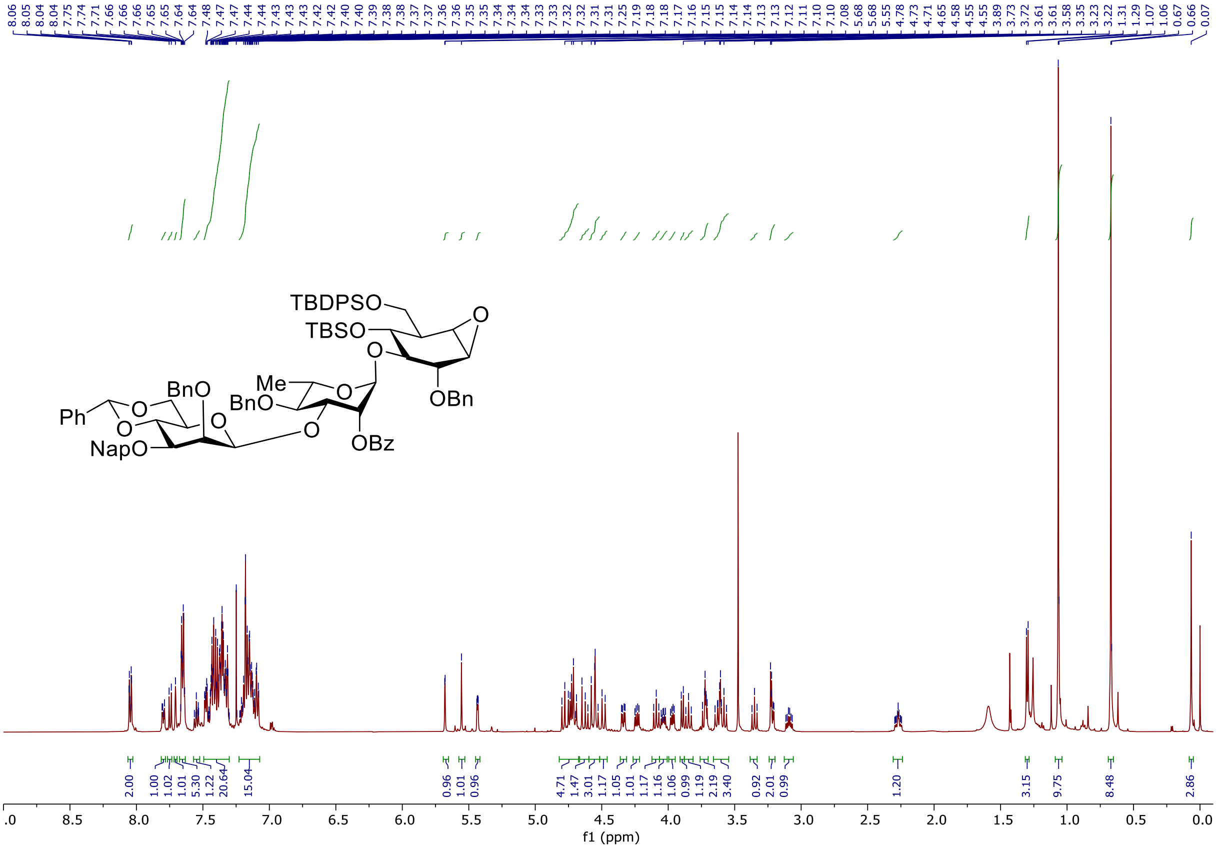

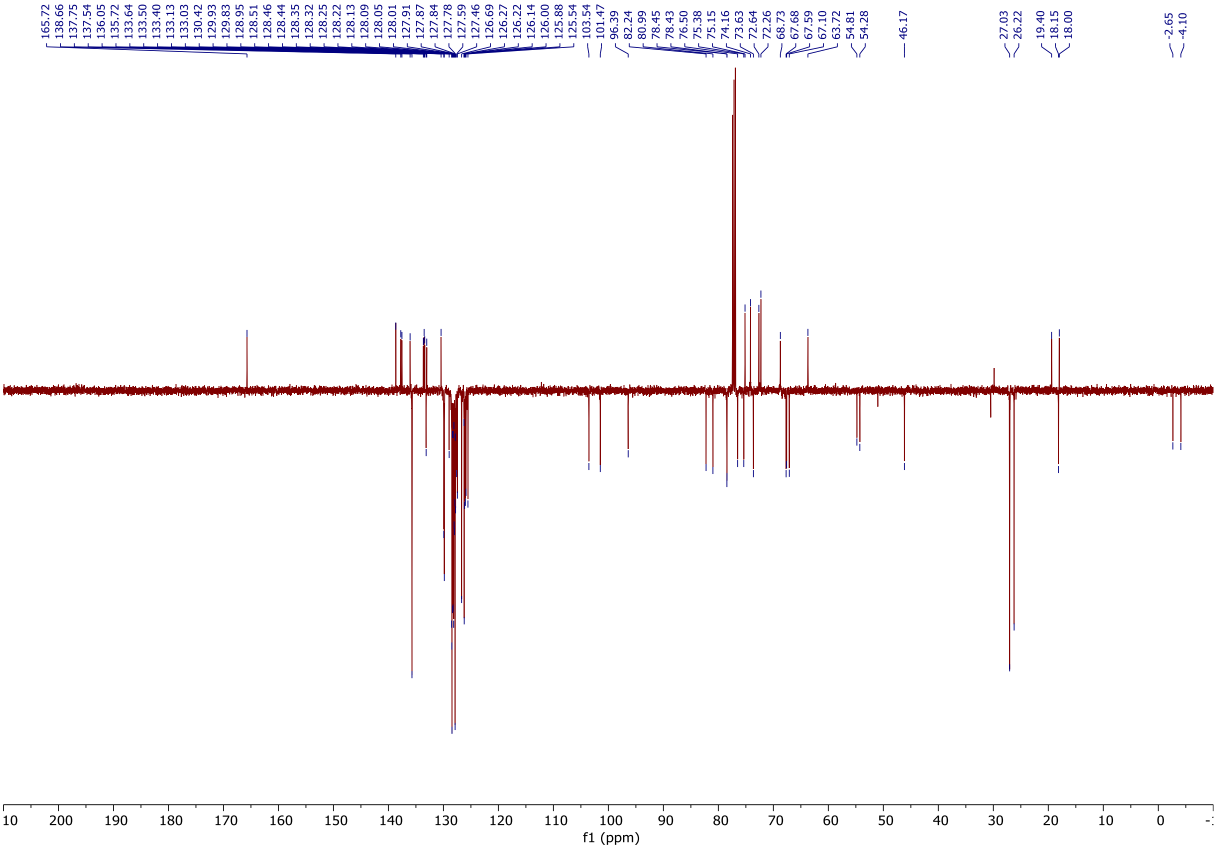

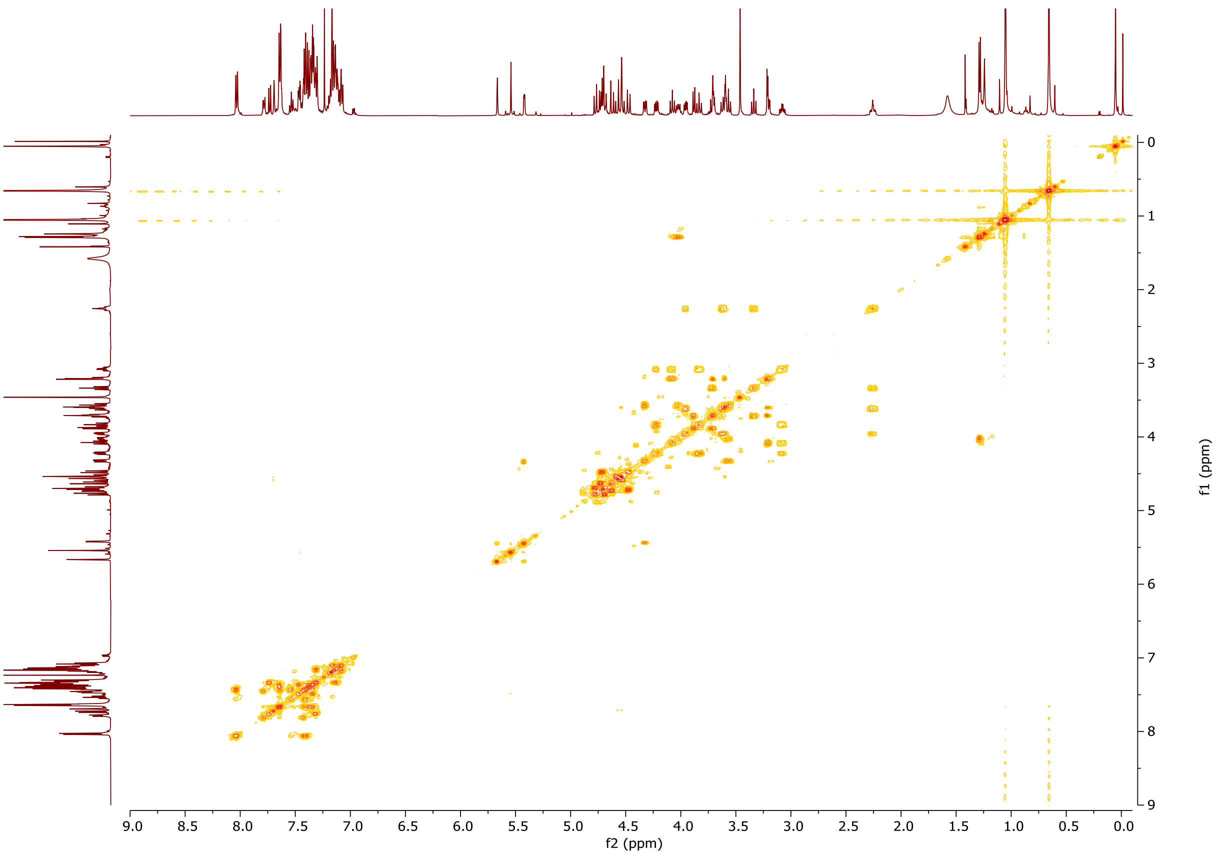
9.0
8.5
8.0
7.5
7.0
6.5
6.0
5.5
5.0
4.5
4.0
3.5
3.0
2.5
2.0
1.5
1.0
0.5
0.0
f2 (ppm)
f1 (ppm)

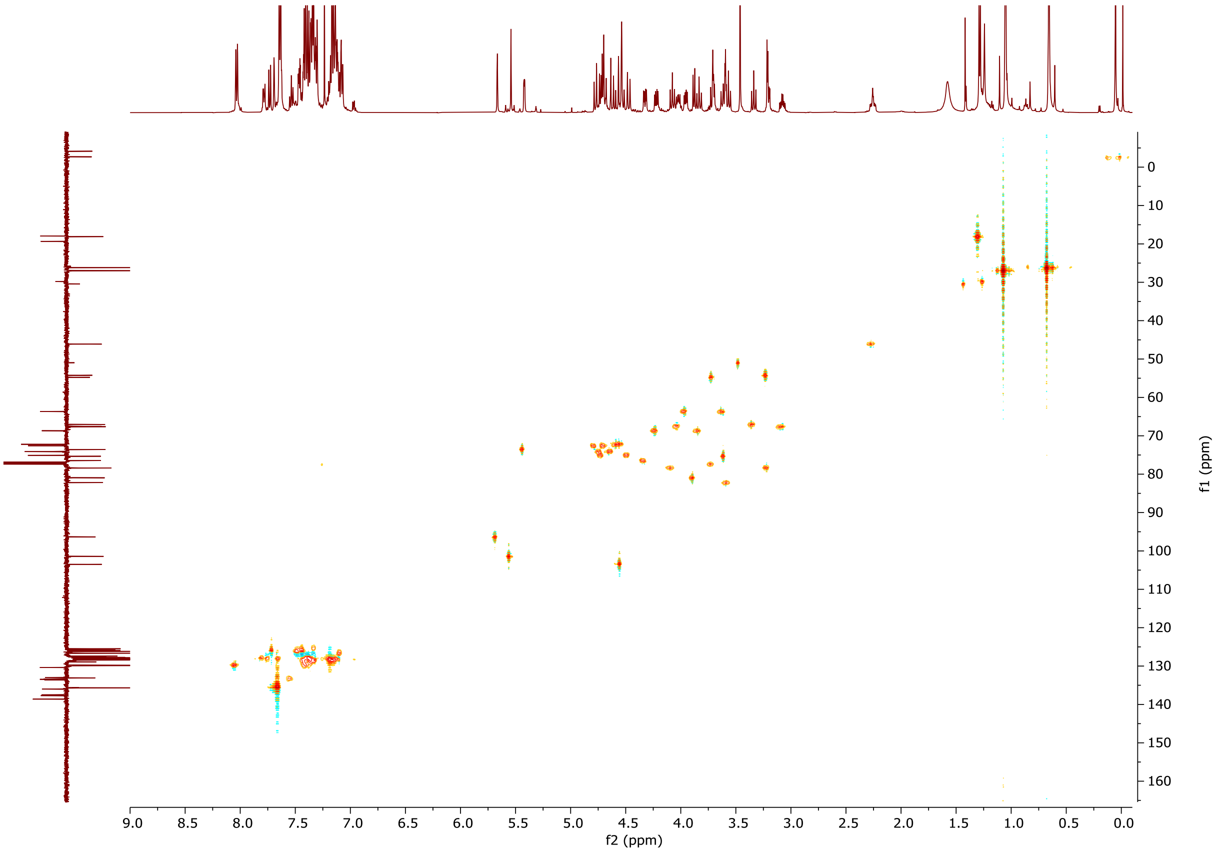
9.0
8.5
8.0
7.5
7.0
6.5
6.0
5.5
5.0
4.5
4.0
3.5
3.0
2.5
2.0
1.5
1.0
0.5
0.0
f2 (ppm)
f1 (ppm)

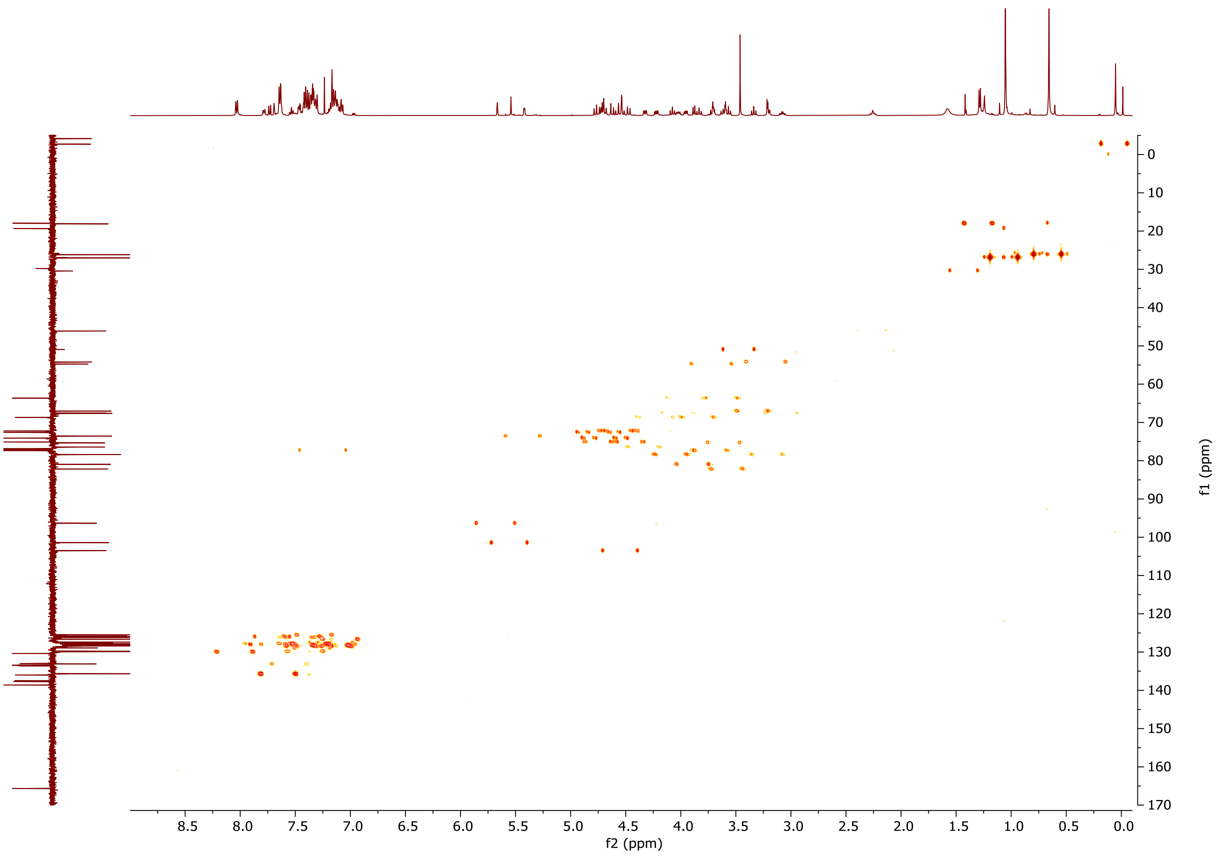

β-D-mannose-(1->3)-α-L-rhamnose-(1->3)-β-D-glucose-cyclophellitol (18)

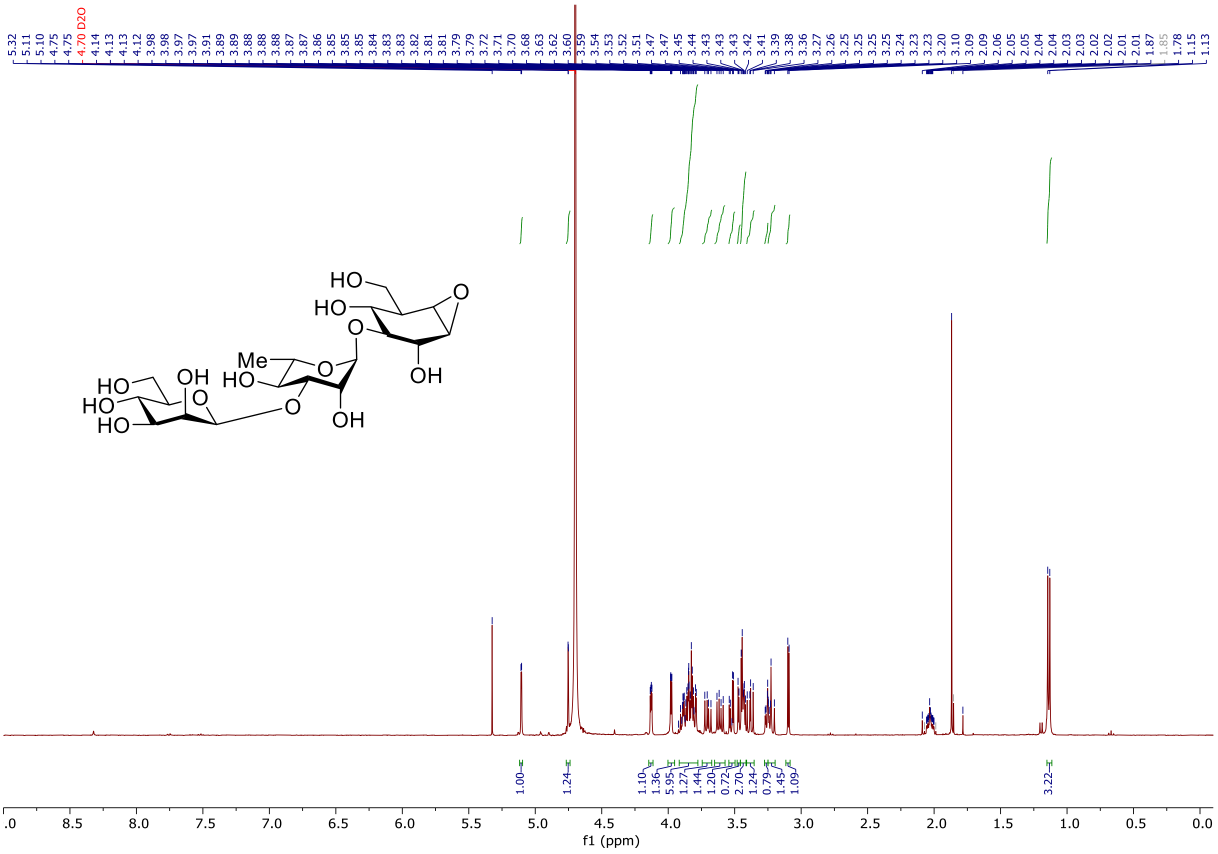

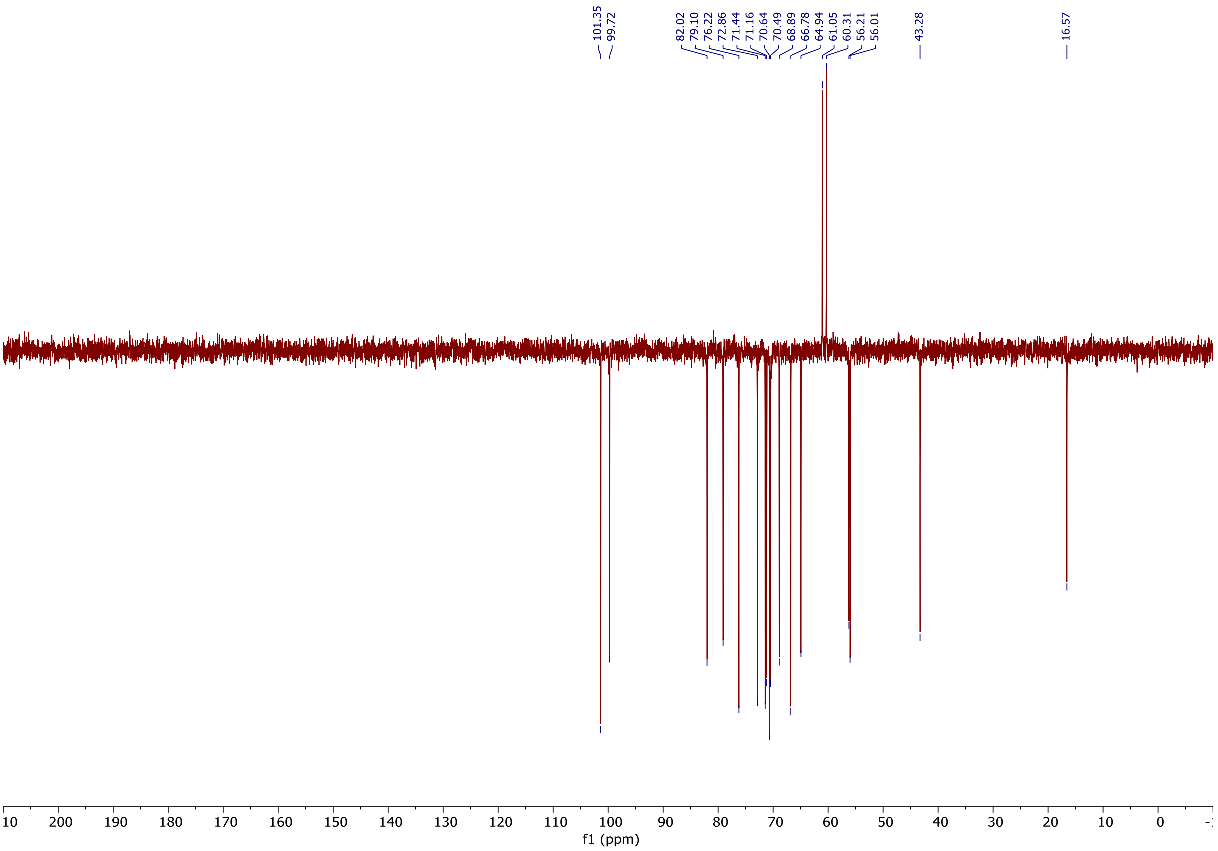
101.35
99.72
82.02
79.10
76.22
72.86
71.44
71.16
70.64
70.49
68.89
66.78
64.94
61.05
60.31
56.21
56.01
43.28
16.57
f1 (ppm)

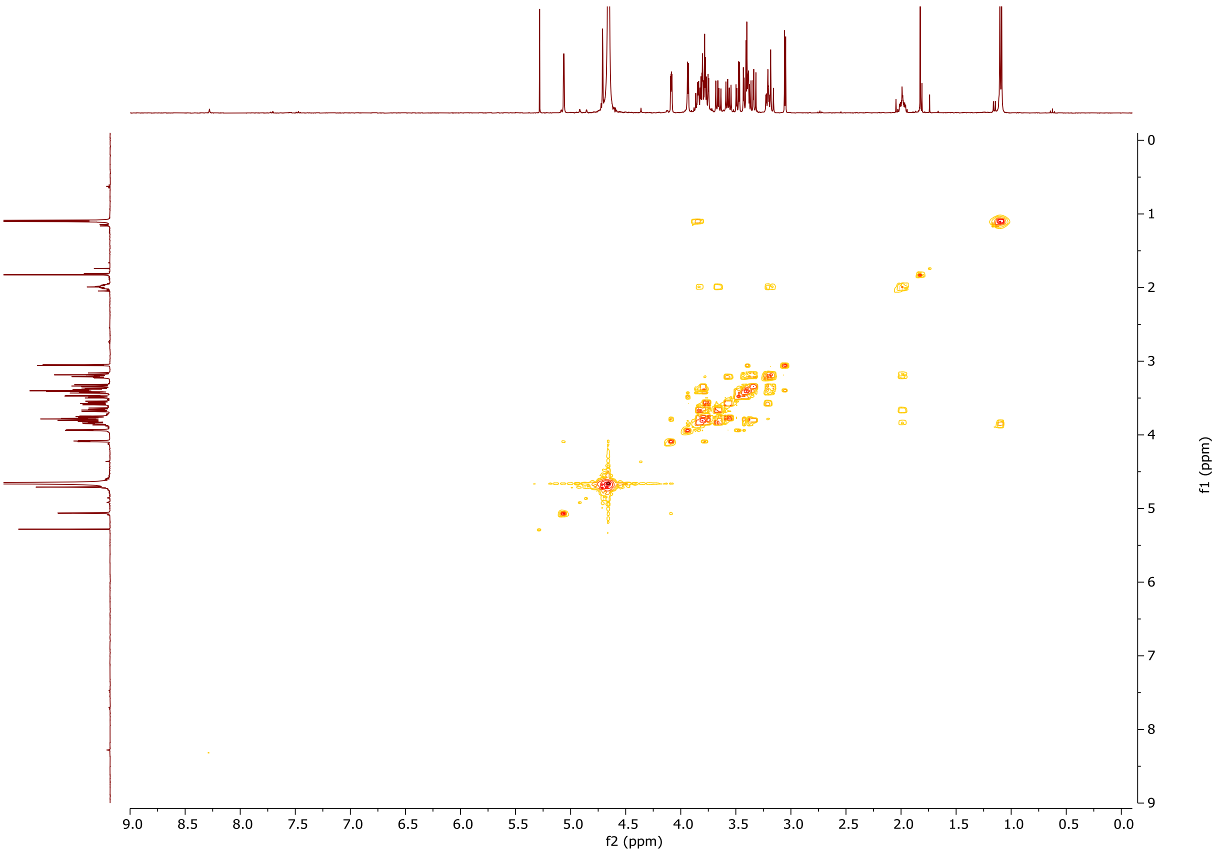
f2 (ppm)
f1 (ppm)

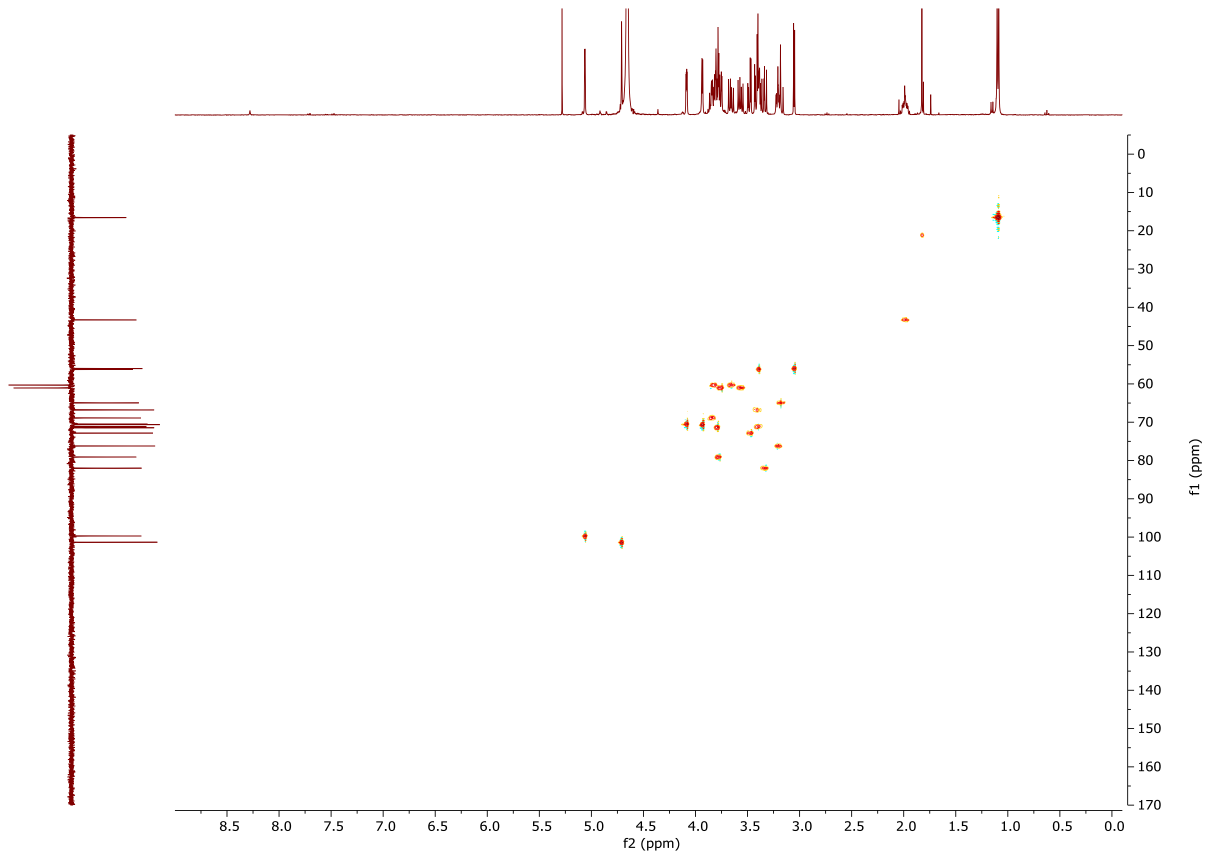

2-*O*-benzoyl-4,6-*O*-benzylidene-3-*O*-naphthyl-β-D-mannose-(1->3)-2-*O*-benzyl-4,6-*O*-benzylidene-β-D-mannose-(1->3)-1-*O*-allyl-2-*O*-benzoyl-4-*O*-benzyl-α-L-rhamnose (97)

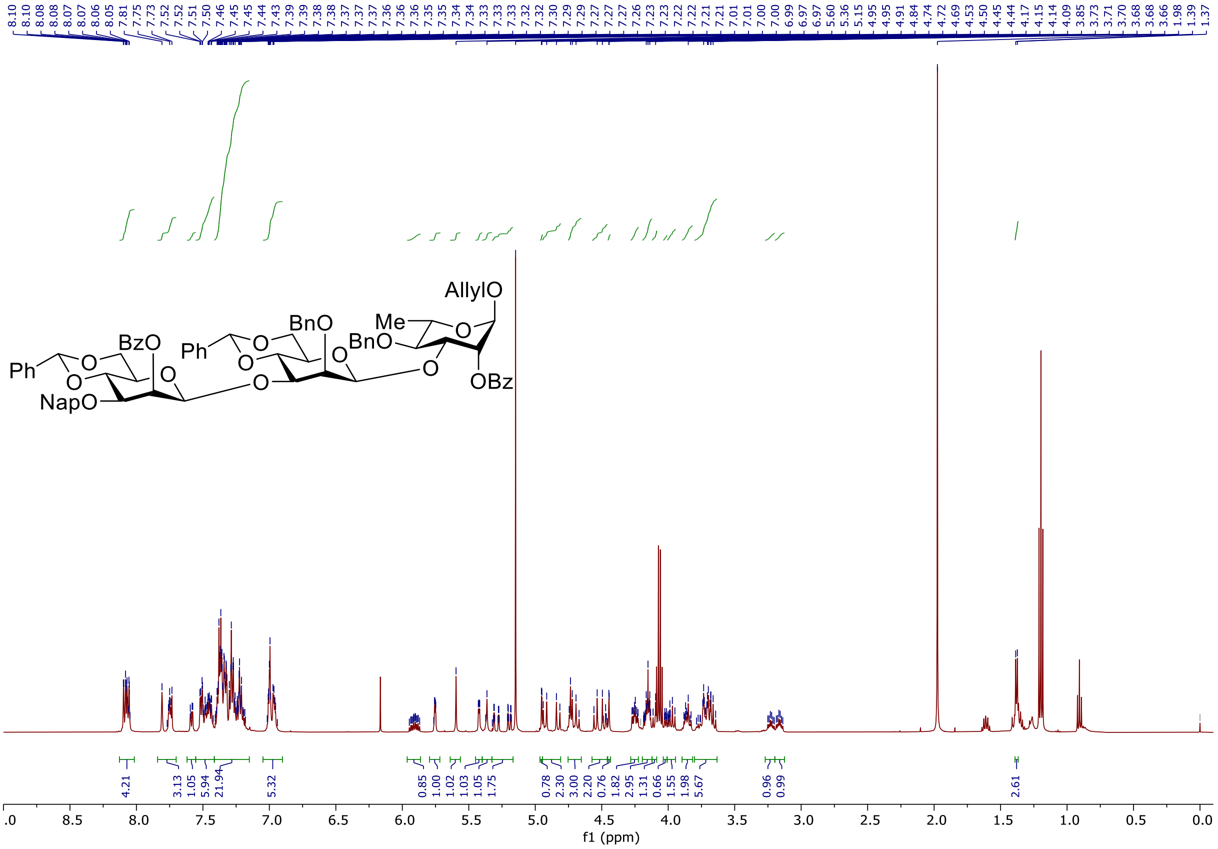

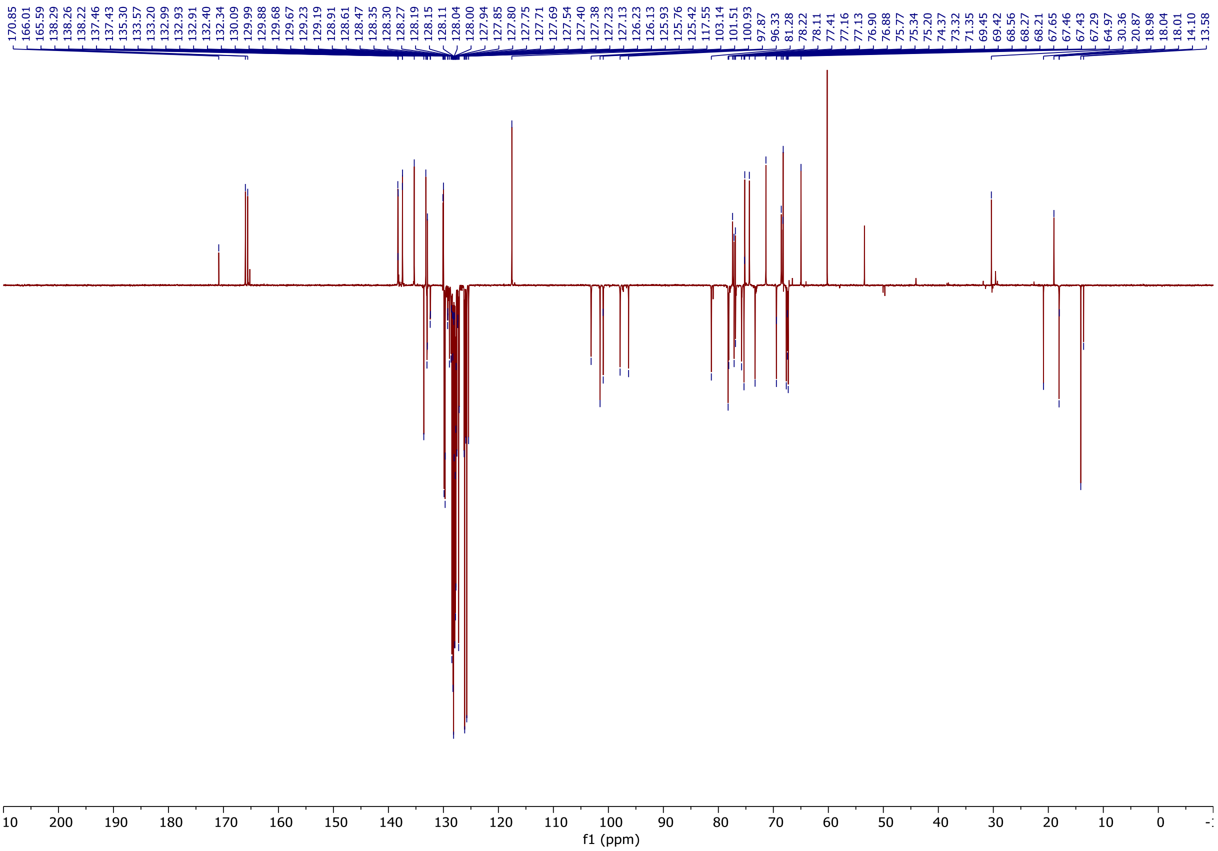

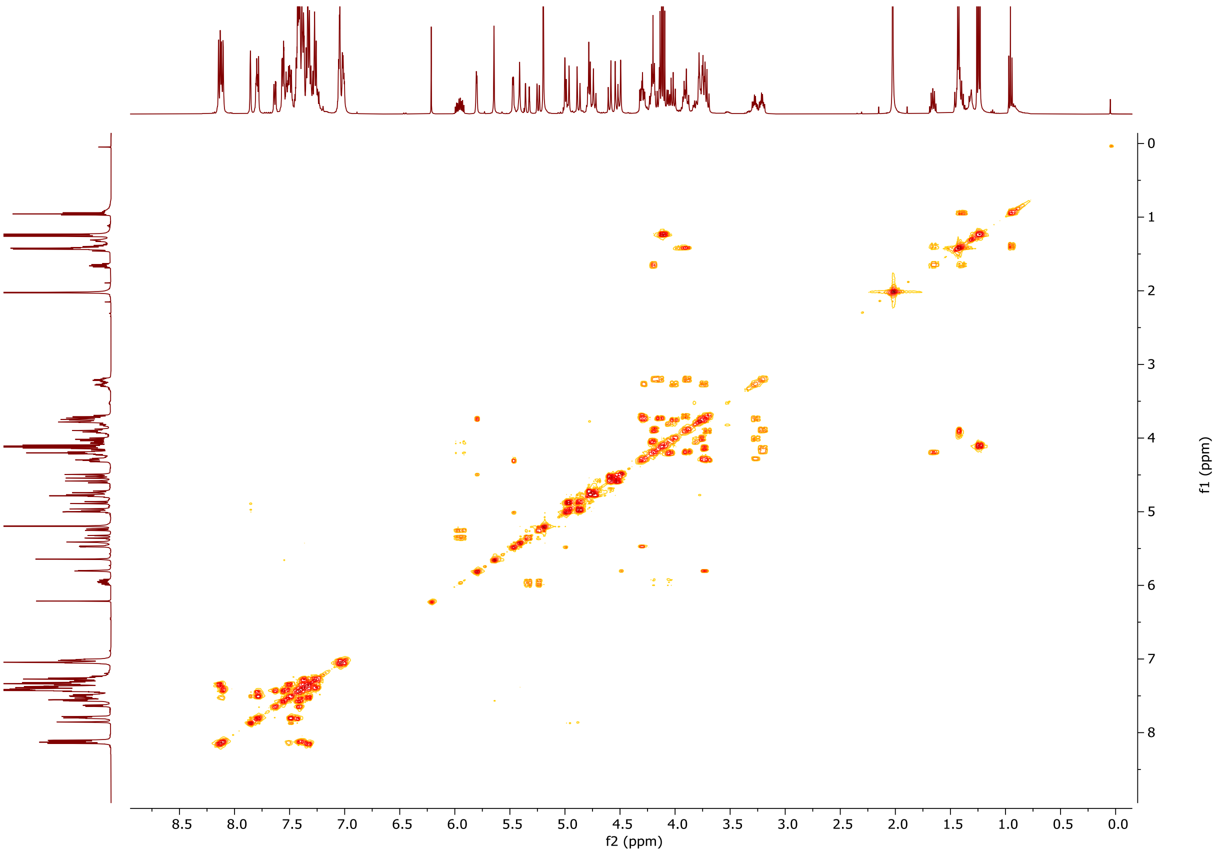

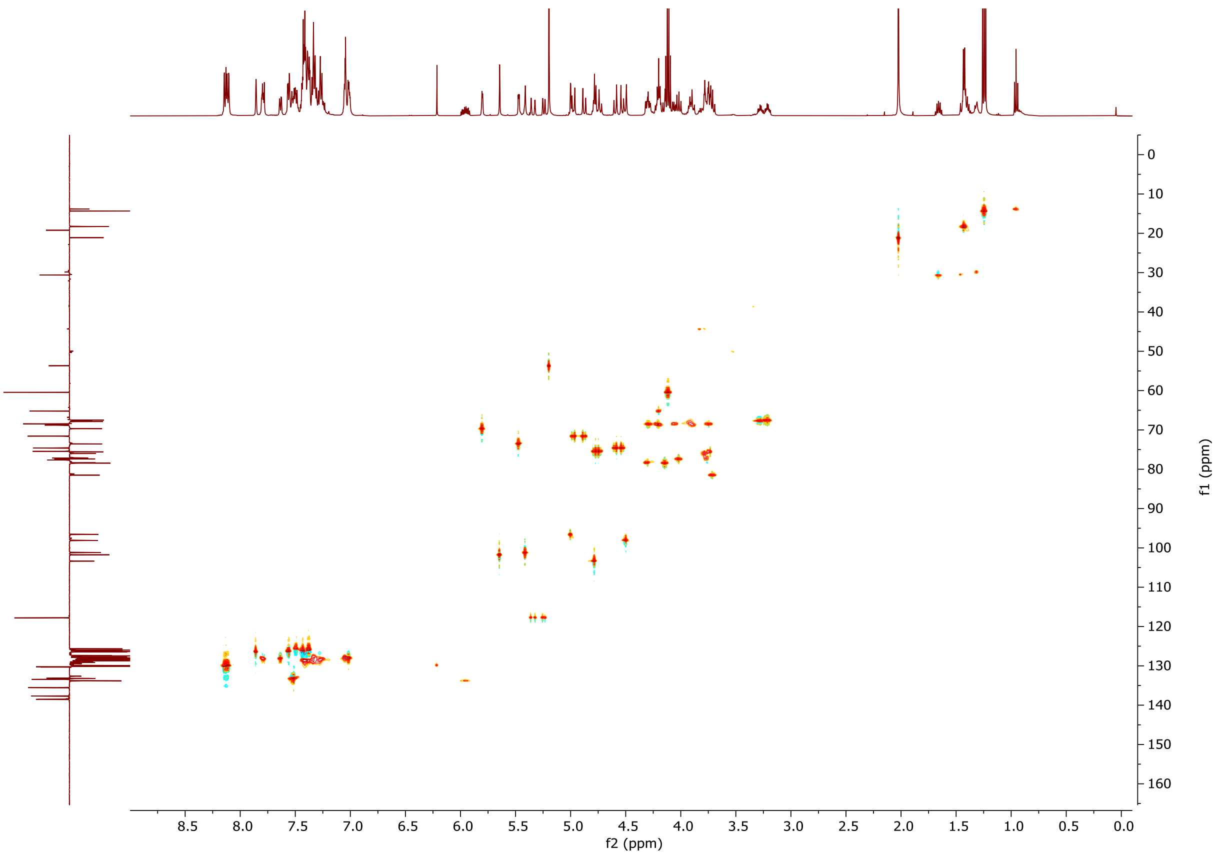

2-*O*-benzoyl-4,6-*O*-benzylidene-3-*O*-naphthyl-β-D-mannose-(1->3)-2-*O*-benzyl-4,6-*O*-benzylidene-β-D-mannose-(1->3)-2-*O*-benzoyl-4-*O*-benzyl-α-L-rhamnose-(1->3)-2-*O*-benzyl-6-*O*-t-butyldiphenylsilyl-3-*O*-t-butyldimethylsilyl-β-D-glucose-cyclophellitol (100)

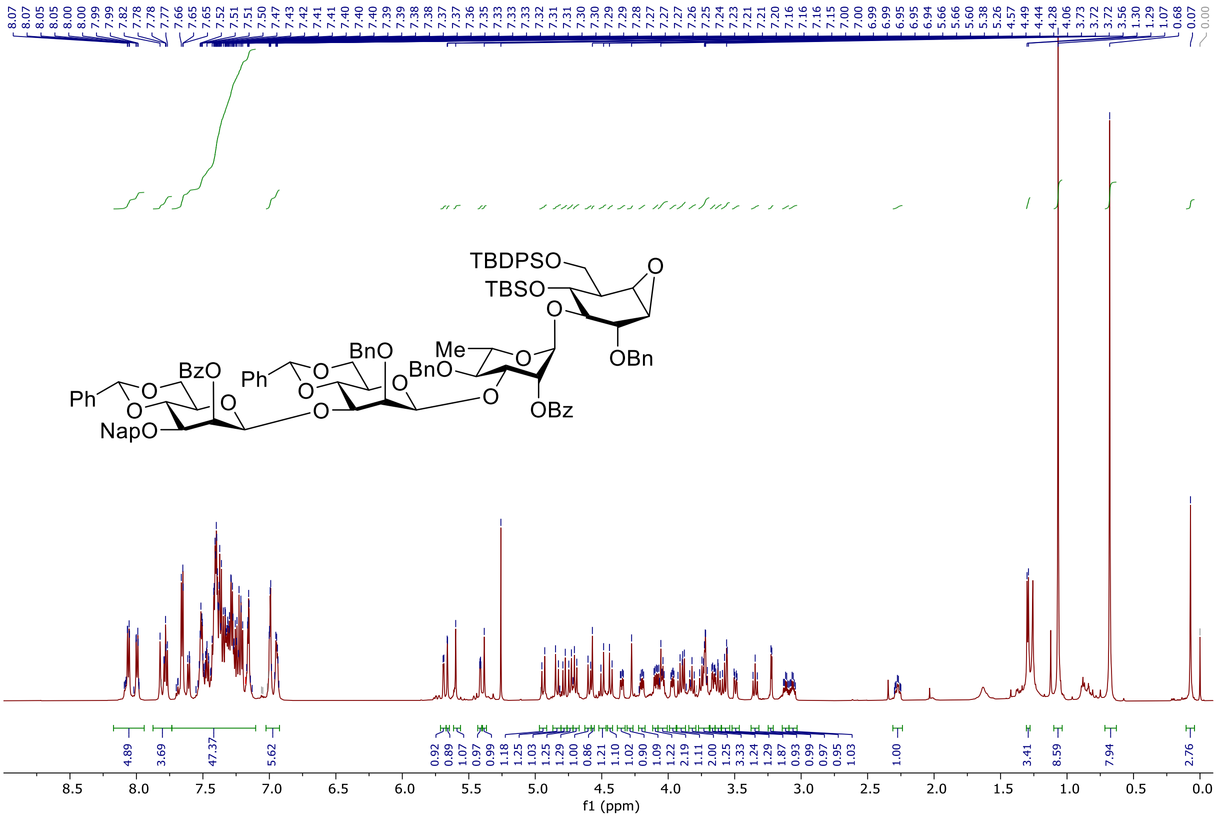

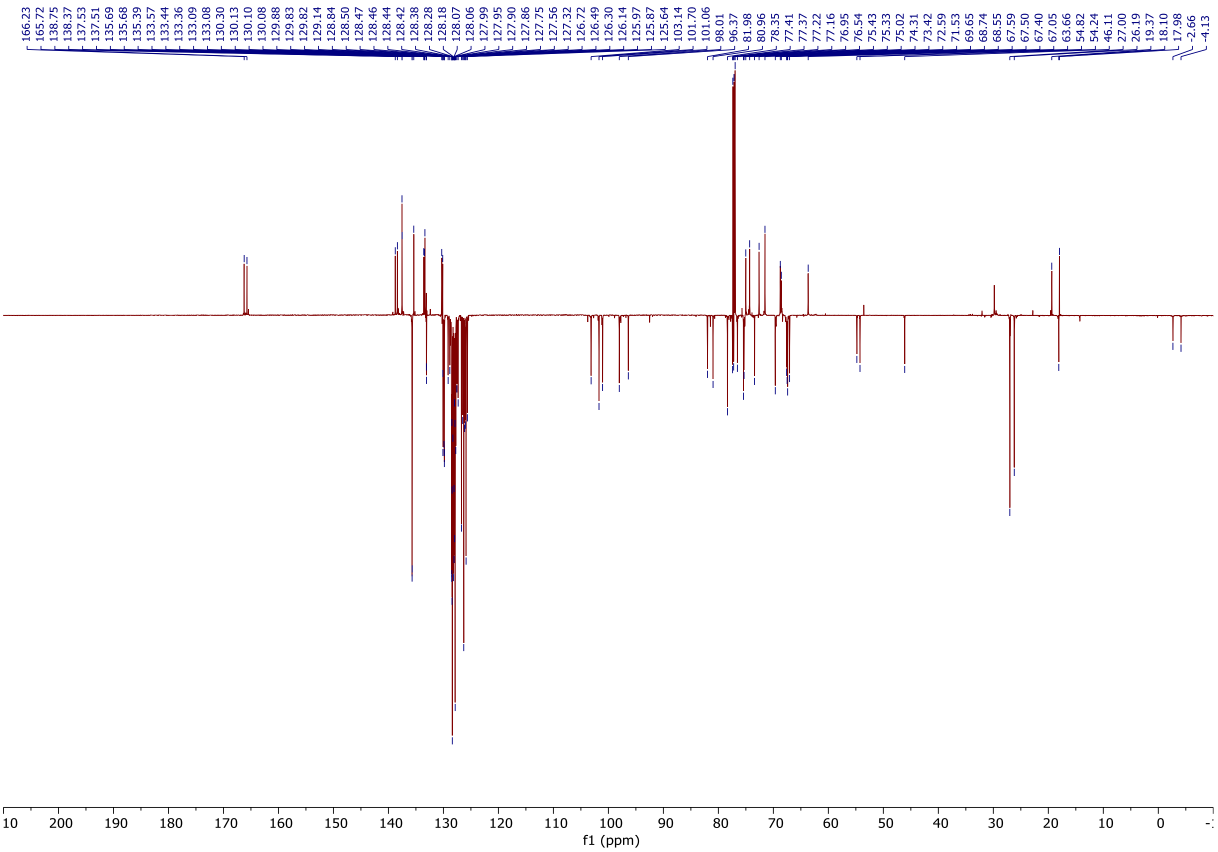

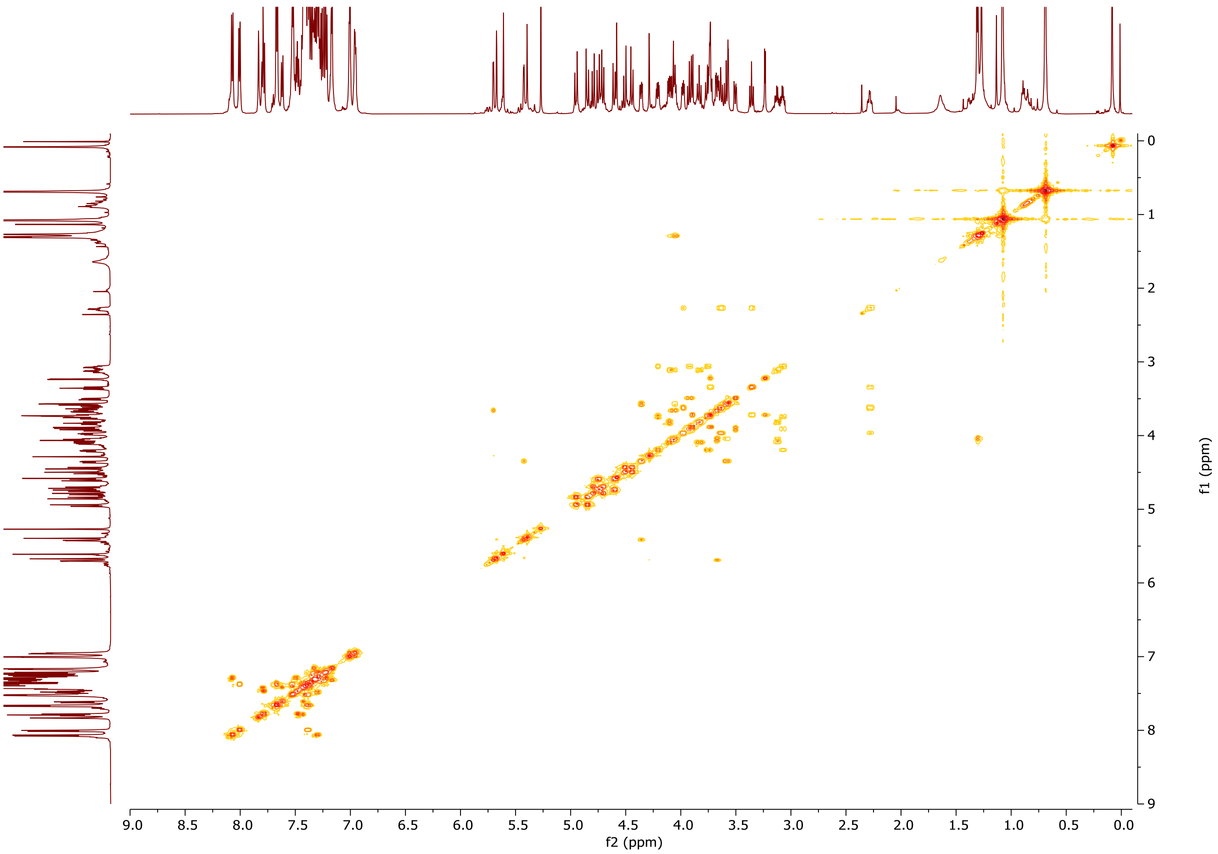

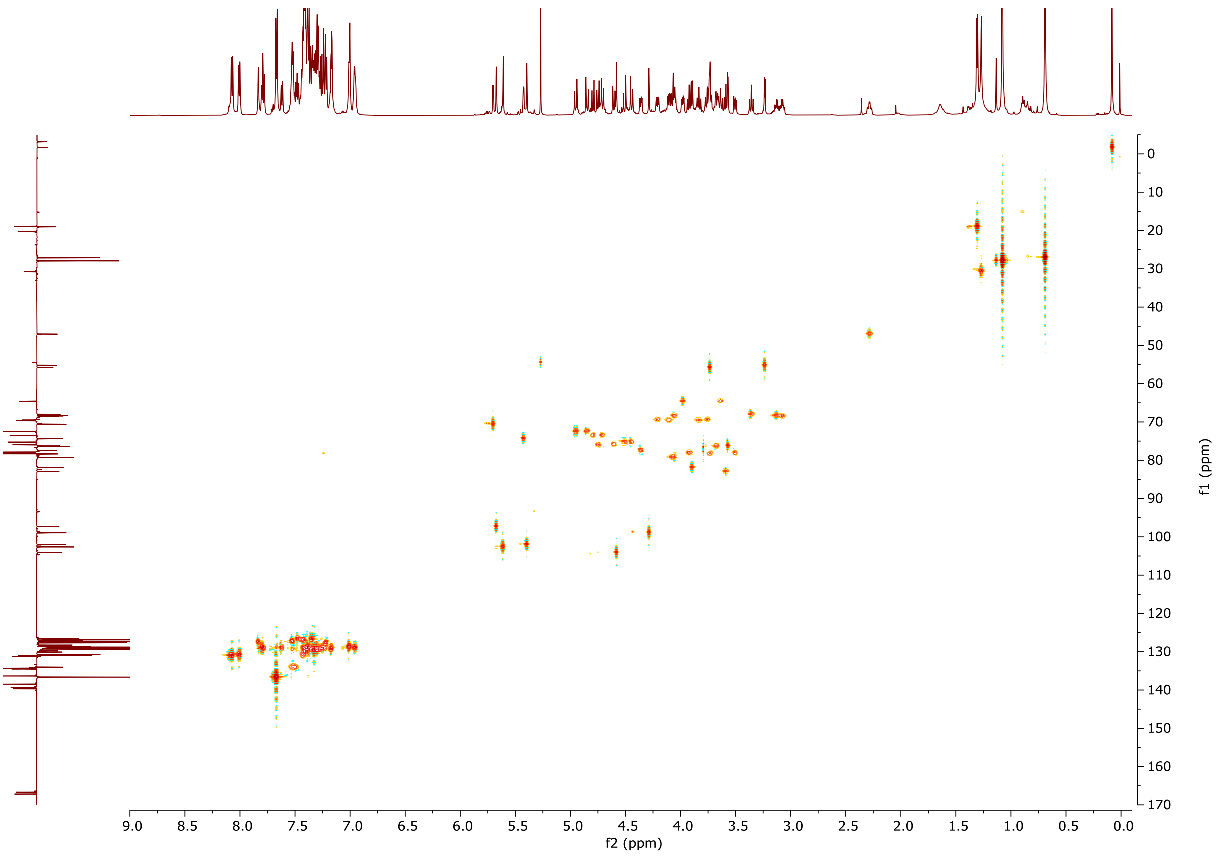

β-D-mannose-(1->3)-β-D-mannose-(1->3)-α-L-rhamnose-(1->3)-β-D-glucose-cyclophellitol (19)

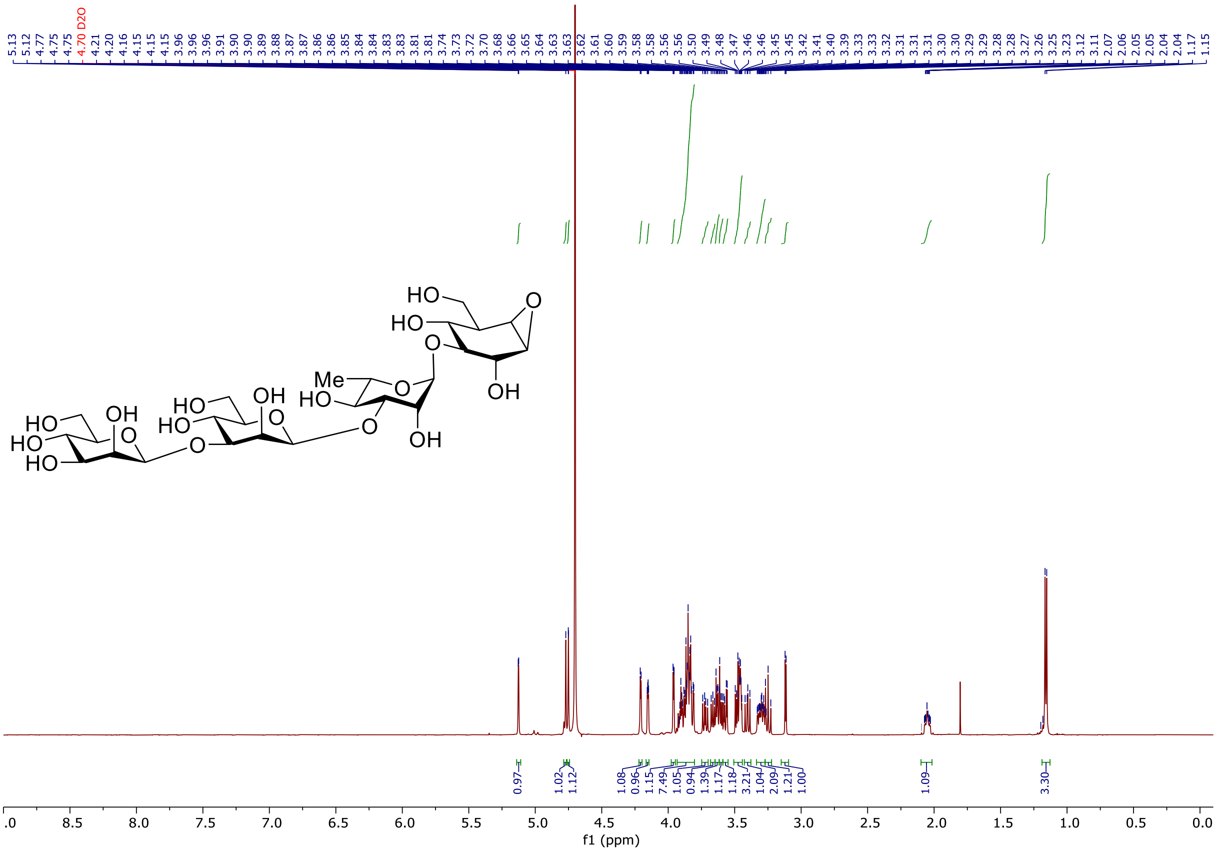

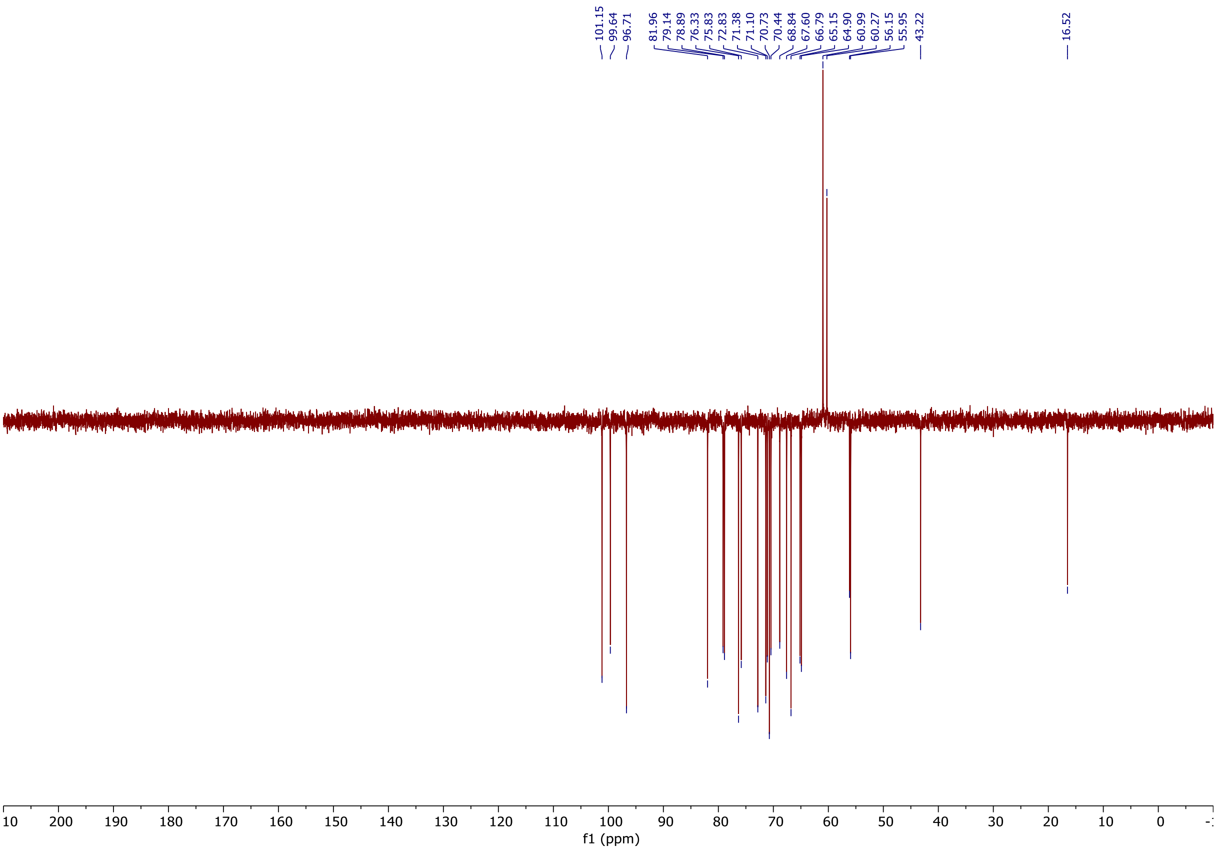
101.15
99.64
96.71
81.96
79.14
78.89
76.33
75.83
72.83
71.38
71.10
70.73
70.44
68.84
67.60
66.79
65.15
64.90
60.99
60.27
56.15
55.95
43.22
16.52
f1 (ppm)

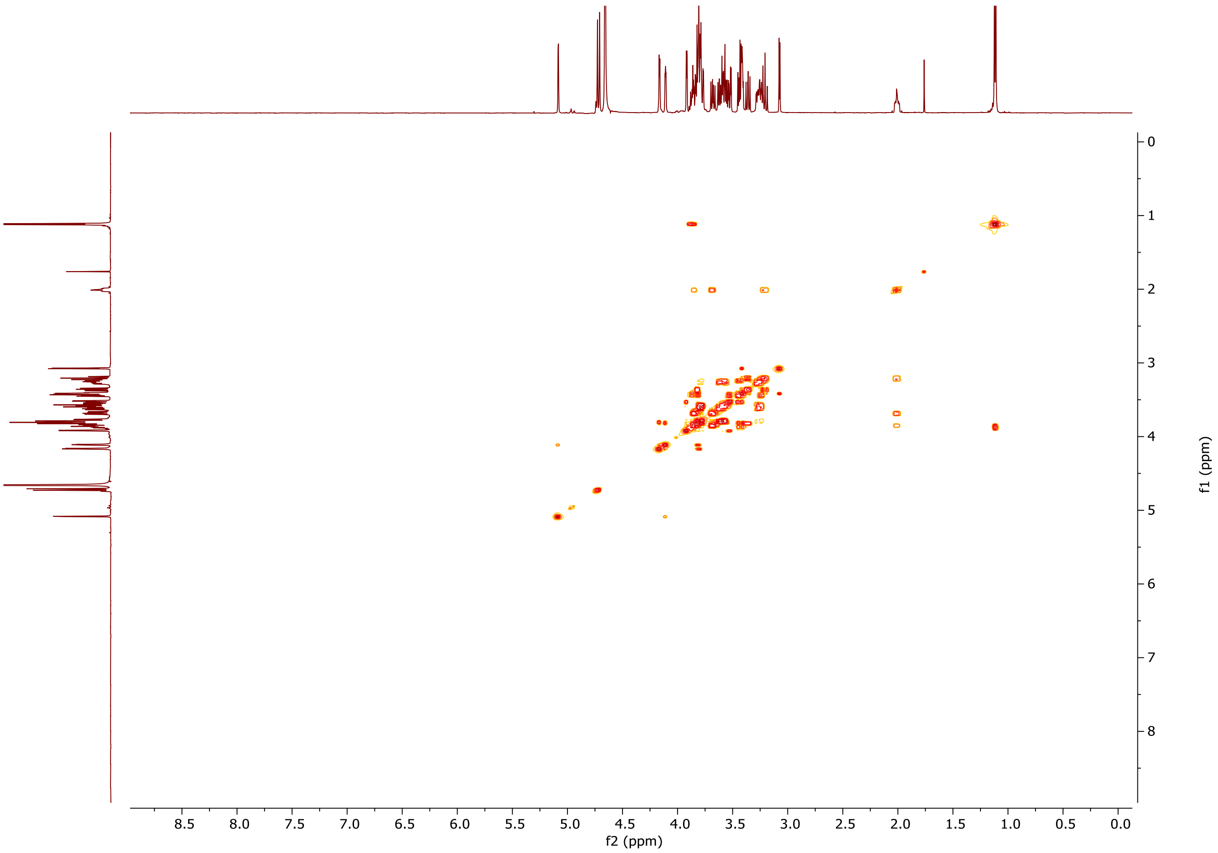
f2 (ppm)
f1 (ppm)

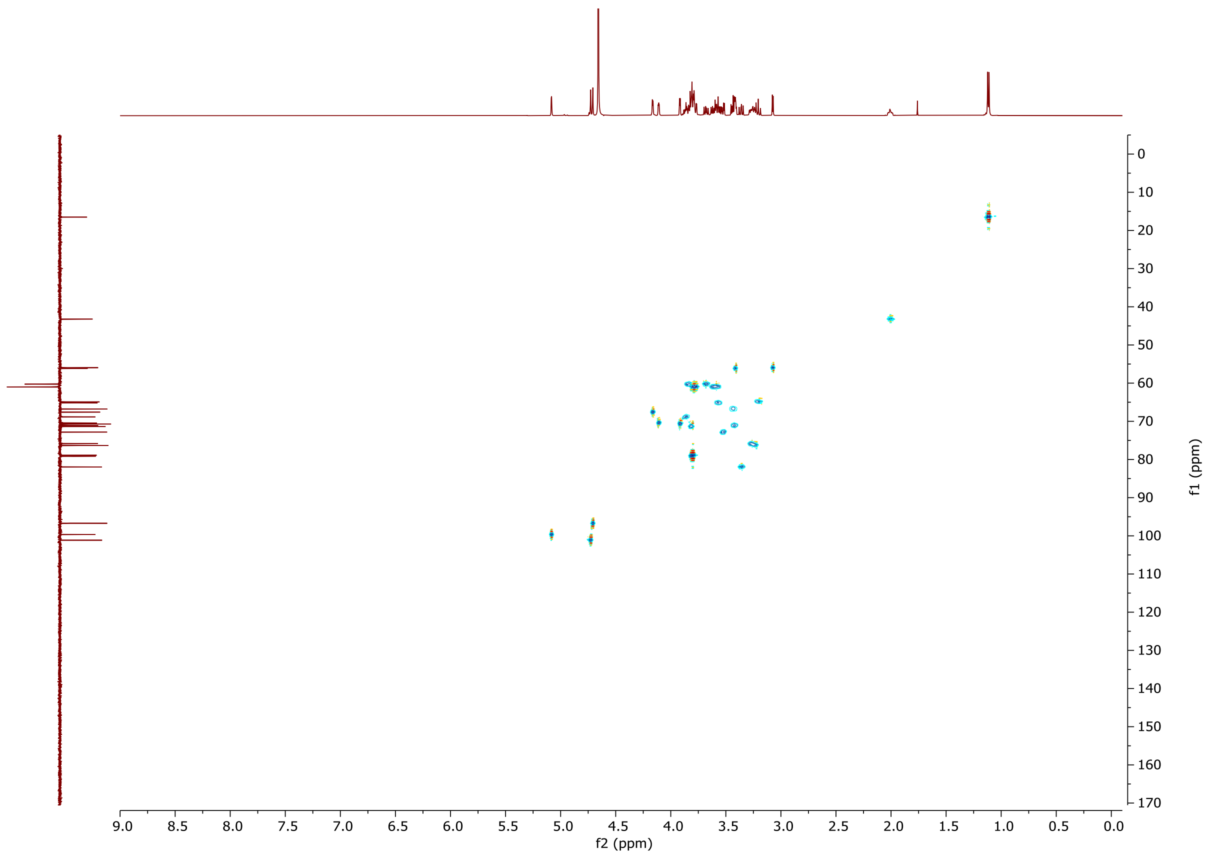

f2 (ppm)
f1 (ppm)

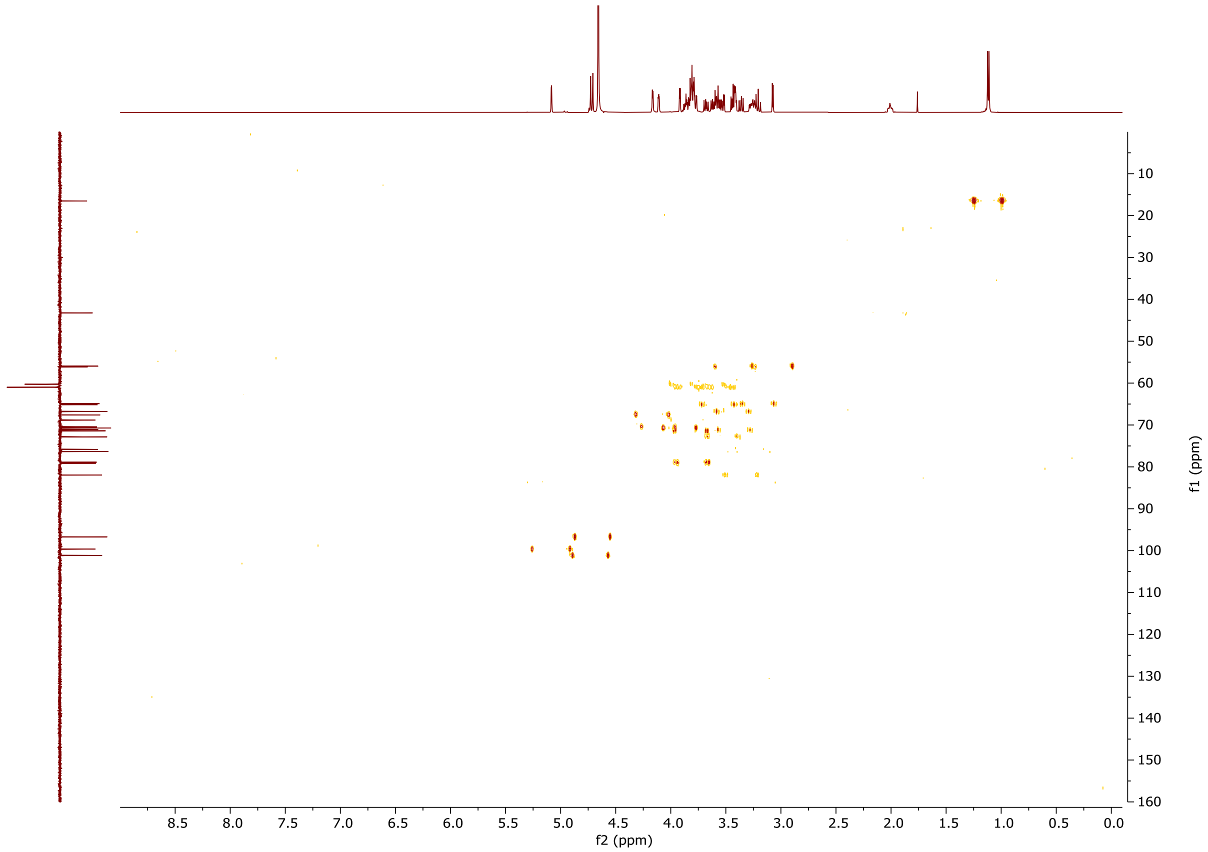

f2 (ppm)
f1 (ppm)

2,3,4,6-*O*-benzoyl-α-D-mannose-(1->2)-4,6-*O*-benzylidene-3-*O*-naphthyl-β-D-mannose-(1->3)-2-*O*-benzyl-4,6-*O*-benzylidene-β-D-mannose-(1->3)-2-*O*-benzoyl-4-*O*-benzyl-α-L-rhamnose (101)

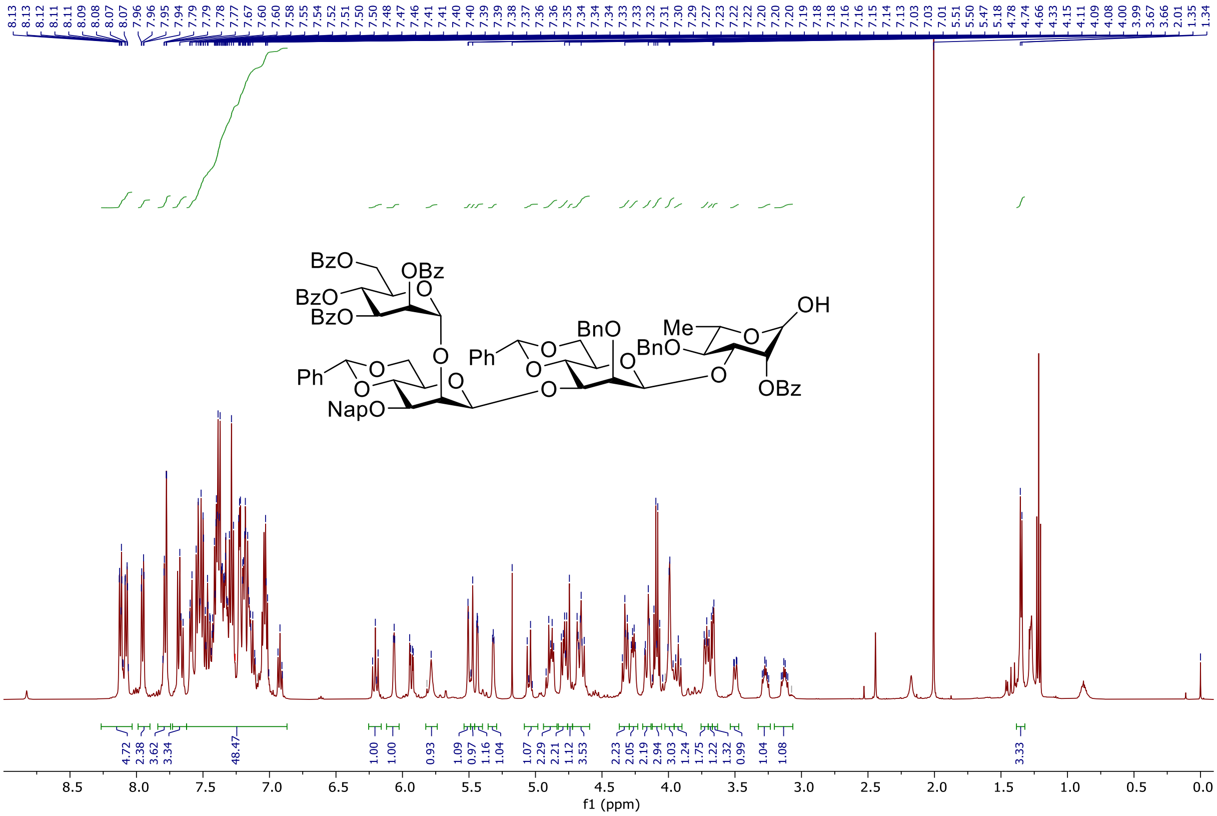

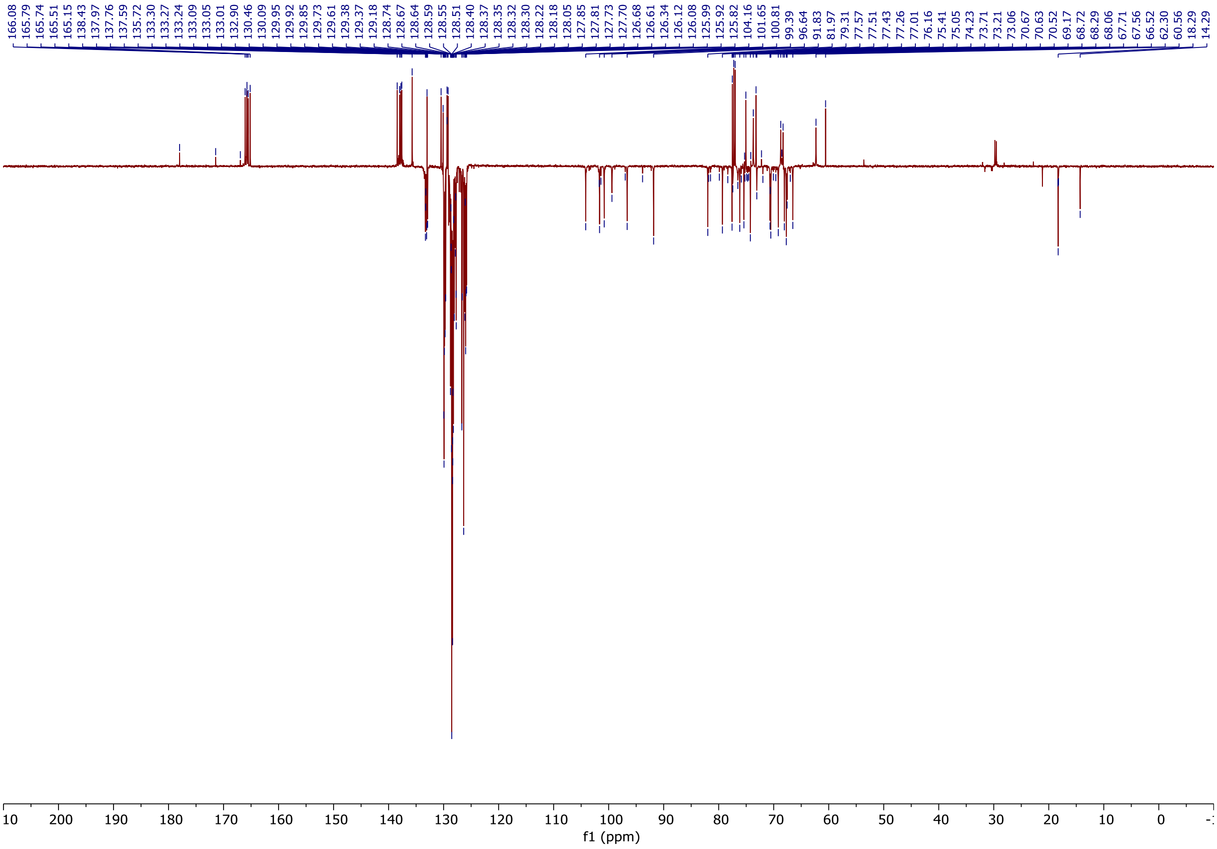

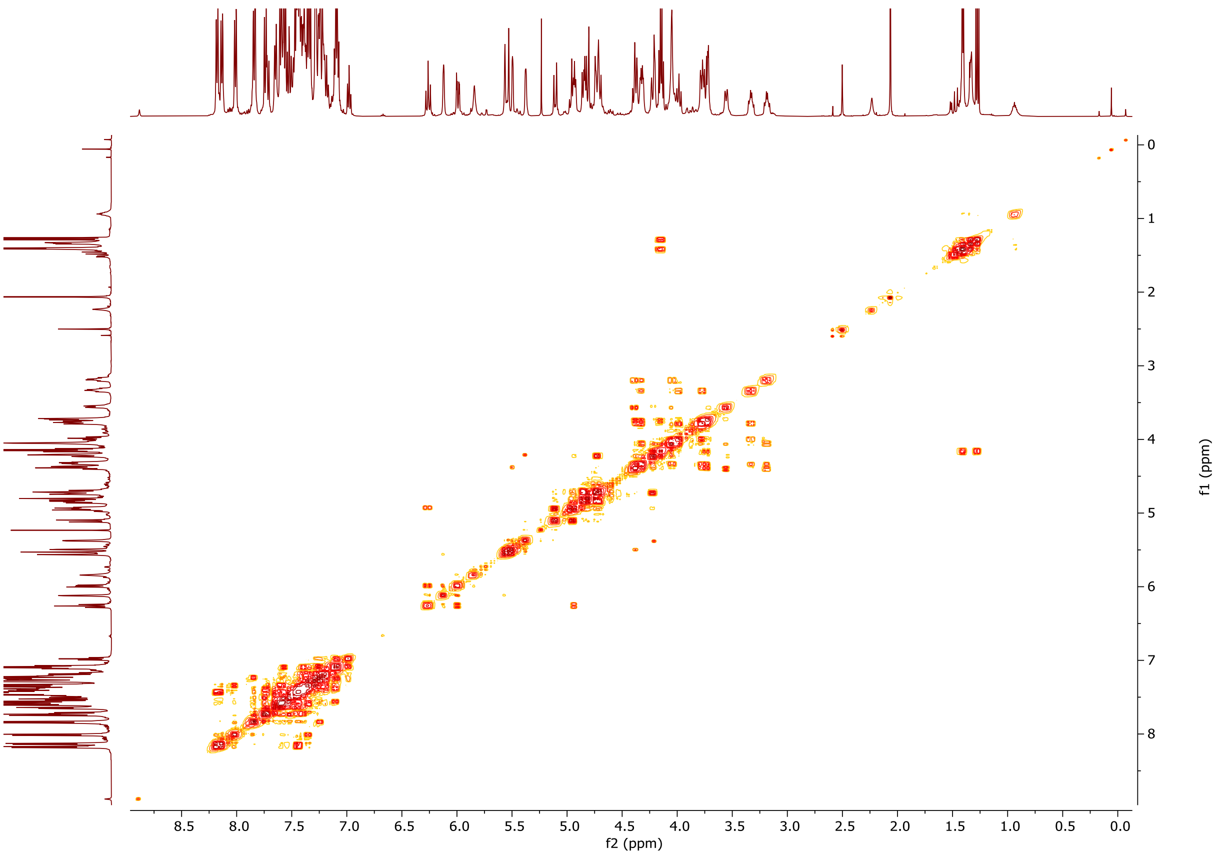
8.5 8.0 7.5 7.0 6.5 6.0 5.5 5.0 4.5 4.0 3.5 3.0 2.5 2.0 1.5 1.0 0.5 0.0
f2 (ppm)
0 1 2 3 4 5 6 7 8
f1 (ppm)

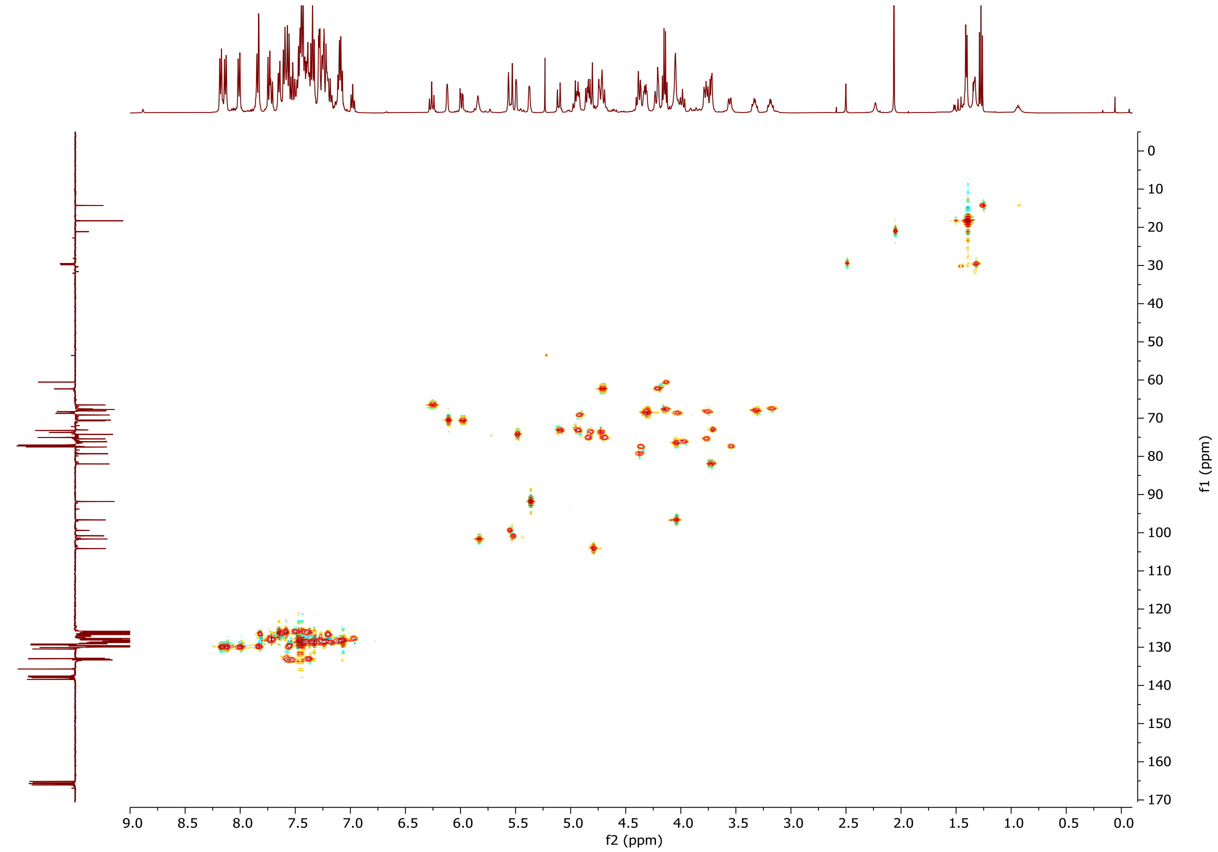
9.0 8.5 8.0 7.5 7.0 6.5 6.0 5.5 5.0 4.5 4.0 3.5 3.0 2.5 2.0 1.5 1.0 0.5 0.0
f2 (ppm)
0 10 20 30 40 50 60 70 80 90 100 110 120 130 140 150 160 170
f1 (ppm)

2,3,4,6-*O*-benzoyl-α-D-mannose-(1->2) 4,6-*O*-benzylidene-3-*O*-naphthyl-β-D-mannose-(1->3)-2-*O*-benzyl-4,6-*O*-benzylidene-β-D-mannose-(1->3)-1-*O*-trichloroimidate-2-*O*-benzoyl-4-*O*-benzyl-α-L-rhamnose (102)

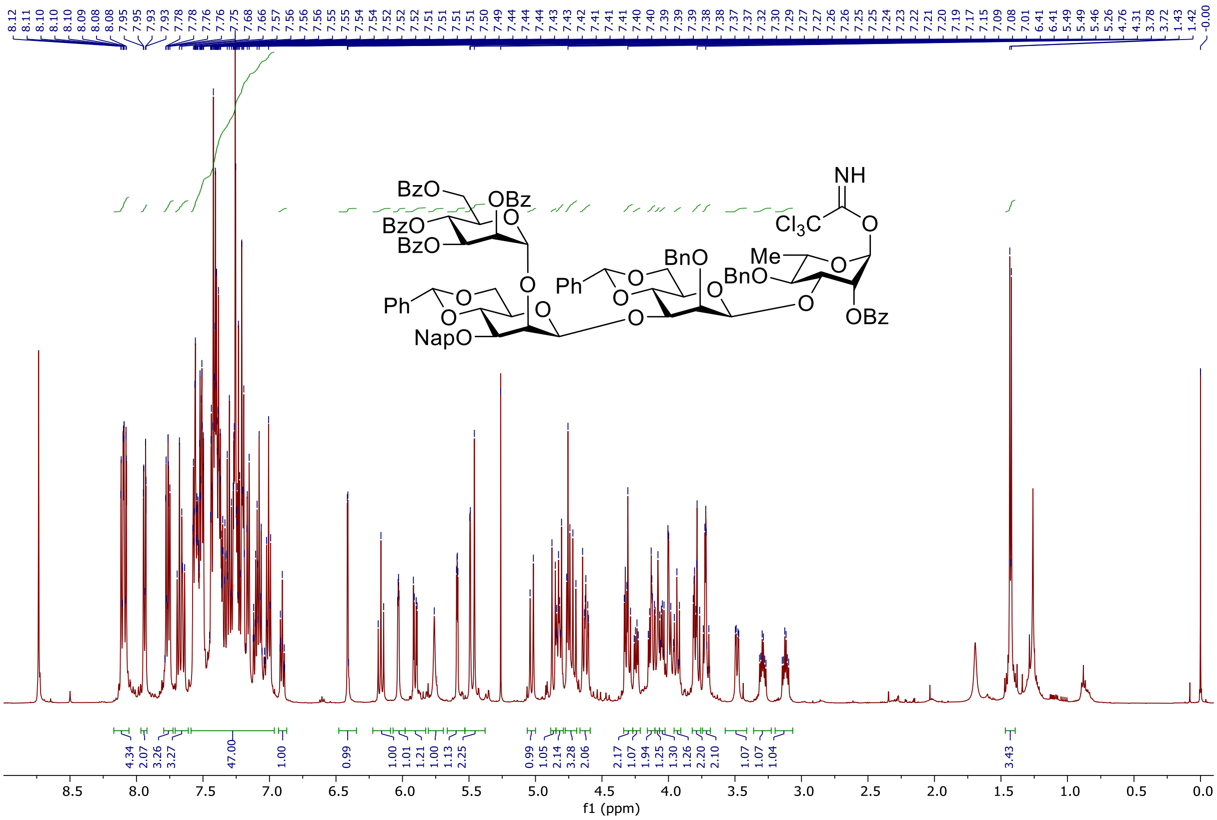

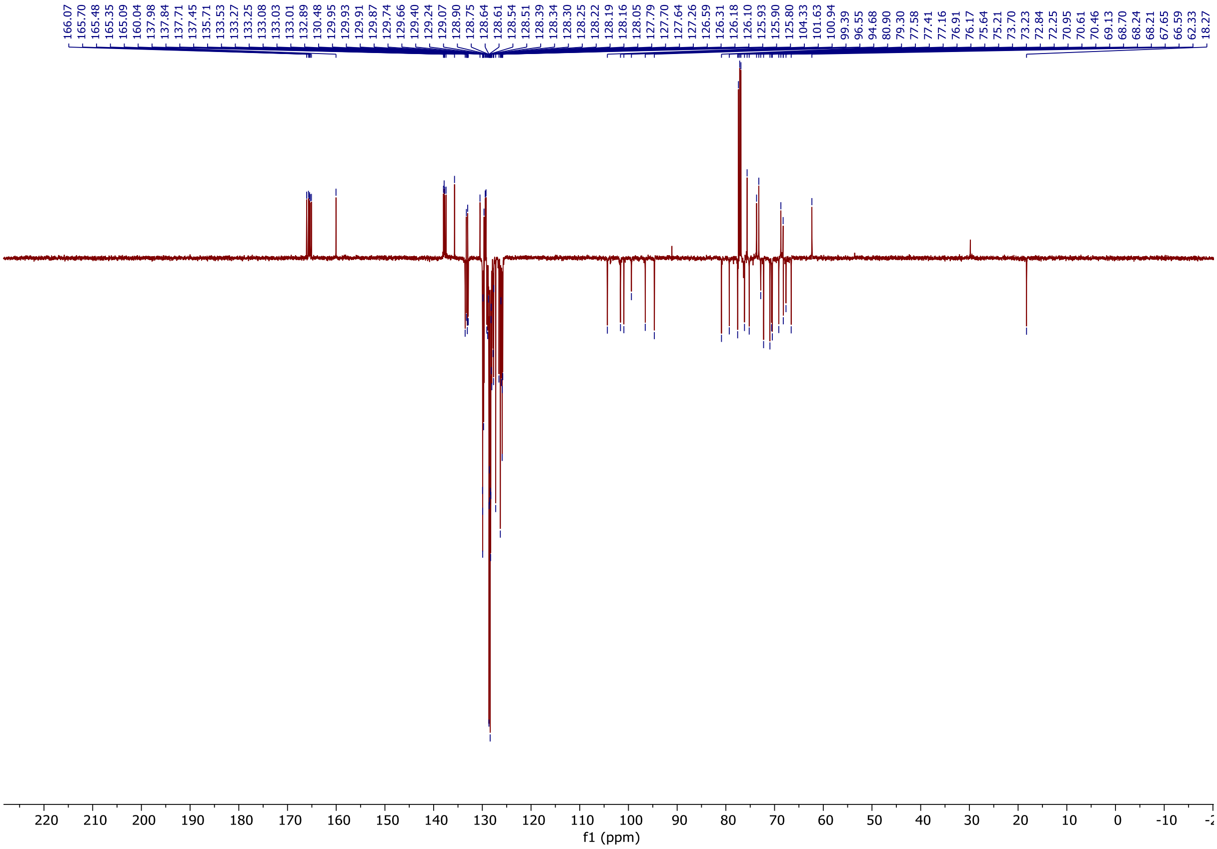

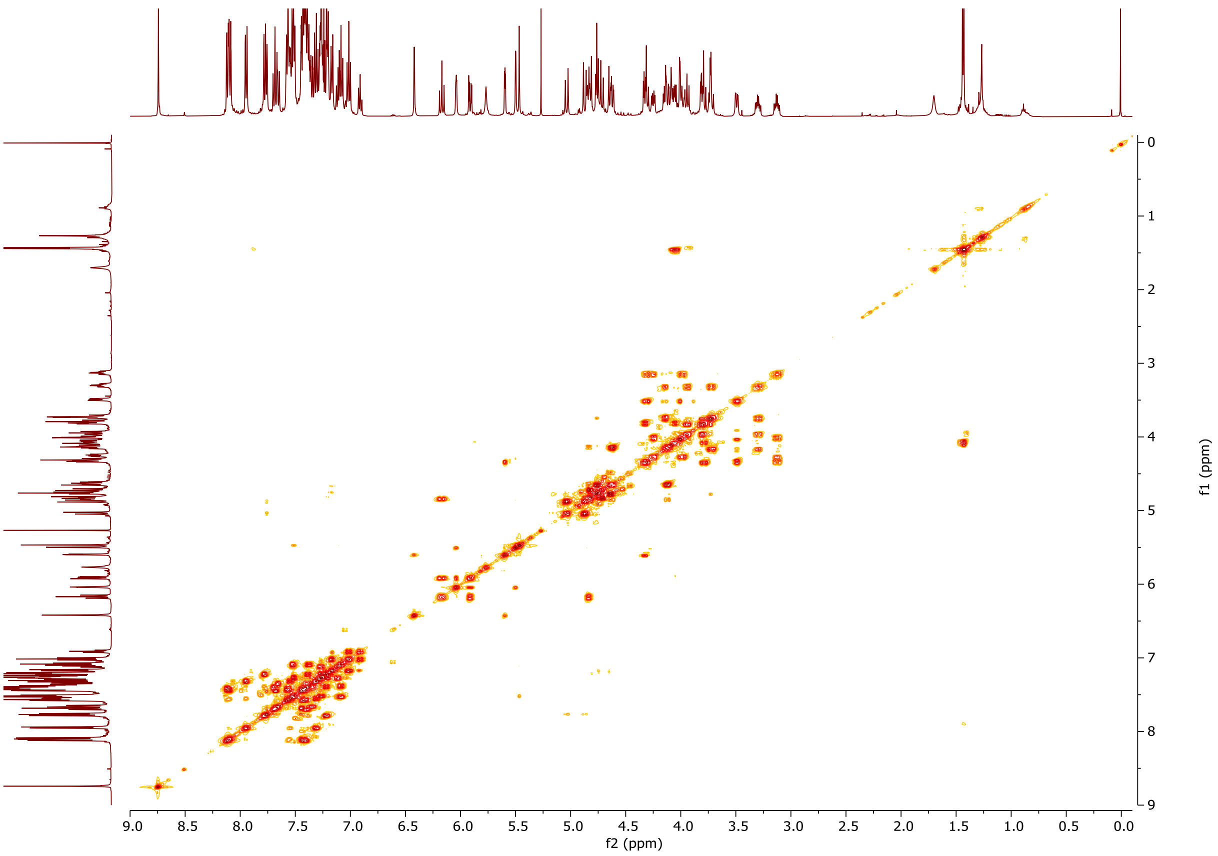
f2 (ppm)
f1 (ppm)

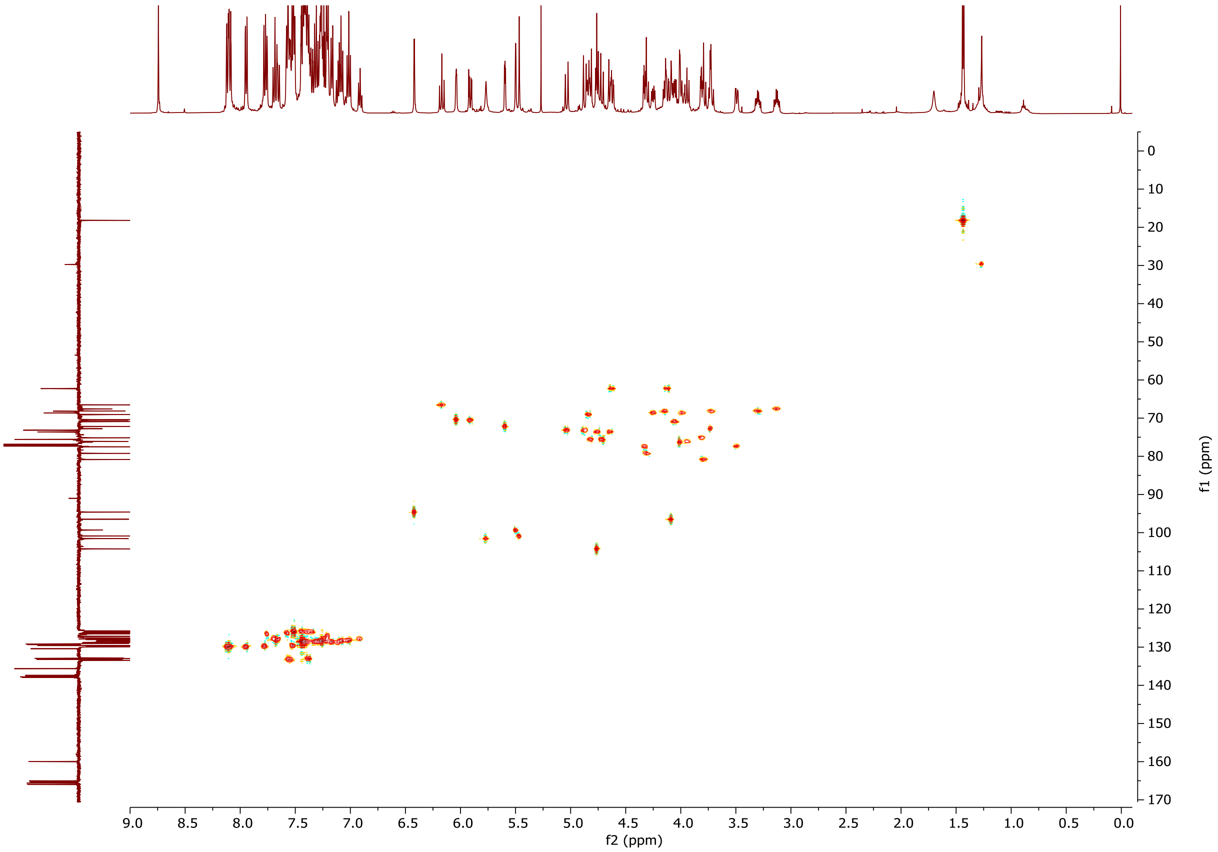
f2 (ppm)
f1 (ppm)

2,3,4,6-*O*-benzoyl-α-D-mannose-(1->2) 4,6-*O*-benzylidene-3-*O*-naphthyl-β-D-mannose-(1->3)-2-*O*-benzyl-4,6-*O*-benzylidene-β-D-mannose-(1->3)-2-*O*-benzoyl-4-*O*-benzyl-α-L-rhamnose-(1->3)-2-*O*-benzyl-6-*O*-t-butyldiphenylsilyl-3-*O*-t-butyldimethylsilyl-β-D-glucose-cyclophellitol (103)

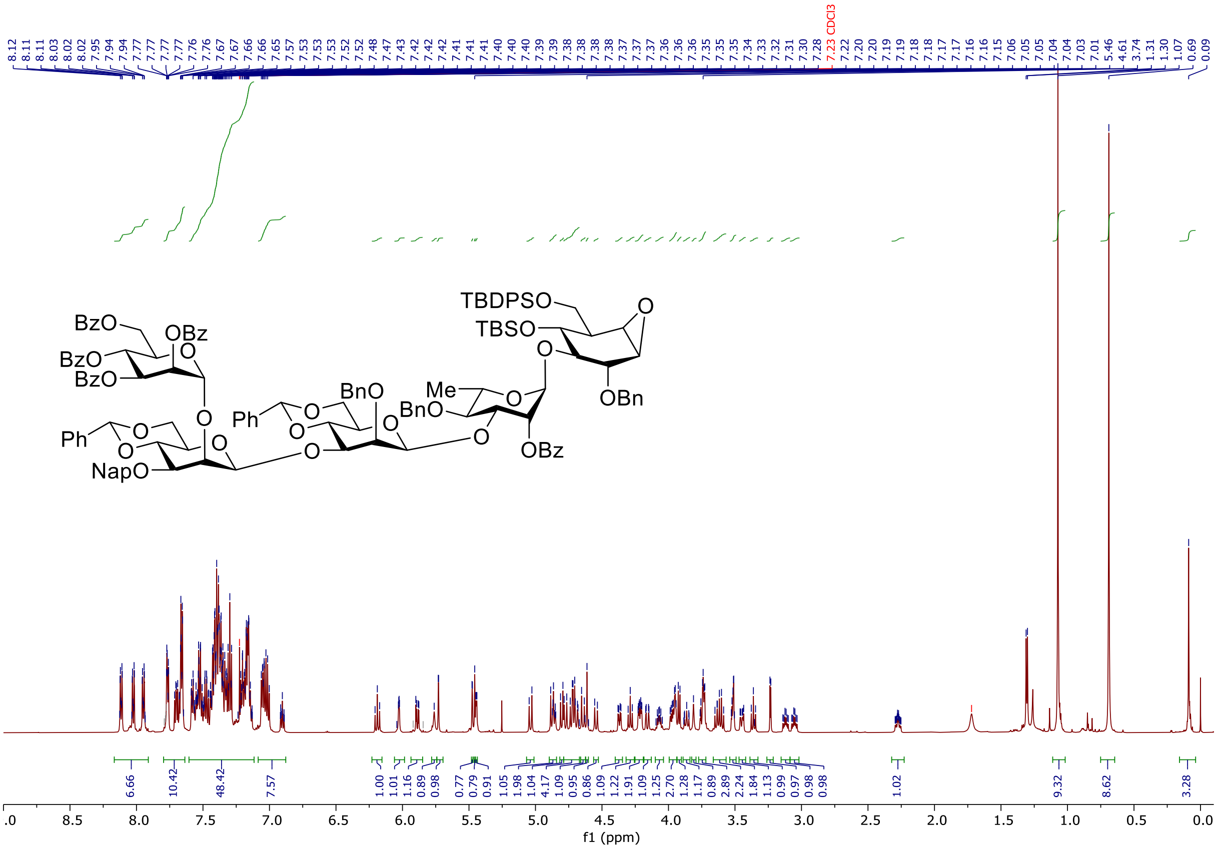

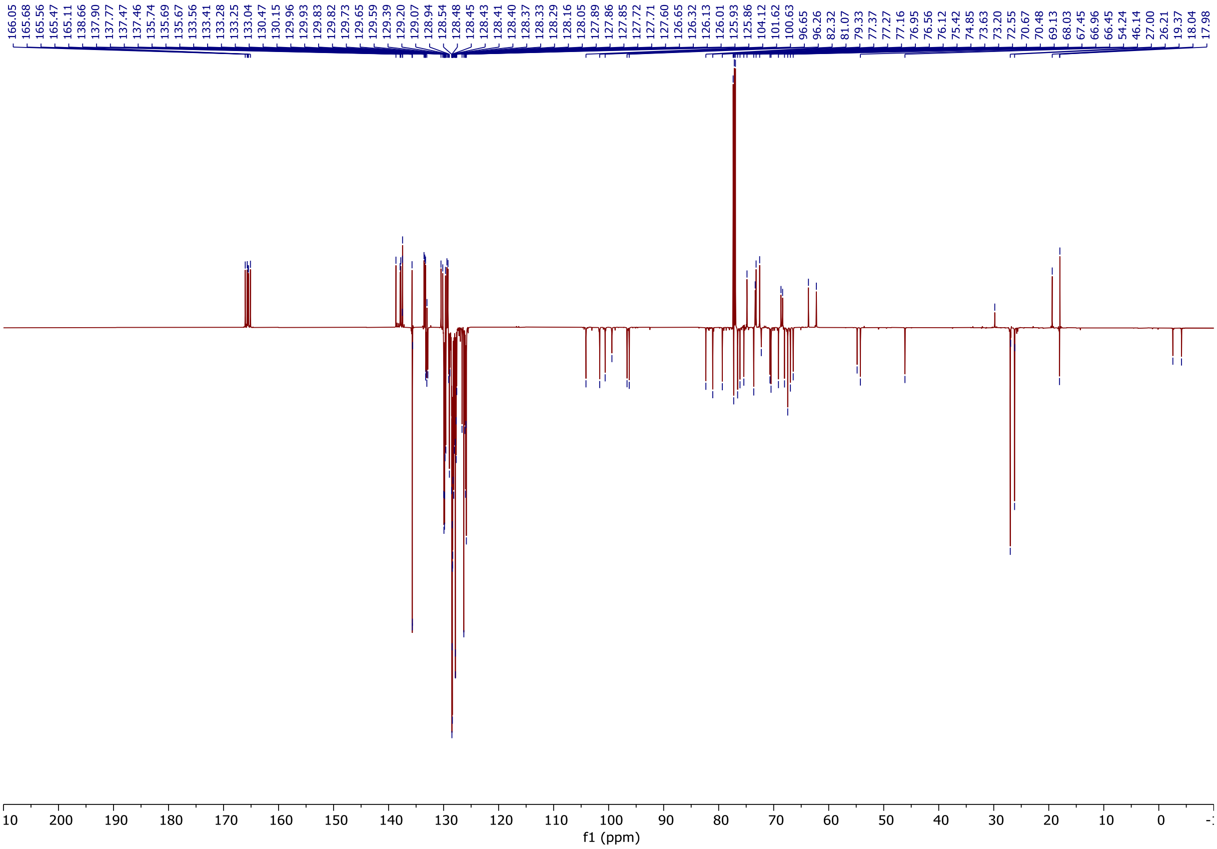

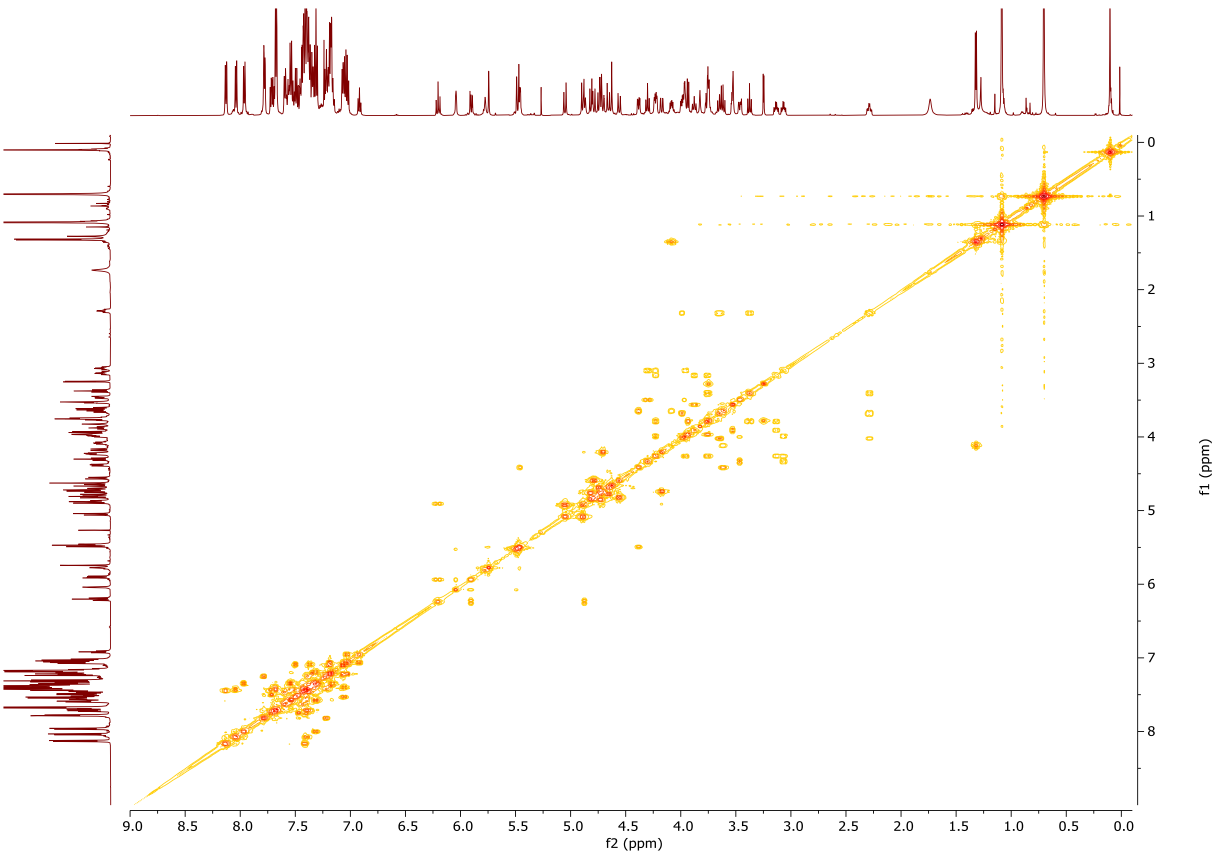

9.0
8.5
8.0
7.5
7.0
6.5
6.0
5.5
5.0
4.5
4.0
3.5
3.0
2.5
2.0
1.5
1.0
0.5
0.0
f2 (ppm)
f1 (ppm)

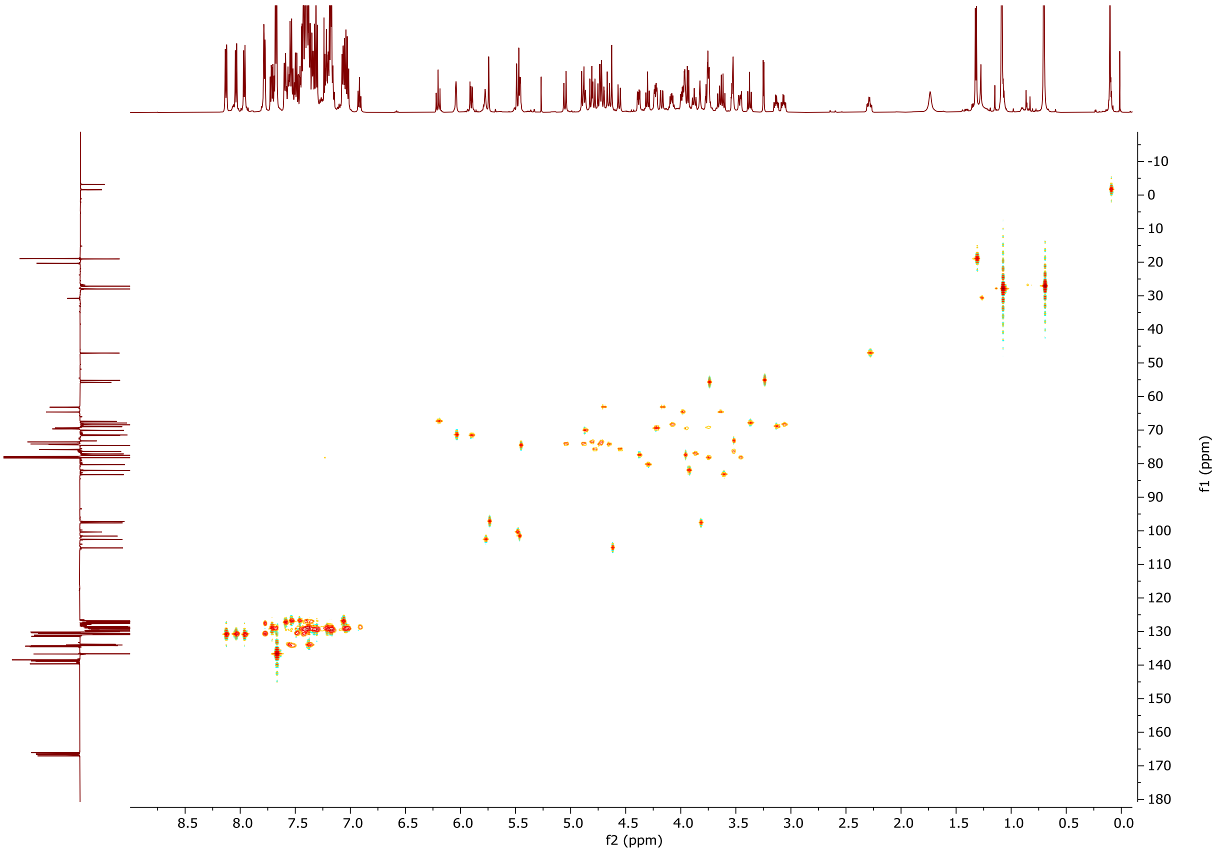

8.5
8.0
7.5
7.0
6.5
6.0
5.5
5.0
4.5
4.0
3.5
3.0
2.5
2.0
1.5
1.0
0.5
0.0
f2 (ppm)
f1 (ppm)

α-D-mannose-(1->2)-β-D-mannose-(1->3)-β-D-mannose-(1->3)-α-L-rhamnose-(1->3)-β-D-glucose-cyclophellitol (20)

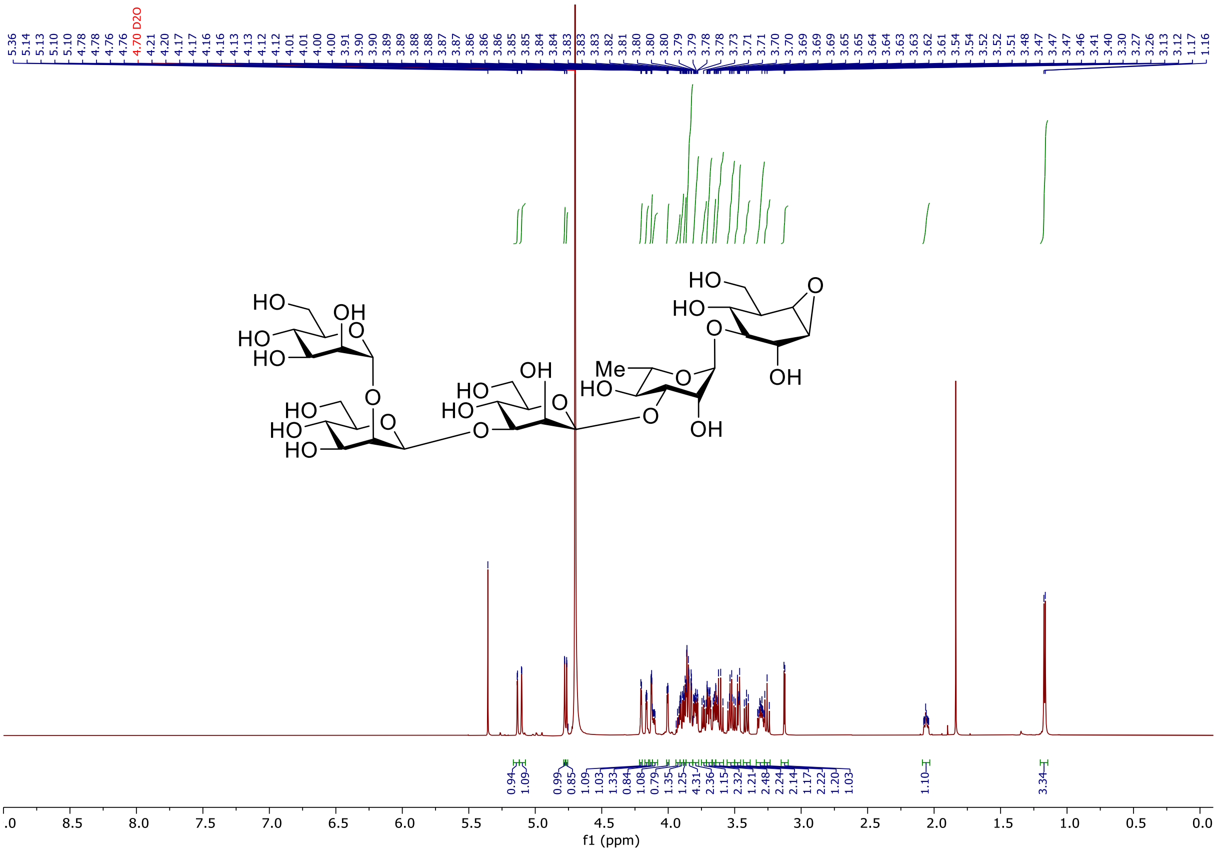

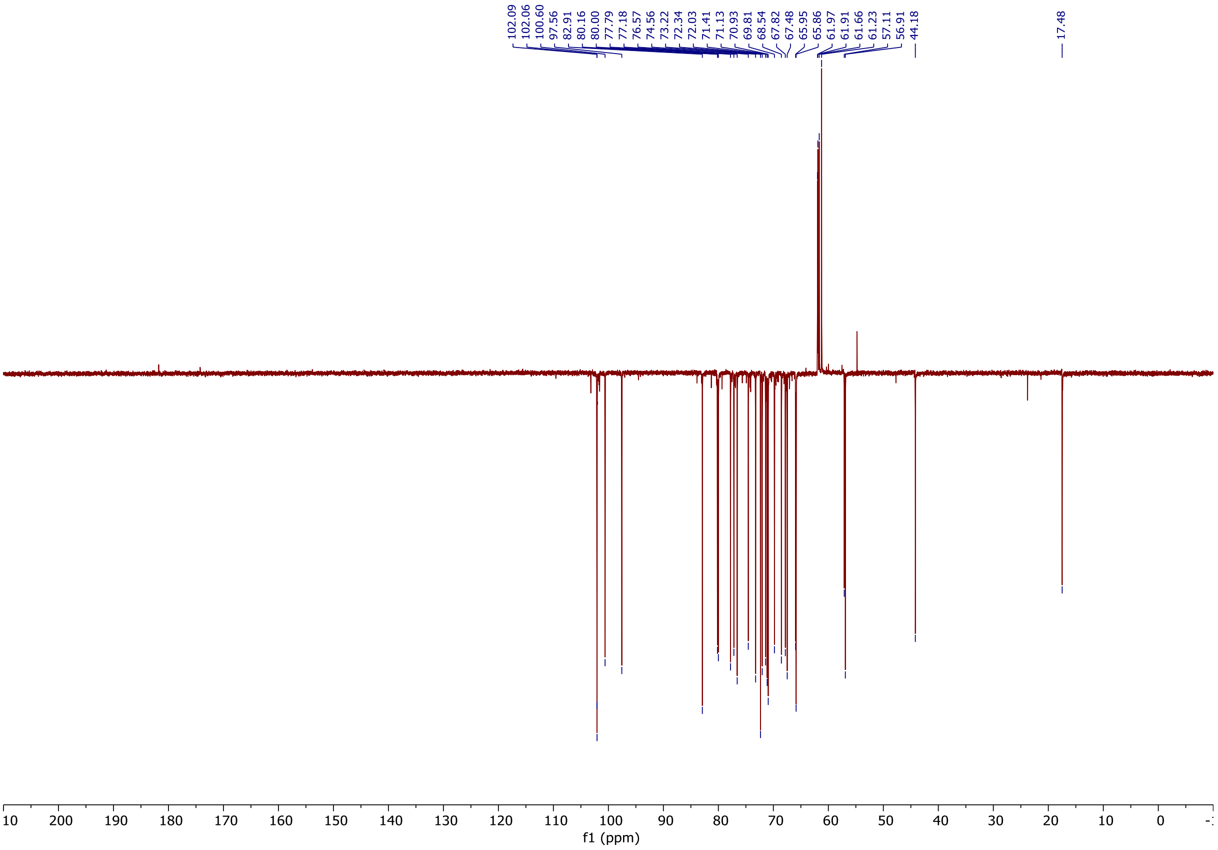

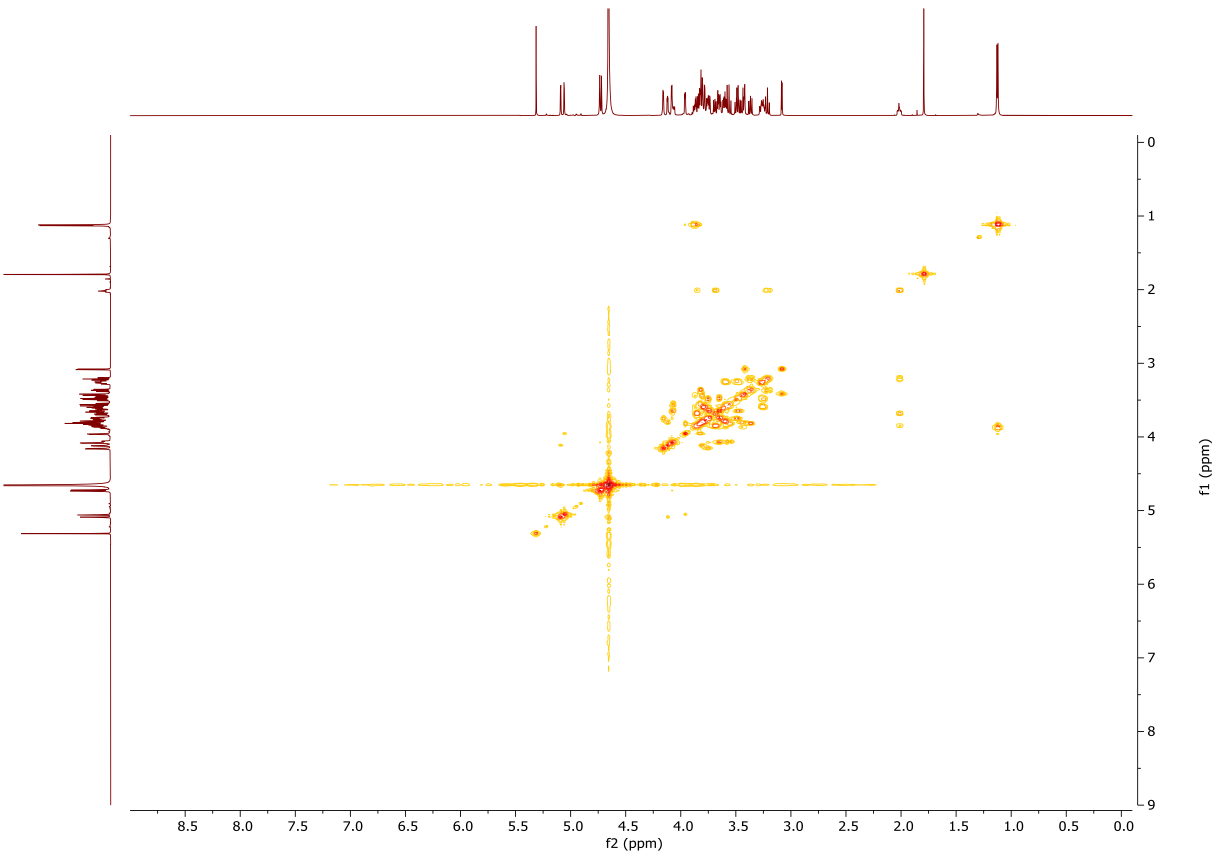
f1 (ppm)
f2 (ppm)

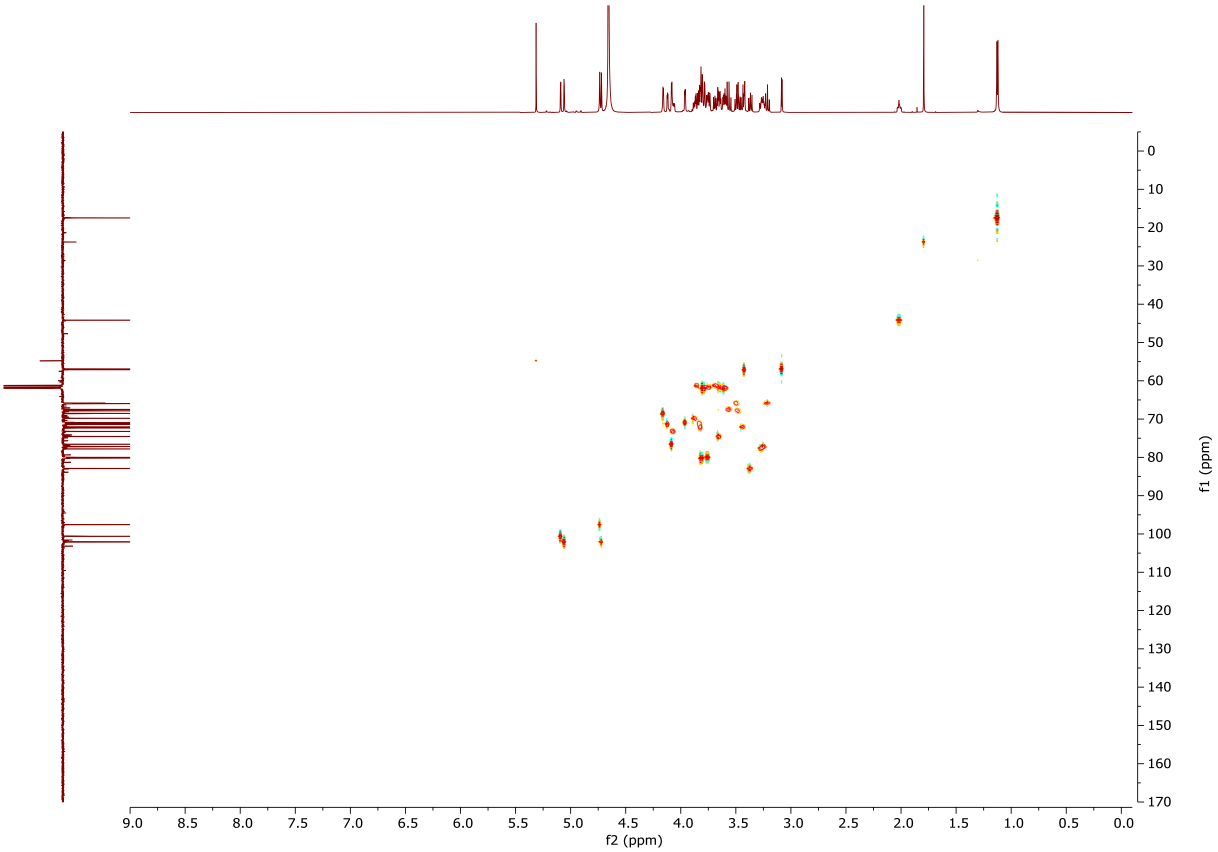
f1 (ppm)
f2 (ppm)

1-*O*-allyl-2,4,6-tri-*O*-benzyl-3-*O*-naphthyl-β-D-glucose (105)

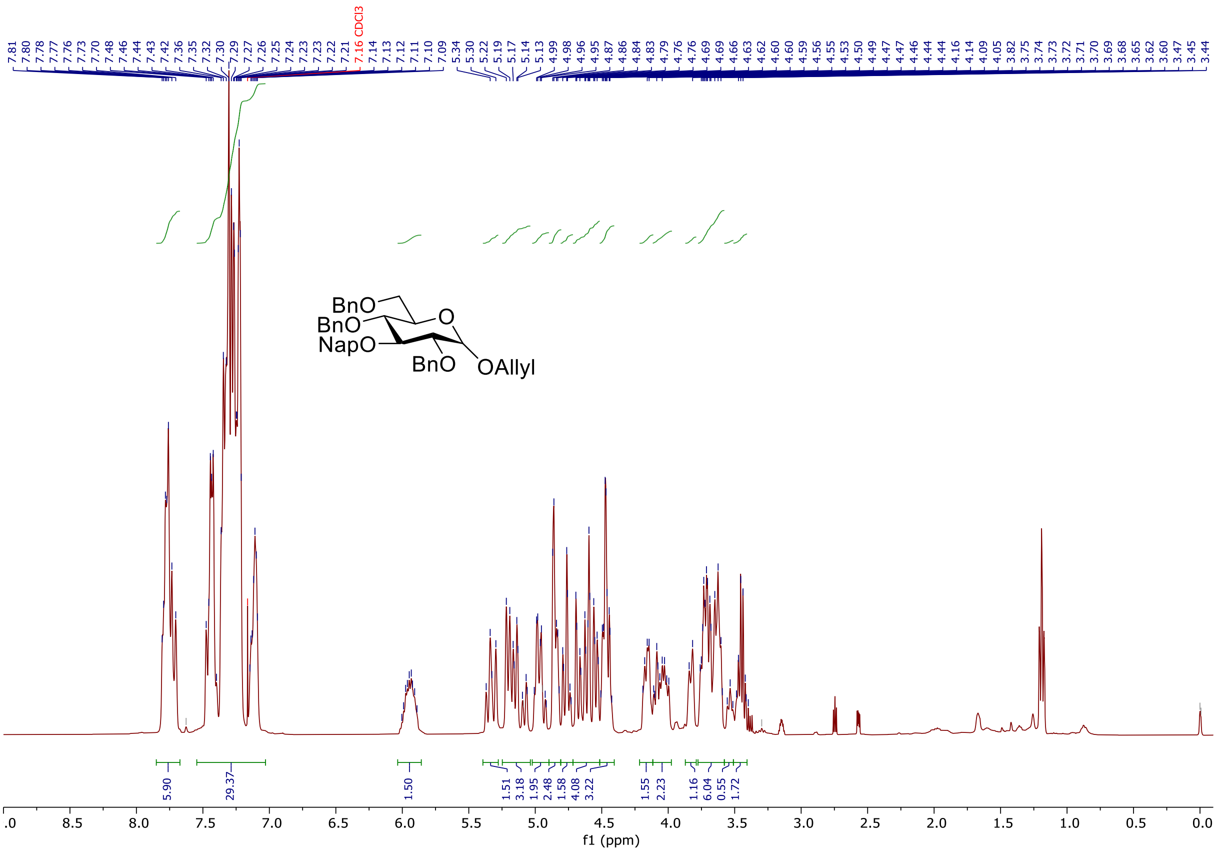

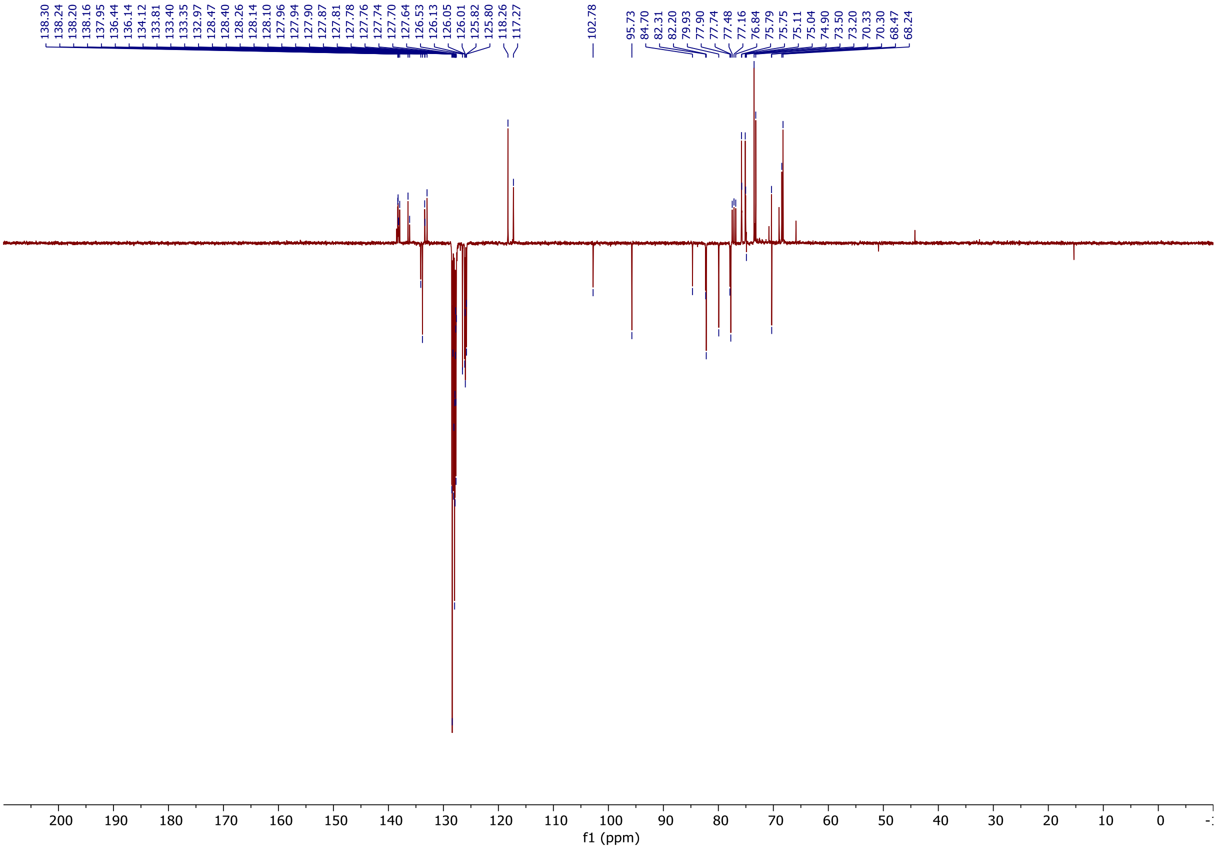

1-*O*-allyl-2,4,6-tri-*O*-benzyl-β-ᴅ-glucose (106)

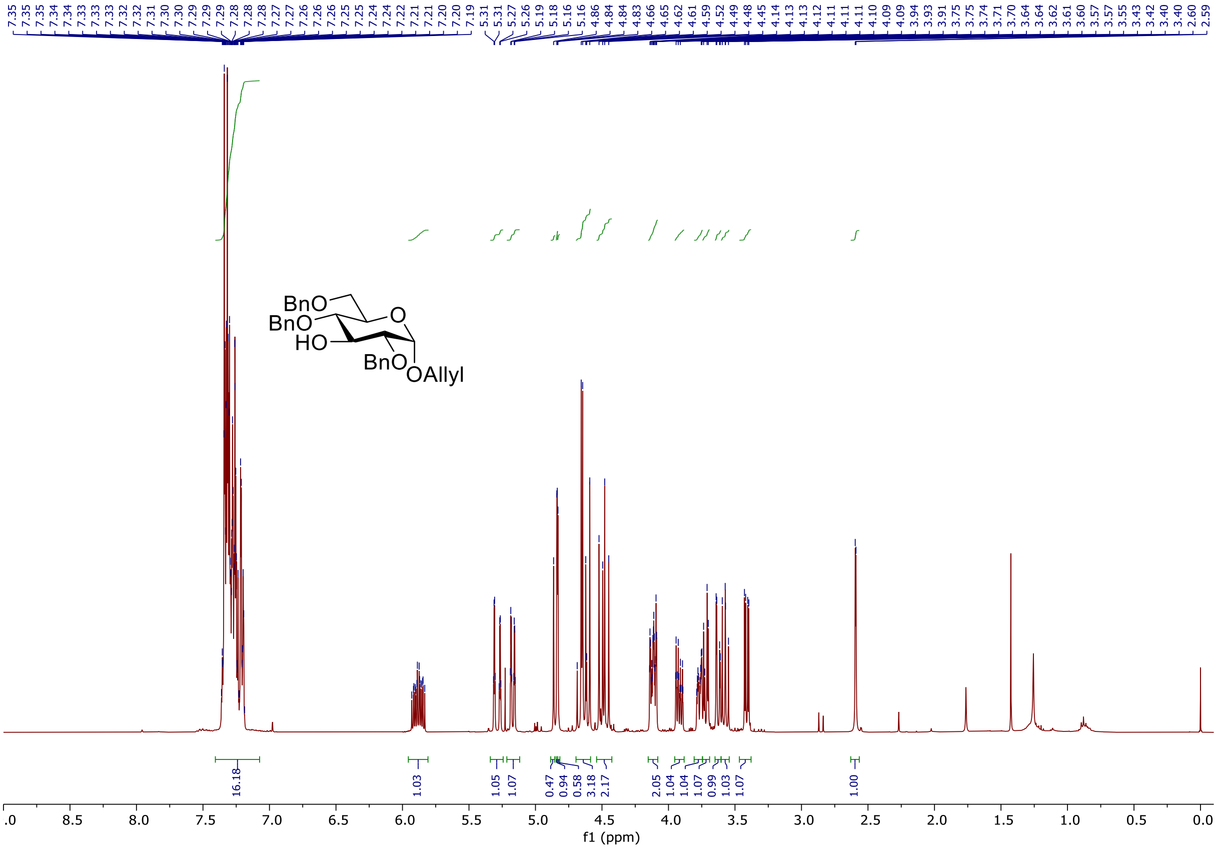

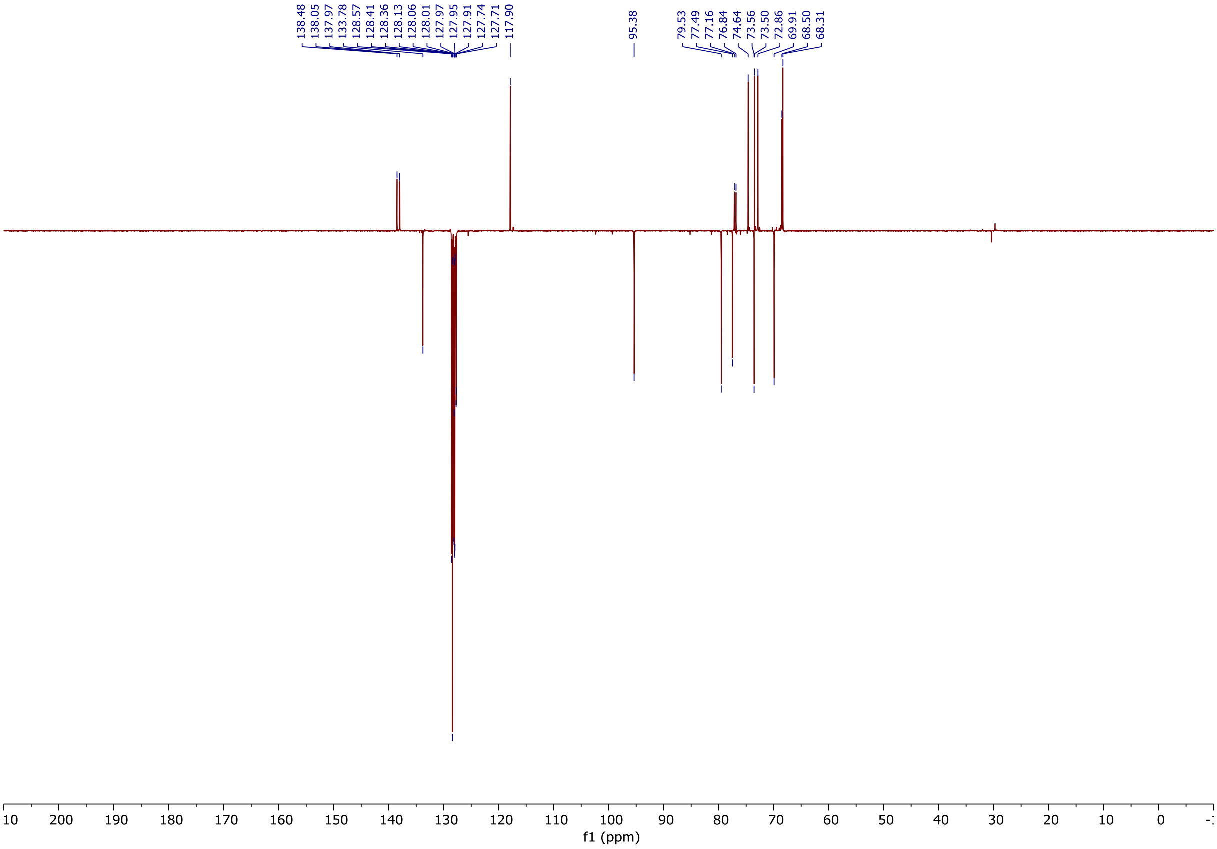

2-*O*-benzyl-4,6-*O*-benzylidene-3-*O*-naphthyl-β-D-mannose-(1->3)-2-*O*-benzoyl-4-*O*-benzyl-α-L-rhamnose-(1->3)-1-*O*-allyl-2,4,6-tri-*O*-benzyl-β-D-glucose (107)

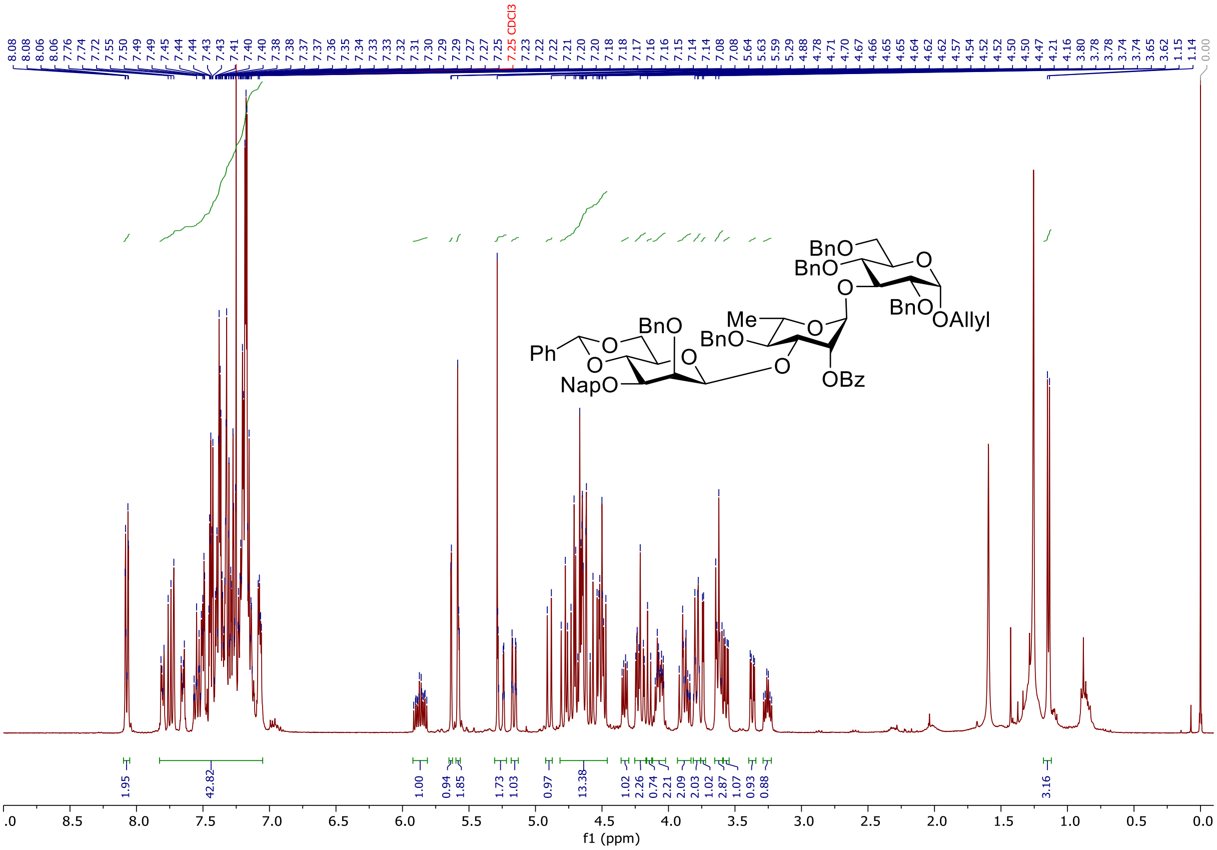

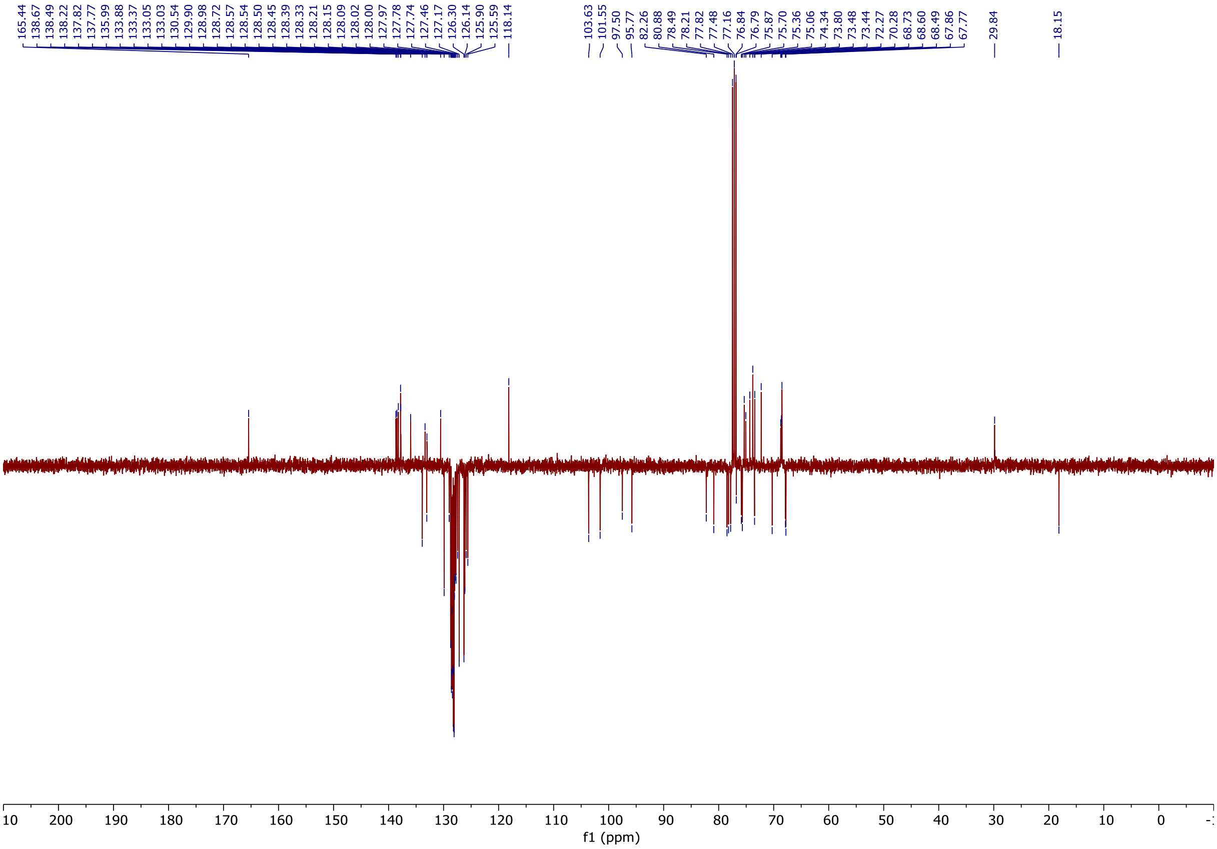

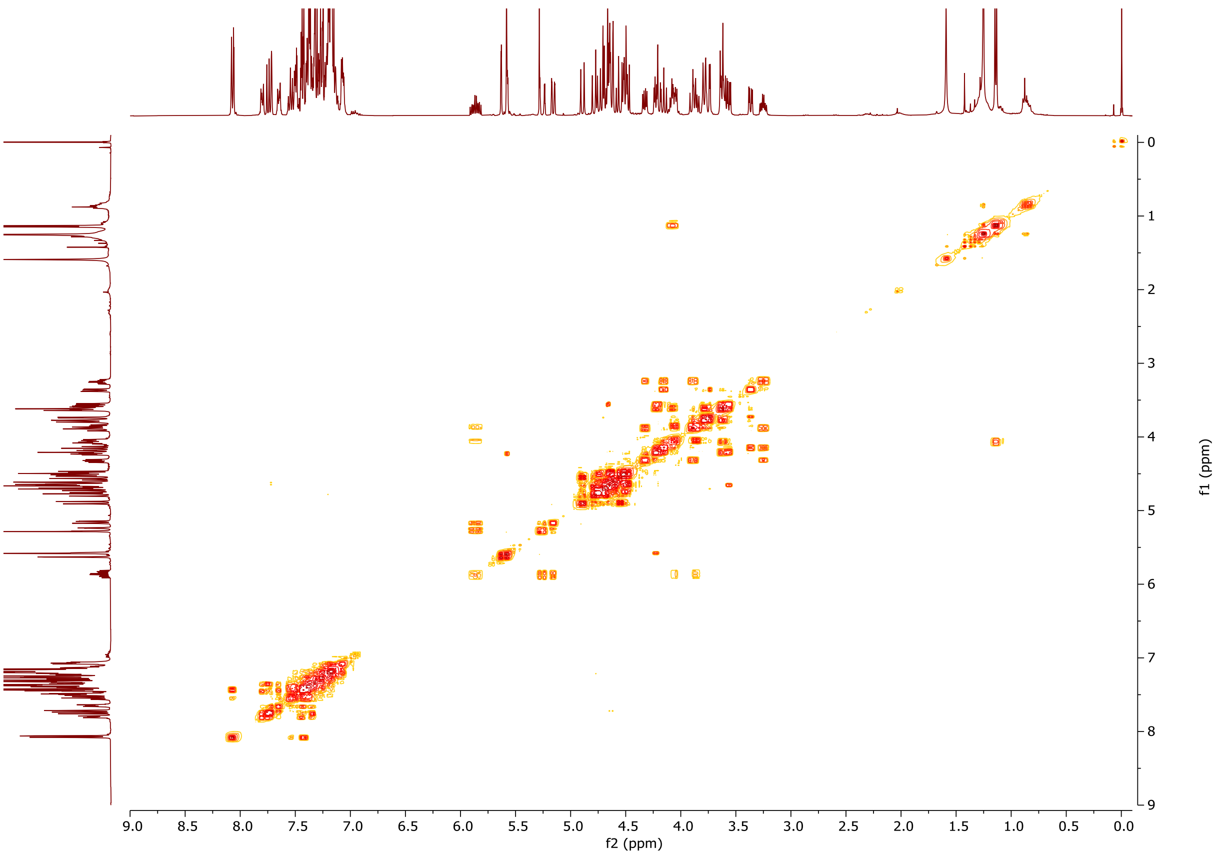
f2 (ppm)
f1 (ppm)

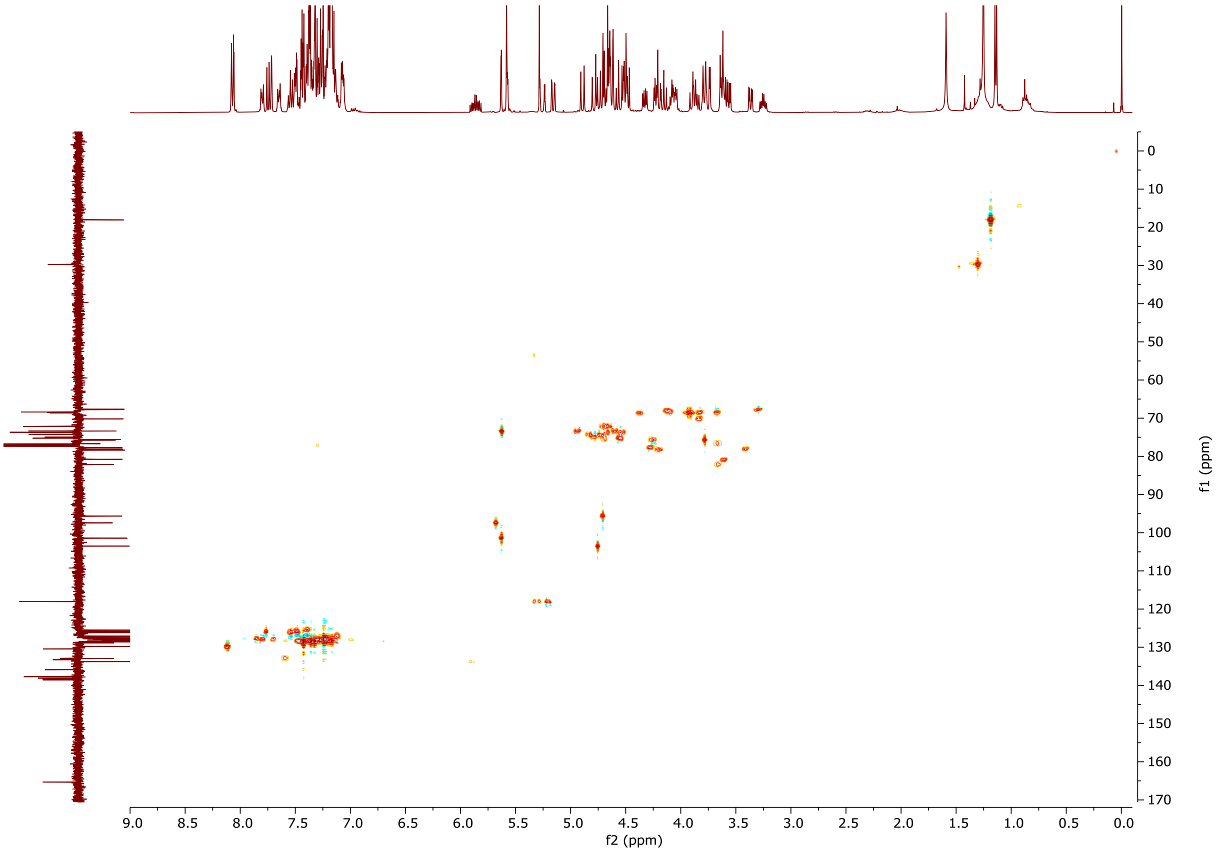
f2 (ppm)
f1 (ppm)

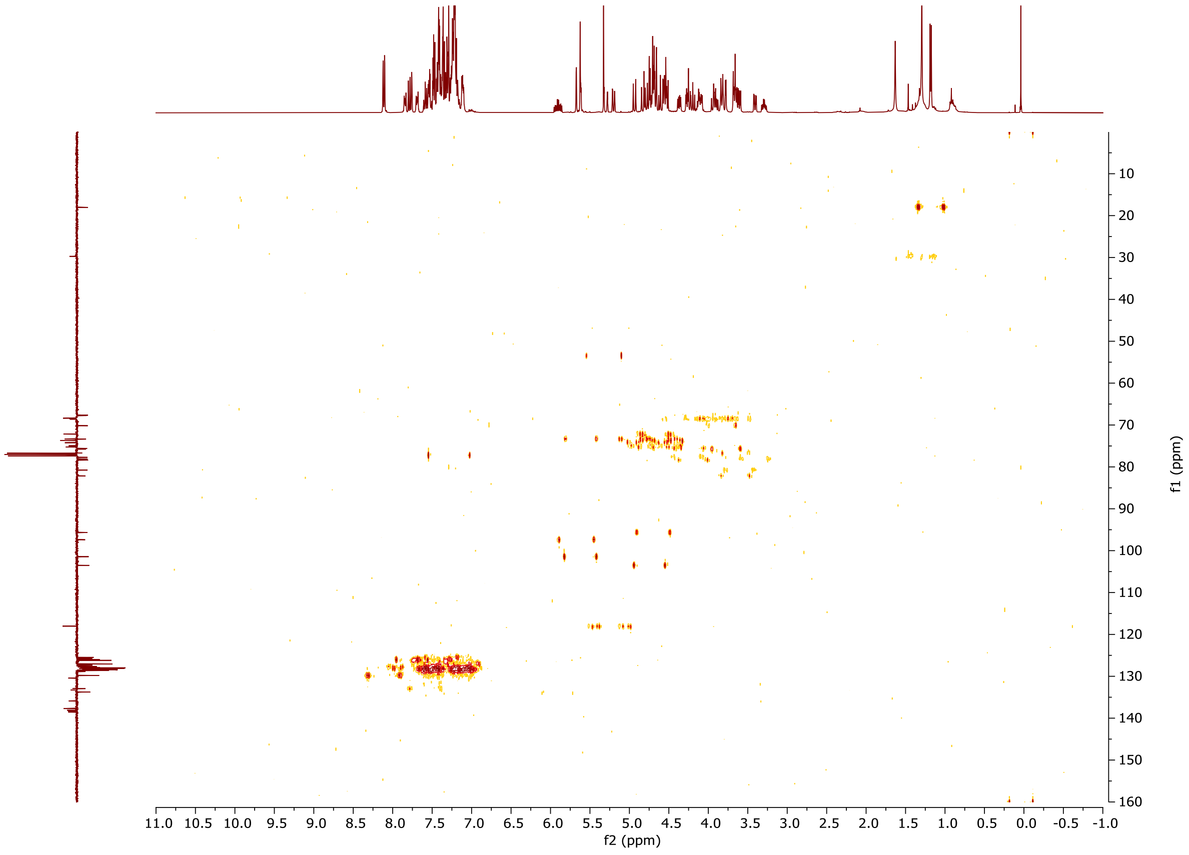

2-*O*-benzyl-4,6-*O*-benzylidene-β-ᴅ-mannose-(1->3)-2-*O*-benzoyl-4-*O*-benzyl-α-ʟ-rhamnose-(1->3)-1-*O*-allyl-2,4,6-tri-*O*-benzyl-β-ᴅ-glucose (108)

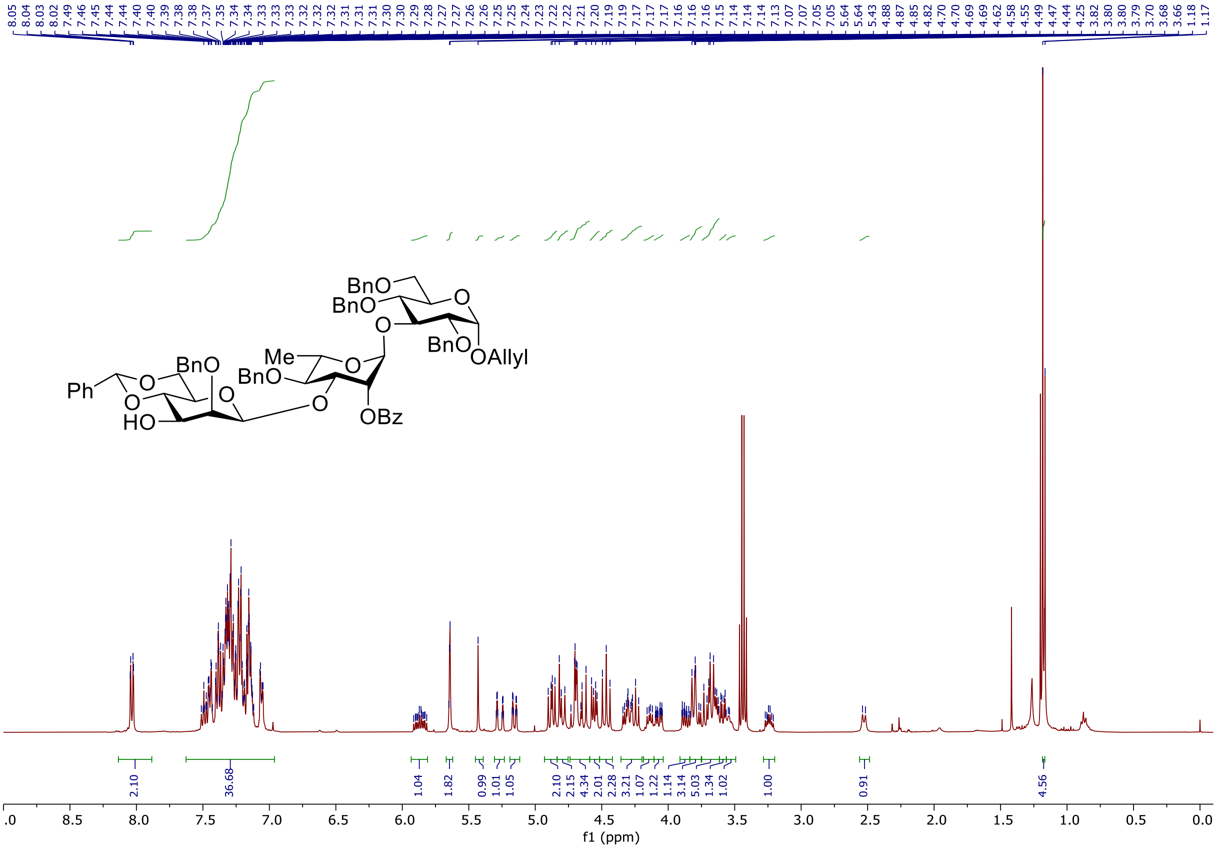

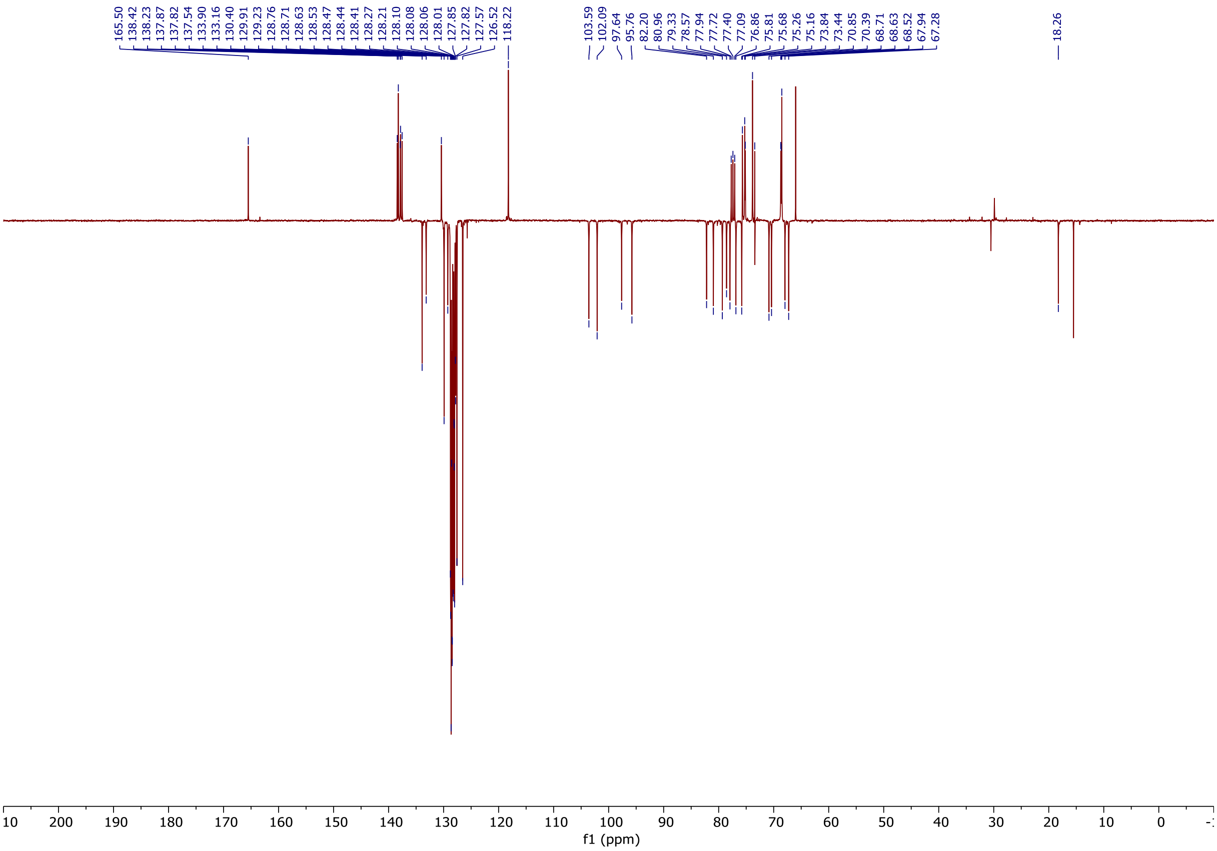
165.50
138.42
138.23
137.87
137.82
137.54
133.90
133.16
130.40
129.91
129.23
128.76
128.71
128.63
128.53
128.47
128.44
128.41
128.27
128.21
128.10
128.08
128.06
128.01
127.85
127.82
127.57
126.52
118.22
103.59
102.09
97.64
95.76
82.20
80.96
79.33
78.57
77.94
77.72
77.40
77.09
76.86
75.81
75.68
75.26
75.16
73.84
73.44
70.85
70.39
68.71
68.63
68.52
67.94
67.28
18.26
f1 (ppm)

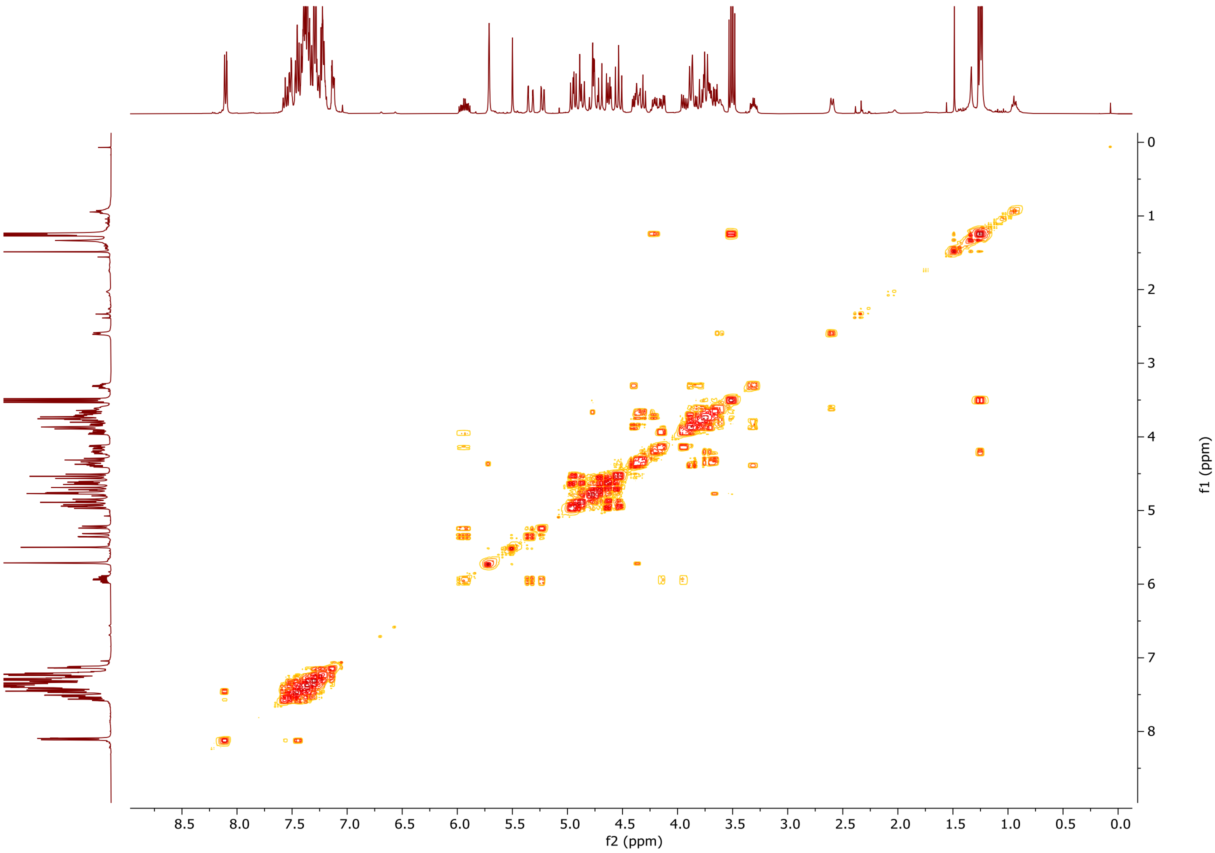
f2 (ppm)
f1 (ppm)

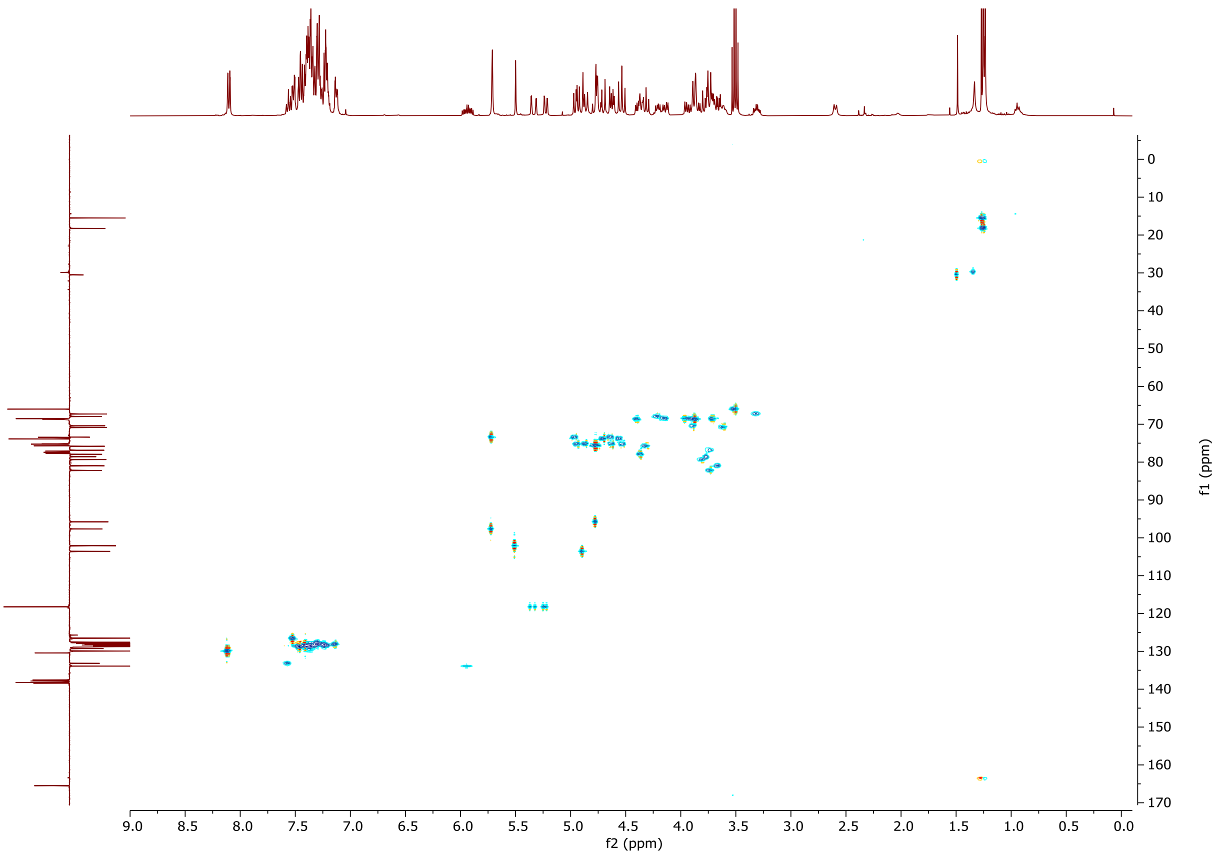

f1 (ppm)
f2 (ppm)

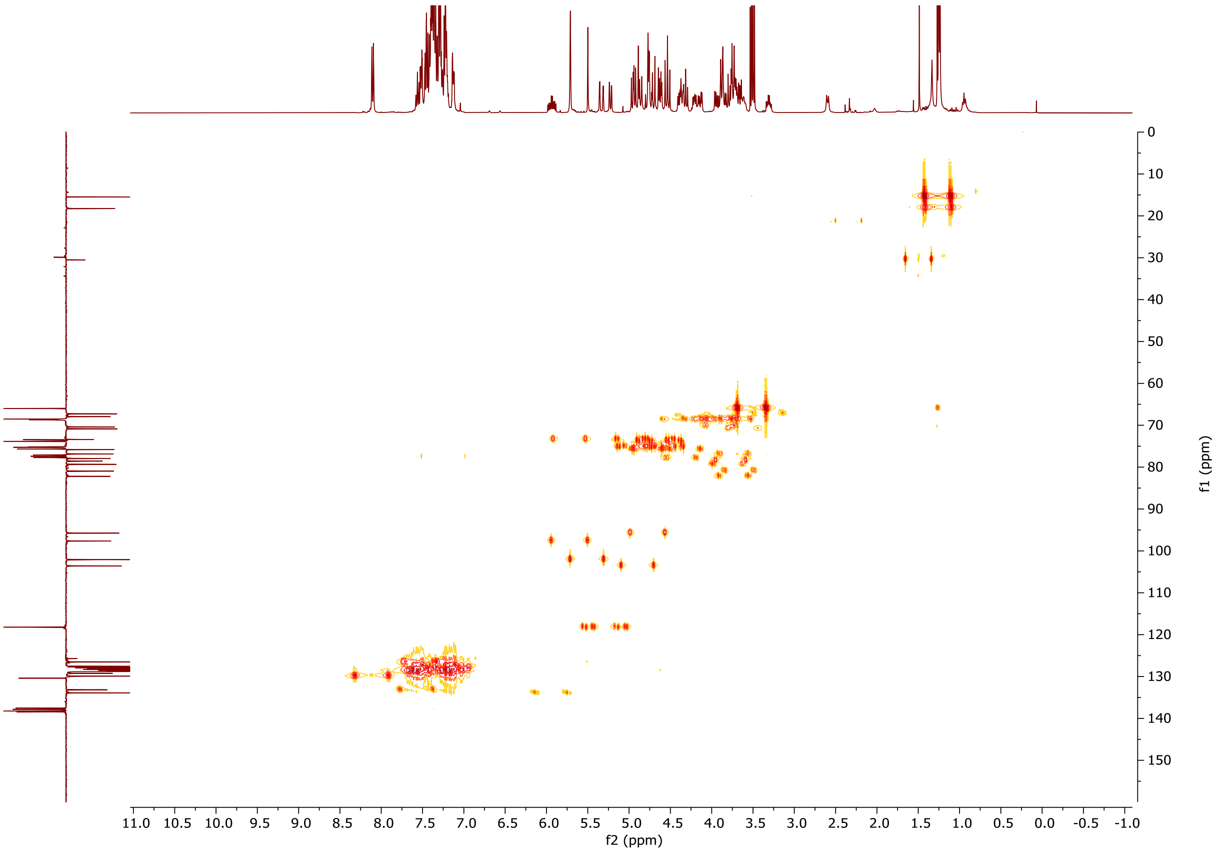

f1 (ppm)
f2 (ppm)

4,6-*O*-benzylidene-2-*O*-tert-butyldimethylsilyl-3-*O*-naphthyl-β-ᴅ-mannose-(1->3)-2-*O*-benzyl-4,6-*O*-benzylidene-β-ᴅ-mannose-(1->3)-2-*O*-benzoyl-4-*O*-benzyl-α-ʟ-rhamnose-(1->3)-1-*O*-allyl-2,4,6-tri-*O*-benzyl-β-ᴅ-glucose (109)

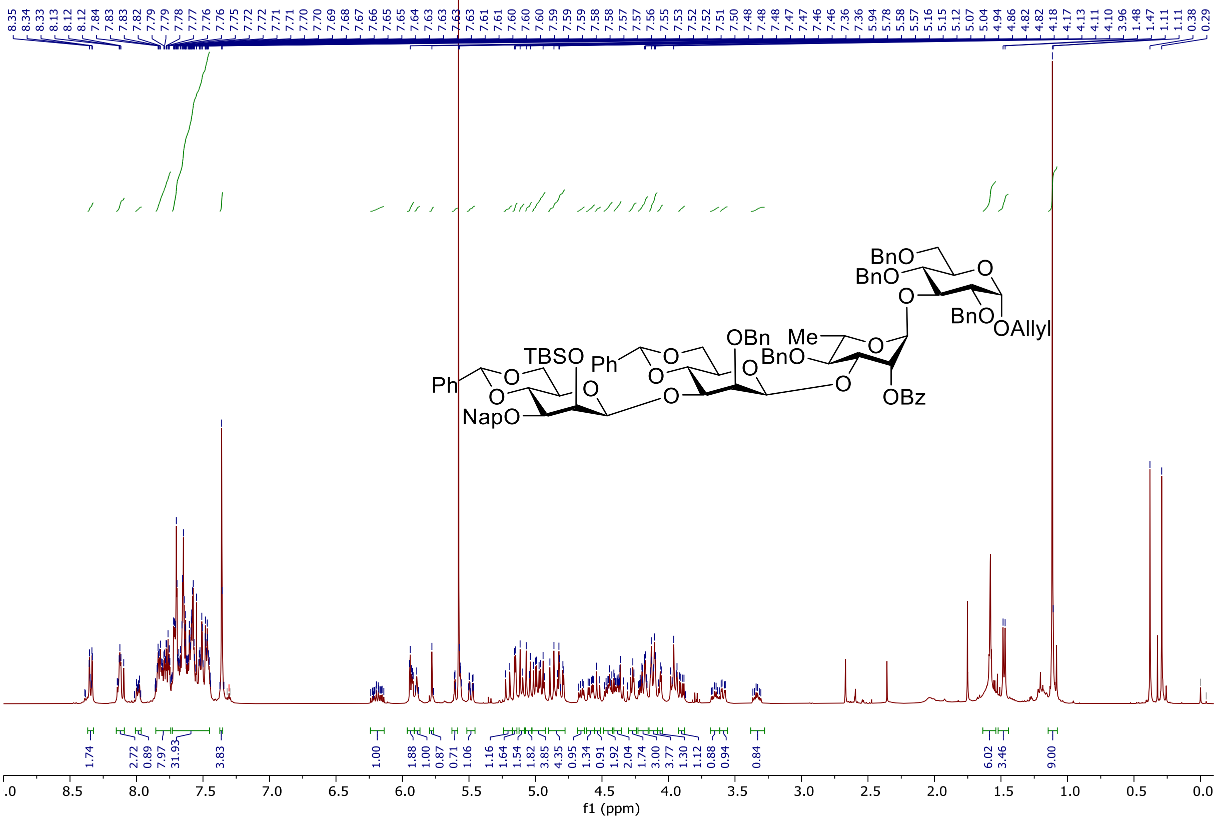

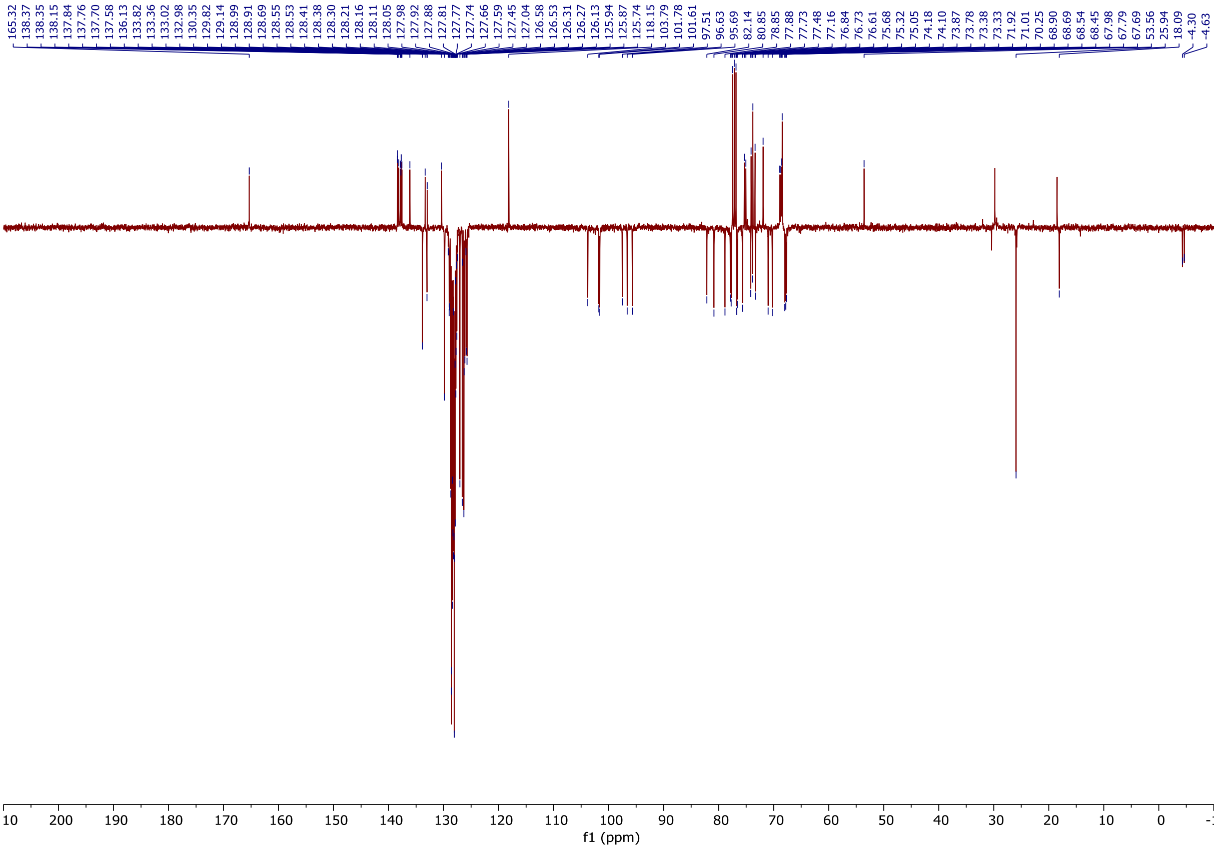

4,6-*O*-benzylidene-3-*O*-naphthyl-β-D-mannose-(1->3)-2-*O*-benzyl-4,6-*O*-benzylidene-β-D-mannose-(1->3)-2-*O*-benzoyl-4-*O*-benzyl-α-L-rhamnose-(1->3)-1-*O*-allyl-2,4,6-tri-*O*-benzyl-β-D-glucose (110)

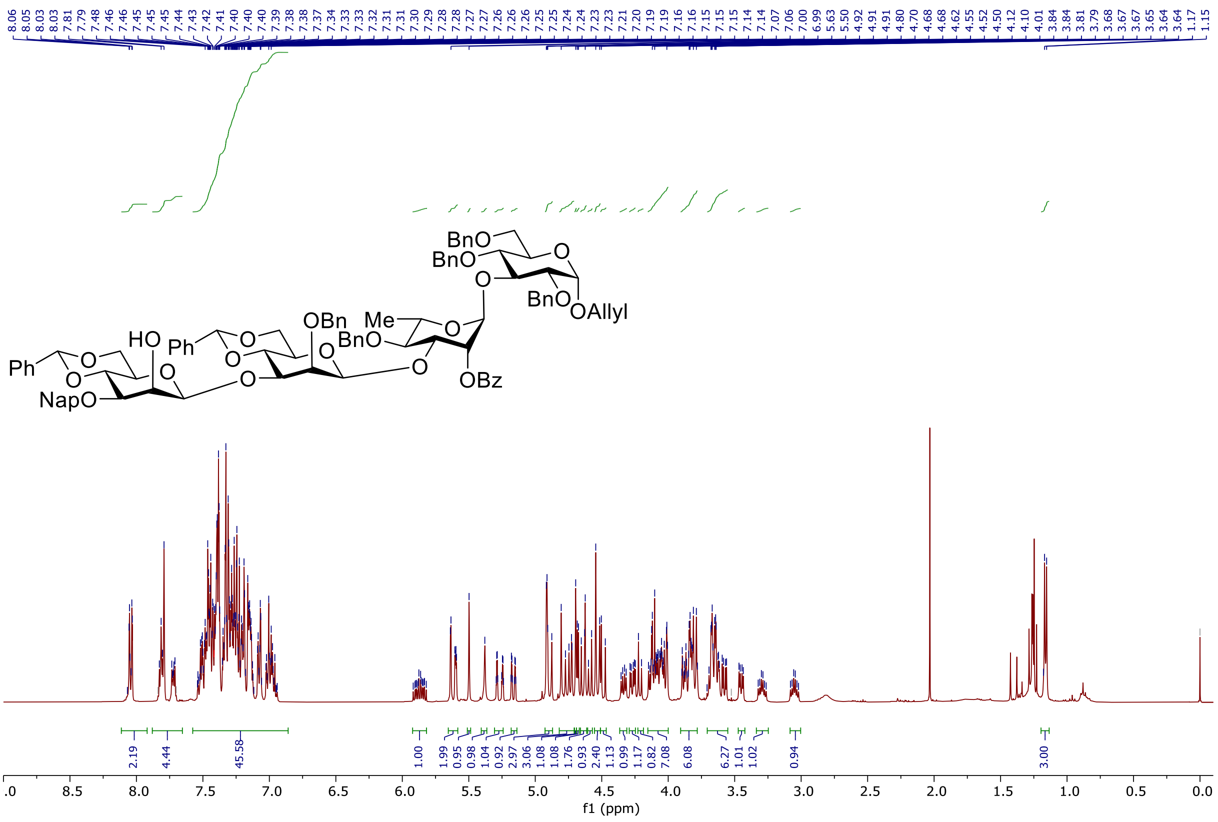

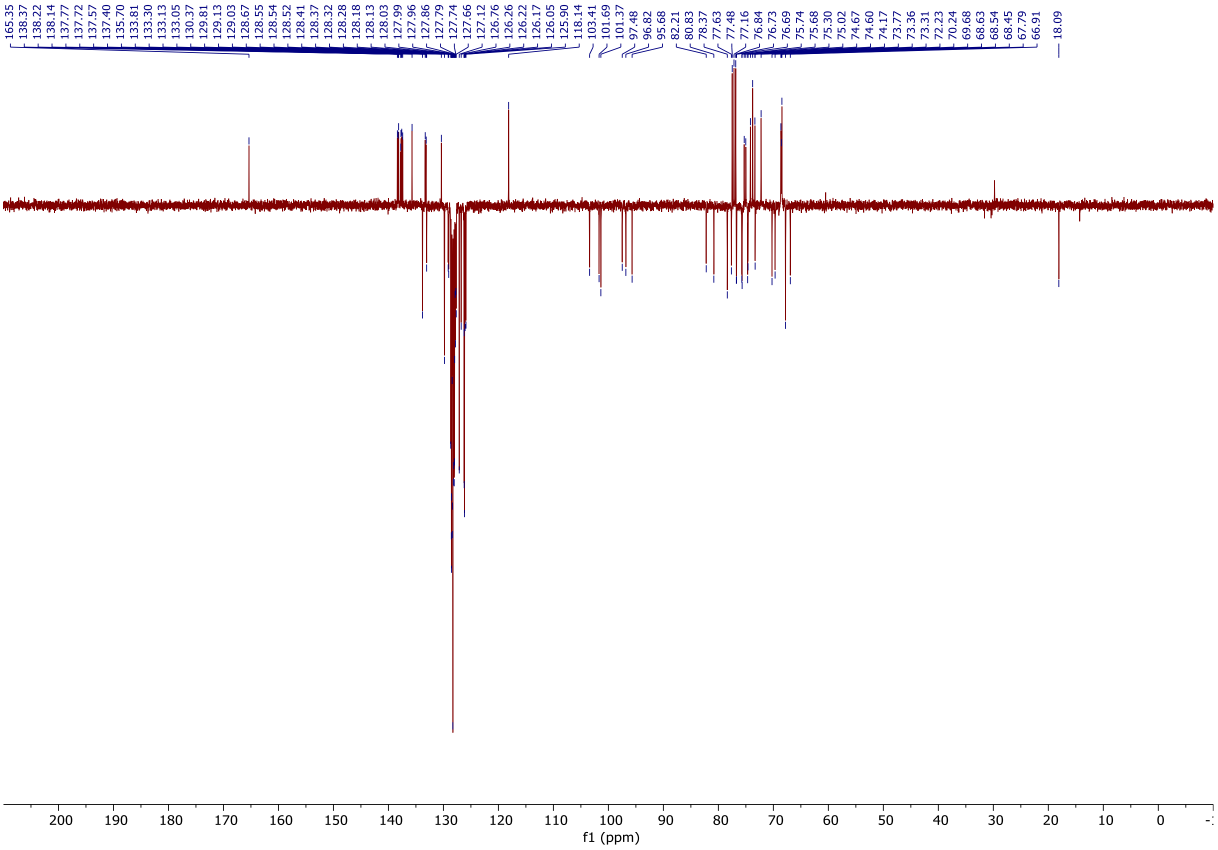

2,3,4,6-tetra-*O*-benzoyl-α-D-mannose-(1->2)-4,6-*O*-benzylidene-3-*O*-naphthyl-β-D-mannose-(1->3)-2-*O*-benzyl-4,6-*O*-benzylidene-β-D-mannose-(1->3)-2-*O*-benzoyl-4-*O*-benzyl-α-L-rhamnose-(1->3)-1-*O*-allyl-2,4,6-tri-*O*-benzyl-β-D-glucose (111)

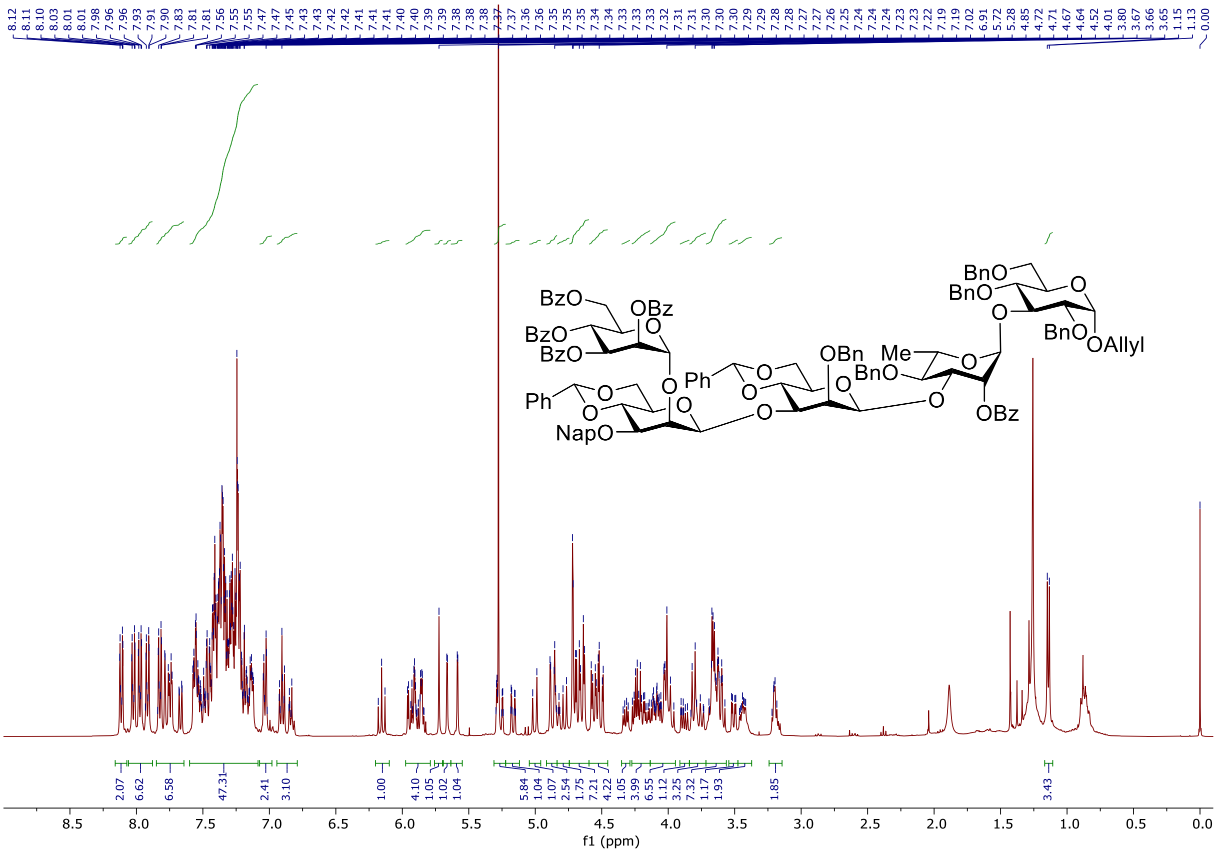

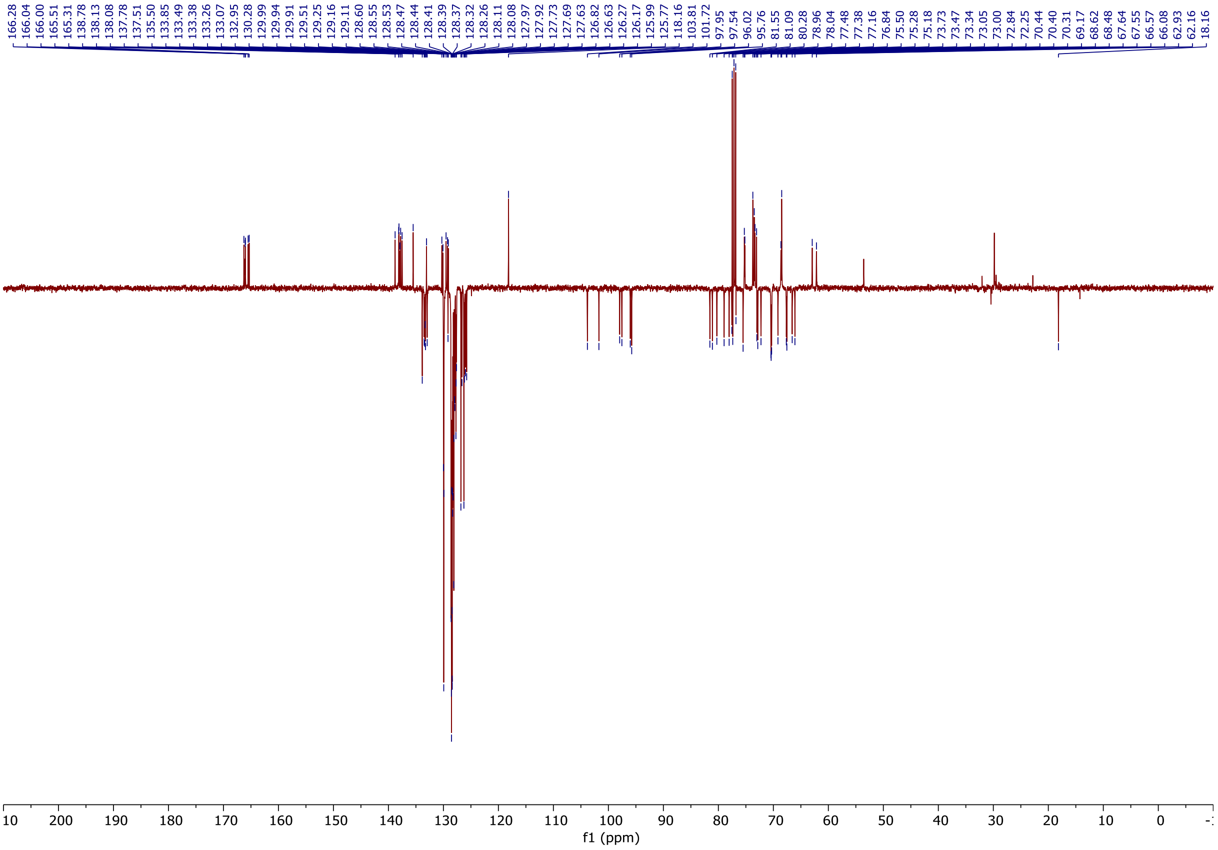

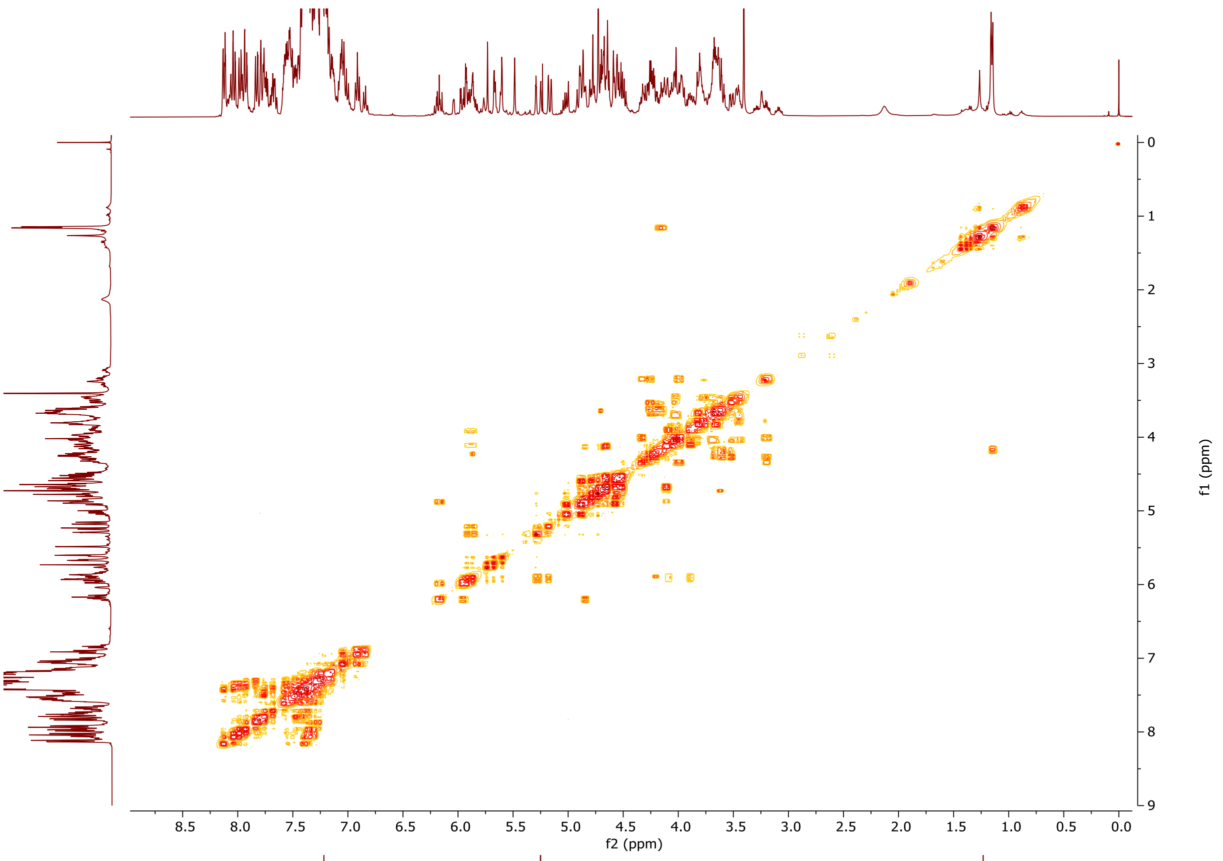
8.5 8.0 7.5 7.0 6.5 6.0 5.5 5.0 4.5 4.0 3.5 3.0 2.5 2.0 1.5 1.0 0.5 0.0
f2 (ppm)
0 1 2 3 4 5 6 7 8 9
f1 (ppm)

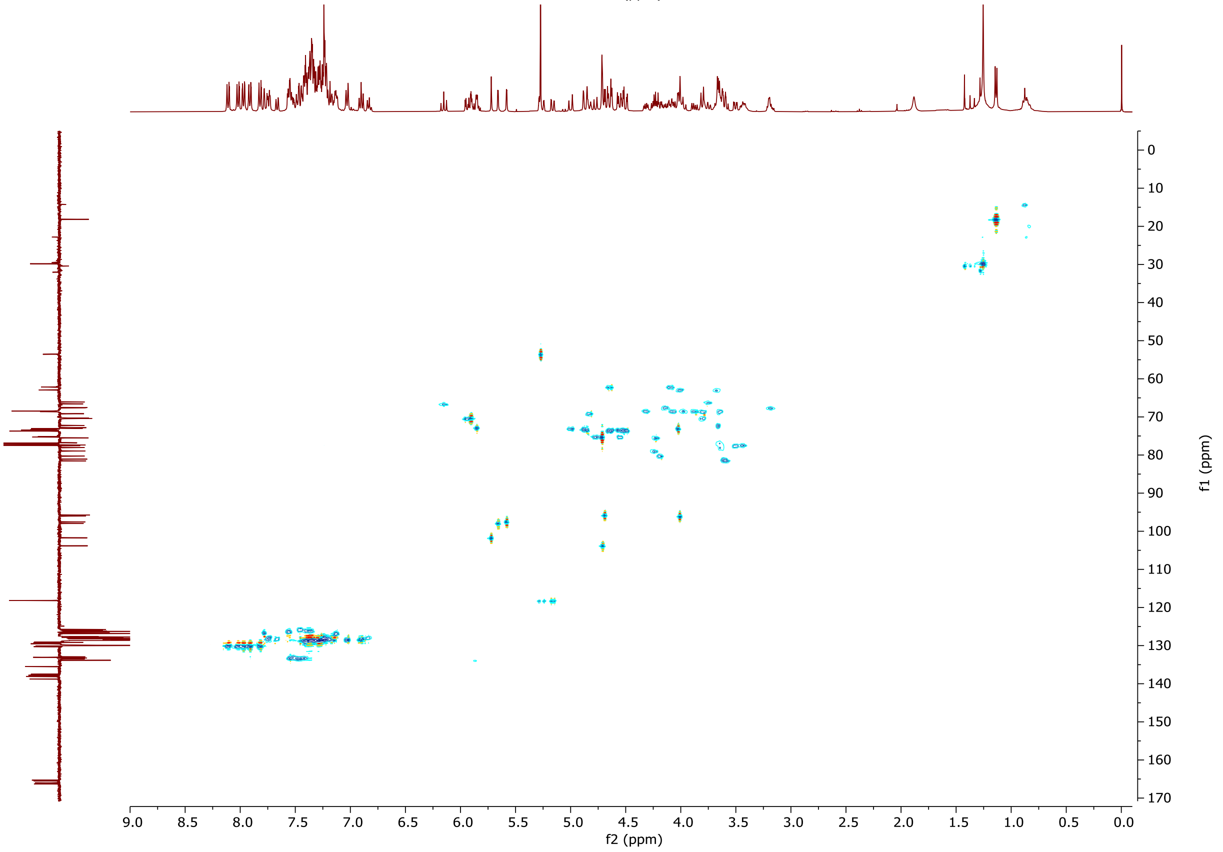
9.0 8.5 8.0 7.5 7.0 6.5 6.0 5.5 5.0 4.5 4.0 3.5 3.0 2.5 2.0 1.5 1.0 0.5 0.0
f2 (ppm)
0 10 20 30 40 50 60 70 80 90 100 110 120 130 140 150 160 170
f1 (ppm)

α-D-mannose-(1->2)-4,6-*O*-benzylidene-3-*O*-naphthyl-β-D-mannose-(1->3)-2-*O*-benzyl-4,6-*O*-benzylidene-β-D-mannose-(1->3)-4-*O*-benzyl-α-L-rhamnose-(1->3)-1-*O*-allyl-2,4,6-tri-*O*-benzyl-β-D-glucose (112a)

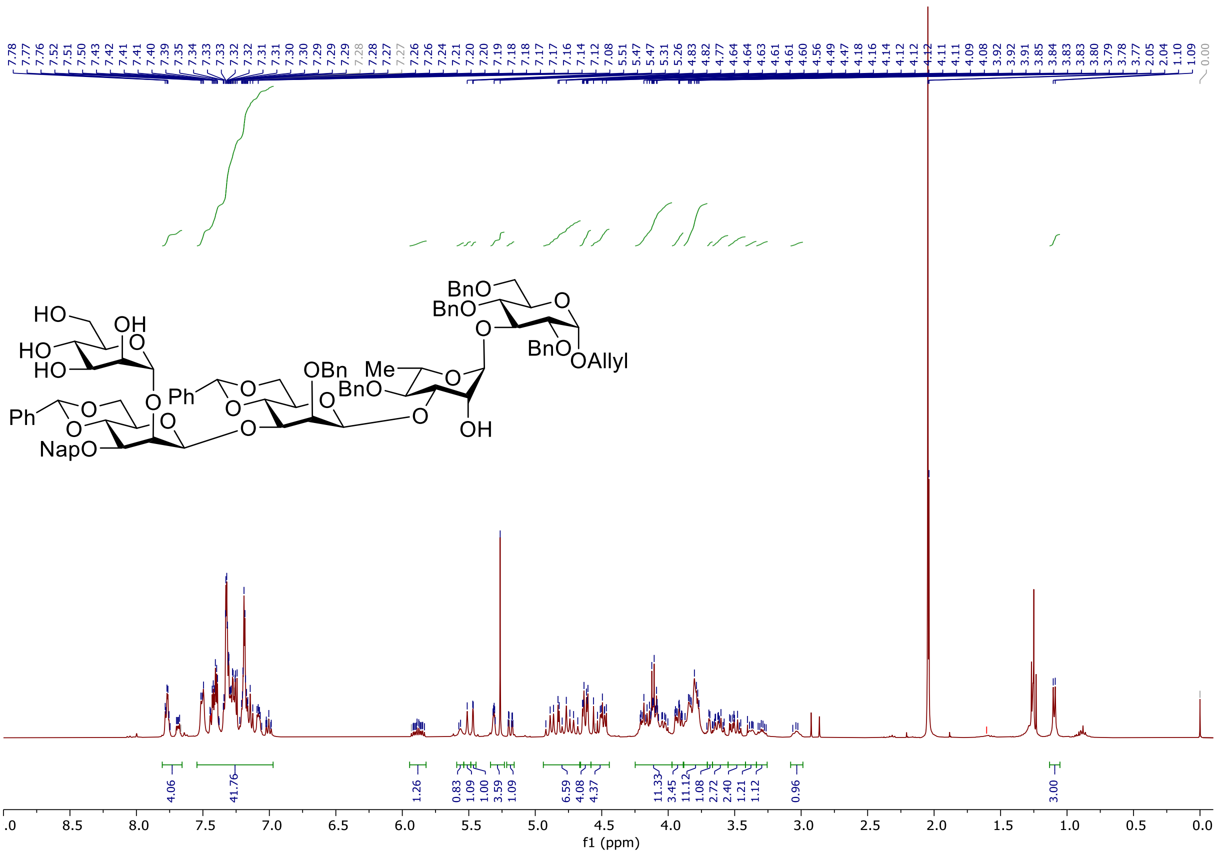

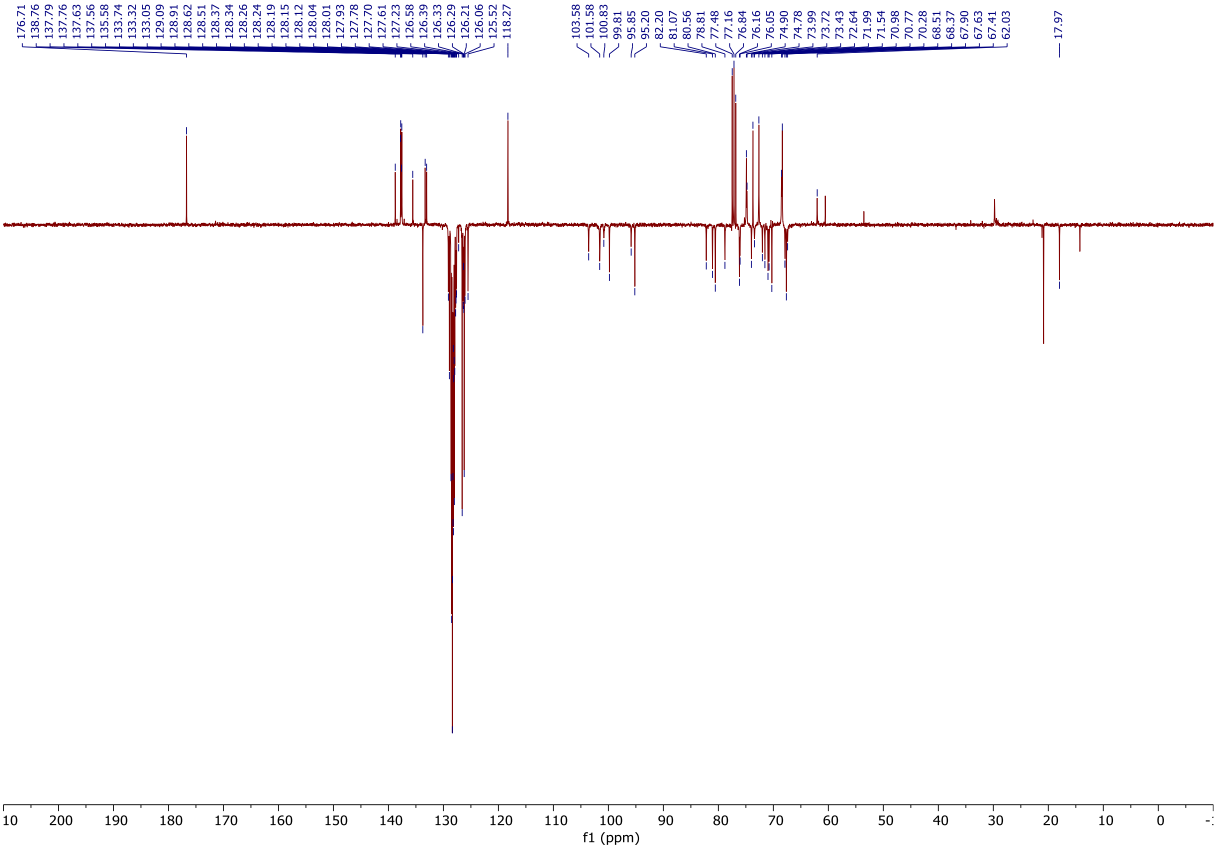

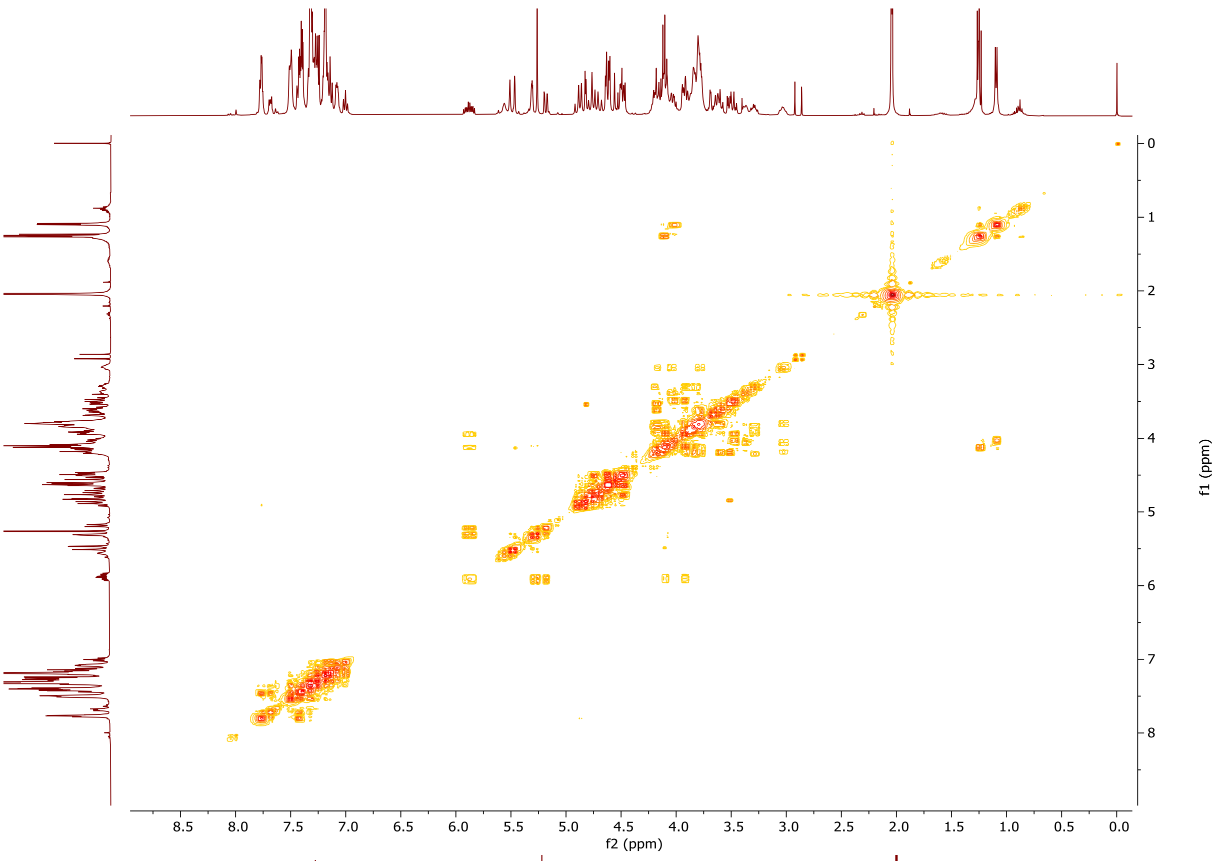
f1 (ppm)
f2 (ppm)

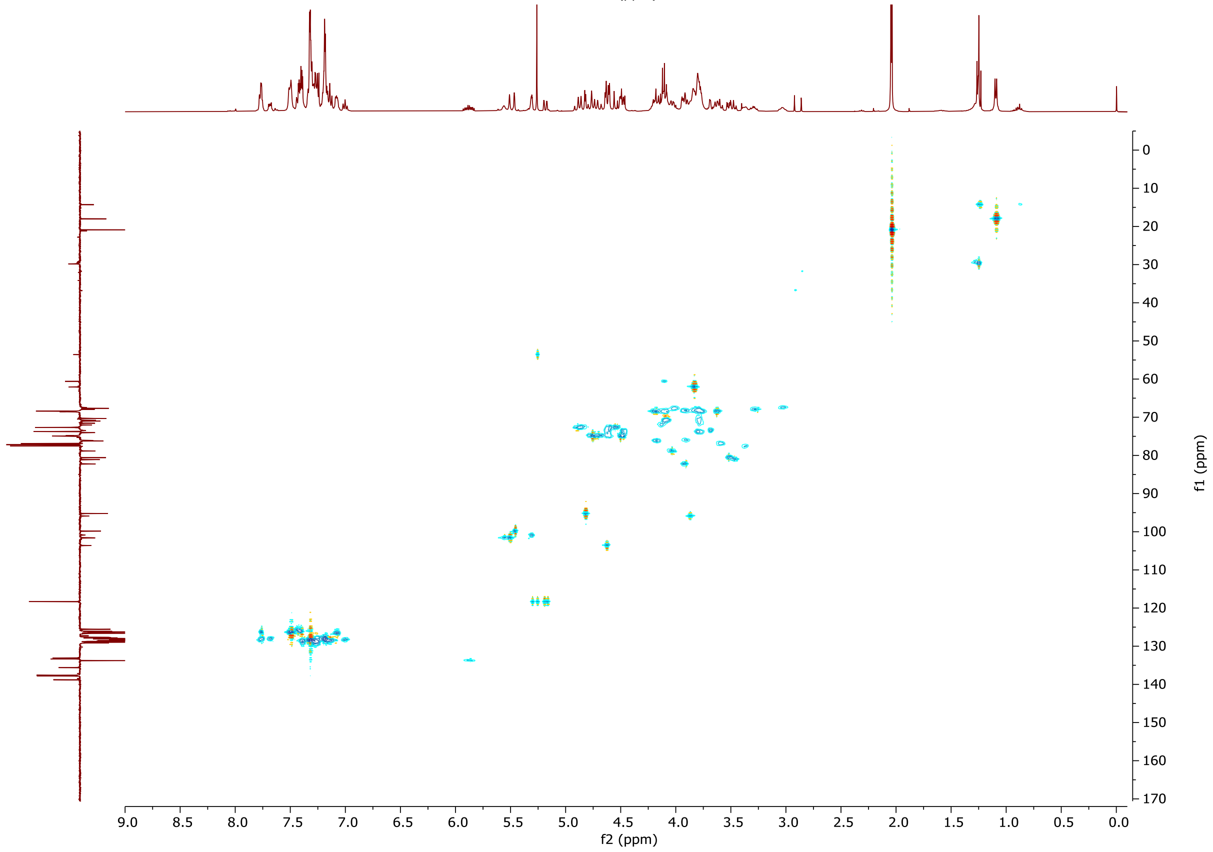
f1 (ppm)
f2 (ppm)

2,3,4,6-tetra-*O*-benzyl-α-ᴅ-mannose-(1->2)-4,6-*O*-benzylidene-3-*O*-naphthyl-β-ᴅ-mannose-(1->3)-2-*O*-benzyl-4,6-*O*-benzylidene-β-ᴅ-mannose-(1->3)-2-*O*-benzyl-4-*O*-benzyl-α-ʟ-rhamnose-(1->3)-1-*O*-allyl-2,4,6-tri-*O*-benzyl-β-ᴅ-glucose (112)

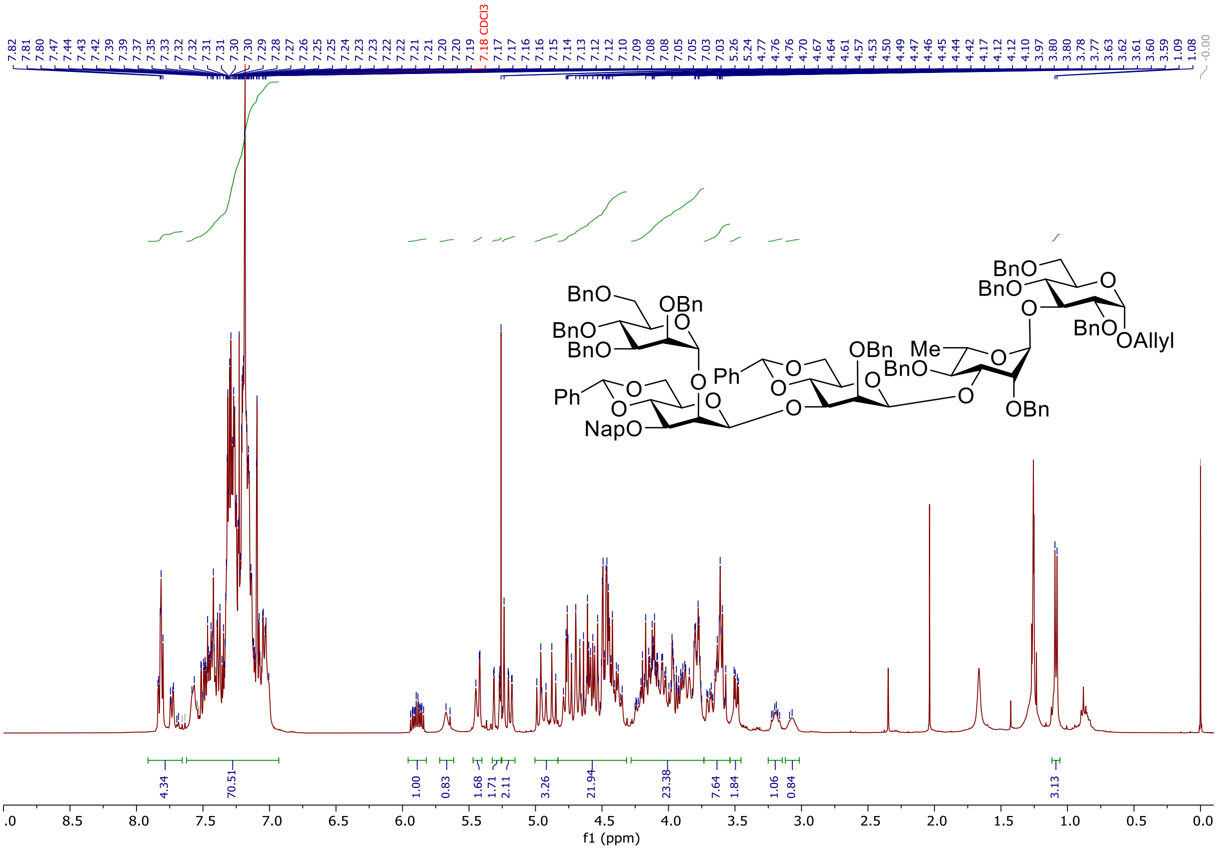

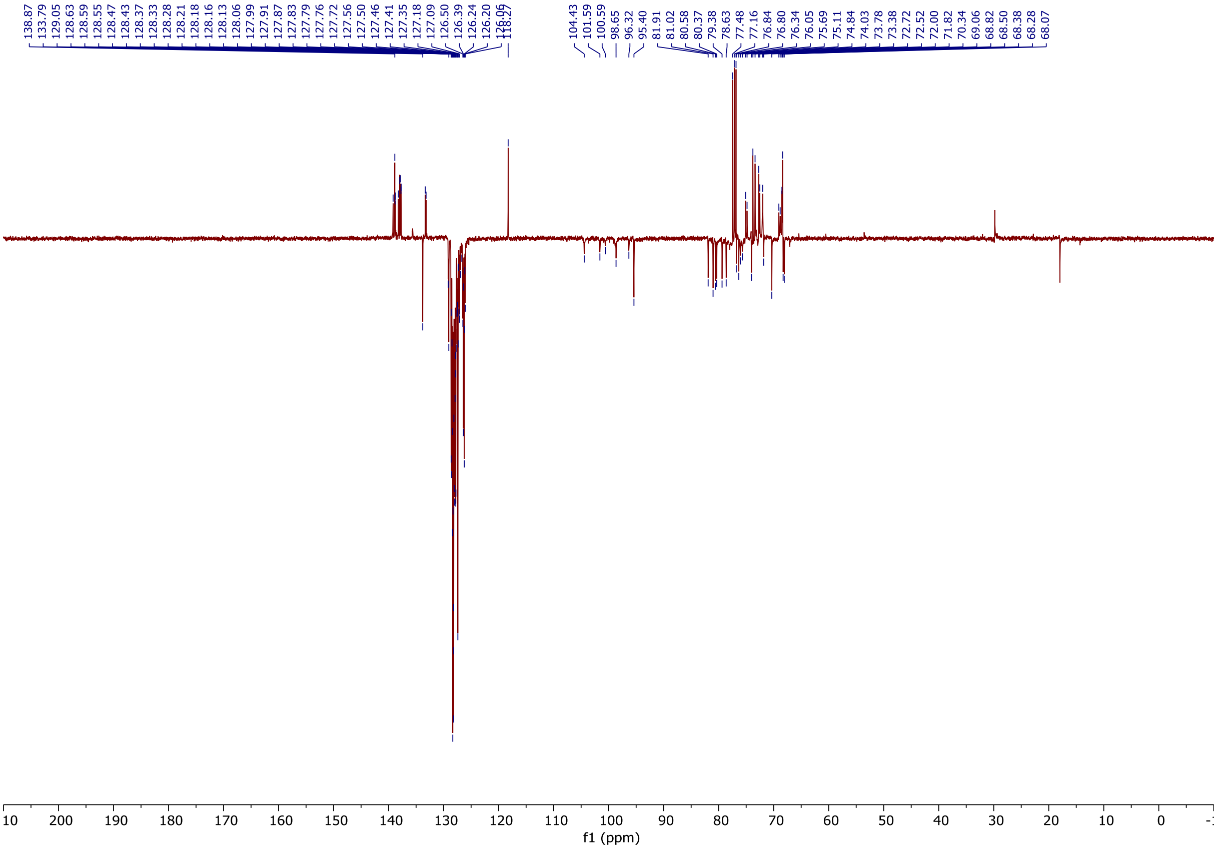

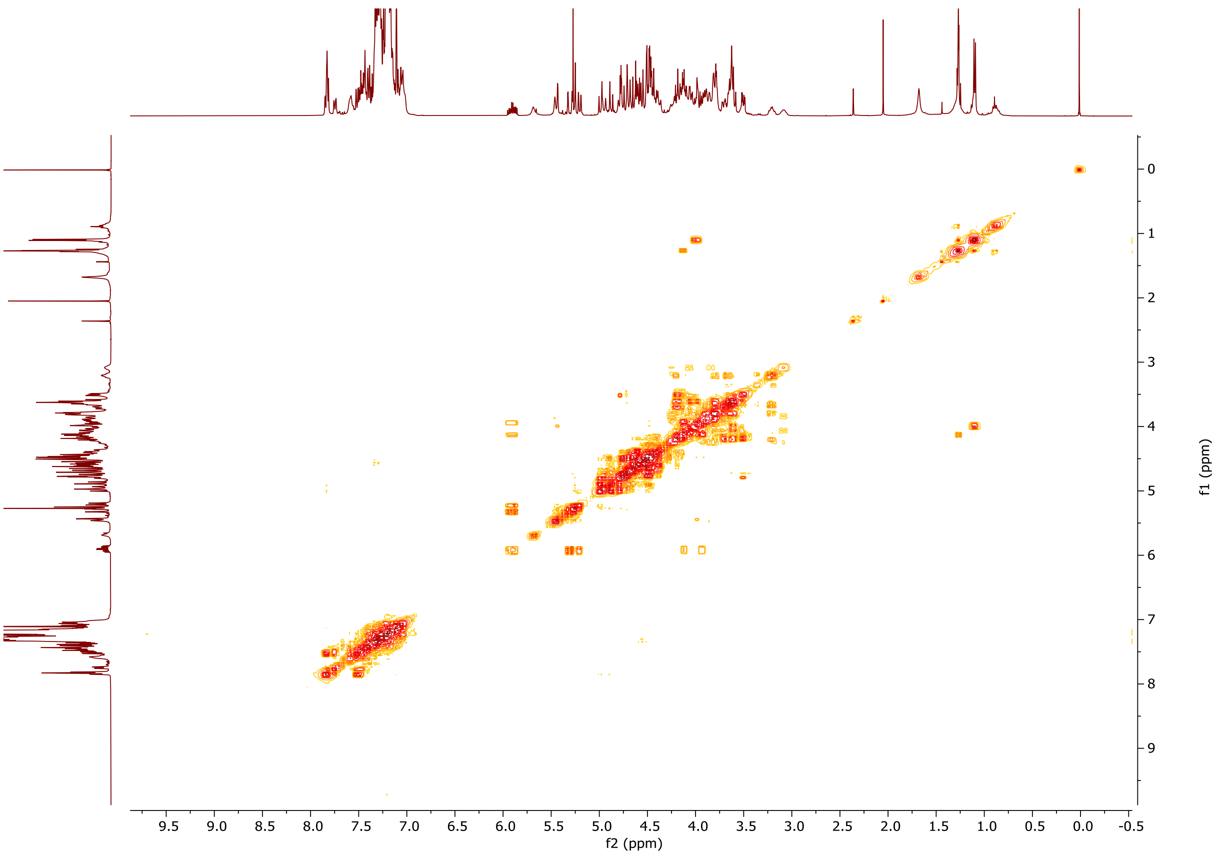
f2 (ppm)
f1 (ppm)

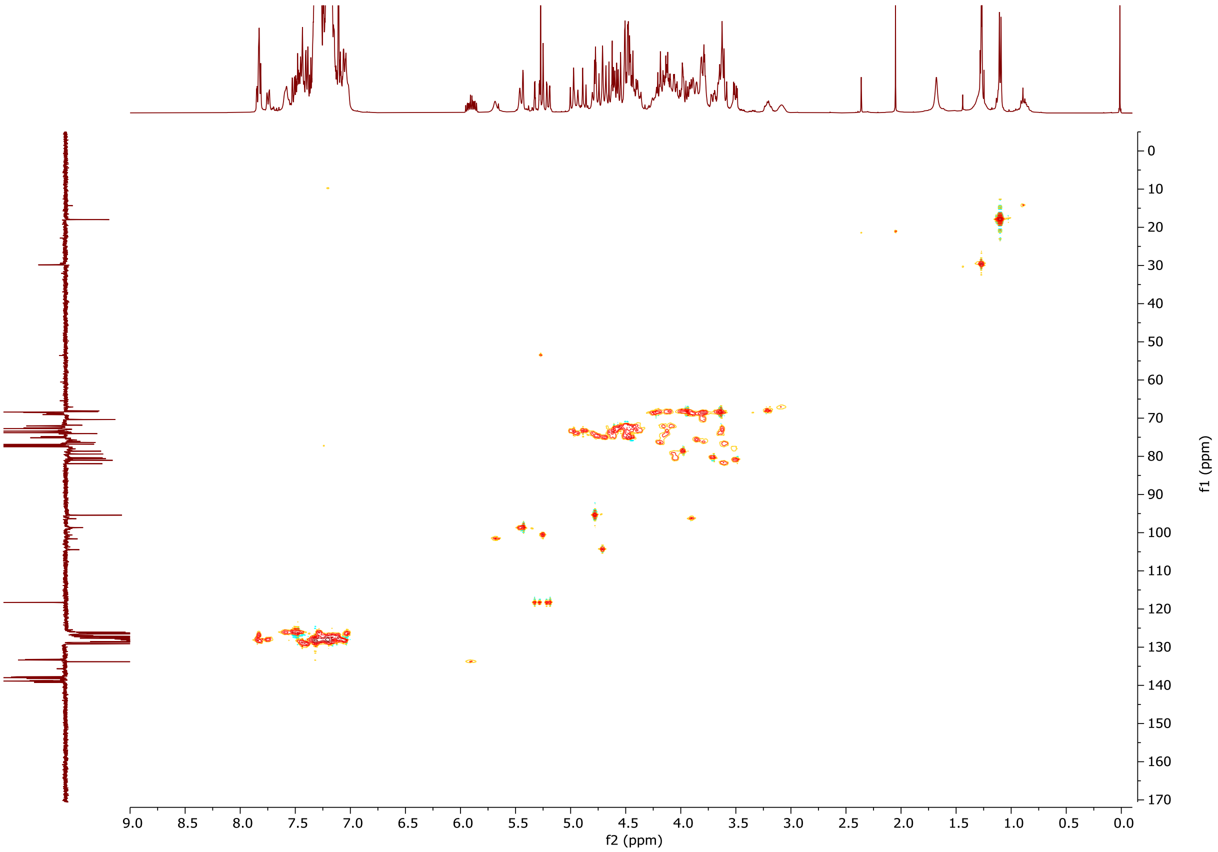
f2 (ppm)
f1 (ppm)

2,3,4,6-tetra-*O*-benzyl-α-ᴅ-mannose-(1->2)-4,6-*O*-benzylidene-3-*O*-naphthyl-β-ᴅ-mannose-(1->3)-2-*O*-benzyl-4,6-*O*-benzylidene-β-ᴅ-mannose-(1->3)-2-*O*-benzyl-4-*O*-benzyl-α-ʟ-rhamnose-(1->3)-2,4,6-tri-*O*-benzyl-β-ᴅ-glucose (21)

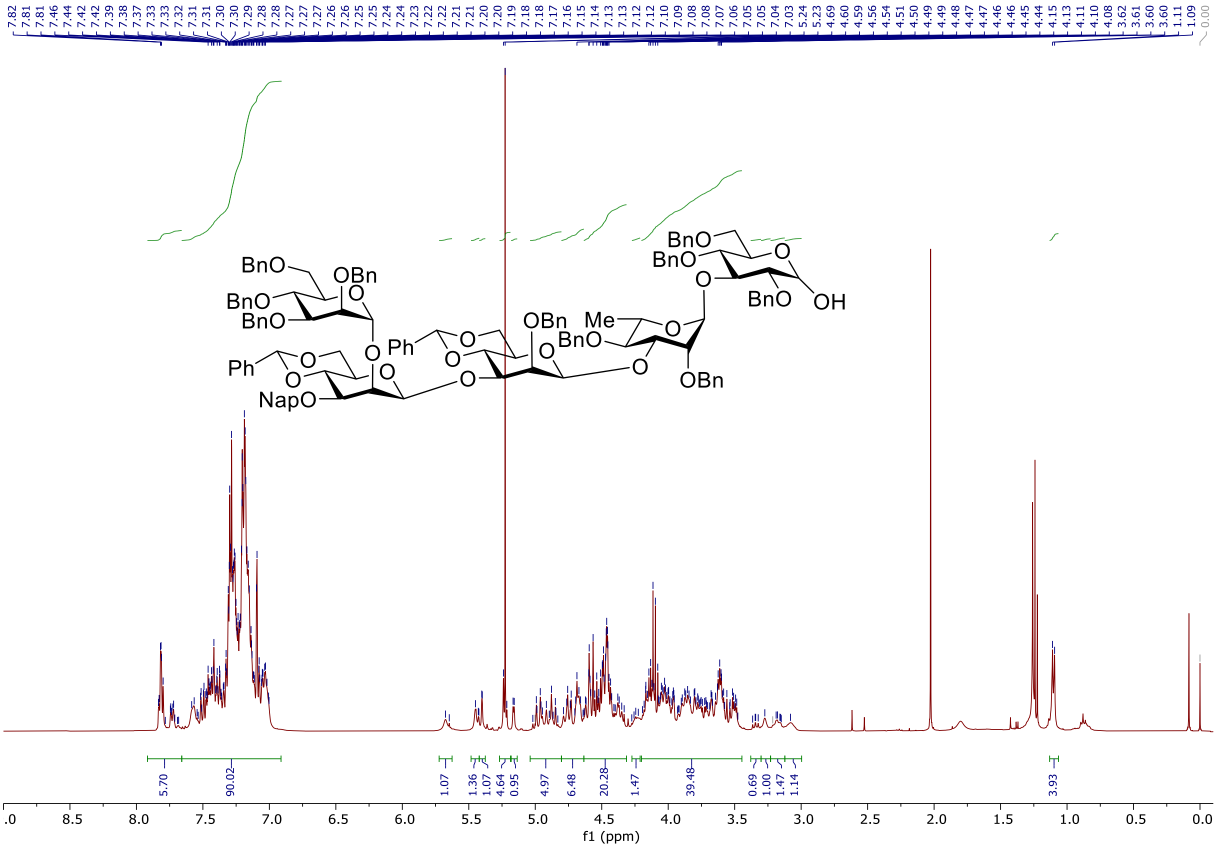

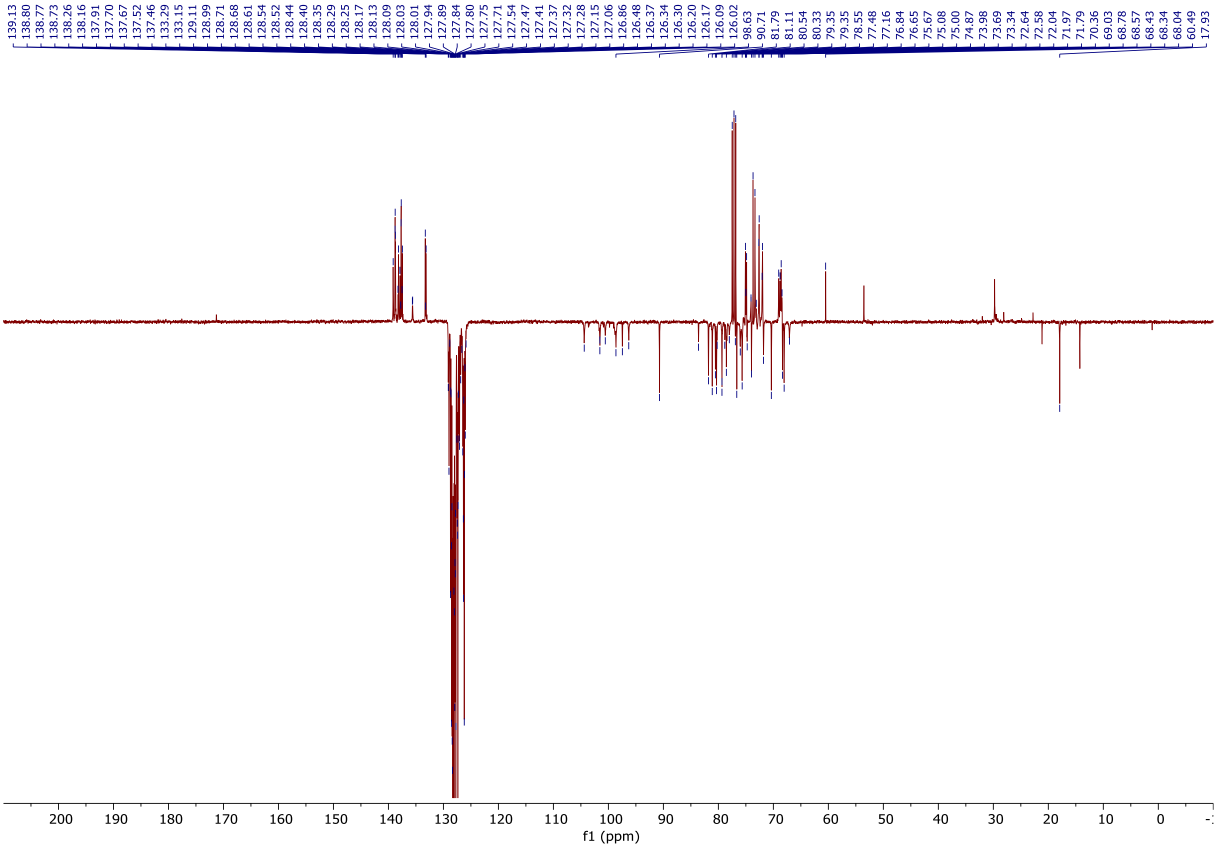

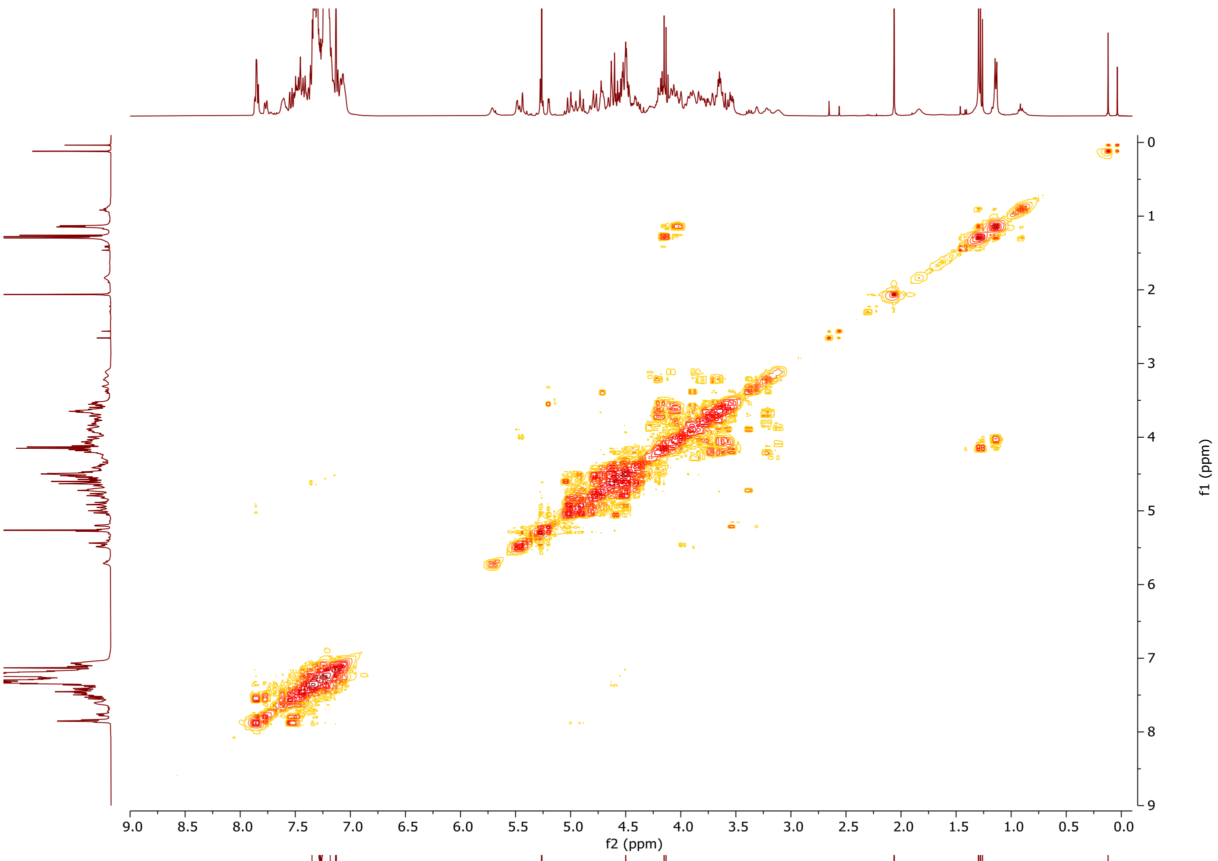
9.0
8.5
8.0
7.5
7.0
6.5
6.0
5.5
5.0
4.5
4.0
3.5
3.0
2.5
2.0
1.5
1.0
0.5
0.0
f2 (ppm)
f1 (ppm)

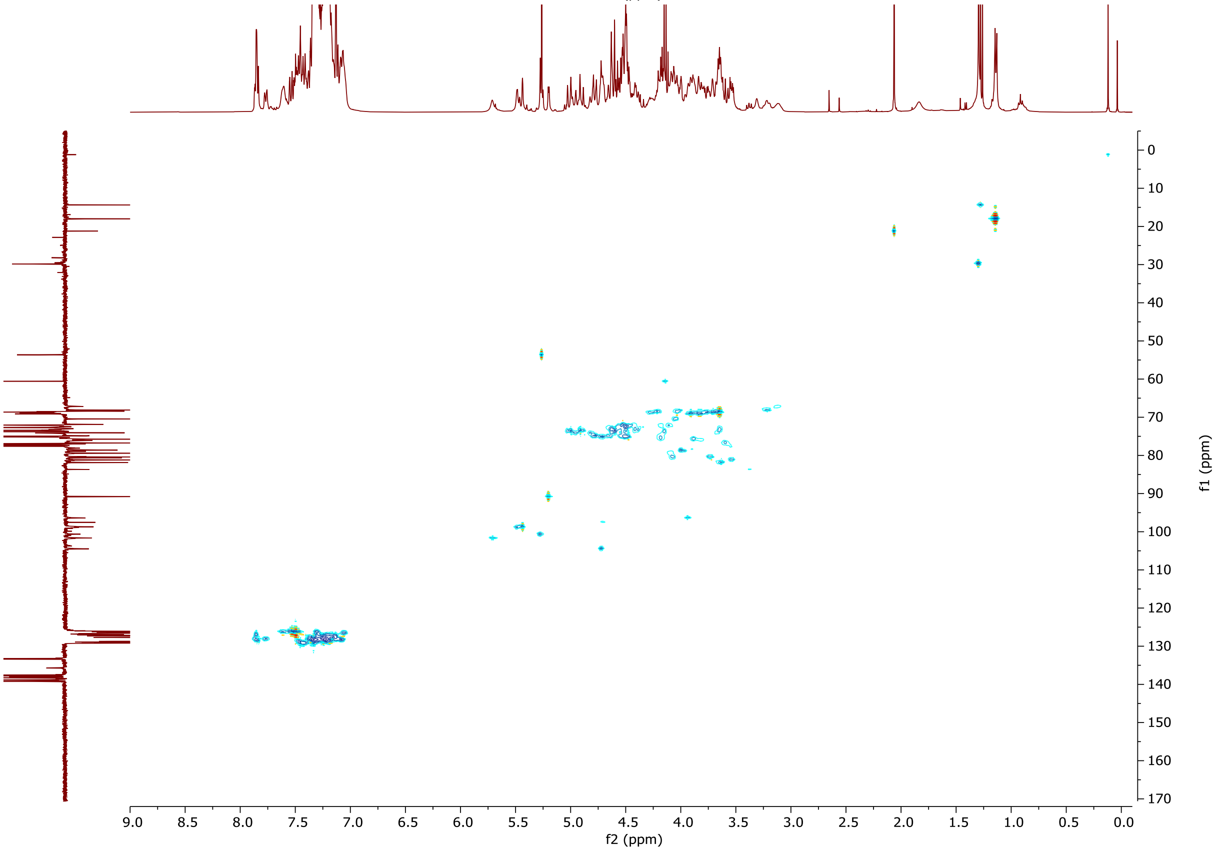
9.0
8.5
8.0
7.5
7.0
6.5
6.0
5.5
5.0
4.5
4.0
3.5
3.0
2.5
2.0
1.5
1.0
0.5
0.0
f2 (ppm)
f1 (ppm)

2,3,4,6-tetra-*O*-benzyl-α-ᴅ-mannose-(1->2)-4,6-*O*-benzylidene-3-*O*-naphthyl-β-ᴅ-mannose-(1->3)-2-*O*-benzyl-4,6-*O*-benzylidene-β-ᴅ-mannose-(1->3)-2-*O*-benzyl-4-*O*-benzyl-α-ʟ-rhamnose-(1->3)-2,4,6-tri-*O*-benzyl-β-ᴅ-glucitol (22)

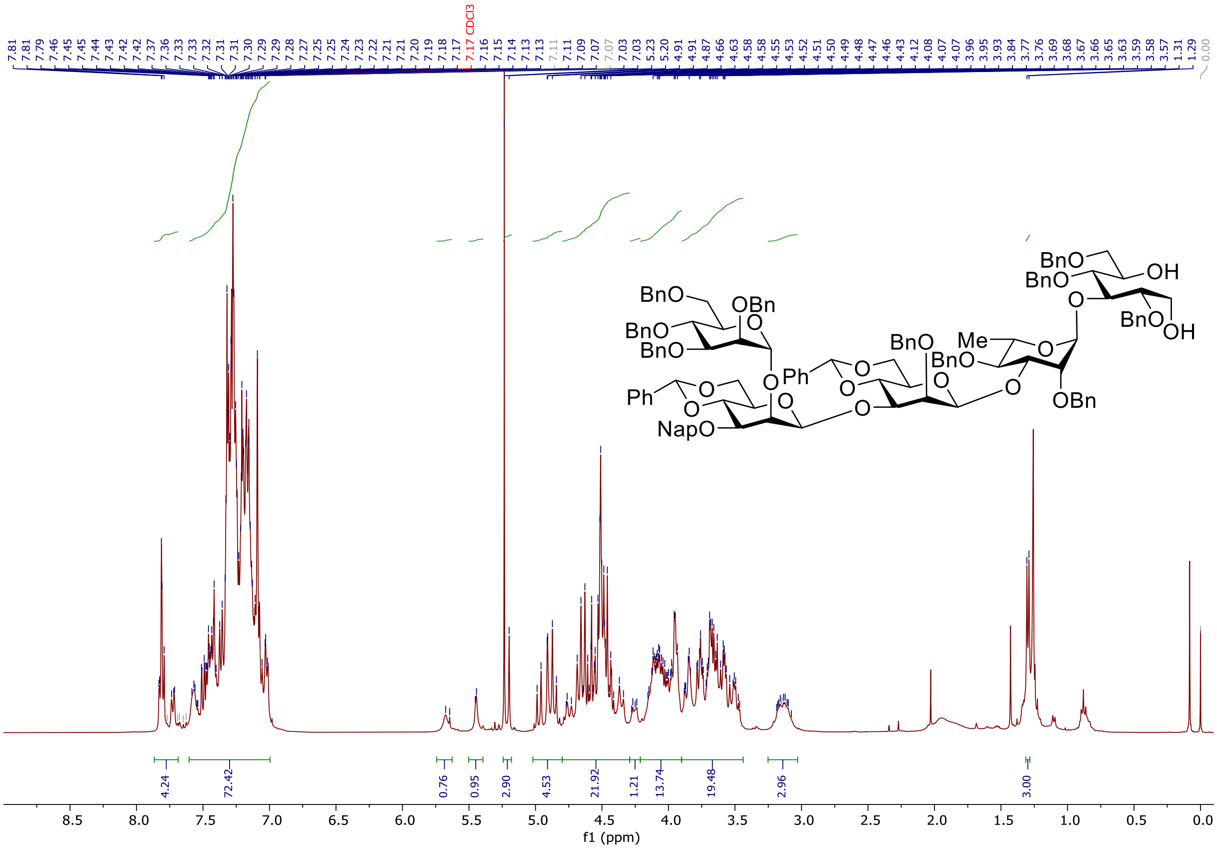

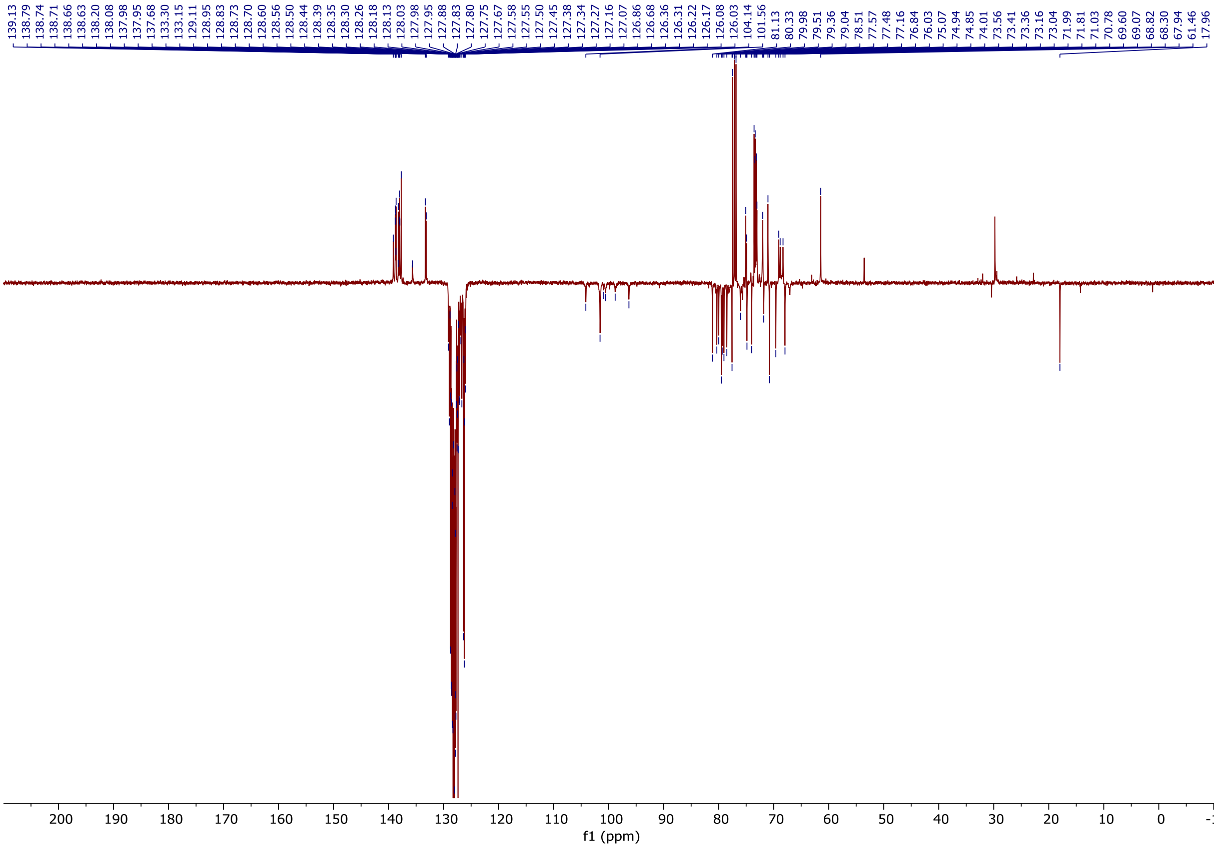

2,3,4,6-tetra-*O*-benzyl-α-ᴅ-mannose-(1->2)-4,6-*O*-benzylidene-3-*O*-naphthyl-β-ᴅ-mannose-(1->3)-2-*O*-benzyl-4,6-*O*-benzylidene-β-ᴅ-mannose-(1->3)-2-*O*-benzyl-4-*O*-benzyl-α-ʟ-rhamnose-(1->3)-2,4,6-tri-*O*-benzyl-1-deoxynojirimycin (24)

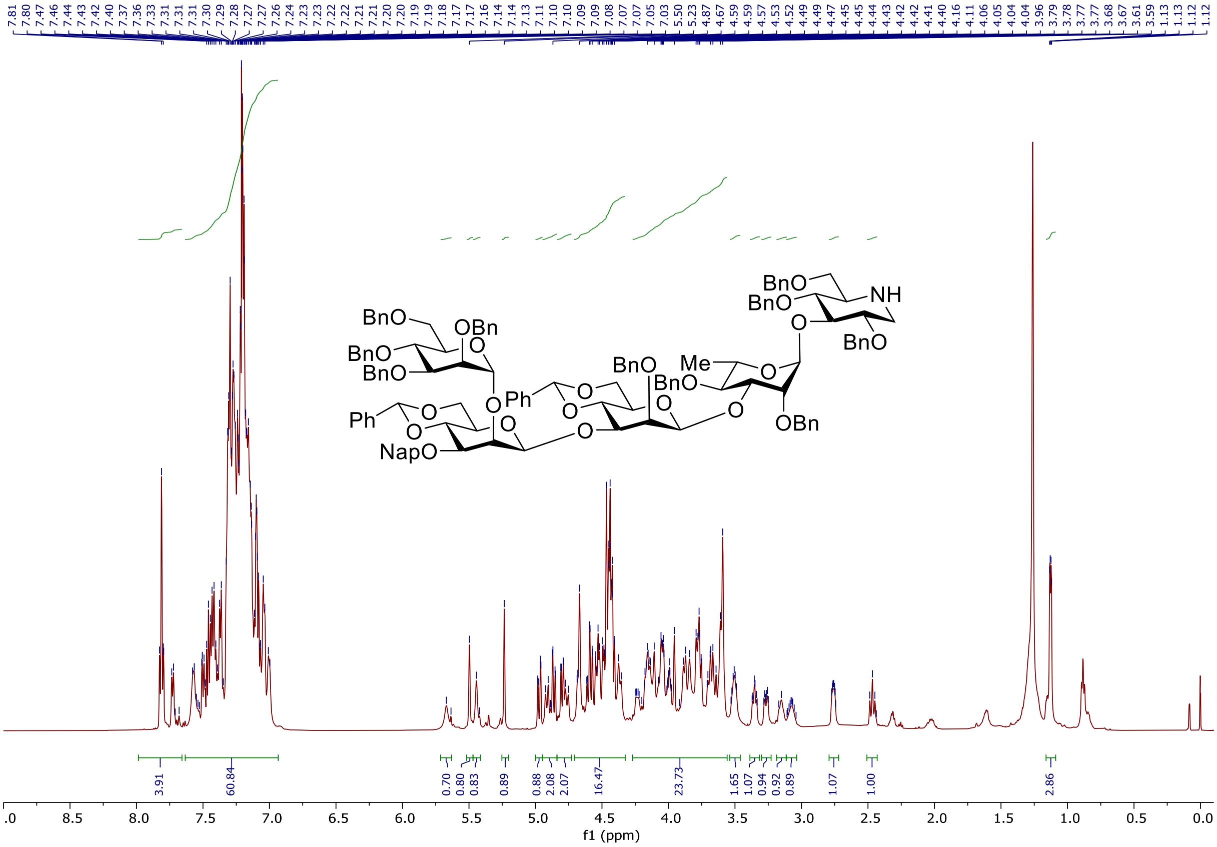

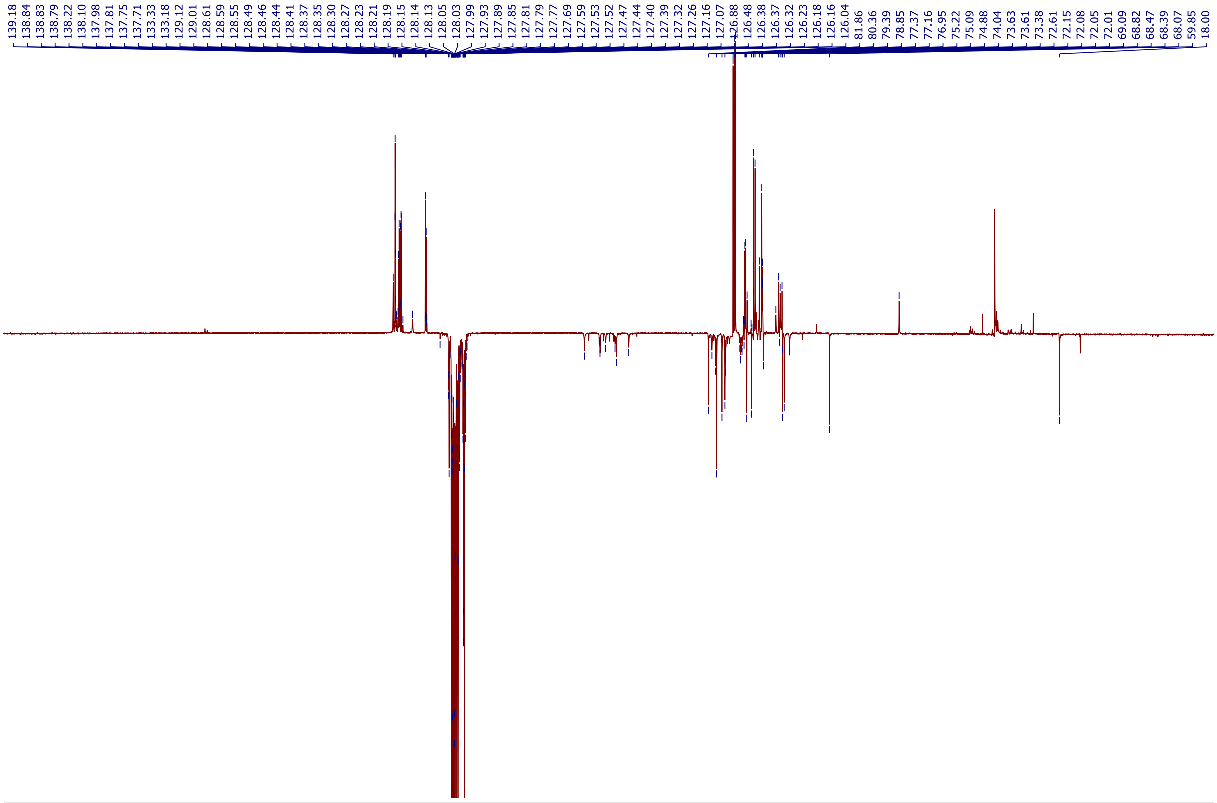

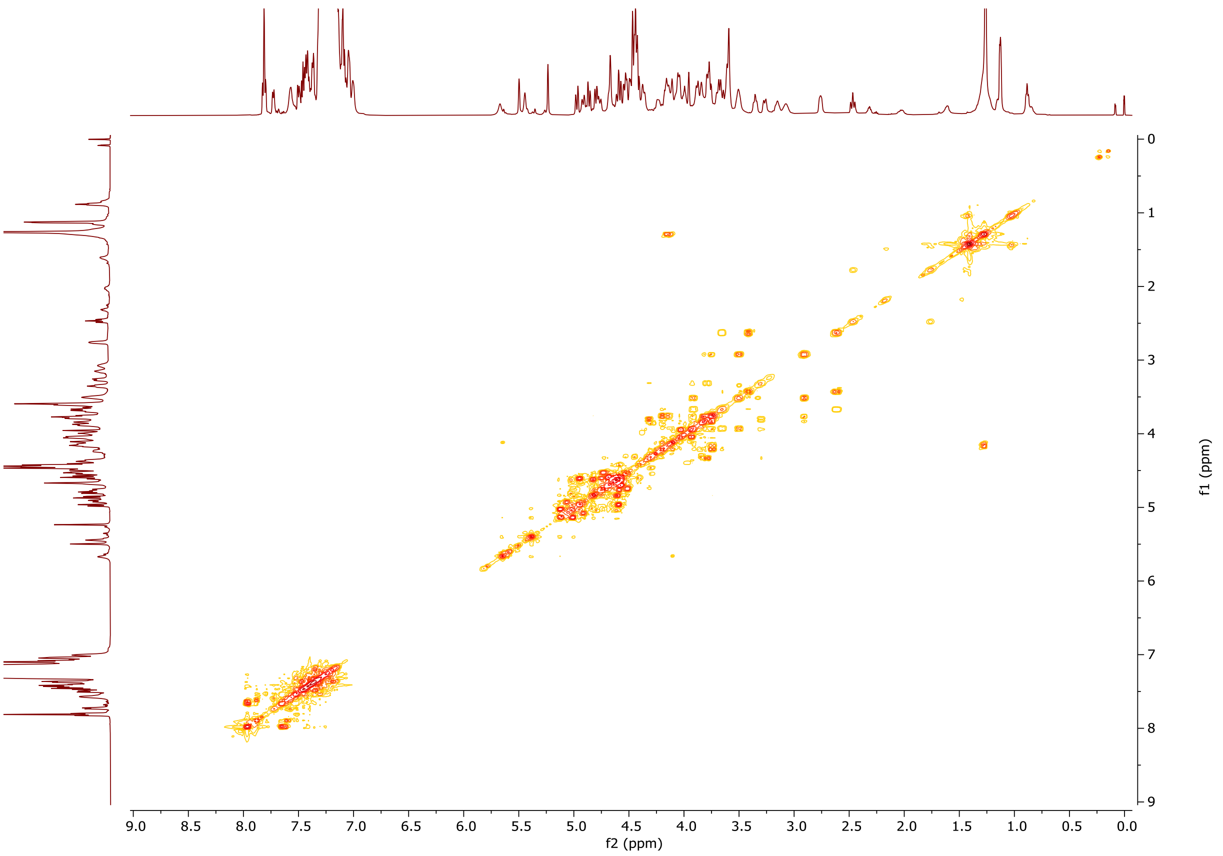
f2 (ppm)
f1 (ppm)

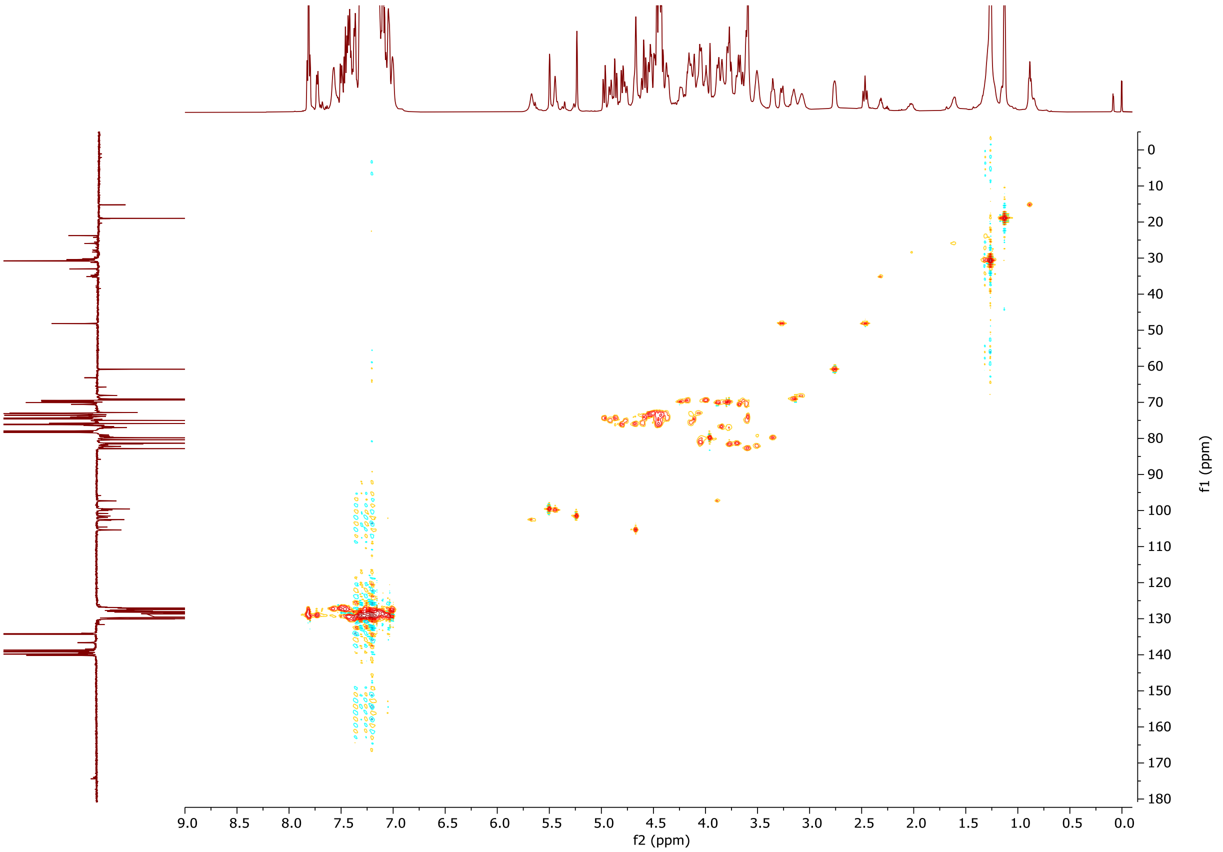
f2 (ppm)
f1 (ppm)

α-ᴅ-mannose-(1->2)-β-ᴅ-mannose-(1->3)-β-ᴅ-mannose-(1->3)-α-ʟ-rhamnose-(1->3)-1-deoxynojirimycin (25)

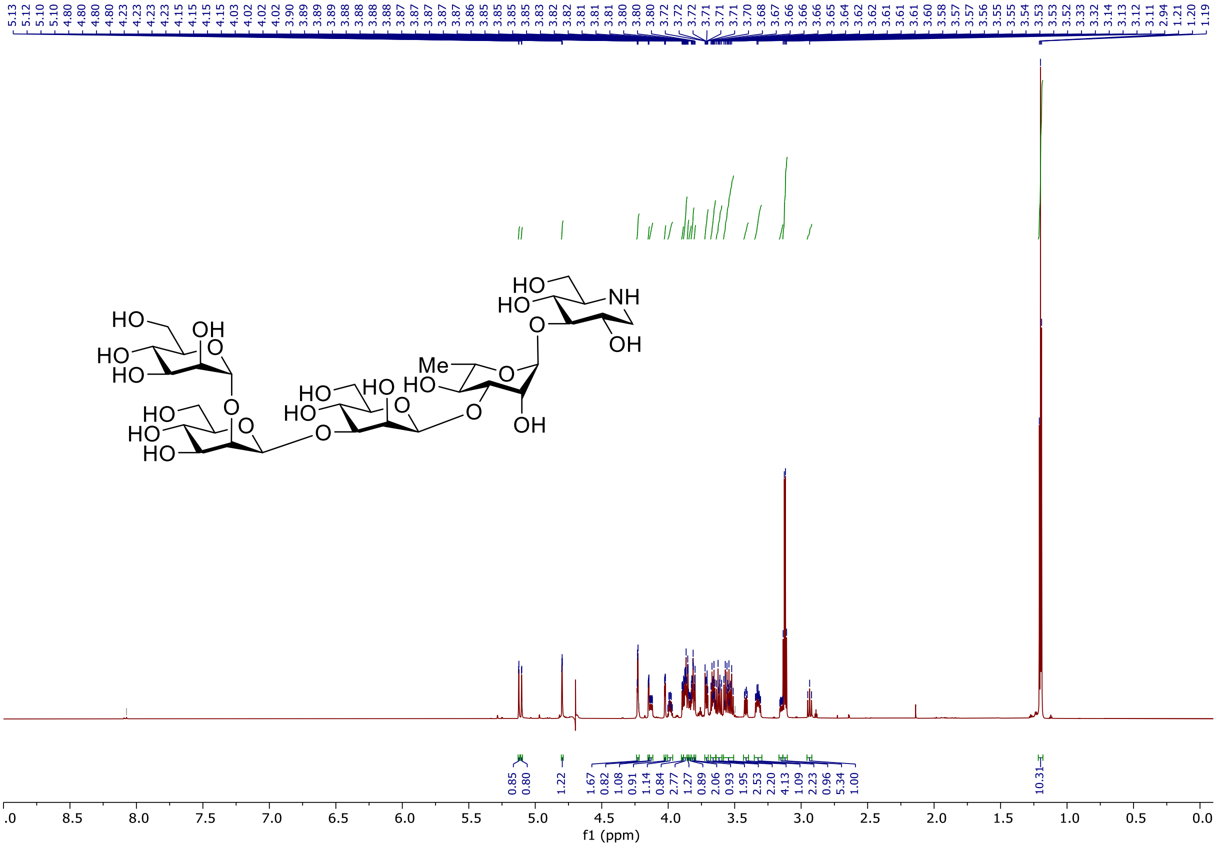

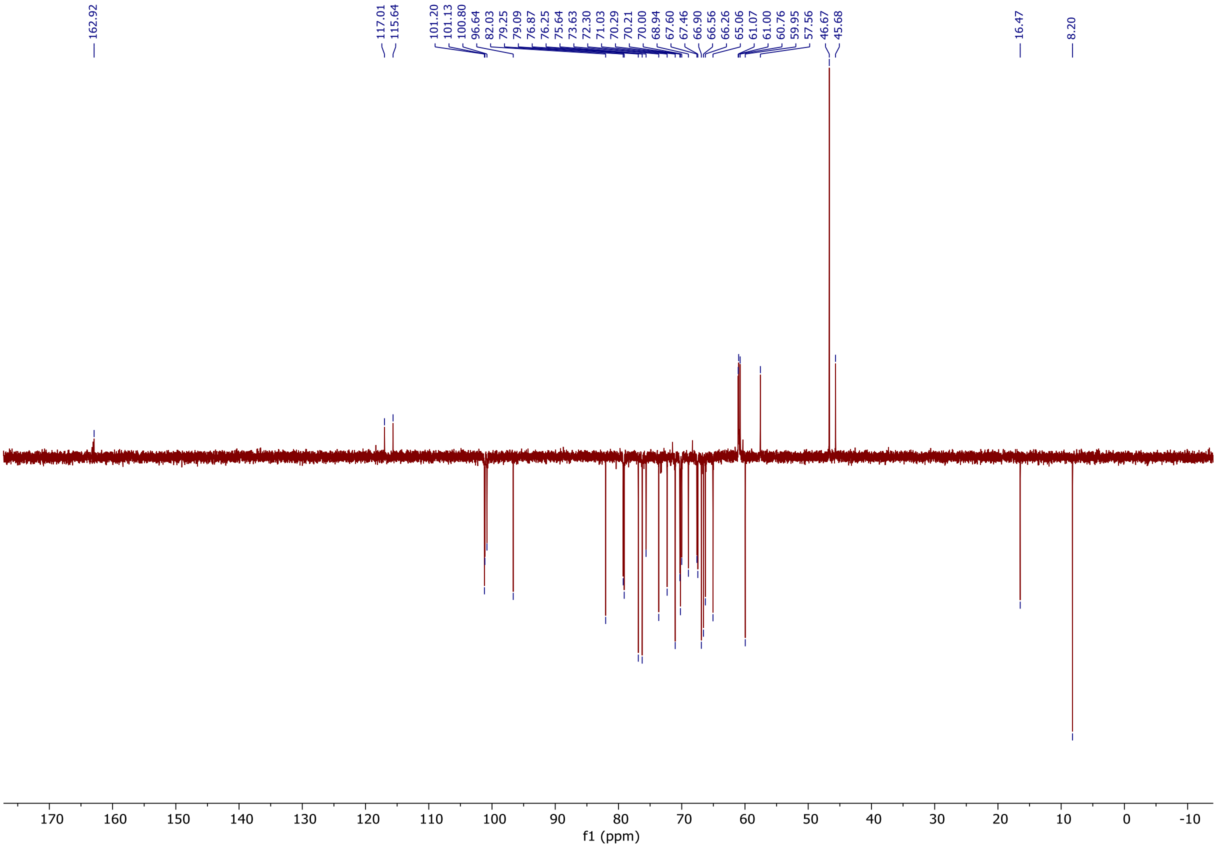

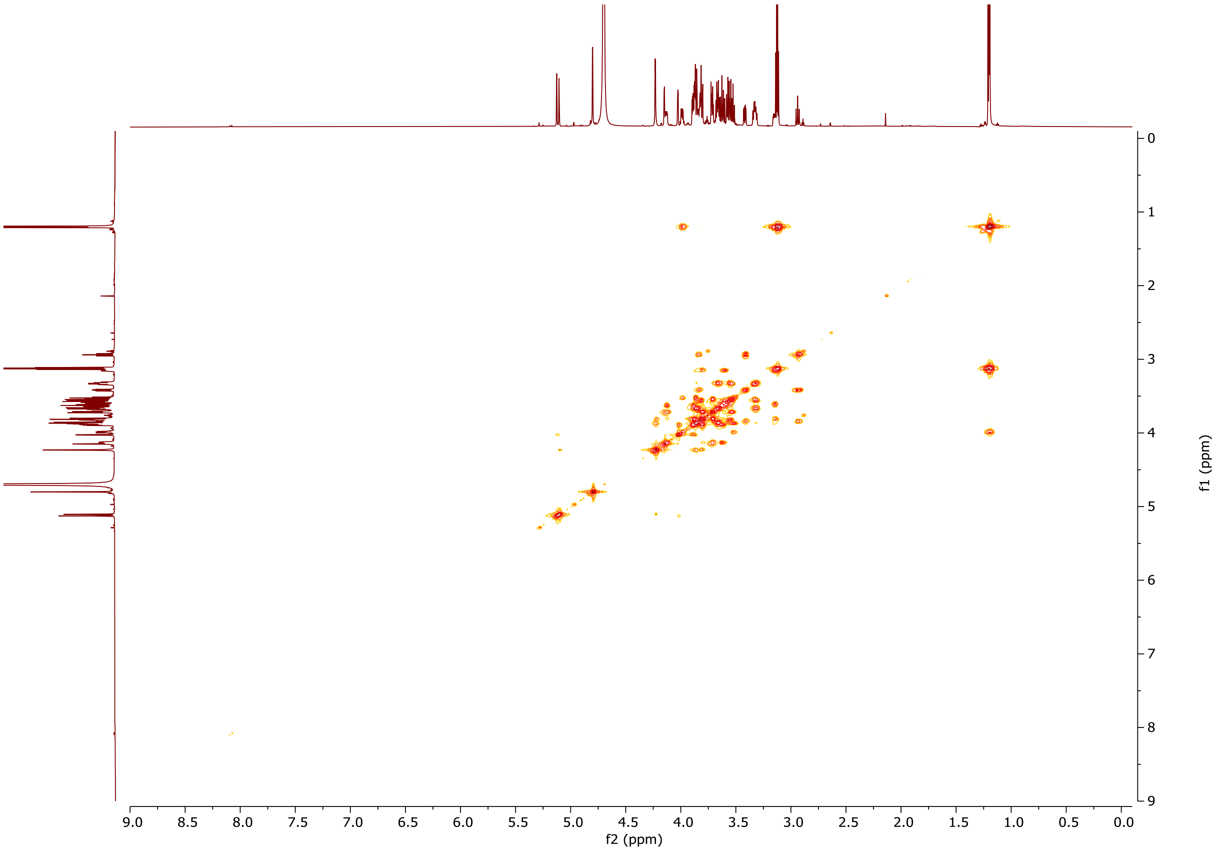
f1 (ppm)
f2 (ppm)

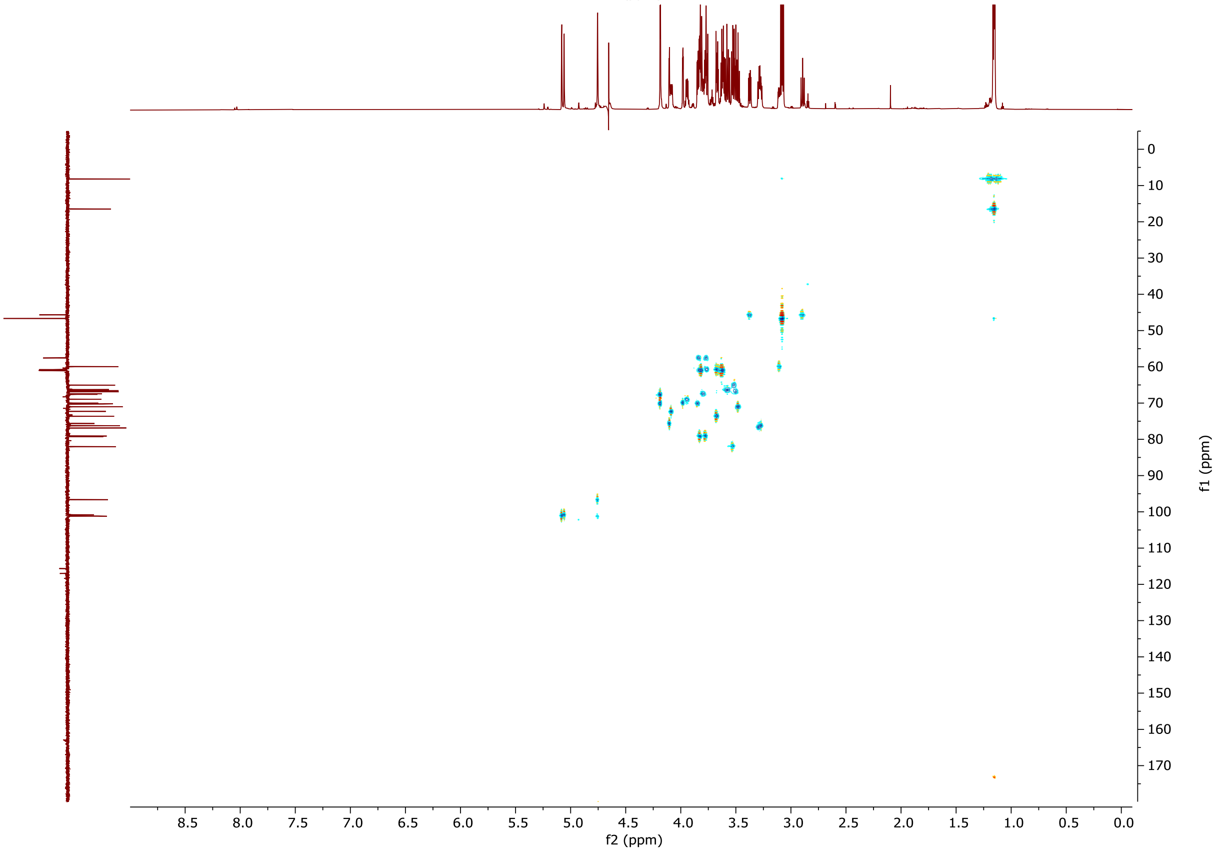
f1 (ppm)
f2 (ppm)

Compound **25** with a reduced amount of counterion.

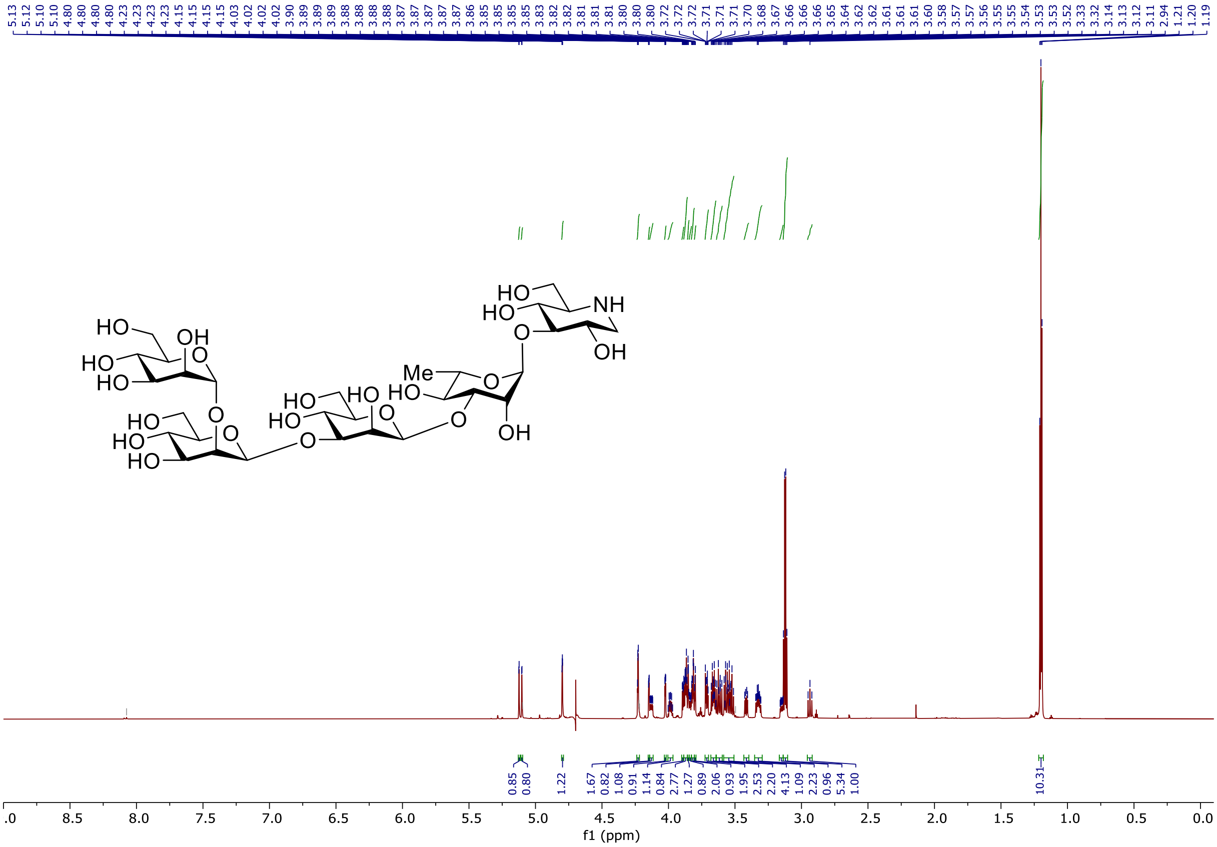

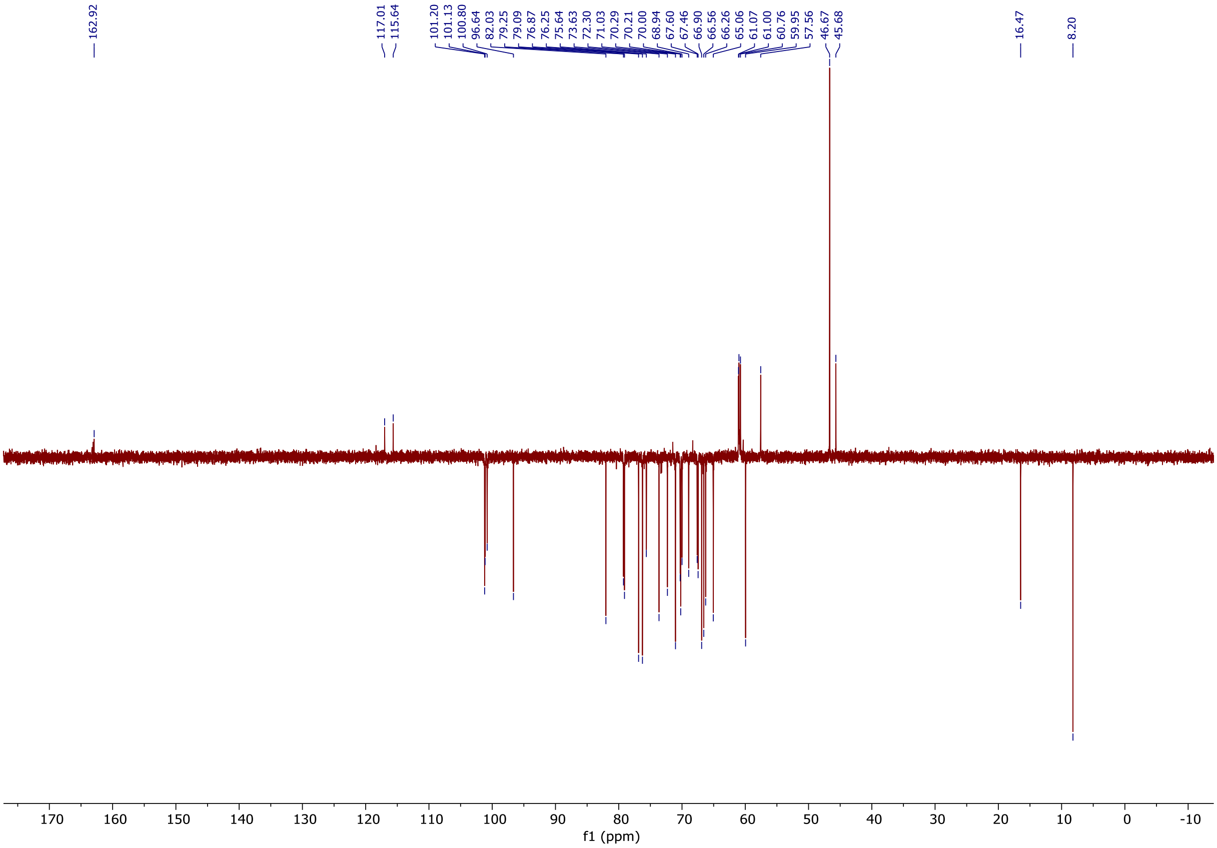

Supplement: Supplementary file 1 — ja4c16806_si_001.pdf [file ja4c16806_si_001.pdf]
